# An umbrella review of reviews on challenges to meaningful adolescent involvement in health research

**DOI:** 10.1111/hex.13980

**Published:** 2024-01-27

**Authors:** Azza Warraitch, Maria Lee, Delali Bruce, Paul Curran, Qusai Khraisha, Ciara Wacker, Joshua Hernon, Kristin Hadfield

**Affiliations:** ^1^ Trinity Centre for Global Health, Trinity College Dublin Dublin Ireland; ^2^ School of Psychology, Trinity College Dublin Dublin Ireland; ^3^ School of Engineering Stanford University Stanford California USA

**Keywords:** adolescent engagement, adolescent involvement, participatory health research, public and patient involvement

## Abstract

**Background:**

Less than 1% of studies on child and adolescent health report the involvement of adolescents in health research. This is attributed to barriers experienced by researchers and adolescents in the engagement process. To address this under‐involvement of adolescents, we first need a better understanding of the factors that hinder adolescent involvement in health research.

**Objective:**

We conducted an umbrella review of reviews to consolidate the review‐level evidence on the barriers to meaningful involvement of adolescents in health research.

**Methods:**

We preregistered this umbrella review of reviews with PROSPERO (CRD42021287467). We searched 11 databases; Google Scholar; and PROSPERO; supplemented by a hand search of the reference lists of eligible reviews, relevant journals, websites of 472 organisations, and input from experts. This resulted in the inclusion of 99 review articles exploring adolescent involvement in studies on adolescent physical or mental health, which were narratively synthesised. Adolescent coresearchers were engaged at all stages of the review.

**Results:**

We found that adolescent involvement in health research is impeded by several challenges experienced by researchers and adolescents. Some challenges experienced by researchers were organisational issues which included limited resources, gatekeeping and paying adolescents. Some barriers were related to a lack of preparedness among researchers and included a lack of awareness of adolescent involvement, the need for training and guidance, and negative attitudes towards participatory research. There were also barriers around how adolescents can be involved, such as researchers finding it challenging to adapt to new methods, issues with recruitment and retention of adolescents, inclusiveness and accessibility. There were also challenges specific to adolescents, such as adolescents' skills and expertise, training, motivations and study goals. Finally, barriers related to the ethical involvement of adolescents included issues with power dynamics, confidentiality, safety and protection of adolescents. Some of the barriers reported by adolescents included tokenistic involvement, inaccessibility of adolescent involvement, and their competing demands.

**Conclusion:**

Researchers may find this review useful in understanding and planning for potential challenges of involving adolescents in research. Despite many identified barriers to adolescent engagement, few mitigation strategies were identified to address these barriers. There is a clear need to establish best practices for meaningful adolescent involvement in health research.

**Public and Patient Involvement in the Review:**

Adolescents were involved at multiple stages of this umbrella review of reviews. They reviewed the protocol, screened 25% of the articles at title and abstract screening stage, screened 10% of full‐text articles, and worked on data analysis. They also helped plan and conduct a participatory workshop with an adolescent advisory group to discuss the challenges experienced by adolescents in health research.

## 1 INTRODUCTION

The UN Convention on the Rights of the Child (UNCRC) in 1989 highlighted the importance of adolescents' right to be involved in all decisions that affect them,^1^ including their involvement in research on their health. Adolescent involvement in research is defined as ‘research that is done “with” or “by” adolescents, not “to”, “about” or “for” them’.^2^, ^3^ Engagement of adolescents in research is critical for three primary reasons. *First*, Article 12 of UNCRC stated that children and adolescents have a right to be involved in all decisions that affect them.^1^ By extension of this article, it is adolescents' fundamental right to be involved in research on their health. The increasing recognition of adolescents' right to shape health‐related decision‐making has led to an unprecedented demand for their representation in these processes. Many health and funding organisations are strongly advocating for the meaningful involvement of adolescents in health research, as part of their efforts to achieve the United Nations 2030 Agenda for Sustainable Development.^4^, ^5^, ^6^ *Second*, participation in research has positive impacts on adolescents, the research and the researchers.^7^ Adolescent involvement in research improves the quality of the research by making it more relevant to their needs and preferences, expands their knowledge and skills and builds the capacity of researchers to involve adolescents in their work, as well as improving their attitudes towards such involvement.^3^ *Third*, available health services and interventions developed by adults are not currently meeting the needs of adolescents and are often described as unengaging by adolescents.^7^, ^8^

However, despite this increased demand and recognition for participatory research with adolescents, they remain under‐involved in research. This is reflected in the paucity of published literature describing the involvement of adolescents in the design and implementation of health research studies.^9^ A scoping review on adolescent involvement in health research concluded that less than 1% of studies on child and adolescent health reported the involvement of adolescents as advisors.^10^ While the significance of participatory research with adolescents is increasingly being recognised, it is still considered the ‘exception rather than the rule’ as adolescents' perspectives are rarely sought and reported in published research.^3^, ^11^ A review exploring overall public and patient involvement found that most studies reported seeking input from adult carers and stakeholders instead of engaging adolescents in the design of research projects.^12^

This under‐involvement of adolescents in health research can be attributed to the barriers experienced by researchers and adolescents in the adolescent engagement process.^3^, ^10^ Multiple studies conducted with researchers highlighted substantial barriers to engagement of adolescents in health research which could explain the higher engagement of adults in health research as compared to adolescents.^13^, ^14^, ^15^ Lohmeyer indicated that whilst adolescent participatory research should involve adults, it must be led by adolescents. However, in practice this is hindered by ‘social, historical, procedural and institutional barriers’.^16^ These barriers can be experienced at the researcher, organisational and funding levels.^10^ Nowland et al.^17^ argued that without understanding and addressing these barriers to participatory research with adolescents, research is another form of ‘symbolic violence’. This means that adolescents will remain in a passive position in the research process and will be manipulated and exploited with no real power over the issues they want to explore through research.^13^, ^14^, ^15^, ^17^, ^18^, ^19^

To address this under‐involvement of adolescents, we need a better understanding of the factors hindering their engagement and contribution to health research. To achieve this, we need to first consolidate the challenges experienced by researchers and adolescents in the engagement process and then explore the mitigation strategies available to prevent or address these barriers. This will enable the researchers to better prepare for engagement of adolescents in health research, which may lead to an increase in meaningful adolescent involvement in health research. A scoping search of the literature indicated that multiple reviews have been published on adolescent engagement in different types of health research. However, these reviews focused on different adolescent engagement strategies and had different eligibility criteria. Thus, we conducted an umbrella review to synthesise the evidence on barriers to adolescent engagement in health research from these varied reviews, and to obtain a consolidated list of the factors that may limit adolescent involvement in health research.

## 2 METHODS

We have followed the Cochrane guidelines for overviews of reviews.^20^ We preregistered this umbrella review with PROSPERO (#CRD42021287467). Supporting Information files can be accessed at https://osf.io/cx7y9/. We follow the Preferred Reporting Items for Overviews of Reviews statement in reporting of this paper.^21^ The detailed methodology of this review has been described in the protocol paper of this umbrella review.^22^

### 2.1 Eligibility criteria

#### 2.1.1 Study design

We included review articles of all types: systematic, narrative, critical, mixed‐methods, rapid, qualitative, scoping, integrated, realist and state‐of‐the‐art reviews, as well as meta‐analyses, evidence maps and overviews.

#### 2.1.2 Population

We included all reviews that aimed to explore the involvement of adolescents in health research. Adolescents were defined as those between 10 and 24 years old as per the definition of adolescence by Sawyer et al.^23^ We adopted this age range for this review, instead of the other commonly used age range of 10–19, as it aligns with the recent changes in adolescent growth and the shift in their roles and responsibilities.^23^ Reviews that included studies with a sample in the age range of 10–24 years were eligible for inclusion. If the age range in a given review was broader than this—for instance, if a review included studies with young people aged 5–25 years—we only included the subset of studies from that review where participants were between 10 and 24 years.

#### 2.1.3 Intervention

We included reviews of all types that examined adolescent involvement in research on the promotion of physical and mental health among adolescents or on the treatment of adolescents' health difficulties. We defined adolescent involvement as ‘research that is done “with” or “by” “adolescents”, “not” “to”, “about”or “for” them’.^2^, ^3^

#### 2.1.4 Outcomes

As the current paper was part of a broader umbrella review investigating various aspects of adolescent involvement in health research, reviews investigating at least one of the following outcomes were eligible for inclusion: (i) strategies or methods that have been employed to involve adolescents in health research; (ii) best practices in engaging adolescents in health research; (iii) barriers to the meaningful involvement of adolescents, as reported by researchers or adolescents, and strategies for mitigating these challenges; and (iv) gaps in the literature on adolescent involvement in health research.

#### 2.1.5 Other criteria

Reviews published in English were considered for inclusion. If the research aims of a given review were broader than those of the umbrella review, we included only the subset of primary studies that met the umbrella review's eligibility criteria, per the Cochrane guidelines.

#### 2.1.6 Exclusion criteria

Review protocols were excluded. If data extraction tables were unavailable for a systematic or scoping review, this review was excluded from our analyses. We excluded primary studies where the target population was aged below 10 and above 24 years at the data extraction stage.

### 2.2 Information sources

We searched the following sources for peer‐reviewed literature: Cochrane Database of Systematic Reviews, MEDLINE, Scopus, Embase, PsycINFO, PsycArticles, Cumulative Index to Nursing and Allied Health Literature, Epistemonikos, Health Systems Evidence, Google Scholar, 10 leading paediatrics, perinatology and child health journals, the reference lists of eligible reviews, and the connected papers website. For grey literature, we searched the Web of Science, ProQuest, websites of 472 relevant health organisations, PROSPERO, as well as contacted experts in adolescent involvement in health research. We ran the search in English and restricted the results to English language. No restrictions were applied on the date of publication. Search strategies and dates are available at: https://osf.io/cx7y9/.

### 2.3 Selection process

Three researchers undertook title and abstract screening and full‐text screening of the studies in Covidence. A. W. screened all studies at both stages. At the title and abstract screening stage, Q. K. and D. B. screened 25% of the identified titles and abstracts, as well as 10% of the full texts. Any disagreements were resolved through discussion among these three researchers and, if required, through consultation with a fourth researcher (K. H.).

### 2.4 Data collection process and data items

We extracted data on the characteristics of the review articles and primary studies, barriers to adolescent involvement and mitigation strategies, and limitations of reviews. D. B. and P. C. each extracted data from 10% of included reviews, and A. W. extracted data from all reviews. We used Covidence and Excel for data extraction.

Overlap in the primary studies was identified using a citation matrix,^20^, ^24^ where the primary studies were cross‐linked with the reviews in which they were included. The identified overlap was quantified by calculating the corrected covered area^24^ that indicates the degree of overlap. The overall degree of overlap between the primary studies was 0.0026. Where a study was included in more than one review, when extracting data we included the review that provided more details for that study.

### 2.5 Risk of bias assessment

A MeaSurement Tool to Assess Systematic Reviews‐2 (AMSTAR 2)^25^ was used to conduct risk of bias assessment for the included systematic reviews. A. W. assessed the risk of bias for all systematic reviews, while D. B. and P. C. conducted the risk of bias assessment for 10% of eligible reviews. Risk of bias assessment was not conducted for the primary studies included in most reviews and was not reassessed.

### 2.6 Narrative synthesis

Due to the qualitative nature of the data, we analysed the extracted data using narrative synthesis.^26^ A. W. and M. L. analysed the data by coding each sentence using deductive coding. Once the sentences were individually coded, the next step was to merge these codes into broader subthemes. This consolidation allowed for the organisation of related codes, creating a more manageable and structured representation of the data. The purpose of this step was to identify overarching patterns or trends within the coded information. In the subsequent stage, we merged them into more comprehensive themes. This process aimed to distil the coded content further, identifying higher‐level concepts that encompassed multiple subthemes. By doing so, the analysis moved from specific details to more broader and inclusive themes.

### 2.7 Involvement of adolescents in the review

Adolescents aged 18–24 years were involved at multiple stages of this review as advisors and co‐researchers. Of these, three co‐researchers and five advisors were female while two co‐researchers and seven advisors were male. The co‐researchers reviewed the protocol (D. B., M. L., P. C.), screened 25% of the articles at title and abstract screening stage (D. B.), screened 10% of full‐text articles (D. B.), worked on data extraction (D. B., M. L., P. C.), worked on data analysis (M. L.), and helped plan and conduct a participatory workshop with an adolescent advisory group to discuss the challenges experienced by adolescents in health research (C. W., J. H.). The co‐researchers involved in the screening and data extraction were trained using the Cochrane guidelines for overviews of reviews.^20^ The coresearcher involved in the data analysis was trained using the narrative synthesis guidance manual by Popay et al.^26^

We conducted a participatory workshop with 12 adolescent advisors from Kenya and Ireland to discuss the challenges reported by the adolescents in literature. The Health Policy and Management research ethics committee at Trinity College Dublin exempted the workshop from ethics approval as they categorised it as public and patient involvement of adolescents. We aimed to explore adolescents' interpretation of these findings in light of their own experiences and to brainstorm mitigation strategies to these and other challenges experienced by adolescents during their participation in health research studies (Table 2). The workshop had a hybrid structure, with three adolescent advisors from Ireland attending in‐person, alongside the virtual participation of nine adolescent advisors from Kenya. We used icebreakers to foster a positive atmosphere. We then conducted structured brainstorming sessions in smaller groups to explore barriers faced by adolescents when contributing to a research project and to deliberate on strategies to address them. To systematically record the discussions, the suggestions of adolescent advisors were documented on sticky notes and charts by two adolescent coresearchers, C. W. and J. H. Subsequent data analysis was conducted using narrative synthesis, wherein each suggestion was given a unique code. These codes were then aggregated under broader subthemes, facilitating a methodical exploration of insights from the workshop. This process enabled the identification of recurrent themes and meaningful patterns, enhancing our understanding of the perspectives and recommendations provided by adolescent advisors.

## 3 RESULTS

**Figure 1:**
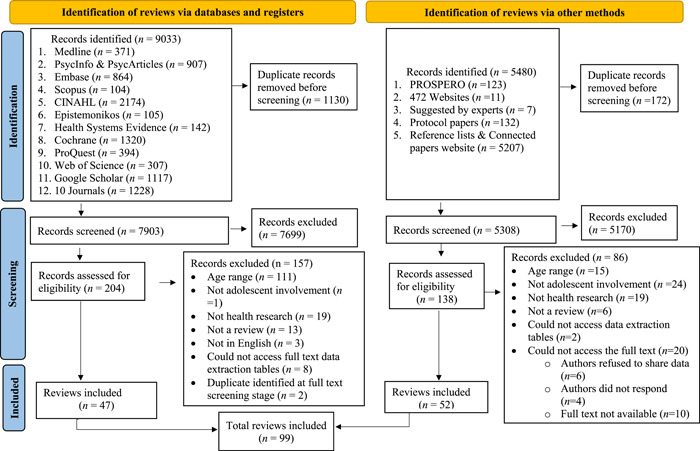
Preferred Reporting Items for Overviews of Reviews flow chart.

Through our searches, we identified 14,513 potentially relevant studies, of which we ultimately included 99 review articles (Figure 1). Most were systematic (41%) and scoping reviews (19%). A total of 92% of the reviews were published since 2010, with 69% published since 2017. On average, the included reviews searched four databases for peer‐reviewed studies and onr database for grey literature. The full database matrix can be accessed at https://osf.io/cx7y9/. Sixty‐five percent of the studies included in the reviews were conducted in high‐income countries while 14% of the studies were from low‐ and middle‐income countries.

Risk of bias was assessed for systematic reviews (*n* = 41) included in this umbrella review using AMSTAR 2. Most reviews did not meet the criteria for a rigorously conducted review. For example, more than 30% of the reviews did not provide details on their selection of the study designs for inclusion in the review, did not extract data in duplicate, did not use a risk of bias assessment method for primary studies, or did not report conflicts of interest. Similarly, more than 70% of the included systematic reviews did not describe whether the methods had been established before the conduct of review, provide a list of excluded studies, or report the funding sources for included studies.

### 3.1 Barriers to meaningful involvement of adolescents in health research

Multiple challenges to adolescent involvement in health research were identified for researchers (Table 1).

**Table 1:** Barriers to adolescent involvement in health research reported by researchers.

Categories of challenges	List of challenges
Organisational challenges	Limited resources Gatekeeping in adolescent involvement Paying adolescents in research
Researchers' preparedness	Lack of awareness and need for training and guidance Inadequate planning of adolescent involvement Negative attitudes towards participatory research Challenges of ensuring meaningful engagement Negative impact on researchers' careers
Methodology of adolescent involvement	Adapting to new methods of working Recruitment of adolescents Retention of adolescents Inclusiveness and accessibility
Adolescent‐specific issues	Adolescents having limited research skills and expertise Lack of consensus on the need for training adolescents Differences in adolescents' motivations and study goals Adolescents' input and involvement Building relationships with adolescents Time commitment of adolescents
Ethical involvement	Informed consent Confidentiality Safety and protection of adolescents Ethical approval Power dynamics

### 3.2 Challenges reported by the researchers

#### 3.2.1 Organisational challenges

1. *Resources*. Adolescent involvement can be expensive due to the logistics of engaging adolescents in codesign activities,^27^, ^28^ the need for specific equipment such as digital cameras in photovoice methods,^29^ paying them honorariums or a salary, hiring more than one co‐researcher for interviews to be conducted in pairs and so forth,^8^, ^28^, ^30^, ^31^ and hiring dedicated staff to support adolescent engagement.^32^ Lack of appropriate funding can aggravate all the other challenges of adolescent involvement and be a key structural barrier to meaningful involvement of adolescents.^3^

A related challenge is the power dynamics created due to the funding‐related pressures of funders,^33^ universities and research organisations.^28^ For instance, funding institutes often require detailed plans from researchers at the proposal development stage, which leaves little room for adolescents to provide input in the project design at later stages.^28^, ^34^ Similarly, adolescent engagement requires a substantial amount of time^28^ as the research process may be prolonged, and it can be difficult to allocate sufficient time to meaningful adolescent engagement within short funding periods.^35^, ^36^ This can be attributed to the iterative nature of participatory research,^37^, ^38^ detailed planning that is required for the involvement activities, and the need for flexibility.^27^ Researchers may have to work around adolescents' schedules and commitments which may extend the projects' timelines.^30^

Limited time and resources can curtail dialogues among different stakeholders and may contribute to adolescents dropping out from the research.^38^ Time restrictions also make it difficult to involve adolescents who appear less engaged^39^ and to deliver outputs on time.^40^, ^41^

2. *Gatekeeping*. The involvement of gatekeeper organisations such as schools can affect the recruitment and selection of adolescents.^28^ When schools are involved as gatekeepers, headteachers can try to steer adolescents towards certain topics which can be difficult to manage for researchers.^28^ Involving these gatekeepers can be considered antithetical to the idea that adolescents are competent agents.^33^ Furthermore, in meetings with adolescents with disabilities, gatekeepers can interrupt communication and prevent them from speaking up.^35^

3. *Payment*. Payment to adolescents can be an ethical dilemma,^3^ especially in low‐ and middle‐income countries, with marginalised groups,^42^ and in school settings when adolescents may be involved in research as part of their school activities.^33^ Payment can sometimes be seen as contentious,^28^ especially when adolescents are below the legal age of employment.^28^, ^43^ In some situations, compensating adolescents with alternative methods such as gifts has been equated with economic exploitation, especially when research is interfering with adolescents' income‐generating work.^43^

#### 3.2.2 Challenges related to researchers' preparedness

1. *Lack of awareness of adolescent involvement and need for training and guidance*. The involvement of adolescents in research is often hindered by a lack of awareness and knowledge among researchers. Some researchers may be unaware of the benefits of involving them in research or may assume that adolescents do not have the skills to be engaged in research.^3^ Researchers may not be used to seeing adolescents as experts, which results in them not valuing adolescents' views and expertise.^28^, ^44^, ^45^ Adolescents tend to be seen as incompetent, disordered or asocial which is also a reported barrier to their meaningful involvement in participatory research.^28^, ^44^, ^45^ Some researchers highlight that while there might be recognition of the validity of adolescents' voices in high‐income countries, that is not the case in many low‐ and middle‐income countries, especially in Africa.^43^

To address this lack of awareness among researchers, there is a need for comprehensive training and guidance for researchers on involving adolescents in health research. While there are some resources for researchers on adolescent involvement, these are not standardised or comprehensive, making it challenging for researchers to navigate adolescent involvement.^3^, ^27^, ^28^

2. *Inadequate planning of adolescent involvement*. Poor planning of adolescent engagement can result in tokenistic involvement and poor‐quality research.^3^ However, the requirement to submit extensive plans for adolescent engagement to funding panels and ethics committees can also make it challenging to adapt the procedures later to suit adolescents' needs and preferences.^46^

3. *Negative attitudes towards participatory research*. Concerns were reported about the scientific rigour and validity of participatory research. These may be attributed to several factors. For instance, it can be difficult to apply standardised methods in participatory research because the methods are decided by the researchers and the participants together^47^ and may involve the use of creative activities. Adolescents prefer these methods because they are more interested and skilled in these.^48^ However, these methods may not be considered scientific or rigorous.^37^ This may result in negative perceptions about participatory research, with researchers who employ participatory methodologies reporting that their work is often not held in the same esteem as compared to other methodologies. Similarly, some researchers report that while they understand the value of adolescent involvement in research, they find it difficult to justify in grant applications. This may be due to the participatory involvement of adolescents not being taken seriously or not being perceived as a mainstream method, which makes it difficult for researchers to seek funding for involving and training adolescents.^3^

4. *Challenges of ensuring meaningful engagement*. Engaging adolescents in a meaningful way can be challenging.^27^ Researchers must navigate the delicate balance of avoiding tokenism and exploitation of adolescents,^49^ while protecting the authenticity of their voices.^36^ One major concern is ensuring that their contributions are accurately captured and not lost in translation.^27^, ^36^ Ensuring the authenticity and integrity of adolescents' input in research is crucial to avoid misinterpretation or oversimplification of their perspectives, which could lead to inaccurate conclusions or recommendations.

5. *Negative impact on researchers' career*s. The demands of adolescent involvement, resource needs and challenges associated with engaging adolescents may have a negative impact on researchers' careers.^30^ For example, the time invested in ensuring adolescents are properly trained and involved in a meaningful way might result in publication delays for researchers.

#### 3.2.3 Barriers related to the methodology of adolescent involvement

1. *Adapting to new methods of working*. Researchers need to be adaptable in working with adolescents.^30^ This can involve conducting preparation meetings,^3^ changing the format of meetings to incorporate different preferences,^3^, ^50^ orienting adolescents beforehand on the topics and methods being used,^3^ and being flexible to accommodate adolescents' personal situations.^3^, ^27^, ^51^

2. *Recruitment of adolescents*. Some of the factors that can make recruitment of adolescents difficult include having a very specific target sample such as adolescents with mental health conditions or previous history of suicide attempts, having to seek parental consent,^52^ and the identification of an organisation through which to recruit adolescents.^52^, ^53^

It is also challenging to recruit a diverse group of adolescents.^54^ Engaged adolescents can be more outspoken, confident and critical than their nonparticipating peers.^38^ It is crucial to recruit adolescents using a method that does not favour privileged subgroups, further perpetuate inequalities, or result in the overinclusion of a specific subgroup.^28^, ^49^, ^55^ Furthermore, those adolescents who put themselves forward to be part of health research projects may not be the most influential among peers as peer educators and may not be the ones who would benefit the most from participation.^46^, ^56^ They already may have more knowledge and awareness of health issues than their peers.^27^ Finally, self‐selection bias in peer education can also result in a homogenous group of high‐achieving female peer leaders.^57^, ^58^

Recruitment and retention can be especially challenging in high‐risk minority populations.^59^ Marginalised adolescents may be reluctant to lead research or be involved as they might feel intimidated by researchers or unfamiliar environments.^33^ Indigenous adolescents' experiences of colonial exploitation may make them wary of predominantly nonindigenous researchers and participation in research.^60^

3. *Retention of adolescents*. Adolescents tend to drop out of studies due to strenuous processes involving heavy workload and long‐term time commitment,^38^ study delays,^27^, ^39^ or due to decreased interest in the study with the passing of time.^61^ Competing demands and interests of adolescents, the need for payment, and a lack of incentives also contribute to attrition.^57^, ^62^

4. *Inclusiveness and accessibility*. Adjusting the activities and methods to suit the needs of adolescents from different age groups and abilities can be difficult.^33^ However, if the study is noninclusive, adolescents may feel inadequate and less confident instead of feeling competent. This may also lower their self‐esteem and may result in adolescents having a negative perception of the research which can make them less likely to get involved in research in the future. They can also feel excluded if they are expected to have pre‐existing knowledge of research methods and to perform at the same level as established researchers.^30^

#### 3.2.4 Adolescent‐specific challenges

1. *Adolescents having limited skills and expertise*. Adolescent co‐researchers having limited skills (e.g., interviewing skills) were reported as a concern by researchers.^49^ Researchers tend to overestimate or underestimate adolescents' skills and knowledge^27^ and can have unrealistic expectations from adolescents.^33^ This can sometimes lead to researchers investing substantial resources in building capacity in adolescents who are not ultimately engaged or interested in research.^27^ Additionally, depending on the project, certain tasks may be methodologically too advanced, and adolescents may not be interested in working on those tasks, making it impractical for researchers to involve adolescents in all aspects of the research.^27^

2. *Lack of consensus on the need for training adolescents*. On one hand, researchers are cognisant of the importance and need for training adolescents to ensure the quality of research,^3^, ^28^, ^42^ and indicate that it would be immoral to not train adolescents.^28^ On the other hand, adolescents are experts in their own lives, and their perspectives provide valuable insights into their lived experiences. This raises concerns that their training can affect these lay perspectives, making their views too similar to professionals' viewpoints, and may alienate them from other adolescents.^28^

3. *Difference in motivations of adolescents and goals of the study*. Adolescents may have different motivations from adults and this difference can hinder study progress. For example, in a study by Bagnoli et al.,^63^ adolescents requested inclusion of more creative methods, but these methods were considered by researchers to pose challenges with analysis and dissemination. Similarly, the researchers reported some ethical concerns when the participants requested the data to not be anonymised to have their voices heard. This conflicted with the researchers' desire to maintain privacy and confidentiality.^64^

4. *Adolescents' input and involvement*. Researchers may perceive adolescents' feedback to be unrealistic and unfeasible to adopt or it may be in conflict with the institutional policies.^39^ Cultural norms or local political scenarios can also affect adolescents' input (e.g., strict state control and a collectivist cultural structure) as they might deter the adolescents from speaking up.^42^ Additionally, when adolescents are engaged as interviewers of other adolescents, they may overidentify with the interviewees and assume they know too much and can risk losing their stance as an outsider interviewer. They may also lose the insider perspective when they take on the peer interviewer role.^28^

5. *Building relationships with adolescents*. If adolescents do not trust the researchers, they might be reluctant to get involved in research and collaborate with researchers. However, their limited availability due to competing schedules may also contribute to challenges in building trust‐based relationships with the researchers.^37^ Insufficient focus on the quality of relationships with adolescents may be one of the reasons for the lack of sustainability of adolescent involvement in health research.^65^

6. *Time commitments of adolescents*. Adolescents have competing demands that make it difficult for researchers to involve them regularly for extended periods.^3^, ^52^, ^66^, ^67^ They have busy lives due to school, extracurriculars, family responsibilities and social activities, which makes it challenging to schedule meetings,^3^, ^67^ especially during the day.^28^ These demands may limit their ability to participate intensively in research activities,^27^ such as the interpretation of findings.^68^ The time available to spend on a project necessarily may limit adolescents' level of involvement with and contributions to projects.^28^

#### 3.2.5 Ethical involvement of adolescents

1. *Informed consent*. Researchers struggle to balance between the two imperatives in informed consent: (1) respecting adolescents' autonomy by ensuring they can freely decide to participate and (2) respecting parental responsibility for adolescents' wellbeing and safety. Parental consent is well‐intentioned but may be restrictive and can affect adolescents' right to decide about their participation in studies.^43^ It may be inappropriate to seek parental consent in some situations.^8^ This can include research on sensitive topics that require complete privacy from parents to protect participants, such as research on drug abuse.^43^, ^69^ It may be unethical to exclude participants for lack of parental consent in some cases, for example, excluding street children who may not have contact with their parents^43^, ^70^ or when adolescents are emancipated.^43^

When participatory research is conducted at a group level, for example, in schools or communities, group‐level consent takes precedence over individual‐level consent, which can make individuals feel pressured to participate.^42^ Within multicultural settings, consent can be complicated if the researchers and participants do not speak the same language, or share the same culture or understanding of the consent process.^42^

2. *Power dynamics*. Some factors that can contribute to the power dynamics between researchers and the adolescents include; (i) language‐related differences,^42^ (ii) restrictive and limited adolescent involvement,^68^, ^71^ (iii) researchers holding power over further opportunities,^49^ (iv) culture‐ and gender‐related power differences,^36^, ^43^ (v) researchers' perception of adolescents' capacity and competence,^37^ (vi) role of funding organisations in dictating the level of adolescent involvement^33^ and (vii) conducting research in settings where adults are the authority figures, such as schools.^72^

3. *Confidentiality*. It is difficult to ensure the confidentiality and anonymity of adolescents in participatory research.^3^, ^35^, ^42^, ^49^, ^73^ For example, participatory work involving exhibition of photovoice projects can involve identifying content.^42^ When adolescents are engaged as co‐researchers, it may involve them knowing the identity of their peers who are participating in research^37^ and they may be involved in data management.^42^ Finally, adolescents interviewing peers on sensitive topics may require additional confidentiality parameters.^36^, ^42^

4. *Safety and protection of adolescents*. Participation in research may sometimes put adolescents at risk of psychological and emotional harm^3^, ^42^ as some of the research‐related content or activities may be distressing to adolescents.^3^, ^35^, ^42^ This may especially be the case if the adolescent researchers and participants share their own experiences.^33^ For example, adolescents' participation in the analysis of sensitive content may cause them distress due to reliving their own difficulties.^33^ Similarly, asking people to share their lived experiences of a disability or health condition can be distressing.^3^ In peer‐led interventions, peer leaders can feel more guilt after intervention delivery due to being unable to help their peers as much as they could have or becoming more aware of the inequalities among their peers.^74^

5. *Ethical approval*. Researchers face substantive barriers in seeking ethical approval for the codesign of research with adolescents. Codesign research methodology contradicts the existing protocols for the ethics committees that dictate the requirement for defined interventions, delivery methods and outcome measures, given that these tend to be adaptive and emergent in participatory research.^75^

### 3.3 Challenges reported by adolescents

Six main barriers to involvement in health research were reported by the adolescents. These included tokenistic involvement of adolescents, reluctance to get involved in research, inaccessible adolescent involvement practices, lack of confidence in competence and skillset, competing demands and workload, and not getting proper acknowledgement for their contributions.

#### 3.3.1 Tokenistic involvement

Adolescents may feel like they are not being listened to^35^, ^49^ or not taken seriously.^76^ They can perceive their involvement to be tokenistic^35^ and can feel manipulated^49^ if their involvement is not reflected in decision‐making.^31^ This can result in disillusionment about the benefits of involvement.^35^, ^77^ Adolescents can experience consultation fatigue if they are involved, but their views are not taken into account and may feel ‘what's the point’ when invited to participate in further research.^33^, ^78^

#### 3.3.2 Reluctance to get involved

Adolescents may be reluctant to get involved in health research studies.^79^ This could be due to negative perceptions of health research among adolescents.^3^ They may also be disinterested as they might perceive research to be boring, complex or slow.^3^ For instance, adolescents often consider data analysis to be a time‐consuming and mundane task.^3^ Sometimes adolescents' negative assumptions about research are related to a lack of awareness of how research works.^3^ In marginalised communities, adolescents' reluctance to get involved in research may be due to a lack of trust in researchers and decision‐makers.^79^

#### 3.3.3 Inaccessibility

Adolescents who attend research meetings often report different accessibility issues, such as people speaking too fast, the words used being ‘too big’, and meeting agenda not being shared in advance.^28^, ^52^ Meetings may be scheduled at a time that is, inconvenient for adolescents,^61^ such as at lunch or after school^39^ when they are hungry and tired.^28^

Adolescents can feel intimidated around professionals or about working in unfamiliar places.^33^, ^35^ They may find some methods exhausting,^46^, ^80^ activities too dry^46^, ^81^ or the work not age‐friendly.^82^ Lack of proper aids can lead to the exclusion of adolescents with disability.^35^ They also report difficulties in transportation to meeting venues.^8^, ^52^, ^61^

#### 3.3.4 Competence and skillset

Adolescents sometimes require specific skills to be able to contribute to research in a meaningful way.^38^ Adolescents may doubt their competence to get involved^8^, ^28^, ^68^ and whether they can acquire the required skills due to limited time.^39^, ^83^ They may lack confidence^35^, ^65^ in leading intensive research activities^79^ and may feel uneasy about conducting research.^65^ This is especially true in cases where adolescents are involved in projects that are unrelated to their experiences.^67^ Some adolescents hesitate to speak in public^49^ and can have camera shyness.^68^ They may also feel stressed about ‘performing’ in front of other people.^61^

Inadequate training and capacity building of adolescents^3^, ^65^, ^84^, ^85^ was also a reported concern of adolescents. Without adequate training, the resulting research may be of poor quality,^30^ and adolescents may not retain the necessary information required for them to carry out their roles.^61^ Adolescents may also question other adolescents' competence and abilities. In one of the studies, adolescents refused to interview younger people because they questioned their ability to understand the questions.^36^

#### 3.3.5 Competing demands and workload

Participation in research projects can increase adolescents' workload.^61^ This additional workload can be problematic for younger adolescents or those with chronic health issues.^73^ They can also feel pressured due to time constraints of the research.^61^ Participation in research can also affect adolescents' studies and they may feel conflicted about participating in projects if this diverts their time and attention from their schoolwork.^76^

#### 3.3.6 Not getting proper acknowledgement

Adolescents find it difficult to claim their work if they cannot be acknowledged in research by name.^42^

#### 3.3.7 Challenges reported by the members of Adolescent Advisory Board

Less than 10 challenges were reported from the perspective of adolescents in the included reviews. The limited reporting of challenges experienced by adolescents could be due to adolescents not having the right platform to voice their opinions and to share their experiences, as research articles are usually written and published by adult researchers. To expand on the limited evidence from the reviews, we conducted a participatory workshop with an advisory group of adolescents (aged 18–24 years) to discuss and expand on the challenges reported by adolescents identified in the umbrella review. This advisory group was comprised of 12 adolescents. Nine advisors were from Kenya, while three were from Ireland. The advisory group included seven male advisors and five female advisors. This advisory group agreed with most of the challenges that were reported for adolescents in our review and shared several additional barriers (Table 2).

**Table 2:** Results of participatory workshop with the adolescents.

*Level of involvement*: Adolescents often have limited impact on the decision making. Researchers sometimes come in with everything predecided and want adolescents to sign off on the project. Adolescents are usually not involved in data analysis. They are not asked to review resulting outputs, let alone be acknowledged with authorship of those outputs.
*Respect and trust*: Researchers should not make adolescents feel inferior for not knowing as much as them. Mistrust gets in the way of genuine collaborations.
*Different perspectives*: Adolescents sometimes have very different ideas from researchers, and these are not always appreciated or incorporated by researchers. Researchers are afraid adolescents will disagree with them.
Inaccessible language: The use of jargon‐heavy language makes research inaccessible to adolescents. It alienates adolescents, prevents them from feeling welcome or included in the research process and may discourage them from participating in research altogether. It can also lead to miscommunication.
*Environment*: Researchers should make efforts to create a welcoming environment. It is hard for adolescents to get involved when they feel intimidated, like they do not belong there, or as if they do not know what they are doing.
*Competing demands and workload*: Researchers need to manage adolescents' time effectively. Adolescents have busy schedules and need to balance research work with their other obligations. Researchers must consider the burden of participation for adolescents.
*Timelines*: Tedious and lengthy work can result in loss of motivation. Postponing activities and lag in timelines have a negative effect on adolescents' motivation to contribute to research.
*Compensation*: Compensation is usually provided in the form of vouchers. Vouchers are not appropriate for every context. Adolescents cannot afford to wait to be reimbursed so they should be compensated in advance. If the project is multicountry, the hourly rate of compensation should not be different for different contexts. For example, if an adolescent in a high‐income country is being paid a certain amount per hour, those in a low‐ or middle‐income country should be paid the same. If researchers are unable to compensate adolescents, they should be honest and upfront with them about it. They should discuss alternatives to monetary compensation with adolescents in advance.
*Benefits to adolescents*: Researchers need to carefully think about how adolescent involvement in research benefits adolescents. A 1‐hour workshop may not necessarily increase adolescents' knowledge or build their skills. Often adolescents do not get any direct benefits from their engagement in research.
*Adolescent‐led research*: Adolescents are not supported as independent researchers. Adolescents should be supported as researchers by teaching them specific skills like grant writing.
*Cultural differences*: There are cultural differences in the dynamics between adults and adolescents. These differences should be taken into account when planning adolescent involvement.
*Feedback to adolescents*: Sometimes adolescents do not hear back from the researchers after they are finished with data collection. They only get to learn about the final outcome of the project they contributed to when it is presented on a big forum or published.
*Acknowledgement*: Being mentioned in the acknowledgements section of a paper is not sufficient. Adolescents should be acknowledged as coauthors. Being a coauthor can be important to them and help them as future researchers.

## 4 DISCUSSION

This umbrella review consolidates the barriers to meaningful adolescent engagement from 99 reviews on adolescent involvement in health research. Through this review, we have highlighted the multifaceted challenges faced by researchers and adolescents, the limited reporting of mitigation strategies available, and the need for the establishment of best practices in adolescent engagement.

The challenges reported by the researchers ranged from issues related to organisational context, researchers' preparedness, methodological issues in engaging adolescents, difficulties in working with adolescents and involving them ethically. Although the challenges and mitigation strategies vary depending on factors such as study aims, target population, settings and methodology of adolescent involvement, there are recurring themes that were consistently reported. Some of the most consistently reported challenges include limited resources, gatekeeping, ensuring diversity and inclusion, designing appropriate methodologies for involvement, providing adequate training and support to adolescents, and addressing ethical concerns.^38^, ^86^ It is noteworthy that many of these challenges are not exclusive to one discipline or methodology. For instance, both mental health^87^ and overall health studies^10^ report similar issues, as do different methodologies like Participatory Action Research^37^ and Youth Advisory Groups.^10^ The same holds true across diverse adolescent populations, including neurodivergent adolescents.^88^ This highlights the need for cross‐disciplinary action and efforts among researchers and organisations to address these barriers by engaging in initiatives like establishing common platforms for learning and organising joint training programmes for researchers from different disciplines.

Most of the reported challenges are interlinked; for example, researchers' concerns about engaging adolescents in a meaningful way are linked to the need for training and guidance on how to achieve this. Similarly, researchers' attitudes and concerns about the rigour of the participatory methods may be associated with their perception of adolescents' abilities. Just as these challenges are interlinked, the mitigation strategies may also address multiple barriers simultaneously. For example, addressing the constraint of limited time and resources can alleviate some of researchers' concerns. This can be achieved by granting researchers more time to customise the methodology of adolescent participation based on adolescents' preferences, thereby ensuring their voices are effectively incorporated.^86^

The challenges experienced are related to organisations, researchers and adolescents and require efforts from multiple stakeholders to address. The key to addressing most of these challenges lies within collaboration between adolescent health organisations, funding bodies that fund adolescent health research, researchers, and adolescents. For example, organisations and funders should offer training opportunities and platforms that facilitate researchers coming together to share their experiences in adolescent involvement.^14^ This approach aims to tackle the lack of awareness on how to involve adolescents meaningfully and the need for guidance among researchers in this regard.

Similarly, some researchers report that participatory research is not held in the same esteem as other methods by their colleagues.^15^ Colleagues believing in the value of adolescent participation, conversely, is a facilitator to adolescent engagement in their research.^15^ To address this barrier, embedding adolescent involvement in the organisational culture and structures may be key. Organisations can do this by adding mandatory reporting on adolescent engagement, providing incentives for meaningful adolescent engagement, incorporating plans for adolescent involvement in funding applications, and requiring evaluation of adolescent engagement in the project to ensure accountability.^14^ Organisations also need to provide researchers with adequate resources and time to (1) invest in capacity building of their research teams, (2) plan for meaningful and long‐term involvement of adolescents that can be sustained beyond the project life, (3) compensate adolescents for their time, (4) form partnerships with gatekeepers that will enable them to access a diverse group of adolescents,^3^, ^89^ and (5) provide researchers with a supportive environment that encourages participatory research with adolescents.^14^ These supports are critical because limited resources and time are the most frequently reported barriers to adolescent involvement.^15^, ^87^ Moreover, when organisations adopt practices to embed adolescent involvement in their culture, it ensures that the extra time needed for adolescent involvement does not negatively affect the outputs that contribute to researchers' career growth such as timely grant submissions and publications.^14^

A key recommendation that has been highlighted across multiple reviews is the need for more guiding resources and training. For instance, a survey conducted with researchers in the United Kingdom revealed 60%–74% of researchers reporting a need for more guidance, training, and support on involving adolescents in research.^86^ Similarly, in an international consultation with stakeholders, two‐third of the participants revealed the need for more training resources and guidelines on adolescent involvement in health research.^89^ Despite researchers expressing an interest and motivation to engage adolescents, their limited know‐how of adolescent involvement limits their ability to engage them in a meaningful way.^15^, ^90^ Training and resources may help researchers overcome the many practical barriers to adolescent involvement identified in this review, such as the challenges of engaging adolescents in a meaningful and ethical way, managing recruitment and retention, and adopting adolescent friendly and flexible practices. This need for training and more guidance has been reported by researchers across a range of career levels.^15^

Understanding and addressing these challenges is the first step towards ensuring meaningful involvement of adolescents. Very few mitigation strategies or best practices have been reported in the literature to address the challenges to adolescent involvement. A recent consultation with stakeholders similarly also reported the lack of best practices in adolescent involvement as a key challenge.^89^ Mitigation of the factors hindering adolescent involvement necessitates establishing the best practices of adolescent involvement. To achieve this goal, we need to consult researchers, funders, administrative personnel, and adolescents about the optimal methods and strategies for meaningful adolescent involvement. These stakeholder consultations can be crucial in operationalising the mitigation strategies to address these challenges and establishing best practices of adolescent involvement.

The landscape of engaging adolescents in health research is marked by a complex interplay of barriers and facilitators. Some factors are considered facilitators to the meaningful involvement of adolescents but there are barriers to achieving these enabling factors. Some examples of these facilitators include training adolescents,^91^, ^92^ involving adolescents from diverse backgrounds,^93^, ^94^ building positive relationships with them,^67^, ^95^ and establishing inclusive environments.^96^ Some reported facilitators to adolescent involvement could also be used to address some of the barriers to meaningful adolescent involvement. For instance, having compassionate staff and fostering dialogic knowledge exchange can facilitate relationship‐building and mitigate power imbalances between researchers and adolescents,^61^, ^67^, ^97^, ^98^, ^99^ as seen in public and patient involvement with adults.^61^, ^93^ Addressing the power dynamics in turn is a facilitator to meaningful involvement of adolescents in health research as it enables adolescents to be involved at all stages of the research process, starting their engagement early on.^44^

Barriers to meaningful involvement of adolescents in health research align closely with those identified in overall public and patient engagement. Ocloo et al.^100^ conducted a review of reviews on overall public and patient involvement and noted similar challenges across individual, team, and organisational levels to those we have noted here. These included negative professional attitudes, tokenistic engagement, power imbalances, limited diversity among contributors, inadequate training, compensation issues, and constraints related to resources and time. Notably, our review mirrors work on public and patient involvement generally in that both identify a scarcity of effective mitigation strategies and facilitators. A collaborative approach incorporating all stakeholders to reshape organisational cultures, policies and practices to foster genuine collaboration, inclusivity and effective mechanisms for both adolescent engagement—and, more generally, public and patient involvement—in health research is needed.

It is important to note that most studies explored the barriers to involvement of adolescents that are experienced by researchers which explains why we only found six barriers reported from adolescents' perspective. Our umbrella review captures what is in reviews, and the lack of adolescent barriers reported in these reviews is striking. The adolescents in our participatory workshop highlighted additional barriers related to adolescent‐led research, emphasised the need for researchers to support adolescents in becoming independent researchers, and discussed the importance of seeking adolescents' input on key aspects of the project. However, the findings from the participatory workshop represent the perspectives of a small number of geographically limited adolescents. This highlights the need for future research to engage adolescents in the evaluation of adolescent involvement to explore how to improve the involvement procedures for adolescents.

The findings of this review can be used by researchers to plan for the potential challenges that can be experienced and budget for the additional actions needed to address those challenges. This can be achieved through development of a risk plan for adolescent involvement in research projects by listing all the potential barriers that could hinder the involvement of adolescents in research and working with the research team, organisational administrators, and the adolescent co‐researchers to develop the plan for addressing those barriers in light of the best practices of adolescent involvement.

## 5 LIMITATIONS

The findings of this umbrella review should be interpreted in light of several limitations. First, we only included review articles published in the English language. Second, while we aimed to ensure inclusion of all reviews focusing on different types of adolescent involvement by using a comprehensive search strategy incorporating a wide range of relevant keywords and searching multiple sources of peer reviewed and grey literature, there is a possibility that we may have missed some relevant reviews due to the wide range of terminologies used to describe adolescent involvement. Third, we applied eligibility criteria at the review and primary study levels and excluded primary studies where the age range of participants was other than 10–24 or where the study focus was not health research. However, some of the studies did not report the age range of participants and in some cases, we could not access the full text of primary studies to determine the age range, so some reviews with participants outside of the range of 10–24 years old may have been included.

## 6 CONCLUSION

This paper consolidates the barriers to meaningful adolescent engagement in health research, bringing together evidence from 99 reviews. The challenges range from individual and organisational barriers experienced by researchers to ethical and practical issues encountered in involving adolescents. Additionally, adolescents reported tokenistic involvement, negative perception of research and inaccessibility as significant barriers to their participation. Limited mitigation strategies have been reported for addressing these barriers, which highlights the need to establish best practices for adolescent involvement in health research. Future research needs to ensure adolescents' perspectives are incorporated in the evaluation of adolescent involvement to assess what worked or did not work for them. The findings of this umbrella review can help researchers plan for potential challenges associated with adolescent involvement in their research proposals and add buffers to obviate barriers or mitigate the effects of these challenges. The consolidated evidence from this review will be used to inform the development of a comprehensive set of guidelines on adolescent involvement in health research. Given the lack of best practices of adolescent involvement in health research, these guidelines will be a valuable resource for researchers who want to involve adolescents meaningfully and ethically in future health research projects.

## AUTHOR CONTRIBUTIONS

**Azza Warraitch**: Conceptualisation; investigation; funding acquisition; writing—original draft; methodology; validation; writing—review and editing; visualisation; formal analysis; project administration; data curation; resources. **Maria Lee**: Writing—original draft; writing—review and editing; methodology; formal analysis; data curation; investigation. **Delali Bruce**: Conceptualisation; writing—review and editing; methodology; data curation; investigation. **Paul Curran**: Methodology; investigation; writing—review and editing; data curation. **Qusai Khraisha**: Investigation; writing—review and editing; visualisation; methodology; data curation. **Ciara Wacker**: Investigation; writing—review and editing; methodology; formal analysis; data curation. **Joshua Hernon**: Data curation; formal analysis; methodology; writing—review and editing; investigation. **Kristin Hadfield**: Conceptualisation; investigation; funding acquisition; writing—review and editing; methodology; project administration; supervision; resources.

## CONFLICT OF INTEREST STATEMENT

The authors declare no conflicts of interest.

## Supporting information

Supporting information.

## Data Availability

The data that support the findings of this study are openly available in the Open Science Framework at https://tinyurl.com/UReview, reference number doi:10.17605/OSF.IO/CX7Y9.
