# Supplementary material for: An umbrella review of reviews on challenges to meaningful adolescent involvement in health research
Source: Health Expect. 2024 Jan 27;27(1):e13980. doi: 10.1111/hex.13980 (PMC10821743; doi:10.1111/hex.13980)
Supplement: Supplementary file 1 — Supporting information. [file HEX-27-e13980-s001.zip › Results/Health research areas.docx]

**Health research areas**

| **Research areas** | **n** |
| --- | --- |
| Health research (or multiple areas of health research) | 31 |
| Overall research | 13 |
| Mental health | 13 |
| Sexual and Reproductive Health (SRH) | 9 |
| Oral Health | 5 |
| HIV/AIDS/STIs | 3 |
| Obesity | 3 |
| Physical activity | 3 |
| Substance abuse | 3 |
| Asthma | 2 |
| Chronic health conditions | 2 |
| Adverse childhood experiences | 1 |
| Cancer | 1 |
| Climate change | 1 |
| Decision-making (Health Research) | 1 |
| Diabetes | 1 |
| Family lives | 1 |
| Healthy Neighborhoods and community planning | 1 |
| Humanitarian program(including health research) | 1 |
| Nutrition | 1 |
| Occupation-related health research | 1 |
| Quality of life | 1 |
| Research with vulnerable or marginalized children | 1 |
