# Supplementary material for: An umbrella review of reviews on challenges to meaningful adolescent involvement in health research
Source: Health Expect. 2024 Jan 27;27(1):e13980. doi: 10.1111/hex.13980 (PMC10821743; doi:10.1111/hex.13980)
Supplement: Supplementary file 1 — Supporting information. [file HEX-27-e13980-s001.zip › Results/Summary of primary studies.docx]

**Summary of primary studies**

| **Total studies** | 2926 | **%** |
| --- | --- | --- |
| Included | 2158 | 74% |
| Excluded | 768 | 26% |
| **Reasons for exclusion** | **#** | **%** |
| Age | 663 | 86% |
| Not health research | 104 | 14% |
| Duplicate | 1 | 0% |

**Geographical distribution of included primary studies**

| **Settings** | **f** |  | **%** |
| --- | --- | --- | --- |
| HICs | 1413 | 65 |  |
| LMICs | 294 | 14 |  |
|  |  |  |  |
| HIC & UMIC | 8 |  |  |
| HIC & UMIC & LMIC | 2 |  |  |
| HIC & LIC | 2 |  |  |
| HIC & LMIC | 1 |  |  |
| LIC | 47 |  |  |
| LMIC | 141 |  |  |
| UMIC | 97 |  |  |
| LMIC & LIC | 2 |  |  |
| UMIC & LMIC | 2 |  |  |
| UMIC & LMIC & LIC | 5 |  |  |
| NR or N/A | 438 |  |  |
