# Supplementary material for: An umbrella review of reviews on challenges to meaningful adolescent involvement in health research
Source: Health Expect. 2024 Jan 27;27(1):e13980. doi: 10.1111/hex.13980 (PMC10821743; doi:10.1111/hex.13980)
Supplement: Supplementary file 1 — Supporting information. [file HEX-27-e13980-s001.zip › Results/Summary of youth involvement methods used in reviews.docx]

**Summary of youth involvement methods used in reviews**

| **Youth involvement terminologies used in reviews** | **f** |
| --- | --- |
| Peer education (Peer based interventions, Peer buddying, Peer counselling and peer education, peer proximity, peer-delivered, Peer-to-Peer Health Promotion, peer tutoring), Peer research (Peer involvement, Peer participation) | 21 |
| Participatory research (Participatory approaches and methods, Participatory arts and visual methods) | 12 |
| Peer led, peer leadership | 10 |
| Involvement | 9 |
| Participation | 8 |
| Youth engagement | 8 |
| Patient and Public Involvement (PPI) , Patient and public involvement and engagement (PPIE), Public and patient involvement (PPI) | 6 |
| Peer-mediated intervention (PMI) | 6 |
| Youth Participatory Action Research (YPAR) | 6 |
| Research with children and research by children | 6 |
| Active involvement (e.g. active involvement, active participants, activism, children as active participants) | 4 |
| Co-design | 4 |
| Co-production | 4 |
| Participatory Action Research (PAR) | 4 |
| Community-Based Participatory Action Research (CBPAR) | 3 |
| Photovoice | 3 |
| Community-Based Participatory Research (CBPR) | 2 |
| Co-research and co-researchers | 2 |
| Peer-assisted learning (PAL) | 2 |
| Peer mentoring | 2 |
| Youth advisory groups | 2 |
| Youth organizing | 2 |
| Asset-based community development (ABCD) | 1 |
| Community engagement | 1 |
| Consultation | 1 |
| Counselling | 1 |
| Eliciting children's and young people's views | 1 |
| Human-centered design (HCD) | 1 |
| Just-in-time adaptation | 1 |
| Lived experience | 1 |
| Meaningful participation | 1 |
| Outreach | 1 |
| Peer support | 1 |
| Personalization | 1 |
| Student voice | 1 |
| Youth-led planning | 1 |
