# Supplementary material for: An umbrella review of reviews on challenges to meaningful adolescent involvement in health research
Source: Health Expect. 2024 Jan 27;27(1):e13980. doi: 10.1111/hex.13980 (PMC10821743; doi:10.1111/hex.13980)
Supplement: Supplementary file 1 — Supporting information. [file HEX-27-e13980-s001.zip › Results/Summary of age range of participants in included reviews.docx]

**Summary of age range of participants in included reviews**

| **Age range in years** | **f** |
| --- | --- |
| 10 to 24 | 10 |
| <18 | 6 |
| 10 to 19 | 5 |
| <18 | 4 |
| 5 to 18 | 4 |
| <16 | 3 |
| <25 | 3 |
| <25 | 3 |
| 12 to 18 | 3 |
| <12 | 2 |
| 5 to 25 | 2 |
| >12 | 1 |
| <20 | 1 |
| <24 | 1 |
| 0 to 24 | 1 |
| 10 to 29/30 | 1 |
| 10 to 32 | 1 |
| 11 to 16 | 1 |
| 11 to 20 | 1 |
| 11 to 21 | 1 |
| 12 to 19 | 1 |
| 12 to 21 | 1 |
| 12 to 25 | 1 |
| 13 to 18 | 1 |
| 13 to 20 | 1 |
| 13 to 21 | 1 |
| 13 to 29 | 1 |
| 14 to 26 | 1 |
| 15 to 24 | 1 |
| 15 to 25 | 1 |
| 15 to 29 | 1 |
| 16 to 24 | 1 |
| 3 to 20 | 1 |
| 4 to 18 | 1 |
| 5 to 17 | 1 |
| 5 to 19 | 1 |
| 8 to 26 | 1 |
| Not reported | 28 |
