# Supplementary material for: An umbrella review of reviews on challenges to meaningful adolescent involvement in health research
Source: Health Expect. 2024 Jan 27;27(1):e13980. doi: 10.1111/hex.13980 (PMC10821743; doi:10.1111/hex.13980)
Supplement: Supplementary file 1 — Supporting information. [file HEX-27-e13980-s001.zip › Results/Summary of databases searched by the reviews.docx]

**Summary of databases searched for peer-reviewed literature**

| **Peer reviewed literature** | | | |
| --- | --- | --- | --- |
| **Databases** | **f** | **Databases** | **f** |
| MEDLINE | 46 | ERIC | 37 |
| PubMed | 37 | CINAHL | 37 |
| Scopus | 34 | Embase | 32 |
| Web of Science | 26 | Cochrane Library | 25 |
| (CENTRAL) Cochrane Register of Controlled Trials | 12 | Google scholar | 10 |
| SocINDEX | 15 | JSTOR | 7 |
| Sociological Abstracts | 5 | (ASSIA) Applied Social Science Index and Abstracts | 5 |
| EBSCO host | 5 | ProQuest | 5 |
| ScienceDirect | 4 | SPORTDiscus | 4 |
| PsycARTICLES | 4 | Social WorkAbstracts | 3 |
| Global Health | 3 | AMED (Allied and Complementary Medicine Databas) | 3 |
| Social Care Online | 3 | Education Research Complete | 2 |
| Social Service Abstract | 2 | Cochrane Reviews | 2 |
| Academic Search Premier | 2 | (DARE) Database of Abstracts of Reviews of Effects | 2 |
| Social Science Citation Index | 2 | Academic Search Complete | 2 |
| Anthropology Plus | 2 | ProQuest Social | 2 |
| BIDS International Bibliography of Social Sciences | 2 | ICTRP | 2 |
| Clinical trials | 2 | POPLINE | 2 |
| British Education Index | 2 | Family and Society Studies Worldwide | 2 |
| Environment Complete | 1 | Campbell Collaboration (C2) databases | 1 |
| Psychology and Behavioral Sciences Collection | 1 | Nursing and Allied Health | 1 |
| ProQuest Sociology Collection | 1 | SID | 1 |
| ProQuest Arts and Humanities | 1 | African Index Medicus | 1 |
| Health Management Information Consortium (HMIC) | 1 | Sage full text psychological collection | 1 |
| Institute of statistical information proceedings of conference and seminars | 1 | Project Muse | 1 |
| Web of Knowledge | 1 | PsychLit | 1 |
| Google | 1 | CINCH | 1 |
| SciELO Citation Index | 1 | Child Development & Adolescent Studies | 1 |
| Current contents connect | 1 | Book citations web of science | 1 |
| Child and adolescent studies | 1 | Book citations scopus | 1 |
| Full text APA | 1 | Medline in process | 1 |
| Cochrane Airways Group Specialised Register (CAGR) | 1 | PsycEXTRA | 1 |
| Australian Education Index | 1 | A+ Education | 1 |
| Magiran and Irondoc | 1 | Cochrane Economic Evaluations | 1 |
| ACP Journal Club | 1 | Wiley Online | 1 |
| Cochrane Methodology Register | 1 | specialized bibliographic registers | 1 |
| Database of Abstracts of Reviews of Effects | 1 | DoPher | 1 |
| Health Technology Assessment | 1 | Psyindex | 1 |
| NHS Economic Evaluation Database | 1 | SpringerLink | 1 |
| Ovid OLDMEDLINE | 1 | Pre-Medline | 1 |
| Family studies abstracts | 1 |  |  |

**Summary of databases searched for grey literature**

| **Grey literature search** | | | |
| --- | --- | --- | --- |
| **Sources of grey literature** | **f** | **Sources of grey literature** | **f** |
| Ref lists | 30 | Websites of organizations | 13 |
| Journals | 11 | Experts | 10 |
| Google | 5 | Conf publications | 4 |
| OpenGrey | 3 | Citation chaining | 3 |
| Databases | 2 | Other sources | 6 |
| NS | 2 | N/A | 15 |
| NR | 37 |  |  |
