# Supplementary material for: An umbrella review of reviews on challenges to meaningful adolescent involvement in health research
Source: Health Expect. 2024 Jan 27;27(1):e13980. doi: 10.1111/hex.13980 (PMC10821743; doi:10.1111/hex.13980)
Supplement: Supplementary file 1 — Supporting information. [file HEX-27-e13980-s001.zip › Study Selection/Prior flowchart.docx]

Duplicate records removed before screening (*n* = 1130)

**Identification of reviews via other methods**

**Included**

Records identified (*n* = 5480)

1. PROSPERO (*n* =123)
2. 470 Websites (*n* =11)
3. Suggested by experts (*n* = 7)
4. Protocol papers (*n* =132)
5. Reference lists & Connected papers website (*n* = 5207)

Records screened (*n* = 7903)

Records excluded

(*n* = 7699)

Records assessed for eligibility (*n* = 204)

Records excluded (n = 157)

- Age range (*n* = 111)
- Not adolescent involvement (*n* =1)
- Not health research (*n* = 19)
- Not a review (*n* = 13)
- Not in English (*n* = 3)
- Could not access full text data extraction tables (*n* = 8)
- Duplicate identified at full text screening stage (*n* = 2)

Records assessed for eligibility

(*n* = 138)

Records excluded (*n* = 86)

- Age range (*n* =15)
- Not adolescent involvement (*n* =24)
- Not health research (*n* =19)
- Not a review (*n*=6)
- Could not access data extraction tables (*n*=2)
- Could not access the full text (*n*=20)
  - Authors refused to share data (*n*=6)
  - Authors did not respond (*n*=4)
  - Full text not available (*n*=10)

Reviews included

(*n* = 47)

Reviews included

(*n* = 52)

Total reviews included (*n* = 99)

Records excluded

(*n* = 5170)

Records screened (*n* = 5308)

Records identified (*n* = 9033)

1. Medline (*n* = 371)
2. PsycInfo & PsycArticles (*n* = 907)
3. Embase (*n* = 864)
4. Scopus (*n* = 104)
5. CINAHL (*n* = 2174)
6. Epistemonikos (*n* = 105)
7. Health Systems Evidence (*n* = 142)
8. Cochrane (*n* = 1320)
9. ProQuest (*n* = 394)
10. Web of Science (*n* = 307)
11. Google Scholar (*n* = 1117)
12. 10 Journals (*n* = 1228)

**Identification**

Duplicate records removed before screening (*n* =172)

**Screening**

**Identification of reviews via databases and registers**
