# Supplementary material for: An umbrella review of reviews on challenges to meaningful adolescent involvement in health research
Source: Health Expect. 2024 Jan 27;27(1):e13980. doi: 10.1111/hex.13980 (PMC10821743; doi:10.1111/hex.13980)
Supplement: Supplementary file 1 — Supporting information. [file HEX-27-e13980-s001.zip › Data extraction & analysis/Characteristics of included reviews.docx]

**Characteristics of reviews**

| **Review article** | **Review type** | **Year** | **Age range** | **Health area** | **Aims and objectives of the review** | **Youth involvement terminology** | **Youth involvement definition** | **Databases searched** | **Grey literature sources** | **Eligibility criteria** | **Quality assessment or Risk of bias method** |
| --- | --- | --- | --- | --- | --- | --- | --- | --- | --- | --- | --- |
| Bessaha, M., Hayward, R. A., & Gatanas, K. (2022). A scoping review of youth and young adults' roles in natural disaster mitigation and response: considerations for youth wellbeing during a global ecological crisis. *Child and adolescent mental health*, *27*(1), 14-21. | Scoping | 2021 | 15 to 29 | Climate change | To identify key elements of natural disaster mitigation and response efforts to encourage and empower young people's involvement as well as connectivity with their peers and communities in mitigation and response. | Youth engagement | Youth engagement as explicated by Frank (2006) including giving authentic responsibility and voice to youth participants, building youth capacity as agents of change, encouraging flexible work styles, and involving adults in the process for support and mentorship. | Web of Science, PsycINFO, Social WorkAbstracts, ERIC, and Environment Complete | NR | Excluded publications not written in English, published before 1990, and that did not include a sample of youth or young adults. | NR |
| Sinclair, R. (2004). Participation in practice: Making it meaningful, effective and sustainable. *Children & society*, *18*(2), 106-118. | Narrative | 2004 | <18 | Overall research | To provide a review of children's participation as practised today, by setting the context, mapping briefly some of the influences that have promoted acceptance of children's participation and some of the frameworks that have been used to disentangle how the concept of participation is understood in practice. | Participation | Participation is multi-dimensional. Here we consider four key dimensions for understanding participation: the level of participation; the focus of the decision-making in which children may be involved; the nature of participation activity; and the children and young people involved | N/A | N/A | N/A | NR |
| Jørgensen, C. R. (2019). Children’s Involvement in Research—A Review and Comparison with Service User Involvement in Health and Social Care. *Social Sciences*, *8*(5), 149. | Literature | 2019 | <18 | Health research | To bridge some of the dichotomies between children and adults and different areas of work by explicitly comparing key themes found in the literature on children's involvement with insights from the involvement of service users. | Involvement | Children's involvement: Involvement activities are defined as those that include people (children and service users) taking part in research advisory or steering groups, advising on research topics or design, carrying out data collection and analysis, and/or taking part in dissemination. | Web of Science, ERIC and Scopus | The reference lists of the selected papers and by browsing the online library of publications provided by the British national organisation for public involvement in health and social care research (INVOLVE). | Papers were included if they described projects which involved children up to the age of 18 (as specified in the papers or deduced from the context) as co-researchers or advisors (as per the definition of involvement described above) or position papers about the practice of involving children. Only peer-reviewed literature in English was included, and, in the case of research papers, only those discussing work conducted in the UK were selected. | NR |
| Gavine, A. J., Aleman-Diaz, A. Y., Currie, C. E., Humphris, G. M., Morgan, A. R., & Garcia-Moya, I. (2017). The engagement of young people in the development and implementation of programmes to secure health: a systematic review. | Systematic | 2017 | 5 to 18 | Health research | To summarise through a systematic review, the scientific evidence base the benefits of the involvement of children and young people in the development, implementation and evaluation of programmes aiming to secure health. | Participatory research, community engagement | Participatory research or community engagement, involving communities in decision-making and in the planning, design, governance and delivery of services. | Applied Social Science Index and Abstracts (ASSIA) Campbell Collaboration (C2) databases Cochrane Register of Controlled Trials (CENTRAL) Cumulative Index to Nursing and Allied Health Literature (CINAHL) Database of Abstracts of Reviews of Effects (DARE) Education Resources Information Centre (ERIC) Medline PsycInfo Social Care Online Social Science Citation Index | The grey literature was searched using two approaches. First, the database Open Grey was searched and secondly, websites which were identified as being relevant to youth participation were searched for any relevant material. A call for evidence was put out to the Health Behaviour in School Children (HBSC) Youth Engagement group, whereby members were asked to identify any studies that they were aware of that could answer any of RQ. However, there was no response to the call. | Studies were included if they involved young people in any aspect of the programme development, delivery or evaluation. More specifically programmes were included if they involved young people in any of the following domains: ï‚· Issue identification (i.e. actively involved in determining focus of programme); ï‚· Needs assessment (i.e. conducting research to explore the issue of interest); ï‚· Programme development (i.e. contributed to develop of programme and/or materials); ï‚· Delivery of programme (i.e. facilitating or teaching sessions, outreach work); ï‚· Programme evaluation (i.e. actual conduct of the evaluation). Programmes targeting individual young people who have a specific clinical condition will be excluded. However, if the intervention aims to target risk factors for a clinical condition (e.g. diabetes, asthma) at a population level it will be included. Programmes that involved young people in programme development, delivery or evaluation but did not have a focus on health were excluded. | Effective Public Health Practice Project quality assessment tool for quantitative studies (Thomas et al., 2004) and the EPPI centre tool (EPPI-Centre, 2007). |
| Fløtten, K. J., Guerreiro, A. I. F., Simonelli, I., Solevåg, A. L., & Aujoulat, I. (2021). Adolescent and young adult patients as co‐researchers: A scoping review. *Health Expectations*, *24*(4), 1044-1055. | Scoping | 2021 | >12 | Health research | To summarize empirical evidence and identify knowledge gaps about the involvement of young patients as co-researchers. | Co-research | By co-research, we refer to full participation in the research team, that is levels 4 and 5 of the simplified participation ladder. | MEDLINE, EMBASE, PsychINFO and AMED | All co-authors were asked to consider whether they knew other publications that should be reviewed. The reference lists of the included articles were hand-searched. | The following inclusion criteria were used: articles concerning patients 12 years and above; patients are co-researchers; the research topic is in relation to health care and health systems research; describes 4th and 5th level of participation according to the participation ladder as amended by Teunissen and referenced by de Wit et al 3; and publication date 2000 - current. | A protocol designed by Kmet et al |
| BAILEy, S., BODDy, K., Briscoe, S., & Morris, C. (2015). Involving disabled children and young people as partners in research: a systematic review. *Child: care, health and development*, *41*(4), 505-514. | Systematic | 2015 | 5 to 25 | Health research | 1 To find out how DCYP have been accessed, recruited or selected for involvement in research projects. 2 To investigate how the practicalities of involving DCYP in research have been addressed. 3 To identify the challenges of involving DCYP in research and how have these been overcome. 4 To describe the impacts of involving DCYP in research on the disabled children themselves. 5 To describe the impacts of involving DCYP in research on the research. | Public and patient involvement (PPI) | Public and patient involvement (PPI) is defined by INVOLVE (2013) as:  research being carried out "with" or "by" members of the public rather than "to", "about" or "for" them. | MEDLINE (Ovid); MEDLINEin-process (Ovid); CINAHL (EBSCO); ASSIA (ProQuest); Cochrane Database of Systematic Reviews; DARE (via Cochrane) | Forward and backward citation chasing was carried out on included papers to supplement the database searches. In addition, relevant websites were searched. | The search aimed to find any study design in which DCYP were reported as being involved as partners in research, or reviews that had addressed this type of involvement. Peer reviewed papers were sought, alongside guidelines, reports or other documents from the grey literature. Studies must have involved DCYP aged 5-25 as partners in health research in one of the acknowledged approaches to involvement (INVOLVE 2013). A non-categorical approach was taken; the Equality and Human Rights Commission (1995) definition of disability was used: a physical or mental impairment which has a substantial and long-term adverse effect on his ability to carry out normal day-to-day activities. Examples include cancer, diabetes, multiple sclerosis and heart conditions; hearing or sight impairments, or a significant mobility difficulty; and mental health conditions or learning difficulties. | Critical Appraisal Skills Programme (CASP) qualitative checklist (CASP 2013) |
| Allsop, M. J., Holt, R. J., Levesley, M. C., & Bhakta, B. (2010). The engagement of children with disabilities in health-related technology design processes: Identifying methodology. *Disability and Rehabilitation: Assistive Technology*, *5*(1), 1-13. | Narrative | 2010 | N/A | Health research | To identify research methodology that is suitable for involving children with disabilities in the design of healthcare technology, such as assistive technology and rehabilitation equipment | Research with children and research by children | N/A | N/A | N/A | N/A | NR |
| Bevan Jones, R., Stallard, P., Agha, S. S., Rice, S., Werner‐Seidler, A., Stasiak, K., ... & Merry, S. (2020). Practitioner review: co‐design of digital mental health technologies with children and young people. *Journal of Child Psychology and Psychiatry*, *61*(8), 928-940. | Practitioner review | 2020 | <18 | Mental health | To provide a practitioner review of the literature on the approaches to the design and development of digital mental health technologies in collaboration with CYP and other stakeholders. | Co-design | Co-design is a process of collective creativity or partnership with potential users and stakeholders, who are actively involved across the entire development of the technology helping to ensure it meets the user-needs and preferences | Medline, PsycInfo and Web of Science databases | Searched reviews, guidelines and reference lists, and contacted key authors with expertise in the development of digital mental health interventions for CYP, especially where it was unclear whether CYP were involved in the design/development of certain technologies | Inclusion criteria: articles with information on the (co-) design/development/production, of digital mental health technologies with and for CYP (up to 18 years); papers published or translated into English in a peer-reviewed journal. There was a focus on programmes/applications to help with depression, anxiety, sleep, self-harm and suicide | NR |
| Raanaas, R. K., Bjøntegaard, H. Ø., & Shaw, L. (2020). A scoping review of participatory action research to promote mental health and resilience in youth and adolescents. *Adolescent Research Review*, *5*(2), 137-152. | Scoping | 2020 | 10 to 29/30 | Mental health | The primary question for this exploration and synthesis of the literature was "How are PAR studies conducted to promote mental health and resilience in youth and adolescence and how are the youth/adolescents involved?" | Participatory Action Research (PAR), Participatory Research | NR | Scopus, Web of science, Pubmed, PsychINFO, Cinahl, Eric, Sociological Abstracts, Cochrane Library, Academic Search Premier | NR | Youth, adolescents, or people in the age group 10-30, mental health or resilience was defined as primary outcome, and the study was described as using a PAR approach, scientific peer review articles written in English or a Scandinavian language were included. Empirical studies were included that had clearly defined methods that involved youth participating in research processes consistent with PAR approaches. Theoretical articles or empirical studies were excluded that did not report on outcomes and focused on methods or ethical issues. Evaluations of PAR programs were not included where the PAR method in itself was not described. Related to the concepts of mental health and resilience, studies were excluded where the primary aim was on some kind of life style change, or predefined protective factors even though mental health or resilience may have been mentioned. For instance, these types of studies were excluded: a primary focus on physical activity, healthy eating, safe drinking, tobacco behavior, drug use prevention, safe sexual behavior, preventing teenage pregnancy, promoting healthy computer use, or access to health services. Studies that focused on young mothers were excluded when the aim was children’s health, or mothering abilities, and not specific to mental health and resilience. Finally, studies that focused on trauma prevention following environmental issues were excluded. | NR |
| Haijes, H. A., & van Thiel, G. J. (2016). Participatory methods in pediatric participatory research: a systematic review. *Pediatric research*, *79*(5), 676-683. | Systematic | 2016 | <18 | Pediatric research | To describe and evaluate the available knowledge on participatory methods in pediatric research. | Participatory pediatric research | Participatory pediatric research was defined as research which actively involves children in defining relevant research questions and in the design and conduct of studies. Participatory methods were defined as any method that can be used to obtain children' views, aiming to involve them in the design and conduct of research. | PubMed, CINAHL, PsycINFO, Scopus, and Cochrane databases | A hand search was carried out on bibliographies of included papers | Inclusion criteria were pediatric medical research and the active involvement of children in the design and conduct of the study. Moreover, the articles must contain at least a description of the participatory method used. | NR |
| Alazmah, A. S. A. (2016). *Involving Children In Oral Health Research: Are We Doing Enough?* (Doctoral dissertation, UCL (University College London)). | Systematic | 2016 | <12 | Oral Health | To determine existing methods used to engage with children in order to develop and shape research design and ideas. | Research with children | When children's actual opinions, perspectives, perceptions and contribution are taken in consideration, this is defined as research with children (Punch, 2002). research being carried out "with" or "by" members of the public rather than "to", "about" or "for" them. | PubMed, Scopus, Web of science and Medline Ovid | A manual search for systematic and narrative reviews on the topic was done of the following journals: British Dental Journal, International Journal of Paediatric Dentistry, European Journal of Paediatric Dentistry, Journal of Paediatric for Children Health, Health and Human Rights, Health and Society, Paediatrics, Health, Culture and Society, Children and society, Childhood, Adolescent health, medicine and therapeutics, Journal of Adolescence and Journal of Adolescent Health.Reference lists of eligible studies were screened for further eligible studies. | Inclusion criteria  Any studies describing how children were involved in the design or methodological process of a study in a health related setting. -Any studies describing how children were involved in the development of research ideas or themes in a health related setting. - Studies were included irrespective of design or type. Both quantitative and qualitative studies were included.  Exclusion criteria  Articles targeting or involving parents or caregiver or any other adults only. - Articles that aim to educate, teach or motivate children. - Articles that involve children in any of the research stages rather than design. - Articles related to quality of life. - An article does involve children in the design of the study with not enough details. - Studies that are not English language. | NEWCASTLE - OTTAWA QUALITY ASSESSMENT SCALE (Wells GA. et al, 2000) |
| Preston, J., Stones, S. R., Davies, H., Preston, J., & Phillips, B. (2019). How to involve children and young people in what is, after all, their research. *Arch Dis Child*, *104*(5), 494-500. | Narrative | 2019 | N/A | Health research | To concisely review PPI in research involving CYP, with the main objective in providing practical guidance for researchers to build good PPI with CYP into their worK and to support reviewers of research applications in assessing this important part of the research proposal. | Patient and public involvement (PPI) | According to INVOLVE, involvement is defined as ‘research being carried out with or by CYP, rather than to, about or for them | N/A | N/A | N/A | NR |
| van Schelven, F., Boeije, H., Mariën, V., & Rademakers, J. (2020). Patient and public involvement of young people with a chronic condition in projects in health and social care: a scoping review. *Health Expectations*, *23*(4), 789-801. | Scoping | 2020 | 12 to 25 | Chronic health conditions | To gain insight into how PPI with YPCC in projects in health and social care is conceptualized in the existing literature and possible shifts herein. | Patient and public involvement (PPI) | Patient and Public Involvement: To carry out research projects (eg scientific projects aimed at increasing knowledge) and implementation projects (eg practice-oriented projects aimed at developing, for example, tools and interventions) together with rather than about or for them | Cinahl, Embase, PsycINFO, PubMed and Scopus | NR | Studies were included in the review, if they:  1. Addressed YPCC; a. Young people being defined as people aged 12-25; b. Chronic condition being defined as conditions that last or are expected to last twelve or more months and result in functional limitations and/or the need for ongoing medical care. 2. reported on PPI in research or implementation projects. 3. contained empirical data. 4. were written in English. 5. were published after 1990 (the year after the ratification of the UNCRC | Critical Appraisal Skills Program (CASP), NIH Quality Assessment Tool for Observational Cohort and Cross-Sectional Studies, the Mixed Methods Appraisal Tool (MMAT) |
| Rouncefield-Swales, A., Harris, J., Carter, B., Bray, L., Bewley, T., & Martin, R. (2021). Children and young people’s contributions to public involvement and engagement activities in health-related research: A scoping review. *PloS one*, *16*(6), e0252774. | Scoping | 2021 | <25 | Health research | To identify, synthesise and present what is known from the literature about patient and public involvement and engagement activities with children and young people in health related research. | Patient and public involvement and engagement (PPIE) | Our working definition for PPIE with children and young people for this review builds on the previously mentioned INVOLVE definition. "Health related research being carried out with or by children and young people rather than to, about or for them; or, where information and knowledge about research is provided and disseminated" | Scopus, Medline, CINAHL, Cochrane and PsychInfo | The reference lists of included documents were reviewed for additional papers. A search of the grey literature (pdf files and webpages), including a hand search, was also completed in December 2019. Grey literature was identified via Open Grey, Google and from the websites of the Royal College of Paediatrics and Child Health, Royal College of Nursing, Barnardo's, Department of Health, National Institute of Health Research Portfolio, Childlink, WellChild, Shine, Generation R, UNICEF, Save the Children, INVOLVE and iCan for research and conference publications | Inclusion criteria - PPIE activities occurred with children and young people aged 0-24 years. - General population of children, young people and young adults: but will not exclude studies which solely focus on certain chronic conditions or specific population groups as long as they meet the other inclusion criteria. - Empirical and descriptive studies. - Systematic reviews and meta-analysis (and those studies included in these reviews/analyses) - Health related research studies (including disability research). - Full text available in English.  Exclusion criteria - Studies that do not include children or young people in PPIE activities. - Studies focused on health service design. - Book reviews, opinion pieces, unpublished theses and literature reviews. - Articles in press. | QRIPPAT (Quality of Reporting Involvement of Patients and the Public Appraisal Tool). |
| Alwadi, M. A., Baker, S. R., & Owens, J. (2018). The inclusion of children with disabilities in oral health research: A systematic review. *Community Dentistry and Oral Epidemiology*, *46*(3), 238-244. | Systematic | 2018 | <16 | Oral Health | To explore the extent to which contemporary oral health research has been conducted with or on children with disabilities. | Research with children | Categories of involvement that were developed by Marshman and colleagues (2007)  1. The first category included research with children with disabilities where children were seen as active participants sharing power and responsibility for the research design and process. Children with disabilities are included in all aspects of the research process from generating the questions through to the design as well as being involved in data collection and dissemination. Children are viewed as equals.  2.The second category, also classified as research with children with disabilities, where children are involved in the design and conduct of research. Children worked within pre-arranged focus and research questions and were involved in choices of methods and ways of carrying out research.  3.The third category included research with children with disabilities where their perspectives were taken into consideration around research design. The children would express their views around the research design and were involved in steering groups  4.The fourth category also comprised research with children with disabilities and comprised studies where they were listened to and seen as subjects of research with adults deciding the research questions, design and methods. This category was subdivided into 2 subcategories. The first subcategory included studies where children with disabilities contributed by giving an account of their experiences in their own words (subcategory 4a), using methods for example qualitative interviews, in-depth, unstructured and semi-structured. The second subcategory-contained studies where children with disabilities completed measures designed by adults (subcategory 4b) for example, structured interviews, questionnaires and other scales.  5. The fifth category included studies that used others as proxies for children with disabilitiesâ€”another person reporting on their oral health either parental/caregiver (category 5a) or clinician(category 5b). Using proxies was felt appropriate for children too young or sick or with a profound level of intellectual disability  6.The final category included research on children with disabilities, where they were seen as the objects of research. In this research, children with disabilities were not consulted in any way but only seen. For example, the research included within this category might see them either as; a mouth or a set of teeth to be treated; source of a sample of plaque, saliva or soft/hard tissues; recipient of an intervention such as oral health promotion;population group tobe examined clinically; particular group to be managed and finally a patient on whom aâ€œparticu-lar examinationâ€ was done. | Scopus, Web of Science, Google Scholar | NR | Exclusion criteria;  Studies reported before 2001 because this was prior to the conceptual model of the International Classification of Functioning, Disability and Health andâ€œValuing People in the UK; studies with participants over 16 years of age; studies that do not have children with disabilities and/or aspectsof their oral health as their main topic; studies with no primary data, case reports, conference proceedings and guidance documents | NR |
| Griebler, U., Rojatz, D., Simovska, V., & Forster, R. (2017). Effects of student participation in school health promotion: a systematic review. *Health promotion international*, *32*(2), 195-206. | Systematic | 2017 | 5 to 19 | Health research | A systematic identification of the existing evidence for effects of student participation in school-based health promotion | Participation | We defined student participation as practices that involve collaboration between students and various groups of actors concerning health-related issues in order to influence decision-making regarding designing, planning, implementation or evaluation of health promotion measures (Potvin, 2007). The review endorses the understanding of participation that distinguishes between a tokenistic and a genuine participation quality. Genuine student participation is defined as having influence over the decisions and activities in the school health promotion processes, rather than simply as taking part in them (Simovska, 2007; Hart, 2008). According to this conceptualization, simple forms of student participation such as answering questions and taking part in activities (e.g. sports or music) are not considered participation. We also do not confine participation to individual decision-making (as in curricular choices) but rather see it as a collaborative process. | ASSIA, ERIC, PsycINFO, Scopus, PubMed and the Social Sciences Citation Index | Hand searched all issues (from December 2009 to February 2011) and supplement issues (from 1992 onwards) from selected journals and the reference lists of pertinent articles and contacted experts to provide relevant unpublished studies. | Inclusion criteria   - All types of empirical studies or reports describing empirical research about student participation in decision-making about health promotion measures at school were included  - All reports published in English, German and Danish were considered  - Children and adolescents, in any country, aged 5-19, who attended primary or secondary school, private or public schools  - All papers that describe health promotion measures in the setting school were considered  - All reports describing student participation in designing, planning, implementing and/or evaluating school health promotion were considered  - Effects of student participation in designing, planning, implementing and/or evaluating school health promotion measures on the participating students, on other students, on the health promotion measure or on the school were considered    Exclusion criteria  -Publications describing anecdotal experience/not empirical studies  -Books and other grey literature were excluded except if suggested by experts  -Publications describing participation of other populations (university or college students, kindergarten children, teachers, parents, community etc.)  - Publications describing student participation in another setting than school (e.g. community health promotion)  - Publications not describing student participation according to our definition, i.e. students taking part in health promotion, but not involved in planning, designing, implementing and/or evaluating the health promotion measure  - Articles describing participation rates or interventions to increase participation rates in health promotion programmes, without involvement of students in the planning or organizing phase of the project  - Publications describing individual decision-making of students (e.g. choosing sports lessons, etc.).  - Publications without scientific quantitative or qualitative measure of any effects of student participation | For rating the quality of studies we used checklists for both qualitative and quantitative studies, developed earlier by the first author of this paper. The following criteria were the basis for our quality rating: a clear research question; an appropriate empirical research approach; a clear description of appropriate sampling, data collection and data analysis procedures; a clear description of the study context; the findings; the value of the research; ethical issues and reflexivity (consideration of potential researcher bias). |
| Beck, A. J., & Reilly, S. M. (2017). What can secondary school students teach educators and school nurses about student engagement in health promotion? A scoping review. *The Journal of School Nursing*, *33*(1), 30-42. | Scoping | 2017 | NR | Health research | To examine student engagement in health promotion initiatives in school settings | Involvement | Meaningful student involvement is the process of engaging students as partners in every facet of school change for the purpose of strengthening their commitment to education, community and democracy (Fletcher, 2005, p. 5). | CINAHL, Education Research Complete, ERIC, MEDLINE, Psychology and Behavioral Sciences Collection, SocINDEX, and SPORTDiscus | Gray literature searches from governmental and nongovernmental websites, electronic databases (Canadian Health Research Collection, Canadian Best Practices Portal, Canadian Public Policy Collection and Google), and studies that appear in reference lists. Although the CSH movement began around 2008, the search includes literature published between 2000 and 2015 | All of the qualitative and quantitative studies were eligible for inclusion if they met the following criteria: (1) population included adolescents in secondary school without clinical or developmental disorders, (2) the intervention included any extracurricular activity or project that encouraged student engagement or meaningful participation outside the traditional academic curriculum, (3) the intervention took place in the school community, (4) school-based activities that assessed and evaluated the effectiveness of facilitating adolescent engagement defined the outcomes, (5) the study appeared in English, (6) and the study should have been peer-reviewed. The review excludes studies that had specialized populations with either a clinical or a developmental disorder, used projects or interventions that lacked student involvement, or did not deliberately seek meaningful outcomes. | NR |
| Gibbs, L., Kornbluh, M., Marinkovic, K., Bell, S., & Ozer, E. J. (2020). Using technology to scale up youth-led participatory action research: A systematic review. *Journal of Adolescent Health*, *67*(2), S14-S23. | Systematic | 2020 | 10 to 19 | Health research | To address (1) what forms of technology were used to scale up the participatory research and how were they used and (2) how were the participatory elements of the research maintained within that process? | Youth-Led Participatory Action Research (YPAR) | Youth-Led Participatory Action Research (YPAR): Participatory research approaches involving adolescents in the cocreation of knowledge and advocacy and generation of subsequent health and social change outcomes. | PsycARTICLES, PsycINFO, and PubMed | NR | Articles were included if they were (1) empirically reviewed, (2) in English, (3) published between 2000 and 2018, (4) consisted of adolescent participants (aged 10-19 years), and (5) explicitly discussed using technology to scale up YPAR efforts. Social media have only been developed and accessible to the public within the last two decades; thus, our sample was restricted to the period between January 2000 and 2018. Our search focused on adolescents, with sample participants between 10 and 19 years old. Finally, projects that used technology for internal project activities rather than to extend the reach and impact of the project were excluded from the review. | NR |
| Bray, E. A., Everett, B., George, A., Salamonson, Y., & Ramjan, L. M. (2021). Co-designed healthcare transition interventions for adolescents and young adults with chronic conditions: a scoping review. *Disability and Rehabilitation*, 1-22. | Scoping | 2021 | 10 to 32 | Chronic health conditions | To identify the scope of literature on co-designed HCT interventions for AYA with chronic conditions. | Co-design, Participatory Action Research (PAR) | Co-design is a process, a set of practical tools and a set of principles that allow end-users to collaborate, cooperate or connect knowledge, skills or resources to complete a design task, where engagement may range from passive to active involvement.  Participatory action research (PAR) is a methodological approach to research that may include elements of co-design to varying degrees. It is a research approach that promotes reflection and action and seeks to empower those who are most affected, such that the participant is a collaborative partner ideally from the onset and is involved in improving practices or situations to meet contextual and community needs | Scopus, CINAHL, Medline-Ovid, Cochrane and PsycINFO | Reference lists of key literature were also searched. Grey literature (e.g., conference proceedings, theses, working papers, book chapters and research reports) were excluded. | Included studies described a HCT intervention for AYA with a chronic condition transferring from the paediatric to adult healthcare system. Studies that focused on the general transition experience of AYA or the transition of AYA with chronic conditions within the community (e.g., from school to university/work or to living out of home) were not included. In addition, studies that focused only on self-management skills without reference to a transition model, intervention or program were also excluded. Only those that used a co-design method or that applied elements of co-design in their development such as exploring the current services offered and the experiences of patients, capturing this experience, working together to understand it, and improving it were included. No limitations were placed on study design, quality or location. Only peer reviewed studies published in English were eligible for inclusion. No restrictions were placed on publication dates, as the aim was to retrieve all published peer reviewed research until the date of search (i.e., studies retrieved until May 2020). | Joanna Briggs Institute (JBI) checklists |
| Doyle, A. M., Majozi, N., Simwinga, M., Mayingire, G. R., Simbeye, K., Dringus, S., & Bernays, S. (2021). Engagement with young people as partners in health research: four case studies from Sub-Saharan Africa. *Tropical medicine & international health: TM & IH*. | Targeted | 2021 | 10 to 24 | Health research | To provide practical examples of engagement of YP in health research in Sub-Saharan Africa and to generate critical reflective learning to inform the development of best practice going forward. | Youth involvement and engagement | Youth involvement, youth engagement : Global Consensus Statement on Meaningful Youth Engagement emphasise that engagement should be rights-based, transparent and informative, voluntary and free from coercion, respectful of young people's views, backgrounds, identities, and safety | N/A | N/A | N/A | NR |
| Fountain, S., Hale, R., Spencer, N., Morgan, J., James, L., & Stewart, M. K. (2021). A 10-Year Systematic Review of Photovoice Projects With Youth in the United States. *Health Promotion Practice*, *22*(6), 767-777. | Systematic | 2021 | 8 to 26 | Health research | To identify common practices and lessons learned and to examine to what extent projects conducted with youth and described as photovoice in the literature were able to address these goals. | Photovoice | Photovoice includes teaching the participants photography skills (ethics, safety, and camera basics), using SHOWeD or a similar method to construct the caption for their photo, participating in group discussion to find predominant themes within all the photos, and holding a forum to display the photos for community members, city officials, and/or policy makers. | PubMed | NR | Ten of the 80 articles originally pulled were excluded because they were published before 2010 leaving 70 articles for abstract review. Eleven articles were excluded during this stage and another 14 were excluded after full-text assessment. The majority of the articles that were excluded were not implemented with youth. The next most common reason for exclusion was that the photovoice project took place outside the United States.We included all articles that described their projects as photovoice, rather than applying a strict definition such as requiring them to have addressed all three of Wang and Burris original defining goals. | NR |
| Benjamin-Thomas, T. E., Laliberte Rudman, D., Cameron, D., & Batorowicz, B. (2019). Participatory digital methodologies: Potential of three approaches for advancing transformative occupation-based research with children and youth. *Journal of Occupational Science*, *26*(4), 559-574. | Critical review | 2019 | NR | Occupation related health research | To examine the utility of digital storytelling, participatory videos, and participatory geographic information systems (PGIS) for transformative research with children and youth that addresses occupation. | Participatory methodologies, Participation | Participatory methodologies strive to involve community members facing injustices as coresearchers in all phases of projects, from identification of issues to execution of socially transformative actions (Cargo & Mercer, 2008). Participation involves on-going negotiation of power between researchers and community members, working towards shared understanding, dialogue, and social action or transformation (Grimwood, 2015). | Anthropology Plus, Academic Search Complete, CINAHL, ERIC, JSTOR, Nursing & Allied Health, ProQuest Sociology Collection, ProQuest Arts and Humanities, PsychINFO, PubMed, Scopus, Web of Science | NR | Inclusion criteria required that articles were: a) a research article; b) positioned in relation to a transformative research agenda; c) applied one of the 3 digital methodologies; d) carried out among children/youth, inclusive of cultural variation in age limits identified by authors; e) addressed an issue relevant to occupation (everyday things that people do as individuals or as collectives); f) published between January 2000-March 2017; and g) written in English. | NR |
| Bradbury-Jones, C., Isham, L., & Taylor, J. (2018). The complexities and contradictions in participatory research with vulnerable children and young people: A qualitative systematic review. *Social Science & Medicine*, *215*, 80-91. | Systematic | 2018 | 0 to 24 | Research with vulnerable or marginalized children | To identify, synthesize, and critically examine published literature that reported on the methodological, ethical and practical issues involved in carrying out participatory research with vulnerable or marginalized children. | Co-production | For the purpose of this article, co-production is taken as the underpinning principle on which participatory research is based. | Cumulative Index of Nursing and Allied Health Literature (CINAHL); Embase; Health Management Information Consortium (HMIC), Institute of Statistical Information (ISI) Proceedings of conferences and seminars; Medline and Scopus | In October 2017, we carried out additional 'hand-searching' of relevant journals to identify literature that was not picked up or indexed within major search databases. | Inclusion  1. Empirical, qualitative study. 2. Explores research carried out with children or led by children. This may be defined as co-research, participatory research, participatory action research, peer research or another synonym. 3. Focuses on an aspect of vulnerability/marginalisation. This includes: children in care and/or who have experienced abuse, neglect or violence; disabled children; children with illness/mental health issues; LGBTQ young people. 4. A significant focus of the paper is critical or evaluative, discussing and/or reflecting on participatory research with children (e.g. ethical, methodological, practical challenges, and issues).  Exclusion 1. Discussion papers, literature reviews, mixed-methods studies, conference abstracts, books, book chapters, conference posters, and other grey literature. 2. Research reports on the findings of a participatory study without significant focus on the ethical, methodological, practical challenges and issues. 3. Explores general ethical and methodological issues about children 'participating' in research (e.g. recruitment, engagement, ethics, etc.). | NR |
| Sullivan, E., Egli, V., Donnellan, N., & Smith, M. (2021). Policies to enable children’s voice for healthy neighbourhoods and communities: A systematic mapping review and case study. *Kōtuitui: New Zealand Journal of Social Sciences Online*, *16*(1), 18-44. | Scoping | 2021 | NR | Healthy neighborhoods and community planning | To identify the prevalence of policies and practices including child consultation, engagement, or participation in urban and neighbourhood planning, and identify national and local urban planning policies in NZ that describe the inclusion/exclusion of child participation policies. | Consultation, Codesign, Participation, Engagement | NR | Scopus and PubMed | A manual search of websites of urban and community planning authorities, local boards,and transport authorities in NZ was undertaken. | Studies were eligible at the searching stage if they were: (1) peer reviewed articles published in academic journals, (2) published in the English language, and (3) conducted with human populations.  Studies were included if they detailed an urban or neighbourhood planning policy, recommendation, or process that incorporated child consultation, engagement, or participation in some form. All study types (cross-sectional, natural experiments, prospective, retrospective, experimental, longitudinal studies; quantitative, qualitative and mixed/ multi methods) were included providing they met other inclusion criteria | NR |
| Clavering, E. K., & McLaughlin, J. (2010). Children's participation in health research: from objects to agents?. *Child: care, health and development*, *36*(5), 603-611. | Narrative | 2010 | NR | Health research | To examine ways in which children have been included in health-related studies to identify strengths and weaknesses. | Research with children and research by children. | Not clearly defined | Web of Knowledge | N/A | Searching was designed to capture both work discussing research with children and also exemplary studies in applying such approaches to research activity. We were particularly focused on capturing work which included considerations of both power dynamics and ethical issues within their research approach and design. | NR |
| Boswell, N., & Woods, K. (2021). Facilitators and barriers of co-production of services with children and young people within education, health and care services. *Educational and Child Psychology*. | Systematic | 2021 | <25 | Health research | To explore the facilitators and barriers of the co-production of services with children and young people? | Co-production | This paper adopts a definition of co-production as an equal relationship between people who use services and the people responsible for services. [through which] They work together from design to delivery, sharing strategic decision-making about policies, as well as decisions around the best ways to deliver services. (Think Local Act Personal, 2016. p.2). | Applied Social Science Index and Abstract (ASSIA), Education Resources Information Centre (ERIC) and Google Scholar | Further references were also found from the studies identified through database searches (reference harvesting). | inclusion criteria: - must include CYP and their views and use this to create a sustained change for a service as part of a process of co-production (the study does not need to explicitly use the term co-production). - must be available in the English Language. - must include CYP up to 25 years with SEND or/and up to 18 years with no identified SEND. - must be published between 1 January 2014 and 21 August 2019 encompassing the timespan of the enactment of The Children and Families Act 2014 And one exclusion criterion: - Views being gathered as part of routine service evaluation exercises. | Gough's (2007) Weight of Evidence framework to assess methodological quality (WoE A), methodological appropriateness (WoE B) and relevance of focus (WoE C). |
| Marshman, Z., Gupta, E., Baker, S. R., Robinson, P. G., Owens, J., Rodd, H. D., ... & Gibson, B. (2015). Seen and heard: towards child participation in dental research. *International Journal of Paediatric Dentistry*, *25*(5), 375-382. | Systematic | 2015 | <16 | Oral Health | To explore the extent to which children were involved in the research, the type of studies involving children, the country of origin, and the subject area. | Active participants | Defined as children seen as active participants  a) Including children in the research process  b) In their own words e.g. qualitative interviews, in-depth, unstructured, semi-structured | MEDLINE. Web of Science (core collections) and Scopus | NR | The following exclusion criteria were applied:  - Studies with participants over 16 years of age. - Reports with no primary data. - Conference proceedings. - Articles not having children and aspects of their oral health as their main topic, including laboratory-based studies and studies of craniofacial morphology and injury. - Case reports or case series. | NR |
| Marshman, Z. O. E., Gibson, B. J., Owens, J., Rodd, H. D., Mazey, H. U. W., Baker, S. R., ... & Robinson, P. G. (2007). Seen but not heard: a systematic review of the place of the child in 21st‐century dental research. *International Journal of Paediatric Dentistry*, *17*(5), 320-327. | Systematic | 2007 | <16 | Oral Health | To explore the extent to which contemporary oral health research has been conducted with or on children. The review will enable any deficiencies in approaches to research in this field to be highlighted. | Involvement, children as active participants | Children seen as active participants, children involved in research process, children's own accounts | MEDLINE (via Ovid) and EMBASE | NR | At the first pass through the library of references the following exclusion criteria were applied:  - reports before 2000; - studies with participants over 16 years of age; - studies with no primary data; - articles reporting in vitro studies; - conference proceedings; and - articles that did not have children and aspects of their oral health as their main topic. | NR |
| Sellars, E., Pavarini, G., Michelson, D., Creswell, C., & Fazel, M. (2021). Young people’s advisory groups in health research: scoping review and mapping of practices. *Archives of disease in childhood*, *106*(7), 698-704. | Scoping | 2020 | 12 to 18 | Health research | To provide systematic evidence on the methods and impacts of YPAGs in youth focused health research. | Patient and public involvement (PPI), Young People's Advisory Groups (YPAGS) | Young people's advisory groups (YPAGs) work collaboratively with researchers at different stages of the research process. | MEDLINE | Conducted a hand search of all journal articles published from 1 January to 31 December 2019 in the 20 top ranking Pediatrics, Perinatology and Child Health journals using Scimago Journal and Country Rank for 2018. The search investigated the number of papers in each journal that were empirical, applied health studies focused on the target age range and the number of papers that actually involved a YPAG in their study. We also contacted three experts in the field and accessed the NeurOX YPAG database of studies on YPAGs to try and identify any additional studies | Papers were included if they reported on an empirical health study where the majority (>50%) of research participants were likely to be aged between 12 years and 18 years (from either descriptions of age range or frequencies of ages), and would potentially benefit from a YPAG because the research addressed adolescent applied health research (ie, was not related to the prenatal, perinatal or postnatal period, infancy or reported on basic science research). Editorials, commentaries, viewpoints and papers reporting on secondary data analysis were excluded. | NR |
| Branquinho, C., Tomé, G., Grothausen, T., & Gaspar de Matos, M. (2020). Community‐based Youth Participatory Action Research studies with a focus on youth health and well‐being: A systematic review. *Journal of community psychology*, *48*(5), 1301-1315. | Systematic | 2020 | <25 | Health and wellbeing | To gather information related to the characteristics of community-based YPAR, with a focus on youth health and well-being, as well as their recommendations to improve the development of other community-based YPAR programs, with focus on health and well-being. | Community-based Participatory Action Research, Youth Participatory Action Research (YPAR) | In a conceptualization, Rodriguez and Brown (2009), argue that the YPAR programs are based on three principles: (a) Research topics centered on the youth lives and their concerns; (b) youth participation; (c) transformative character through the change of knowledge and practices that benefit youth lives and their communities. | Web of Science, EBSCO host, and Cochrane Library | NR | The criteria of the YPAR studies to be included were based on the fulfillment of the following: (a) Participatory research of young people; (b) power-sharing with adults; and (c) transformative character through the acquisition of knowledge or with impact on practices to improve youth or their communities (Rodriguez & Brown, 2009)  Studies that were excluded: Population under 25 years; not include conceptualization, intervention, or reviews in the field of health promotion and/or youth well-being; were developed in a school or political context or do not constitute scientific article, were excluded | NR |
| Anyon, Y., Bender, K., Kennedy, H., & Dechants, J. (2018). A systematic review of youth participatory action research (YPAR) in the United States: Methodologies, youth outcomes, and future directions. *Health Education & Behavior*, *45*(6), 865-878. | Systematic | 2018 | <25 | Inequities, Health, Education, Violence and Safety, Resources for Youth, and/or Other. | To describe the state of the youth participatory action research (YPAR) literature and synthesize findings about the youth outcomes reported in these studies. | Youth Participatory Action Research (YPAR) | Youth participatory action research (YPAR) involves young people constructing knowledge by identifying, researching, and addressing social problems through youth-adult partnerships (Cammarota & Fine, 2010; Checkoway & RichardsSchuster, 2003; Jacquez, Vaughn, & Wagner, 2013; Shamrova & Cummings, 2017). | PubMed, ERIC, Social Service Abstracts, and PsychInfo | NR | Eligibility criteria focused on four key elements: (1) study characteristics (empirical studies, published in peer-reviewed journals, conducted in the United States, published in English); (2) population (program participants comprised children or youth 25 years or younger; for the youth of ages 18 to 25 years, samples were excluded if they consisted only of undergraduate or graduate students); (3) intervention (an inquiry-based program that involved youth in data collection, data analysis, and data interpretation); and (4) outcomes (study reports on the experiences, outcomes, or impact of the program for the youth participants or their surrounding environment). | NR |
| Peterson, A., Drake, P., Tat, S., Silver, G., Bell, H., & Guinosso, S. (2020). Youth Engagement in Sexual Health Programs and Services. | Literature | 2020 | NR | SRH | To explore the overall context, current research, needs, and resources related to youth engagement in sexual and reproductive health programs and services. | Youth engagement | Meaningful YE is based on the premise that young people have a right to be heard in matters that affect them. Engaging youth in the design, implementation and evaluation of policies, programs, and systems that impact their lives is believed to support young people’s development, strengthen programs, services, and organizations, and provide social, political and economic benefits. | PubMed and Google | Conducted internet searches, and contacted stakeholders to identify existing YE interventions | Included interventions that: 1) explicitly used YE strategies in the development, delivery, evaluation, or leadership capacities of interventions; and 2) aimed to impact sexual health outcomes. To identify the most relevant strategies, we excluded interventions that: 1) involved youth briefly in focus groups and interviews but not in the co-creation of interventions; 2) involved youth as participants of evaluations but not as agents in the evaluation design, data collection, or analysis process; or 3) used YE strategies but did not explicitly intend to influence sexual behavior or outcomes as a primary or secondary outcome. | NR |
| Larsson, I., Staland-Nyman, C., Svedberg, P., Nygren, J. M., & Carlsson, M. (2018). Children and young people’s participation in developing interventions in health and well-being: a scoping review. *BMC health services research*, *18*(1), 1-20. | Scoping | 2018 | <25 | Health and wellbeing | To systematically map recent research involving children and young people in the development of interventions targeting issues of health and well-being. | Participatory research | Participatory research: The stages of participatory research are; nonparticipation, consultative participation, and collaborative participation. In the stage of nonparticipation, children are either not involved at all or are involved in ways that have no real impact on the research or that give a false semblance of partnership and sharing of power. At the stage of consultative participation, adults acknowledge the expertise of children and involve them in sharing their views and experiences, primarily through interviews or questionnaires. However, such approaches are neither giving the children control over the focus of the research or influence over the analysis or interpretation of data. At the collaborative stage, children are not only involved as experts but also take part at various degrees in initiation, planning, analysis and dissemination of the research. | Academic Search Elite, CINAHL, ERIC, Medline, PsycInfo, Sociological Abstracts and SportDiscus | Reference lists, hand-searches of key journals and contact with existing networks | Inclusion criteria were: articles with children and young people under 25 years old; articles in which children and young people participate in one or more levels of the development of an intervention aimed at children and young people in health and well-being.  Exclusion criteria were: articles in which children and young people were only participants in an intervention or evaluated an intervention. | NR |
| Mandoh, M., Redfern, J., Mihrshahi, S., Cheng, H. L., Phongsavan, P., & Partridge, S. R. (2021). Shifting From Tokenism to Meaningful Adolescent Participation in Research for Obesity Prevention: A Systematic Scoping Review. *Frontiers in public health*, *9*. | Scoping | 2021 | 10 to 24 | Obesity or chronic diseases | To assess how and to what extent adolescents are meaningfully participating in the co-design and decision-making in research studies that target overweight, obesity, physical activity, and dietary interventions specifically for adolescents and to provide recommendations on optimal modes of participation in obesity and chronic disease prevention research. | Meaningful participation | Meaningful participation refers to participation in which adolescents have some level of influence over the research and development decision-making. The mode of meaningful participation is dependent on the degree of influence adolescents impart on the research process | Medline (PubMed), Embase, CINAHL, Scopus, Global health, and CENTRAL | Additional sources were identified through hand searching, reference list examination, and citation chaining | All qualitative and quantitative peer-reviewed primary research studies published from 1995 to December 2020 were considered for inclusion; reviews were excluded. Peer-reviewed papers of all languages with an abstract in English were considered. Studies involved participants aged 10-24 years involved in obesity or chronic disease prevention, nutrition or physical activity research decision-making. Youth participation was the primary outcome sought hence inclusion of broad search terminologies such as Youth-PAR, CBPR, youth involvement, and youth engagement. | NR |
| Martins, F. A. F. S. (2021). Impacts of patient and public involvement in mental health research with young people: a systematic review. | Systematic | 2021 | 11 to 20 | Mental health | To systematically describe the landscape of the understudied area of youth PPI in mental health research | Patient and Public Involvement (PPI) | INVOLVE defines PPI as a process through which members of the public are being actively involved in the research process and in research organisations, so that the research is being carried out with or by them, instead of to, about or for them. | PsycINFO (OVID), MEDLINE (OVID), EMBASE (OVID), web of science core collection, current contents connect, SciELO Citation Index, Cochrane Library of Systematic Reviews, CINAHL (EBSCO), ERIC (EBSCO), and child and adolescent studies (EBSCO). | NR | Eligibility criteria is broad to include qualitative, quantitative and mix-methods studies on mental health research, that encompass PPI with young people from 11 to 20 years of age, reporting on the impacts of PPI. As the age of reference for young people, in this TREATme project, is comprised between 11 and 20 years old, this is the age range considered for the samples of interest in the present review. Controlled and uncontrolled trials, pre-post studies, cross sectional studies, methodological or developmental study, pilot/feasibility trials and reflections from the field are included. Case study/series and studies on mental health prevention are excluded | The critical appraisal mainly focused on assessing the quality and integrity of reporting on the essential elements of PPI. Through a partnership with young people with lived experience of mental health difficulties, existing guidelines for PPI reporting (GRIPP) were reviewed and new guidelines were co-produced, the Reporting Guidelines for PPI in mental health research with young people: Design through delivery (Edridge et al., 2020). |
| Reed, H., Couturiaux, D., Davis, M., Edwards, A., Janes, E., Kim, H. S., ... & Evans, R. (2021). Co-production as an Emerging Methodology for Developing School-Based Health Interventions with Students Aged 11–16: Systematic Review of Intervention Types, Theories and Processes and Thematic Synthesis of Stakeholders’ Experiences. *Prevention Science*, *22*(4), 475-491. | Systematic | 2021 | 11 to 16 | Health research | To understand the types and underlying theories and processes for co-production in school-based health interventions with students | Co-production | Elements of co-produced interventions included in the review  (1) Involvement is conducted within the school context. Secondary schools vary contextually so situating coproduction within individual settings allows stakeholders to generate school-specific interventions responsive to student and staff needs. (2) Interventions are developed based on the views of those who will use them. At a minimum, this must be school students as the recipients of school-based health promotion interventions, omitting interventions where only adults contribute to co-production processes. (3) Stakeholders are involved iteratively in problem-setting and solving | Medline and PsycINFO (Ovid); Embase; ASSIA; and ERIC | Consulting a panel of international experts, citation tracking of included studies and contacting study authors for further papers describing co-production processes and/or assessing stakeholders' experiences. | To be eligible for this review, papers needed to meet the following criteria: (i) population: young people aged 11â€“16, either as a subpopulation or as the whole population; (ii) intervention: fall within the review's remit of intervention co-production, namely be conducted in schools, involve students, and include problem-setting and solving; (iii) setting: secondary school context (or international equivalent); (iv) outcome: violence and aggression, mental health and wellbeing, and/or substance use as a primary outcome, as review scoping indicated that these were the most productive areas of development around co-production; (v) data: a range of studies with co-produced interventions where qualitative data about the processes and stakeholders' experiences were available in study documents; (vi) study: any country; (vii) language: published in English; (viii) date: published between 1986 and the date searches were conducted (February 2018) to coincide with the Ottawa Charter for Health Promotion which foreground stakeholder involvement (WHO 1986). | The EPPI Centre health promotion review criteria |
| Partridge, S. R., & Redfern, J. (2018, September). Strategies to engage adolescents in digital health interventions for obesity prevention and management. In *Healthcare* (Vol. 6, No. 3, p. 70). Multidisciplinary Digital Publishing Institute. | Narrative | 2018 | 10 to 24 | Obesity | To appraise the strategies for effective engagement include co-designing interventions with adolescents, personalization of interventions, and just-in-time adaptation using data from wearable devices | Co-design, personalization, Just-in-time adaptation | Co-design is an umbrella term used to describe the array of approaches that can be utilized to engage the end-users (i.e., those affected by the issue being studied) or other stakeholders in the research process.  Personalization: Definition not provided  Just-in-Time-Adaptation: Just-in-time adaptive interventions are a form of personalized interventions that provide support relevant to an individualâ€™s changing behaviors and contexts over time. | N/A | N/A | N/A | NR |
| Montreuil, M., Bogossian, A., Laberge-Perrault, E., & Racine, E. (2021). A review of approaches, strategies and ethical considerations in participatory research with children. *International Journal of Qualitative Methods*, *20*, 1609406920987962. | Realist review | 2021 | <12 | Overall research | To help researchers make more informed decisions on how to involve children in the research process, as opposed to solely a mean of data collection, and identify ethical issues that may arise. | Participatory research | Participatory research approaches: ... the consideration of children as agents who can contribute to research development, design, conduct, analysis and dissemination (Clavering & McLaughlin, 2010)  Children's engagement within participatory research processes: By the term engagement, we refer notably to Dewey's concept of learning and growing which involves children acting about an idea, broadening their perception through the idea; and valuing it in relation to everyday experiences, from children's perspectives (Aubrey & Riley, 2015) | CINHAL, ERIC, Scopus, ProQuest Social Sciences, PsycInfo, Pubmed, Web of Science, Academic Search Complete (EBSCO) and Anthropology Plus | NR | Inclusion and exclusion criteria were the following: (1) studies using a participatory research approach, in which children were engaged in at least one aspect of the research process (i.e. not a study in which children were solely involved as research subjects for data collection, but in which children were involved, for example, to refine the research question, to collect data, to interpret data or plan dissemination (L. W. Green et al., 1995); (2) children aged 12 years old or younger: (3) all research designs; (3) publication in English or French language; (4) no geographical, date or disciplinary limitations. Articles were excluded if samples included children from a broad range of ages (i.e. with children above 12 years old) for which younger children's specific participation in the research process could not be determined. Articles were also excluded if children were solely involved as research subjects for data collection. | NR |
| Gaillard, S., Malik, S., Preston, J., Escalera, B. N., Dicks, P., Touil, N., ... & Kassaï, B. (2018). Involving children and young people in clinical research through the forum of a European Young Persons’ Advisory Group: needs and challenges. *Fundamental & clinical pharmacology*, *32*(4), 357-362. | Narrative | 2018 | N/A | Health research | To describe the needs for involving children and young people in clinical research, highlighting the methods and benefits of including their perspectives and to describe the challenges researchers can face when designing pediatric research, and how a network such as the European Young Persons' Advisory Group network (eYPAGnet) can help. | Involving children and young people, Youth advisory groups | "Christensen and Prout suggested that there are four ways of considering research about involving young people; these includeyoung people can be viewed as ‘objects’ to be studied ;as ‘subjects’ in their own right; as ‘social actors’ able to act, change, and be changed by their actions; and as active participants in the research process, able to shape, change, and challenge the research process and knowledge development." | N/A | N/A | N/A | NR |
| Dubois, A. C., Lahaye, M., & Aujoulat, I. (2022). From research ‘on’to research ‘with’children about their family lives: a scoping review of ethical and methodological challenges. *Child: Care, Health and Development*, *48*(2), 203-216. | Scoping | 2021 | 5 to 17 | Family lives | To identify more precisely the specific ethical challenges inherent to such research and the recommended methodological strategies documented by the authors to address these challenges throughout the research process | Research with children, participation, lived experience | NR | PubMed,Embase, PsycInfo, ERIC and Scopus | NR | The eligibility criteria were defined as follows: primary research OR reflective papers that draw on pieces of research conducted by their authors, reporting ethical and methodological issues in conducting research that explores children and adolescents' lived experience of some aspects of their family lives. Peer-reviewed journal papers were included if written in English, French, Italian, German or Dutch. No date limits were applied. Papers were excluded if they did not fit into the scope of our study. Opinions, editorials, conference abstracts and reviews were excluded. | NR |
| Worrall‐Davies, A., & Marino‐Francis, F. (2008). Eliciting children's and young people's views of child and adolescent mental health services: a systematic review of best practice. *Child and Adolescent Mental Health*, *13*(1), 9-15. | Systematic | 2008 | <18 | Mental health | This review aims to identify the methods that have been used to obtain children's and young people's views of CAMHS, the best practice methods available for eliciting children's and young people's views of CAMHS; and the methods that were most effective in leading to changes in CAMHS | Eliciting children's and young people's views | Children and young people expressing views of CAMHS | PubMed, Medline, Embase, CINAHL, PsychINFO, PsychARTICLES and FULL TEXT APA, BIDS International Bibliography of Social Sciences, Web of Science, and ERIC | Specialist journals likely to contain relevant articles were hand searched for the period 1993-December 2005. These were: Psychiatric Bulletin, Child and Adolescent Mental Health (previously Child Psychology and Psychiatry Review); and YoungMinds magazine. We requested and obtained conference abstracts (up to and including 2005) from The Royal College of Psychiatrists (Child and Adolescent Faculty) and the British Psychological Society (Faculty of Children and Young People) | All studies written in English in which children and young people up to 18 years of age expressed views of CAMHS were eligible for inclusion. Studies that focused upon children's and young people's views of other health services and of other statutory and voluntary services were excluded. For the purposes of this review we classified CAMHS broadly to include primary care (Tier 1) through to specialist community and hospitalbased services (Tier 2/3) to day, intensive outreach, and in-patient services (Tier 4) (HAS, 1995). Health Advisory Service (1995). Child and adolescent mental health services: Together we stand. London: HMSO. | Quality framework commissioned by the Cabinet Office to guide policy-makers in assessing the quality of qualitative research evaluations (Spencer et al., 2003). |
| Ozer, E. J., Abraczinskas, M., Duarte, C., Mathur, R., Ballard, P. J., Gibbs, L., ... & Afifi, R. (2020). Youth participatory approaches and health equity: Conceptualization and integrative review. *American Journal of community psychology*, *66*(3-4), 267-278. | Integrative | 2020 | NR | Health research | To promote greater clarity in the conceptualization, implementation, and evaluation of youth participatory approaches; frame the landscape of youth participatory approaches and their similarities and differences; present an integrative review of the evidence regarding the impact of youth participatory approaches; and describe several illustrative cases so as to consider more deeply how some youth participatory approaches aim to influence the social determinants of health that lead to the physical embodiment of health inequities. | Youth Participatory Action Research (YPAR), youth organizing, youth-led planning, human-centered design (HCD), Participatory arts,  Youth advisory boards and councils | YPAR, a form of community-based participatory research (CBPR; Wallerstein, Duran, Minkler, & Oetzel, 2017), youth train as researchers to study equity issues they want to influence and advocate for changes based on their findings (e.g., Cammarota & Fine, 2010; Fine, 2008; Kidd, Davidson, Frederick, & Kral, 2018; Levac, Ronis, Cowper-Smith, & Vaccarino, 2019; Mirra, Garcia, & Morrell, 2015; Ozer & Piatt, 2018; Rodr Ä±guez & Brown, 2009). YPAR is an orientation, rather than a method, that challenges dominant assumptions about who holds and creates knowledge. Youth are considered experts who generate valid knowledge about the conditions they seek to change while working to shift power structures and change inequitable systems, policies, and practices (Fine, 2008).   Youth organizing is a rights- and youth development focused form of community organizing in which adolescents activate to claim power and make change for themselves (Christens & Kirshner, 2011).   In youth-led planning, adolescents typically work on a specific planning problem identified by a defined client, such as a government agency (McKoy & Vincent, 2007; Youth-Plan Learn Act Now, 2019); for example, how can public transportation routes or parks best serve youth to promote health and well-being?   Youth who engage in human-centered design (HCD; also design thinking or user-centered design) contribute the user experience perspective to adults design of programs or products; in the health realm, this can take diverse forms such as informing the development of health-related apps for smartphones, or youth-informed design of health-related services intended to serve youth.  Participatory arts refer to adolescents making and sharing various forms of art (e.g., theater, poetry, photography) to express themselves. Often, these methods are used only as an additional aspect of data collection, but without (or with little) youth agentic roles. Here, we use the term emancipatory participatory arts to refer to the use of participatory arts in projects that provide agentic roles for youth to raise the visibility and critical consciousness of health-related issues and promote action.    Youth advisory boards and councils provide sustained opportunities for youth to express ideas and provide their perspectives to guide policies and practices of adult-led organizations. | PsycINFO, PubMed, Google Scholar | NR | We excluded reviews that focused only on adults or on community-based approaches that were not participatory (e.g., collecting focus group data, culturally tailoring interventions). | NR |
| Hawke, L. D., Relihan, J., Miller, J., McCann, E., Rong, J., Darnay, K., ... & Henderson, J. L. (2018). Engaging youth in research planning, design and execution: practical recommendations for researchers. *Health Expectations*, *21*(6), 944-949. | Narrative | 2018 | NR | Mental health | To provide practical recommendations to help researchers engage youth in meaningful ways in academic research, from initial planning to project completion | Youth engagement | Not clearly defined | N/A | N/A | N/A | NR |
| Valdez, E. S., Skobic, I., Valdez, L., O Garcia, D., Korchmaros, J., Stevens, S., ... & Carvajal, S. (2020). Youth participatory action research for youth substance use prevention: a systematic review. *Substance use & misuse*, *55*(2), 314-328. | Systematic | 2020 | NR | Substance abuse | To identify and describe YPAR studies in the context of youth substance use prevention research. | Youth participatory action research (YPAR) | Youth participatory action research (YPAR): YPAR is a form of participatory action research (PAR) that provides youth with the opportunity to study social problems affecting their lives and to determine actions to solve these problems (Cammarota & Fine, 2008). PAR is an approach that engages researchers and participants in collective, self-reflective inquiry so they can understand themselves and the world around them, and improve upon their circumstances (Livingston, 2017). | PsycINFO, PubMed, Web of Science, Scopus, EMBASE and Google Scholar | Reviewed reference lists from all articles that met the inclusion criteria for additional studies for potential review. | Inclusion criteria included peer-reviewed, English-language articles published from January 1, 1998 through April 30, 2018 that referenced YPAR, PAR, CBPR, youth, and substance use prevention. We included articles on YPAR interventions/programs/projects for youth substance use prevention; youth-led PAR or CBPR projects; and studies addressing substance use prevention as a primary or secondary outcome. All included studies described research that was youth-led (versus adult-led or in partnership with adults). Articles inconsistent with the inclusion criteria or which were editorial, historical or theoretical in nature were excluded. We excluded articles on adult-led interventions with youth collaborators and articles or other publications from non-peer reviewed sources. For example, we excluded articles where youth were used to validate or test an intervention if that intervention was not originally conceived by and developed by youth. | The Reliability-Tested Guidelines for Assessing Participatory Research Projects (Mercer et al., as cited in Minkler & Wallerstein, 2011) were adapted to assess youth engagement in each stage of the participatory research process |
| Pavarini, G., Smith, L. M., Shaughnessy, N., Mankee‐Williams, A., Thirumalai, J. K., Russell, N., & Bhui, K. (2021). Ethical issues in participatory arts methods for young people with adverse childhood experiences. *Health Expectations*, *24*(5), 1557-1569. | Narrative | 2021 | NR | Adverse childhood experiences | To examine ethical issues emerging from participatory arts methods with young people with traumatic experiences. | Participatory arts based approaches | Participatory or emancipatory research values a commitment to creating spaces for children and adolescent voices to be fully heard and prioritised in the research process. Rather than taking on a passive role as subjects of research, adolescents are seen as activists and actors who set the agenda for research; coproduce interventions; collect and generate data; analyse results; and disseminate outputs. | N/A | N/A | N/A | NR |
| Willis, J., Zeratkaar, D., Ten Hove, J., Rosenbaum, P., & Ronen, G. M. (2021). Engaging the Voices of Children: A Scoping Review of How Children and Adolescents Are Involved in the Development of Quality-of-Life–Related Measures. *Value in Health*, *24*(4), 556-567. | Scoping | 2021 | <18 | Quality of life | To identify instruments that measure various aspects of health and QoL and to identify, collate, and report qualitative methods used to collect the raw material used to develop the content and items for these instruments. | Engagement | Engaging children and adolescents in qualitative research, from which operational definitions and conceptualization of QoL outcome instruments are developed. Engaging children and adolescents, as experts in their own health and quality of life (QoL), in the development of instruments. | Medline and EMBASE were searched through the Ovid interface | NR | Search results were used to identify unique self-report instruments that were developed with the intent of measuring QoL or HRQoL (either exclusively or as a domain of a larger construct) in children and adolescents (usually under 18 years). We excluded econometric scales, instruments with only proxy respondents, and instruments that were developed for use in adult populations but later validated for use with children or adolescents without explicitly seeking their voices.7 If an instrument was a module, we included only the prototype of the instrument development and did not include any subsequent measures developed using the original methodology. | NR |
| Vaughn, L. M., Wagner, E., & Jacquez, F. (2013). A review of community-based participatory research in child health. *MCN: The American journal of maternal/child nursing*, *38*(1), 48-53. | Literature | 2013 | NR | Health research | To identify published CBPR studies that use a direct collaborative CBPR approach with children and/or youth about health issues, and to highlight the role of children and adolescents in the CBPR process in health-related studies/projects | Community-based Participatory Research (CBPR) | CBPR is a collaborative, partnership approach to research that directly and equitably involves community members in all phases of the research process. | PubMed, Scopus, and Web of Science databases | NR | Inclusion criteria consisted of English-language articles published from 1985 to January 14, 2012, that described CBPR with children and youth about health issues. Only published articles in refereed journals were included. We evaluated articles to ensure that (1) children and adolescents were actively involved in the research process (a fundamental principle of CBPR) unless the topic was maternal/child health and directly focused on the neonates who could not be active research participants, and (2) studies were focused on a health topic and/or setting. We were interested in articles that claimed the terminology of CBPR (or related terms such as participatory action research). We excluded CBPR articles with children/youth that were not health-related (12 articles) or were related to health only in a broad sense focused on topics such as immigration or community engagement (18 articles). We omitted literature reviews and community-placed (i.e., research conducted in a community setting but without a participatory component) articles. We also excluded CBPR studies where the adults were exclusively the active participants rather than the youth even if the topic was related to child health (e.g., teachers and parents engaging in research about childhood obesity). | NR |
| Mathur, S. R., & Rutherford Jr, R. B. (1991). Peer-mediated interventions promoting social skills of children and youth with behavioral disorders. *Education and Treatment of Children*, 227-242. | Literature | 1991 | 3 to 20 | Mental health (Behavioral disorders) | To describe different types of peer mediation, critically analyze the efficacy of peer-mediated approaches that promote social skills of children and youth with behavioral disorders, and critically examine the success of these interventions in terms of promoting generalization. | Peer-mediated interventions | Peer-mediated interventions can be described as those in which peers serve as behavior change agents (Kalfus, 1984; Odom & Strain, 1984; Strain & Odom, 1986). Peer-mediated interventions are based on the premise that children develop social skills through their interactions with peers (McEvoy & Odom, 1987). | ERIC | NR | Articles meeting the following criteria were selected: (a) Participants were students from age 3 through 20 years exhibiting social behavior problems; (b) a peer-mediated intervention was employed either alone or with other procedures; (c) a valid single-subject design or between-groups design was used; and (d) direct measures of overt behavior were employed | NR |
| Cordier, R., Vilaysack, B., Doma, K., Wilkes-Gillan, S., & Speyer, R. (2018). Peer inclusion in interventions for children with ADHD: A systematic review and meta-analysis. *BioMed research international*, *2018*. | Systematic | 2018 | NR | Mental health (ADHD) | To examine the efficacy of peer inclusion in interventions targeting the social functioning of children with ADHD and to capture the use of peers in interventions in the existing literature | Peer involvement, peer mediation, peer proximity | 1. Peer involvement: Peer involvement is most commonly characterised by interventions where participants facilitate each other’s learning. Peers included in these interventions often include children with similar diagnoses and skill difficulty in a group therapy context.  2. Peer-mediated intervention involves a combination of peer initiation, modelling, prompting, and reinforcing of the desired behaviour. Peer-mediated interventions are based on the notion that individuals’€™ behaviour is influenced by their peers, an influence that can be both overt and powerful. 3. Peer proximity involves carefully selected peers of increased skill, likely without a diagnosis, who are placed in close proximity to the child, such as sitting at the same table in a classroom. | CINAHL, PsycINFO, Embase, and Medline | Supplementary search approaches such as checking reference lists were also used to identify studies. | Inclusion/Exclusion Criteria. The following criteria for inclusion were applied: (1) children and/or adolescents had to have a primary diagnosis of ADHD according to the Diagnostic and Statistical Manual of Mental Disorders 3rd Edition (Revised, DSM-III-R) or Diagnostic and Statistical Manual of Mental Disorders 4th Edition (DSM-IV) criteria; (2) studies included a control group; (3) the interventions included peers; (4) the treatment content focused on social functioning; and (5) the treatment outcome could be related to the peer inclusion intervention. Multimodal intervention programs in which the peer inclusion intervention was part of a variety of empirically based behavioural components were included if results can be extrapolated to provide insight into the value of including peers as a core variable. These criteria were selected to identify peer inclusion intervention studies that would be classed as either level II or III on the National Health and Medical Research Council (NHMRC) Hierarchy of Evidence. The NHMRC Hierarchy of Evidence was developed by the Australian NHMRC to rank and evaluate the evidence of healthcare interventions. According to the NHMRC Hierarchy of Evidence, level I studies are systematic reviews of randomized controlled trials (RCTs), level II studies are a well-designed RCTs, and level III studies are, for example, quasi-experimental designs without random allocation. Studies with level III evidence were included as it was unlikely that a search limited only to level II studies would identify all required studies to review the literature. | NHMRC Evidence Hierarchy evels of evidence and the Kmet appraisal checklist |
| Vujcich, D., Thomas, J., Crawford, K., & Ward, J. (2018). Indigenous youth peer-led health promotion in Canada, New Zealand, Australia, and the United States: a systematic review of the approaches, study designs, and effectiveness. *Frontiers in Public Health*, *6*, 31. | Systematic | 2018 | 13 to 29 | Health research | To address the gap in the literature by systematically reviewing studies of Indigenous peer-led health promotion programs in Australia, Canada, New Zealand, and the United States of America. | Peer-led health promotion | Peer-led health promotion is defined as the teaching or sharing of health information, values and behaviors by members of similar age or status groups. | Medline, EMBASE, and ProQuest Social Sciences Database | To capture gray literature and publications not contained in electronic databases, supplementary searches were conducted. Google [terms: peer-education AND (young or youth) AND (Indigenous OR Aboriginal) AND health] and Australian Indigenous HealthInfoNet HealthBulletin (terms: peer OR youth OR young) were searched (no comparable Indigenous databases in New Zealand or North America were identified). Only the first 10 pages of results were manually scanned for relevance. Reference lists of included studies were also scanned for relevant literature. | To be included in the systematic review, studies needed to relate to a health promotion intervention that was both aimed at, and delivered by, young people aged 13-29 years who were Indigenous to New Zealand, Australia, Canada, or the United States of America. This systematic review was designed to include both qualitative and quantitative study designs to ensure that both stakeholdersâ€™ perceptions/experiences and numerical indicators of effectiveness were captured. Exclusion criteria Exclude publications that: -are duplicates -merely describe an intervention without results (e.g., study protocols, program descriptions) -do not contain a detailed description of study design and/or findings (e.g., conference posters) - are published in a language other than English. | Critical Appraisal Skills Program Checklists |
| Kew, K. M., Carr, R., & Crossingham, I. (2017). Lay‐led and peer support interventions for adolescents with asthma. *Cochrane Database of Systematic Reviews*, (4). | Systematic | 2017 | 10 to 19 | Asthma | To assess the safety and efficacy of lay-led and peer support interventions for adolescents with asthma. | Pee-led interventions | Peer-led interventions: Studies that assess an intervention delivered by peers or by lay people to adolescents with asthma. We defined peers as people who are not medically trained but are similar to the target population in terms of age, presence of an asthma diagnosis or diagnosis of a different long-term condition. These interventions may also be considered lay led, but other eligible interventions that meet the criteria for a lay-led intervention may not be considered to include peer support (e.g. those delivered by adult community health workers). | Cochrane Airways Group Specialised Register (CAGR), Cochrane Central Register of Controlled Trials (CENTRAL), MEDLINE Ovid, Embase Ovid, PsycINFO Ovid, Cumulative Index to Nursing and Allied Health Literature (CINAHL) EBSCO, Allied and Complementary Medicine Database (AMED) EBSCO, Handsearches of the proceedings of major respiratory conferences, ClinicalTrials.gov (www.ClinicalTrials.gov), the WHO International Clinical Trials Registry Platform (ICTRP; www.who.int/ictrp/en/). | Checked the reference lists of all primary studies and review articles for additional references.  Conference proceedings searched  American Academy of Allergy, Asthma and Immunology (AAAAI) American Thoracic Society (ATS) Asia Pacific Society of Respirology (APSR) British Thoracic Society Winter Meeting (BTS) Chest Meeting European Respiratory Society (ERS) International Primary Care Respiratory Group Congress (IPCRG) Thoracic Society of Australia and New Zealand (TSANZ) | Criteria for considering studies for this review Types of studies: We included parallel randomised controlled trials (RCTs). We included studies that used individual or cluster randomisation, but we excluded cross-over studies owing to the likelihood of carry-over effects. We included studies reported as full text, those published as abstract only and unpublished data. Types of participants: We included adolescents with a diagnosis of asthma. We included studies that described inclusion criteria for asthma, such as confirmation by a physician or via spirometry, to exclude people with wheeze not associated with obstructive airways disease. For the purposes of this review, we defined adolescents as those between 10 and 19 years of age, in keeping with the definition of the World Health Organization (WHO 2016). If a study had an unclear age range, included a subset of the age group of interest (e.g. younger adolescents between 10 and 14 years of age) or included participants outside our predefined age criteria (e.g. university students 18 to 21 years of age), we included the study if the mean age of participants was between 10 and 19 years. We excluded studies that enrolled adolescents with other long-term conditions, such as cystic fibrosis, unless the study authors presented results for participants with asthma separately. Types of interventions: We included studies that assessed an intervention delivered by peers or by lay people to adolescents with asthma. We defined peers as people who are not medically trained but are similar to the target population in terms of age, presence of an asthma diagnosis or diagnosis of a different long-term condition. These interventions may also be considered lay led, but other eligible interventions that meet the criteria for a lay-led intervention may not be considered to include peer support (e.g. those delivered by adult community health workers). We undertook metaâ€analyses only when interventions were similar enough for pooling to make sense, and we presented intervention characteristics in a summary table in the review. We explored differences in the characteristics of those who deliver the interventions, when possible, using subgroup analysis. We analysed studies that compared the intervention versus usual care or a minimal control intervention separately from those that compared the intervention against another active intervention. We excluded studies that used basic peer support itself as a minimal control for a more intensive intervention. We included interventions delivered to individuals or groups of adolescents with asthma, irrespective of the mode of delivery (face-to- face or via technology). We excluded studies of interventions that involved multiple components other than the peer support or layâ€led intervention unless the control group also received them.  We included studies regardless of the aim of the intervention (e.g. improving self-esteem, improving medication adherence, providing asthma education). | The criteria outlined in the Cochrane Handbook for Systematic Reviews of Interventions |
| Watkins, L., O’Reilly, M., Kuhn, M., Gevarter, C., Lancioni, G. E., Sigafoos, J., & Lang, R. (2015). A review of peer-mediated social interaction interventions for students with autism in inclusive settings. *Journal of autism and developmental disorders*, *45*(4), 1070-1083. | Literature | 2015 | NR | Autism | To identify and describe the characteristics and components of PMI used to facilitate social interaction skills of students with ASD in inclusive settings, evaluate the effectiveness of PMI in improving social interaction by offering an analysis of intervention results and research design, and suggest recommendations for future research. | Peer-mediated interventions (PMI) | Peer-mediated interventions (PMI): Peer-mediated intervention strategy was subdivided into three different types of PMI as identified by Odom and Strain (1984): proximity, prompting and reinforcing, and peer initiation. Proximity refers to interventions in which socially competent peers are placed in close proximity to participants in order to model appropriate social interactions but are not directly trained in strategies to increase interaction with participants. Prompting and reinforcing refers to interventions in which peers are explicitly taught to prompt and/or reinforce a specific response or behavior from participants. Peer initiation refers to interventions in which peers are specifically taught to initiate interaction with participants. | PsycINFO, Education Resources Information Center (ERIC) | In order to identify relevant studies potentially missed by electronic search, ancestry searches through the reference lists of studies meeting inclusion criteria were conducted. | Articles were included in this review if a PMI was used to improve the social interaction skills of an individual diagnosed with autism, autism spectrum disorder, Asperger’s syndrome, or pervasive developmental disorder-not otherwise specified (PDD-NOS). If studies incorporated participants with other diagnoses, only the data for participants with ASD were considered (Hughes et al. 2011). Additionally, only interventions that took place in inclusive settings in which participants with ASD shared the context and activities with typically developing peers were included. As inclusion refers to the placement of special education students in general education settings (Camargo et al. 2014; Mesibov and Shea 1996), studies that took place in a self-contained special education class were excluded (e.g., Banda and Hart 2010; Kuhn et al. 2008). Studies in which typical peers served as the intervention agents but that took place outside of the usual context such as in a secluded classroom or therapy room were also excluded (e.g., Ganz et al. 2012; Krebs et al. 2010). Furthermore, at least one dependent variable needed to include a direct measure of social interaction involving an initiation and/or response between a participant with ASD and at least one peer without ASD. An initiation included verbal or nonverbal behaviors produced by a participant and directed toward a peer for the purpose of (a) beginning or maintaining a conversation, (b) beginning a joint activity or, (c) conversing during an ongoing joint activity (operational definition of initiating was adapted from the definition used by Fey 1986; Hughes et al. 2000). A response included verbal or nonverbal behaviors produced by a participant for the purpose of (a) answering an initiation made by a peer, (b) maintaining a joint activity with a peer, (c) demonstrating understanding of an initiation made by a peer (c.f., Fey 1986; Hughes et al. 2000). Finally, the study must have used an experimental research design that allowed for direct analysis of the effect of the intervention on participant behavior (i.e., single case design or group comparison design). Studies that did not utilize an experimental design were excluded. A total of 14 articles, all of which utilized single case research designs, met these criteria and are included in this review. | Evaluative Method for Determining Evidence-Based Practices in Autism |
| Siddiqui, M., Kataria, I., Watson, K., & Chandra-Mouli, V. (2020). A systematic review of the evidence on peer education programmes for promoting the sexual and reproductive health of young people in India. *Sexual and reproductive health matters*, *28*(1), 1741494. | Systematic | 2020 | 10 to 24 | SRH | To understand the inputs, processes, outputs, and outcomes of youth peer education interventions undertaken in the Indian context and to gain insights on their effectiveness on the above-mentioned outcomes | Peer education | Peer education is a strategy whereby individuals from a target group provide information, training, or resources to their peers. These groups can be determined by social or demographic characteristics (e.g. age, education, type of work) or by risk-taking behaviour (e.g. injection drug use, commercial sex work). Peer networks can increase the credibility and effectiveness of the message being presented as they convey information to often hard-to-reach populations. Peer education is widely used and is generally a low-cost intervention. It is a good approach for conveying information in natural settings where target groups are located (e.g. schools, work sites, social gathering places such as parks or clubs), when group members are unlikely to receive services without such an approach, or when a peer is much more likely to appear credible than a non-group member (e.g. among stigmatised groups). | PubMed and POPLINE databases | The bibliographies of select articles were then reviewed to identify further publications. The list of relevant organisations to target with outreach was developed in consultation with the expert advisory group and included both youth- and adult-led national and international NGOs and donor agencies. | Inclusion criteria The research team included publications only if they adhered to all of the following criteria: were studies and evaluations of interventions that took place in India; were published on or after 1 January 2000 and on or before 31 December 2016; the research was initiated on or after 1 January 2000; the intervention included a stand-alone or integrated peer education component focusing on the promotion of young people's SRH; the target group was young people aged 10-24; measurements on changes in knowledge, attitudes, and/or behaviours were reported; were published in English.  Exclusion criteria The research team excluded publications that were: secondary analyses of existing data sets for the purpose of presenting integrative outcomes from different research studies or programmes; discussions of literature included in contributions to theory building or critique; summaries of the literature for the purpose of information or commentary; editorial discussions that argue the case for a field of research or course of actions | NR |
| Milburn, K. (1995). A critical review of peer education with young people with special reference to sexual health. *Health education research*, *10*(4), 407-420. | Critical review | 1995 | NR | SRH | To highlight the potential of the ideal of peer education whilst paying critical attention to inherently problematical issues. | Peer health education | Peer health education: Peer health education is the teaching or sharing of health information, values and behaviours by members of similar age or status groups. | N/A | N/A | N/A | NR |
| Christensen, J. H., Elsborg, P., Melby, P. S., Nielsen, G., & Bentsen, P. (2021). A scoping review of peer-led physical activity interventions involving young people: theoretical approaches, intervention rationales, and effects. *Youth & Society*, *53*(5), 811-840. | Scoping | 2021 | 10 to 24 | Physical activity | To assess and synthesize the elements and conditions that contribute to the effectiveness of youth-led PA interventions. | Peer-to-Peer Health Promotion, peer education | Peer-to-Peer Health Promotion Interventions: We use the term peer-to-peer intervention as an umbrella term, in line with Shiner's (1999) definition of peer education as an umbrella term used to describe a range of interventions where the educators and the educated are seen to share something that creates an affinity between them. The relationship between participants and peer leaders should constitute genuine peerness, characterized by being tied to shared identity, for example, based on similarity in roles, group categories, or experiences (Shiner, 1999). Similar age alone does thus not constitute a peer relationship, if other characteristics significantly distort the peerness. | Embase, PubMed, Scopus, SPORTDiscus, and Web of Science | Reference lists of included papers were checked, providing nine additional relevant papers. | Included studies (a) reported on a peer-to-peer intervention, (b) involved young people in the role as peer leaders, and (c) included a peer-led, PA-related intervention component. Only peer-reviewed papers in English published between 1997 and 2017 were included. Studies were not excluded based on their quality; however, study quality is considered in the 'Results' section. | NR |
| Zhong, C. S., & Melendez-Torres, G. J. (2017). The effect of peer-led self-management education programmes for adolescents with asthma: A systematic review and meta-analysis. *Health Education Journal*, *76*(6), 676-694. | Systematic | 2017 | 10 to 19 | Asthma | To systematically review the effectiveness of peer-led self-management education programmes for adolescents with asthma on health status, health behaviours and harms, as compared to adult-led programmes or no intervention and to examine whether the interventio's effectiveness depends on the age, gender and characteristics of the peer leader (e.g. if the peer leader has asthma); intervention components (e.g. action plans, monitoring diaries, etc.); and intervention characteristics (e.g. generic vs disease-specific, stand-alone vs combined). | Peer-led | Peer-led self-management programmes: Peers had to undergo training, but did not necessarily have to have asthma, as long as the participant could relate to the educator, which could be through age (Bandura, 1986). | CINAHL, CENTRAL, EMBASE, MEDLINE and PsycINFO | Reference sections of related articles, journals and websites were reviewed to enhance comprehensiveness | For inclusion in this systematic review, studies had to (1) be a RCT, as defined as participants or groups randomly assigned to intervention or control; (2) include 10- to 19-year-olds with asthma; (3) use a structured, peer-led self-management programme that addressed self-care for asthma; and (4) report a measure of health status or behaviour as an outcome. | Cochrane Collaboration's risk of bias tool |
| McHale, F., Ng, K., Taylor, S., Bengoechea, E., Norton, C., O’Shea, D., & Woods, C. (2021). A Systematic Literature Review of Peer-led Strategies for Promoting Physical Activity Levels of Adolescents. *Health Education & Behavior*, 10901981211044988. | Systematic | 2021 | 12 to 19 | Physical activity | To identify peer-led programs that showed promise in improving PA levels of 12- to 19-year-old participants and/or peer leaders and to determine peer-leadership training factors and identify the most promising BCTs employed by peer-led interventions reporting on improved PA outcomes. | Peer-leadership, peer-mentorship, peer-tutoring, peer-delivered, and peer-assisted learning (PAL) | These terms describe any learning process whereby adolescents learn from and with others of similar ages, similar experiences, or those who are older but within the same environment (Colvin, 2007; Jenkinson et al., 2013). For this review, the term peer-led will be all encompassing to describe interacting with and motivating peers to initiate, continue, and sustain positive behavior (Barr-Anderson et al., 2012; Campbell et al., 2008). | PubMed, PsychINFO, and Scopus | Reviewing bibliographies from included articles to identify additional relevant studies. | Original studies, peer reviewed and in English language were included. Studies were eligible if all of the following criteria were fulfilled: (1) they reported on the delivery of a PA intervention that was either peer-led or had a peer-led component, (2) they reported on interventions in which both the peer leaders and participants were aged 12 to 19 years, (3) the comparison group were adolescents not exposed to a peer-led intervention, (4) PA was an outcome measure of the study, and (5) PA outcomes were measured by means of self-report or by a device-based measure. | Study quality was assessed using a 10-item scale used in previous work, further adapted for the needs of this study (van Sluijs et al., 2007) |
| MacArthur, G. J., Harrison, S., Caldwell, D. M., Hickman, M., & Campbell, R. (2016). Peer‐led interventions to prevent tobacco, alcohol and/or drug use among young people aged 11–21 years: a systematic review and meta‐analysis. *Addiction*, *111*(3), 391-407. | Systematic | 2016 | 11 to 21 | Substance abuse | To identify and review the effects of peer-led interventions that aim to prevent tobacco, alcohol and/or illicit drug use among young people aged 11-21 years. | Peer-led | To be classed as a peer-led intervention, programmes needed to include a substantial component in which peers were involved in the delivery of the intervention; for instance, via the direct delivery of curriculum components, or by acting as a mentor or "buddy" to study participants. | Medline, Embase, PsycINFO (all via OvidSP), CINAHL (via EBSCOhost), ERIC (via ProQuest), the Australian Education Index, British Education Index and the Cochrane Library (via Wiley Online Library) | Based on the authors' own knowledge and advice from colleagues in the field we searched for the most relevant grey literature by prioritizing and checking websites of organizations renowned in the field, such as the Joseph Rowntree Foundation, World Health Organization, Evidence for Policy and Practice Information and Co-ordinating Centre (Eppi Centre) and National Youth Agency. | Studies were eligible if they targeted tobacco, alcohol and/or drug use and included young people aged 11-21, or if more than half the participants were aged within this bracket. Comparators could be usual practice, no intervention or teacher, adult or professional-led intervention. Exclusion criteria included the following: interventions targeting young people with a clinical disorder; those that targeted young people and another population group such as parents; multi-component interventions; brief interventions, clinical interventions; studies with less than 6 weeks of follow-up; studies that targeted prescription drug or body-enhancing drugs; and study designs other than RCTs, including pilot RCTs and feasibility studies. There was no limit on the setting of the intervention. | The Cochrane tool |
| King, T., & Fazel, M. (2021). Examining the mental health outcomes of school-based peer-led interventions on young people: A scoping review of range and a systematic review of effectiveness. *PloS one*, *16*(4), e0249553. | Scoping | 2021 | 4 to 18 | Mental health | To conduct a scoping review of the range of peer-led interventions used to address mental health outcomes in schools, conduct a systematic review to collate and evaluate the data on the effectiveness of school-based peer-led interventions on mental health outcomes and map the range of mental health outcomes that have been identified. | Peer-led, peer mentoring, peer buddying, peer counselling and peer education | Peer-led interventions take a variety of forms and names, such as peer mentoring, peer buddying, peer counselling and peer education; in this paper we will use the term "peer-led" to include all of these activities, and "peer leaders" or "peer recipients" (whether giving or receiving an intervention, respectively) to describe participants in these interventions. Peer-led interventions typically involve the selection, training and supervision of a group of pupils in preparation for a supportive or educational role among similar-aged pupils in their school. | PsycINFO; PubMed; EMBASE; CINAHL; CENTRAL; BEI; Scopus; Web of Science; ERIC; Social Sciences Citation Index (SSCI); and Social Care Index. | Forward and backward referencing was performed on all included and any relevant studies. A range of grey literature sources were searched, including conference proceedings, dissertations, and government documents. | Scoping review Inclusion criteria The scoping review included peer-led interventions targeting mental health or wellbeing outcomes. The interventions must have taken place within a primary or secondary school and have been predominantly led by students within that school. As definitions of 'mental health' and 'wellbeing' can vary, the studies included were those that had identifiable mental health outcomes. Any programmes with an online element were included as long as they were peerled and based within the school. There were no restrictions based on research design or quality for the scoping review  Systematic review Inclusion criteria The systematic review included randomised controlled trials (RCTs), observational studies, quasi-experimental studies and studies with a pre- and post-test design. All eligible studies had to include at least one mental health or wellbeing outcome (either observational or self-report). The intervention under evaluation must have been at least partly peer-led; therefore, programs jointly led by a peer and an adult were eligible. Studies set in a primary, secondary or special education school, or further education institution for those under 18 years old, were included. School interventions that had an online delivery element were included only if a peer leader was involved. The format of the intervention could be either one-to-one or group-based, as long as any groups were at least partly peer-led. Any studies where an adult facilitated peer-to peer contact, such as a teacher leading a discussion group, were not included. We included studies that looked at either leader or recipient outcomes, or both. Studies were eligible even if they evaluated only the training component for a peer-led programme. Within our protocol, we specified that all peers had to be of school age (4-18 years old) and a current student within the intervention school. However, we expanded the age range to include slightly older students if a study was based in a country or culture where it was not uncommon to be at school beyond 18 years. Both quantitative and qualitative studies were included. Studies including young people with or without a diagnosis of any psychological, emotional or behavioural conditions were eligible, so long as they attended school. We included studies with a minimum sample size of 50 peer pairs in the intervention group, or 50 peer leaders or recipients if only one group was reported. | The Cochrane Risk of Bias Assessment Tool for randomised controlled trials, the Joanna Briggs Institute (JBI) Critical Appraisal Checklist for quasi-experimental studies, and the NIH Quality Assessment Tool for studies with a pre-post design. |
| Kazemi, S., Parvizy, S., Atlasi, R., & Baradaran, H. R. (2016). Evaluating the effectiveness of peer-based intervention in managing type I diabetes mellitus among children and adolescents: A systematic review. *Medical Journal of the Islamic Republic of Iran*, *30*, 442. | Systematic | 2016 | <20 | Diabetes | To identify the effectiveness of peer-based clinical trial interventions in managing T1DM among children and adolescents. | Peer based interventions | Peer-based clinical trial interventions in managing T1DM among children and adolescents (Therefore, considering the age of the suffering individual, his/her peers are considered a good resource of support. Increasing the participation rate of peers and friends leads to raising self-confidence and social acceptance in children and adolescents and finally results in sticking to prescribed regimen) | CINAHL, Cochrane, Pubmed, Google scholar, science direct, web of science and Scopus | Any related websites along with the list of references for all papers. | Three types of studies were included: Randomized Control Trial (RCT), Non-randomized Control Trial (CT), and pre- post.  Inclusion Criteria 1- All English papers, which had been done through peer-based intervention studies on managing T1DM, suffering children and adolescents, with/without comparison groups 2- A maximum age of 20 years for individuals at the time of conducting the study 3- Measuring at least one outcome related to the disease, using a reliable instrument | Cochrane Checklist |
| Ghasemi, V., Simbar, M., Rashidi Fakari, F., & Kiani, Z. (2019). The effect of peer education on health promotion of Iranian adolescents: A systematic review. *International Journal of Pediatrics*, *7*(3), 9139-9157. | Systematic | 2019 | 10 to 19 | Health research | To systematically review the effect of peer education on health promotion of Iranian adolescents. | Peer education | Not clearly defined | Web of Science, Scopus, Embase, Cochrane library and Medline (via PubMed) databases, SID, Magiran and Irondoc as Iranian databases | NR | The inclusion criteria included: the study was perform in Iran, in Persian or English language, from January 2000 to October 2018, interventional design, and in adolescents (10-19 years old). The exclusion criteria include lack of access to the full text of the article, unrelated results, repeated studies, review studies, and target group other than adolescents. | The Effective Public Health Practice Project (EPHPP) tool |
| Cole, C. L. (2015). Peer-mediated intervention for social communication difficulties in adolescents with autism: Literature review and research recommendations. *International Journal of Psychological and Behavioral Sciences*, *9*(4), 347-350. | Critical review | 2015 | 13 to 21 | Autism | To provide a critical review of published research evaluating the use of PMI to improve social-communication skills of adolescents with ASD. | Peer-mediated intervention (PMI) | Peer-mediated intervention (PMI). PMI involves providing information, training, and/or support to typically-developing peers in an effort to facilitate social interactions with children with ASD. | ERIC and PsychINFO | NR | All studies reviewed met the following inclusion criteria: (a) evaluated a PMI strategy; (b) dependent variables included observed social interactions between adolescents with ASD and typical peers; (c) involved adolescents, ages 13-21; (d) involved at least some students with a diagnosis of ASD; (e) employed a single-subject designs with at least three baseline and intervention data points via detailed graphs; and (f) published in peer-reviewed English language journals between 1984 and 2015. | NR |
| Rose-Clarke, K., Bentley, A., Marston, C., & Prost, A. (2019). Peer-facilitated community-based interventions for adolescent health in low-and middle-income countries: a systematic review. *PLoS One*, *14*(1), e0210468. | Systematic | 2019 | 10 to 24 | Health research (key areas of adolescent health defined by the Lancet Commission on Adolescent Health and Wellbeing: infectious and vaccine preventable diseases, undernutrition, HIV and AIDS, sexual and reproductive health, unintentional injuries, violence, physical disorders, mental disorders and substance use) | To systematically review the community-based peer-facilitated interventions in LMICs for the key areas of adolescent health defined by the Lancet Commission on Adolescent Health and Wellbeing: infectious and vaccine preventable diseases, undernutrition, HIV and AIDS, sexual and reproductive health, unintentional injuries, violence, physical disorders, mental disorders and substance use | Peer education, counselling, activisim, outreach | Peer education where peers sought to increase adolescents' knowledge or influence their attitudes, "counselling", defined as peers providing support to help adolescents resolve personal or psychological problems, "activism" involving peer-led campaigns to change health-related policy, and "outreach" with peers engaging marginalised adolescents. | Medline, Embase, Cochrane Library, CINAHL, African Index Medicus, Web of Science, Psycinfo and ERIC | Identified ongoing studies by contacting adolescent health experts. We found further studies by searching relevant reviews. | We only included randomised controlled trials (RCTs) because these studies have a lower risk of bias compared to quasi-experimental studies. We included trials in which the majority (>50%) of participants were adolescents or participants with a mean or median age of 10-19. Trials had to be located in the community (e.g. schools, youth clubs or primary health care centres) because this is where peer-facilitated interventions are commonly located. Trials also had to take place in LMICs (as defined by the World Bank ), and test an intervention delivered in whole or part by peer facilitators, defined here as persons or a majority of persons (>50%) with a mean or median age of 10-24 recruited from the group or community meant to benefit from interventions. We included trials of interventions involving peer education where peers sought to increase adolescents' knowledge or influence their attitudes, counselling, defined as peers providing support to help adolescents resolve personal or psychological problems, activism involving peer-led campaigns to change health-related policy, and outreach with peers engaging marginalised adolescents. We included trials with primary or secondary outcomes relevant to areas of health need outlined in the report of the Lancet Commission on Adolescent Health and Wellbeing: infectious and vaccine preventable diseases, undernutrition, HIV and AIDS, sexual and reproductive health, unintentional injuries, violence, physical disorders, mental disorders and substance use. We deliberately included interventions from across multiple adolescent health areas in order to compare effects across areas. For each area of health need, we included studies with outcomes related to the diseases and risk factors highlighted by the Lancet Commission Report, as well as diseases constituting the 10 main global causes of death or years lived with disability for 10-19 year olds. We also included educational and employment marginalisation, which were considered key determinants of adolescent health. We did not include studies that were conducted in underprivileged populations in high-income countries. No date or language restrictions were applied. | The Cochrane Collaboration's Risk of Bias Tool |
| Choi, S. H. J. (2007). Peer training methods for children and adolescents with autism: A review. *International Journal of Pedagogies and Learning*, *3*(3), 92-100. | Literature | 2007 | NR | Autism | To review how the training of peers is undertaken in peer-mediated interventions for children and adolescents with autism and to provide some practical notions about designing peer involvement for inclusion. | Peer-mediated intervention (PMI) | Peer-mediated interventions for children and adolescents. Common elements of training include peer modelling, peer initiation, peer response, and multiple interaction training for peers. | ERIC, PsycINFO, ProQuest, and the Sage full-text psychology collection | NR | Only papers reporting subjects as having autism were included; thus both low- and high-functioning autism are included, but Asperger syndrome is excluded, as are more general descriptions of disability | NR |
| Aldabas, R. (2020). Effectiveness of peer-mediated interventions (PMIs) on children with autism spectrum disorder (ASD): a systematic review. *Early Child Development and Care*, *190*(10), 1586-1603. | Systematic | 2020 | NR | Autism | To identify the age range of study participants, to identify skills measured and to examine if PMI is effective at improving social skills and communication skills of children diagnosed with ASD | Peer-mediated intervention (PMI) | Not clearly defined | EBSCO, Google Scholar, Educational Resource Information Center (ERIC), ProQuest and Medline | Reference lists of relevant articles were reviewed. | Eligibility criteria Types of studies: Only studies that: used a single-subject design, were published in a peer reviewed journal in English between 1970 and 2017 and were conducted in a school, clinic or home setting.  Types of participants: Only studies where participants were children with ASD were eligible for inclusion.  Types of intervention: Only studies that documented the effectiveness of a variant of PMI on children formally diagnosed with autism.  Types of outcome measures: Only studies that described, measured and reported changes in social skills of children with autism. | NR |
| Villa-Torres, L., & Svanemyr, J. (2015). Ensuring youth's right to participation and promotion of youth leadership in the development of sexual and reproductive health policies and programs. *Journal of Adolescent Health*, *56*(1), S51-S57. | Critical review | 2015 | 10 to 24 | SRH | To present a critical review of the research that has attempted to evaluate the implementation and impact of youth participation in the field of SRHR and describe some approaches to move forward on the adoption of youth participation as an essential practice in policy and program development. | Peer education | Peer education is defined as a form of teaching and/or sharing information, values and behaviors by members of similar age and/or status groups. It is a way in which a small group of peer representatives from a specific group or population actively attempt to inform and influence the rest of the group. | PubMed, Education Resources Information Center, Cumulative Index to Nursing and Allied Health Literature, Family and Society Studies Worldwide, Global Health, and Sociological Abstracts | Additional publications on youth participation were identified from reports and gray literature, with searches done through Google, as well as through searches at Internet sites for youth-led or youth-focused organizations; adult-led nongovernmental organizations and programs working on SRH and/or youth; United Nations (UN) official reports; and youth SRH forum minutes and official statements. | NR | NR |
| Sun, W. H., Miu, H. Y. H., Wong, C. K. H., Tucker, J. D., & Wong, W. C. W. (2018). Assessing participation and effectiveness of the peer-led approach in youth sexual health education: systematic review and meta-analysis in more developed countries. *The Journal of Sex Research*, *55*(1), 31-44. | Systematic | 2018 | <24 | SRH | To synthesize the results of peer-led sexual health education in MDCs through meta-analysis, as well as narrative synthesis, and describes the extent of youth involvement. | Peer participation | Peer Participation. The extent of peer participation was examined based on needs assessment, design, recruitment and selection, training, supervision and monitoring, and follow-up and debriefing. The level of peer participation was evaluated by the flower model, developed from Hart's ladder of participation (Hart, 1992). Each program was first ranked from one to eight according to Hart's ladder of participation, based on the peers' level of responsibility, as described in the study. In the flower model, the first three levels were grouped as 'no responsibility', the fourth and fifth levels as 'low', the sixth as 'medium' and the top two levels were considered 'high'. No responsibility was regarded as tokenism, whereby peers were used only as 'decoration' without reinforcing active participation; the involvement of peers was only a gesture. When peers are passively assigned a particular role designed by adults, it is categorized as low responsibility. Equal responsibility among adults and peers is regarded as medium responsibility. For high responsibility, arrangements are designed for peer empowerment and adult' “youth partnership. | PubMed, ERIC, PsycINFO, Sociological Abstracts, and Cochrane | To further capture relevant articles, reference lists and related journals were examined. The journal Sex Education: Sexuality, Society, and Learning was manually searched. | Studies were included if they (a) examined interventions that promoted adolescent sexual health using peer educators, (b) conducted preintervention and postintervention tests with quantitative outcome evaluations, and (c) reported details of peer participation. For this review, youth is defined as those aged under 24 years (United Nations Department of Economic and Social Affairs, n. d.). Although a randomized controlled trial (RCT) is the gold standard for study design, this review also included nonrandomized controlled trials (non-RCTs) as they could be more aligned with the flexibility and adaptability that are often seen in peer-led programs (Guse et al., 2012). Studies on sex education that specifically focused on a functional group, such as people with a disability or special learning needs, sex workers, sex offenders, or people living with HIV or AIDs (PLWHA), rather than adolescents as a whole, were excluded. | The Quality Assessment Tool for Quantitative Studies, developed by the Effective Public Health Practice Project (Canada) |
| Shamrova, D. P., & Cummings, C. E. (2017). Participatory action research (PAR) with children and youth: An integrative review of methodology and PAR outcomes for participants, organizations, and communities. *Children and Youth Services Review*, *81*, 400-412. | Integrative | 2017 | <18 | Overall research | To support critical conversation about PAR, especially in regard to its methodological features and outcomes of genuine children's participation in PAR for children and youth themselves, social service organizations, and communities. | Participatory research, Participatory Action Research (PAR) | Participatory research: Participatory research with children and youth has drawn a significant amount of attention, in part due to the intensive push in the implementation of the UNCRC (Jones, 2004). It is a transformational perspective which is a part of a continuum of participatory research approaches in which power is redistributed between researchers and participants. In addition, children and youth as participants are given an opportunity to be a part of social change agenda (Nolas, 2011, Pascal and Bertram, 2009). PAR is not a research method by itself, rather it is a post-constructivist epistemological orientation that highlights the importance of subjective experiences in knowledge construction (Minkler & Wallerstein, 2008). Thus, a variety of different methodologies can be utilized under a PAR approach, both widely used methods (e.g. surveys and focus groups) as well as more creative approaches (e.g. Photovoice and drama techniques) (Clark, 2010, Langhout and Thomas, 2010). | ProQuest, EBSCO, Jstor, Project Muse, PubMed, Scopus and Web of Science. | NR | These preliminary exclusion criteria are: (1) Articles that focus entirely on theory building were excluded. (2) Only empirical English-language peer-reviewed articles were included. (3) Articles discussing studies in which only adults participated were removed. (4) Articles in which majority of the participants, even if defined as youth or young adults were over 18 years of age were excluded. (5) The focus of this review is on the impact of PAR on social issues. Thus, the papers that were outside of this area (e.g. marketing or specialized medical treatment). (6) Articles published prior to January 2000 were excluded. Articles published after January 2016 are not included due to the timing of the search.  The two evaluation inclusion criteria utilized were: (1) Papers selected to be included in the review incorporate discussion of the outcomes of children and youth's participation in research (not the outcome of the research or the answer to the research questions) for children themselves, organizations, and/or communities. Authors looked at the evidence of children's or adults' report on what changes PAR made on these three levels. If children were involved in PAR research testing new teaching methods and the paper did not include the discussion on how it affected students, schools or communities, those articles were excluded as well. In addition, the attention was paid to the depth of children's involvement.  (2) Articles that limit children's participation to only being a source of data were excluded due to the lack of participation component and therefore, less likelihood to have and describe social change and impact occurring as a result of the study. The most prominent example of those papers would be CBPR research where children were asked to fill out a survey without participation in other phases of research. Another example is papers that had some elements of PAR methodology (e.g. consultation with stakeholders) utilized, but only included adults in those conversations. | NR |
| Primeau, C., Giddings, C., & Cheeseman, K. (2019). Annotated bibliography: Participatory action research (PAR) and community engaged research (CER) with youth. | Literature | 2019 | 12 to 21 | Overall research | To provide brief summaries of a small sample of the most relevant academic research on creative methods for engaging young people in research. | Participatory research, Photovoice | Participatory research methods: Study approaches in which the planning and conducting of the research process takes place in collaboration with the individuals groups, or communities under study. Typically, researcher-participants also direct the research outputs and how the research will be shared (i.e., knowledge mobilization).  Photovoice: A participatory photography method that lets participants identify, represent, and enhance their community using a specific photographic technique. Study participants are encouraged to capture visual representations of their everyday lives to help identify challenges and opportunities the community faces (Wang & Burris, 1997). | CINAHL, ERIC, Google Scholar, ProQuest, PsychINFO, PubMed, and MEDLINE | NR | Inclusion Criteria - Peer-reviewed articles published between 2008-2018 - Articles published in English - Articles focused on research with youth (aged 12-21 years) - Research involving participatory or arts-based methods - Research involving groups of youth (as opposed to research with individuals) - Focus on Canadian contexts and small-town communities, completed in schools or community settings  Exclusion Criteria  - Research conducted before 2008 - Research involving individuals over 21 years of age - Research involving individuals less than 12 years of age - Research using individual methods, such as individual interviews | NR |
| McLaughlin, H. (2006). Involving young service users as co-researchers: possibilities, benefits and costs. *British Journal of Social Work*, *36*(8), 1395-1410. | Narrative | 2006 | 13 to 20 | Health research | To identify the mandate for involvement of service users, the different levels of service user involvement and the claimed benefits and costs of such an approach. | Involving, co-researchers | Not clearly defined | N/A | N/A | N/A | NR |
| Maticka-Tyndale, E., & Barnett, J. P. (2010). Peer-led interventions to reduce HIV risk of youth: a review. *Evaluation and program planning*, *33*(2), 98-112. | Critical review | 2010 | 15 to 24 | HIV/AIDS | To critically review and synthesize the results and lessons learned from evaluated peer-led programs with an HIV/AIDS risk reduction component that target youth in geographical communities (i.e. communities where they live) and are delivered in low-income countries. | Peer education, Per led programs | Peer education: One approach in HIV prevention programming targeting youth worldwide is to use peer, rather than professional, leaders in what have been referred to as peer education programs. Peer-led programs have been delivered in schools, clinics, community centers, workplaces, and in informal settings where members of target populations congregate. They build on the natural exchange of information between people of similar age or status (Turner & Shepherd, 1999). Peer education and peer-led interventions typically target peer groups and communities rather than individuals as the unit of change, with agents of change coming from within the group or community (i.e. peers) rather than brought in from outside. The approach is based on the assumption that, especially among adolescents, peers learn from each other, are important influences on each other, and that norms and behaviors are most likely to change when liked and trusted group members take the lead in change (Aggleton and Campbell, 2000, Campbell, 2004, Fee and Youssef, 1996, Shiner, 1999, Turner and Shepherd, 1999). | Psychological Literature (PsychLit), Population Information Program (POPLINE), Sociological Abstracts, and MEDLINE | Publication lists from international organizations such as the United Nations Joint Commission on HIV/AIDS (UNAIDS) and the World Health Organization Global Program on AIDS (WHO/GPA). The tables of contents of journals that published articles evaluating interventions with an HIV/AIDS content between 1994 and 2008 | To be included in this review, an intervention had to meet the following criteria: (a) youth (as culturally defined) were included in the target population (most targeted those 15-24); (b) it was delivered in a geographical community; (c) at least some content dealt with knowledge, attitudes, norms, and/or behaviors relevant to the prevention of HIV/AIDS; (d) it was designed to be delivered primarily by youth peers; (e) it was delivered in a low- or middle-income country; (f) it was evaluated (both quantitative and qualitative evaluations were included) and information about the evaluation methods and results was provided; (g) the content and delivery methods were described; (h) the report or paper describing the intervention and its evaluation was available in English or French and published between January 1994 and November 2008. This time period encompassed the dates used in the WHO publication (Ross et al., 2006) where similar syntheses were reported and the last date on which we accessed the literature in preparing this paper. Interventions were excluded if they did not meet these criteria or if they were delivered primarily in a school, workplace, or health facility. | NR |
| Maley, M. (2017). Peer Education for Adolescent Reproductive and Sexual Health. *New York*, 1-4. | Overview | 2017 | NR | SRH | To help inform decisions about using peer education in adolescent reproductive health programs, this article summarizes the results of those reviews, providing an overview of the current state of the evidence. | Peer education | Peer education is a method for intervention or program delivery that uses members of the learner group to partly or fully facilitate program activities. Using members of similar age or status (Tolli, 2012) to share health information is thought to work through the social influence of the peer group, which can have a strong impact on adolescents (Maticka-Tyndale & Barnett, 2010). | Electronic databases were searched using EBSCOHost | NR | NR | NR |
| Grace, R., Knight, J., Baird, K., Ng, J., Shier, H., Wise, S., ... & Kemp, L. (2019). Where are the silences? A scoping review of child participatory research literature in the context of the Australian service system. *Children Australia*, *44*(4), 172-186. | Scoping | 2019 | <18 | Health, mental health and disability related research | To scope the landscape of participatory research on service policy, design, implementation and evaluation in Australia across eight service sectors and to answer questions about where child and youth voice is being sought and where it is silent and the extent to which the current research reflects a diversity of cultural and other life experiences. | Participation | The term 'participation', for the purposes of this paper, is used broadly to describe research in which children have been involved in one or more of the nine stages of a research study, including: (1) determining the research questions; (2) designing the research and choosing the methods; (3) preparing the research instruments; (4) identifying and recruiting participants; (5) collecting data; (6) analysing the data and drawing conclusions; (7) producing a report; (8) disseminating the report and its findings and (9) advocating and mobilising to achieve policy impact (Shier, Reference Shier, Berson, Berson and Gray2019). | Scopus; ERIC; PsycINFO; MEDLINE; CINAHL; CINCH and SocINDEX | Relevant papers were also identified in the reference lists of previously identified papers. | To be included, the paper needed to meet two inclusion criteria:  1.The paper under review needed to present the perspectives of children and young people aged 18 years and younger. We selected 18 years as the cut-off, reflecting both Australian general law which sees 18 years as marking the commencement of the legal rights and responsibilities of an adult, and the UN Convention on the Rights of the Child, cited above, which guarantees the participation rights of all those under 18.  2.The study needed to employ participatory research methods. We applied a very broad definition of 'participatory', requiring that papers report on research that provided an opportunity for children and young people to express their views on the topic at hand, to be treated as social actors whose experiences and opinions were important. This included both qualitative and quantitative studies where participating children and young people were asked to either talk about their views and experiences, present them in a creative form (e.g. photos and drawings) and discuss what their creations represented to them, or give their opinions in a survey form. Very broadly, the research needed to be with or by children, and not on children (i.e. children treated as the objects of study without opportunity to express their own opinions) (Mason & Watson, Reference Mason, Watson, Ben-Arieh, Casas, Franes and Korbin2014). If, on the other hand, the researchers had included some qualitative or quantitative questions in their design asking the participating children to share their perspectives on bullying and/or its impact, then the study would be included. | NR |
| Gilchrist, F., Rodd, H. D., Deery, C., & Marshman, Z. (2013). Involving children in research, audit and service evaluation. *British dental journal*, *214*(11), 577-582. | Narrative | 2013 | NR | Oral Health | To discuss methods for actively involving children in research, audit and service development and to consider the ethical issues that may be encountered when conducting such studies. | Active involvement | Active involvement can be defined as: where children are seen, listened to and heard and where priority is given to establishing children's own perspectives. | N/A | N/A | N/A | NR |
| Brown, A., Spencer, R., McIsaac, J. L., & Howard, V. (2020). Drawing out their stories: A scoping review of participatory visual research methods with newcomer children. *International Journal of Qualitative Methods*, *19*, 1609406920933394. | Scoping | 2020 | <18 | Overall research | To systematically map how PVM have been used in research with newcomer children. | Participatory visual methods | For the purpose of this review, 'participatory visual methods' is an umbrella term capturing a broad range of methods including, but not limited to, the use of drawing, photography, digital storytelling, or mapping to share stories in a visual narrative (Richards, 2011). | Academic Search Premier, Child Development & Adolescent Studies, CINAHL Plus, Education Research Complete, ERIC, MEDLINE, PsycArticles, PsycINFO, and SocINDEX | NR | Studies were included for consideration if they were peer-reviewed, English-language, and used visual participatory methods with newcomer children (exclusively or in combination with other approaches). We made a distinction between studies exploring PVM within interventions, therapies, or academic programs and studies exploring PVM for research; we included only those studies exploring PVM as research. Studies were excluded if the primary participants were youth and did not include children younger than 16. Studies were also excluded if there was no discussion regarding outcomes of newcomer children's participation in the research or little description of the methods, context, or ethical considerations. Note, articles did not have to include a robust discussion of all these elements to be included (i.e., if an article articulated well the outcomes of newcomer children participation but did not fully address ethical considerations, it was still included). Finally, articles were excluded if they did not report original research (e.g., discussion papers, book reviews, conference proceedings); review articles were not included, though their reference lists were hand-searched. | NR |
| Asuquo, S. E., Tahlil, K. M., Muessig, K. E., Conserve, D. F., Igbokwe, M. A., Chima, K. P., ... & Tucker, J. D. (2021). Youth engagement in HIV prevention intervention research in sub‐Saharan Africa: a scoping review. *Journal of the International AIDS Society*, *24*(2), e25666. | Scoping | 2021 | 10 to 24 | HIV | To categorize and determine the extent of youth engagement in HIV prevention research in sub-saharan Africa using a scoping review. | Youth engagement | We used Hart's ladder to specify the extent of youth engagement. Hart's ladder is a typology that describes different degrees of youth engagement in projects or programmes. It has eight steps, which progress in a bottom to top fashion, from no engagement to different degrees of engagement. We modified Hart's ladder by grouping the steps with youth engagement into substantial, moderate, minimal and no youth engagement, based on the decisionâ€making power of the youth in the research study. Substantial youth engagement was defined as research activities that were youth-initiated and directed. Adults either created an enabling environment or made relevant contributions, with youth having substantial decision-making power and opportunities for youth leadership. Moderate youth engagement was defined as adultâ€initiated activities with shared decision making between youth and adults. Minimal youth engagement was defined as youth being consulted to get their opinions, assigned specific roles or informed about events surrounding research activities, without any decisionâ€making power. No youth engagement was defined as the absence of participatory approaches or activities during research. We assumed that meaningful youth engagement would be described in the research study. | PubMed, Global Health, Scopus, Embase, Cochrane, ClinicalTrials.gov | Open Gray | Inclusion criteria were behavioural and biomedical research studies with human subjects conducted between January 2000 and January 2020, focused primarily (>50%) on youth, related to HIV prevention intervention, and conducted in sub-Saharan Africa. Studies with stakeholder engagement in the manuscript but without youth engagement were still included. We excluded records that were focused on secondary prevention for youth living with HIV; were cross-sectional or observational; were systematic or narrative reviews; were secondary data analyses; or were not written in English. | NR |
| Agdal, R., Midtgård, I. H., & Meidell, V. (2019). Can asset-based community development with children and youth enhance the level of participation in health promotion projects? A qualitative meta-synthesis. *International Journal of Environmental Research and Public Health*, *16*(19), 3778. | Systematic | 2019 | NR | Health research | To review literature on ABCD employed in projects with or by children and youth, and ask if, and how, ABCD enhance the level of participation. Can ABCD enhance the participation of children and youth in health promotion projects? | Asset-based community development (ABCD) | Asset-based community development (ABCD) is one of several approaches employed in health promotion strategies to engage communities, focusing on establishing networks and collective action. Kretzmann and McKnight suggest that an ABCD process can be defined by three characteristics:  Citizen led. Local citizens map their resources and needs and lead the collaboration with outside partners. Relationship oriented. There is a focus on building social networks. Asset-based. The process focusses on strengths, resources, and assets. | Cinahl, Scopus, Book citations Scopus, Book citations WoS, Web of Science, Embase, Eric, Medline, PsycInfo, SocIndex | NR | They should either refer to ABCD as the practical approach, or refer to the original handbook of ABCD as the fundament for the approach. texts that included children, schools or youth, no older than twenty-five years. The limit of twenty-five was set to potentially include studies with mixed age groups in schools. We did, however, not identify studies with this wide age range. The oldest youth in the studies were upper secondary school pupils. | EPICURE elements |
| Fauk, N. K., Kristanti, E., Ratu, M., & Ambarwati, A. Effectiveness of School-based, Peer-led Sexual Health Interventions in Increasing Stis/hiv Knowledge Amongst Adolescents: a Systematic Review. | Systematic | 2017 | 13 to 18 | STIs/HIV | To assess the effectiveness of school-based, peer-led interventions in increasing STIs and/or HIV-related knowledge amongst adolescents in low- middle- and high-income countries. | Peer education | Peer Education: Peer educators inform and encourage other students (peers) to recognize their risk and protect themselves from contracting STIs and/or HIV. The goal of peer education is to make prevention of STIs and HIV a peer norm. | PubMed, ERIC and the Cochrane | NR | Inclusion criteria were: studies that investigated peer-led interventions for adolescents in a school setting in which the main or one of the components was the improvement of knowledge regarding STIs and/or HIV/AIDS; and only studies that made use of a comparison group were included because by using a comparison group, interventions can be compared and the effectiveness of interventions can be measured. There were no restrictions on study duration and follow-up period because these varied greatly between interventions. Exclusion criteria were: studies that investigated interventions that were not peer-led or interventions that took place outside the school-setting, interventions without comparison groups; studies that were conducted before 2005; studies that were not written in English; and studies that did not include knowledge as an outcome. | JADAD scale |
| Hunt, S. Evidence for the effectiveness of peer-led education for at risk youth: an Evidence Check rapid review brokered by the Sax Institute ( https://www. saxinstitute. org. au/) for the NSW Ministry of Health. 2016. Available from: www. saxinstitute. org. au/wp-content/uploads. *Evidence-for-the-effectiveness-ofpeer-led-education-for-at-risk-youth. pdf*. | Rapid | 2016 | 16 to 24 | Substance abuse | To review and summarise the evidence base for the effectiveness of peer-led education for promoting resilience and harm minimisation in alcohol and other drug use among at risk youth. | Peer led programs | The most common forms of peer-led education programs involved coaches or peer-support workers to assist the uptake of intervention content and processes within a peer group | A+ Education, CINAHL, Cochrane Economic Evaluations, Cochrane Other Reviews, Cochrane Reviews, Cochrane Trials, Embase, ERIC, Medline, Medline in Process, PsycEXTRA, and PsycINFO | Grey literature regarding peer-led interventions for substance use and similar areas in drug use prevention (e.g. HIV prevention) were also searched to identify related initiatives underway in the community | This review took a focus on studies of interventions with at risk populations where the following criteria were met: 1. Peer involvement was a key component of the intervention (key criterion) 2. The study was published between January 2006 and February 2016 (key criterion) 3. The study was written in English (key criterion) 4. The study reported on intervention outcome data (secondary criterion) 5. Participants ranged in age from 16-24 years (secondary criterion); and 6. Participants were at risk of AOD use due to social, economic or health factors such as not attending school or work, exposure to juvenile justice, living in a low socio-economic area, living in a regional or remote area, and estrangement from family or other sources of support (secondary criterion). | NR |
| Norton, M. J. (2021). Co-production within child and adolescent mental health: A systematic review. *International journal of environmental research and public health*, *18*(22), 11897. | Systematic | 2021 | <18 | Mental Health | To provide a systematic review of the peer-reviewed, academic, and best-available evidence on the concept of co-production within child and adolescent mental health. | Co-production | "The creation of a dialogical space where the service user, family members, carers and service providers enter a collaborative medical partnership to improve their own care and service provision." | CINAHL, JSTOR, PsycARTICLES, PsycINFO, PubMed, Science Direct, Web of Science, Wiley Online Library | The references of the already included papers | Inclusion - Qualitative, Mixed-Method Research Articles  - English Language  - Peer Reviewed  - Child and Adolescent Mental Health Services  - Articles focused on co-production in young people   Exclusion Editorials, Quantitative Studies, Discussion Papers, Literature Reviews/Systematic Reviews/Meta-Syntheses, Meta-Analyses Addiction, Intellectual Disabilities, Physical Health, Older Person Servicesâ€”Dementia, Delirium, etc., Dual Diagnosis, Dissertations, Article focused on co-production in older users of service. | An automated tool developed by McGuiness and Higgins and an adaptation of the tool created by Hawker and colleagues was used. |
| Lindberg, S. (2013). Involving Children in the Design of Online Peer Support for Children with Cancer. | Literature | 2013 | NR | Cancer | To identify properties of design methods that include children in online peer support interventions | Peer support | Peer support builds on the underlying idea that people who have shared experiences can better help each other deal with some issues caused by their problem than physicians can (Klemm & Hardie, 2002). | Google Scholar | NR | Articles were chosen based on their relevance to the subject of design with children. The focus was on participatory design with children, but any literature found relating to design where children had an active part was included. The relevance of the articles was first judged by a scan of the abstracts. | NR |
| Hackett, L. (2019). Engaging Indigenous Youth in Community-Based Participatory Action Research: A Scoping Review. | Scoping | 2019 | NR | Overall research | To identify studies in the academic peer-reviewed literature in which Indigenous youth in Canada and the US were engaged as decision-makers in CBPAR projects. | Community-based participatory action research (CBPAR) | Community-based participatory action research (CBPAR): CBPAR designs prioritize community interests, use culturally-relevant data collection strategies, and include phases devoted to mobilizing findings for community benefit (Kemmis & McTaggart, 2000; Wadsworth, 1998). | MEDLINE (accessed via Ovid), Scopus, and PsycINFO (both accessed via EBSCO). | NR | Inclusion Criteria   - Published in 2000 or later - Available in English  - Title/abstract indicates focus on PAR or comparable research method and Indigenous youth  - Indigenous youth are included in decisionmaking about purpose, design, analysis, or knowledge translation/action - Study population is based in Canada or the US  Exclusion Criteria - Published in 1999 or earlier -,Not available in English - Does not discuss the real extent and nature of community engagement - Indigenous youth are not included in decision-making about purpose, design, analysis, or knowledge translation/action - Systematic reviews  After full-text review, studies were included if they reported engaging Indigenous youth participants in making decisions about at least one of the following areas: (1) research objectives; (2) data collection strategies; (3) meaning of data; or (4) approaches to knowledge translation and action (KT/A). Furthermore, eligible studies needed to discuss the real (as opposed to intended) extent and nature of involvement of Indigenous youth research partners. | NR |
| Jacquez, F., Vaughn, L. M., & Wagner, E. (2013). Youth as partners, participants or passive recipients: A review of children and adolescents in community-based participatory research (CBPR). *American journal of community psychology*, *51*(1), 176-189. | Literature | 2013 | NR | Overall research | To identify the frequency with which youth are included as partners and the role of youth in the research process using CBPR. | Community-based participatory research (CBPR) | Community-based Participatory Research (CBPR) is an orientation to research that values the role of community members and academicians as equitable partners, each contributing unique strengths to the research process (Israel et al. 1998). In contrast to more traditional research, in which 'experts' from academic institutions develop hypotheses based on the literature and then enter communities to recruit subjects, projects using a CBPR orientation rely on the input and contribution not only from academic researchers but also from community members at each step of the research process, from defining the research topic to disseminating the results (Minkler and Wallerstein 2008). Although specific research methods used in CBPR vary widely, CBPR projects are distinguished by the value placed on cooperative efforts that engage community members and researchers equally in decision-making and in the relevance of the research for communities (Israel et al. 2005). | PubMed, Scopus, and Web of Science databases | NR | Inclusion criteria: English-language articles published from 1985 to January 31, 2012 that referenced CBPR and youth. Only published articles in refereed journals were included. Because we were interested in articles willing to claim the terminology of CBPR, we excluded those articles only alluding to participatory practices and not specifically described as CBPR. | NR |
| Kennedy, H., DeChants, J., Bender, K., & Anyon, Y. (2019). More than data collectors: A systematic review of the environmental outcomes of youth inquiry approaches in the United States. *American Journal of Community Psychology*, *63*(1-2), 208-226. | Systematic | 2019 | <25 | Overall research | To ummarize the findings from empirical studies of youth inquiry approaches in the United States, with a focus on their environmental outcomes. | Youth participation, involvement , youth organizing, student voice, youth participatory action research (YPAR) | Youth participation refers to children and adolescents' involvement in meaningful and sustained efforts to improve the settings, systems, and organizations that impact them (Ozer, Afifi, Gibbs, & Mathur, 2018). Several models of youth participation, such as youth organizing, student voice, and youth participatory action research (YPAR), engage young people in conducting inquiry to generate new knowledge about their lives, schools, and communities. These youth inquiry approaches are characterized by young people investigating topics that are important to them by collecting, analyzing, and interpreting information, data, or evidence (Rodriguez & Brown, 2009). | PubMed, ERIC, Social Service Abstracts, and PsychInfo | NR | Eligibility criteria focused on four key elements: (a) study characteristics (empirical research, published in peer-reviewed journals, conducted in the United States [so as to focus in one socio-political context of which we are familiar], published in English); (b) target population (project participants were comprised of children or youth 25 years or younger; for youth ages 18-25, samples were excluded if they consisted only of undergraduate or graduate students, as this group appeared to represent young adults living independently); (c) inquirybased process that involved youth in data collection, data analysis, data interpretation, or use of knowledge to improve lives); and (d) outcomes (study reported on the experiences, outcomes, or impact of youth inquiry on youth participants or their surrounding environment). | NR |
| Kim, C. R., & Free, C. (2008). Recent evaluations of the peer‐led approach in adolescent sexual health education: A systematic review. *Perspectives on sexual and reproductive health*, *40*(3), 144-151. | Systematic | 2008 | 14 to 26 | SRH | To conduct a systematic review and methodological appraisal of randomized and quasi-randomized controlled trials of peer-led sex education interventions. | Peer-led | Peer-led sexual health education is one means of addressing deficiencies in adolescent sexual health. Defined as the teaching or sharing of information, values, and behaviors by members of similar age or status group. | EMBASE, ERIC, PubMed, International Bibliography of Social Science, PsycINFO, specialized bibliographic registers, DoPHER and the Cochrane Central Register of Controlled Trials | Because of resource constraints, unpublished works were omitted. | We examined all randomized and quasi-randomized controlled trials that evaluated interventions to promote adolescent sexual health using peer educators and that were published in 1998-2005. Any peer-led intervention intended to promote sexual health in any setting (e.g., health center, youth group, local extracurricular center, school) in high-, middle- or low-income countries was eligible. For inclusion in the review, studies had to have intervention and control groups, include adolescents aged 10-19 and be published in English.In addition, studies were required to meet four methodological criteria: The studies had to include a control or comparison group whose social and demographic characteristics were similar to those of the intervention group, provide preintervention data for all groups, provide postintervention data for all groups and report all outcomes. Primary outcomes of interest were the occurrence of pregnancy or STIs, age at first sex, number and types of sexual partnerships, condom use and contraceptive use. Relevant secondary outcomes were measures of knowledge of sexual health or contraceptive services; behavioral intentions regarding sex or contraceptive use; and attitudes about sex, sexual health or contraceptives. | Criteria developed by the Evidence for Policy and Practice Information and Coordinating Centre, with additional criteria based on the Cochrane review guidelines. |
| Moilanen, T., Pietilä, A. M., Coffey, M., & Kangasniemi, M. (2018). Adolescents’ health choices related rights, duties and responsibilities: an integrative review. *Nursing ethics*, *25*(4), 418-435. | Integrative | 2018 | 10 to 19 | Health research | To identify, describe and synthesize previous studies on adolescents' health choices in relation to their rights, duties and responsibilities. | Involvement | NR | CINAHL, PubMed, Web of Science and Scopus databases | Manual searches were conducted in order to avoid the search bias and to maximize the number of relevant studies.The journals that included the selected articles were scrutinized, together with their reference lists. In addition, two journals, Nursing Ethics and Bioethics, were included in the manual searches because of their close links to our research topic | Our inclusion criteria were that the focus of the original study was on healthy children or adolescents (10-19 years old), that the focus of the paper was on health choices1-3 and that it covered rights, duties or responsibilities. The exclusion criteria were that the original study focused mainly on adults, a specific disease, such as diabetes or the human immunodeficiency virus, or a particular health-related decision, such as vaccination or tooth-brushing frequency, or a reproductive health issues, such as pregnancy and breast-feeding, or an environmental issues affecting health choices, such as the influence of buildings or food menus. In addition, studies that reviewed other studies were excluded. | NR |
| Tolli, M. V. (2012). Effectiveness of peer education interventions for HIV prevention, adolescent pregnancy prevention and sexual health promotion for young people: a systematic review of European studies. *Health education research*, *27*(5), 904-913. | Systematic | 2012 | 10 to 24 | SRH | To perform a systematic description of peer education programs for HIV prevention, adolescent pregnancy prevention and promotion of sexual health among young people conducted in the European Union in order to determine their effectiveness. | Peer education | Peer education, defined as the teaching or sharing of health information, values and behaviours by members of similar age or status | Medline, Psyinfo and Psyindex | NR | Inclusion criteria We considered as relevant for this systematic review, all studies that described and evaluated an intervention designed to be implemented, in full or partially, by peer educators and that had as objective the prevention of HIV, the prevention of adolescent pregnancy and/or the promotion of sexual health in young people, aged between 10 and 24 years.  In addition, the studies were required to meet the following criteria: (i) the intervention was conducted in any of the 27 member countries of the European Union, (ii) it was evaluated using randomized controlled trials (RCTs), it was a non-randomized controlled study (CTs) or it had a before-and-after study design (non-CTs) (iii) at least one of the outcomes considered relevant in the present work were evaluated. The primary outcomes of interest were occurrence of unintended pregnancy and STDs, sexual experience, age at first sexual intercourse, number of sexual partners and use of contraceptive methods. Relevant secondary outcomes were knowledge about sexual health, HIV, contraceptive methods or sexual health services; behavioural intentions regarding sex or contraceptive methods; skills regarding communication and negotiation and attitudes toward sex, sexual health, contraceptive methods or people living with AIDS, (iv) as acceptable comparators for the intervention are taken no intervention or standard practice and (v) the article describing the intervention and evaluation was available in English, German or Spanish. | Criteria catalogue developed by the Institute of Health Economics and Clinical Epidemiology of the University of Cologne |
| Yip, C., Gates, M., Gates, A., & Hanning, R. M. (2016). Peer-led nutrition education programs for school-aged youth: a systematic review of the literature. *Health education research*, *31*(1), 82-97. | Systematic | 2016 | 5 to 18 | Nutrition | To summarize the results of published, refereed research investigating peer-led nutrition education programs in schools in the United States and Canada, and identify research gaps and provide recommendations for future research. | Peer led | Not clearly defined | PubMed (Medline), Scopus and Education Resources Information Center (ERIC) databases | Reference lists were scanned to retrieve articles that were not previously identified. | Inclusion Criteria  - included nutrition education that was led by peers (same age or older) - took place in a school (elementary or high school) - investigated outcomes for school-aged youth (5-18 years old) - took place in Canada or the United States - reported quantitative results - published in English - published between January 2000 and November 2013  Exclusion criteria - did not include an intervention or was not led by peers - did not include nutrition education - did not take place in a school - investigated outcomes for individuals <5 or >18 years of age - took place in a country other than Canada or the United States - did not report quantitative results - not published in English - published before January 2000 or after November 2013 - not a peer-reviewed primary study  Multi-component programs that incorporated a peer component (either same-age or older peers) were eligible, but only results relevant to peer influences were included for the purposes of this review. The setting was restricted to schools in Canada and the United States to limit heterogeneity in school systems. | With respect to the assessment of the risk of bias, the methodologies of the studies included in the review were accounted for |
| Pusmaika, R., & Novianti, L. L. (2017). The positive impact of school-based peer education program towards adolescent sexual behavior: A systematic review. *LIFE: International Journal of Health and Life-Sciences*, *3*(1), 69-81. | Systematic | 2017 | NR | SRH | To positively impact school-based peer education program for adolescent sexual behavior. | Peer education | Not clearly defined | Scopus, ProQuest, SpringerLink, ScinceDirect, and JSTOR database | NR | Selected studies for inclusion in the systematic review are researches that qualify as follows: (1) research that includes health programs at school through peer education; (2) is an experimental research design; (3) the population or sample is teenagers/students/college students; (4) the substance could be a reproductive health; (5) research shows indicators of sexual behavior (condom use, sexual intercourse, sexual harassment behavior). | NR |
| Powell, M. A., Fitzgerald, R. M., Taylor, N., & Graham, A. (2012). International literature review: Ethical issues in undertaking research with children and young people. *Childwatch International Research Network*. | Literature | 2012 | NR | Overall research | To review the recent literature regarding ethical issues in research with children and young people. | Undertaking research with children and young people | Research with children, who are actively participating and expressing their views and opinions | NR | NR | NR | NR |
| Larkins, C., Nowland, R., Robertson, L., Farrelly, N., Sharpe, D., Roy, A. N., ... & de Lemos, J. B. (2021). Peer research by children and young people and their allies Rapid Evidence Review of best practices in health and social science literature. | Rapid | 2021 | 5 to 25 | Overall research | To identify theoretical principles and practice modes and mechanisms of what works in peer research in the field of health and social sciences, that are generalisable as a basis for designing effective peer research projects, protocols and establishing best practice. | Peer research | We used a working definition of peer research as studies where children and/or young people are explicitly involved in at least one stage of the research process beyond collecting data about themselves and beyond involvement in dissemination or recruitment. This excluded, for example, studies which involved child participants who gave information about their ideas and experiences and then helped create an accessible summary. | PsycINFO, Medline, CINAHL, Embase, SocINDEX, ASSIA: Applied Social Sciences Index and Abstracts (Proquest), Social Care Online and SCOPUS | NR | We used a working definition of peer research as studies where children and/or young people are explicitly involved in at least one stage of the research process beyond collecting data about themselves and beyond involvement in dissemination or recruitment. This excluded, for example, studies which involved child participants who gave information about their ideas and experiences and then helped create an accessible summary. We included studies about peer research involving children and young people (aged 5-25 years) and excluded peer research conducted exclusively between adults. Using an adapted version of Vaugh et al. (2018) papers were categorised as follows:  Review papers: scoping or systematic reviews of studies or projects of peer research, including grey literature reviews/reports Process and descriptive papers: articles describing lessons learnt or a description of the programme,process or training of a peer model About papers: articles that focused on the peers themselves and their experiences within a peer model/approach  Generalised critique: articles where an author reflects on the peer research methodology and/or critiques of the approach or reflects on specific aspects relating to peer research with children and young people (i.e. issues to do with ethics).  Empirical papers only including findings of the studies were not included. We also excluded dissertations, editorials, opinion pieces, commentaries, book or movie reviews, protocols, case studies and erratum. Only studies written in English were included and those published after 2000. | NR |
| Wilson, O., Daxenberger, L., Dieudonne, L., Eustace, J., Hanard, A., Krishnamurthi, A., ... & Vergou, A. (2020). A rapid evidence review of young people’s involvement in health research. *London: Wellcome*, *3*. | Rapid | 2020 | 10 to 24 | Health research (key areas of adolescent health defined by the Lancet Commission on Adolescent Health and Wellbeing: infectious and vaccine preventable diseases, undernutrition, HIV and AIDS, sexual and reproductive health, unintentional injuries, violence | To identify the strengths, weaknesses, and gaps in the literature and develop a framework for youth involvement in health research. | Involvement | For the purposes of this review, 'involvement' means research that is done 'with' or 'by' young people, as opposed to 'for', 'about', or 'on' them (adapted from NHS Health Research Authority, 2020b). Young people can contribute to research through tasks like defining research agendas, designing research, collecting and analysing data, or disseminating and translating findings. In the literature terms such as engagement or participation are often used interchangeably to the term involvement. | PubMed and Science Direct | Articles that appeared in the keyword search that met the relevance and quality criteria were also screened so that the articles referenced in them could be considered for review. An EAG was convened during this study to bring together a small group of experts in the field of young people's involvement in research, some of which were young people.The group's role was to direct the research team to articles that did not appear in the keyword search or during initial snowballing from the bibliography of relevant papers. Furthermore, Wellcome staff members provided additional papers for consideration. Both sets were reviewed using a full read to test for relevance and quality criteria. | Articles were considered relevant when they described the involvement of young people in health research or any aspect of this involvement deemed relevant to the review. Articles were excluded when they were describing tokenistic involvement of young people or provided insufficient description of the nature of youth involvement. | NR |
| O’Kane, C. (2018). Review of children’s participation in humanitarian programming. | Literature | 2018 | NR | Humanitarian program(including health research) | To review children’s participation in the humanitarian response. | Participation | Participation is about having the opportunity to express a view, influencing decision making and achieving change. Children’s participation is an informed and willing involvement of all children, including the most marginalised and those of different ages and abilities in any matter concerning them directly or indirectly. Children’s participation is a way of working and is an essential principle that cuts across all programmes, and takes place in all arenas, from homes to government and from local to international levels. | NR | NR | NR | NR |
| A Mapping of Child Participation Initiatives in Public Decision Making and Monitoring | Evidence map | 2020 | 12 to 18 | Decision making (Health research) | To analyse mainly qualitative information concerning the form and structures, scope, quality, and outcomes of children’s participation processes in public decision making and monitoring. | Participation | Participation: Article 12 of the CRC recognises that children have a right to be heard and taken seriously. The application of this right has been expressed as ‘participation’, although the term itself does not appear in the article. It is a very broad ranging right which:  • applies to every child capable of forming his or her own views. This does not just mean older children. The very youngest children, as well as children with disabilities, are able to form views, even where they are not able to communicate them verbally.  • requires that children are able to express their views freely – in other words, that space and time are created for them to be heard and they express themselves voluntarily and safely. • applies to all matters that affect children whether they relate to the home, school, local community or local and national government.  • requires that children’s views are given due weight in accordance with their age and maturity. This means it is necessary to take account of how far they have the capacity to understand the implications and consequences of those views.  At a minimum, children’s participation in public decision-making and monitoring processes must comply with the nine basic requirements for the implementation of the right of the child to be heard that are set out in the UN Committee on the Rights of the Child’s General Comment No. 12. This states that children’s participation must always be: transparent and informative; voluntary; respectful; relevant; child-friendly; inclusive; supported by training (of adults and children); safe and sensitive to risk; and is accountable to children. | N/A | N/A | N/A | NR |
| Jenkinson, K. A., Naughton, G., & Benson, A. C. (2014). Peer-assisted learning in school physical education, sport and physical activity programmes: a systematic review. *Physical Education and Sport Pedagogy*, *19*(3), 253-277. | Systematic | 2013 | 5 to 18 | Physical activity | To evaluate existing PAL intervention approaches and assess the effectiveness of these approaches on physical activity and physical education participation and behaviours, motor skill performance, health behaviours, psychosocial behaviours and anthropometric outcomes; to evaluate the selection criteria and the training process undertaken by tutors and tutees in the implementation of PAL interventions. | Peer-assisted learning (PAL) | Peer-assisted learning (PAL) is one generic label used to describe teaching strategies and models that facilitate peer education practice (Ward and Lee 2005) and will be used throughout this review to represent the collective ‘PAL’ process. Commonly used teaching models that incorporate various components of peer-based interaction and learning in physical education and school physical activity programmes include: peer tutoring, peer teaching and class-wide peer tutoring (Byra 2006; Damon and Phelps 1989; Meztler 2005; Ward and Lee 2005). | AMED (1985 to January 2012); EBM Reviews – ACP Journal Club (1991 to December 2011); EBM Reviews – Cochrane Register of Controlled Trials (Fourth Quarter, 2011); EBM Reviews – Cochrane Database of Systematic Reviews (2005 to December, 2011); EBM Reviews – Cochrane Methodology Register (First Quarter, 2012); EBM Reviews – Database of Abstracts of Reviews of Effects (Fourth Quarter, 2011); EBM Reviews – Health Technology Assessment (First Quarter, 2012); EBM Reviews – NHS Economic Evaluation Database (First Quarter, 2012); ERIC (1966 to present); Ovid MEDLINE(R) (1946 to January, Week 2, 2012); Ovid MEDLINE(R) Daily Update (20 January 2012); Pre-MEDLINE (most recently published); Ovid OLDMEDLINE(R) (1947 –1965); PsycINFO (1806 to January Week 3, 2012); CINAHL (1981 to January Week 3, 2012); SPORTS DISCUS (1830 – January Week 3, 2012). | NR | Studies that met the following criteria were included in this review: (i) published in English; (ii) cohorts were children or adolescents (aged 5 – 18); (iii) physical activity, physical education or sport interventions; (iv) included a PAL component that was reciprocal or unidirectional with peer interactions occurring predominately in dyads (pairs); (v) interventions were in schools; (vi) interventions were conducted post1990. Thesis, doctoral dissertations, conference proceedings, edited books and structured abstracts were excluded. Subsequent publications derived from these were searched. In addition, cooperative learning interventions were also excluded due to the lack of a defined tutor role | NR |
| Cullen, O., & Walsh, C. A. (2020). A narrative review of ethical issues in participatory research with young people. *Young*, *28*(4), 363-386. | Narrative | 2020 | 15 to 25 | Overall research | To provide a narrative review of peer-reviewed literature regarding ethical issues of involving young people in health research | Participatory action research (PAR) | Participatory action research (PAR) is a research methodology with a focus on emancipatory ideologies and engages directly with communities as co-researchers to create knowledge and social action beneficial to those living in the communities (Kidd & Kral, 2005; McTaggart, 1991). PAR is used as a research method to address issues of social injustice and marginalization (Cahill, Quijada Cerecer, & Bradley, 2010). Youth participatory action research (YPAR) is a form of PAR with an explicit focus on youth involvement in the research process. | PsycInfo; CINAHL Plus with Full Text; Social Work Abstracts; Family and Society Studies Worldwide; Family Studies Abstracts; SocINDEX with Full Text;ERIC; and PubMed. | N/A | Criteria for inclusion were that the article focused on participatory research with an examination of ethical issues. Articles were excluded if the population focus was adults, or exclusively young children (under the age of 15). Articles that included young people across childhood, adolescence and emerging adulthood were included. In this review, youth refers to adolescence (ages 15–17) and emerging adulthood (18–25) based on criteria outlined by Arnett (2014). | NR |
| Frerichs, L., Ataga, O., Corbie‐Smith, G., & Tessler Lindau, S. (2016). Child and youth participatory interventions for addressing lifestyle‐related childhood obesity: a systematic review. Obesity reviews, 17(12), 1276-1286. | Systematic | 2016 | 5 to 18 | Obesity | To identify and describe the specific types of interventions, theoretical foundations, engagement strategies and challenges involved in studies, examine the impact of child and youth participatory interventions on obesity and obesity-related lifestyle, and summarize reported adverse effects. | Engagement, participation, participatory approaches | The degree of participation ranges from a functional approach where youth are “assigned but informed” (i.e., provided clear information about the purpose of the project and volunteer for meaningful roles) to more interactive approaches where youth conceive and carry-out their projects with adult guidance. Participatory approaches may be more sustainable because the research tends to focus on interests that arise from the community, rather than solely driven by researchers’ academic pursuits. | PubMed/Medline, psychINFO, and ERIC | NR | Inclusion criteria were studies that engaged a subset of children or youth (from age 5 up to college-age youth) in participatory roles to design or implement obesity or obesity-related lifestyle interventions that targeted their peers or younger children. Studies that engaged older youth (ages 19–26) in participatory roles were excluded unless the targeted intervention recipients were ages 5–18. Studies were excluded if engagement of children or youth was limited to involvement of a few children or youth on majority adult advisory committees. Studies were also excluded if engagement with children or youth was limited to formative research (e.g., focus groups, interviews). For inclusion, studies were required to report a diet, physical activity, or weight outcome that was measured at baseline and at least once following intervention implementation among the peers or younger children who were targeted by the intervention. We included prevention, early intervention, or treatment studies that used controlled before and after (CBA), individual randomized controlled trial (RCT), or cluster randomized controlled trial (CRT) designs; all other study designs were excluded. | Risk of bias assessed using guidelines from the Cochrane Collaboration Handbook for Systematic Review of Interventions based on domains of: sequence generation, allocation concealment, blinding of outcome assessment, incomplete outcome data, and selective reporting |
| Yang, Y., Lim, A. C., Wallace, R. E., Marhefka-Day, S., & Liller, K. D. (2020). Photovoice and youth on violence and related topics: a systematic review. Florida Public Health Review, 17(1), 6. | Systematic | 2020 | 12 to 18 | Violence, community safety, risk behaviors, substance use, mental health | To explore how photovoice has been applied to violence and related studies among youth. | Photovoice | Photovoice is a qualitative method that enables individuals, including youth, to identify health issues and enhance their community through phototaking and photo discussions. The main steps and goals of photovoice are to explore community assets and issues through multiple sessions of photo-taking, to inspire critical thinking of community issues via follow-up photo discussions, and to take actions and reach policy makers through photo exhibitions and other advocacy activities. | PubMed, CINAHL,PsycINFO, Embase, and Web of Science | NR | Inclusion criteria were predefined in the protocol as follows: 1) research method: photovoice; 2) types of participants: adolescents, youth, middle/high school students, or the median age of participants between 12-18 years old; 3) types of topics: violence, community safety, risk behaviors, substance use, mental health (e.g. depression), gang activities; 4) types of studies: qualitative study using the technique of photovoice, mixed methods study including photovoice; 5) language: English; and 6) published after January 1997, when the term “photovoice” was first mentioned and the photovoice methodology was formally developed. | McMaster Critical Review Form for critical appraisal of qualitative research |
| Kim, J. (2016). Youth involvement in participatory action research (PAR): Challenges and barriers. Critical Social Work, 17(1). | Literature | 2016 | NR | Overall research | This paper examines the major challenges and barriers to engaging youth in PAR. | Participatory Action Research (PAR), Community-based participatory action research | Participatory (action) research focuses more broadly on increasing participants’ voices and power in the research context, while action research is more interested in facilitating social action to solve problems (Jason, Keys, Suarez-Balcazar, Taylor, & Davis, 2003). Community-based participatory research tends to emphasize a community as a group of participants that share common identities and interests (Banks et al., 2013; Israel, Schultz, Parker, & Becker, 1998). However, current researchers have not clearly distinguished these methods, but used them interchangeably by incorporating their key elements (Cargo & Mercer, 2008; Pain & Francis, 2003). | ERIC, EBSCO Host, JSTOR, SocINDEX, Social Work Abstracts | NR | Selection criteria: (a) providing empirical evidence or actual cases (b) engaging youth in the research process as active partners or (c) offering sufficient information about research procedures, the extent of youth participation, outcomes, and lessons learned from a PAR project. | NR |
