# Supplementary material for: An umbrella review of reviews on challenges to meaningful adolescent involvement in health research
Source: Health Expect. 2024 Jan 27;27(1):e13980. doi: 10.1111/hex.13980 (PMC10821743; doi:10.1111/hex.13980)
Supplement: Supplementary file 1 — Supporting information. [file HEX-27-e13980-s001.zip › PROSPERO registration and protocol paper draft/Protocol paper.pdf]

Involving adolescents in the design, implementation, evaluation, and dissemination of health research: an umbrella review protocol

Azza Warraitch*[1,2], Qusai Khraisha[1,2], Kristin Hadfield[1,2]

[1] Trinity Centre for Global Health, Trinity College Dublin, Dublin, Ireland.

[2] School of Psychology, Trinity College Dublin, Dublin, Ireland.

*Correspondence: warraita@tcd.ie

Abstract

Background: Despite growing recognition of the importance of participatory approaches in youth health research, less than 1% of studies on child and adolescent health report youth involvement. Given this gap, there is a need for an in-depth understanding of the challenges associated with youth involvement and the strategies to mitigate these challenges. To address this, this umbrella review will: i) summarize and synthesize findings from reviews on involving young people in health research, ii) consolidate the challenges experienced in youth involvement and the recommendations to mitigate these challenges, iii) identify the best practices in involving young people in health research, and iv) identify gaps and methodological weaknesses in the extant literature.

Methods: We will include review articles exploring adolescents' involvement (aged 10-24 years) in studies aiming to improve their physical or mental health. Databases to be searched include Cochrane Database of Systematic Reviews, Medical Literature Analysis and Retrieval System Online (MEDLINE), Scopus, Embase, PsycINFO, PsycArticles, Cumulative Index to Nursing and Allied Health Literature (CINAHL), Epistemonikos, and Health Systems Evidence. Grey literature search will be conducted in Web of Science, ProQuest, Google Scholar, PROSPERO, and OpenGrey, supplemented by a hand search of the reference lists of eligible reviews, relevant journals, websites of organizations, and input from experts. Data will be analyzed using narrative synthesis.

Discussion: This review will identify the most effective methods of involving young people in health research, challenges experienced in this process by the researchers and the young people, and the recommended strategies to mitigate these challenges. These findings will inform the development of guidelines on involving young people in health research.

Umbrella review registration: PROSPERO CRD42021287467.

Keywords: Youth involvement, Participatory action research, Public and Patient Involvement (PPI), Health research, Adolescent health.

Background

Adolescents aged 10–24 years comprise 24% of the world's population (1). In the past two decades, there has been a global shift in attention towards the promotion of adolescent health (2, 3). The recent prioritization of adolescent health in global strategies (4, 5) is underpinned by a number of factors. First, adolescents experience a considerable proportion of the global population's disease burden, attributed to different communicable and non-communicable diseases and injuries (6, 7). Around 168 million Disability-Adjusted Life Years (DALYs) are lost to adolescents' mental health and physical health difficulties (6, 7). Second, establishing healthy behavioral patterns and minimizing exposure to risk factors among adolescents is a strong determinant for future health trajectories and can also impact the health of the next generation (3, 6). Third, the substantial improvements in maternal and child health outcomes, achieved through considerable global efforts, are at risk without adequate investments in adolescent health (3, 6, 8, 9). Lastly, improvement in adolescent health outcomes promotes productivity, academic success, future financial stability of adolescents and reduces the direct and indirect costs associated with disease burden, resulting in societal and economic benefits (3, 6, 10-13). For these reasons, adolescence is now widely considered a critical time to invest in (3, 5, 9, 14), as reflected in the increase in development assistance for adolescent health, from 109.7 million dollars in 2003 to 528.5 million dollars in 2015 (15).

Heralded by the UN convention on the Rights of the Child, the involvement of young people in all decisions that affect their lives, including health and wellbeing, is now widely acknowledged as their fundamental right (16, 17). Meaningful involvement of youth in health research is defined as "research that is done 'with' or 'by'" young people, "not 'to', 'about' or 'for' them" (18, 19). Young people can be engaged to define and prioritize research questions, design the research process,

ensure the appropriateness of the research methodology for the young population, collect and analyze data, and disseminate the research findings (17, 19-24). There are several frameworks to describe the involvement of children and adolescents in research and health services. These include Hart's ladder of young people's participation framework(25), Shier's pathways to participation framework (26), Treseder's non-linear model of participation (27), Wong's TYPE Pyramid framework (28), Arunkumar's rope ladder model (29), and the five-dimensional framework for young people's involvement in health research (19). The most commonly used frameworks to describe and evaluate youth involvement are Hart's ladder of young people's participation framework(25) and Shier's pathways to participation framework (26).

Hart's ladder of young people's participation builds on Arnstein's ladder of citizen participation for adults (30). This framework refers to eight steps in the ladder of participation: manipulation, decoration, tokenism, assigned but informed, consulted and informed, adult-initiated shared decisions with children, child-initiated and directed, and child-initiated shared decisions with adults. Meaningful engagement begins at the fourth step of the ladder and ends with shared decision-making between children and the researchers at the highest step (25). Shier proposed an alternative framework that focuses on elements of meaningful involvement. Shier's 'pathways to participation' model proposes five levels of involvement where on the first level young people are listened to, on the second level they are supported to express their views, on the third level their views are taken into account, on the fourth level they are involved in decision-making processes and on the fifth level they share power and responsibility for decision making with the researchers (26). The relative simplicity of this model makes it one of the most widely used frameworks for youth involvement (19, 31).

In recent years, there has been an increasing emphasis on the meaningful involvement of young people in health research, using participatory approaches (17, 23, 31-36). Three driving factors explain the context of this change around the involvement of young people in health research and service delivery(31). The first one is "consumer movement" (37), also equated with the term "user involvement", emerging in the 1970s, that focuses on the integration of patient's views to ensure responsive and acceptable health services (31, 38). This approach has assumed a central position in NHS health policy for adults since the late nineties (39-41) and now encompasses the involvement of young people in the design and delivery of health services and research (22, 31, 42).

Second, Article 12 of the UN Convention on Rights of the Child (16) emphasized that children and adolescents have a right to contribute to decisions regarding all matters relevant to young people and that their views must be taken into account. Almost all countries have now ratified this convention to achieve health equity by sharing power over decisions about young people (6, 43). This has led to a change in the perception of young people as social actors rather than passive recipients of care and services designed and delivered by adult professionals (31, 35). This has also resulted in an unprecedented demand for the representation of young people in health-related decision-making, with multiple health and funding organizations strongly advocating for the meaningful involvement of young people in health research to achieve the 2030 agenda for Sustainable Development (3, 17, 19, 44-46).

Third, preliminary evidence on the impact of young people's contribution in health research highlights multiple benefits of youth involvement from an operational, developmental, and societal perspective (3, 6, 17, 19, 22, 31, 47, 48), further strengthening the cause for youth involvement. From an operational perspective, the involvement of young people improves research in several ways: i) ensures that the research questions reflect the needs and preferences of young people (17,

19, 21, 49), ii) enhances the recruitment and retention rates of participants (19, 50-54), iii) improves data collection (19, 21, 55-59), iv) improves data analysis by bringing unique insight of young people in translating the responses (19, 60, 61), and v) facilitates broader and effective dissemination of the findings (6, 19, 57, 62). Organizations that work with young people report an overall change in the organization's culture to be more inclusive (63). From a developmental perspective, several positive outcomes have been reported for young people who contribute to research projects, including i) learning new research skills (19, 47, 64-69), ii) increased knowledge about health topics (19, 47, 51, 70-72), iii) better health outcomes (19, 47, 71, 73), and iv) better academic or career outcomes (6, 19, 22, 64, 74-76). While on the societal level, the involvement of young people has been linked with an increase in awareness of different health issues in the community (17, 19, 63, 77-80).

Despite growing recognition of the fundamental rights of young people to contribute to research and the potential benefits of involving young people in research, Sellars et al. (2020) (17) found that less than 1% of studies on child and adolescent health report involving young people. While involving young people in health research is on the rise, the overall number of studies that involve youth is still low (19). This lack of involvement of young people in health research may be attributed to the challenges or barriers experienced by researchers and young people contributing to research projects (17). These challenges include inadequate funding for ensuring meaningful youth involvement (81-83), the need for high-quality training of research teams and young people (81, 84-86), difficulties in recruiting and retaining young people (81, 86, 87), complex ethical procedures such as additional consent and assent requirements and compliance with different safeguard-

ing practices that vary for different contexts (49, 57, 88), and lack of awareness about the importance of youth involvement (17, 19, 48, 57, 82, 89). However, evidence is scarce on how to address these challenges or to overcome the barriers to meaningful youth involvement (90).

Aims

Given the dearth of studies reporting youth involvement, there is a need for an in-depth understanding of the challenges associated with youth involvement and the potential mitigation strategies to address these challenges. An initial scoping search identified several reviews exploring youth involvement in health research (17, 19, 47, 48, 54, 64, 91). These reviews aimed to identify the use of different methods to involve young people in health research and their level of involvement, quantify the reporting of youth involvement in health research studies, the challenges associated with youth involvement, and the best practices in youth involvement. However, these reviews focused on different aspects of youth involvement, included studies with different age groups of young people, searched different databases, had significant variations in the search terms, and restricted their search to different years and languages. For example, one of the scoping reviews on youth involvement reports the use of different youth involvement methods, evaluation methods for youth involvement, outcomes, and limitations of engaging young people but does not explore the best practices of youth involvement or the recommendations to navigate the challenges associated with youth involvement (48).

While these reviews extensively synthesize the evidence around different areas of youth involvement, there is a need to collate this review-level evidence to understand the full breadth of challenges experienced in youth involvement and the potential mitigation strategies that could be used to address these challenges. To achieve this, the proposed umbrella review aims to i) summarize and synthesize findings from reviews on involving young people in health research, ii)

consolidate the challenges experienced in youth involvement and the recommendations to mitigate these challenges, iii) identify the best practices in involving young people in health research, and iv) identify gaps and methodological weaknesses in the extant literature.

Methods

This umbrella review will be conducted as per the Cochrane guidelines for overviews of reviews (92) and will be reported in accordance with the Preferred Reporting Items for Systematic Review and Meta-Analysis (PRISMA) statement (Additional file 1) (93). The umbrella review has been registered with the International Prospective Register of Systematic Reviews (PROSPERO CRD42021287467).

Search strategy

The search strategy for this umbrella review includes keywords for; a) population (adolescents aged 10-24 years), b) intervention or exposure (involvement of young people in health research), c) condition under study (physical and mental health of adolescents), and d) article type (including narrative reviews, targeted reviews, rapid reviews, scoping reviews, literature reviews, qualitative reviews, integrated reviews, evidence maps, critical reviews, mixed methods reviews, overviews, state of the art reviews, systematic reviews, as well as meta-analyses) using Boolean syntax. Authors AW and KH developed the search strategy in consultation with a research librarian at Trinity College Dublin.

A commonly reported problem in studying youth involvement is the inconsistent use of terminologies and a wide range of methodologies classified as youth involvement (19, 48). A few other terms interchangeably used for youth involvement are given in Table 1. Our search strategy includes these terminologies in addition to some less frequently used keywords for youth

involvement (such as people with lived experience). The search strategy is attached in the supplementary materials (Additional File 2).

Table 1. Common terminologies for involving young people in research

**Terminologies**	**Definitions**
Youth involvement	"Research that is done 'with' or 'by'" young people, "not 'to', 'about' or 'for' them." (18, 19)
Community-Based Participatory Research (CBPR)	"Valuing the role of community members as equitable partners and acknowledging the importance of building partnerships with the people that ultimately are targeted by research efforts." (94)
Public and Patient Involvement (PPI)	"An active partnership between the public and researchers in the research process, rather than the use of people as 'participants' of research." (95)
Peer researcher	"Person with lived experience of the issues being studied who takes part in directing and conducting the research." (96)
Photovoice	"Participatory method that asks participants to take photos in their local communities and personal lives that are used to visualize issues and serve as the basis for discussions." (19, 55)
Young Person's Advisory Group (YPAG) or Youth Advisory Board/Council/Panel	"A method of implementing co-production with young people in health research through advisory groups that provide a forum for young people to collaborate with and support researchers." (21)
Youth-led Participatory Action Research (YPAR)	"An approach to scientific inquiry and social change grounded in principles of equity that engages young people in identifying problems relevant to their own lives, conducting research to understand the

	problems, and advocating for changes based on research evidence." (97)
Human-Centered Design (98)	"The process of integrating human perspectives in all steps of the problem-solving process. The process aims to better understand an issue from the human perspective and focuses on how it looks and feels to users and stakeholders within their environment and context. This understanding informs the iterative development of concepts designed to address a problem." (99)
Consultation	"Asking young people for their views and using these views to inform decision-making." (22)
Collaboration	"Active, on-going partnership with young people in research and the development of services." (22)
User-control	"Putting the locus of power, initiative and subsequent decision-making with young people" (22)
Engagement	"Information and knowledge about research is shared with the public." (18)

Information sources

Electronic databases

We will search Cochrane Database of Systematic Reviews, Medical Literature Analysis and Retrieval System Online (MEDLINE), Scopus, Embase, PsycINFO, PsycArticles, Cumulative Index to Nursing and Allied Health Literature (CINAHL), Epistemonikos, and Health Systems Evidence databases for potentially eligible reviews conducted up to 30th November 2021.

Grey literature search

The grey literature search involves several components. First, a simplified search strategy – based on different combinations of fewer key search terms – will be used to search Google Scholar for additional reviews. The Google Scholar search will be restricted to 10 pages. Second, we will identify the top 10 ranking pediatrics, perinatology, and child health journals using the Scimago Journal and Country Rank list for 2020 (17). A similar simplified version of the search strategy will be used to search these journals for additional reviews that could be potentially eligible for inclusion. Third, Web of Science, ProQuest, and OpenGrey will be searched to identify additional conference abstracts, theses, reports, and unpublished relevant reviews. The search strategy will be modified to suit the interface of these databases, as required. The search strategy for Web of Science is attached in the supplementary materials (Additional File 3). Fourth, we will search for relevant reviews registered on PROSPERO. Authors of potentially eligible reviews will be contacted to check whether the reviews are close to completion or have been completed. The authors will be requested to share the extracted data for inclusion in the umbrella review for complete or close to completion reviews. The number of contacts made and the number of authors who agreed to share the data will be recorded and reported. Fifth, websites of relevant organizations will also be searched for grey literature. This will involve compiling a list of organizations (including governmental, non-profit, and funding organizations) working on young people's health. We will conduct a targeted search of up to 20 pages of Google, the Mental Health Innovation Network database, and including organizations known to the authors. Then, we will search for relevant documents by running a simplified search strategy, like the one used for searching Google Scholar, on the website homepages of these organizations. The first ten pages of the results will be searched. Websites without a search option will be hand-searched. We will

also search Google for youth health organizations in low- and middle-income countries and search the website of one organization (top google result) for each country. Names of all organizational websites searched, and the titles and URL links to eligible materials will be extracted in the excel spreadsheet for grey literature, which will be posted on the review's Open Science Framework (OSF) page. Sixth, we will contact five to ten experts in youth involvement in health research. They will be regarded as an expert if, a) they oversee youth involvement in health research component at a funding organization (e.g., Wellcome Trust, Grand Challenges Canada) or youth health organization and/or, b) have conducted studies by involving adolescents as collaborators at different stages of the research process. We will include experts from high, middle, and low-income countries. After identifying the potential experts to be contacted, we will send them an email stating the aims of the umbrella review, eligibility criteria for reviews, and a statement requesting that they share the references of any relevant materials they might be familiar with. Seventh, reference lists of all eligible reviews will be reviewed to identify further relevant reviews. Finally, all the eligible reviews will be entered in the connectedpapers software to identify similar papers. This comprehensive strategy aims to identify all published and unpublished reviews on this topic and get the broadest range of views possible for inclusion in the umbrella review.

Data management

All references from electronic databases will be exported to Covidence. We will record the search date, the number of results for each database and each combination of the search terms for grey literature search. For the grey literature search, potentially relevant documents will be book-marked, and their titles and URL links will be entered in an excel spreadsheet for grey literature (100).

Eligibility Criteria

Study Design

Only review articles will be eligible for inclusion in this umbrella review. A review will be defined as "a knowledge synthesis of evidence that includes a clear research question, describes the methods used (which are reproducible) to identify and select the primary research studies, and synthesizes data from its included studies" (101, 102). Review articles of all types, including narrative reviews, targeted reviews, rapid reviews, scoping reviews, literature reviews, qualitative reviews, integrated reviews, evidence maps, critical reviews, mixed methods reviews, overviews, state of the art reviews, systematic reviews, as well as meta-analyses will be included (102).

Participants

All reviews that include primary studies including adolescents within the age range of 10 to 24 years will be eligible for inclusion. We will only include reviews that discuss youth-specific results; the age range of participants in studies included in the review can vary, but the target population of the review must include adolescents. No other restrictions will be applied based on demographic characteristics or context.

Intervention

Reviews exploring youth involvement in studies that focus on the promotion of physical and mental health or treatment of physical and mental health difficulties among young people will be eligible.

Outcomes

Reviews including one or more of the following outcomes will be eligible for inclusion; i) different types of strategies that have been used to involve adolescents in conducting health research, ii) best practices in involving young people in health research, iii) barriers to meaningful youth involvement, iv) mitigation strategies to address these barriers and, v) evidence gaps in youth involvement in health research.

Other criteria

Only reviews published in the English language will be considered for inclusion. Reviews, where data extraction tables for the primary studies are inaccessible will be excluded. As per the Cochrane guidelines for overviews of reviews (92), if a review's research objectives are broader than the current umbrella review, the subset of primary studies meeting the review's eligibility criteria will be included in this umbrella review.

Selection of studies

Search results will be imported into Covidence for the removal of duplicate references. The eligibility criteria will be discussed among researchers conducting the title and abstract screening to ensure mutual understanding of the criteria. Title and abstract screening of the studies will be undertaken by two PhD-level psychology researchers and an adolescent co-researcher. They will first conduct a pilot screening of 1% of randomly selected search results. Any discrepancies will be discussed and resolved before proceeding with title and abstract screening for the rest of the articles. AW will conduct the title and abstract screening of all articles, while co-researchers (QK) and an adolescent researcher will conduct title and abstract screening of 25% of articles to minimize the risk of bias in data extraction. Where eligibility cannot be determined based on abstract, such articles will be labeled ambiguous and reviewed in full.

AW will conduct full-text screening and extract data from all eligible articles, while QK and an adolescent researcher will conduct full-text screening and data extraction for 10% of articles. Reasons for exclusion will be recorded for all excluded articles at this stage. Any discrepancies or disagreements will be resolved through discussion among these three researchers and, if required, through discussion with a fourth researcher (KH).

Overlap in primary studies across multiple reviews can give undue weightage to studies included more than once in the synthesis of findings. It is recommended to address the risk of overlap at the inclusion, data extraction, or synthesis stage (103). We will assess the overlap in primary studies at the data extraction and synthesis stage by creating a citation matrix (92, 104) where the primary studies will be cross-linked with the reviews they are included in. The reviews will be listed in columns, and the primary studies will be added in rows, with a tick mark added under all reviews that include a primary study. The overlap will be quantified by calculating the corrected covered area measure (104) that indicates the degree of overlap. It is calculated by subtracting the number of unique studies from the total number of studies, including the overlapping studies. The result is divided by the number of unique studies, subtracted from the product of the total number of publications and the total number of reviews. The citation matrix will help us ensure that the results of primary studies included in multiple reviews are only included once (103, 104). This citation matrix will be posted on the review's OSF page.

Data extraction

Data from the included reviews will be extracted using a data extraction form designed in Covidence. The data extraction form will be piloted by researchers extracting the data, using 1% of eligible full-text articles. Inter-rater reliability between the researchers will be assessed using the kappa statistic. Researchers will proceed with data extraction after achieving inter-rater kappa

statistics from 0.8 to 1.0 during the pilot phase. Data on results will be extracted only from the included reviews, not the primary studies included in the reviews, as the current umbrella review aims to summarize the findings from different reviews instead of re-analyzing the data from primary studies in the included reviews. Data will be extracted on:

a) Characteristics of eligible reviews including review title, names and contact details of study authors, publication type (e.g., article, conference abstract, report, etc.), review type (scoping, narrative, systematic, etc.), the age range of study population in the review, the condition under study (e.g., physical health, mental health, or specific disease/disorder), aim of the review, definition of youth involvement used, search strategy, databases and grey literature sources searched, search end date, method of synthesis, and tool used to appraise the risk of bias for the primary studies.

b) Characteristics of primary studies include their eligibility criteria, the number of primary studies included in the review, the study design of primary studies, and the risk of bias results for the primary studies.

c) Findings will consist of the use of different youth involvement strategies, level of youth involvement, challenges, or barriers in the use of youth involvement strategies, recommendations to address these barriers, best practices in youth involvement in health research, limitations of the review, and gaps identified in the literature.

Risk of bias assessment

A MeaSurement Tool to Assess systematic Reviews-2 (AMSTAR 2) (105) will be used to assess the methodological quality of included reviews. The risk of bias assessments conducted for the primary studies in the included reviews will be narratively summarized. AW will conduct the risk

of bias assessment of all eligible reviews, while QK will conduct the risk of bias assessment for 10% of randomly selected eligible reviews. Any discrepancies or disagreements will be resolved through discussion among these two researchers and, if required, through discussion with a third researcher (KH).

Narrative synthesis

A narrative synthesis will be conducted to analyze the data. The first step will include familiarization with the extracted data through close reading, followed by coding the extracted data using deductive coding. Then, the codes will be structured under broader themes. Finally, these themes will be summarized in a descriptive and tabular form, centered around the research questions. AW will primarily conduct the narrative synthesis.

Youth involvement in the review

We will invite young people aged 10-24 years to participate as youth researchers and advisors at the title and abstract screening, full-text screening, data-extraction, analysis, and dissemination phases of the umbrella review. Youth advisors will be invited to participate through local non-profit organizations and/or academic institutions.

An adolescent co-researcher will be recruited to conduct title and abstract screening for 25% of the articles and undertake full-text screening and data extraction for 10% of articles to ensure the representation of youth in the decision-making process. To increase the relevance of the findings, youth advisors will be invited to share their interpretation of the results and reflections on the challenges and recommendations highlighted in the literature. Additionally, they will contribute by reviewing the outputs of this umbrella review and ensuring the language used is accessible and inclusive. We will also facilitate the young co-researcher and advisors to present the findings to

the scientific community via video abstracts and to youth and lay audiences through dissemination methods determined by the youth themselves to ensure more effective and wider dissemination of the results.

Discussion

This review will identify the most effective ways of involving adolescents in health research, challenges experienced in this process, and the recommended mitigation strategies to address or prevent these challenges. These findings will inform the development of guidelines on involving young people in health research. The need for a comprehensive set of guidelines and resources on involving young people in health research has been highlighted by several youth researchers (90) and studies (82, 106-109). These guidelines will facilitate researchers to collaborate with adolescents more effectively, leading to more meaningful involvement of young people in research.

Any deviations to the submitted protocol will be documented as amendments to the PROSPERO registration. This umbrella review has a few limitations. The search for eligible reviews will be restricted to the English language. Additionally, while the search strategy incorporates a wide range of terms to account for the variation in terminology around youth involvement, there is a possibility that relevant reviews indexed using different terms will not be included.

An initial scoping search for relevant reviews highlighted a scarcity of literature on the recommendations or guidelines to address the barriers to meaningful youth involvement (19, 106). If this evidence gap is also highlighted in the findings of this umbrella review, we will plan a Delphi study to establish expert consensus on the mitigation strategies to address the barriers to youth involvement and to identify the best practices in involving young people in health research.

Abbreviations

DALYS**:** Disability-Adjusted Life Years; UN: United Nations; NHS: National Health Service; PRISMA-P: Preferred Reporting Items for Systematic Review and Meta-Analysis Protocols; MEDLINE: Medical Literature Analysis and Retrieval System Online; CINAHL: Cumulative Index to Nursing and Allied Health Literature; CBPR: Community-Based Participatory Research; PPI: Patient and Public Involvement; YPAG: Young Person's Advisory Group; YPAR: Youth-led Participatory Action Research; AMSTAR 2: A MeaSurement Tool to Assess systematic Reviews-2; OSF: Open Science Framework.

Supplementary Information

Additional file 1. PRISMA-P Checklist.

Additional file 2. Search strategy for MEDLINE

Additional file 3. Search strategy for Web of Science

Declarations

Ethics approval and consent to participate

Not applicable

Consent for publication

Not applicable

Availability of data and materials

Data sharing does not apply to this article as no datasets will be generated or analyzed during the current study. Tables generated for the analysis will be shared as supplementary materials with the umbrella review outcome paper and the review's OSF page.

Competing interests

The authors declare that they have no competing interests.

Funding

Azza Warraitch received the Ussher Fellowship from Trinity College Dublin to support her Ph.D. research, including this umbrella review. The funder did not play any role in design of this study.

Authors' contributions

AW and KH conceptualized and wrote the protocol with valuable inputs from QK. All authors have read and approved the final manuscript.

Acknowledgments

We would like to acknowledge all the members at the Trinity Centre for Global Health, especially Dr. Sadhbh Byrne, for their valuable inputs on involving young people at different stages of the review. We would also like to thank Ms. Geraldine Fitzgerald, the research librarian at the School of Medicine, Trinity College Dublin, for her feedback and input on the search strategy for this umbrella review.

References

1. Nations U. Department of Economic and Social Affairs, Population Division. World Population Prospects 2019: Data Booklet (ST/ESA/SER. A/424).

2. Dick B, Ferguson BJ. Health for the world's adolescents: a second chance in the second decade. Journal of Adolescent Health. 2015;56(1):3-6.

3. Patton GC, Sawyer SM, Santelli JS, Ross DA, Afifi R, Allen NB, et al. Our future: a Lancet commission on adolescent health and wellbeing. The Lancet. 2016;387(10036):2423-78.

4. Kuruvilla S, Bustreo F, Kuo T, Mishra C, Taylor K, Fogstad H, et al. The Global strategy for women's, children's and adolescents' health (2016–2030): a roadmap based on evidence and country experience. Bulletin of the World Health Organization. 2016;94(5):398.

5. Temmerman M, Khosla R, Bhutta ZA, Bustreo F. Towards a new global strategy for women's, children's and adolescents' health. bmj. 2015;351.

6. Organization WH. Global accelerated action for the health of adolescents (AA-HA!): guidance to support country implementation. 2017.

7. WHO. Global health estimates 2015: DALYs by cause, age, sex, by country and by region, 2000-2015 Geneva. 2016 [Available from: http://www.who.int/healthinfo/global_burden_disease/estimates/en/index2.html.

8. Resnick MD, Catalano RF, Sawyer SM, Viner R, Patton GC. Seizing the opportunities of adolescent health. The Lancet. 2012;379(9826):1564-7.

9. Sawyer SM, Afifi RA, Bearinger LH, Blakemore S-J, Dick B, Ezeh AC, et al. Adolescence: a foundation for future health. The lancet. 2012;379(9826):1630-40.

10. Gribble JN, Bremner J. The challenge of attaining the demographic dividend: Population Reference Bureau; 2012.

11. Deogan C, Ferguson J, Stenberg K. Resource needs for adolescent friendly health services: estimates for 74 low-and middle-income countries. PLoS One. 2012;7(12):e51420.

12. Stenberg K, Axelson H, Sheehan P, Anderson I, Gülmezoglu AM, Temmerman M, et al. Advancing social and economic development by investing in women's and children's health: a new Global Investment Framework. The Lancet. 2014;383(9925):1333-54.

13. Curtain R. The case for investing in young people as part of a national poverty reduction strategy. Paper commissioned by the United Nations Population Fund, New York. 2004.

14. Salam RA, Das JK, Lassi ZS, Bhutta ZA. Adolescent health and well-being: Background and methodology for review of potential interventions. Journal of adolescent health. 2016;59(4):S4-S10.

15. Li Z, Li M, Patton GC, Lu C. Global development assistance for adolescent health from 2003 to 2015. JAMA network open. 2018;1(4):e181072-e.

16. Unicef. Convention on the Rights of the Child. 1989.

17. Sellars E, Pavarini G, Michelson D, Creswell C, Fazel M. Young people's advisory groups in health research: scoping review and mapping of practices. Archives of Disease in Childhood. 2021;106(7):698-704.

18. Authority NHR. What is public involvement in research? 2020 [Available from: https://www.hra. nhs.uk/planning-and-improving-research/best-practice/public-involvement/.

19. Wilson O, Daxenberger L, Dieudonne L, Eustace J, Hanard A, Krishnamurthi A, et al. A rapid evidence review of young people's involvement in health research. London: Wellcome. 2020:3.

20. Oliveras C, Cluver L, Bernays S, Armstrong A. Nothing about us without RIGHTS—meaningful engagement of children and youth: from research prioritization to clinical trials,

implementation science, and policy. Journal of acquired immune deficiency syndromes (1999). 2018;78(1):S27.

21. Pavarini G, Lorimer J, Manzini A, Goundrey-Smith E, Singh I. Co-producing research with youth: The NeurOx young people's advisory group model. Health Expectations. 2019;22(4):743-51.

22. Kirby P. A guide to actively involving young people in research. For Researchers, Research Commissioners and Managers, Involve Support Unit, Eastleigh, Hamphires. 2004.

23. Ozer EJ, Piatt AA. Adolescent Participation in Research: Innovation, rationale and next steps. 2017.

24. NIHR. Involving children and young people as advisors in research. Top tips and essential key issues for researchers. 2021.

25. Hart RA. Children's participation: From tokenism to citizenship. 1992.

26. Shier H. Pathways to participation: Openings, opportunities and obligations. Children & society. 2001;15(2):107-17.

27. Fund StC, Treseder P. Empowering Children and Young People: A Training Manual: Promoting Involvement in Decision-making: Save the Children Fund; 1997.

28. Wong NT, Zimmerman MA, Parker EA. A typology of youth participation and empowerment for child and adolescent health promotion. American journal of community psychology. 2010;46(1):100-14.

29. Arunkumar K, Bowman DD, Coen SE, El-Bagdady MA, Ergler CR, Gilliland JA, et al. Conceptualizing youth participation in children’s health research: insights from a youth-driven process for developing a youth advisory council. Children. 2019;6(1):3.

30. Arnstein S. A ladder of citizen participation. J Am Inst Planners 35 (4): 216-224. 1969.

31. Gibson F. Building a culture of participation: young people's involvement in research. Cancer Care for Adolescents and Young Adults Oxford: Blackwell Publishing. 2008:214-28.

32. Clarke S. A "Child's rights perspective": the "right" of children and young people to participate in health care research. Issues in comprehensive pediatric nursing. 2015;38(3):161-80.

33. Daly W. "Adding their flavour to the mix": involving children and young people in care in research design. Australian Social Work. 2009;62(4):460-75.

34. Nygren JM, Lindberg S, Wärnestål P, Svedberg P. Involving children with cancer in health promotive research: a case study describing why, what, and how. JMIR research protocols. 2017;6(2):e7094.

35. Kellett M. Children as active researchers: a new paradigm for the 21st century. National Centre for Research Methods Review Papers NCRM/003, ESRC National Centre for Research Methods. 2005.

36. Jacquez F, Vaughn LM, Wagner E. Youth as partners, participants or passive recipients: A review of children and adolescents in community-based participatory research (CBPR). American journal of community psychology. 2013;51(1-2):176-89.

37. Telford R, Boote JD, Cooper CL. What does it mean to involve consumers successfully in NHS research? A consensus study. Health Expectations. 2004;7(3):209-20.

38. Health Do. Strengthening Accountability: Involving Patients and the Public. Policy Guidance, Section 11 of the Health and Social Care Act 2001. Department of Health London; 2001.

39. Smith E, Ross F, Donovan S, Manthorpe J, Brearley S, Sitzia J, et al. User involvement in the design and undertaking of nursing, midwifery and health visiting research. Nursing Research Unit, King's College London. 2005.

40. Hanley B, Bradburn J, Gorin S, Barnes M, Evans C, Goodare H, et al. Involving consumers in research & development in the NHS: briefing notes for researchers: Consumers in NHS Research Support Unit Winchester; 2000.

41. Health Do. Patient and public involvement in the new NHS: The Stationery Office London; 1999.

42. Health GBDo. Listening, hearing and responding Department of Health Action Plan: core principles for the involvement of children and young people. 2002.

43. Watch HR. 25th anniversary of the convention on the rights of the Child. 2017.

44. Partnership for Maternal NaCHIYAfFP, and Family Planning. Global consensus statement on meaningful adolescent and youth engagement. 2020 [Available from: https://www.who.int/pmnch/mye-statement.pdf?ua=1.

45. Bulc B, Al-Wahdani B, Bustreo F, Choonara S, Demaio A, Jácome DI, et al. Urgency for transformation: youth engagement in global health. The Lancet Global Health. 2019;7(7):e839-e40.

46. Nations U. Youth2030: The United Nations Strategy on Youth 2018 [Available from: https://www.un.org/youthenvoy/youth-un/.

47. Valdez ES, Skobic I, Valdez L, O Garcia D, Korchmaros J, Stevens S, et al. Youth participatory action research for youth substance use prevention: a systematic review. Substance use & misuse. 2020;55(2):314-28.

48. Rouncefield-Swales A, Harris J, Carter B, Bray L, Bewley T, Martin R. Children and young people's contributions to public involvement and engagement activities in health-related research: A scoping review. PloS one. 2021;16(6):e0252774.

49. Denison JA, Pettifor A, Mofenson LM, Kasedde S, Marcus R, Konayuma KJ, et al. Youth engagement in developing an implementation science research agenda on adolescent HIV testing and care linkages in sub-Saharan Africa. AIDS (London, England). 2017;31(Suppl 3):S195.

50. Gaillard S, Malik S, Preston J, Escalera BN, Dicks P, Touil N, et al. Involving children and young people in clinical research through the forum of a European Young Persons' Advisory Group: needs and challenges. Fundamental & clinical pharmacology. 2018;32(4):357-62.

51. Kulbok PA, Meszaros PS, Bond DC, Thatcher E, Park E, Kimbrell M, et al. Youths as partners in a community participatory project for substance use prevention. Family & community health. 2015;38(1):3-11.

52. Tsang VW, West L, Woods C, Koh CJ, McCune S, Mullin T, et al. Role of patients and parents in pediatric drug development. Therapeutic innovation & regulatory science. 2019;53(5):601-8.

53. Comfort M, Raymond-Flesch M, Auerswald C, McGlone L, Chavez M, Minnis A. Community-engaged research with rural Latino adolescents: design and implementation strategies to study the social determinants of health. Gateways: international journal of community research & engagement. 2018;11(1):90.

54. Vaughn LM, Wagner E, Jacquez F. A review of community-based participatory research in child health. MCN: The American journal of maternal/child nursing. 2013;38(1):48-53.

55. Valdez ES, Korchmaros J, Sabo S, Garcia DO, Carvajal S, Stevens S. How the US-Mexico border influences adolescent substance use: Youth participatory action research using photovoice. International Journal of Drug Policy. 2019;73:146-55.

56. Jardine CG, James A. Youth researching youth: Benefits, limitations and ethical considerations within a participatory research process. International Journal of Circumpolar Health. 2012;71(1):18415.

57. Bird D, Culley L, Lakhanpaul M. Why collaborate with children in health research: an analysis of the risks and benefits of collaboration with children. Archives of Disease in Childhood-Education and Practice. 2013;98(2):42-8.

58. Kelly B, Friel S, McShane T, Pinkerton J, Gilligan E. "I haven't read it, I've lived it!" The benefits and challenges of peer research with young people leaving care. Qualitative Social Work. 2020;19(1):108-24.

59. Panter-Brick C, Eggerman M, Ager A, Hadfield K, Dajani R. Measuring the psychosocial, biological, and cognitive signatures of profound stress in humanitarian settings: impacts, challenges, and strategies in the field. Conflict and health. 2020;14(1):1-7.

60. Delman J. Participatory Action Research and young adults with psychiatric disabilities. Psychiatric Rehabilitation Journal. 2012;35(3):231.

61. Chappell P, Rule P, Dlamini M, Nkala N. Troubling power dynamics: youth with disabilities as co-researchers in sexuality research in South Africa. Childhood. 2014;21(3):385-99.

62. Mitchell K, Durante SE, Pellatt K, Richardson CG, Mathias S, Buxton JA. Naloxone and the Inner City Youth Experience (NICYE): a community-based participatory research study examining young people's perceptions of the BC take home naloxone program. Harm reduction journal. 2017;14(1):1-8.

63. Shamrova DP, Cummings CE. Participatory action research (PAR) with children and youth: An integrative review of methodology and PAR outcomes for participants, organizations, and communities. Children and Youth Services Review. 2017;81:400-12.

64. Anyon Y, Bender K, Kennedy H, Dechants J. A systematic review of youth participatory action research (YPAR) in the United States: Methodologies, youth outcomes, and future directions. Health Education & Behavior. 2018;45(6):865-78.

65. BAILEy S, BODDy K, Briscoe S, Morris C. Involving disabled children and young people as partners in research: a systematic review. Child: care, health and development. 2015;41(4):505-14.

66. Brosnan CA, Upchurch SL, Meininger JC, Hester LE, Johnson G, Eissa MA. Student nurses participate in public health research and practice through a school-based screening program. Public Health Nursing. 2005;22(3):260-6.

67. Cepanec D, Clarke D, Plohman J, Gerard J. Engaging undergraduate nursing students in research: The students' experience of a summer internship program pilot project. Journal of Nursing Education. 2013;52(8):466-9.

68. Suleiman AB, Soleimanpour S, London J. Youth action for health through youth-led research. Journal of Community Practice. 2006;14(1-2):125-45.

69. Lindquist-Grantz R, Abraczinskas M. Using youth participatory action research as a health intervention in community settings. Health promotion practice. 2020;21(4):573-81.

70. Abma T, Lips S, Schrijver J. Sowing seeds to harvest healthier adults: The working principles and impact of participatory health research with children in a primary school context. International journal of environmental research and public health. 2020;17(2):451.

71. Aceves-Martins M, Aleman-Diaz AY, Giralt M, Solà R. Involving young people in health promotion, research and policy-making: practical recommendations. International Journal for Quality in Health Care. 2019;31(2):147-53.

72. Lane HG, Porter KJ, Hecht E, Harris P, Zoellner JM. A participatory process to engage Appalachian youth in reducing sugar-sweetened beverage consumption. Health promotion practice. 2019;20(2):258-68.

73. Sherman SG, Sutcliffe C, Srirojn B, Latkin CA, Aramratanna A, Celentano DD. Evaluation of a peer network intervention trial among young methamphetamine users in Chiang Mai, Thailand. Social science & medicine. 2009;68(1):69-79.

74. Mance GA, Mendelson T, Byrd III B, Jones J, Tandon D. Utilizing community-based participatory research to adapt a mental health intervention for African American emerging adults. Progress in community health partnerships: research, education, and action. 2010;4(2):131-40.

75. Stewart K-A, Brown SL, Wrensford G, Hurley MM. Creating a comprehensive approach to exposing underrepresented pre-health professions students to clinical medicine and health research. Journal of the National Medical Association. 2020;112(1):36-43.

76. Sheridan R, Preston J, Stones S, Ainsworth S, Horton-Taylor D, Challinor R, et al. Patient and public involvement in a study of multimedia clinical trial information for children, young people and families. Research for All. 2020:47-65.

77. Davies A, Mwangome N, Yeri B, Mwango G, Mumba N, Marsh V, et al. Evolution of a programme to engage school students with health research and science in Kenya. Wellcome open research. 2019;4.

78. Dennis Jr SF, Gaulocher S, Carpiano RM, Brown D. Participatory photo mapping (PPM): Exploring an integrated method for health and place research with young people. Health & place. 2009;15(2):466-73.

79. Findholt NE, Michael YL, Davis MM. Photovoice engages rural youth in childhood obesity prevention. Public Health Nursing. 2011;28(2):186-92.

80. Masuku B, Mkhwanazi N, Young E, Koch A, Warner D. Beyond the lab: Eh! woza and knowing tuberculosis. Medical humanities. 2018;44(4):285-92.

81. Hawke LD, Darnay K, Relihan J, Khaleghi-Moghaddam M, Barbic S, Lachance L, et al. Enhancing researcher capacity to engage youth in research: Researchers' engagement experiences, barriers and capacity development priorities. Health Expectations. 2020;23(3):584-92.

82. Wadman R, Williams AJ, Brown K, Nielsen E. Supported and valued? A survey of early career researchers' experiences and perceptions of youth and adult involvement in mental health, self-harm and suicide research. Research involvement and engagement. 2019;5(1):1-12.

83. James RD, McGlone West K, Madrid TM. Launching native health leaders: Reducing mistrust of research through student peer mentorship. American journal of public health. 2013;103(12):2215-9.

84. Bradbury-Jones C, Taylor J. Engaging with children as co-researchers: challenges, counter-challenges and solutions. International Journal of Social Research Methodology. 2015;18(2):161-73.

85. Franklin A, Sloper P. Listening and responding? Childrens participation in health care within England. Children's Health and Children's Rights: Brill Nijhoff; 2006. p. 11-29.

86. McCartan C, Burns S, Schubotz D. Recruitment and capacity-building challenges in participatory research involving young people in Northern Ireland. Community Research for Participation: From theory to method. 2012:283.

87. Peek L, Tobin-Gurley J, Cox RS, Scannell L, Fletcher S, Heykoop C. Engaging youth in post-disaster research: Lessons learned from a creative methods approach. Gateways: International Journal of Community Research and Engagement. 2016;9(1):89-112.

88. Huang X, O'Connor M, Ke L-S, Lee S. Ethical and methodological issues in qualitative health research involving children: A systematic review. Nursing ethics. 2016;23(3):339-56.

89. Kendal SE, Milnes L, Welsby H, Pryjmachuk S, Group CR, Shafeeah C, et al. Prioritizing young people's emotional health support needs via participatory research. Journal of psychiatric and mental health nursing. 2017;24(5):263-71.

90. Das S, Daxenberger L, Dieudonne L, Eustace J, Hanard A, Krishnamurthi A, et al. Stakeholder consultation on involving young people in health research. London: Wellcome. 2020:3.

91. Orlowski SK, Lawn S, Venning A, Winsall M, Jones GM, Wyld K, et al. Participatory research as one piece of the puzzle: a systematic review of consumer involvement in design of technology-based youth mental health and well-being interventions. JMIR human factors. 2015;2(2):e4361.

92. Pollock M, Fernandes RM, Becker LA, Pieper D, Hartling L. Chapter V: overviews of reviews. Cochrane Handbook for systematic reviews of interventions version. 2018;6.

93. Shamseer L, Moher D, Clarke M, Ghersi D, Liberati A, Petticrew M, et al. Preferred reporting items for systematic review and meta-analysis protocols (PRISMA-P) 2015: elaboration and explanation. Bmj. 2015;349.

94. Israel BA, Schulz AJ, Parker EA, Becker AB. Review of community-based research: assessing partnership approaches to improve public health. Annual review of public health. 1998;19(1):173-202.

95. Boote J. Patient and public involvement in health and social care research: a bibliography. NIHR Research Design Service for Yorkshire and the Humber. 2011.

96. Lushey CJ, Munro ER. Participatory peer research methodology: An effective method for obtaining young people's perspectives on transitions from care to adulthood? Qualitative Social Work. 2015;14(4):522-37.

97. Ozer EJ. Youth-led participatory action research: Developmental and equity perspectives. Advances in child development and behavior. 2016;50:189-207.

98. Cooley M. Human-centered design. Information design. 2000:59-81.

99. Health Df. Glossary of Design Terms2020. 38 p.

100. Godin K, Stapleton J, Kirkpatrick SI, Hanning RM, Leatherdale ST. Applying systematic review search methods to the grey literature: a case study examining guidelines for school-based breakfast programs in Canada. Systematic reviews. 2015;4(1):1-10.

101. Graham-Kevan N, Archer J. Investigating three explanations of women's relationship aggression. Psychology of Women Quarterly. 2005;29(3):270-7.

102. Grant MJ, Booth A. A typology of reviews: an analysis of 14 review types and associated methodologies. Health information & libraries journal. 2009;26(2):91-108.

103. Lunny C, Pieper D, Thabet P, Kanji S. Managing Overlap of Primary Studies Results Across Systematic Reviews: Practical Considerations for Authors of Overviews of Reviews. 2021.

104. Pieper D, Antoine S-L, Mathes T, Neugebauer EA, Eikermann M. Systematic review finds overlapping reviews were not mentioned in every other overview. Journal of clinical epidemiology. 2014;67(4):368-75.

105. Shea BJ, Reeves BC, Wells G, Thuku M, Hamel C, Moran J, et al. AMSTAR 2: a critical appraisal tool for systematic reviews that include randomised or non-randomised studies of healthcare interventions, or both. bmj. 2017;358.

106. Flynn R, Walton S, Scott SD. Engaging children and families in pediatric health research: a scoping review. Research involvement and engagement. 2019;5(1):1-8.

107. Faithfull S, Brophy L, Pennell K, Simmons MB. Barriers and enablers to meaningful youth participation in mental health research: qualitative interviews with youth mental health researchers. Journal of Mental Health. 2019;28(1):56-63.

108. Postma L, Luchtenberg ML, Verhagen AE, Maeckelberghe EL. The attitudes of healthy children and researchers towards the challenges of involving children in research: an exploratory study. Research involvement and engagement. 2021;7(1):1-10.

109. Chan W, Thurairajah P, Butcher N, Oosterwijk C, Wever K, Eichler I, et al. Guidance on development and operation of Young Persons' Advisory Groups. Archives of disease in childhood. 2020;105(9):875-80.
