# Supplementary material for: An umbrella review of reviews on challenges to meaningful adolescent involvement in health research
Source: Health Expect. 2024 Jan 27;27(1):e13980. doi: 10.1111/hex.13980 (PMC10821743; doi:10.1111/hex.13980)
Supplement: Supplementary file 1 — Supporting information. [file HEX-27-e13980-s001.zip › PROSPERO registration and protocol paper draft/CRD42021287467 - 24 June 2022.pdf]

To enable PROSPERO to focus on COVID-19 submissions, this registration record has undergone basic automated checks for eligibility and is published exactly as submitted. It has since been amended by the author and the PROSPERO team have checked the record for eligibility. PROSPERO has never provided peer review, and usual checking by the PROSPERO team does not endorse content. Therefore, automatically published records should be treated as any other PROSPERO registration. Further detail is provided here.

Review methods were amended after registration. Please see the revision notes and previous versions for detail.

Citation

Azza Warraitch, Qusai Khraisha, Kristin Hadfield. Involving adolescents in the design, implementation, evaluation, and dissemination of health research: an umbrella review. PROSPERO 2021 CRD42021287467 Available from: https://www.crd.york.ac.uk/prospero/display_record.php?ID=CRD42021287467

Review question

An umbrella review of reviews will be conducted to consolidate the evidence and to identify the gaps in literature on youth involvement in health research. Our research questions are:

• What are the different youth involvement strategies that are being used in health research for adolescents?

• What are the best practices for youth involvement in health research?

• What are the challenges associated with different youth involvement strategies? What strategies have been used to mitigate these challenges?

Searches [2 changes]

We will search Cochrane Database of Systematic Reviews, Medical Literature Analysis and Retrieval System Online (MEDLINE), Scopus, Embase, PsycINFO, PsycArticles, Cumulative Index to Nursing and Allied Health Literature (CINAHL), Epistemonikos, and Health Systems Evidence databases for eligible reviews conducted till 30th November 2021. No restrictions on year will be applied. The search strategy will include a combination of the terms for a) condition (health), b) age range (youth aged 10-24 years), c) Intervention (youth involvement), and d) article type (review) using Boolean syntax. The search strategy will be reviewed and refined in consultation with a research librarian.

We will run a simplified version of the search strategy in Google Scholar to identify additional reviews, restricting the search up to 10 pages. We will identify child and adolescent health journals from the Journal Citation Reports (JCR) list. A similar simplified version of the search strategy will be used to search these journals. We will search three databases for grey literature: Web of Science, ProQuest, and OpenGrey. Authors of potentially eligible reviews registered on PROSPERO that are close to completion or have been completed will be contacted to request the dataset. Websites of relevant organizations will also be searched for grey literature. We will also identify 6-10 researchers considered experts in the field of youth engagement in health research, requesting them to share the references of any relevant materials they might be familiar with. The reference lists of eligible reviews will also be reviewed to identify further relevant reviews. Lastly, all the eligible reviews will be entered in the connected papers software to identify similar papers.

Search strategy

https://www.crd.york.ac.uk/PROSPEROFILES/287467_STRATEGY_20211026.pdf

Types of study to be included [2 changes]

Inclusion criteria:

a) review articles, including narrative reviews, targeted reviews, rapid reviews, scoping reviews, literature reviews, qualitative reviews, integrated reviews, evidence maps, critical reviews, literature reviews, mixed methods reviews, overviews, state of the art reviews, systematic reviews, as well as meta-analyses, which

b) aim to explore the involvement of adolescents in the design, implementation and/or evaluation of health research, including physical and mental health, and

c) where the full text of the review is available in English.

Exclusion criteria:

a) Unavailability or lack of access to data extraction tables for the primary studies included in the review

b) Protocol papers of reviews

Condition or domain being studied

Health of adolescents, including physical and mental health, is the condition being studied in this review.

Participants/population

Adolescents, aged 10-24 years, are the population of interest for this study.

Intervention(s), exposure(s)

Youth involvement in health research, defined as “research that is done ‘with’ or ‘by’” young people, “not ‘to’, ‘about’ or ‘for’ them. It means that young people contribute to tasks like defining research agendas, designing research, collecting and analyzing data, or disseminating and translating findings” (Wilson et al., 2020). It can range from getting advice or input from young people at one stage of the research process to empowering youth to lead the research projects (Sellars et al., 2020). Youth involvement is an umbrella term that includes different types of methods used to involve young people in research, including conducting workshops or focus groups with them, forming a youth advisory panel for collaboration (Sellars et al., 2020), hiring young people as co-researchers, and using the Human-Centered Design or User-Centered Design that puts the needs and preferences of the target population at the centre of all procedures (Cooley, 2000) etc.

Comparator(s)/control

Not applicable.

Context

Research conducted in any context will be included if it meets the eligibility criteria.

Main outcome(s)

Our outcomes are descriptive in nature and include:

a) Use of different youth involvement strategies: We will consolidate a list of different types of strategies that have been used to involve adolescents in conducting health research (e.g., human-centered design, youth advisory panels, photovoice etc), including their use at different stages of the research process, level of involvement of adolescents and the rationale for using different types of youth involvement strategies.

b) Best practices in youth involvement: This is defined as the most effective methods of involving adolescents in the design, implementation and evaluation of health interventions, including factors that shed light on which youth involvement strategies work, for whom (specific context and population), and why?

c) Barriers to meaningful youth involvement and mitigation strategies: We will explore the challenges experienced in engaging adolescents in health research, from the perspective of researchers working on youth health and adolescents contributing to health research, along with mitigation strategies to address these challenges.

d) Evidence gaps in youth involvement in health research: After consolidating the outcomes listed above from eligible reviews, we will go through the findings to identify the gaps or what is not known about ensuring

meaningful involvement of adolescents in health research.

Measures of effect

We will not conduct meta-analysis as all our outcomes listed above are descriptive in nature and include summarization and description of themes, based on data extracted from individual reviews. Data will not be extracted on any quantitative measurements, so a meta-analysis is not planned.

Additional outcome(s)

None

Measures of effect

None

Data extraction (selection and coding) [1 change]

A data extraction form will be designed in Covidence, to extract data that informs the research objectives and outcomes of interest. Data will be extracted as per Cochrane guidelines for overviews of reviews (Pollock et al., 2021) on; a) characteristics of systematic reviews (and included primary studies), b) search strategies used by different reviews and c) outcome data.

The data extraction form will include fields for:

a) Review title, b) names and contact details of study authors, c) publication type (e.g. article, conference abstract, report etc), d) review type (scoping, narrative, systematic review etc), age range of study population in the review, e) condition under study (e.g. physical health, mental health or specific disease/disorder), d) aim of the review, e) definition of youth engagement used, f) search strategy, g) databases and grey literature sources searched, h) search end date, i) inclusion criteria/exclusion criteria for primary studies, j) findings of the review on the use of youth engagement strategies (use of different youth engagement strategies, effect on health and implementation outcomes, challenges or barriers in the use of youth engagement strategies, recommendations to address these barriers, best practices in youth engagement in health research), l) limitations of the review, and m) gaps identified in the literature.

All references will be exported to Covidence. After removing duplicates, title and abstract screening of the studies will be conducted in Covidence by two researchers. AW will conduct the title and abstract screening of all articles while QK and a young co-researcher will conduct title and abstract screening of 25% of randomly selected articles to minimize the risk of bias in data extraction. Similarly, AW will conduct full-text screening and will extract data from all eligible articles, while QK will conduct full-text screening and data extraction for 10% of randomly selected articles.

Risk of bias (quality) assessment

We will use A MeaSurement Tool to Assess systematic Reviews-2 (AMSTAR 2) (Shea et al., 2017) to assess methodological quality of included reviews. AMSTAR 2 is a tool used to assess risk of bias in reviews that include studies with different designs. Risk of bias assessments conducted for the primary studies in the included reviews will be narratively summarized. Risk of bias assessment of all eligible reviews will be conducted by AW, while QK will conduct the risk of bias assessment of 10% of randomly selected eligible reviews. Any discrepancies or disagreements will be resolved through discussion among the two researchers and then, if, required, through discussion with a third researcher (KH).

Strategy for data synthesis

A narrative synthesis will be conducted to synthesize and analyze the findings. The first step will include familiarization with the extracted data through close reading, followed by coding the extracted data using deductive coding. Then, the codes will be structured under broader themes. Finally, these themes will be summarized in a descriptive and tabular form, centered around the research questions.

Analysis of subgroups or subsets

None

Contact details for further information

Azza Warraitch
warraita@tcd.ie

Organisational affiliation of the review [1 change]

Trinity Centre for Global Health, Trinity College Dublin, Dublin, Ireland.
School of Psychology, Trinity College Dublin, Dublin, Ireland.
https://www.tcd.ie/medicine/global-health/

Review team members and their organisational affiliations [1 change]

Ms Azza Warraitch. Trinity Centre for Global Health, Trinity College Dublin, Dublin, Ireland.
Mr Qusai Khraisha. Trinity Centre for Global Health, Trinity College Dublin, Dublin, Ireland.
Dr Kristin Hadfield. Trinity Centre for Global Health, Trinity College Dublin, Dublin, Ireland.

Type and method of review

Narrative synthesis, Review of reviews, Systematic review

Anticipated or actual start date

30 November 2021

Anticipated completion date

01 September 2022

Funding sources/sponsors

Azza Warraitch received the Ussher Fellowship from Trinity College Dublin to support her PhD research, including this umbrella review.

Grant number(s)

State the funder, grant or award number and the date of award

Ussher Fellowship, Trinity College Dublin September 2021

Conflicts of interest

Language

English

Country

Ireland

Stage of review

Review Ongoing

Subject index terms status

Subject indexing assigned by CRD

Subject index terms

Adolescent; Humans

Date of registration in PROSPERO

26 November 2021

Date of first submission

26 October 2021

Stage of review at time of this submission

Preliminary searches	Yes	No
Piloting of the study selection process	No	No
Formal screening of search results against eligibility criteria	No	No
Data extraction	No	No
Risk of bias (quality) assessment	No	No
Data analysis	No	No

Revision note

We identified a minor error in the databases listed and made this update to correct the error before starting the review.

The record owner confirms that the information they have supplied for this submission is accurate and complete and they understand that deliberate provision of inaccurate information or omission of data may be construed as scientific misconduct.
The record owner confirms that they will update the status of the review when it is completed and will add publication details in due course.

Versions

26 November 2021
26 November 2021
21 February 2022
