# Supplementary material for: An umbrella review of reviews on challenges to meaningful adolescent involvement in health research
Source: Health Expect. 2024 Jan 27;27(1):e13980. doi: 10.1111/hex.13980 (PMC10821743; doi:10.1111/hex.13980)
Supplement: Supplementary file 1 — Supporting information. [file HEX-27-e13980-s001.zip › Participatory workshops/Workshop 1/Agenda.docx]

**Youth involvement in health research workshop 1**

**December 14th, 2022 12:30 PM - 2:00 PM**

**Hosts:** Ciara Wacker, Joshua Hernon**,** Azza Warraitch

**Venue** Trinity Centre for Global Health and Zoom meeting

**Time Topic**

12:30- 12:50 PM 1. Welcome, introductions, icebreakers and refreshments

12:50– 1:00 PM 2. Overview of youth involvement in health research, the review of the literature and the outline of the activities in the participatory workshop

1:00– 1:25 PM 3. Breaking out in groups to create personas of young people who participate in health research projects and using these personas and worksheets to discuss the challenges experienced by young people in health research projects and potential mitigation strategies

1:25– 1:50 PM 4. Developing a storyboard/ river of life map with all young people to merge

the challenges and mitigation strategies shared by young people and to

map these onto the challenges and mitigation strategies in the literature

1:50- 2:00 PM 5. Summarising the findings and sharing the details of the next workshop
