# Supplementary material for: An umbrella review of reviews on challenges to meaningful adolescent involvement in health research
Source: Health Expect. 2024 Jan 27;27(1):e13980. doi: 10.1111/hex.13980 (PMC10821743; doi:10.1111/hex.13980)
Supplement: Supplementary file 1 — Supporting information. [file HEX-27-e13980-s001.zip › Search record and results/Academic databases and search engines/APA PsycInfo and PsycArticles/APA PsycInfo and PsycArticles.docx]

**Database: APA PsychInfo**

**Date of search: 30 November 2021**

| **#** | **Query** | **Limiters/Expanders** | **Last Run Via** | **Results** |
| --- | --- | --- | --- | --- |
| S16 | S13 OR S14 OR S15 | Expanders - Apply equivalent subjects Search modes - Boolean/Phrase | Interface - EBSCOhost Research Databases Search Screen - Advanced Search Database - APA PsycInfo;APA PsycArticles | 930 |
| S15 | S10 AND S11 | Expanders - Apply equivalent subjects Narrow by Methodology: - meta analysis Narrow by Methodology: - systematic review Narrow by Methodology: - literature review Search modes - Boolean/Phrase | Interface - EBSCOhost Research Databases Search Screen - Advanced Search Database - APA PsycInfo;APA PsycArticles | 42 |
| S14 | S10 AND S11 | Expanders - Apply equivalent subjects Narrow by Methodology: - systematic review Narrow by Methodology: - literature review Search modes - Boolean/Phrase | Interface - EBSCOhost Research Databases Search Screen - Advanced Search Database - APA PsycInfo;APA PsycArticles | 375 |
| S13 | S10 AND S11 | Expanders - Apply equivalent subjects Narrow by Methodology: - literature review Search modes - Boolean/Phrase | Interface - EBSCOhost Research Databases Search Screen - Advanced Search Database - APA PsycInfo;APA PsycArticles | 927 |
| S12 | S10 AND S11 | Expanders - Apply equivalent subjects Search modes - Boolean/Phrase | Interface - EBSCOhost Research Databases Search Screen - Advanced Search Database - APA PsycInfo;APA PsycArticles | 2,773 |
| S11 | TI review OR AB review | Expanders - Apply equivalent subjects Search modes - Boolean/Phrase | Interface - EBSCOhost Research Databases Search Screen - Advanced Search Database - APA PsycInfo;APA PsycArticles | 496,292 |
| S10 | S7 AND S8 AND S9 | Expanders - Apply equivalent subjects Search modes - Boolean/Phrase | Interface - EBSCOhost Research Databases Search Screen - Advanced Search Database - APA PsycInfo;APA PsycArticles | 20,773 |
| S9 | TI ( child* OR youth OR adolescen* OR "young people" OR "Young person*" OR "Young adult*" OR teen* OR juven* ) OR AB ( child* OR youth OR adolescen* OR "young people" OR "Young person*" OR "Young adult*" OR teen* OR juven* ) | Expanders - Apply equivalent subjects Search modes - Boolean/Phrase | Interface - EBSCOhost Research Databases Search Screen - Advanced Search Database - APA PsycInfo;APA PsycArticles | 984,477 |
| S8 | S2 OR S3 OR S4 | Expanders - Apply equivalent subjects Search modes - Boolean/Phrase | Interface - EBSCOhost Research Databases Search Screen - Advanced Search Database - APA PsycInfo;APA PsycArticles | 527,258 |
| S7 | S1 OR S5 OR S6 | Expanders - Apply equivalent subjects Search modes - Boolean/Phrase | Interface - EBSCOhost Research Databases Search Screen - Advanced Search Database - APA PsycInfo;APA PsycArticles | 693,425 |
| S6 | TI "health research" OR AB "health research" | Expanders - Apply equivalent subjects Search modes - Boolean/Phrase | Interface - EBSCOhost Research Databases Search Screen - Advanced Search Database - APA PsycInfo;APA PsycArticles | 5,952 |
| S5 | TI health OR AB health | Expanders - Apply equivalent subjects Search modes - Boolean/Phrase | Interface - EBSCOhost Research Databases Search Screen - Advanced Search Database - APA PsycInfo;APA PsycArticles | 627, 134 |
| S4 | TI ( Involv* OR "advisory group*" OR "research advisory group" OR "research advisory panel*" OR "advisory panel" OR "advisory committee*" OR "advisory board*" OR "youth engagement" OR "patient and public involvement" OR "public and patient involvement" OR "public patient involvement" OR "community based participatory research" OR "youth particip*" OR "adolescent engagement" OR "participatory design" OR "participatory action" OR "needs assessment*" OR "co produc*" OR "co design" OR "Human centered design" OR "Human centred design" OR "User centered design" OR "User centred design" OR "user involvement" OR "peer researcher*" OR "co researcher*" OR "Patient Participation" OR "young researcher*" OR "lived experience" ) OR AB ( Involv* OR "advisory group*" OR "research advisory group" OR "research advisory panel*" OR "advisory panel" OR "advisory committee*" OR "advisory board*" OR "youth engagement" OR "patient and public involvement" OR "public and patient involvement" OR "public patient involvement" OR "community based participatory research" OR "youth particip*" OR "adolescent engagement" OR "participatory design" OR "participatory action" OR "needs assessment*" OR "co produc*" OR "co design" OR "Human centered design" OR "Human centred design" OR "User centered design" OR "User centred design" OR "user involvement" OR "peer researcher*" OR "co researcher*" OR "Patient Participation" OR "young researcher*" OR "lived experience" ) | Expanders - Apply equivalent subjects Search modes - Boolean/Phrase | Interface - EBSCOhost Research Databases Search Screen - Advanced Search Database - APA PsycInfo;APA PsycArticles | 516, 089 |
| S3 | (MM "Action Research") OR (MM "Stakeholder") | Expanders - Apply equivalent subjects Search modes - Boolean/Phrase | Interface - EBSCOhost Research Databases Search Screen - Advanced Search Database - APA PsycInfo;APA PsycArticles | 5,801 |
| S2 | MM "Involvement" OR MM "Community Involvement" OR MM "Participation" | Expanders - Apply equivalent subjects Search modes - Boolean/Phrase | Interface - EBSCOhost Research Databases Search Screen - Advanced Search Database - APA PsycInfo;APA PsycArticles | 13,628 |
| S1 | MM "Health" OR MM "Adolescent Health" OR MM "Cardiovascular Health" OR MM "Child Health" OR MM "Disease Outbreaks" OR MM "Disease Surveillance" OR MM "Emotional Health" OR MM "Epidemiology" OR MM "Global Health" OR MM "Health Anxiety" OR MM "Health Awareness" OR MM "Health Disparities" OR MM "Health Literacy" OR MM "Health Locus of Control" OR MM "Health Outcomes" OR MM "Health Promotion" OR MM "Health Status" OR MM "Holistic Health" OR MM "Mental Health" OR MM "Occupational Health" OR MM "Oral Health" OR MM "Physical Health" OR MM "Population Health" OR MM "Public Health" OR MM "Reproductive Health" OR MM "Rural Health" OR MM "Sexual Health" OR MM "Social Health" OR MM "Urban Health" OR MM "Well Being" | Expanders - Apply equivalent subjects Search modes - Boolean/Phrase | Interface - EBSCOhost Research Databases Search Screen - Advanced Search Database - APA PsycInfo;APA PsycArticles | 220,028 |

Bottom of Form
