# Supplementary material for: An umbrella review of reviews on challenges to meaningful adolescent involvement in health research
Source: Health Expect. 2024 Jan 27;27(1):e13980. doi: 10.1111/hex.13980 (PMC10821743; doi:10.1111/hex.13980)
Supplement: Supplementary file 1 — Supporting information. [file HEX-27-e13980-s001.zip › Search record and results/Academic databases and search engines/Embase/Embase.docx]

**Database: Embase**

**Date of search: 30 November 2021**

**[
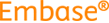
](https://www.embase.com/#search)**

**Results**

**864**

**#21**

**#6** AND **#9** AND **#20** AND [embase]/lim AND ([cochrane review]/lim OR [systematic review]/lim OR [meta analysis]/lim) AND ([conference abstract]/lim OR [conference paper]/lim OR [conference review]/lim OR [review]/lim OR [preprint]/lim) AND [english]/lim

**3,219,157**

**#20**

**#10** OR **#11** OR **#12** OR **#13** OR **#14** OR **#15** OR **#16** OR **#17** OR **#18** OR **#19**

**3,213,592**

**#19**

**involv*** OR **'advisory group*'** OR **'research advisory group'** OR **'research advisory panel*'** OR **'advisory panel'** OR **'advisory committee*'** OR **'advisory board*'** OR **'youth engagement'** OR **'patient and public involvement'** OR **'public and patient involvement'** OR **'public patient involvement'** OR **'community based participatory research'** OR **'youth particip*'** OR **'adolescent engagement'** OR **'participatory design'** OR **'participatory action'** OR **'needs assessment*'** OR **'co produc*'** OR **'co design'** OR **'human centered design'** OR **'human centred design'** OR **'user centered design'** OR **'user centred design'** OR **'user involvement'** OR **'peer researcher*'** OR **'co researcher*'** OR **'patient participation'** OR **'young researcher*'** OR **'lived experience'**

**25,819**

**#18**

**'needs assessment'**/exp

**240**

**#17**

**'universal design'**/exp

**19**

**#16**

**'patient and public involvement'**/exp

**4,392**

**#15**

**'stakeholder engagement'**/exp

**371**

**#14**

**'participatory action research'**/exp

**6,536**

**#13**

**'participatory research'**/exp

**41**

**#12**

**'engagement'**/exp

**13,353**

**#11**

**'advisory committee'**/exp

**10**

**#10**

**'involvement'**/exp

**1,000,893**

**#9**

**#7** OR **#8**

**246,807**

**#8**

**'health research'**

**775,993**

**#7**

**'health'**/exp

**5,525,673**

**#6**

**#1** OR **#2** OR **#3** OR **#4** OR **#5**

**4,982,268**

**#5**

**child*** OR **youth** OR **adolescen*** OR **'young people'** OR **'young person*'** OR **'young adult*'** OR **teen*** OR **juven***:ab,kw,ti

**3,100,833**

**#4**

**'child'**/exp

**433,229**

**#3**

**'young adult'**/exp

**42,598**

**#2**

**'adolescent'**/exp/mj

**4,049,012**

**#1**

**'juvenile'**/exp

[
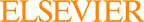
](https://www.elsevier.com/)

**© 2021 RELX Intellectual Properties SA. All rights reserved.**

Embase, RELX Group and the RE symbol are trade marks of RELX Intellectual Properties SA, used under license.
