# Supplementary material for: An umbrella review of reviews on challenges to meaningful adolescent involvement in health research
Source: Health Expect. 2024 Jan 27;27(1):e13980. doi: 10.1111/hex.13980 (PMC10821743; doi:10.1111/hex.13980)
Supplement: Supplementary file 1 — Supporting information. [file HEX-27-e13980-s001.zip › Search record and results/Academic databases and search engines/ProQuest/ProQuest.docx]

**Database:** ProQuest

**Date of search:** 30 November 2021

[noft("health research") AND noft(child* OR youth OR adolescen* OR "young people" OR ("young person" OR "young persons") OR ("young adult" OR "young adulthood" OR "young adults") OR teen* OR juven*) AND noft(Involv* OR ("advisory group" OR "advisory groups") OR "research advisory group" OR "research advisory panel*" OR "advisory panel" OR ("advisory committee" OR "advisory committees") OR ("advisory board" OR "advisory boards") OR "youth engagement" OR "patient and public involvement" OR "public and patient involvement" OR "public patient involvement" OR "community based participatory research" OR ("youth participants" OR "youth participate" OR "youth participated" OR "youth participating" OR "youth participation") OR "adolescent engagement" OR "participatory design" OR "participatory action" OR ("needs assessment" OR "needs assessments") OR "co produc*" OR "co design" OR "Human centered design" OR "Human centred design" OR "User centered design" OR "User centred design" OR "user involvement" OR "peer researcher*" OR "co researcher*" OR "Patient Participation" OR ("young researcher" OR "young researchers") OR "lived experience") AND noft(review)](https://www.proquest.com/myresearch/savedsearches.checkdbssearchlink:rerunsearch/2051204/SavedSearches?t:ac=SavedSearches)

**Number of results=394**
