# Supplementary material for: An umbrella review of reviews on challenges to meaningful adolescent involvement in health research
Source: Health Expect. 2024 Jan 27;27(1):e13980. doi: 10.1111/hex.13980 (PMC10821743; doi:10.1111/hex.13980)
Supplement: Supplementary file 1 — Supporting information. [file HEX-27-e13980-s001.zip › Search record and results/Academic databases and search engines/Scopus/Scopus.docx]

**Database:** Scopus

**Date of search:** 30 November 2021

## ( TITLE-ABS-KEY ( "health research" )  AND  TITLE-ABS-KEY ( child*  OR  youth  OR  adolescen*  OR  "young people"  OR  "Young person*"  OR  "Young adult*"  OR  teen*  OR  juven* )  AND  TITLE-ABS-KEY ( involv*  OR  "advisory group*"  OR  "research advisory group"  OR  "research advisory panel*"  OR  "advisory panel"  OR  "advisory committee*"  OR  "advisory board*"  OR  "youth engagement"  OR  "patient and public involvement"  OR  "public and patient involvement"  OR  "public patient involvement"  OR  "community based participatory research"  OR  "youth particip*"  OR  "adolescent engagement"  OR  "participatory design"  OR  "participatory action"  OR  "needs assessment*"  OR  "co produc*"  OR  "co design"  OR  "Human centered design"  OR  "Human centred design"  OR  "User centered design"  OR  "User centred design"  OR  "user involvement"  OR  "peer researcher*"  OR  "co researcher*"  OR  "Patient Participation"  OR  "young researcher*"  OR  "lived experience" ) )  AND  ( LIMIT-TO ( DOCTYPE ,  "re" ) )

**Number of results=104**
