# Supplementary material for: An umbrella review of reviews on challenges to meaningful adolescent involvement in health research
Source: Health Expect. 2024 Jan 27;27(1):e13980. doi: 10.1111/hex.13980 (PMC10821743; doi:10.1111/hex.13980)

**Database: Web of Science (All databases)**

**Date of search: 30 November 2021**

(((TS=(review)) AND TS=(“Health research” )) AND TS=(child* OR youth OR adolescen* OR "young people" OR "Young person*" OR "Young adult*" OR teen* OR juven*)) AND TS=(Involv* OR "advisory group*" OR "research advisory group" OR "research advisory panel*" OR "advisory panel" OR "advisory committee*" OR "advisory board*" OR "youth engagement" OR "patient and public involvement" OR "public and patient involvement" OR "public patient involvement" OR "community based participatory research" OR "youth particip*" OR "adolescent engagement" OR "participatory design" OR "participatory action" OR "needs assessment*" OR "co produc*" OR "co design" OR "Human centered design" OR "Human centred design" OR "User centered design" OR "User centred design" OR "user involvement" OR "peer researcher*" OR "co researcher*" OR "Patient Participation" OR "young researcher*" OR “lived experience”)

**Number of results=307**


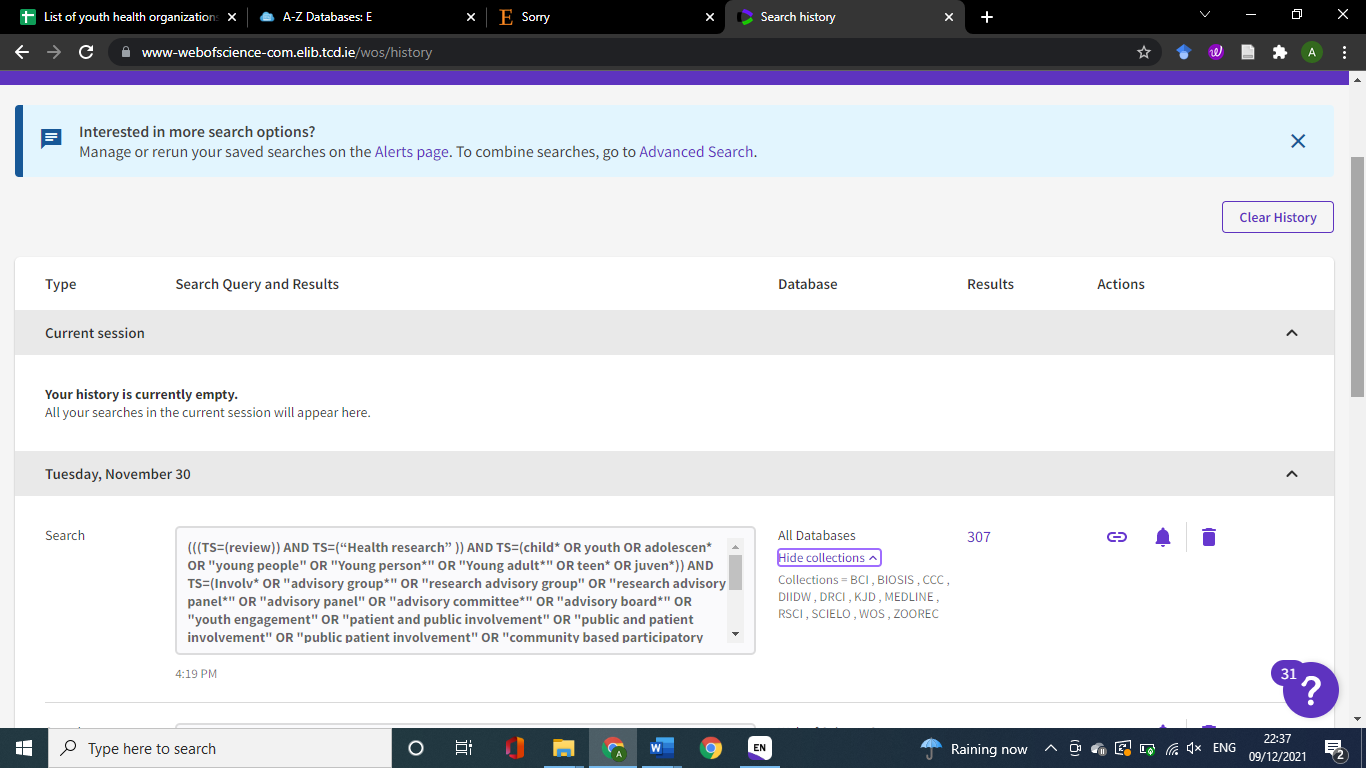

Supplement: Supplementary file 1 — Supporting information. [file HEX-27-e13980-s001.zip › Search record and results/Academic databases and search engines/Web of Science/Web of Science.docx]
