# Supplementary material for: An umbrella review of reviews on challenges to meaningful adolescent involvement in health research
Source: Health Expect. 2024 Jan 27;27(1):e13980. doi: 10.1111/hex.13980 (PMC10821743; doi:10.1111/hex.13980)
Supplement: Supplementary file 1 — Supporting information. [file HEX-27-e13980-s001.zip › Search record and results/Other sources/Google Scholar/Search strings, results, screenshots.docx]

| **Google scholar search**  Date: 8^th^ January 2022  Filters= Review articles  Sort by relevance  Each string searched up to 10 pages  Total results searched= 1400  After removal of duplicates between results of different search strings=1117  **Table of contents**   \| **Sr no.** \| **Search strings** \| **Results** \| **Page number** \| \| --- \| --- \| --- \| --- \| \| 1 \| ("health research") AND (child* OR youth OR adolescen* OR "young people" OR "Young person*" OR "Young adult*" OR teen* OR juven*) AND (Involv* OR "advisory group*" OR "research advisory group" OR "research advisory panel*" OR "advisory panel") \| 13,300 \| 2 \| \| 2 \| ("health research") AND (child* OR youth OR adolescen* OR "young people" OR "Young person*" OR "Young adult*" OR teen* OR juven*) AND ("advisory committee*" OR "advisory board*" OR "youth engagement" OR "patient and public involvement") \| 7900 \| 26 \| \| 3 \| ("health research") AND (child* OR youth OR adolescen* OR "young people" OR "Young person*" OR "Young adult*" OR teen* OR juven*) AND ("public and patient involvement" OR "public patient involvement" OR "community based participatory research") \| 1740 \| 52 \| \| 4 \| ("health research") AND (child* OR youth OR adolescen* OR "young people" OR "Young person*" OR "Young adult*" OR teen* OR juven*) AND ("youth particip*" OR "adolescent engagement" OR "participatory design" OR "participatory action" OR "needs assessment*") \| 3530 \| 77 \| \| 5 \| ("health research") AND (child* OR youth OR adolescen* OR "young people" OR "Young person*" OR "Young adult*" OR teen* OR juven*) AND ("co produc*" OR "co design" OR "Human centered design" OR "Human centred design" OR "User centered design") \| 734 \| 105 \| \| 6 \| ("health research") AND (child* OR youth OR adolescen* OR "young people" OR "Young person*" OR "Young adult*" OR teen* OR juven*) AND ("User centred design" OR "user involvement" OR "peer researcher*" OR "co researcher*" OR "Patient Participation") \| 1640 \| 133 \| \| 7 \| ("health research") AND (child* OR youth OR adolescen* OR "young people" OR "Young person*" OR "Young adult*" OR teen* OR juven*) AND ("young researcher*" OR "lived experience") \| 2810 \| 162 \| | | | | | | | | | | | | | | |
| --- | --- | --- | --- | --- | --- | --- | --- | --- | --- | --- | --- | --- | --- | --- | --- | --- | --- | --- | --- | --- | --- | --- | --- | --- | --- | --- | --- | --- | --- | --- | --- | --- | --- | --- | --- | --- | --- | --- | --- | --- | --- | --- | --- | --- | --- | --- |
| **Sr no.** | | | | | | | **Search strings** | **Results** | | | | | | |
| 1 | | | | | | | ("health research") AND (child* OR youth OR adolescen* OR "young people" OR "Young person*" OR "Young adult*" OR teen* OR juven*) AND (Involv* OR "advisory group*" OR "research advisory group" OR "research advisory panel*" OR "advisory panel") | 13,300 | | | | | | |
| Page 1 | | | | | | | | | | | | | | |
| 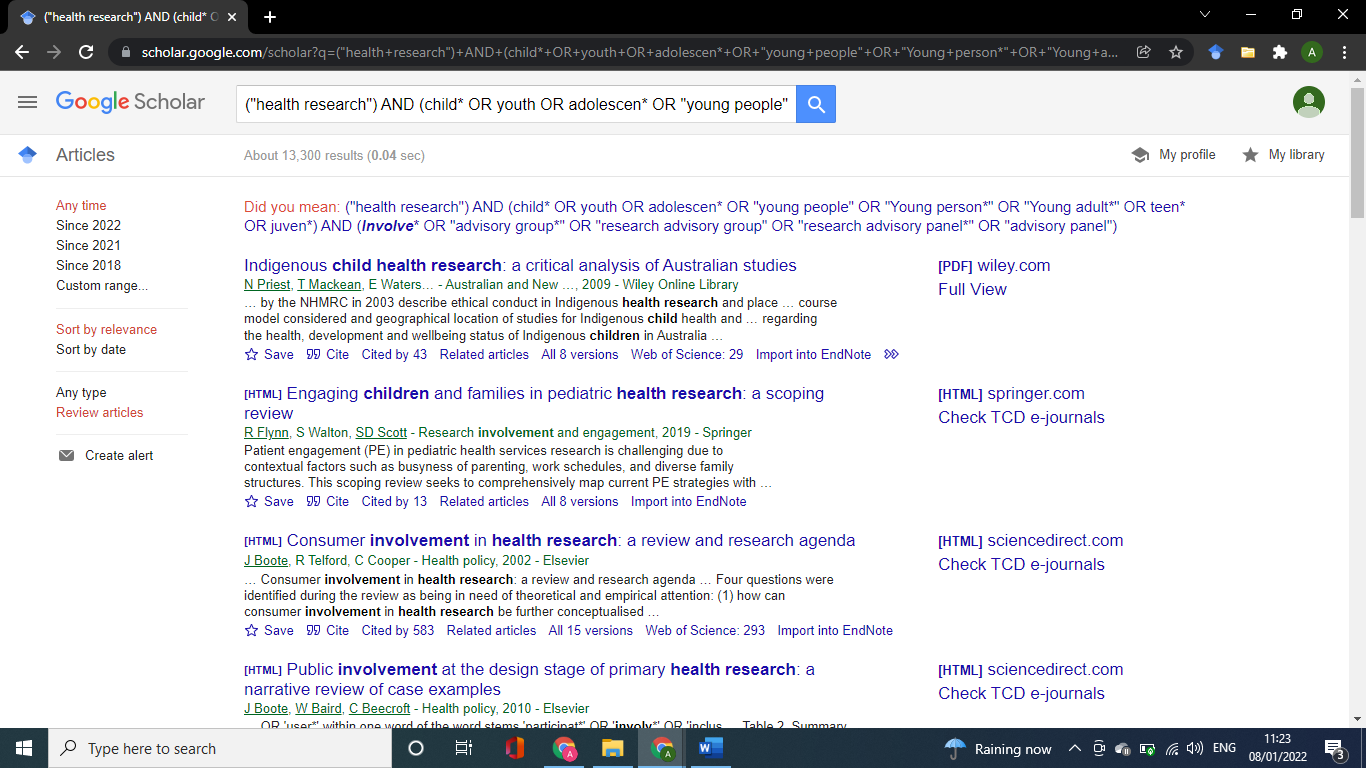 | | | | | | | | | | | | | | |
| 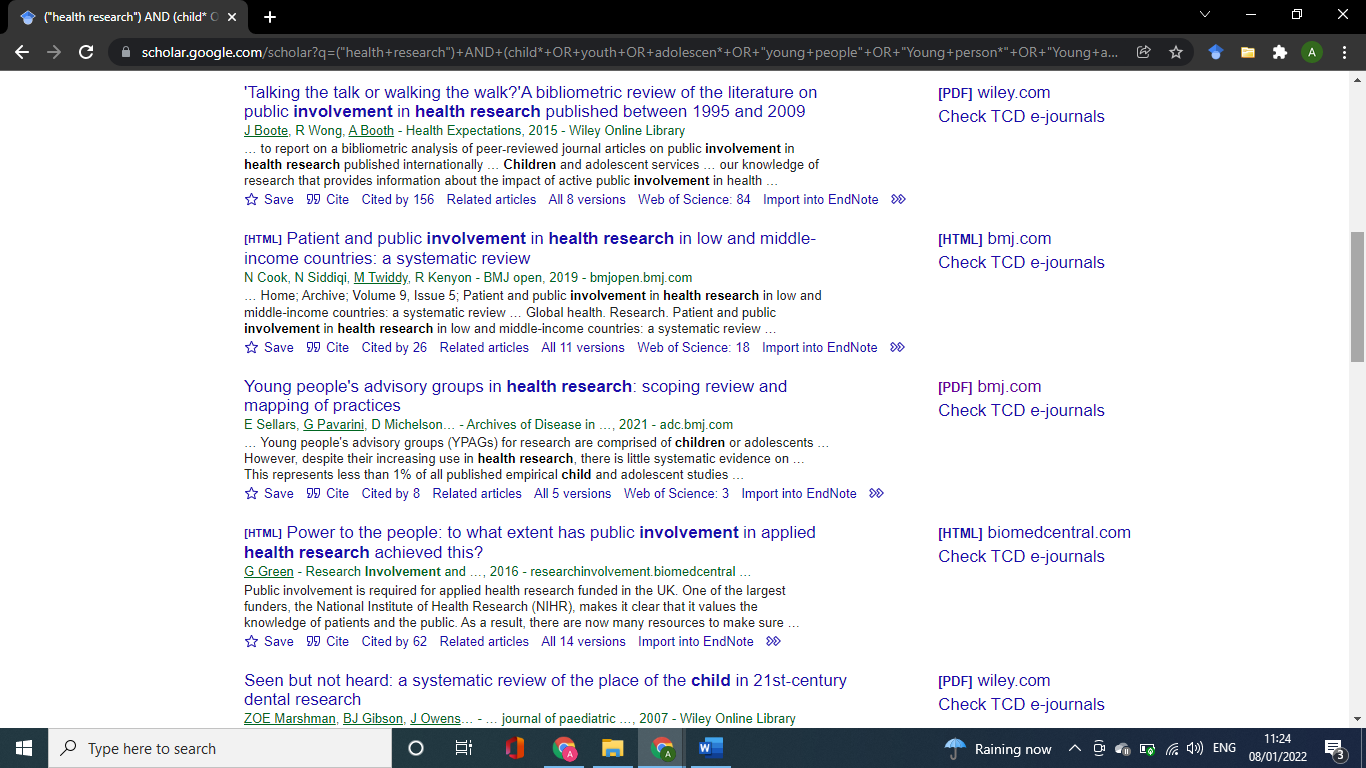 | | | | | | | | | | | | | | |
| 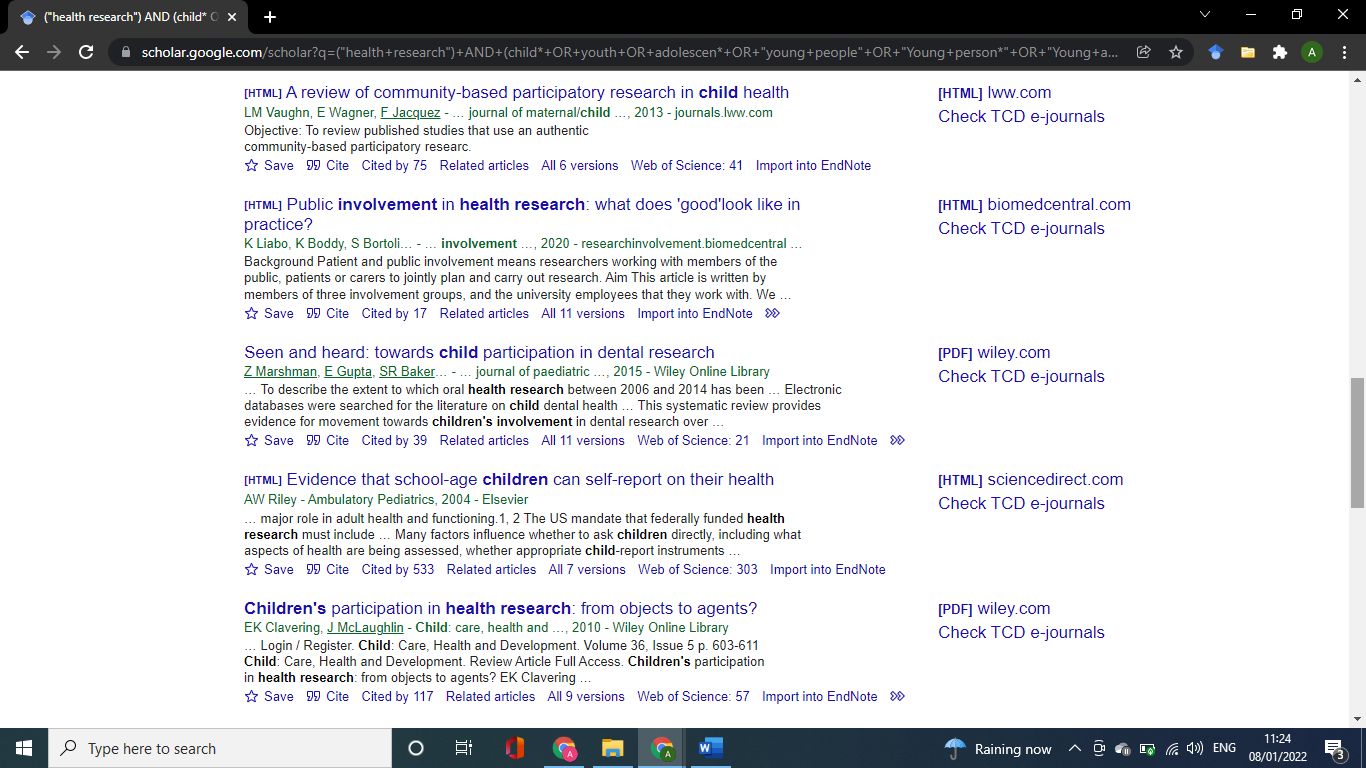 | | | | | | | | | | | | | | |
| 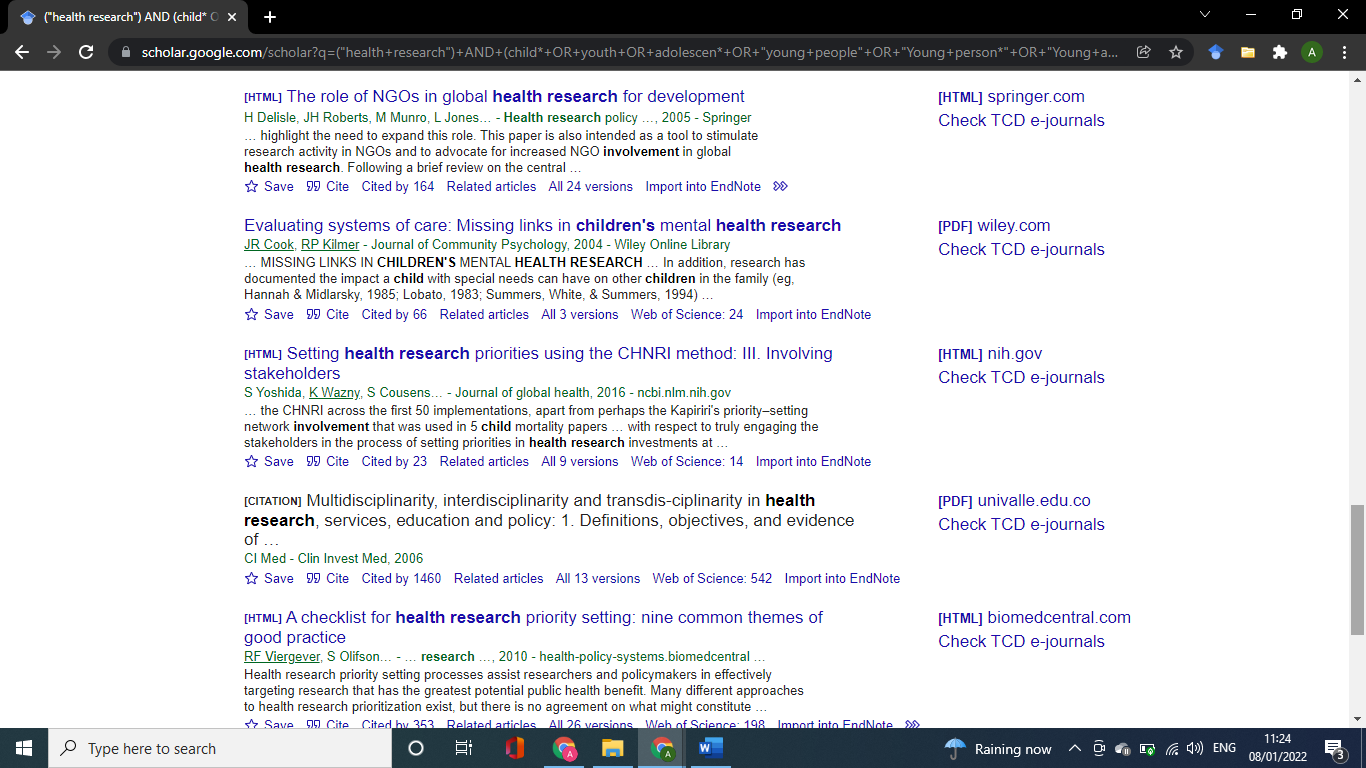 | | | | | | | | | | | | | | |
| 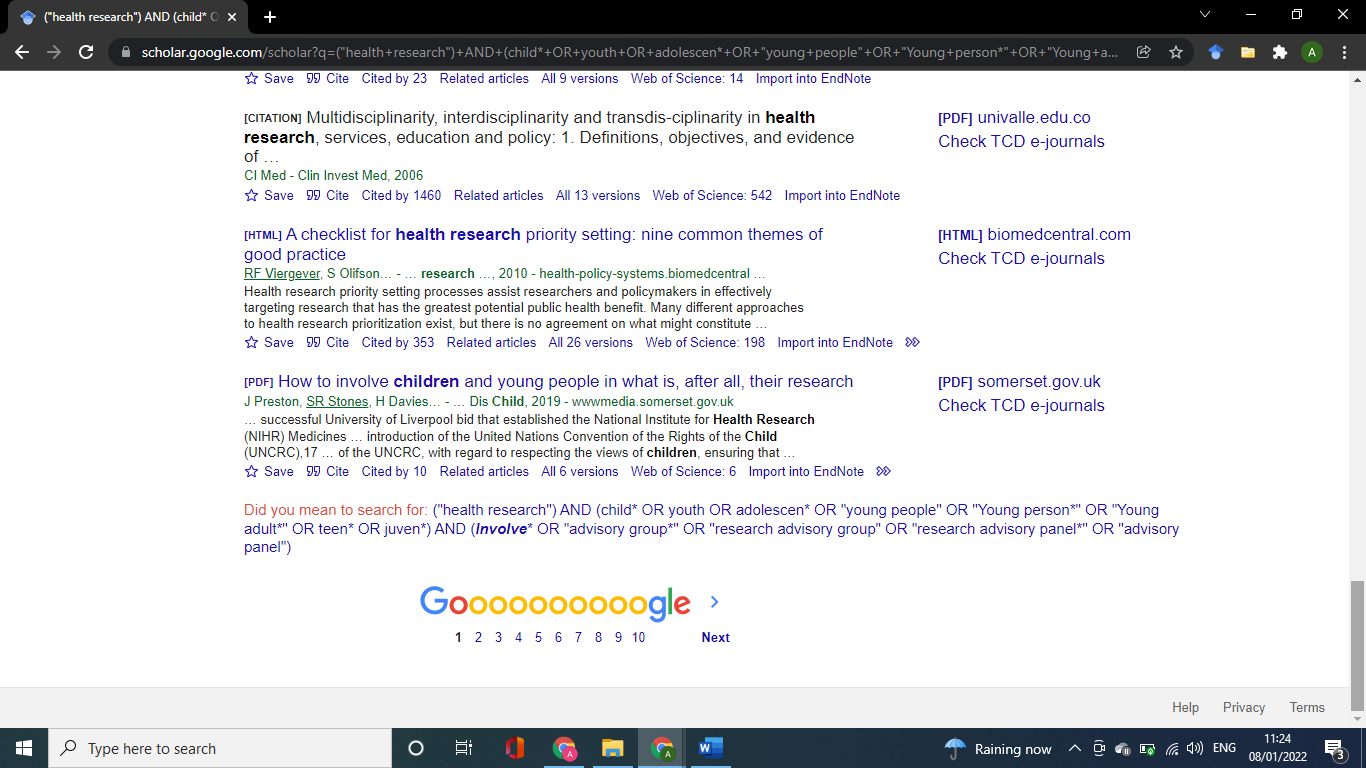 | | | | | | | | | | | | | | |
| Page 2 | | | | | | | | | | | | | | |
| 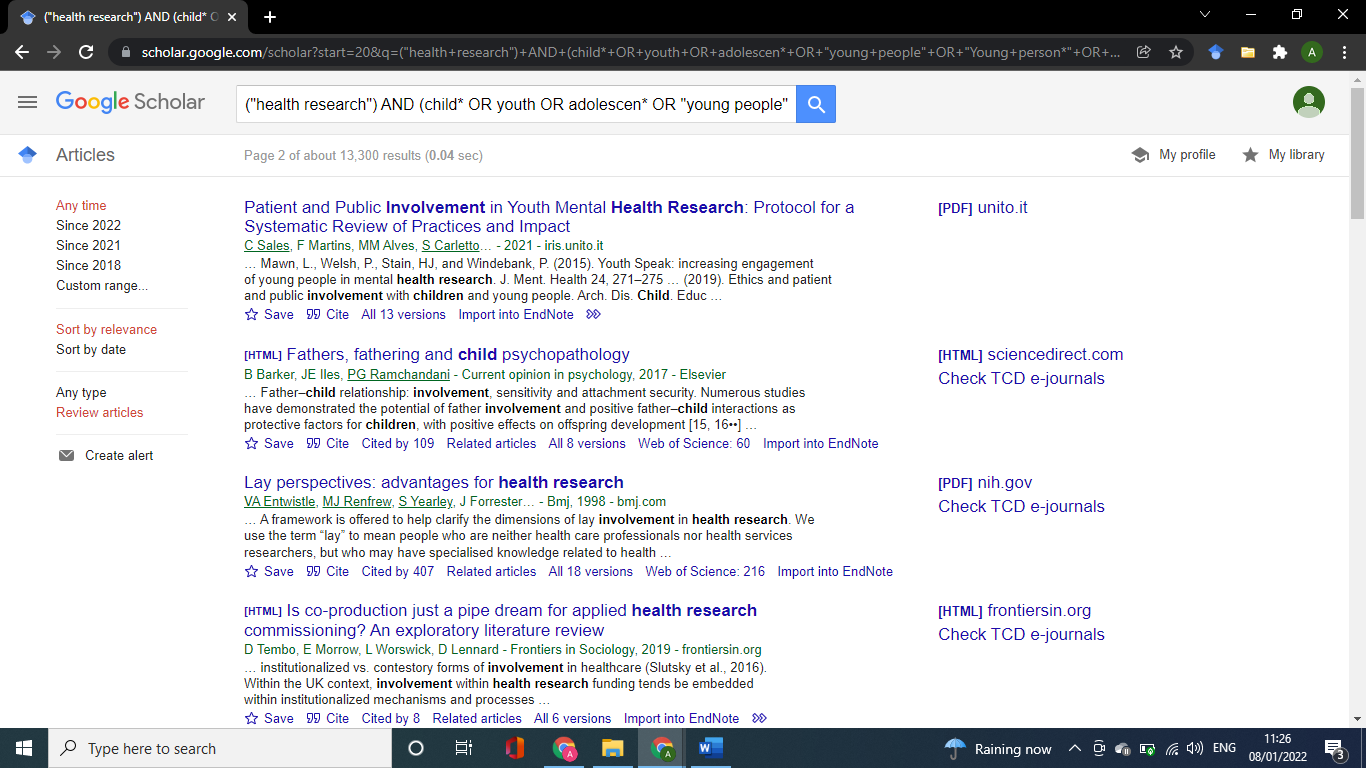 | | | | | | | | | | | | | | |
| 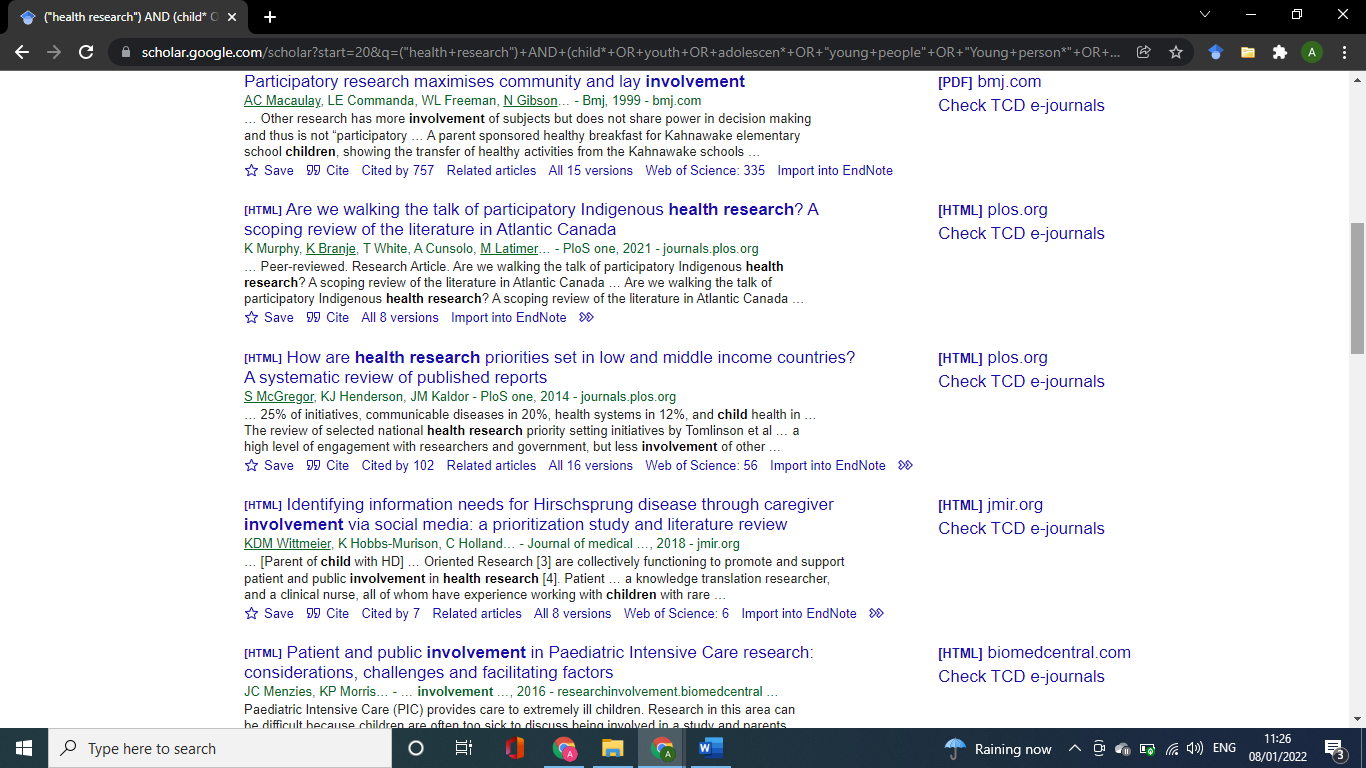 | | | | | | | | | | | | | | |
| 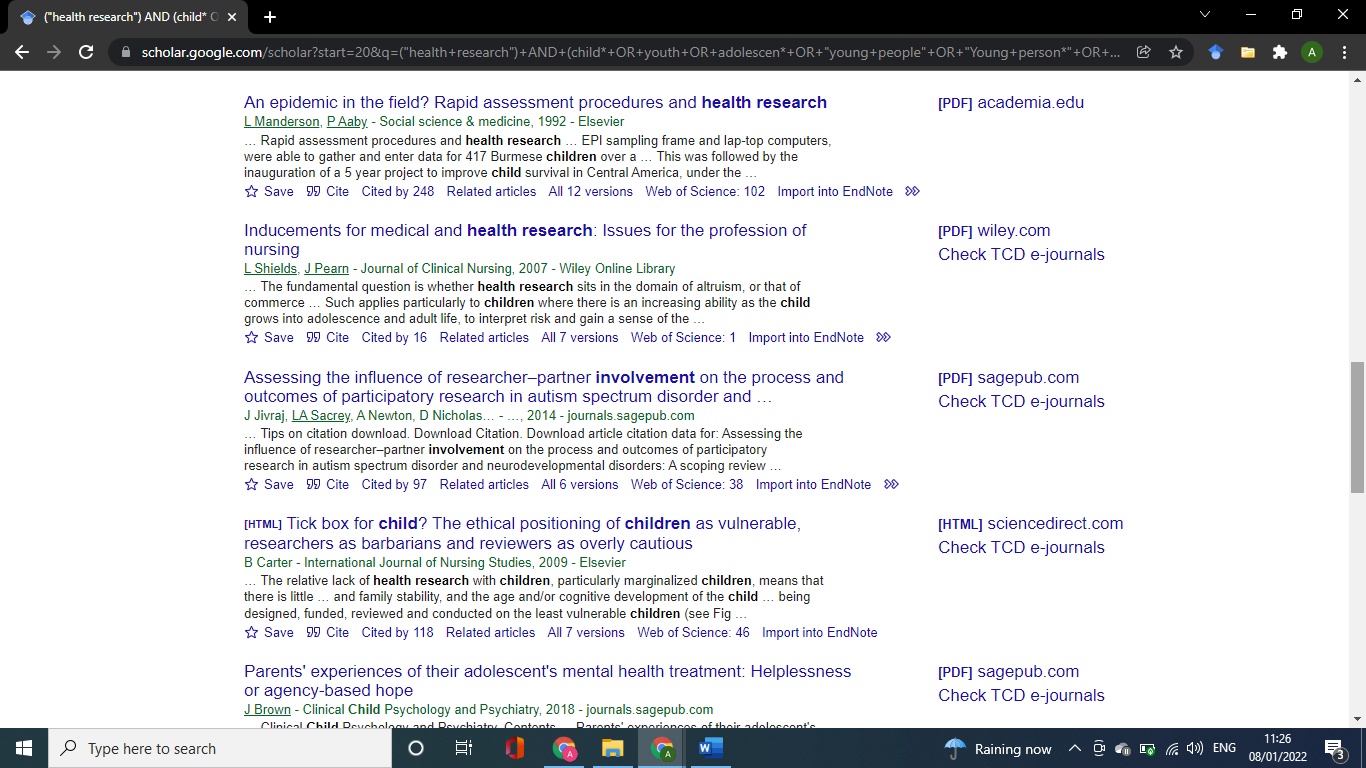 | | | | | | | | | | | | | | |
| 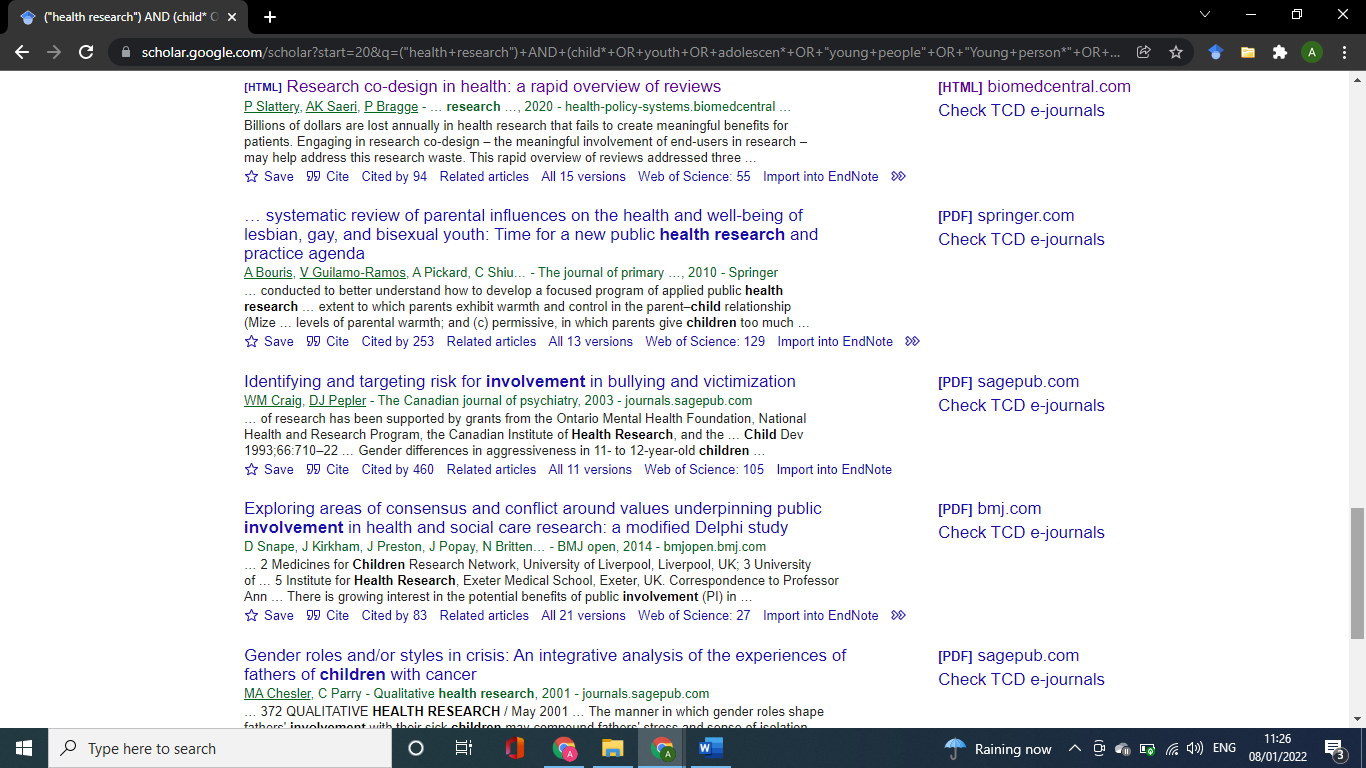 | | | | | | | | | | | | | | |
| 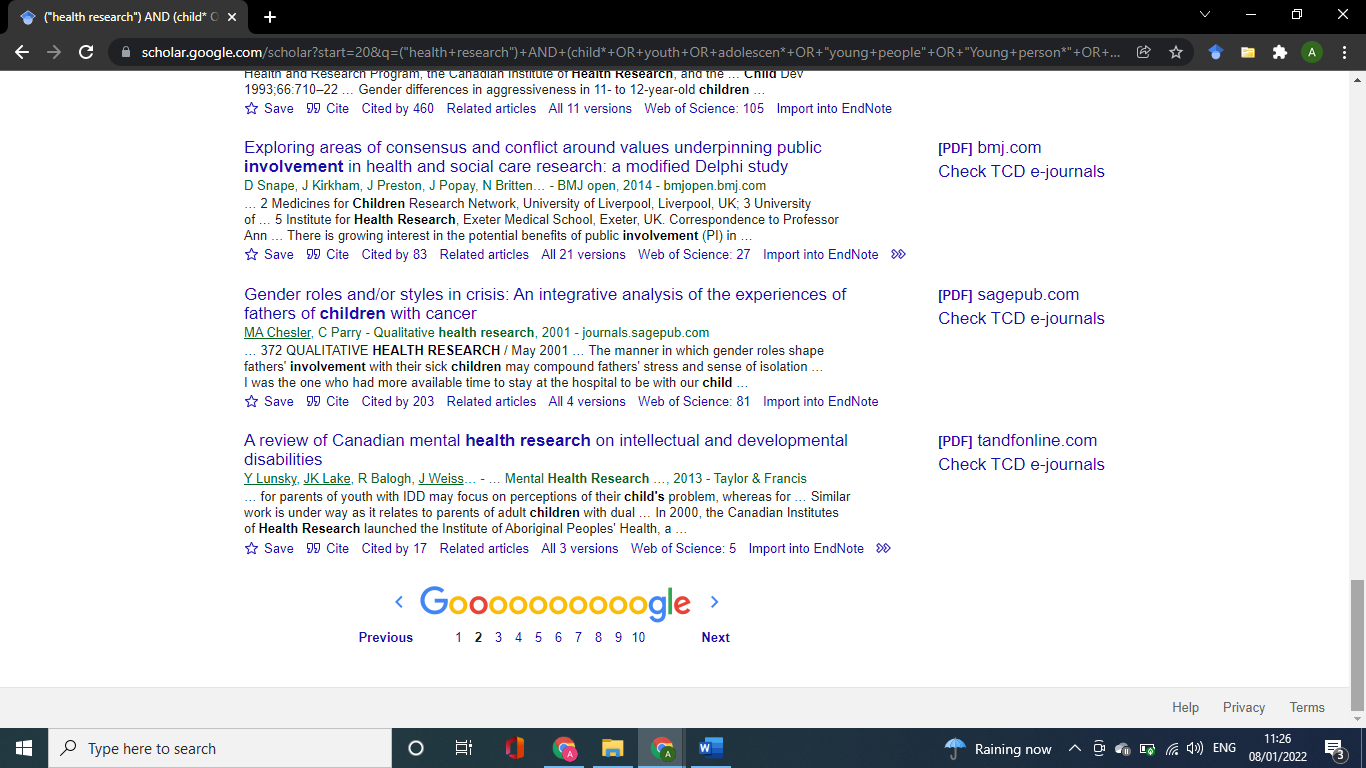 | | | | | | | | | | | | | | |
| Page 3 | | | | | | | | | | | | | | |
| 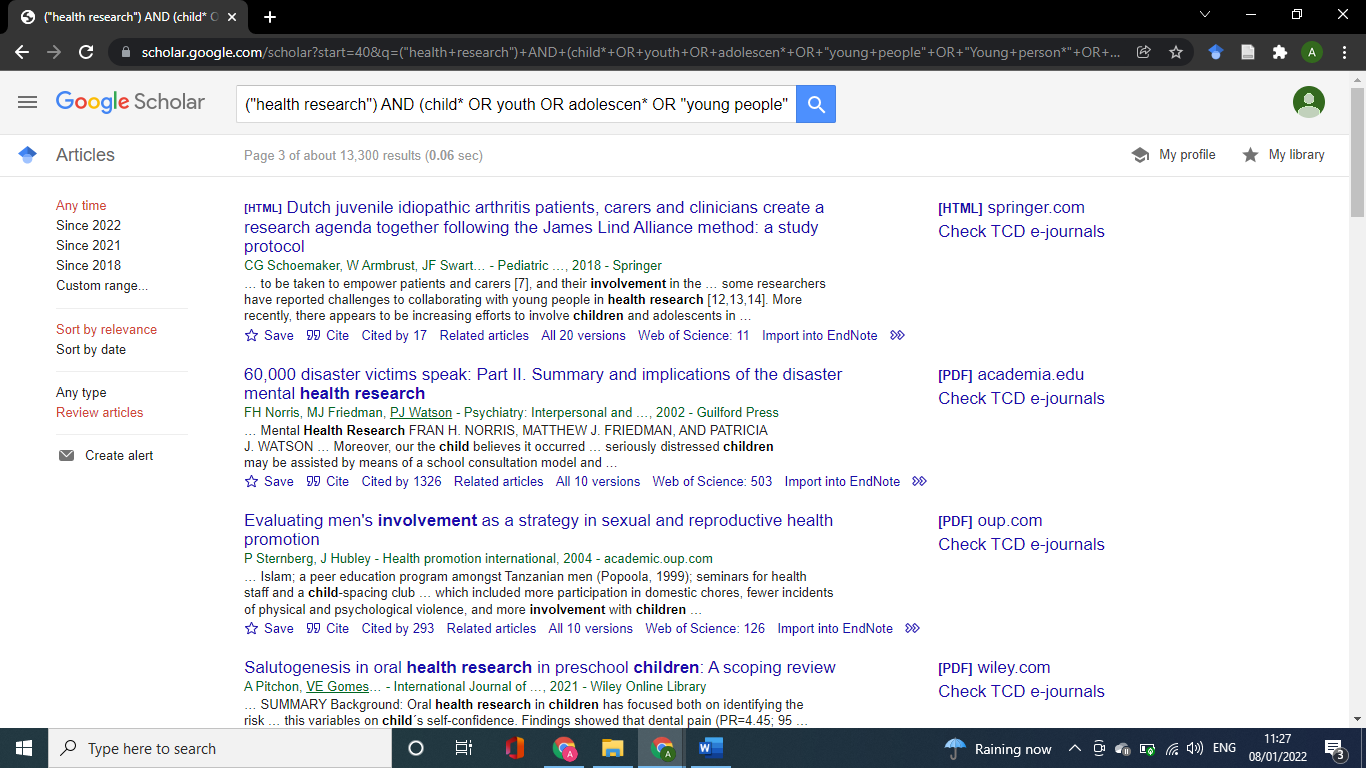 | | | | | | | | | | | | | | |
| 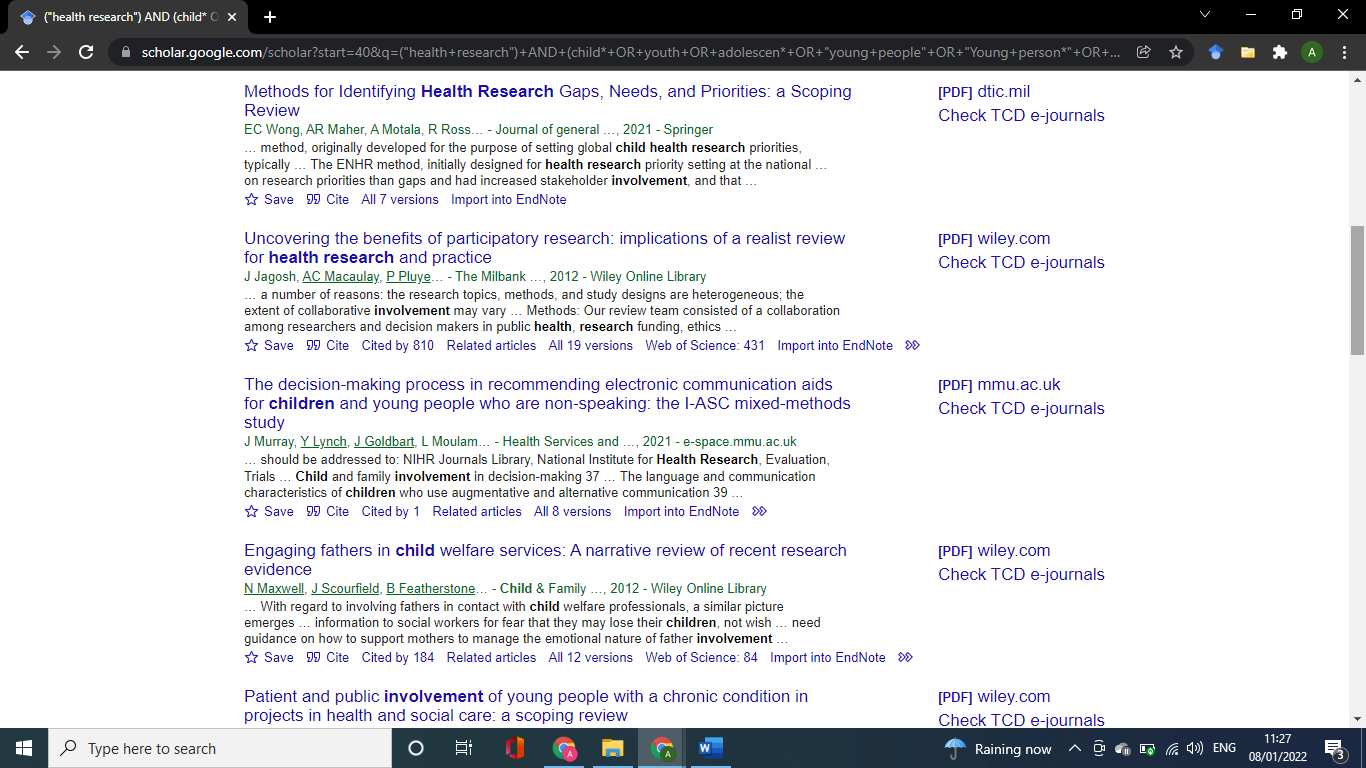 | | | | | | | | | | | | | | |
| 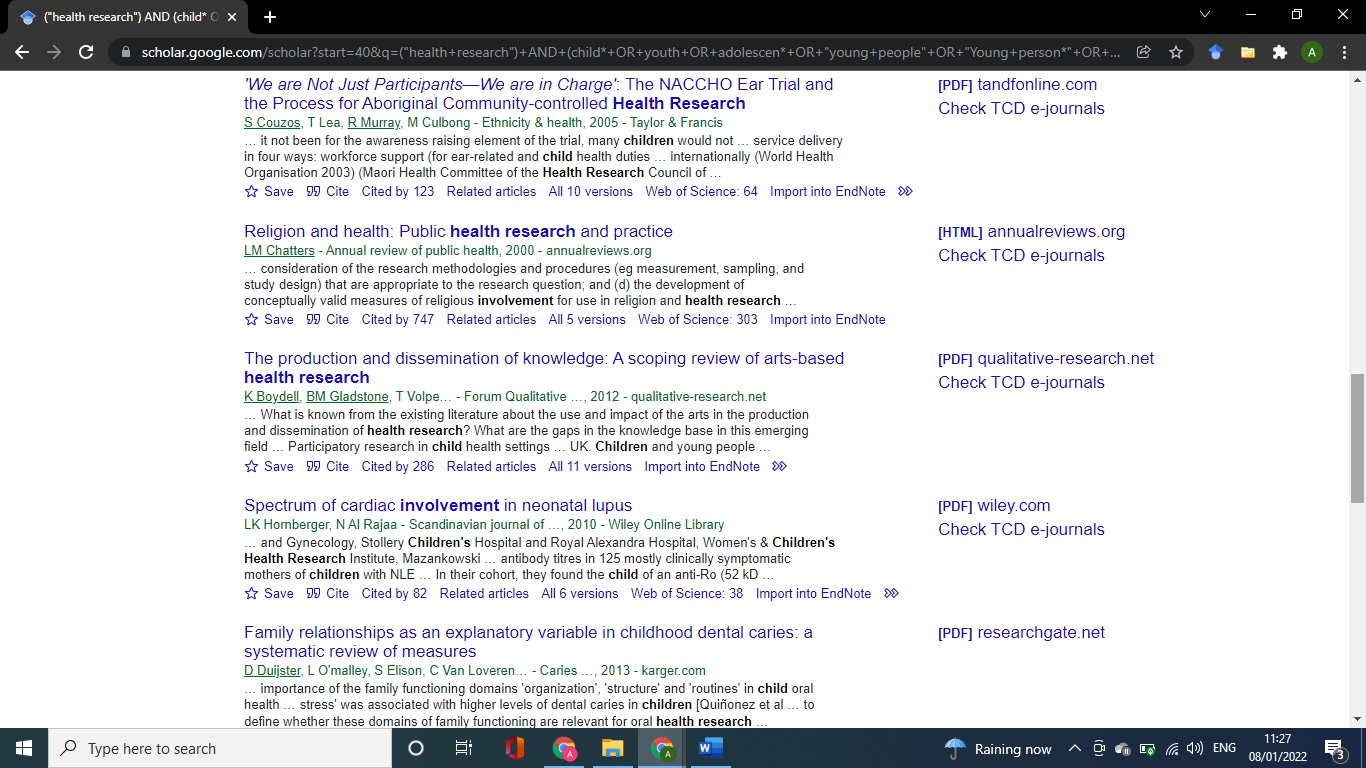 | | | | | | | | | | | | | | |
| 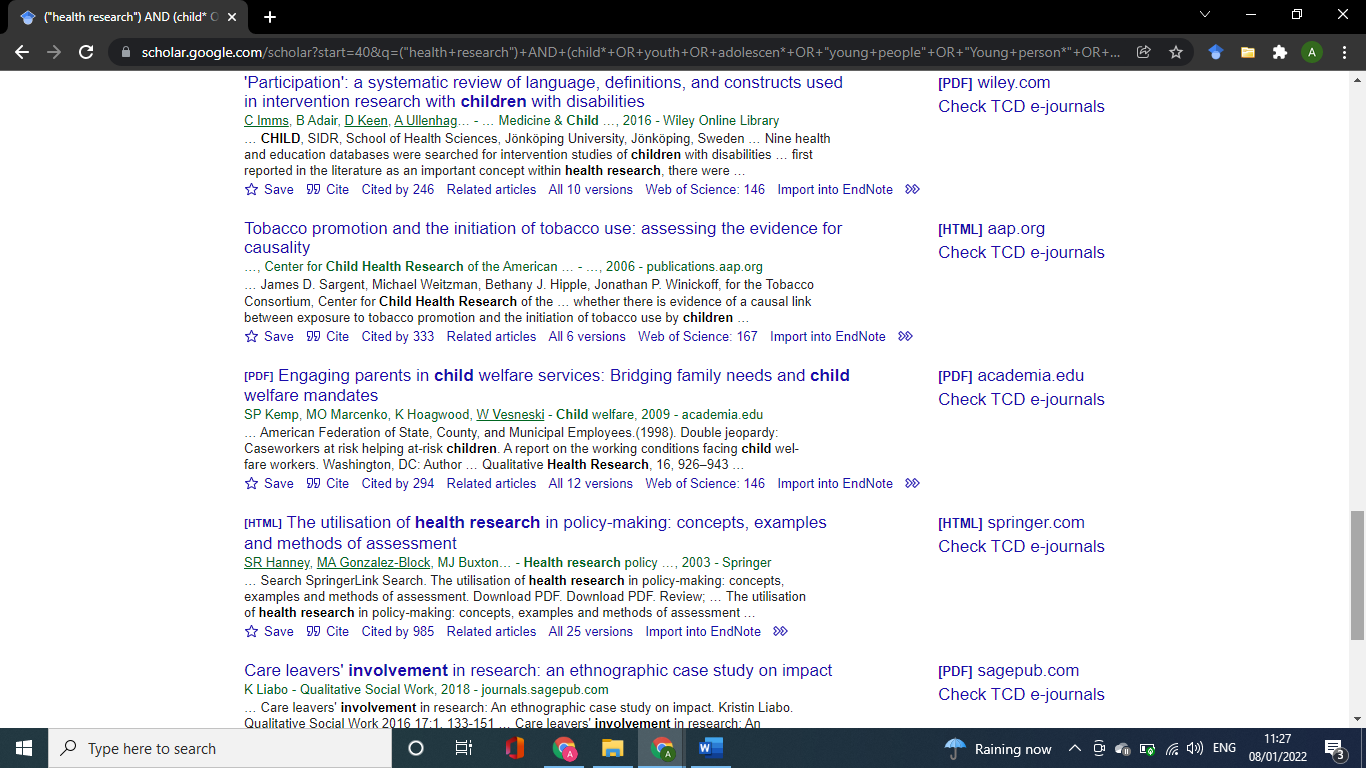 | | | | | | | | | | | | | | |
| 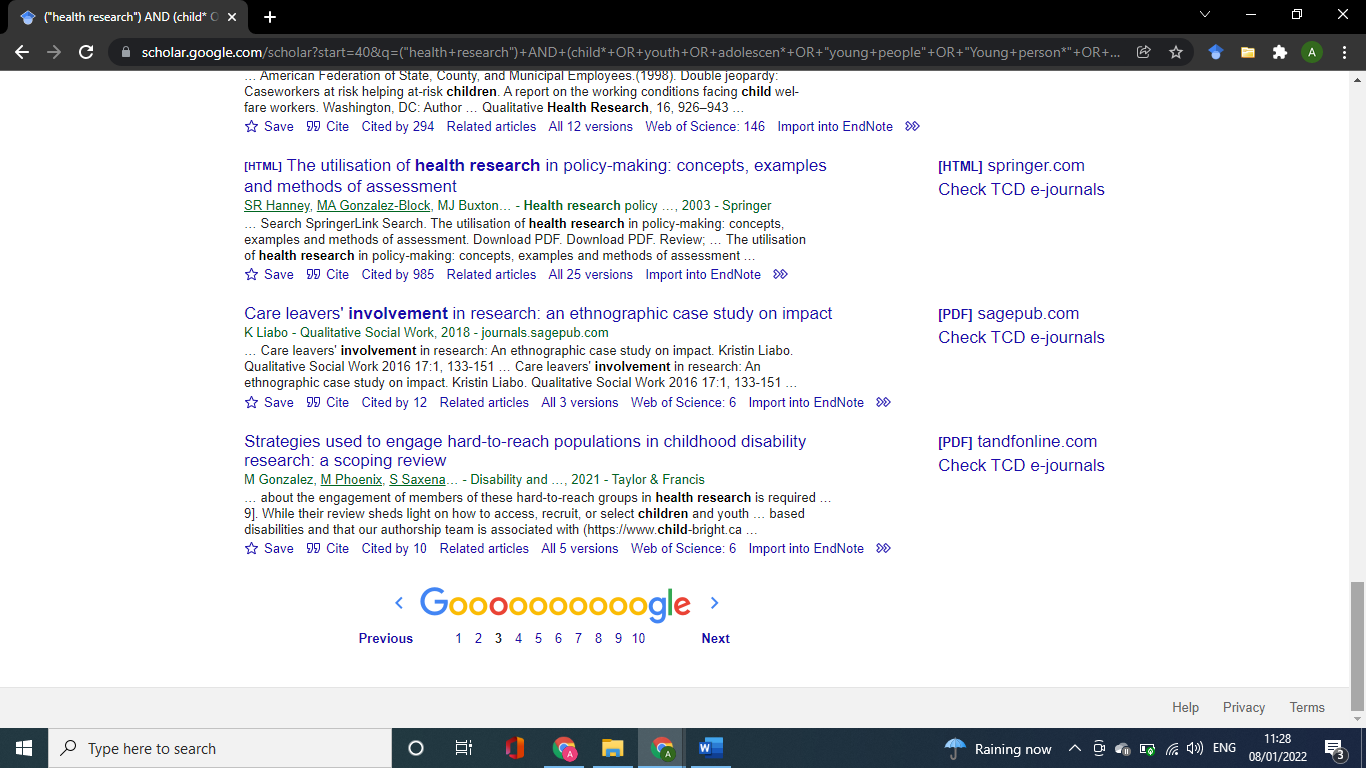 | | | | | | | | | | | | | | |
| Page 4 | | | | | | | | | | | | | | |
| 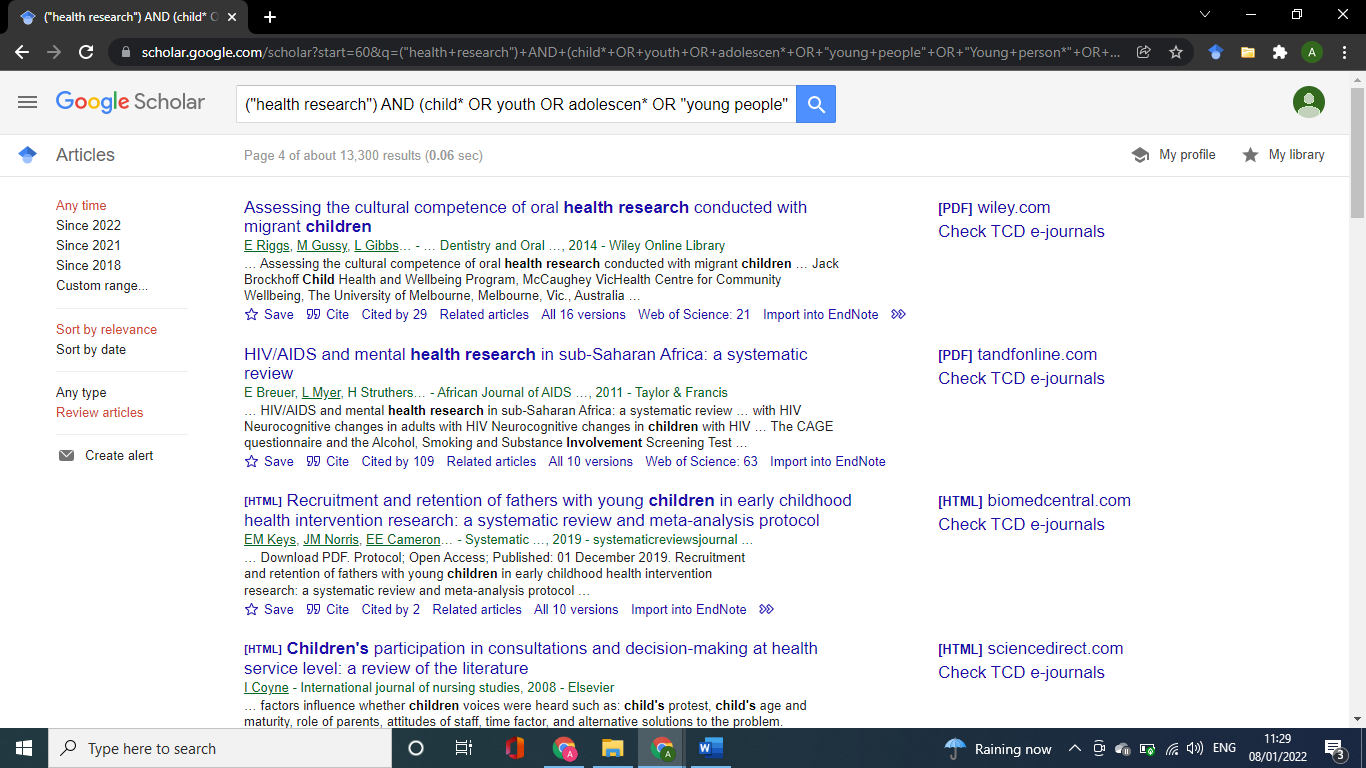 | | | | | | | | | | | | | | |
| 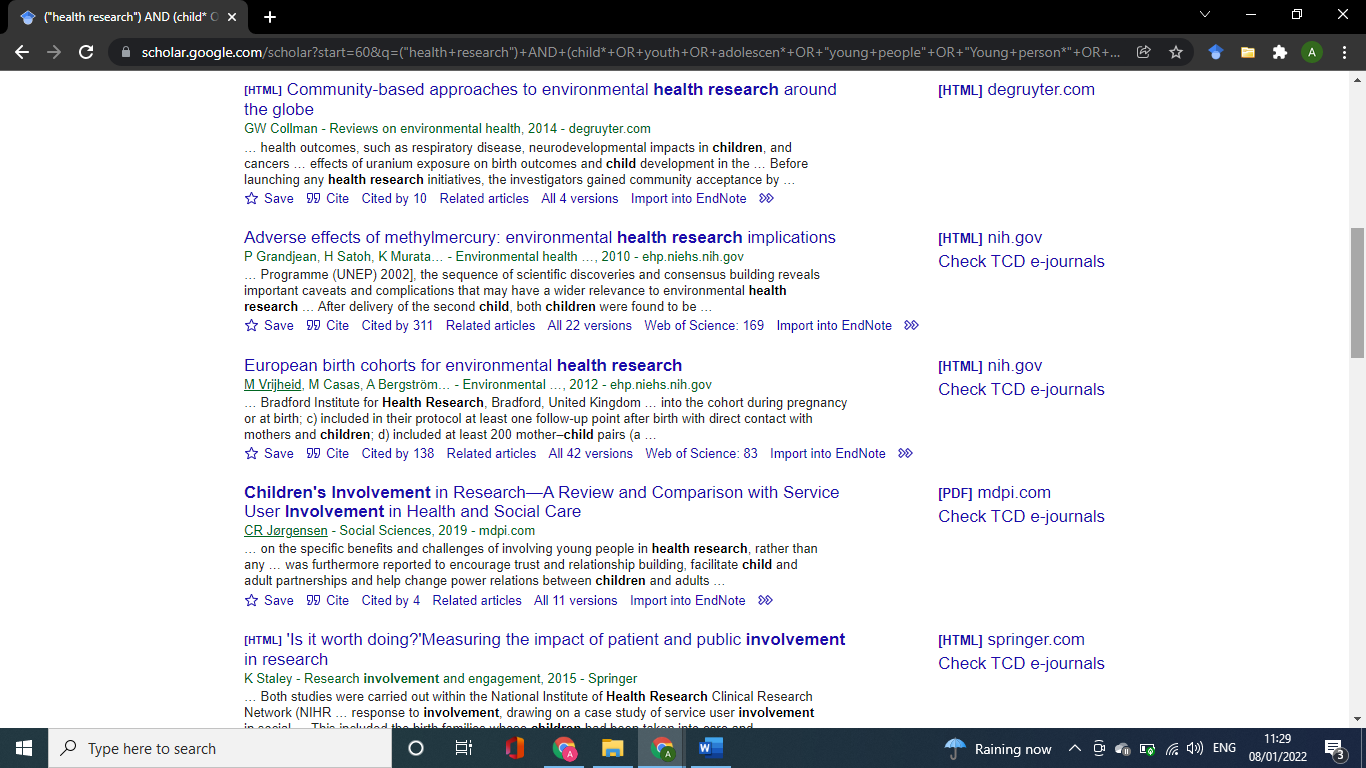 | | | | | | | | | | | | | | |
| 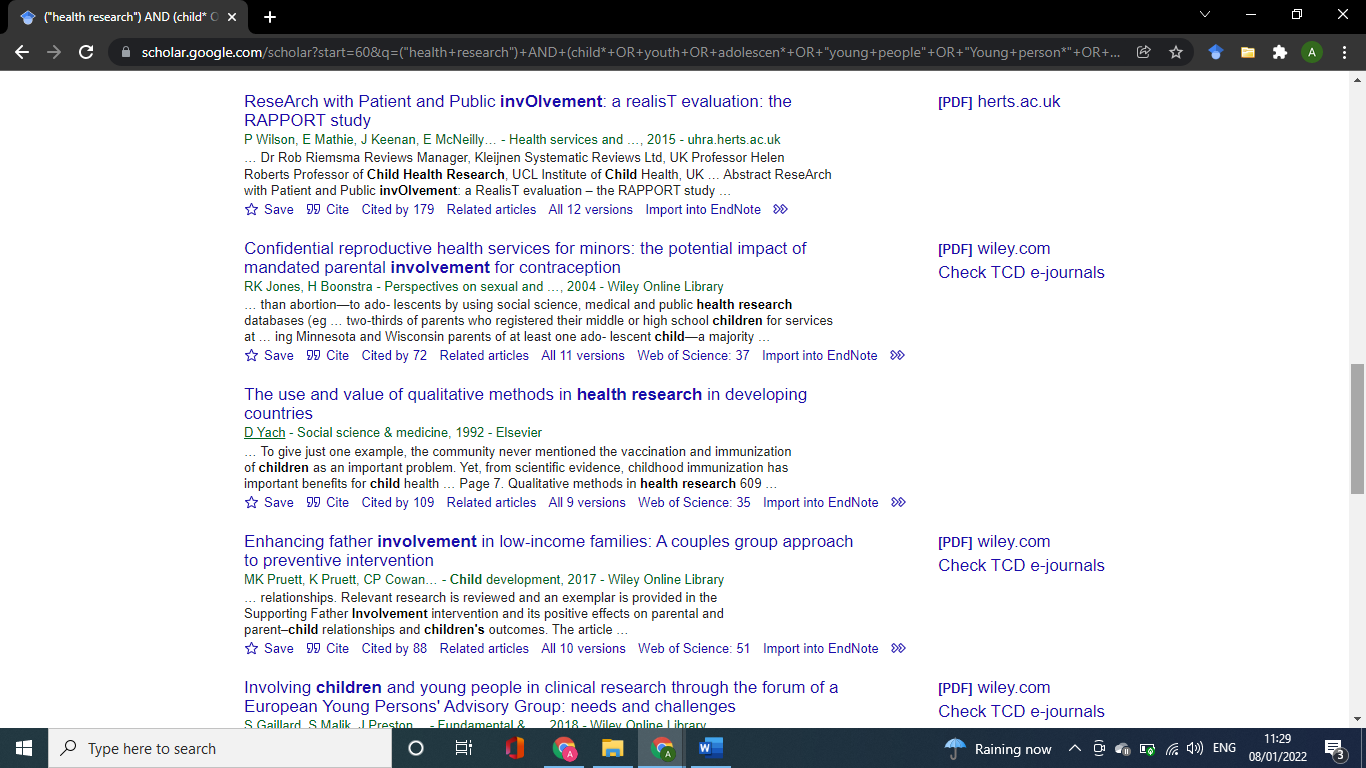 | | | | | | | | | | | | | | |
| 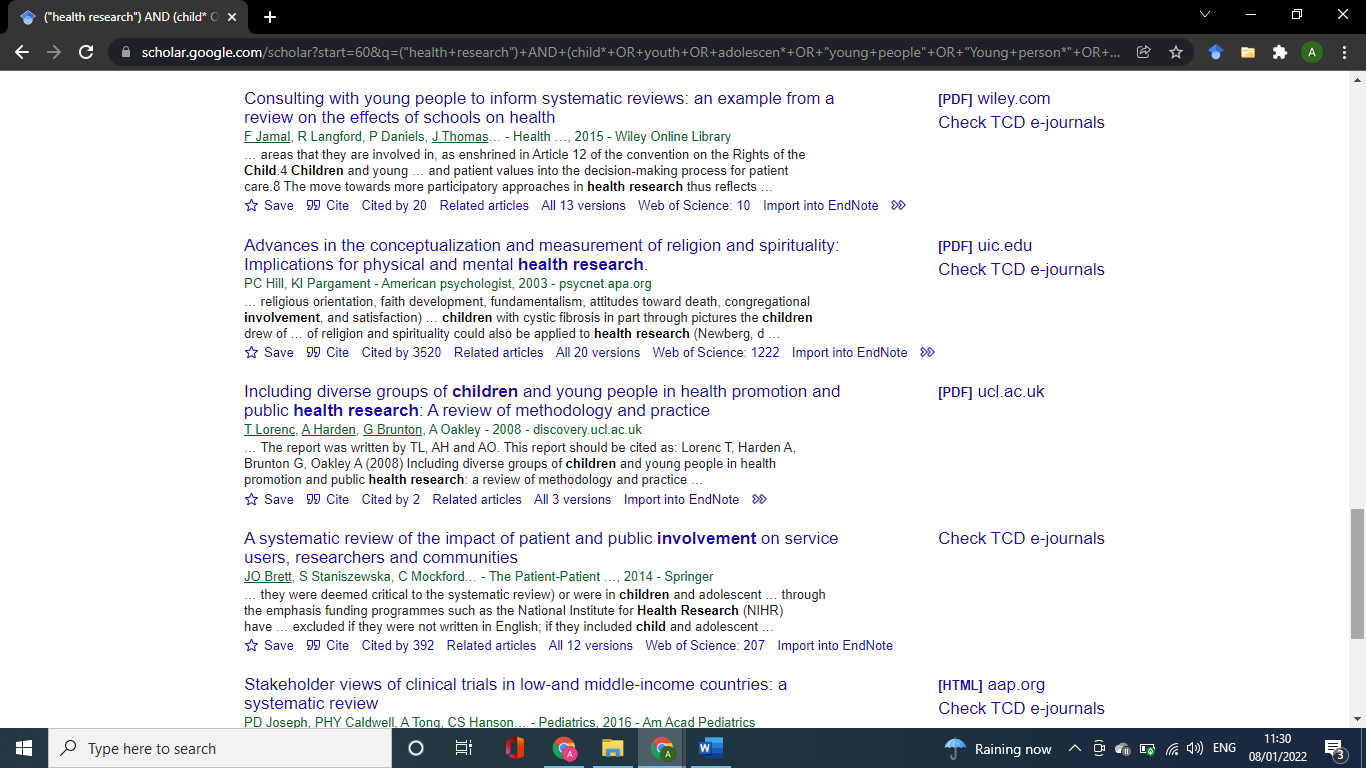 | | | | | | | | | | | | | | |
| 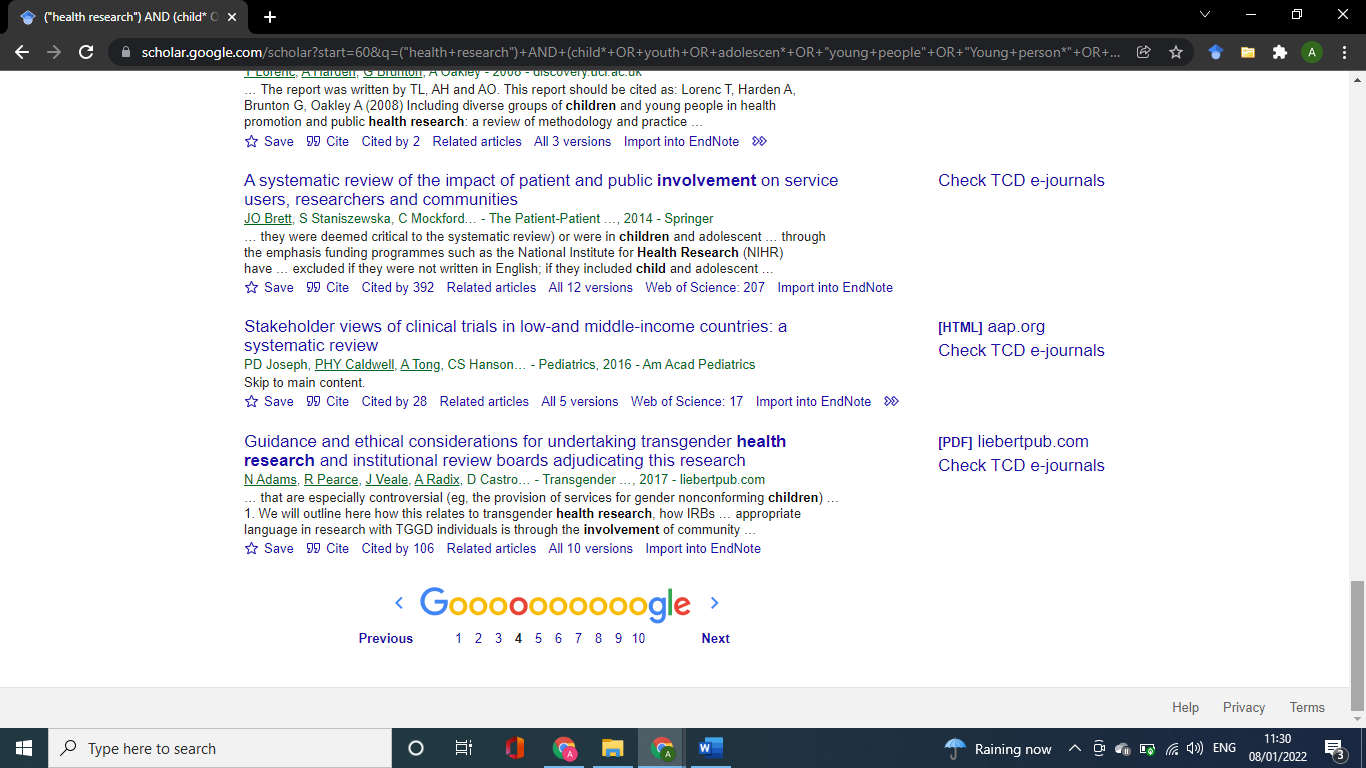 | | | | | | | | | | | | | | |
| Page 5 | | | | | | | | | | | | | | |
| 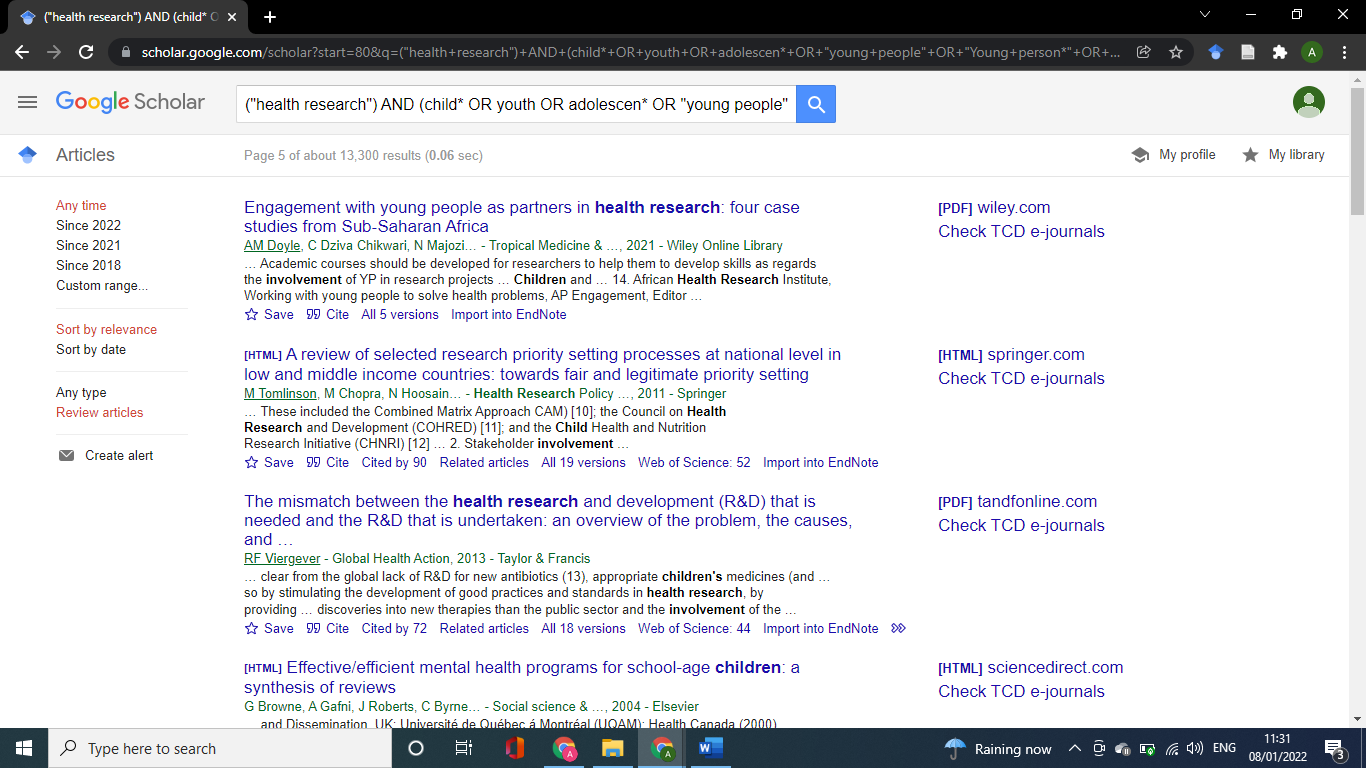 | | | | | | | | | | | | | | |
| 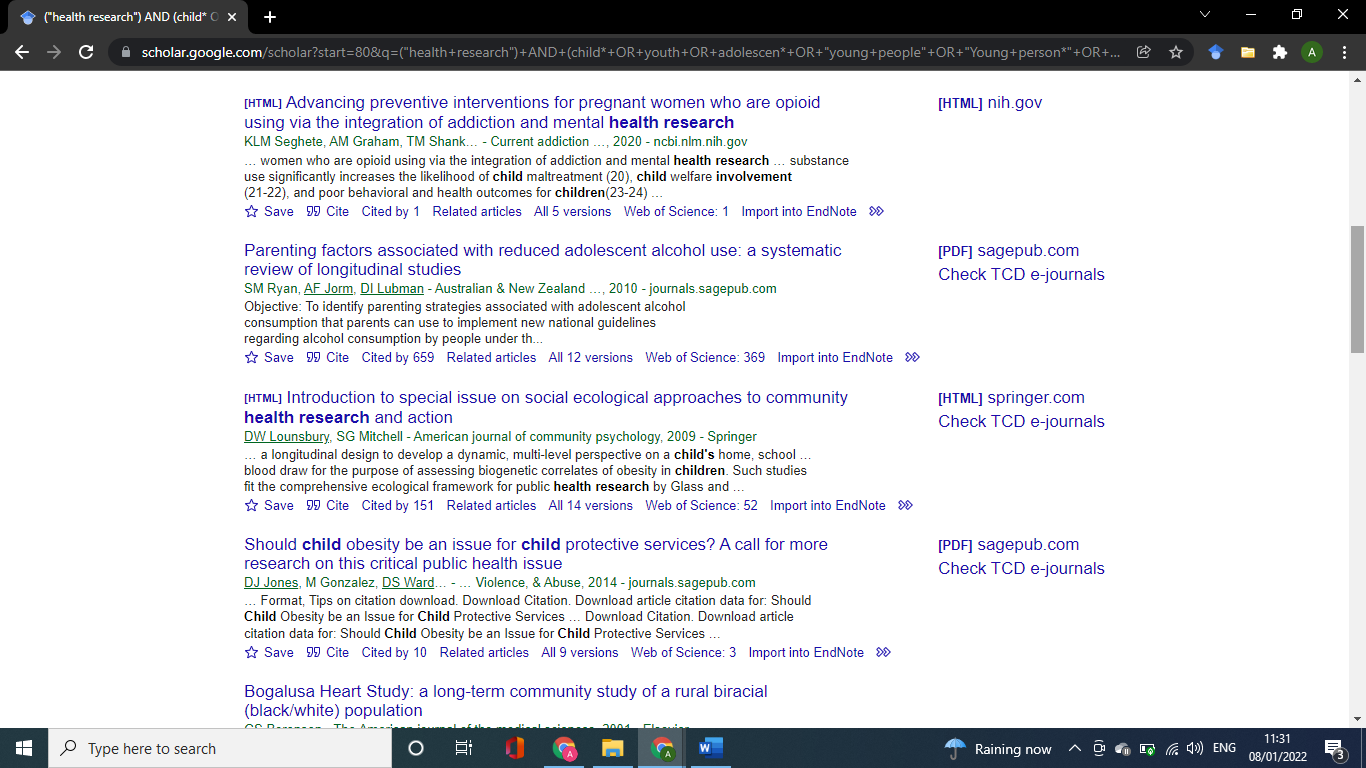 | | | | | | | | | | | | | | |
| 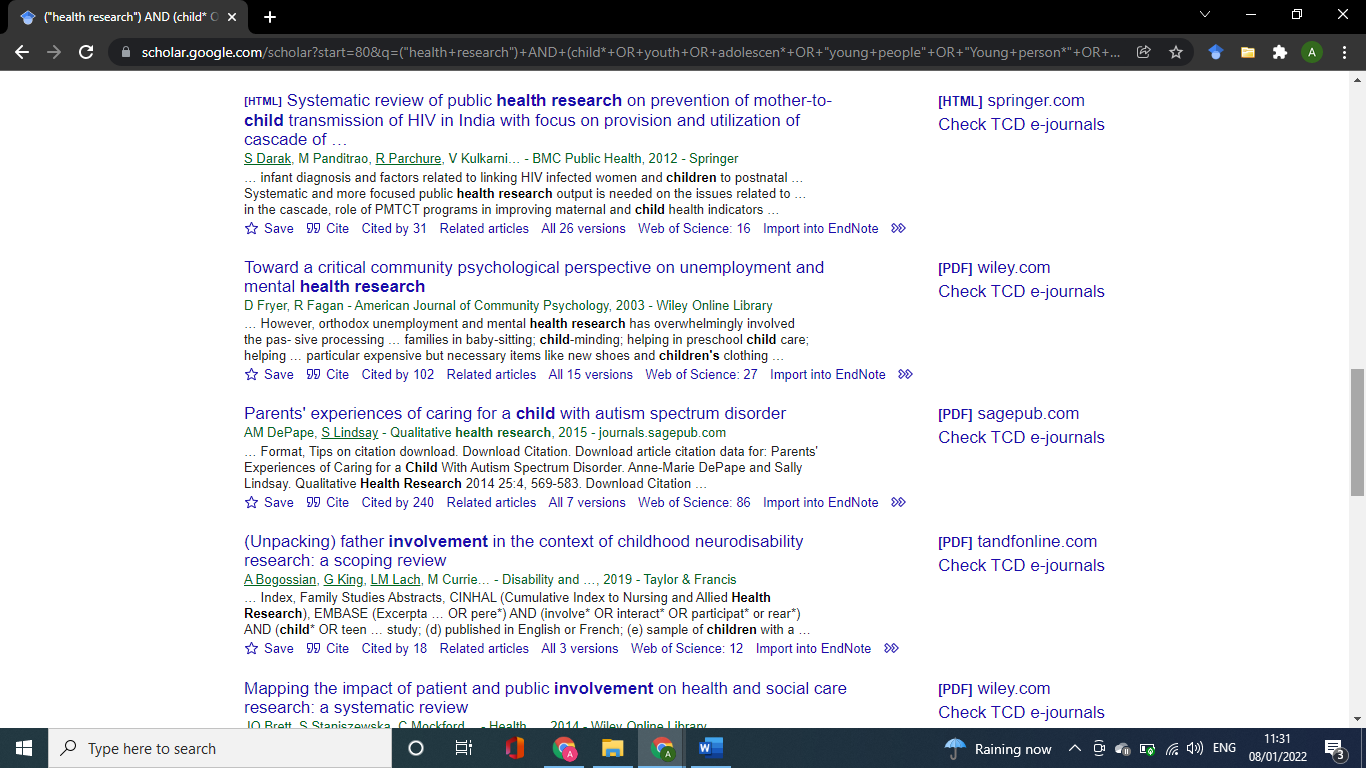 | | | | | | | | | | | | | | |
| 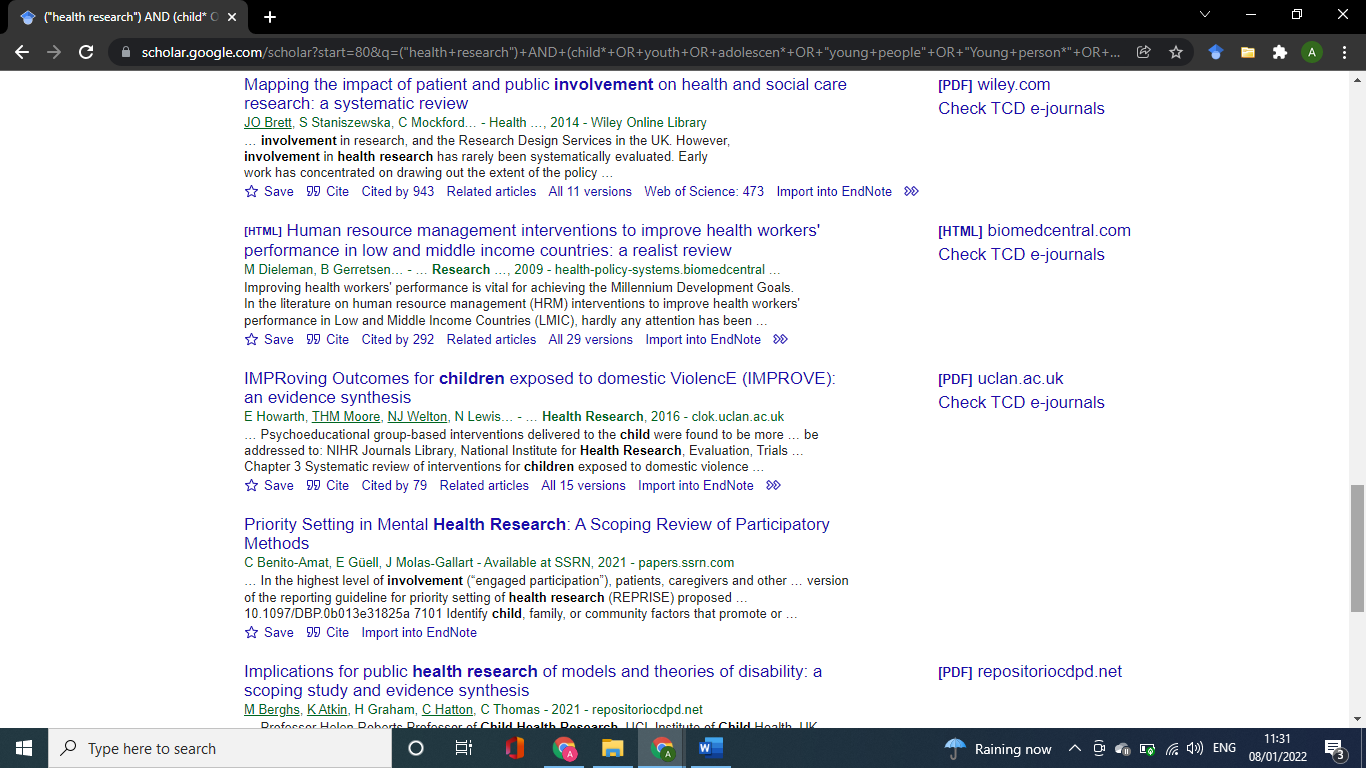 | | | | | | | | | | | | | | |
| 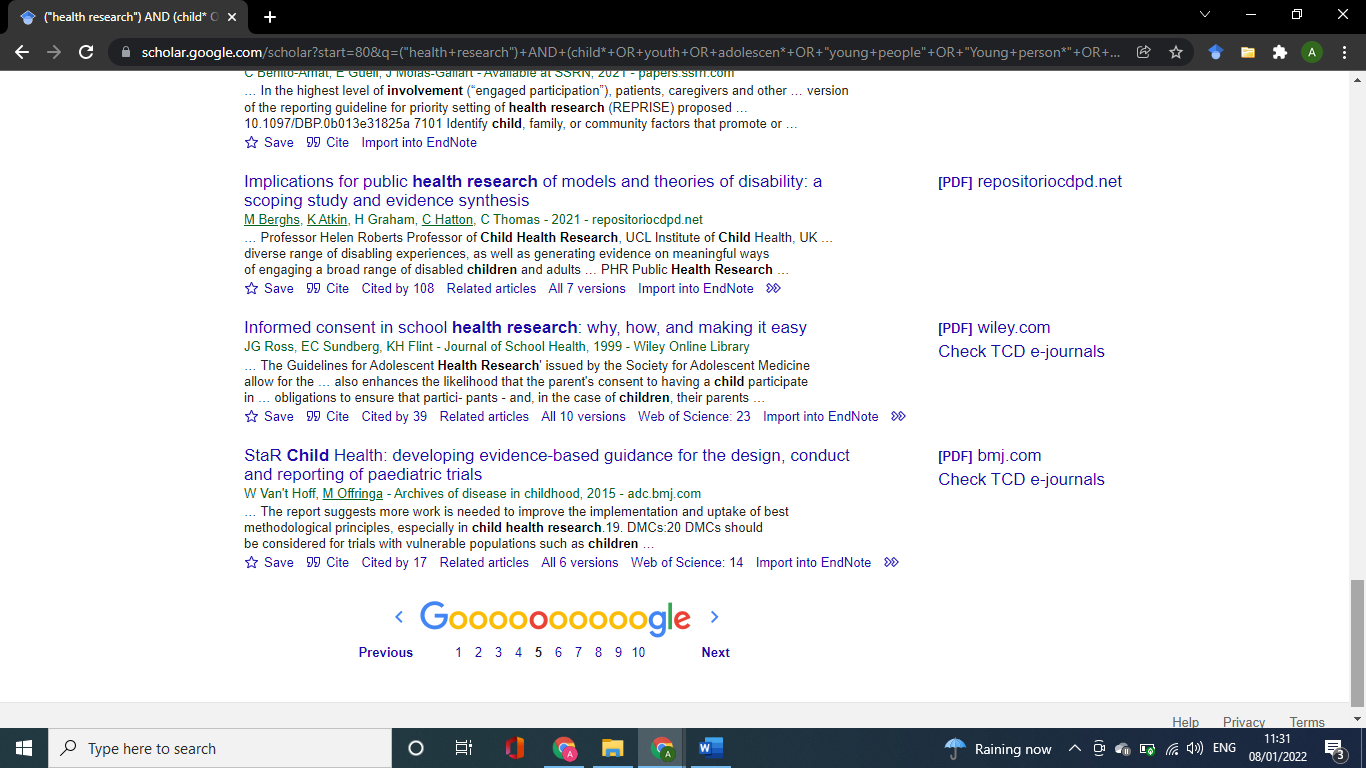 | | | | | | | | | | | | | | |
| Page 6 | | | | | | | | | | | | | | |
| 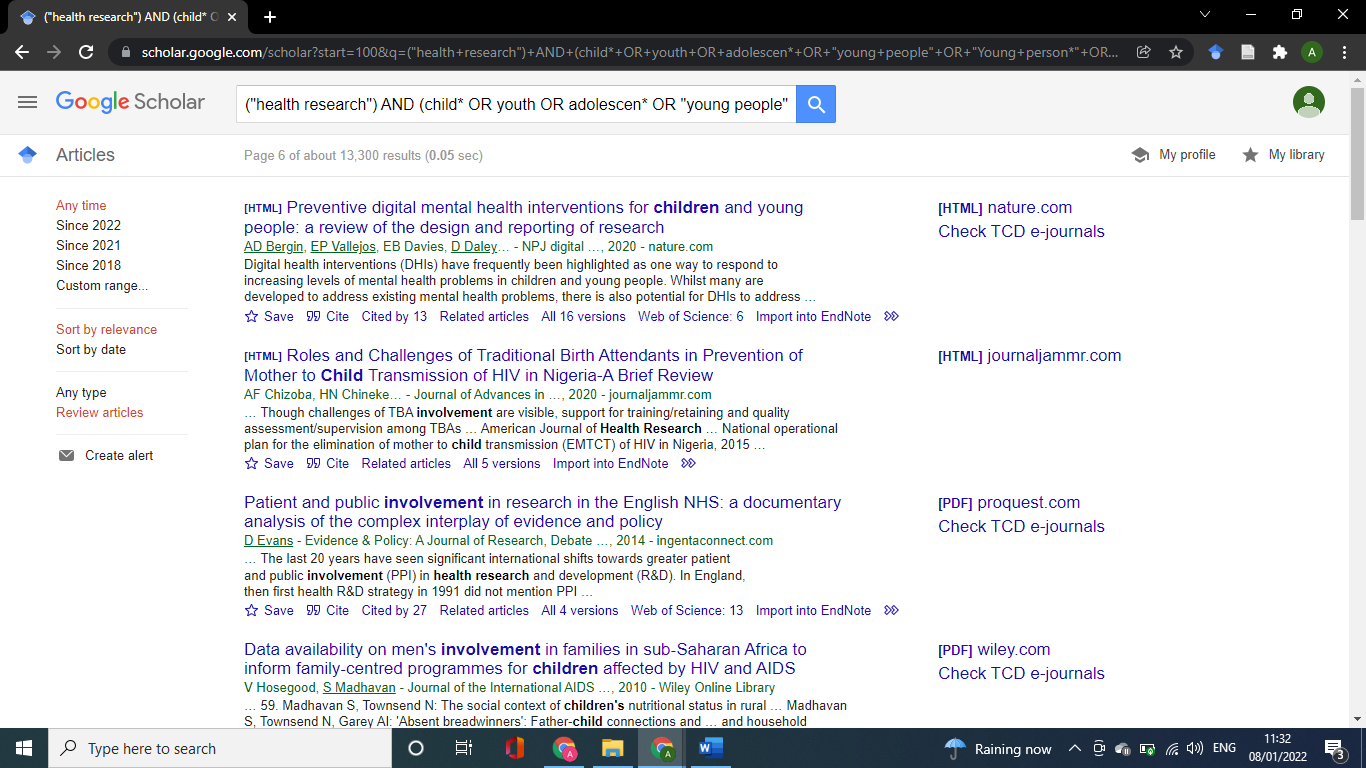 | | | | | | | | | | | | | | |
| 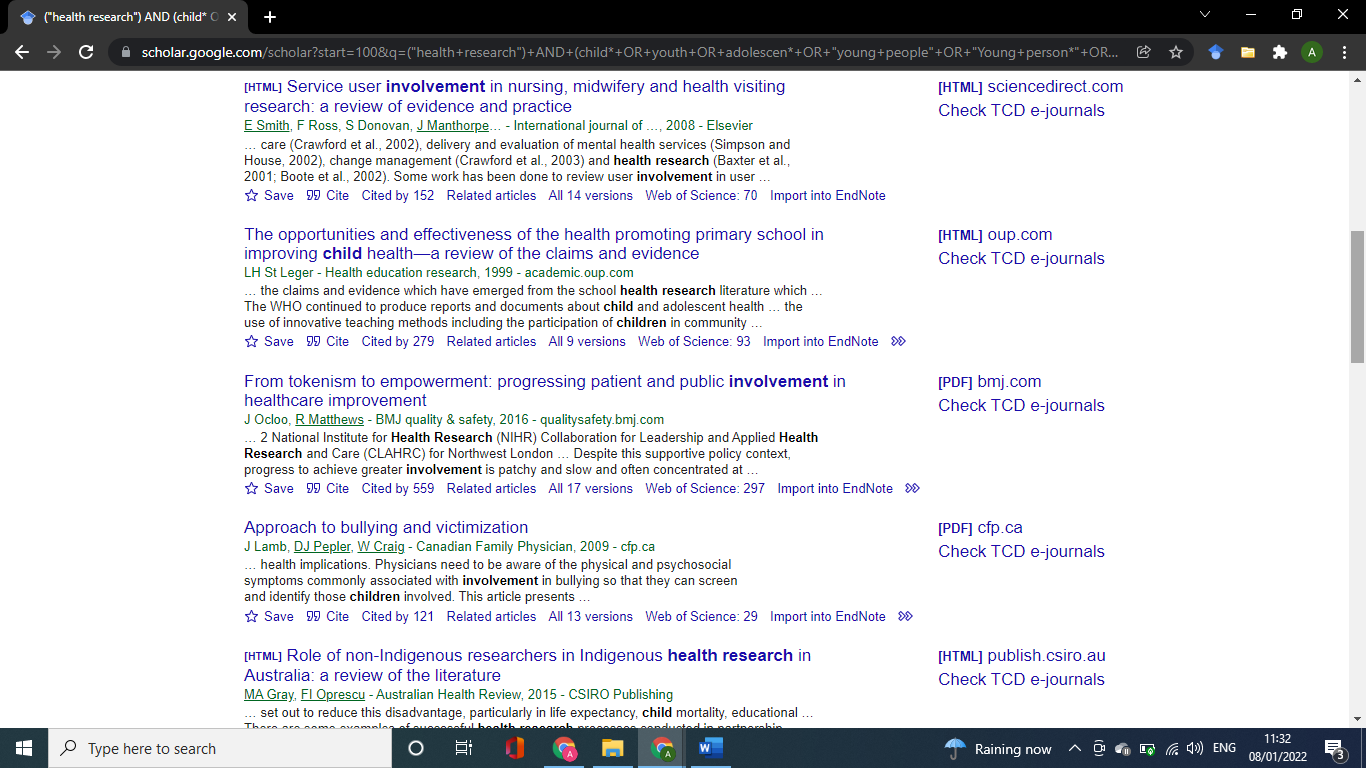 | | | | | | | | | | | | | | |
| 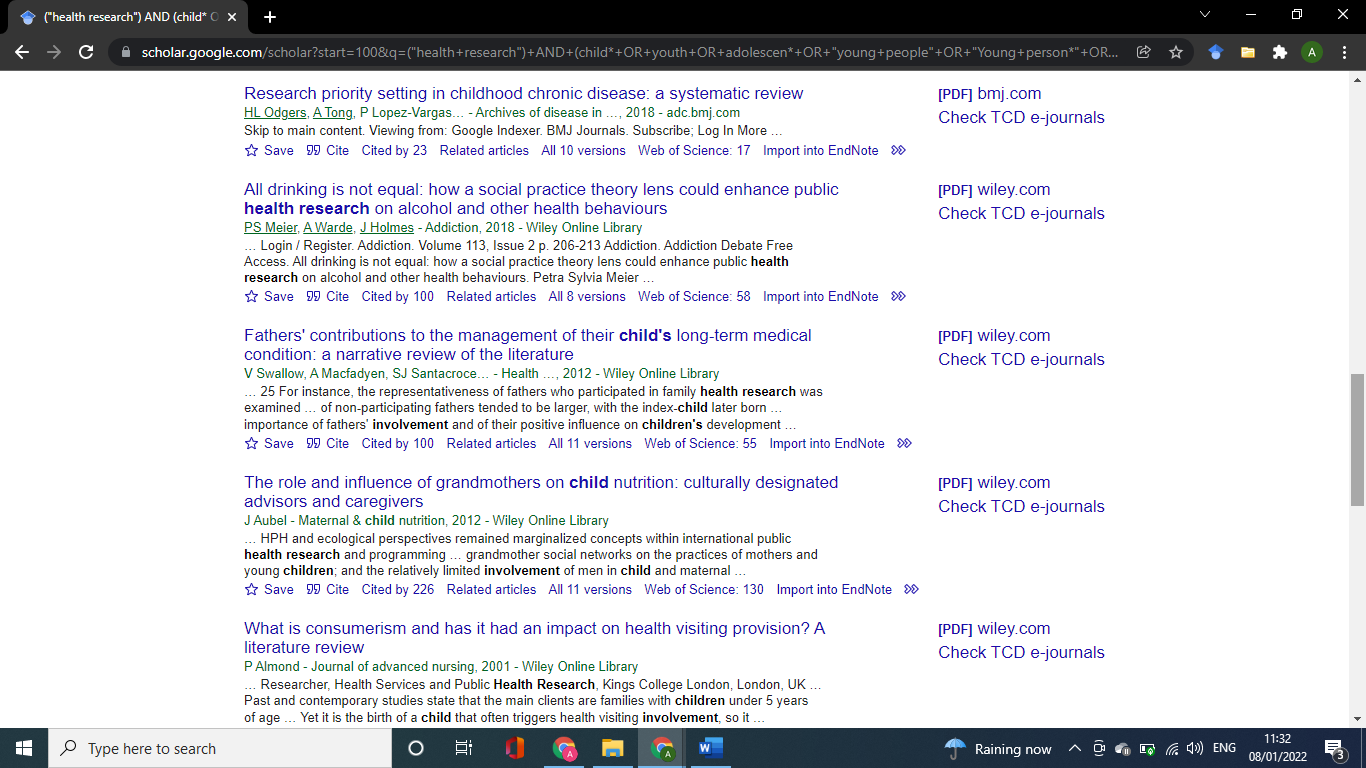 | | | | | | | | | | | | | | |
| 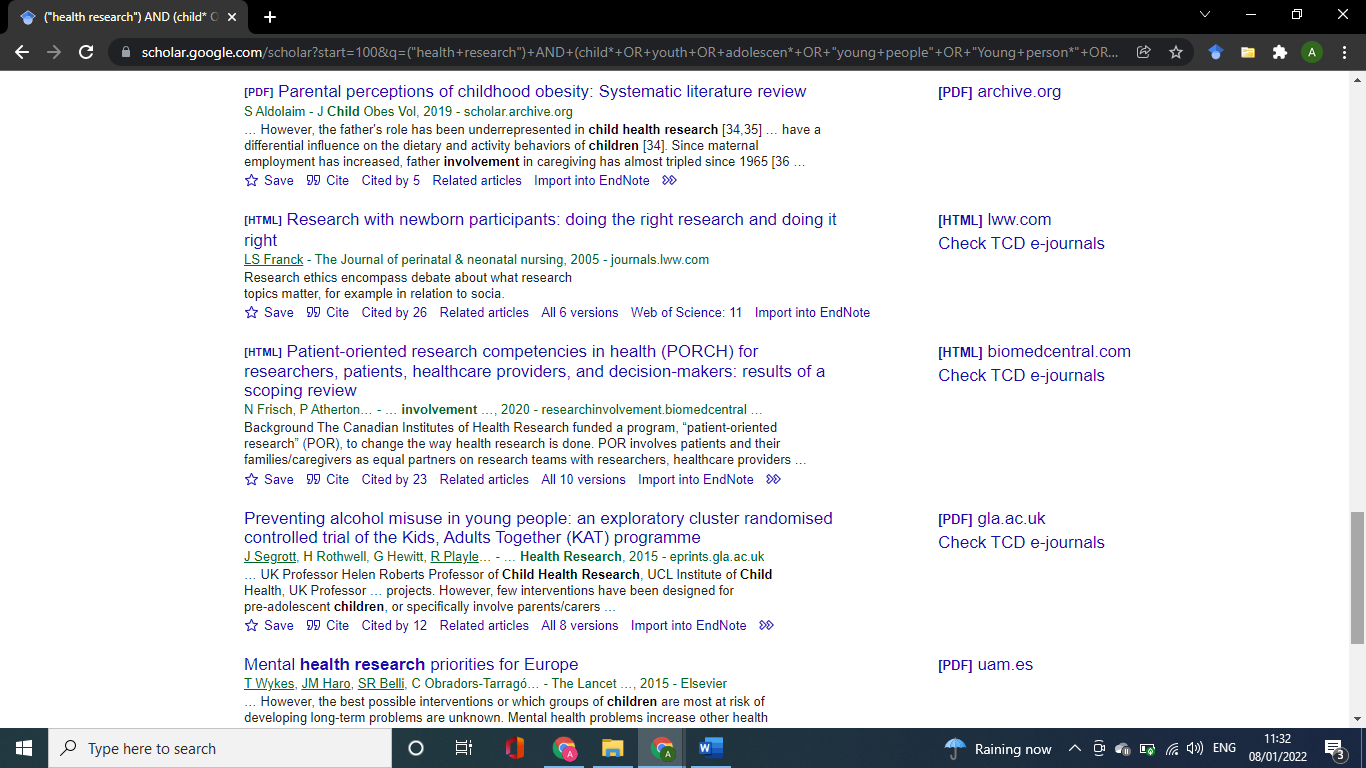 | | | | | | | | | | | | | | |
| 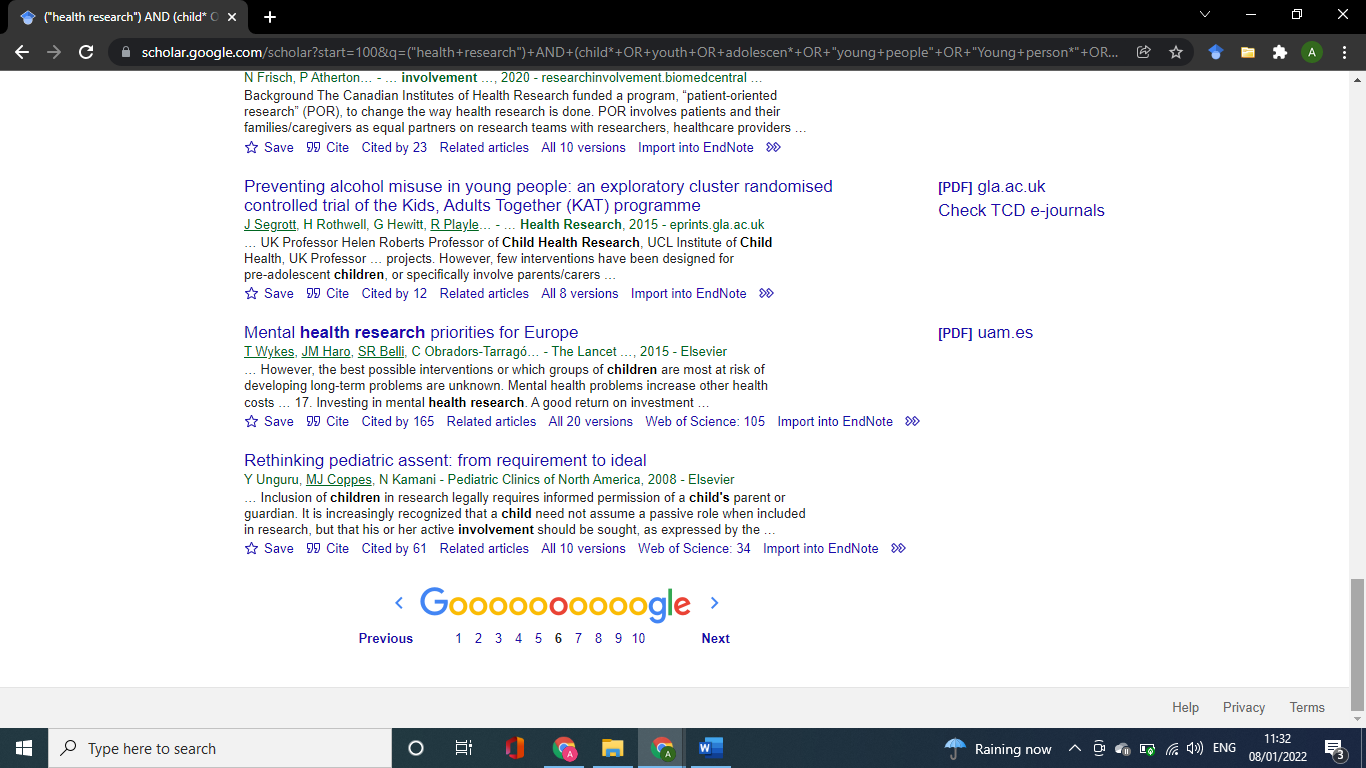 | | | | | | | | | | | | | | |
| Page 7 | | | | | | | | | | | | | | |
| 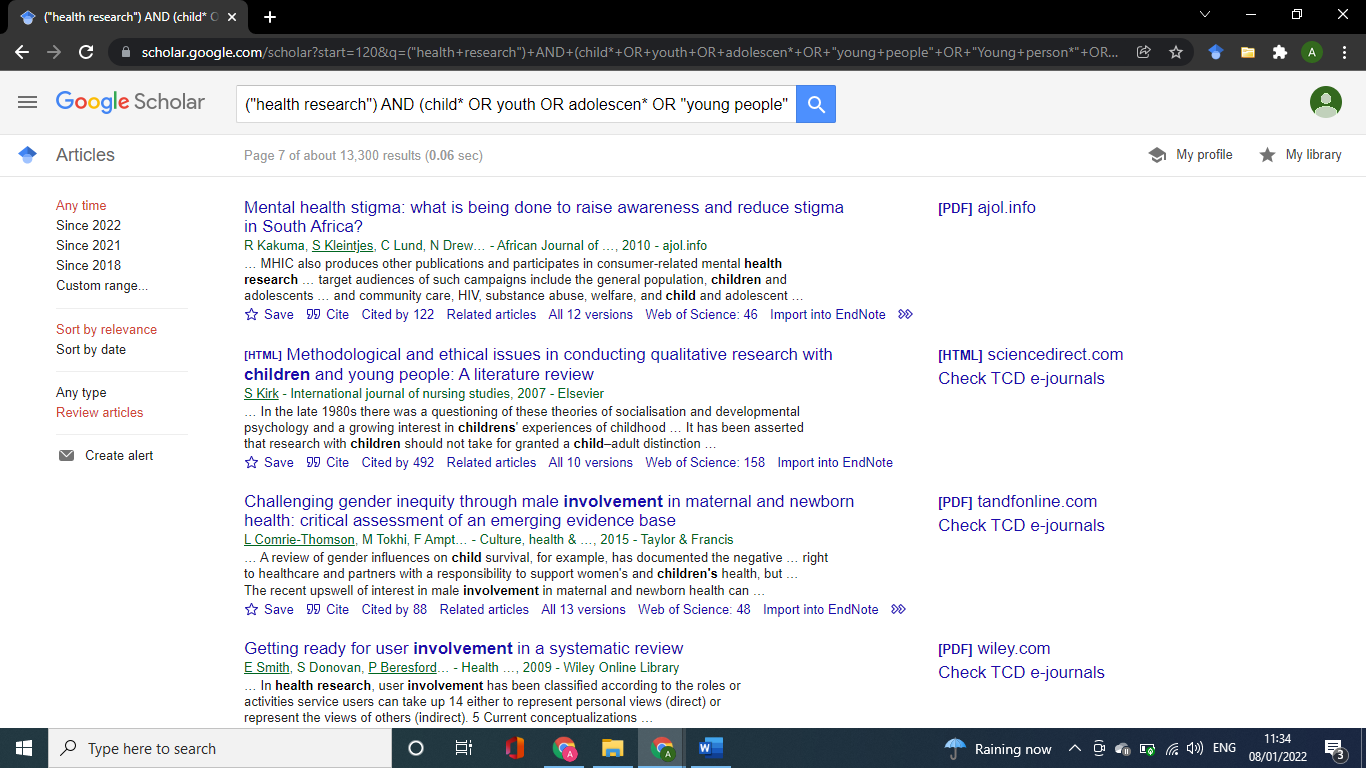 | | | | | | | | | | | | | | |
| 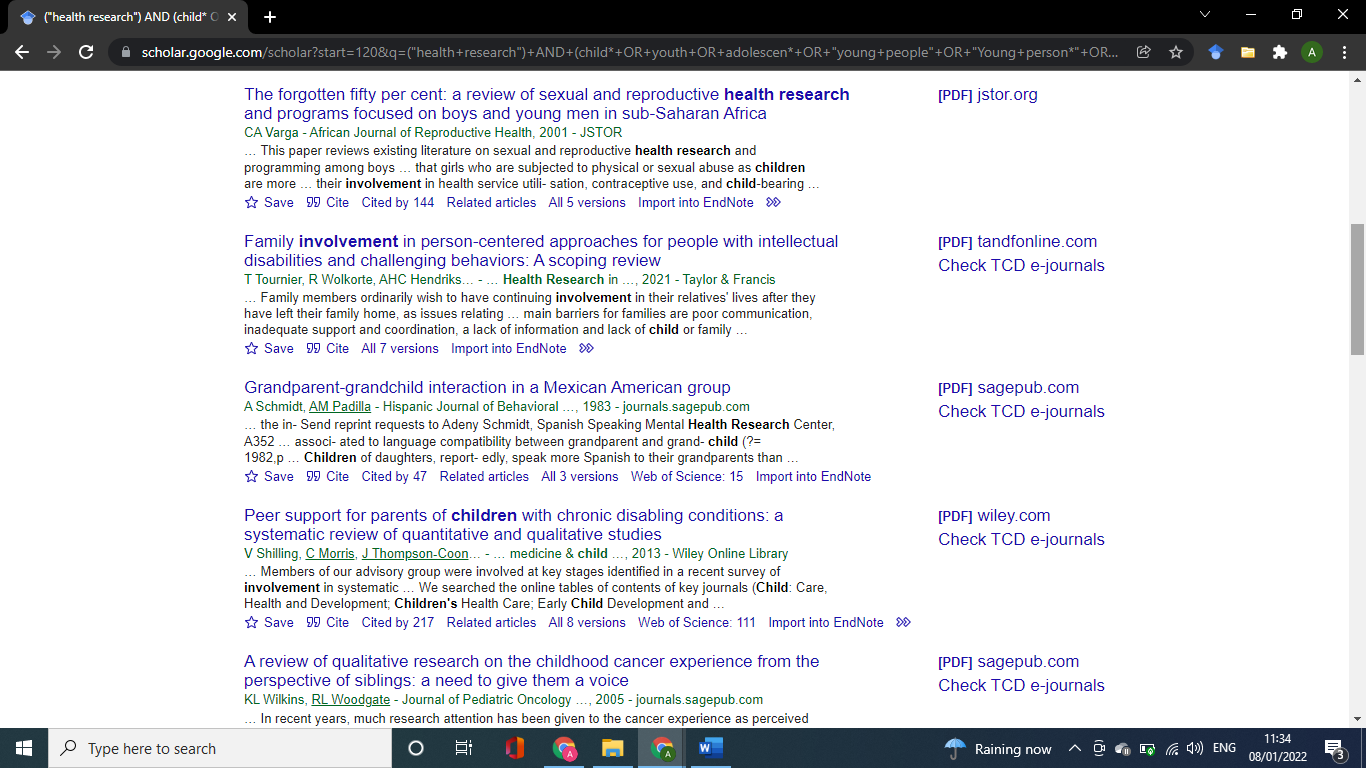 | | | | | | | | | | | | | | |
| 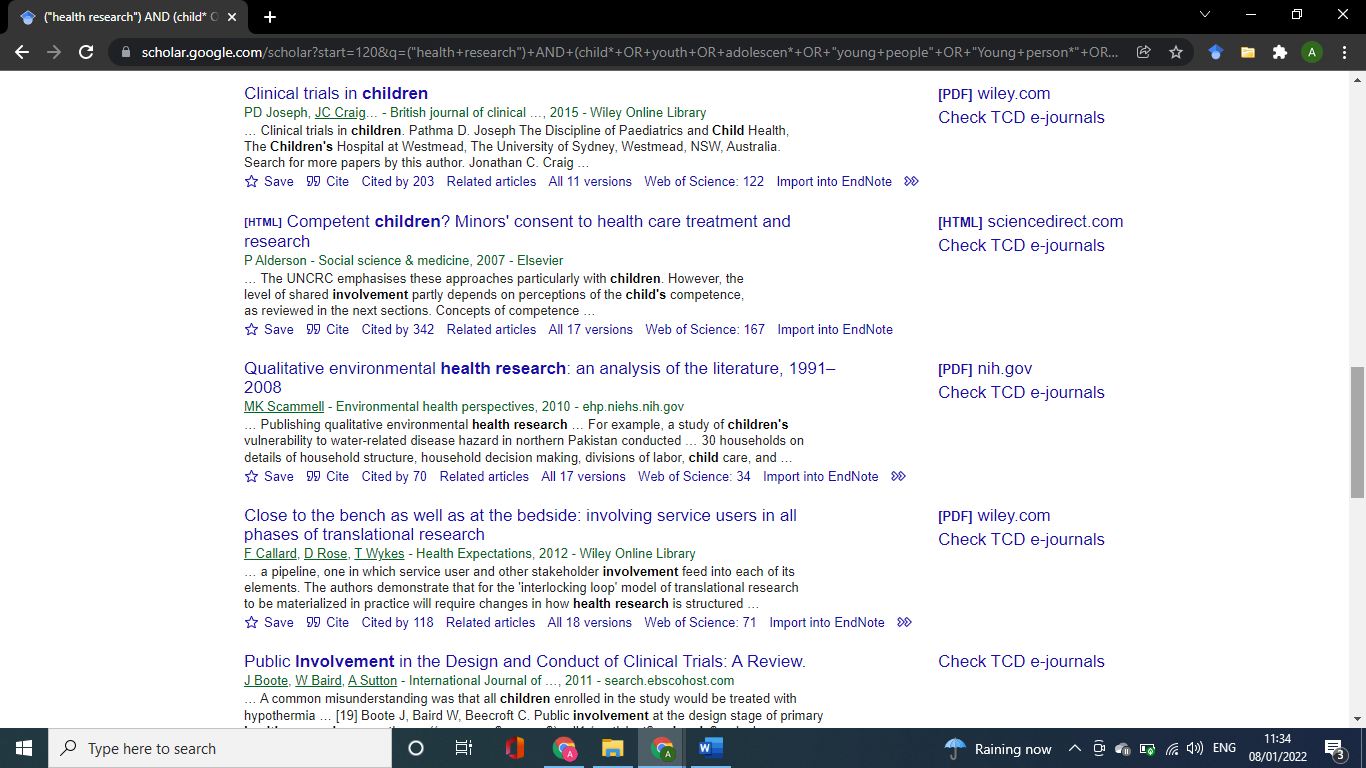 | | | | | | | | | | | | | | |
| 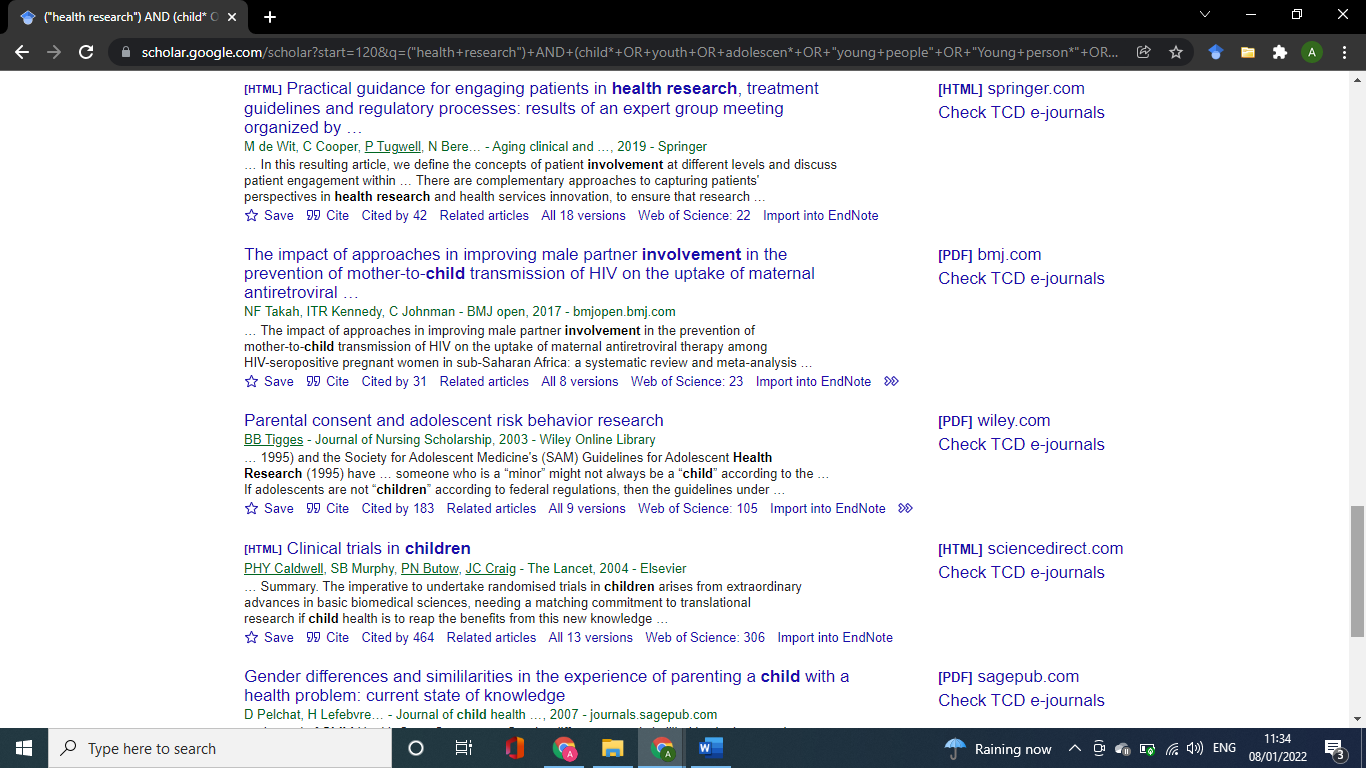 | | | | | | | | | | | | | | |
| 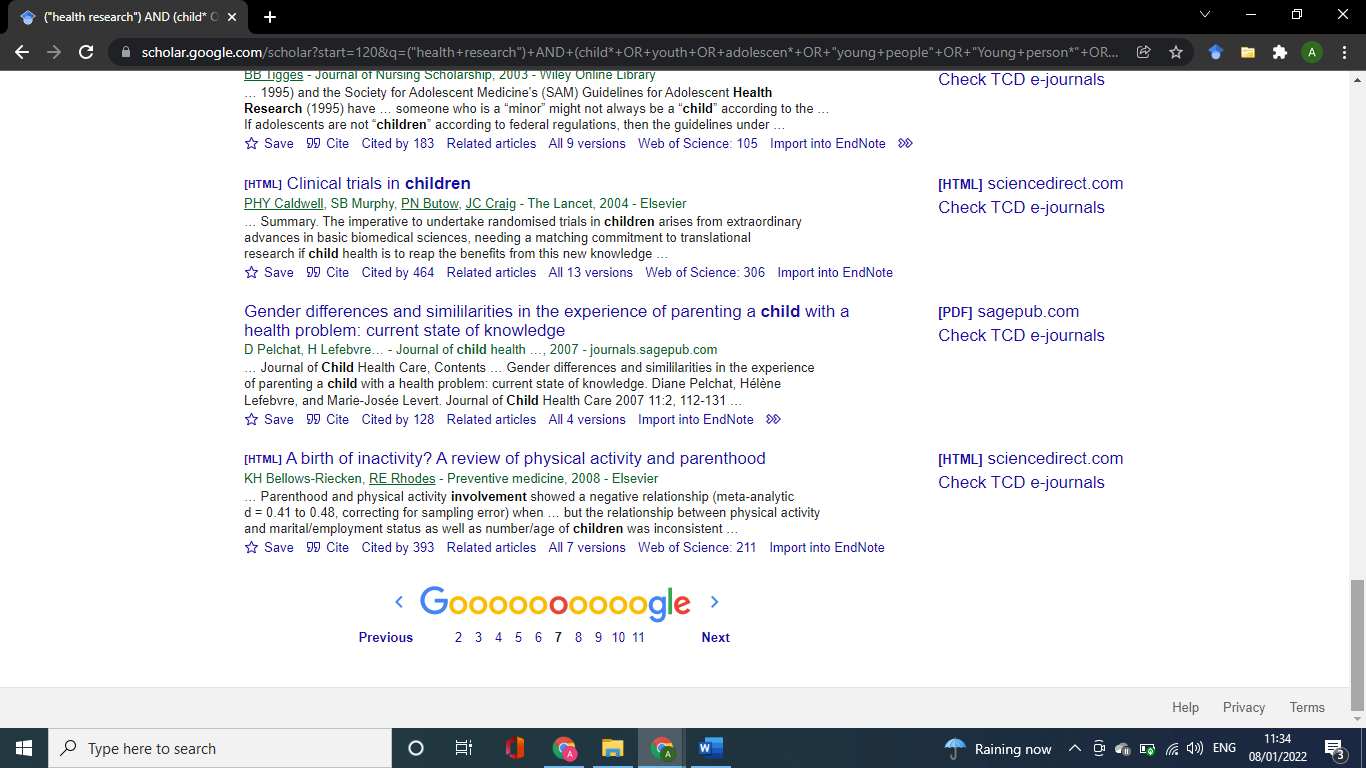 | | | | | | | | | | | | | | |
| Page 8 | | | | | | | | | | | | | | |
| 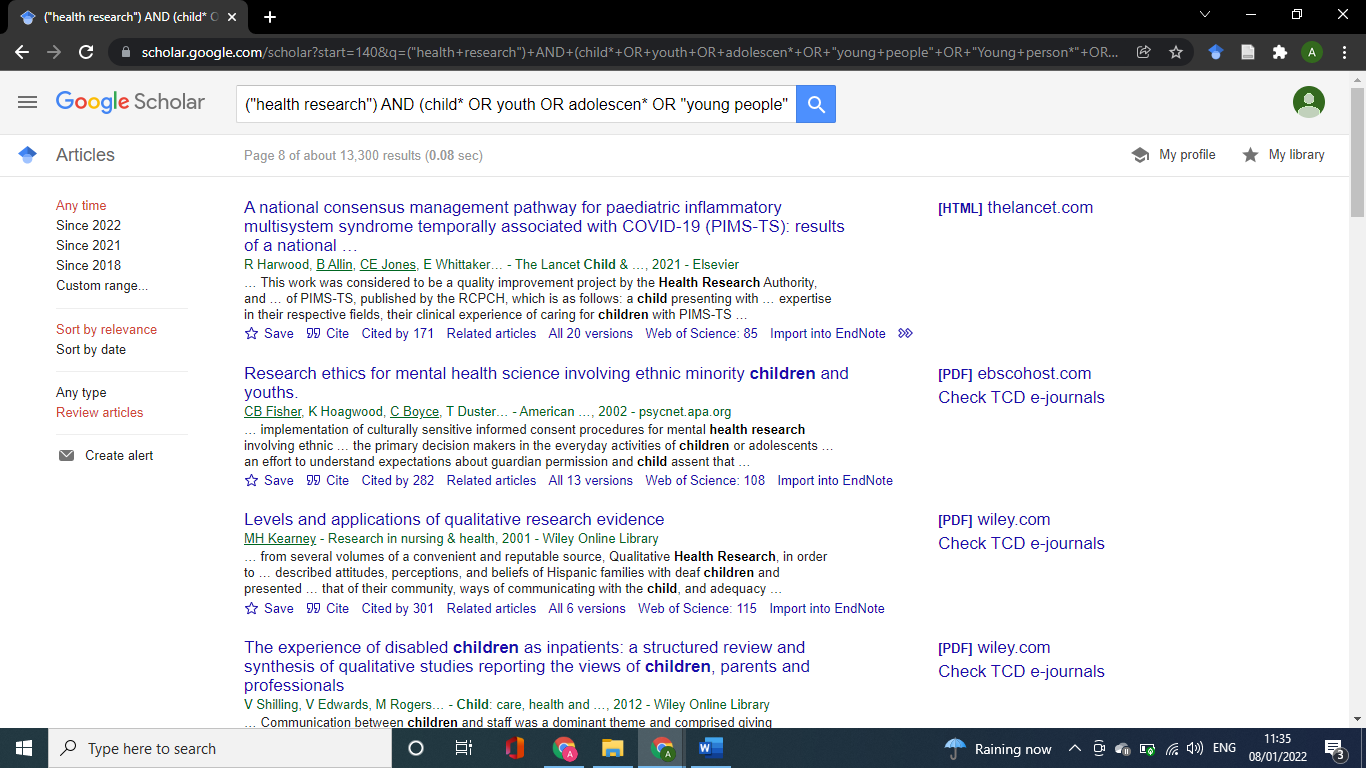 | | | | | | | | | | | | | | |
| 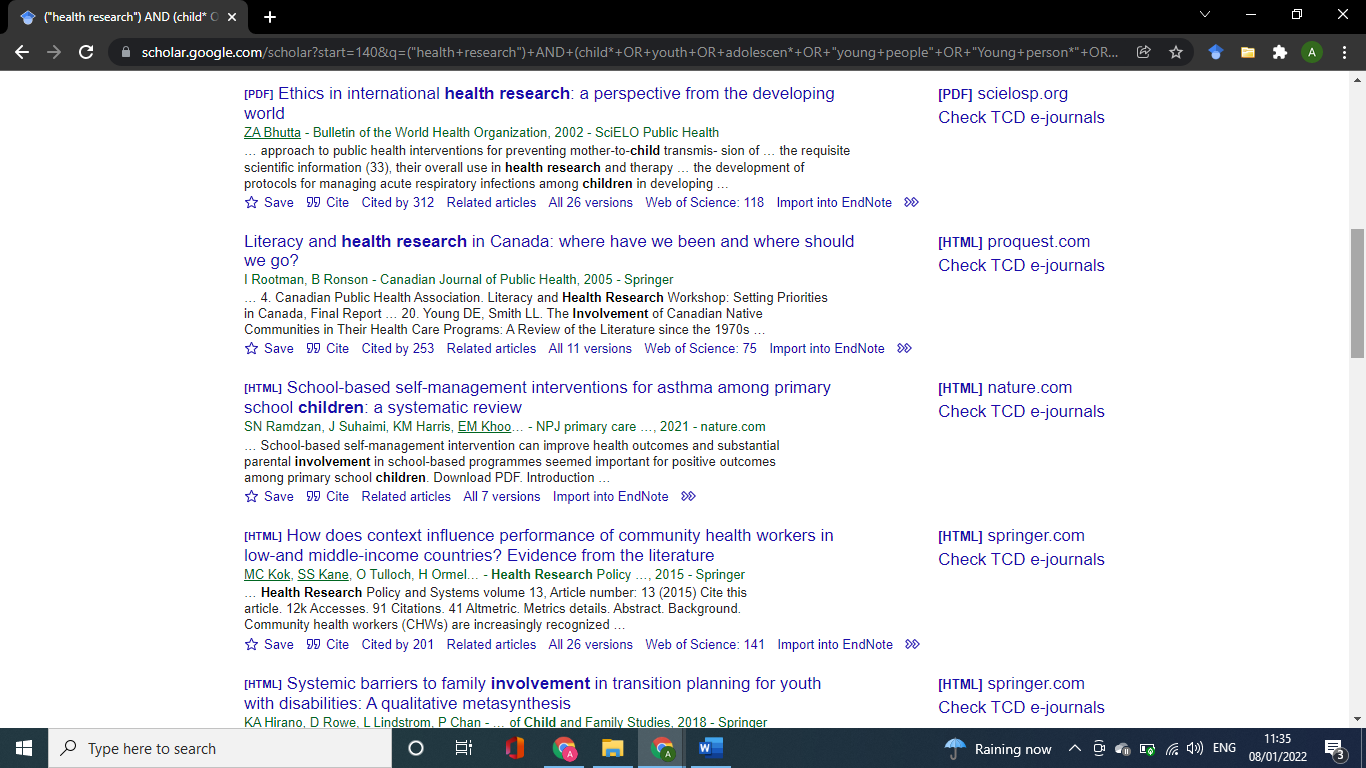 | | | | | | | | | | | | | | |
| 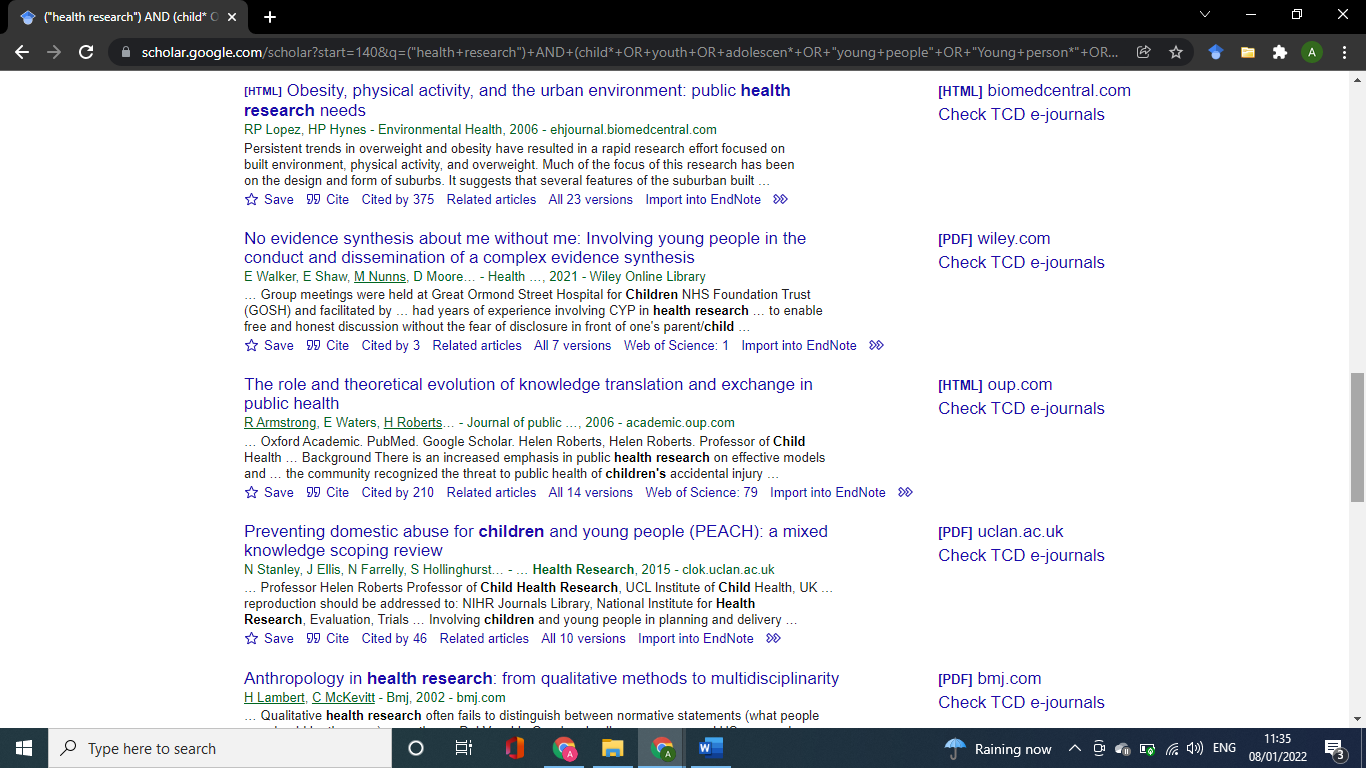 | | | | | | | | | | | | | | |
| 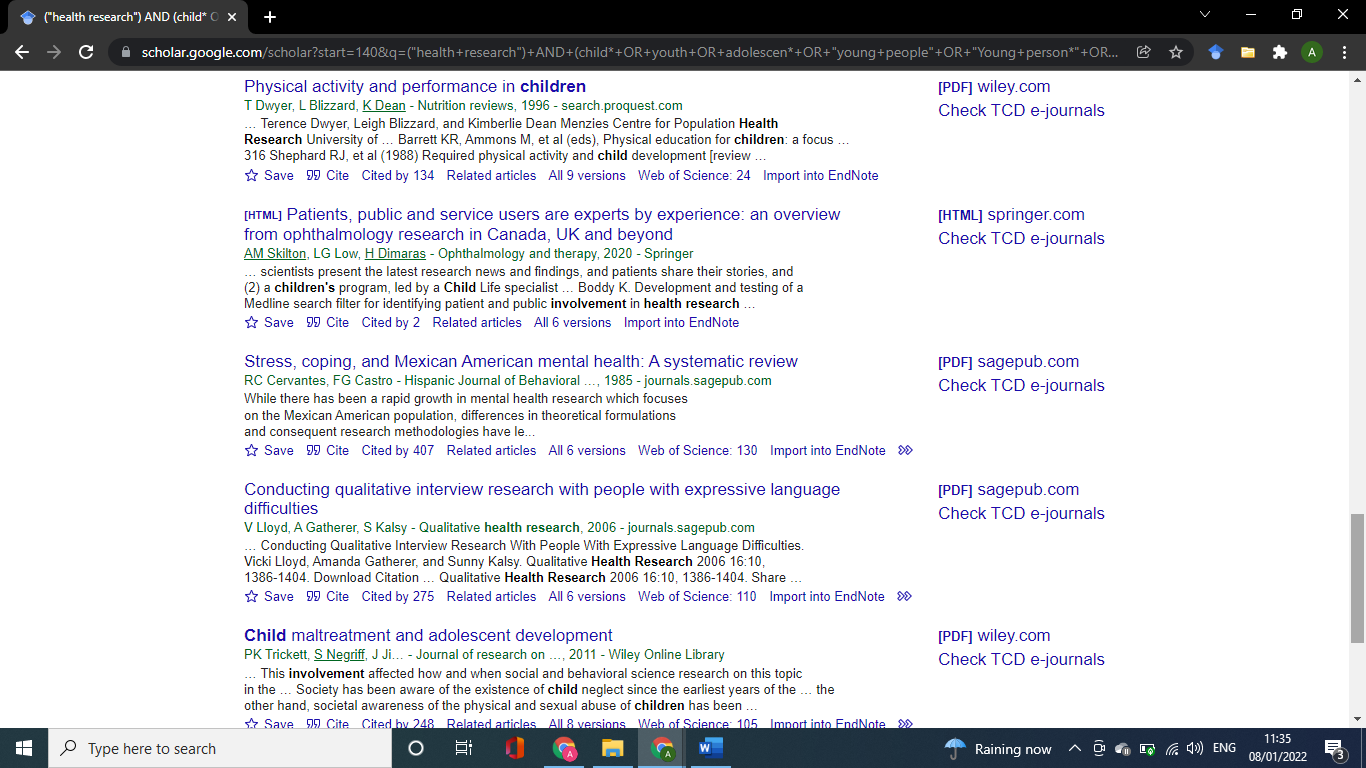 | | | | | | | | | | | | | | |
| 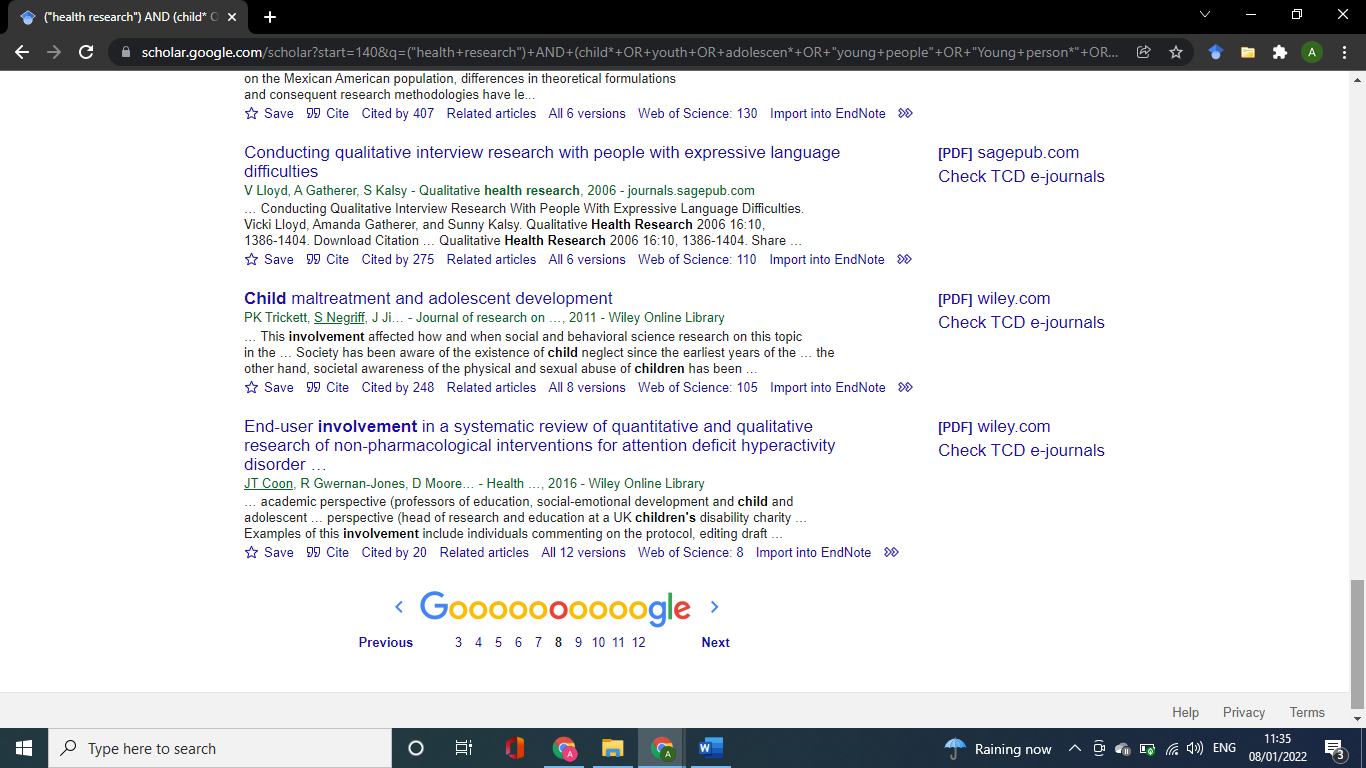 | | | | | | | | | | | | | | |
| Page 9 | | | | | | | | | | | | | | |
| 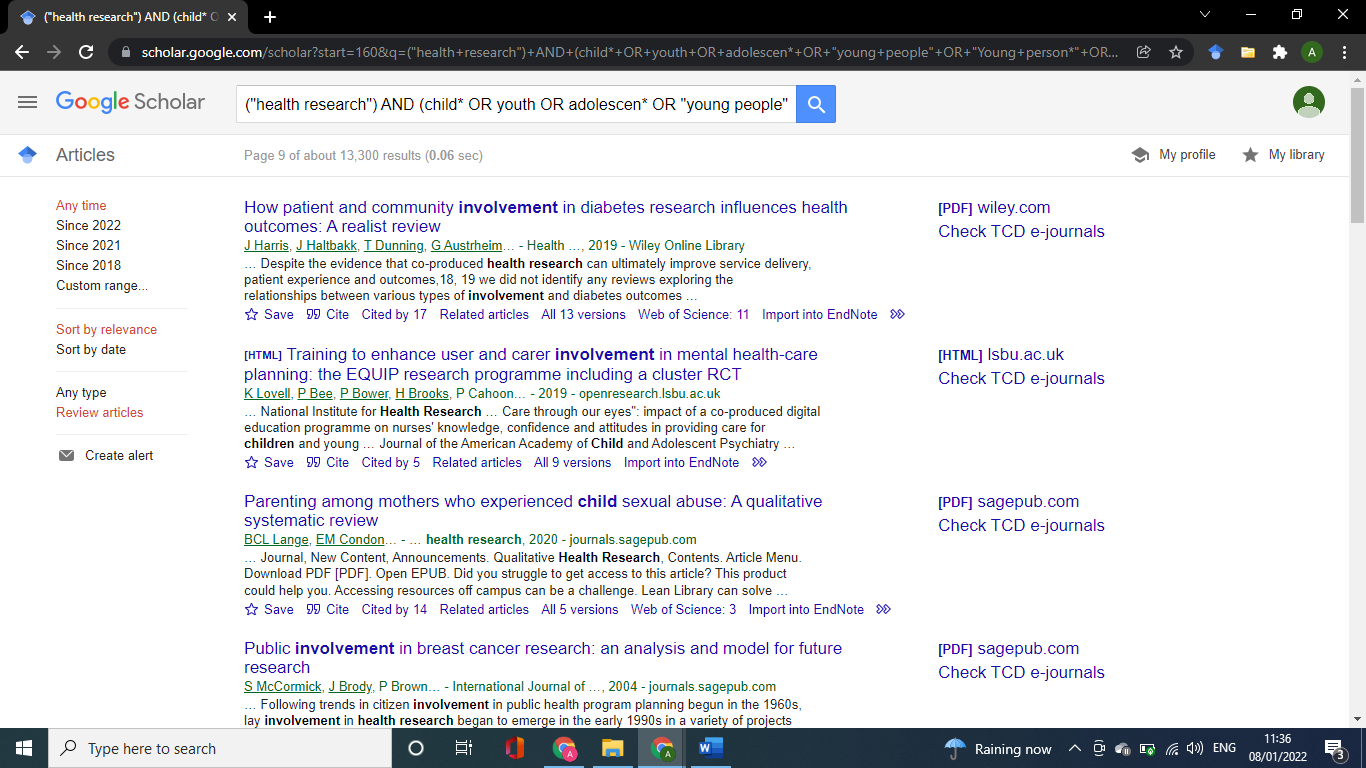 | | | | | | | | | | | | | | |
| 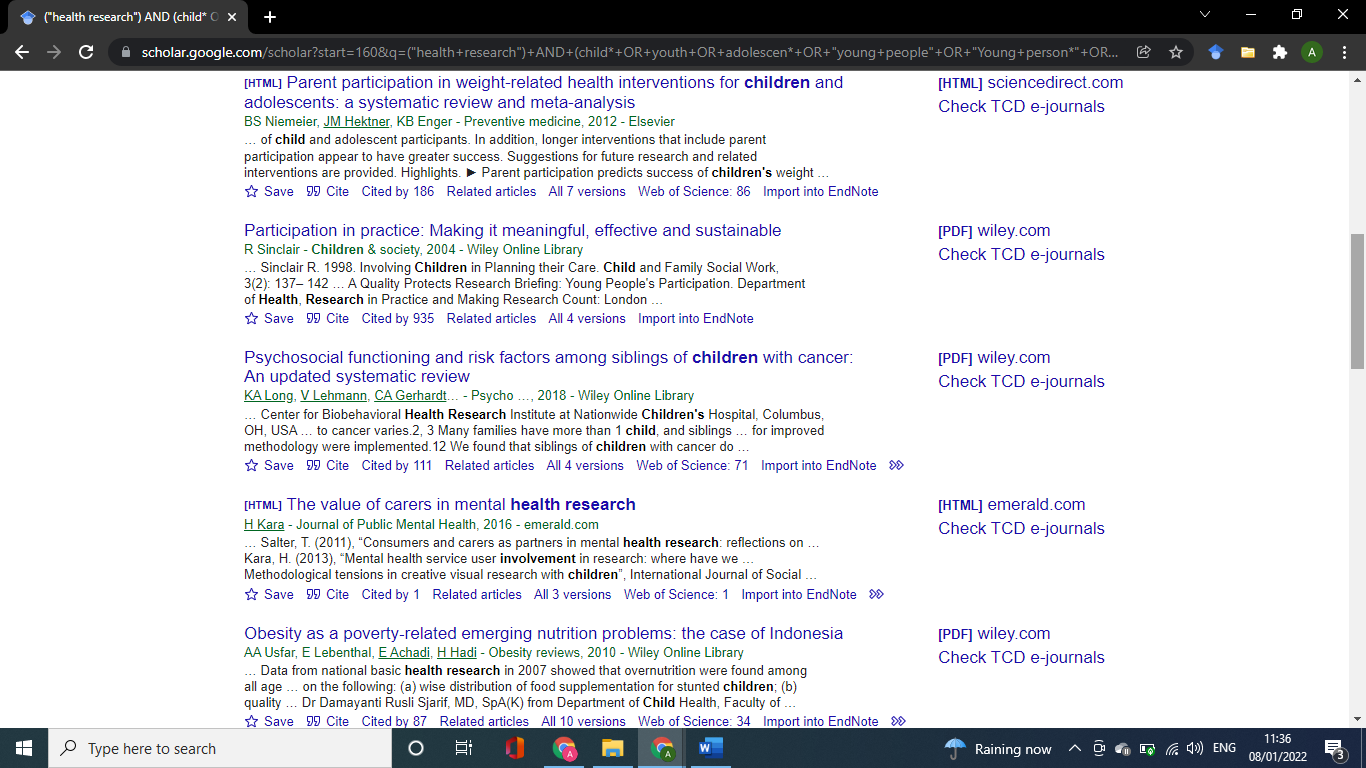 | | | | | | | | | | | | | | |
| 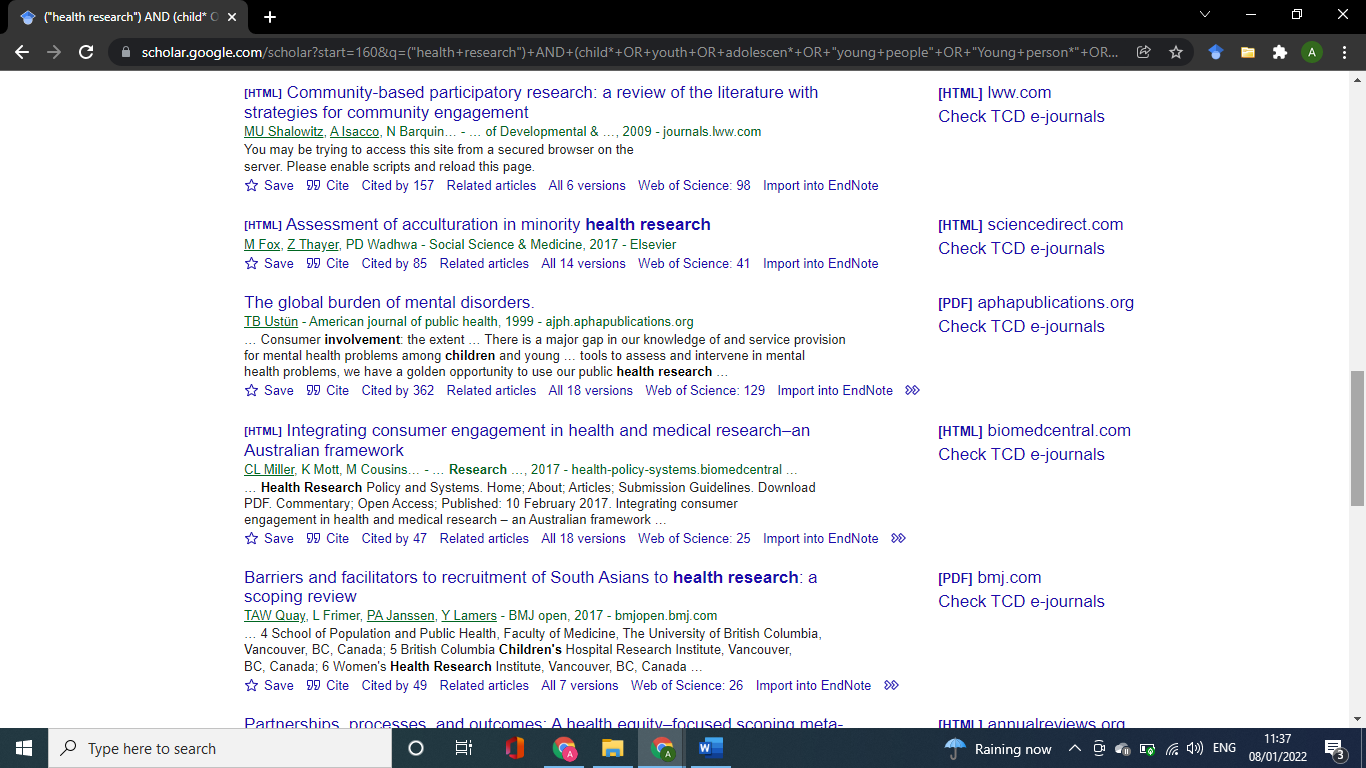 | | | | | | | | | | | | | | |
| 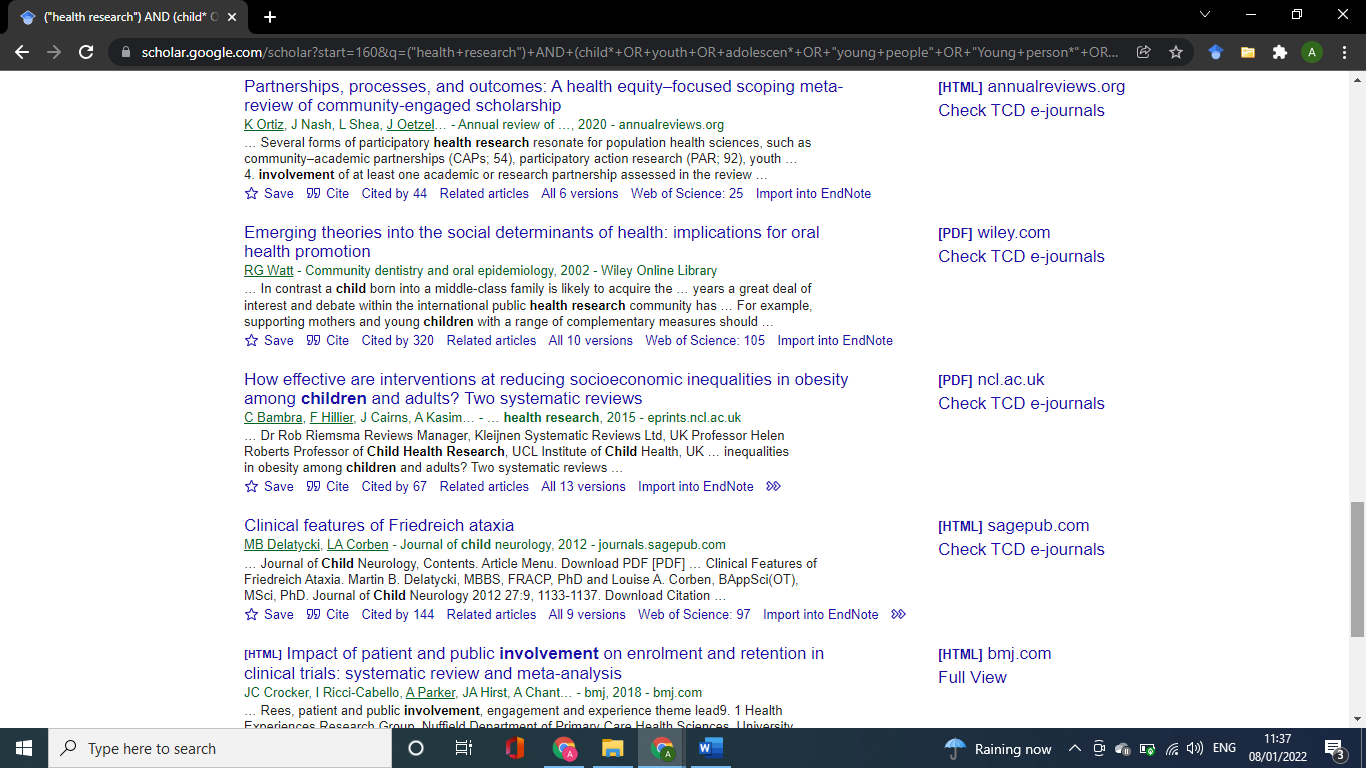 | | | | | | | | | | | | | | |
| 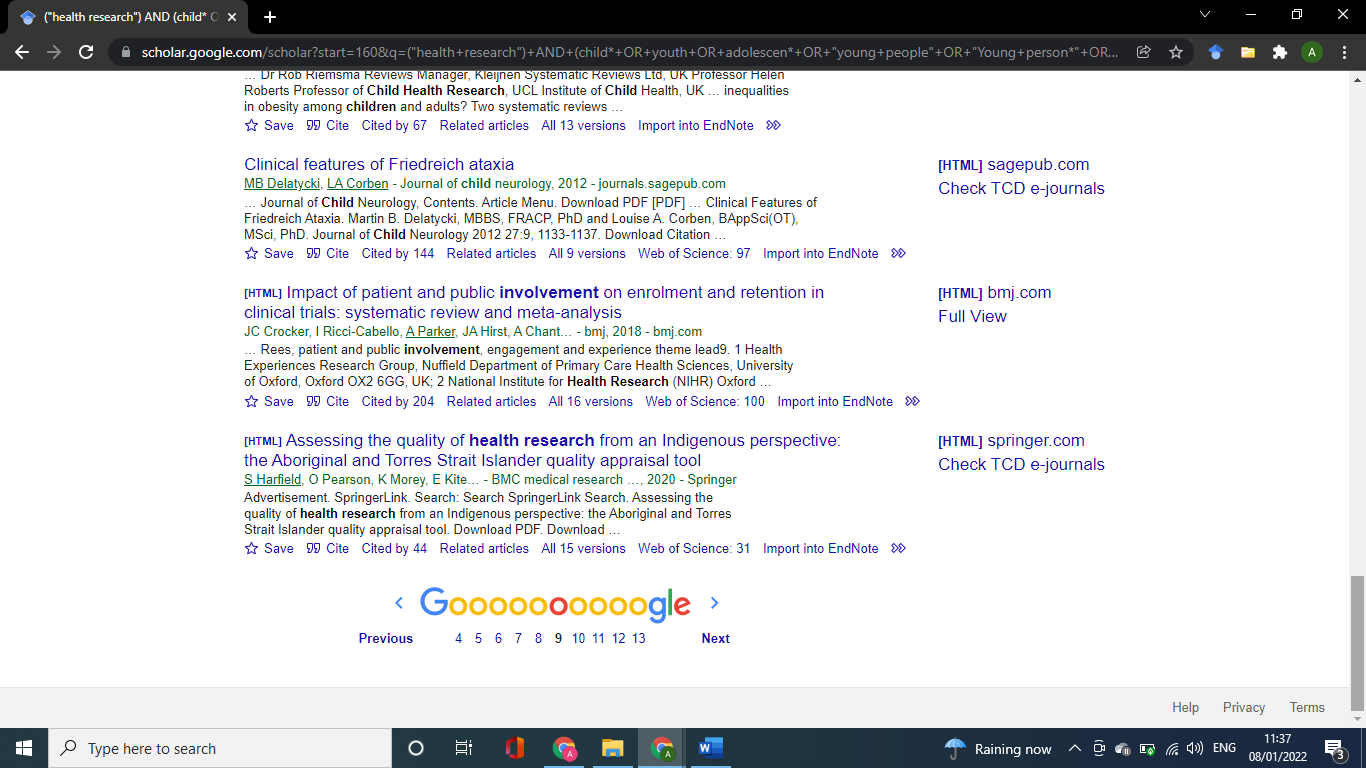 | | | | | | | | | | | | | | |
| Page 10 | | | | | | | | | | | | | | |
| 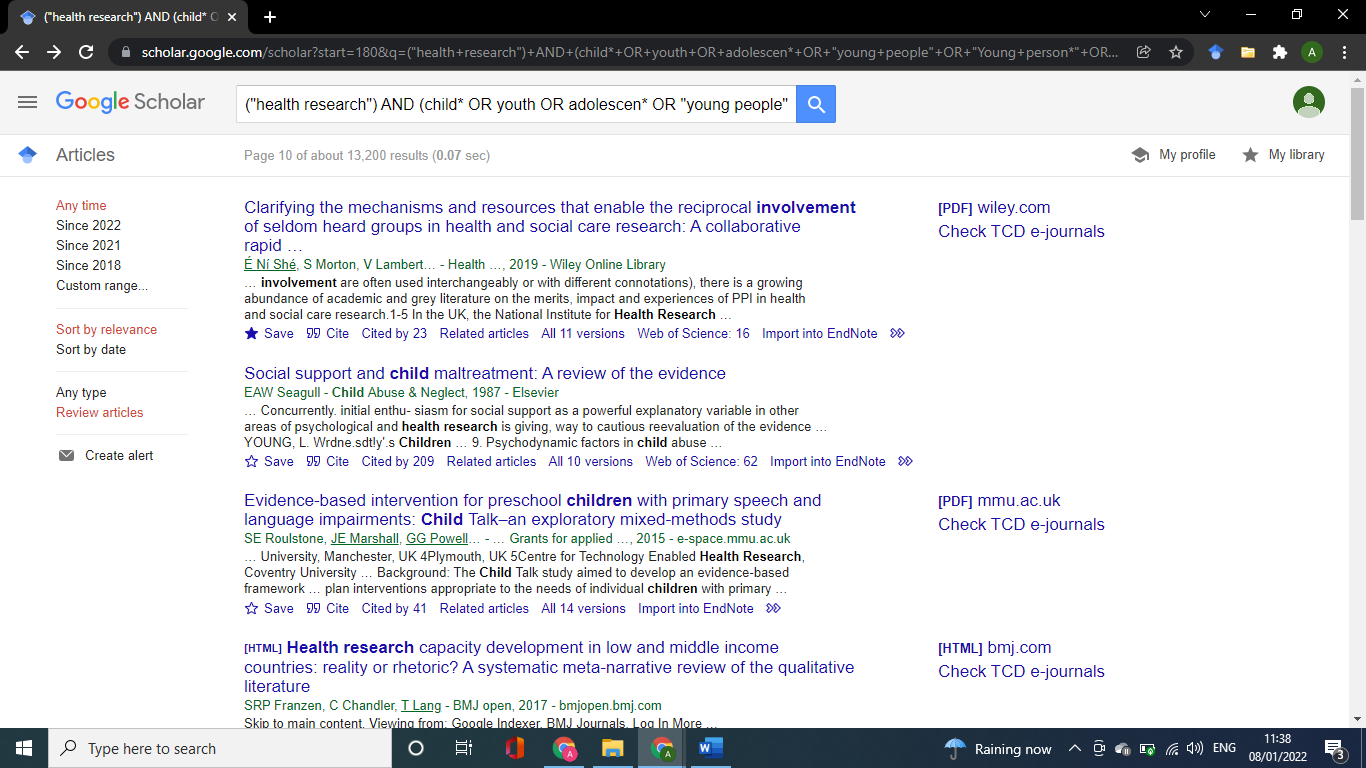 | | | | | | | | | | | | | | |
| 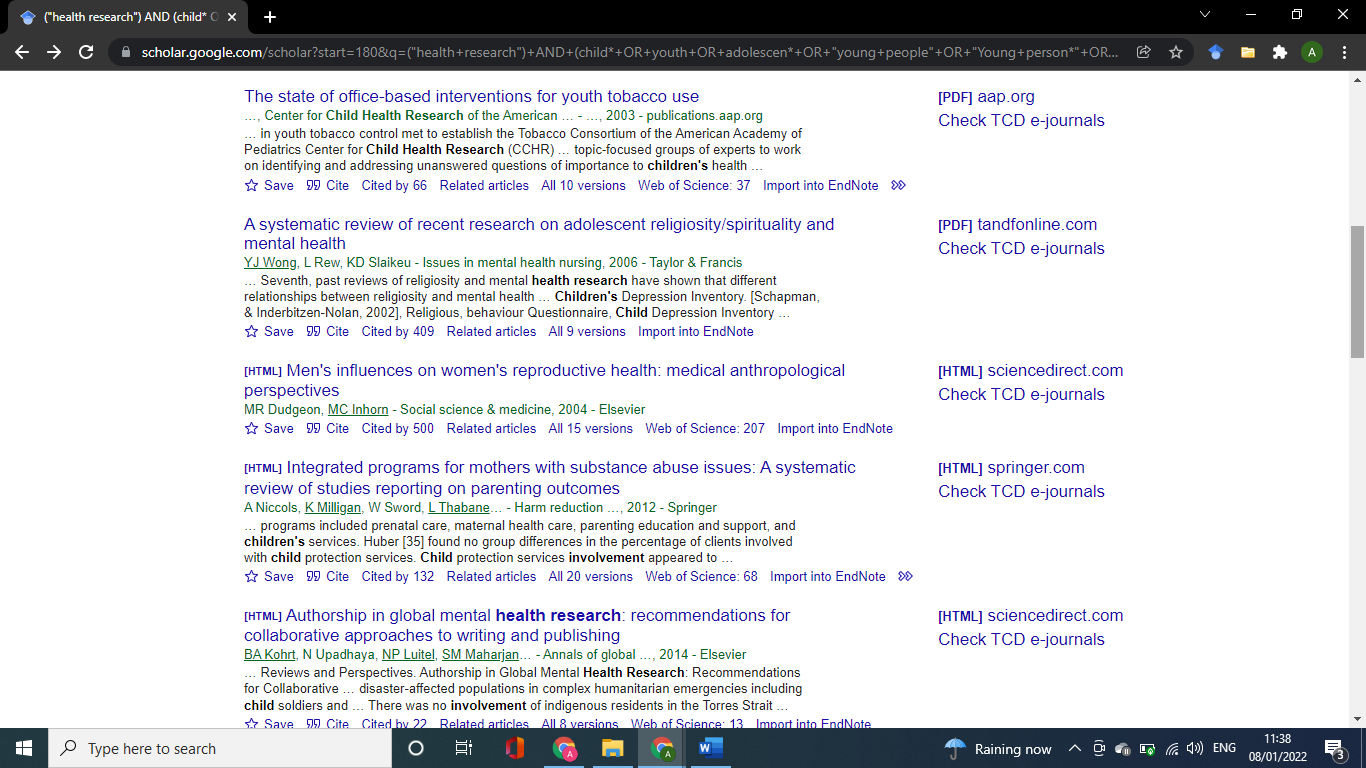 | | | | | | | | | | | | | | |
| 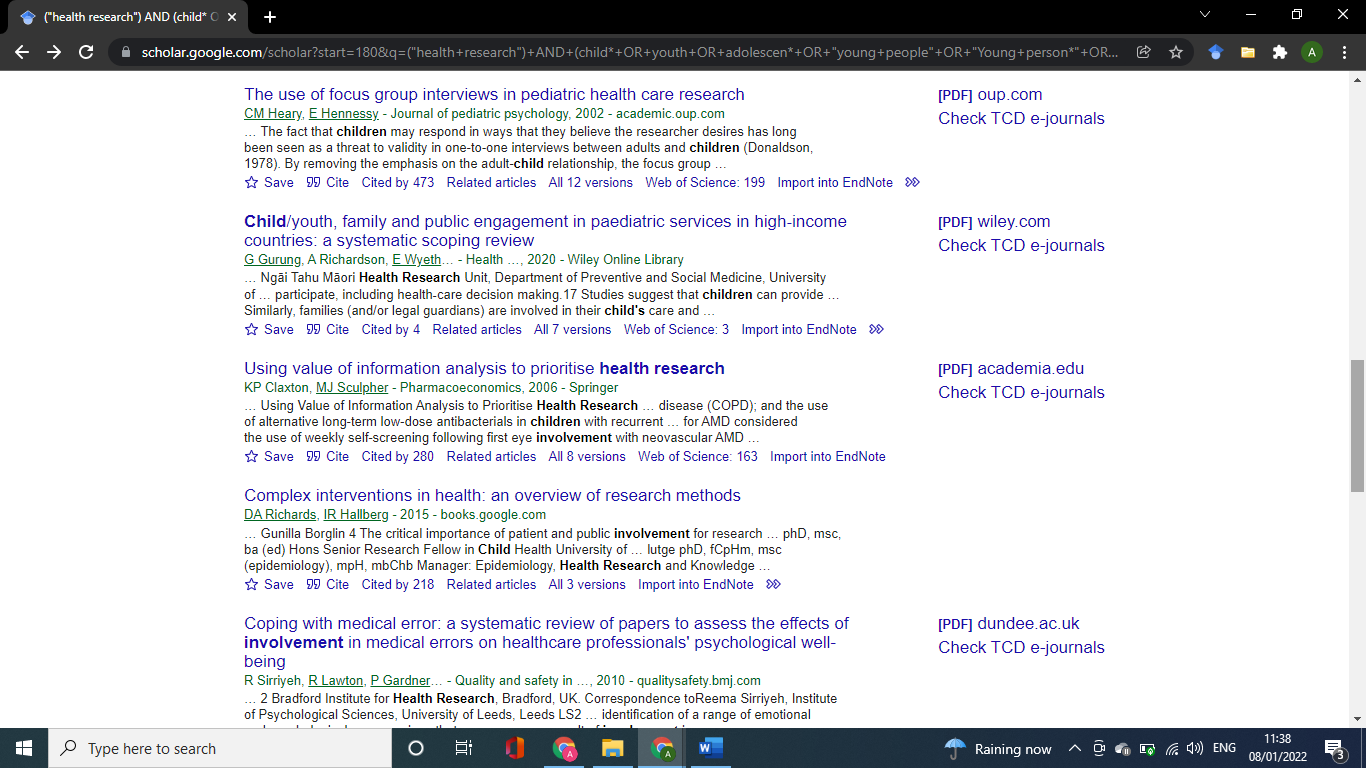 | | | | | | | | | | | | | | |
| 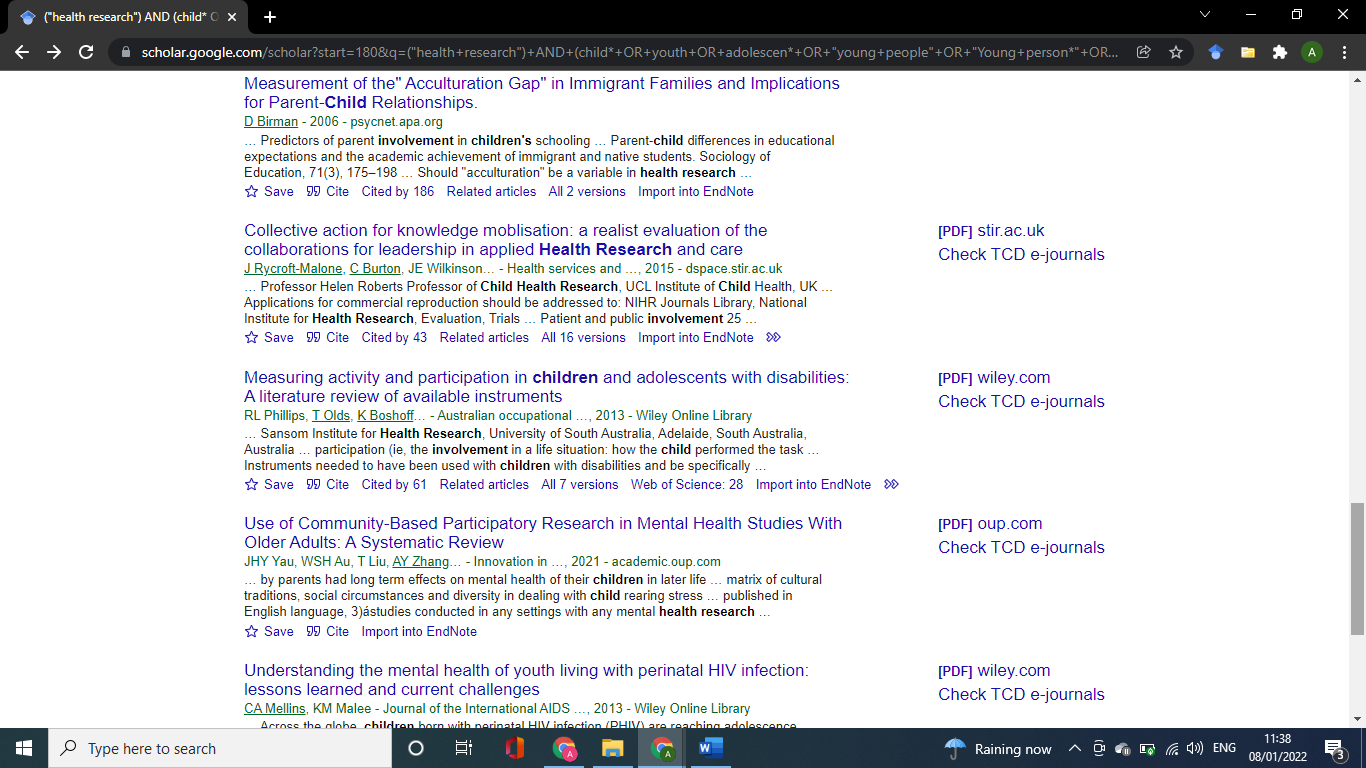 | | | | | | | | | | | | | | |
| 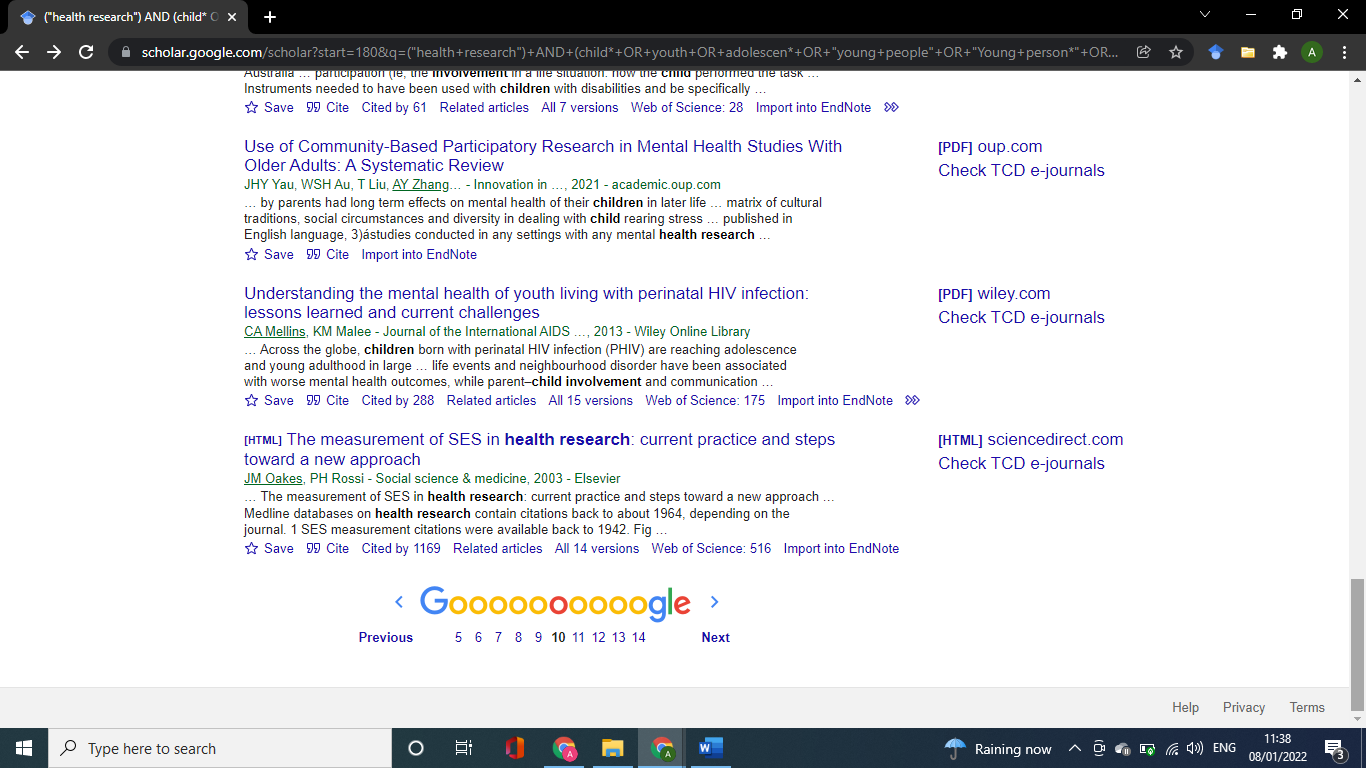 | | | | | | | | | | | | | | |
| 2 | | | | | | ("health research") AND (child* OR youth OR adolescen* OR "young people" OR "Young person*" OR "Young adult*" OR teen* OR juven*) AND ("advisory committee*" OR "advisory board*" OR "youth engagement" OR "patient and public involvement") | | | 7900 | | | | | |
| 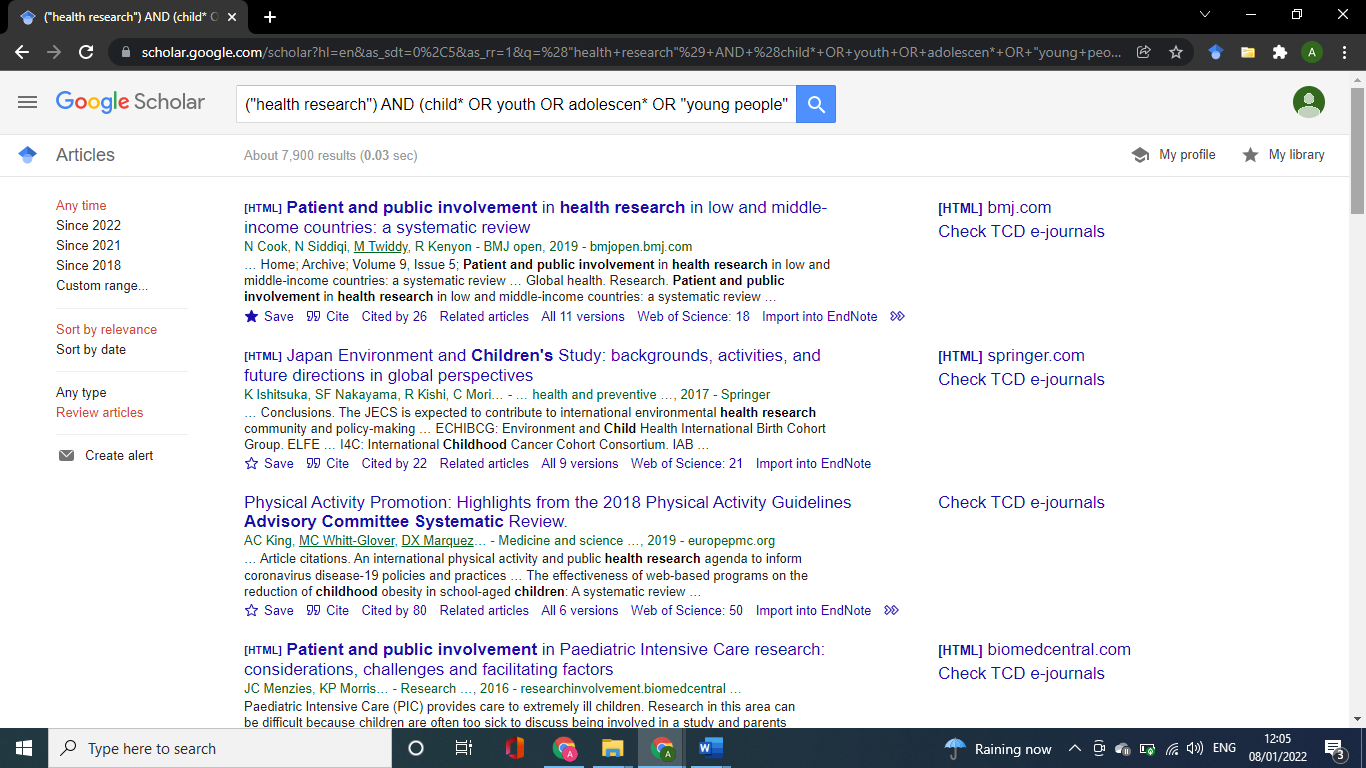 | | | | | | | | | | | | | | |
| 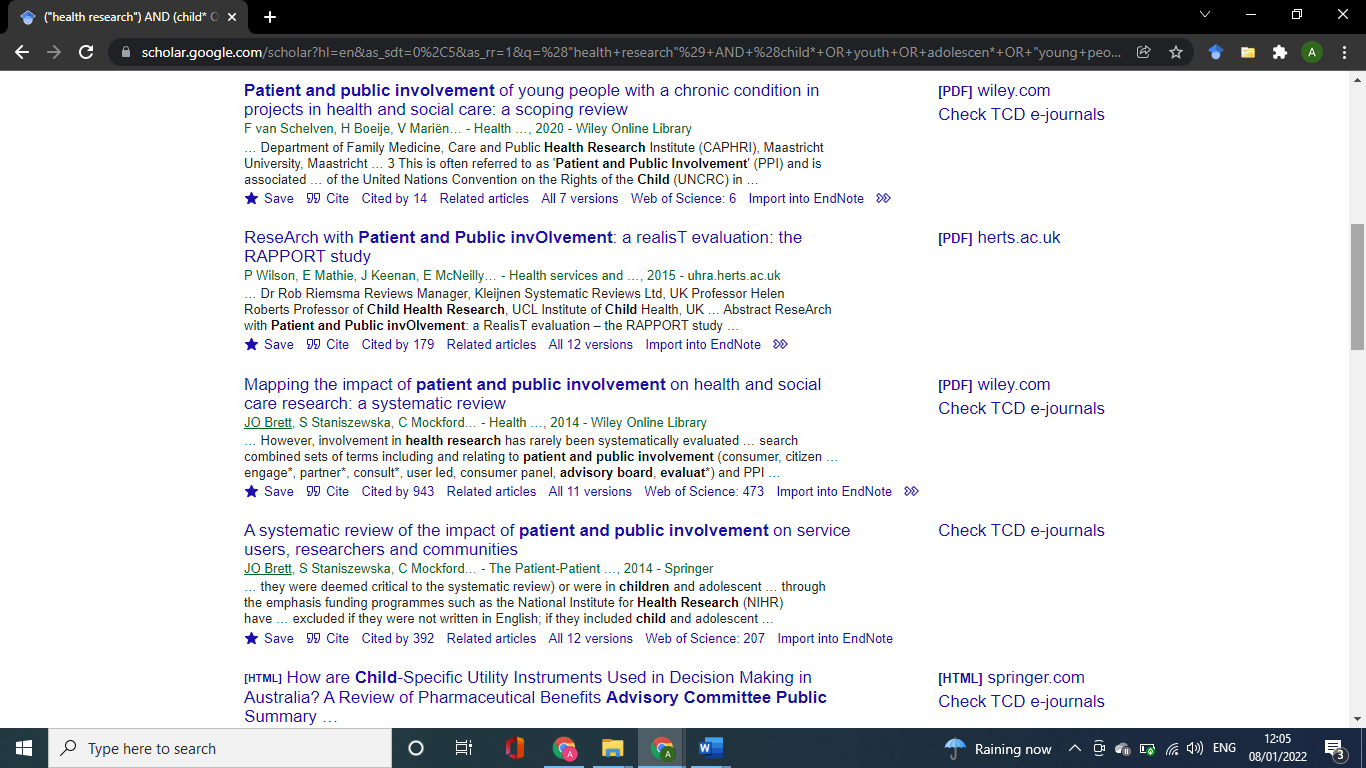 | | | | | | | | | | | | | | |
| 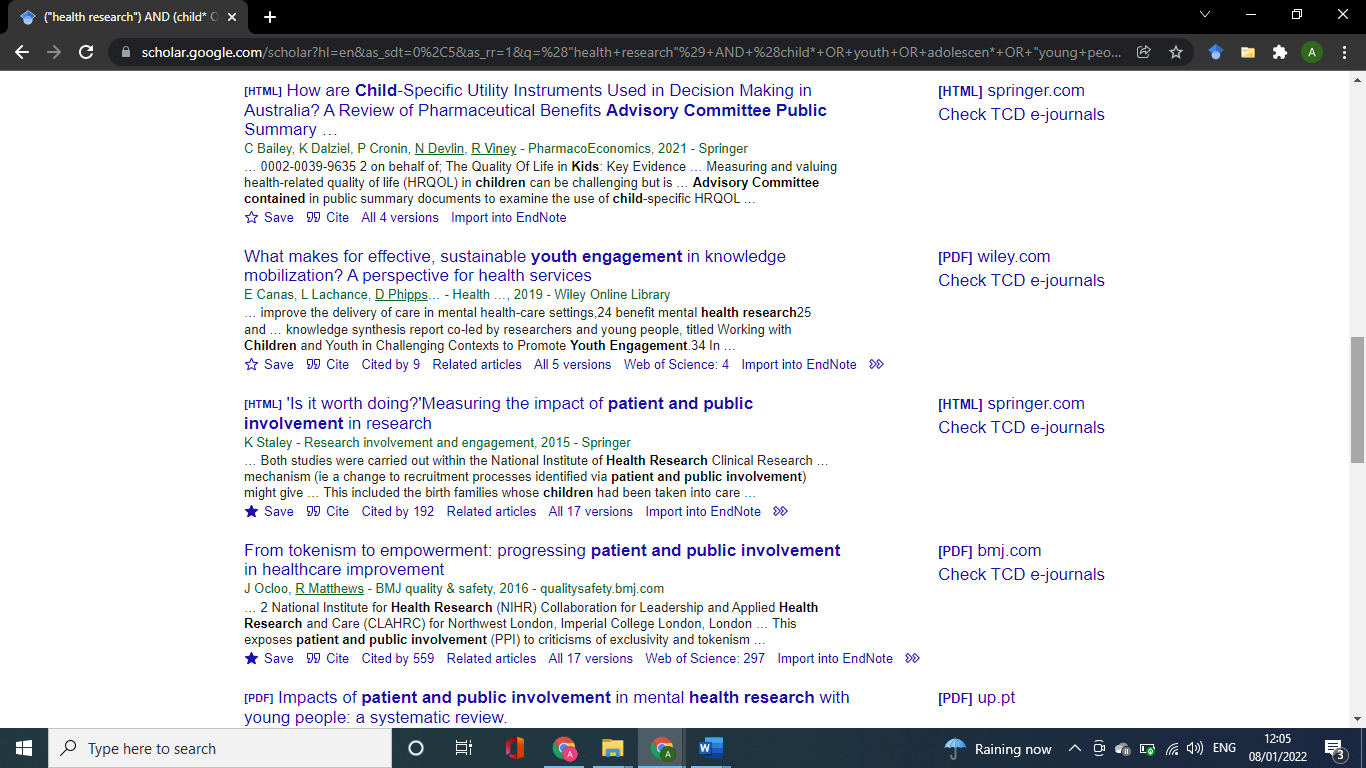 | | | | | | | | | | | | | | |
| 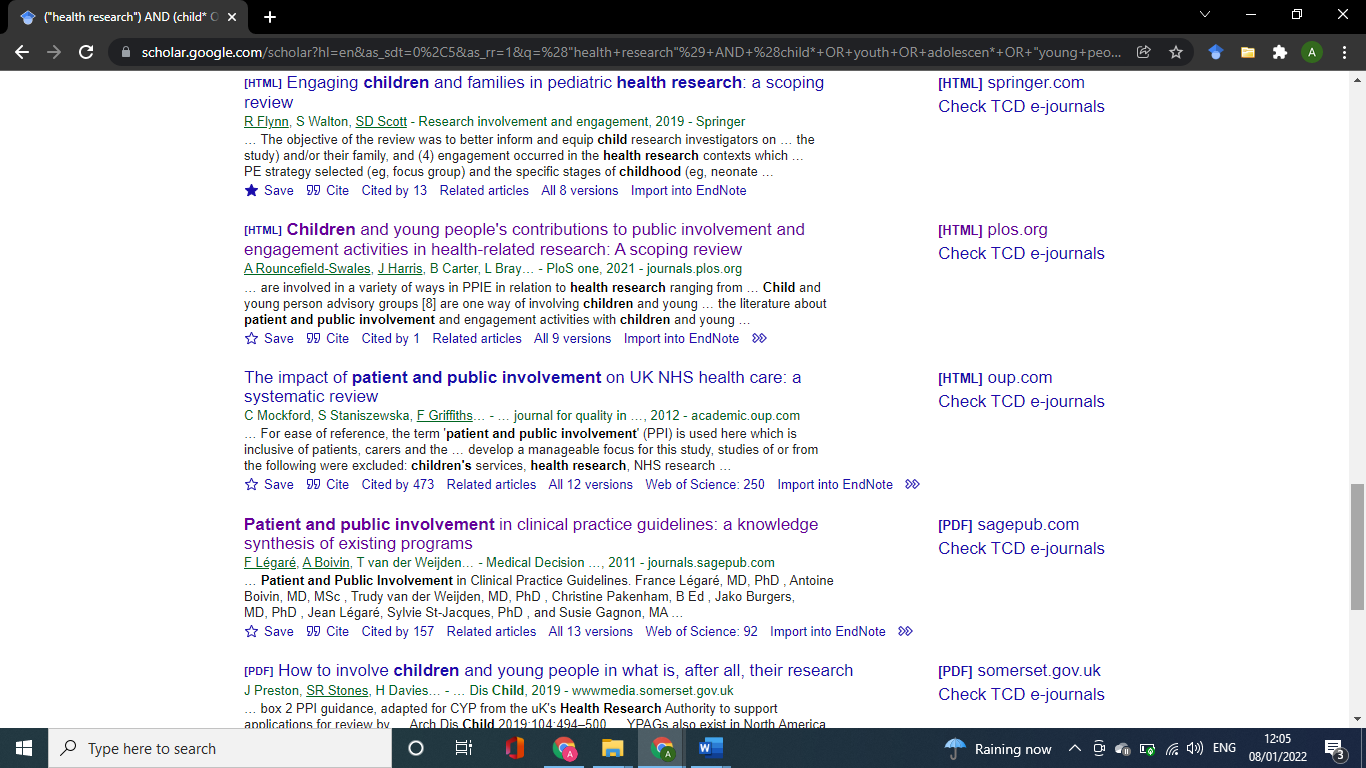 | | | | | | | | | | | | | | |
| 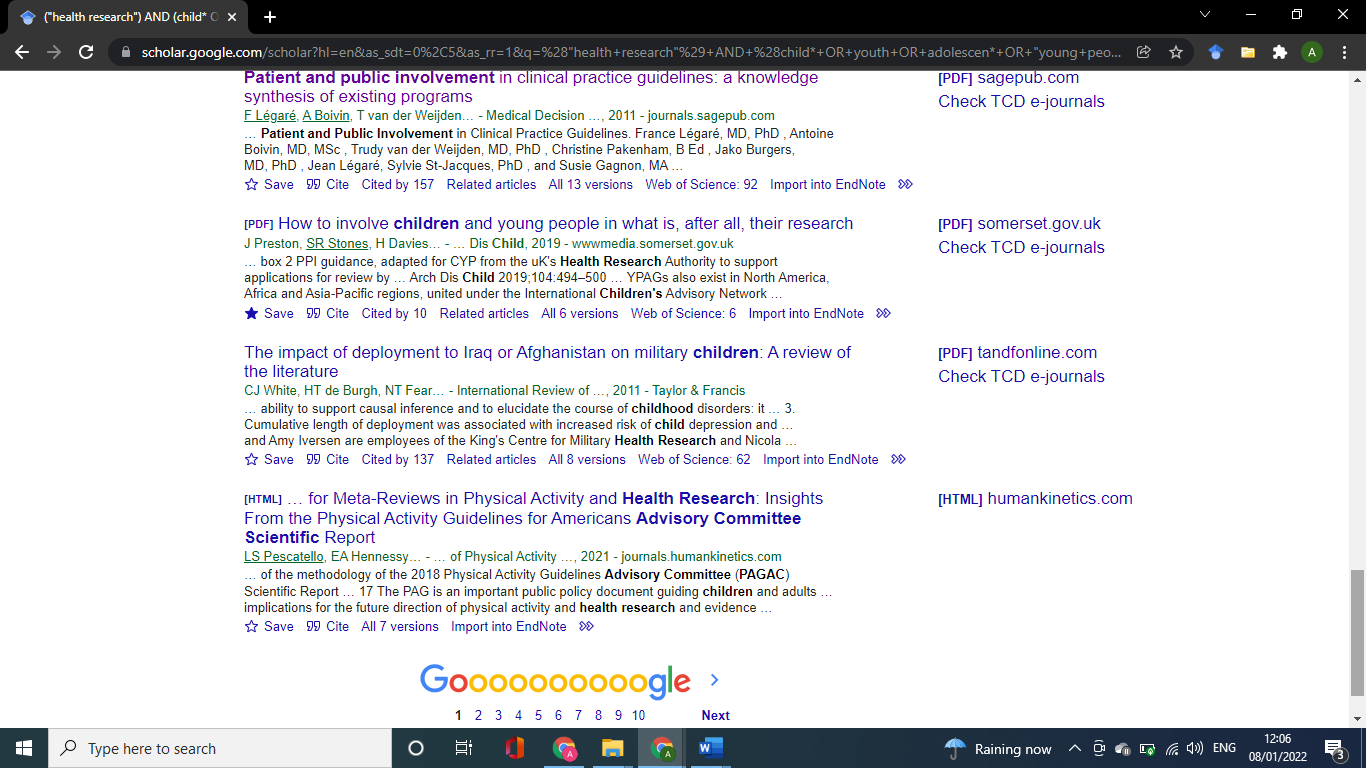 | | | | | | | | | | | | | | |
| Pg2 | | | | | | | | | | | | | | |
| 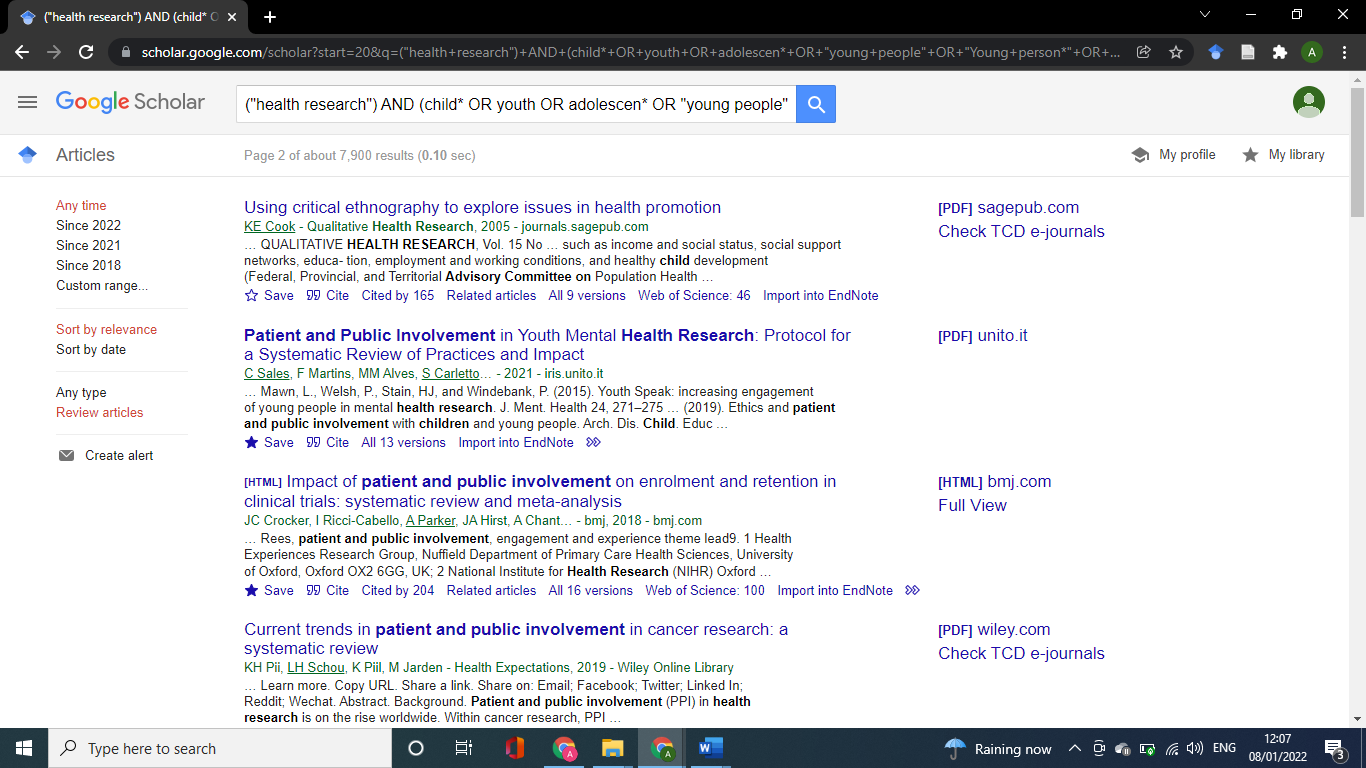 | | | | | | | | | | | | | | |
| 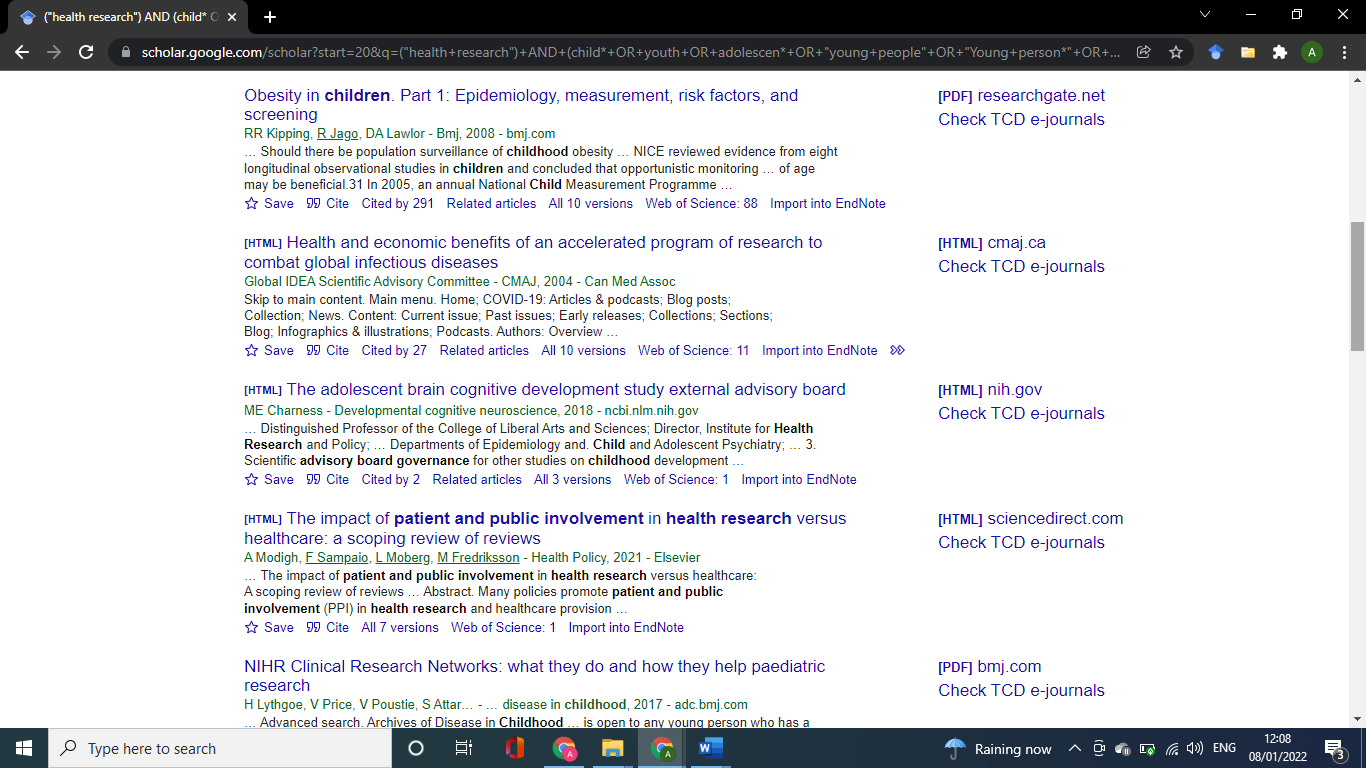 | | | | | | | | | | | | | | |
| 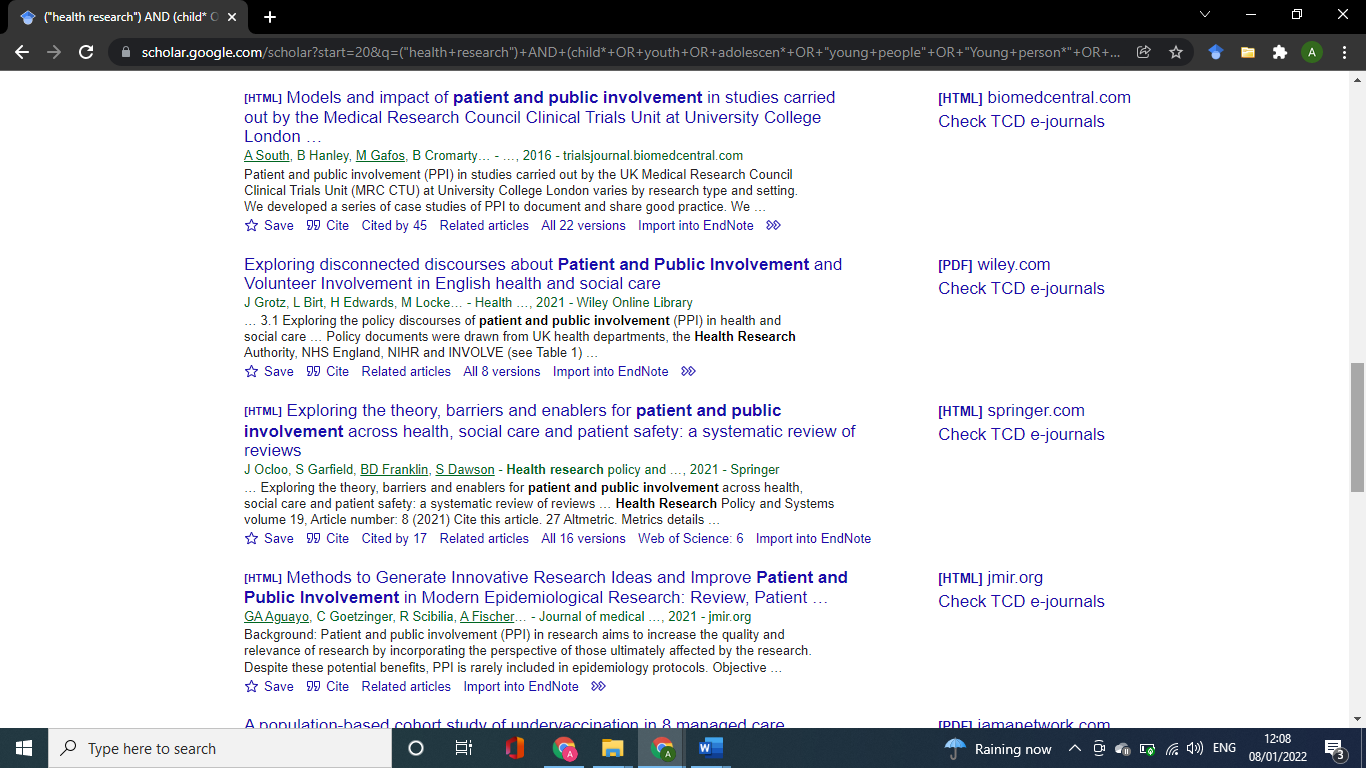 | | | | | | | | | | | | | | |
| 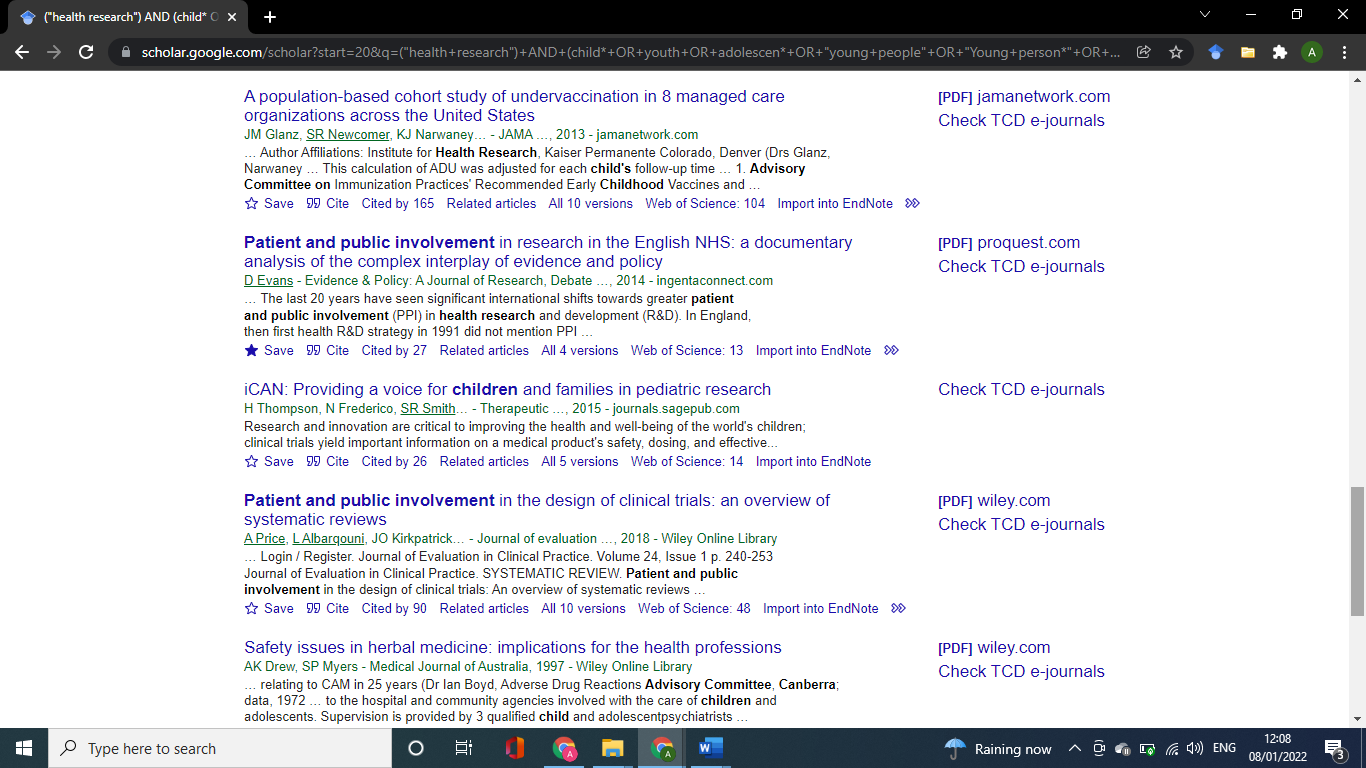 | | | | | | | | | | | | | | |
| 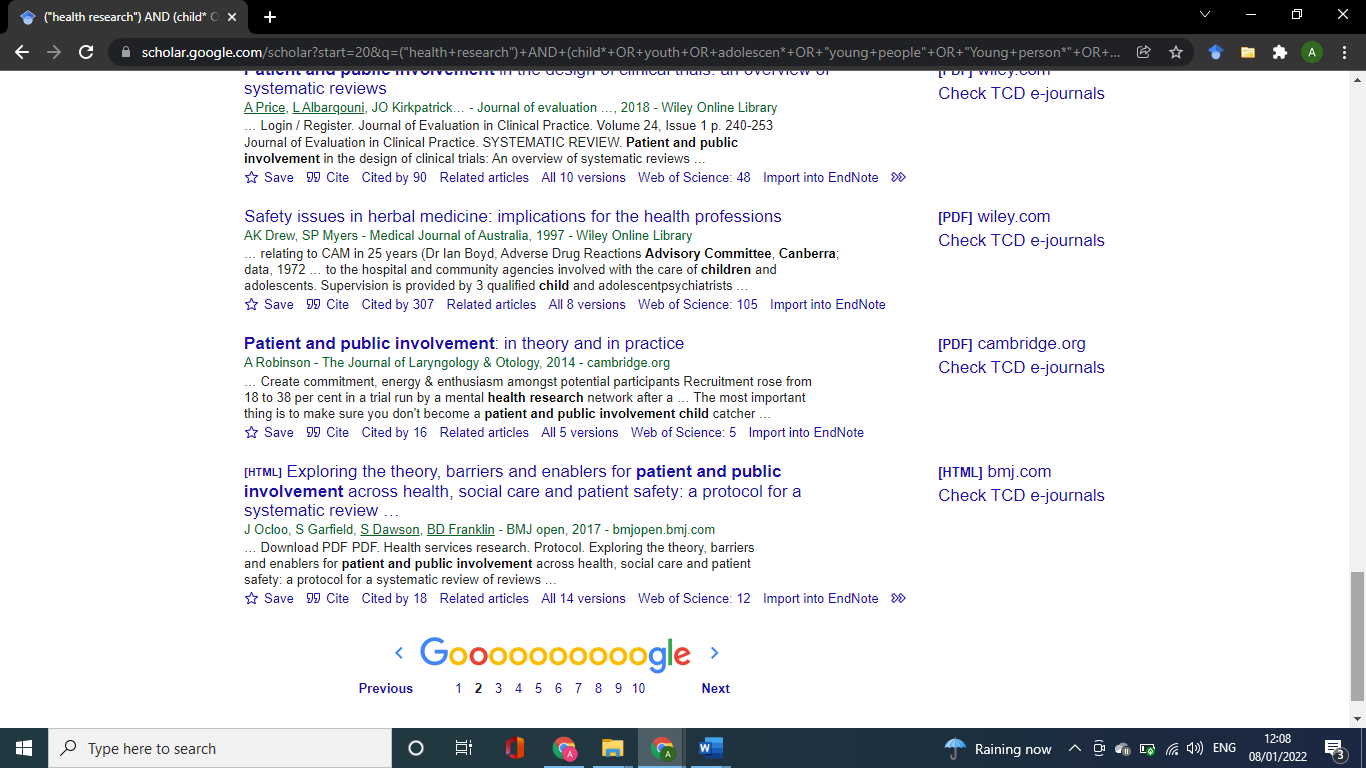 | | | | | | | | | | | | | | |
| Pg3 | | | | | | | | | | | | | | |
| 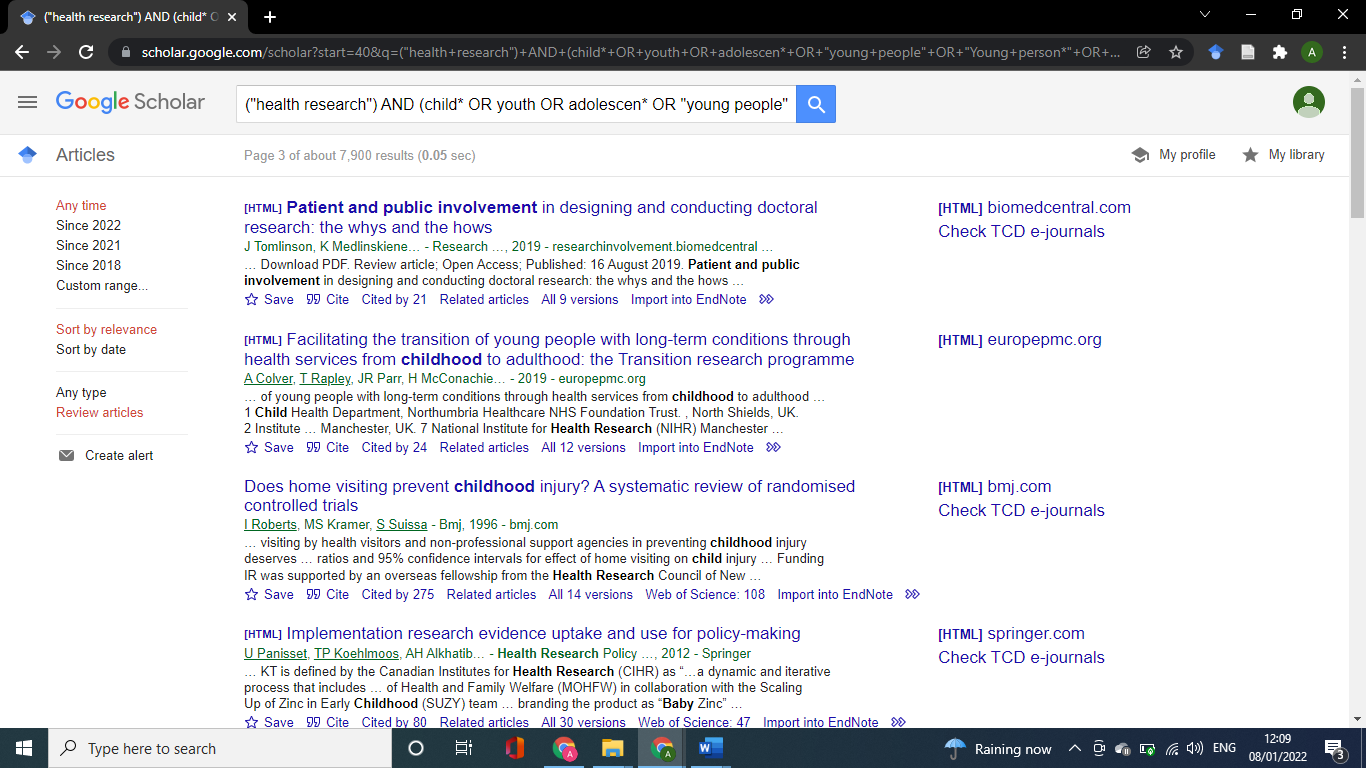 | | | | | | | | | | | | | | |
| 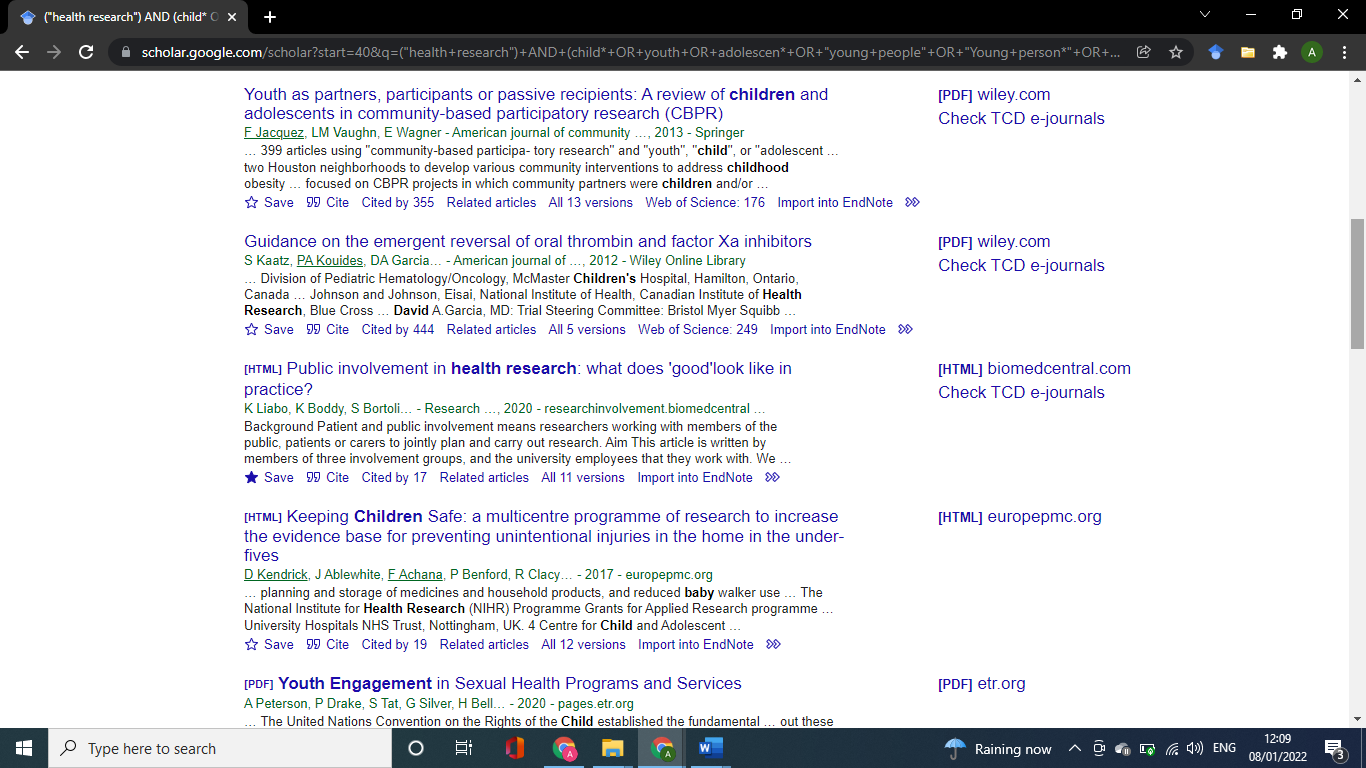 | | | | | | | | | | | | | | |
| 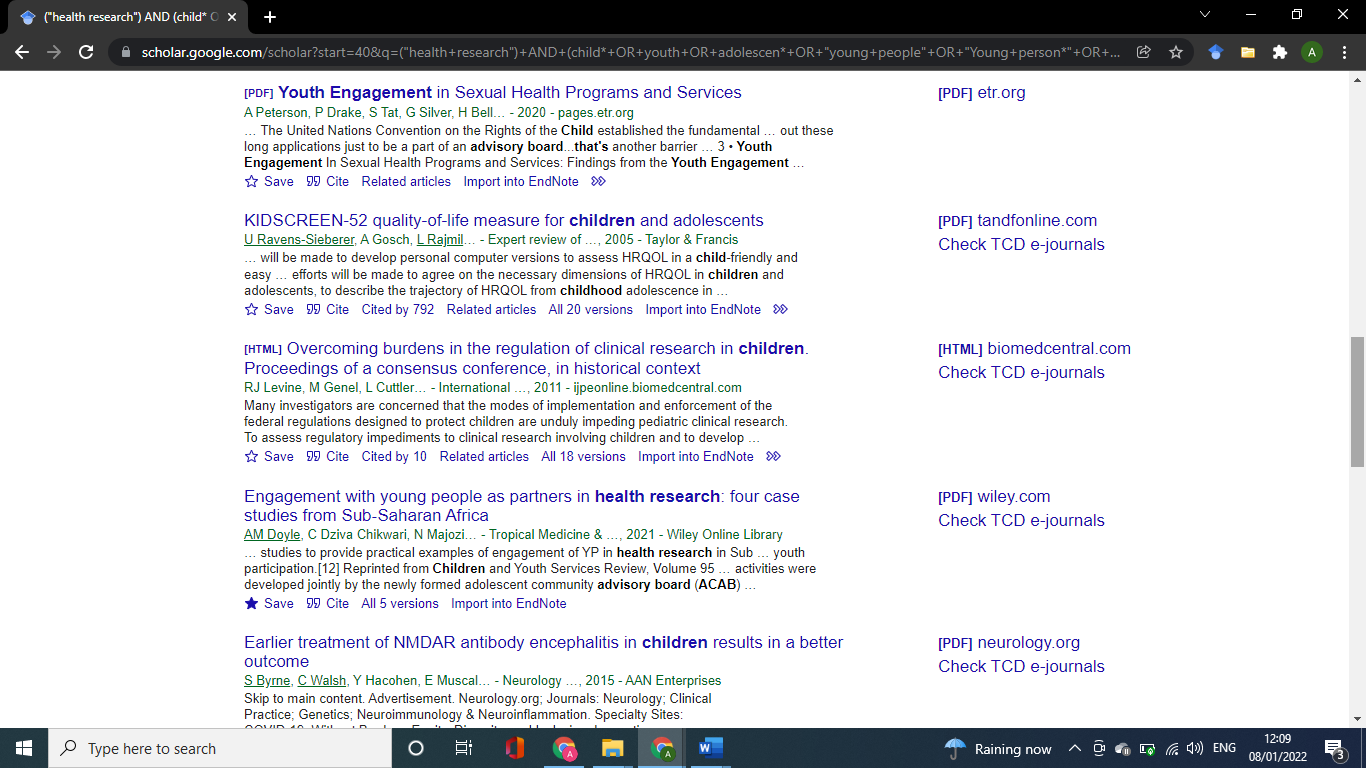 | | | | | | | | | | | | | | |
| 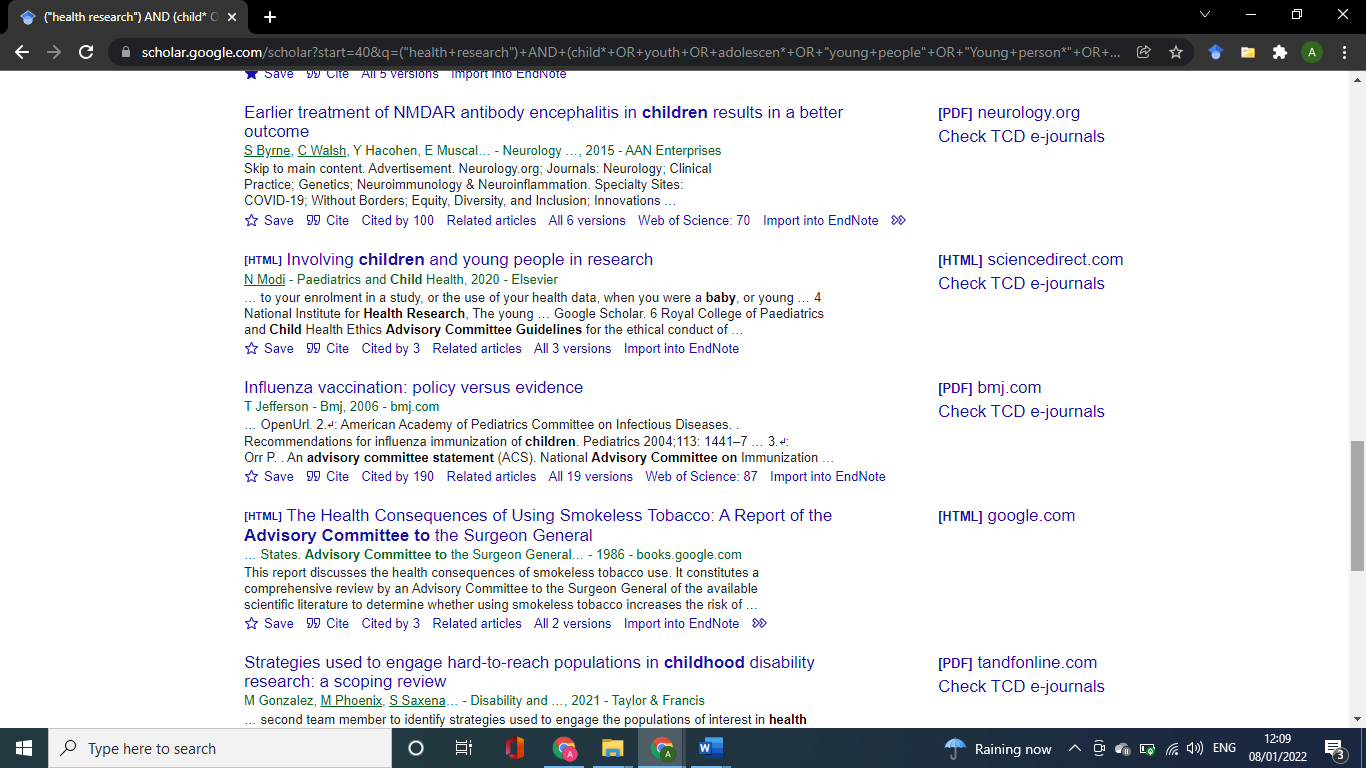 | | | | | | | | | | | | | | |
| 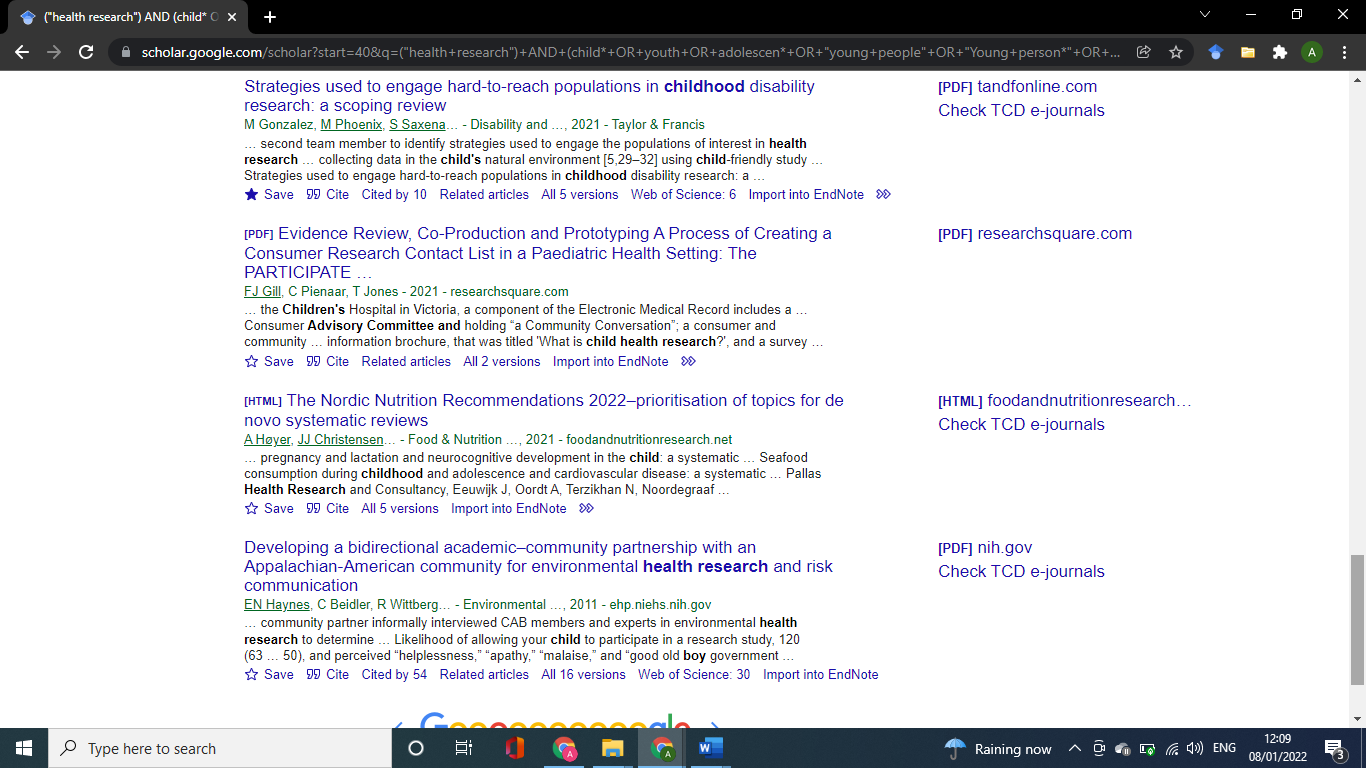 | | | | | | | | | | | | | | |
| 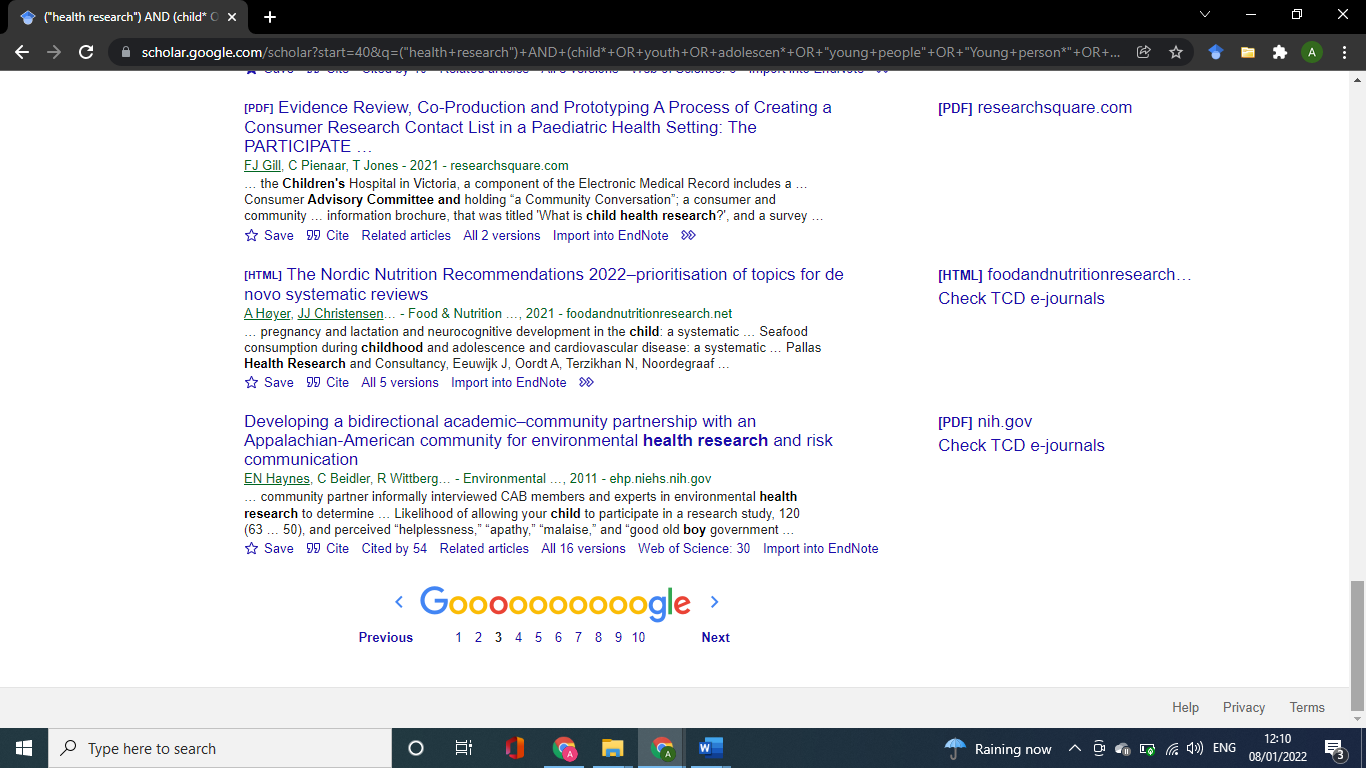 | | | | | | | | | | | | | | |
| Pg 4 | | | | | | | | | | | | | | |
| 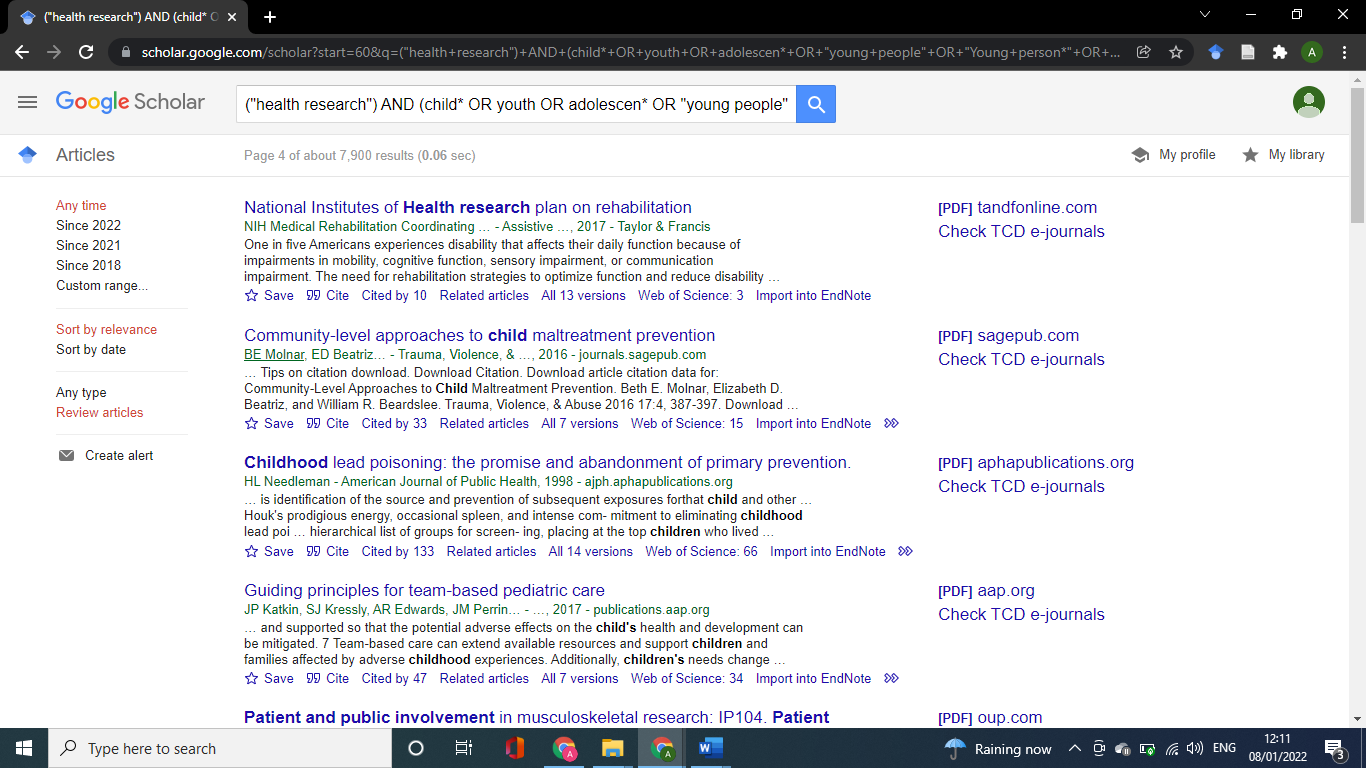 | | | | | | | | | | | | | | |
| 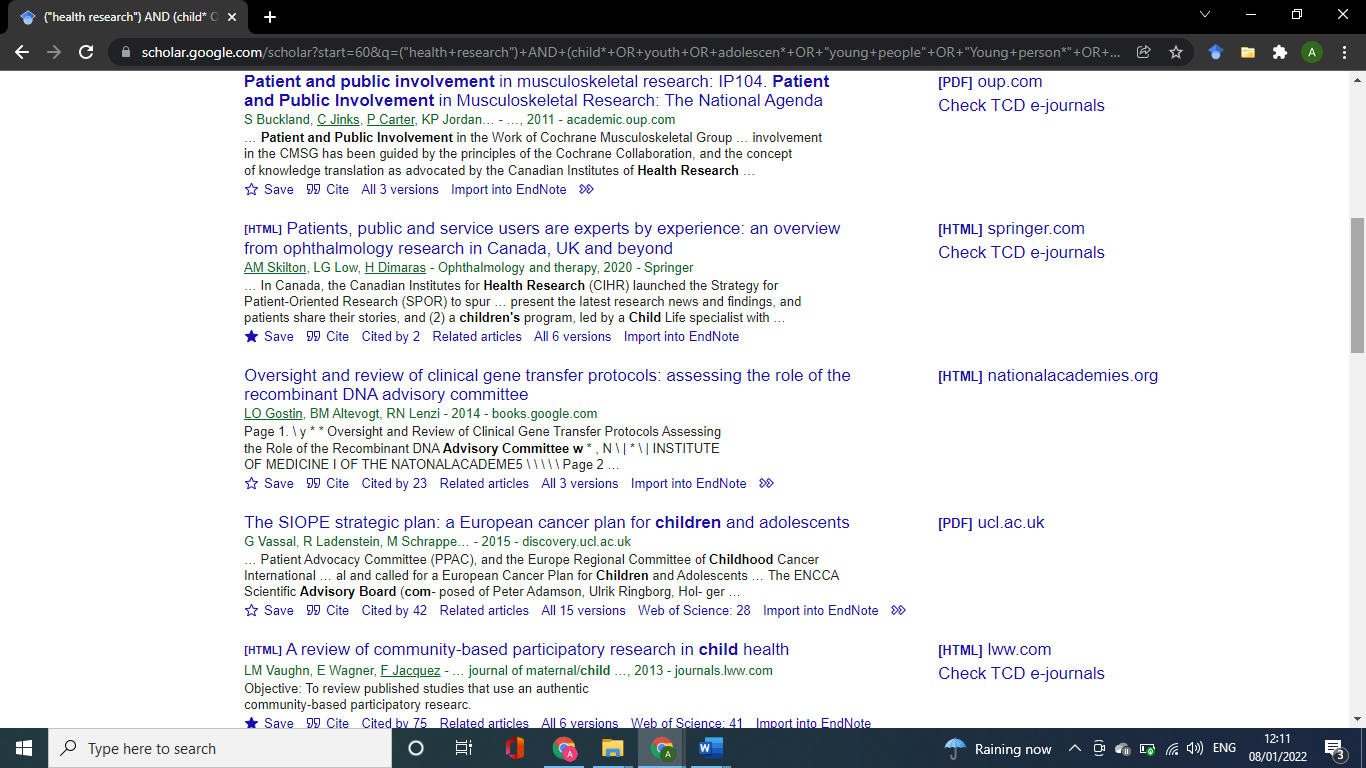 | | | | | | | | | | | | | | |
| 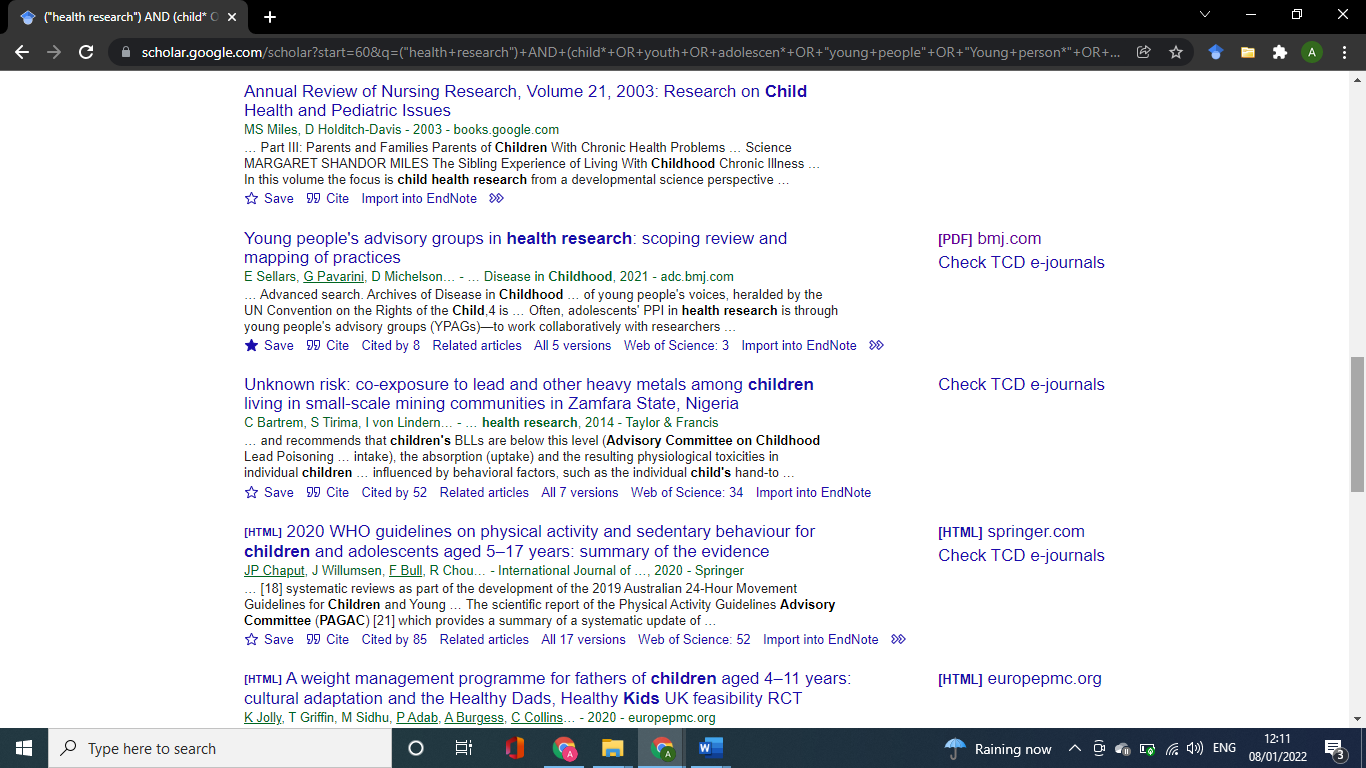 | | | | | | | | | | | | | | |
| 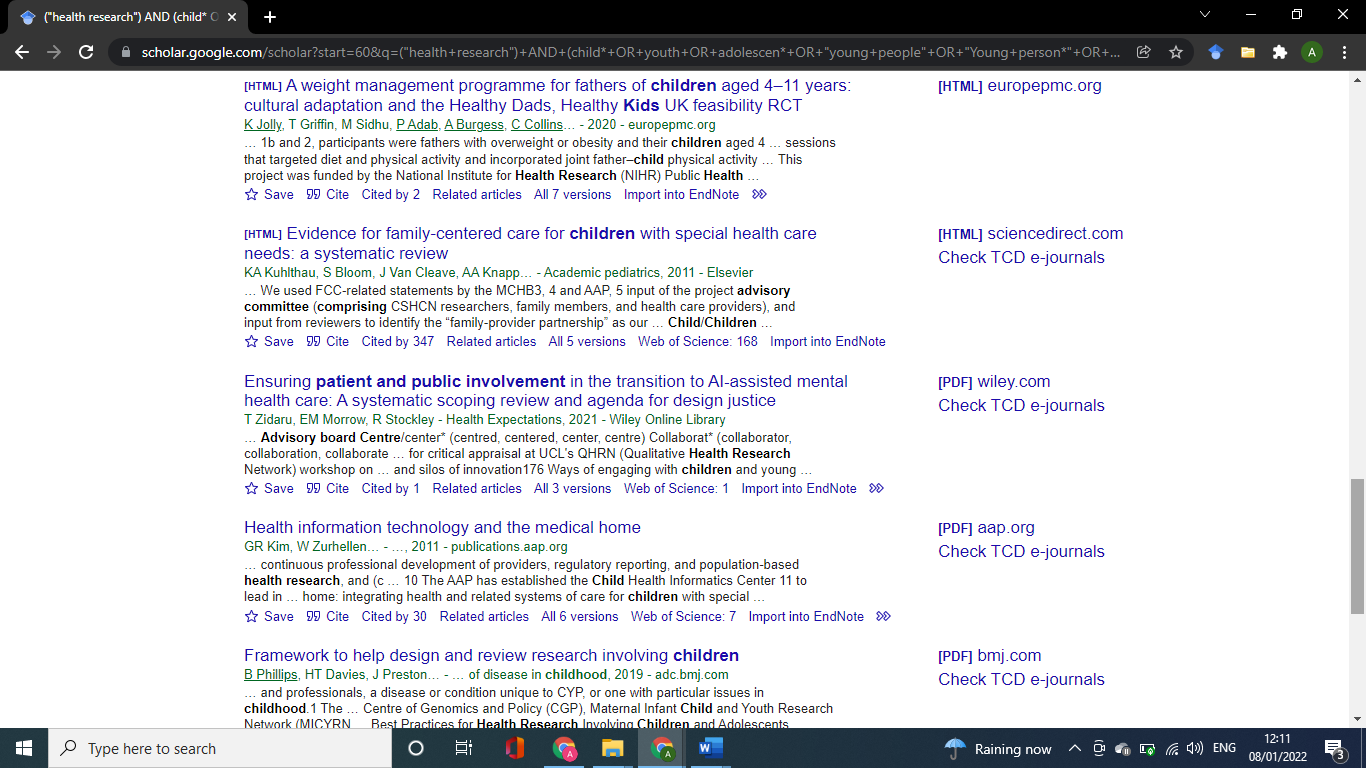 | | | | | | | | | | | | | | |
| 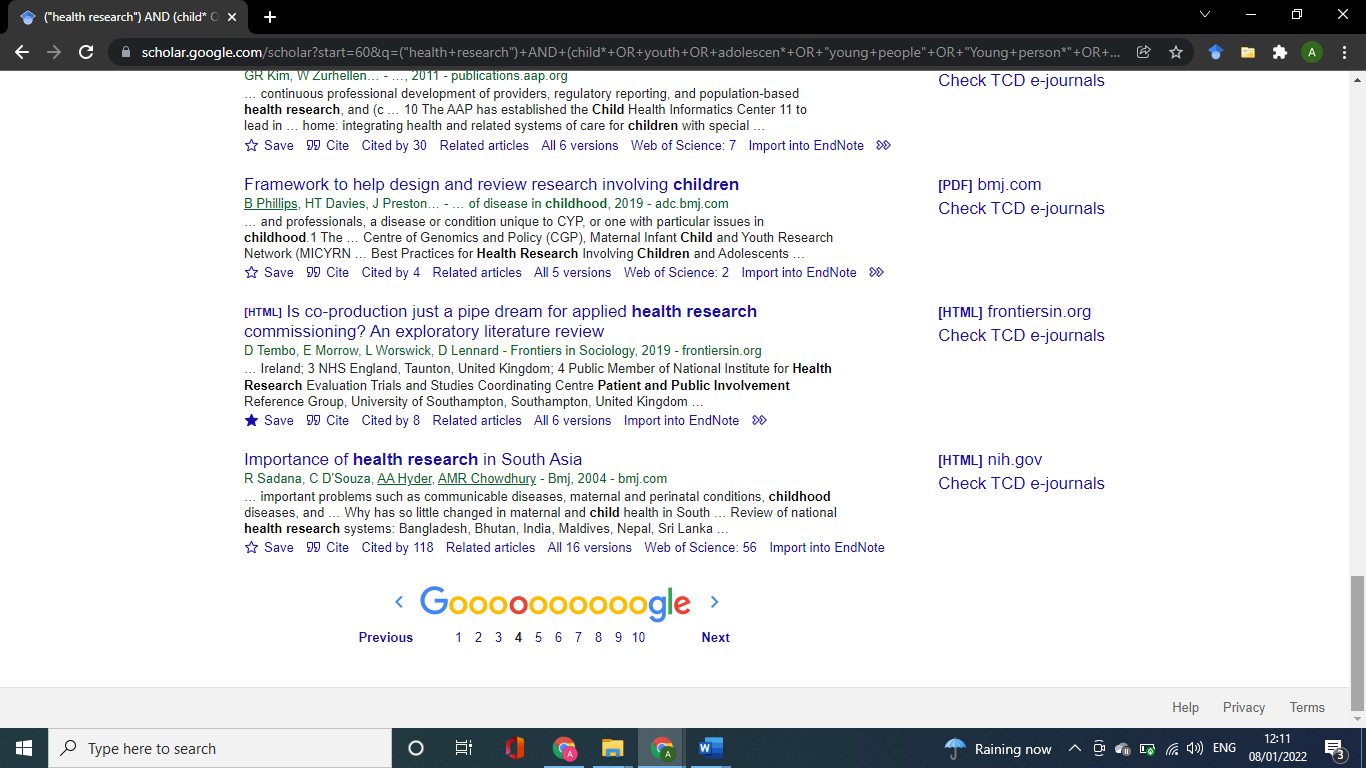 | | | | | | | | | | | | | | |
| Pg 5 | | | | | | | | | | | | | | |
| 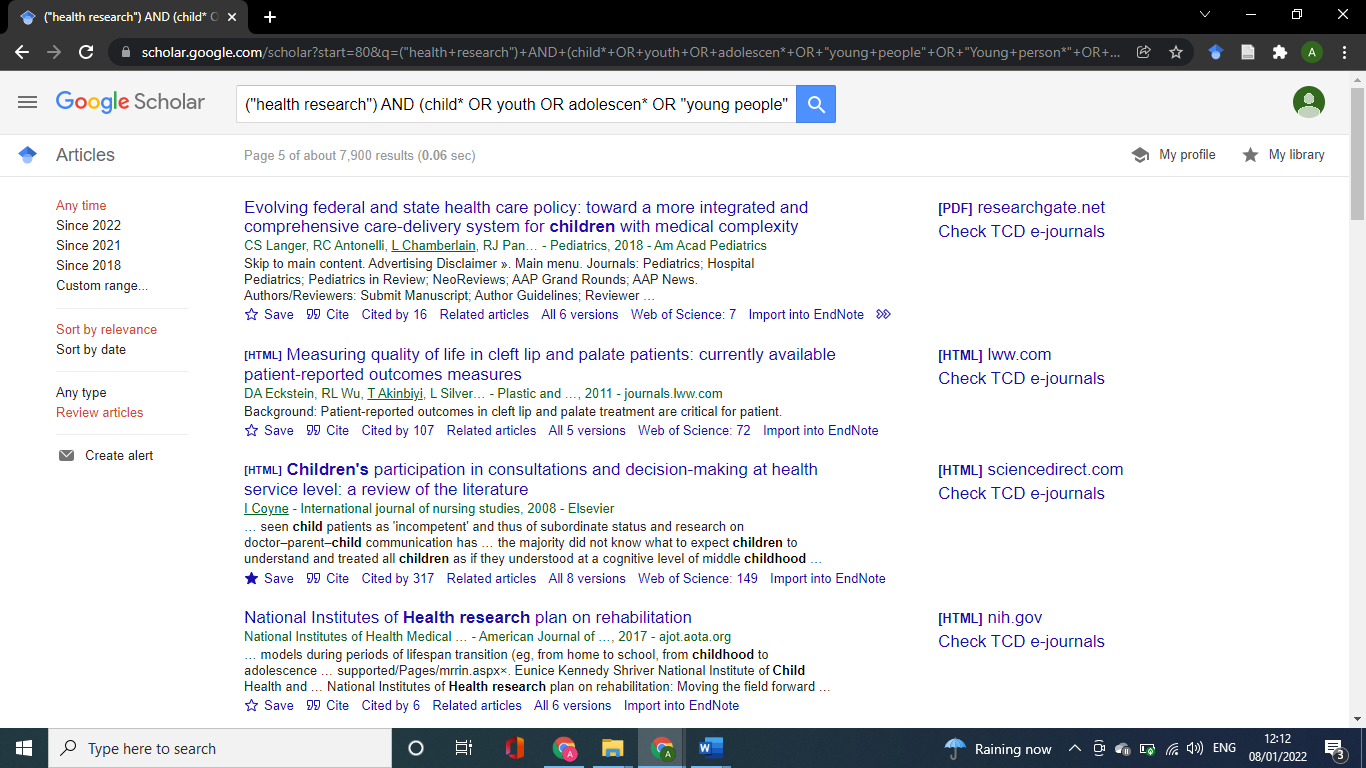 | | | | | | | | | | | | | | |
| 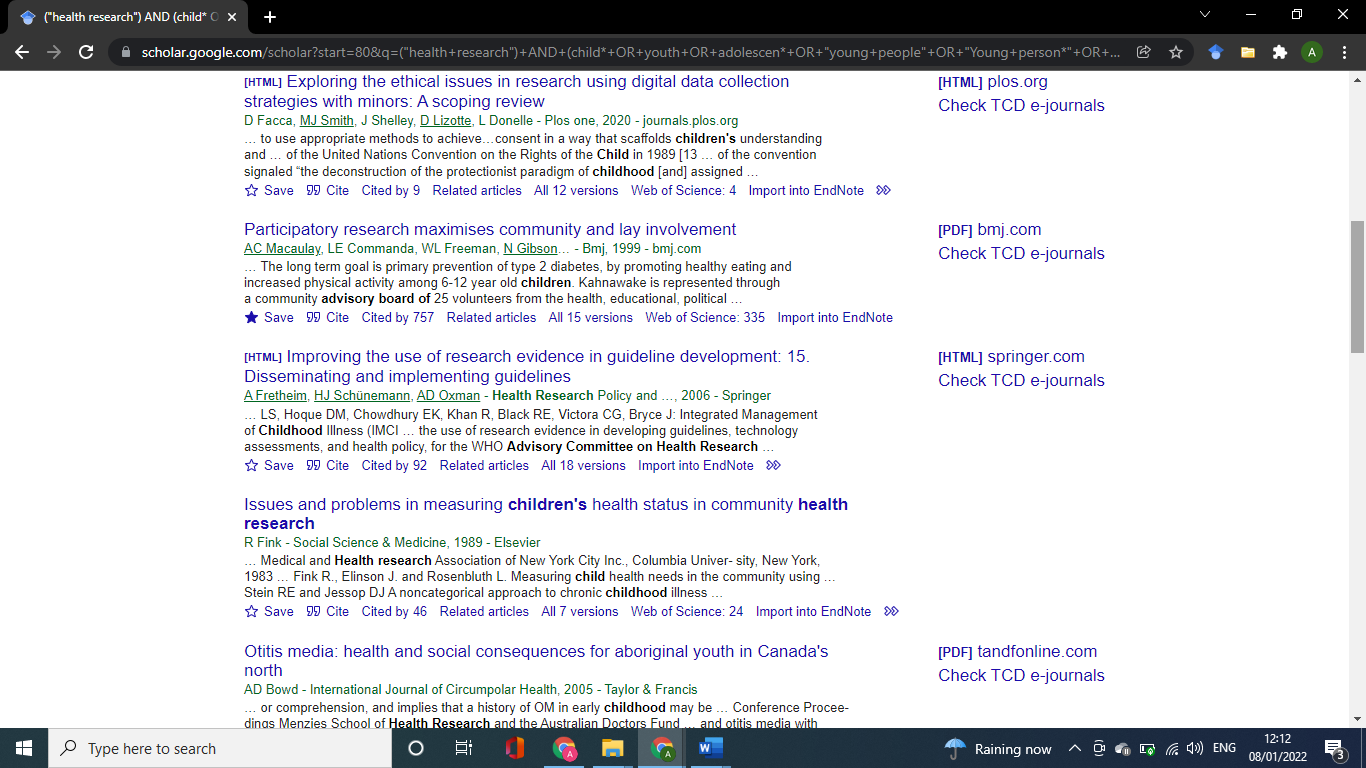 | | | | | | | | | | | | | | |
| 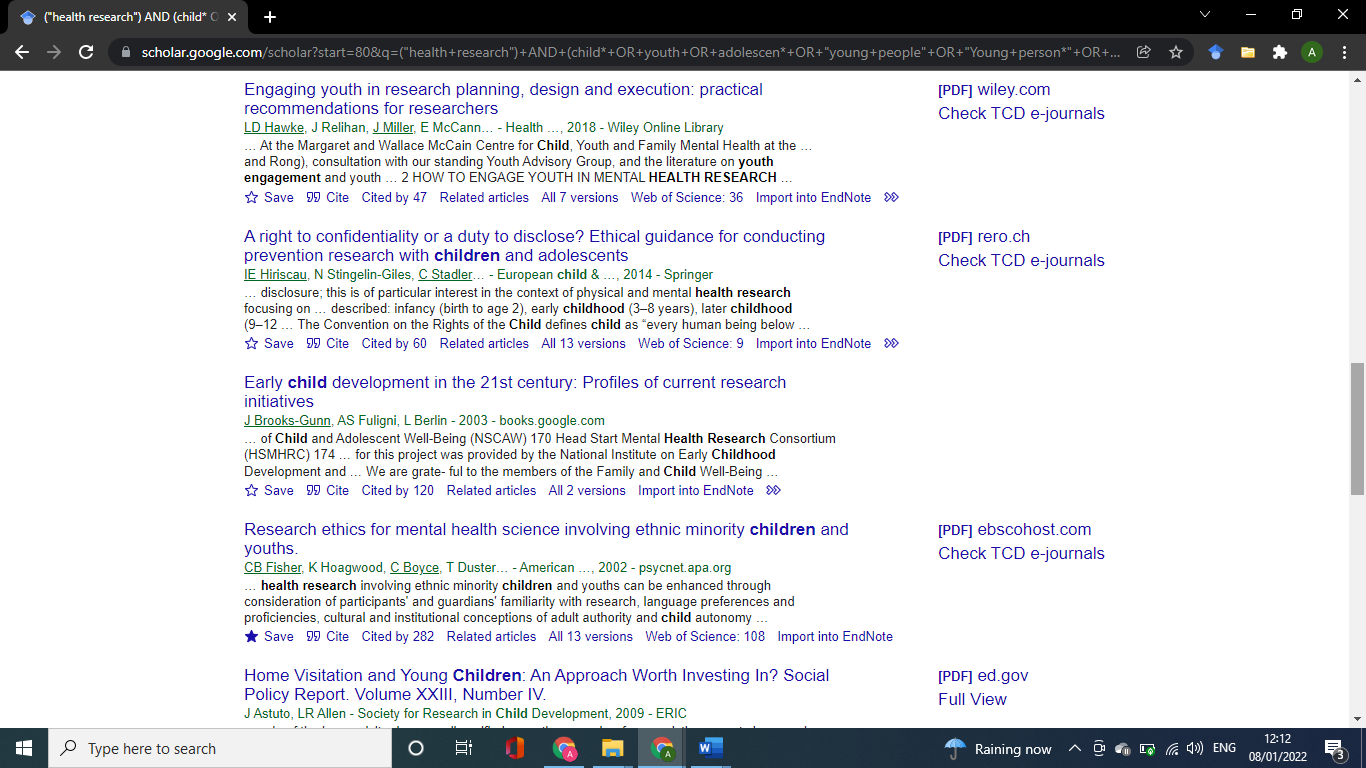 | | | | | | | | | | | | | | |
| 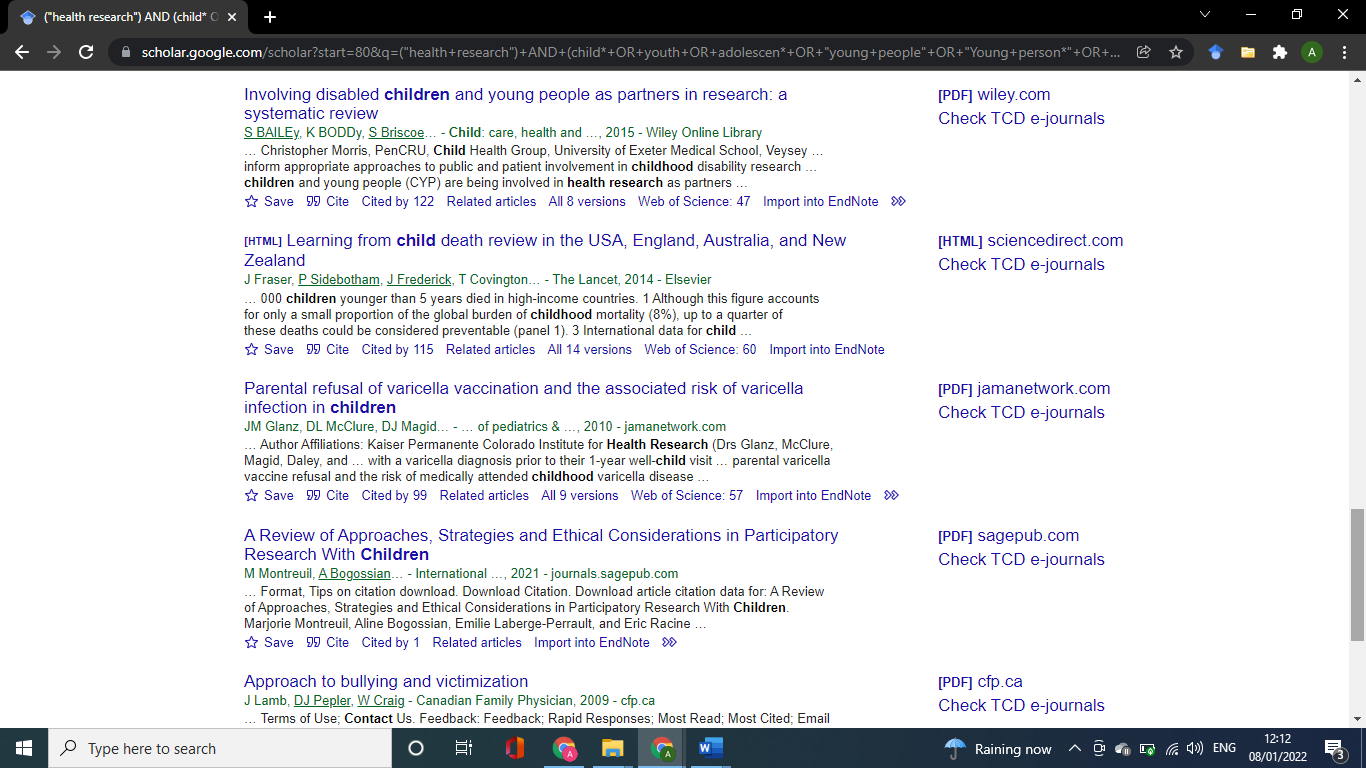 | | | | | | | | | | | | | | |
| 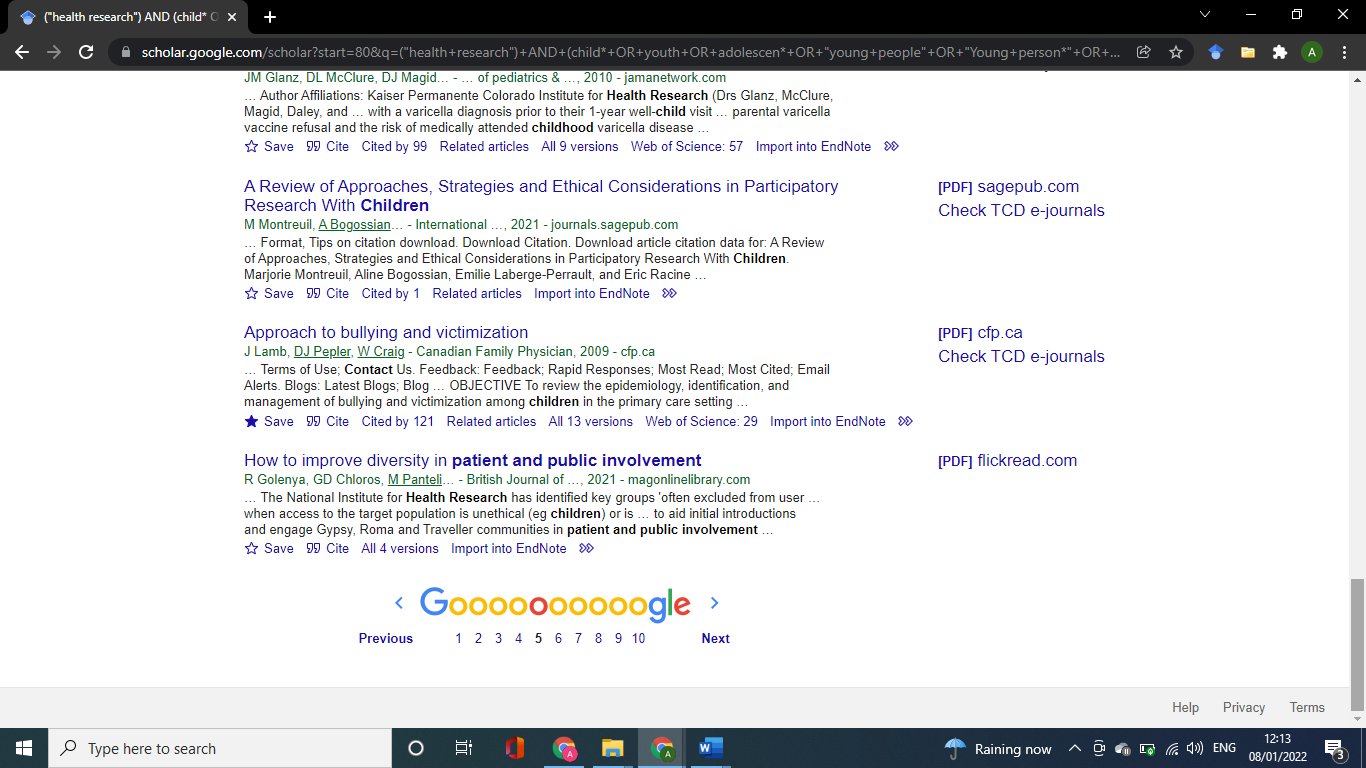 | | | | | | | | | | | | | | |
| Pg 6 | | | | | | | | | | | | | | |
| 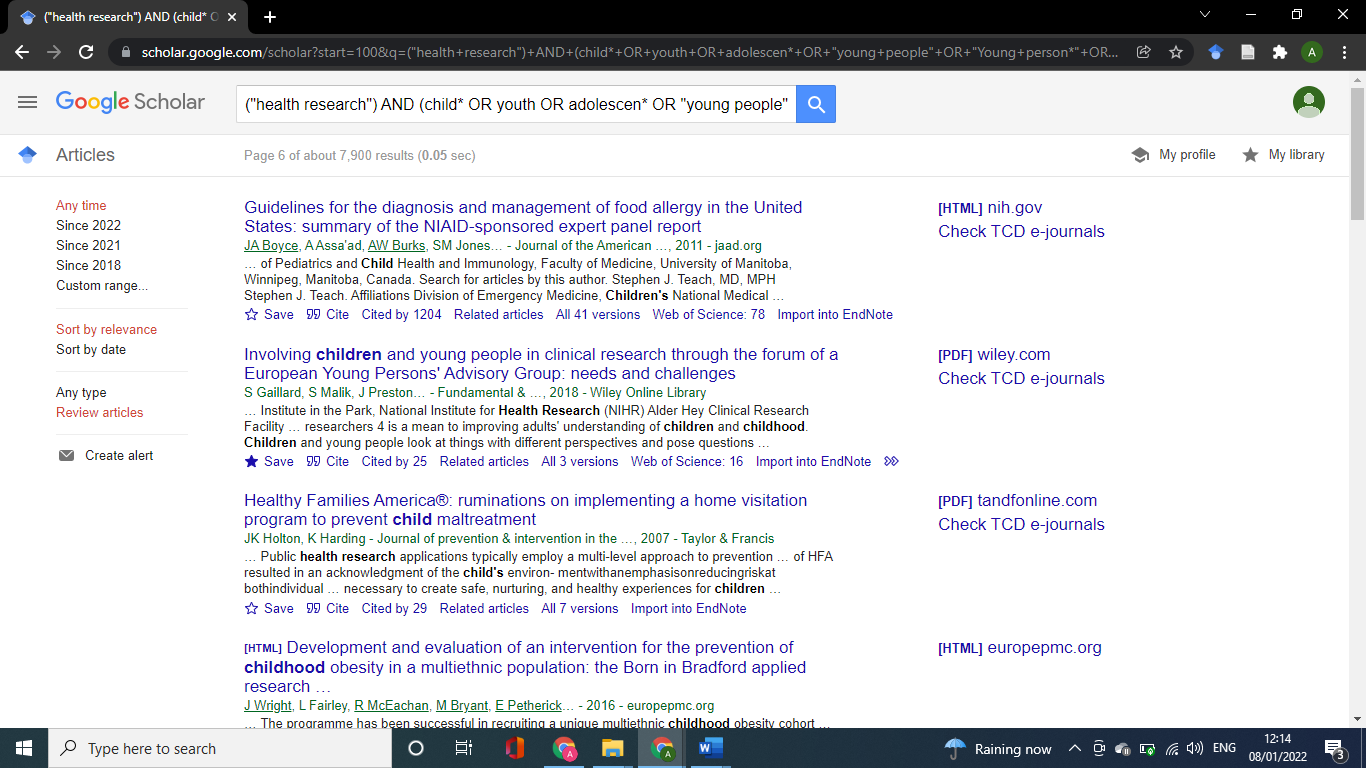 | | | | | | | | | | | | | | |
| 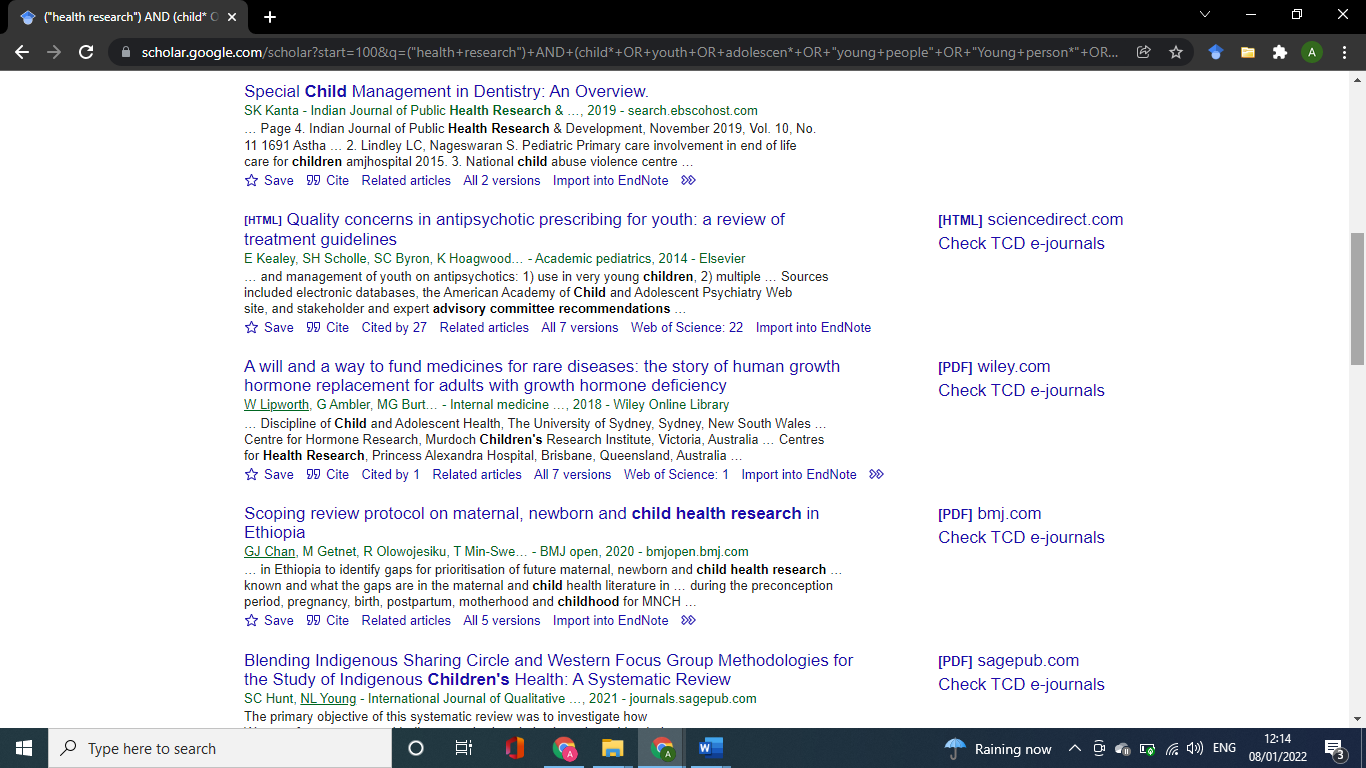 | | | | | | | | | | | | | | |
| 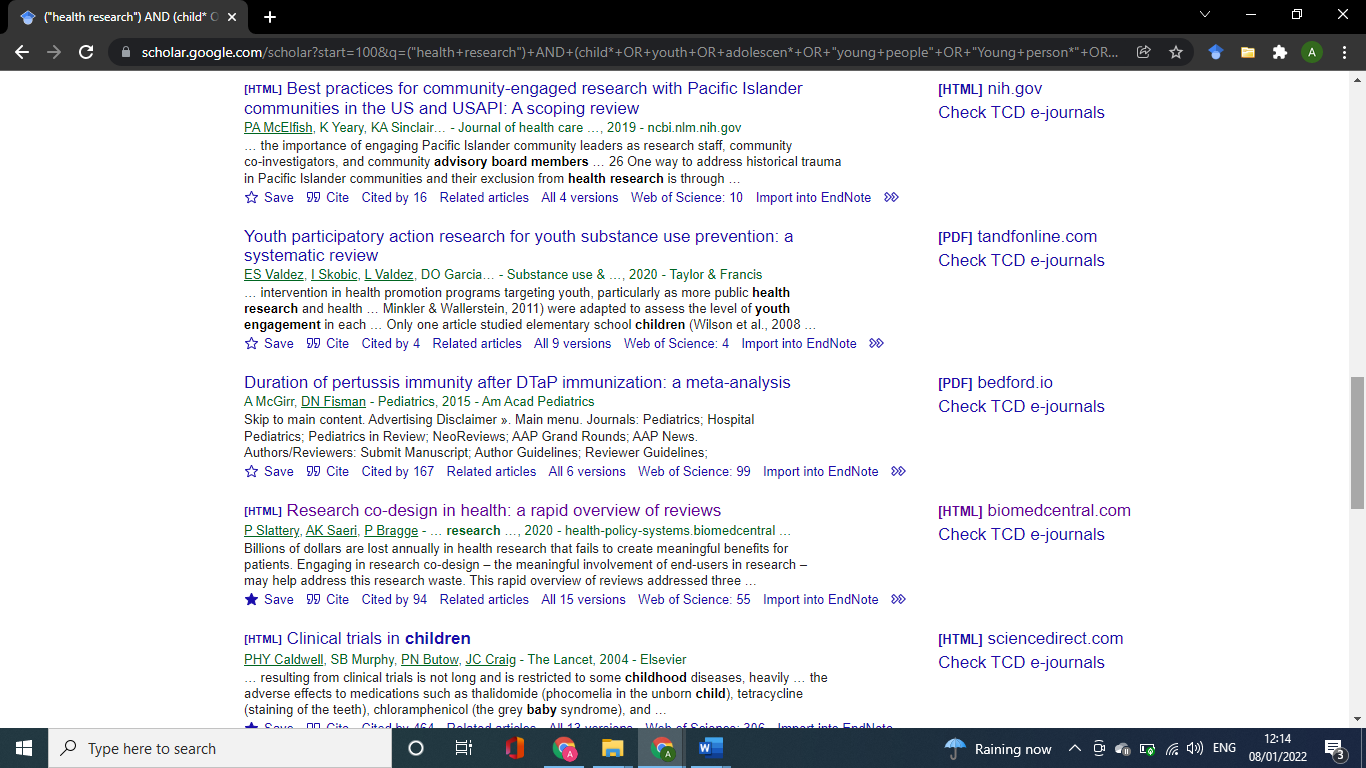 | | | | | | | | | | | | | | |
| 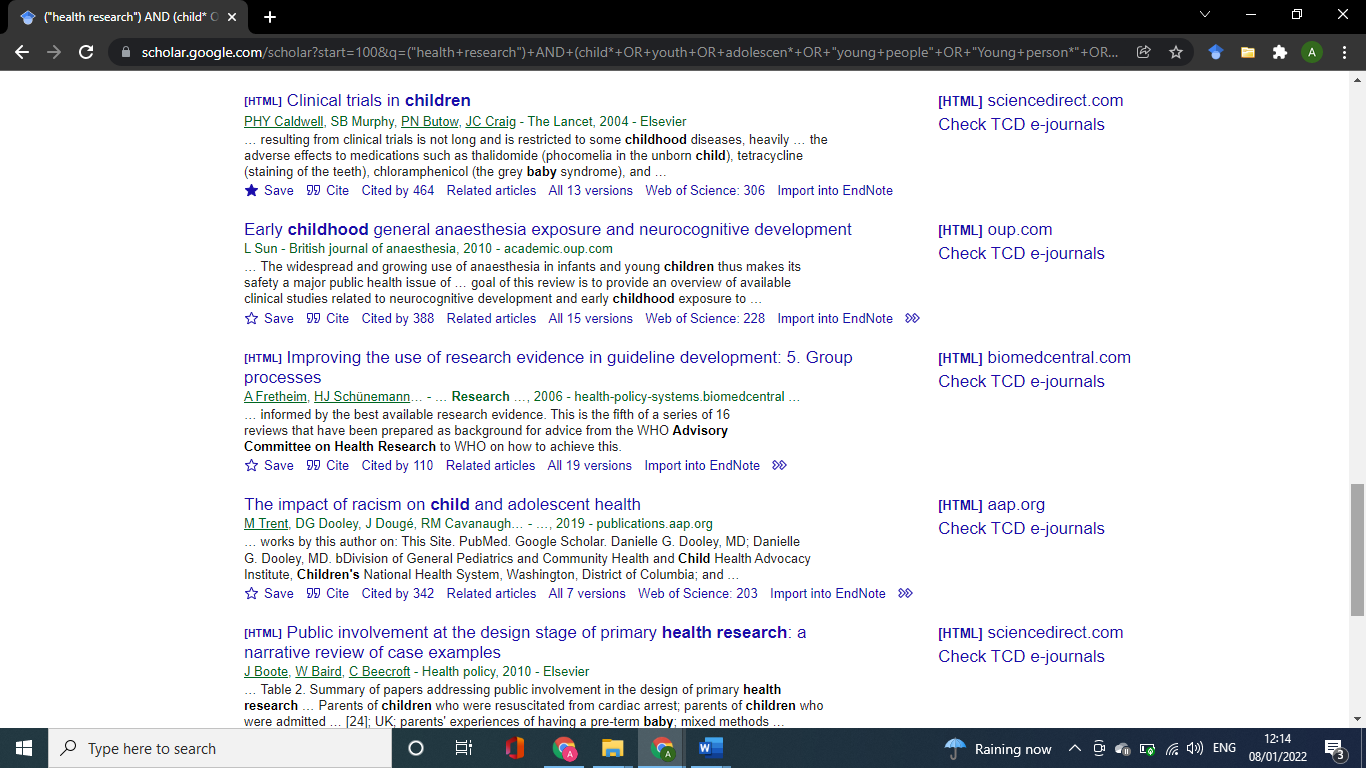 | | | | | | | | | | | | | | |
| 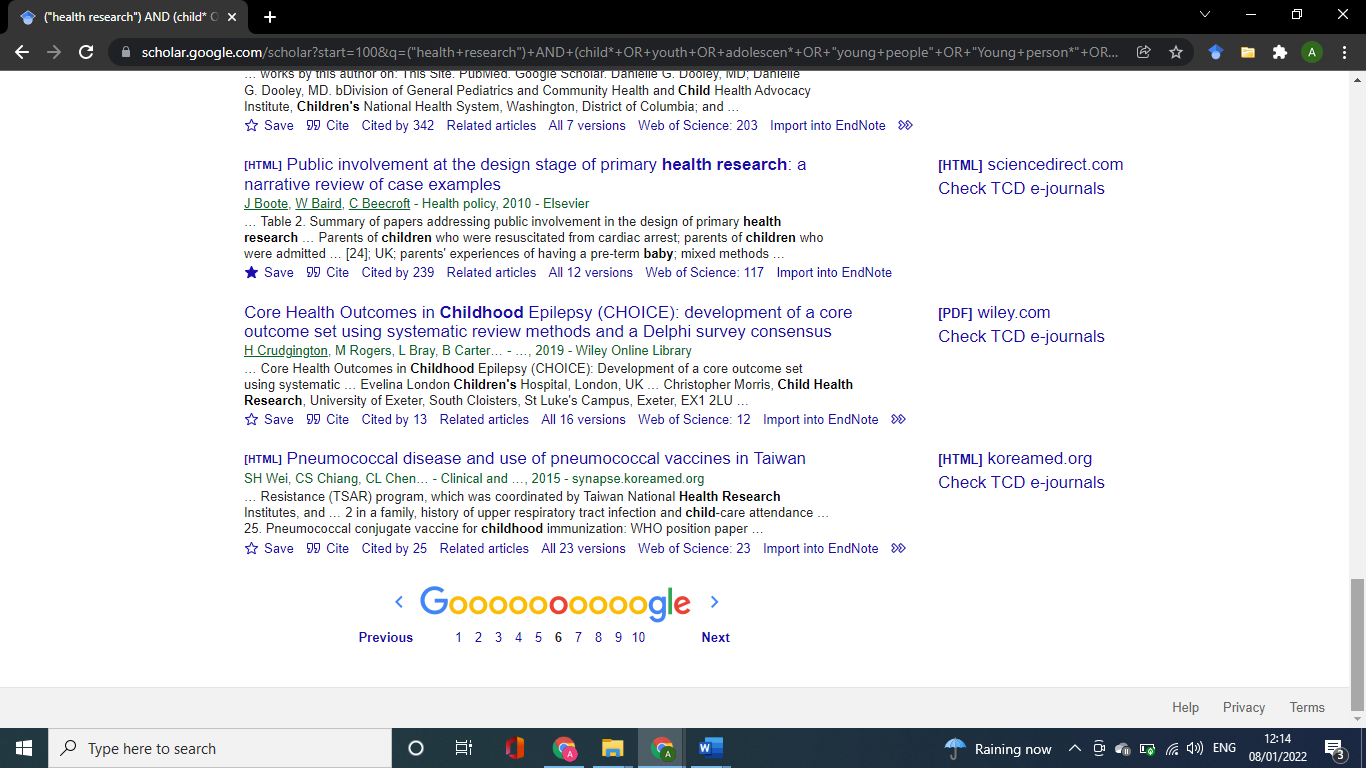 | | | | | | | | | | | | | | |
| Pg 7 | | | | | | | | | | | | | | |
| 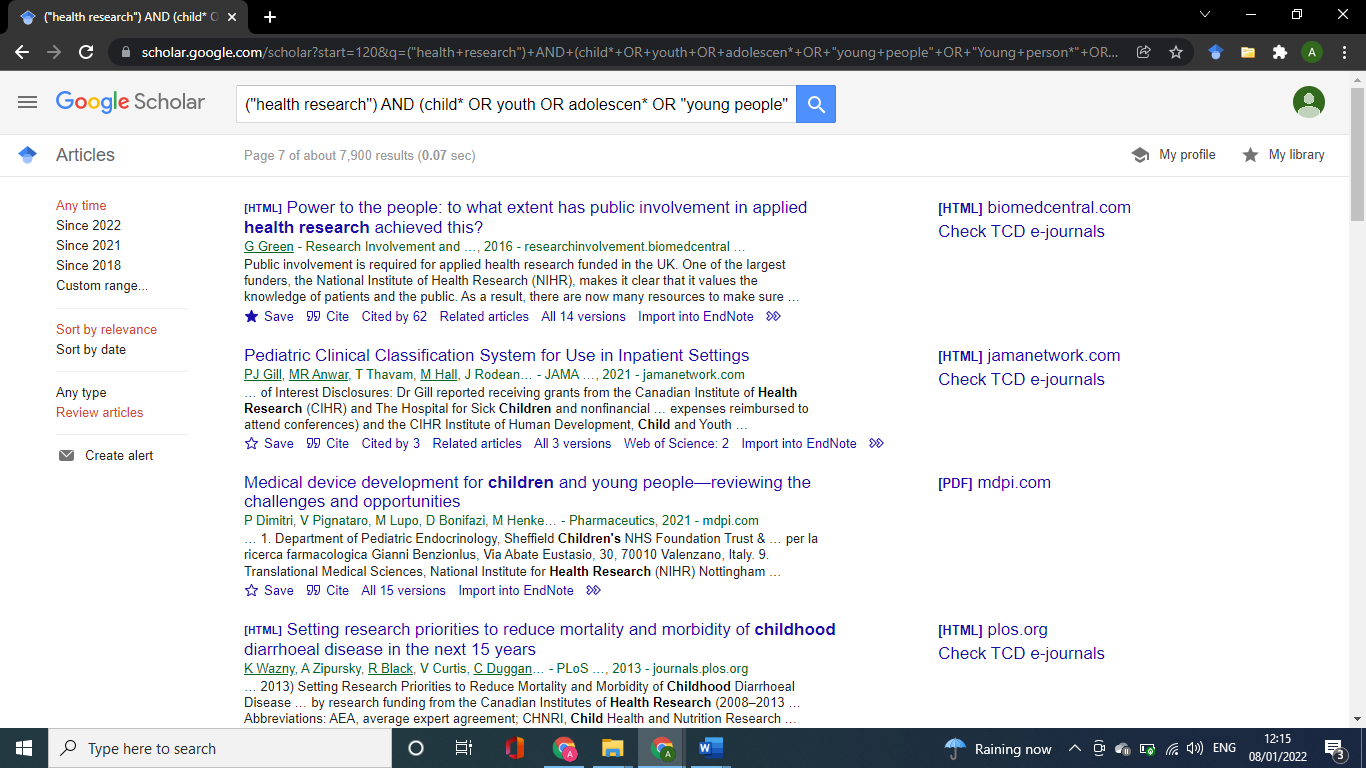 | | | | | | | | | | | | | | |
| 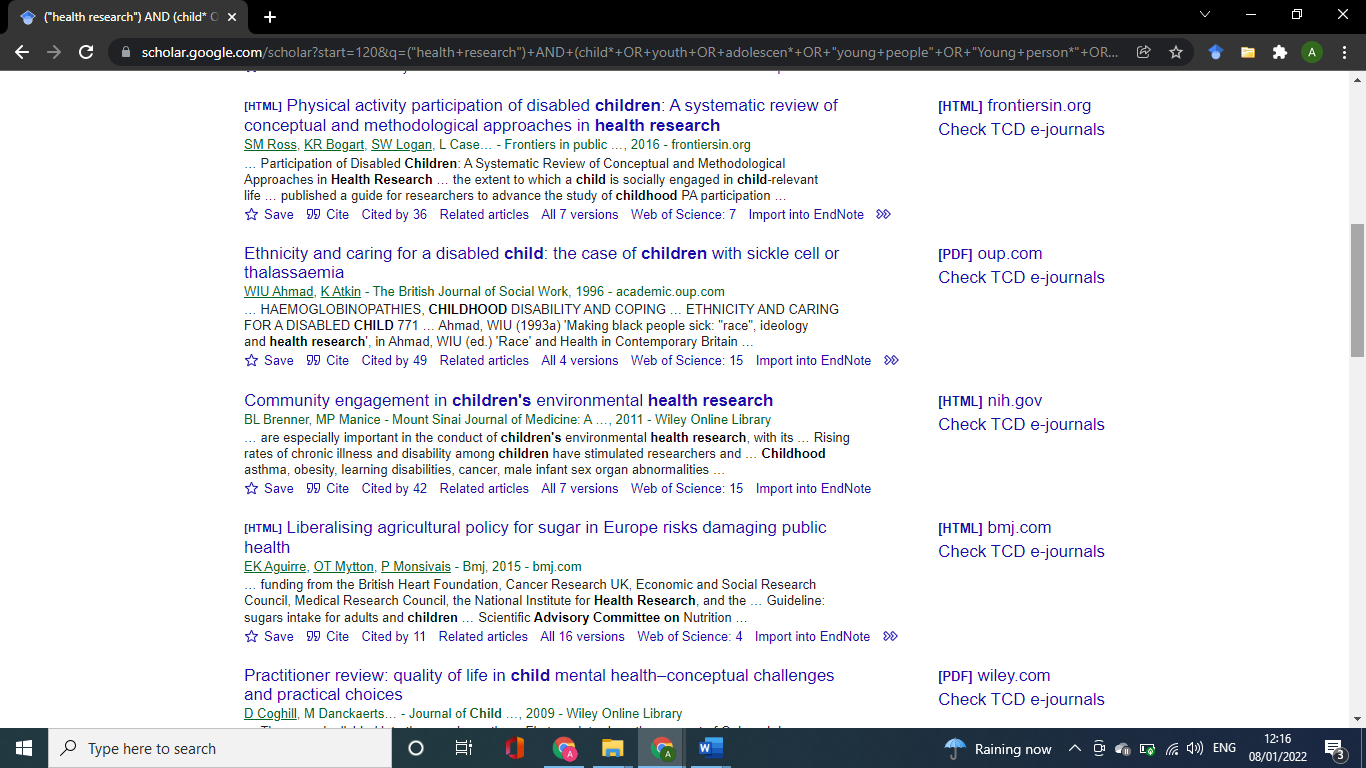 | | | | | | | | | | | | | | |
| 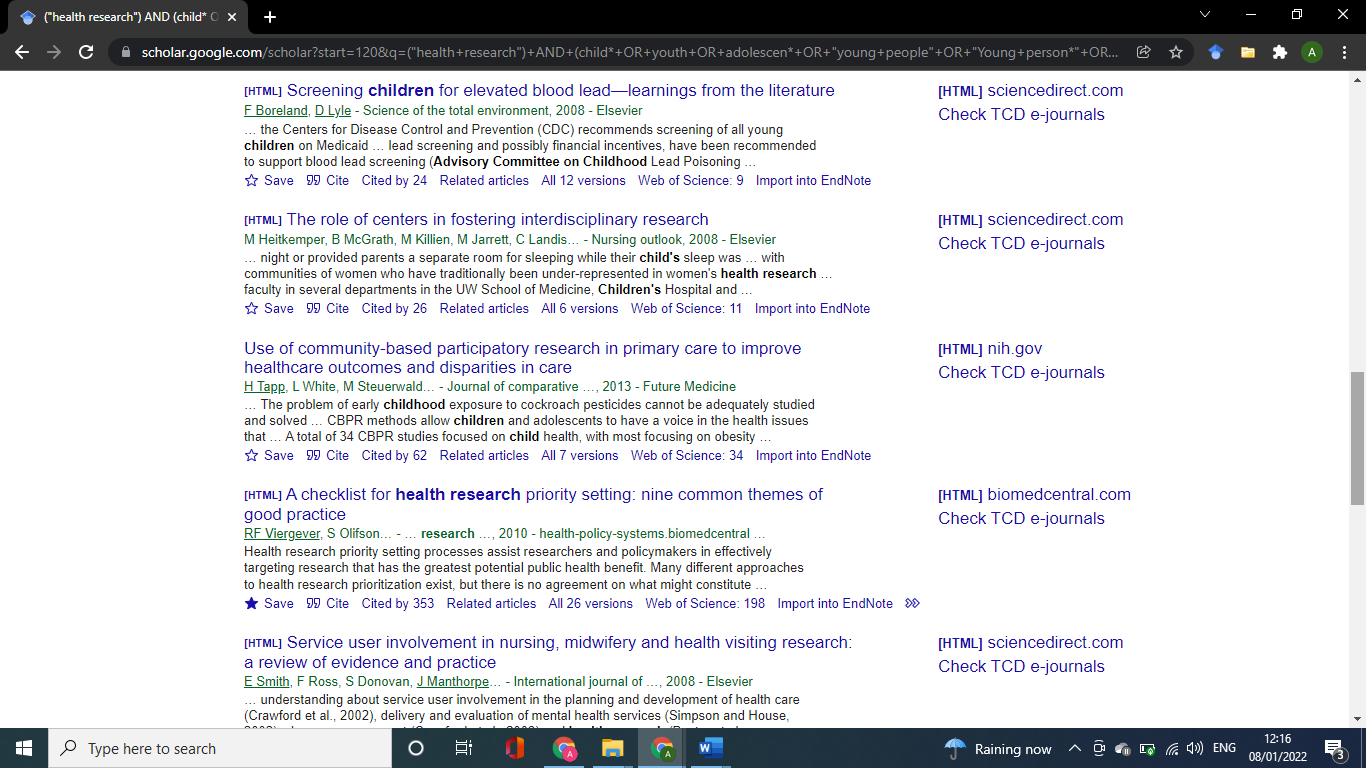 | | | | | | | | | | | | | | |
| 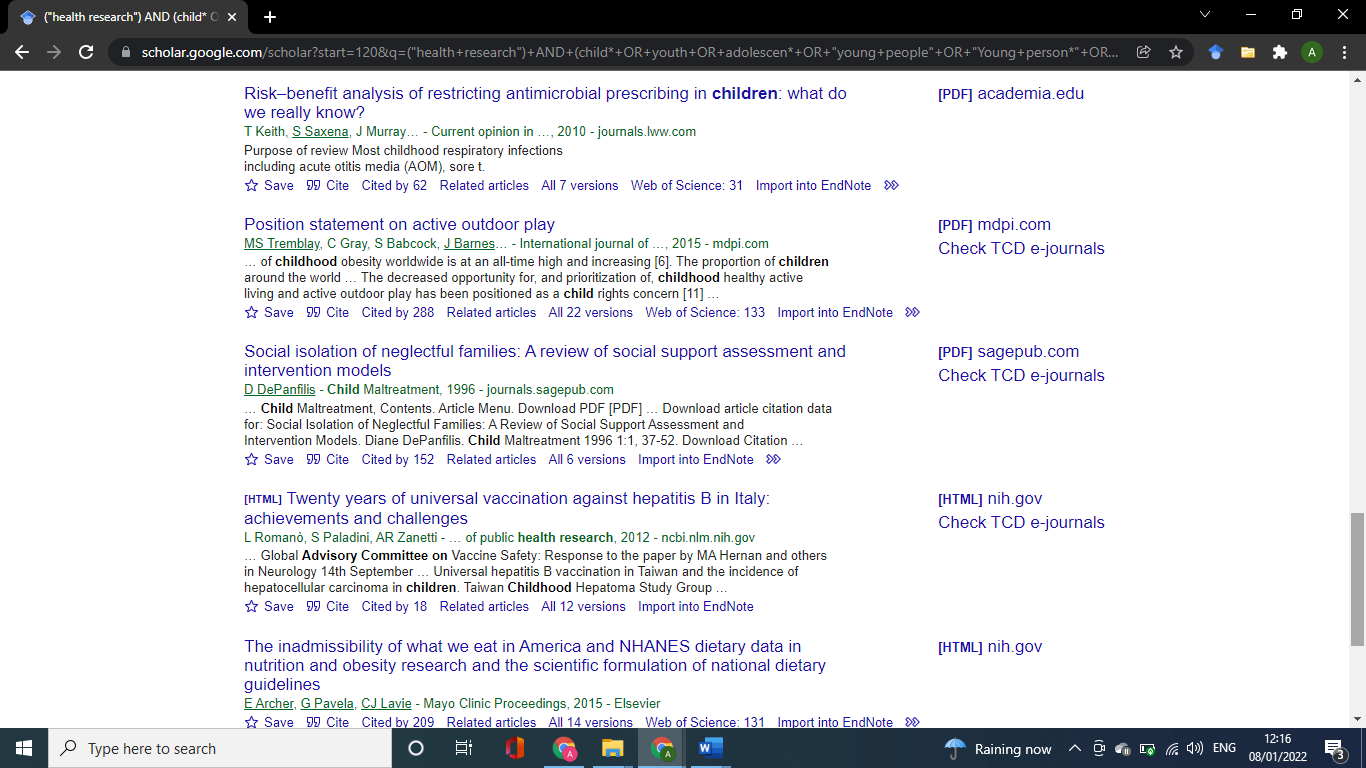 | | | | | | | | | | | | | | |
| 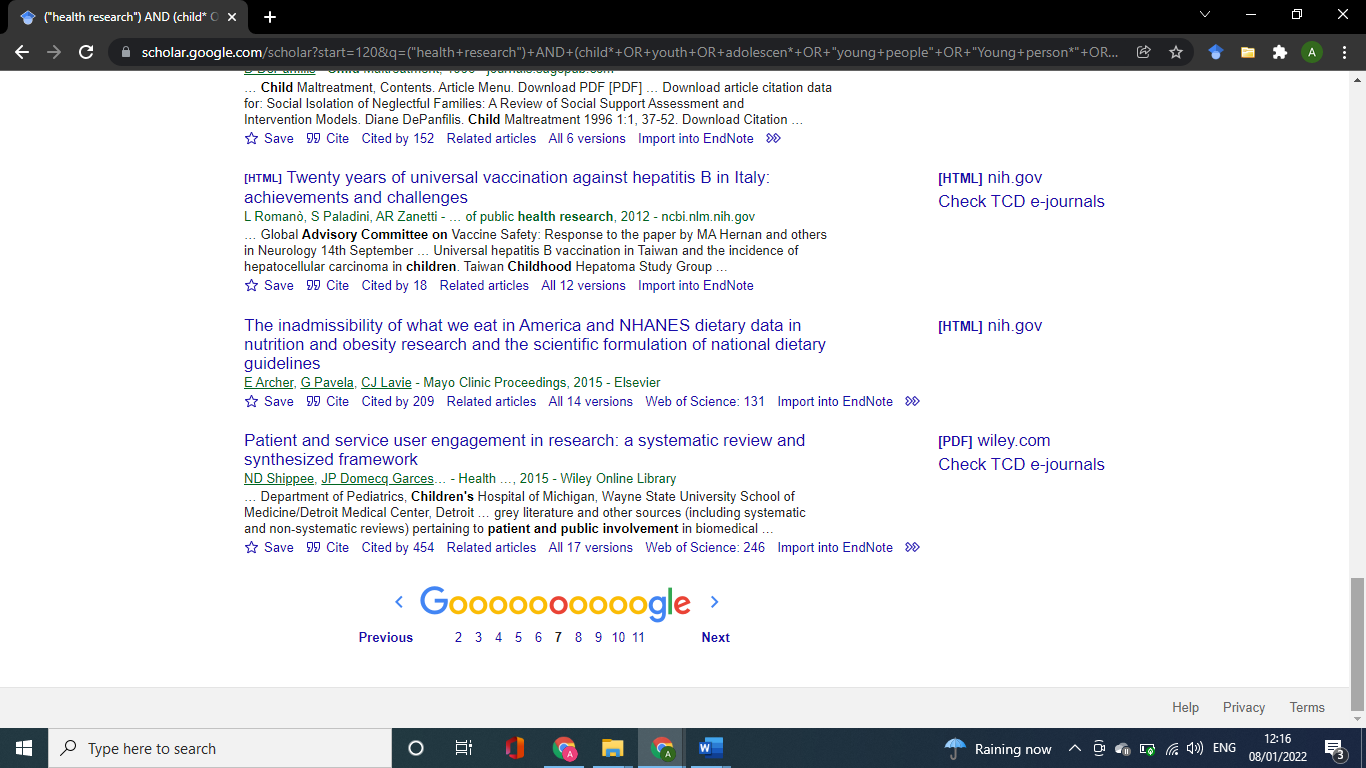 | | | | | | | | | | | | | | |
| Pg 8 | | | | | | | | | | | | | | |
| 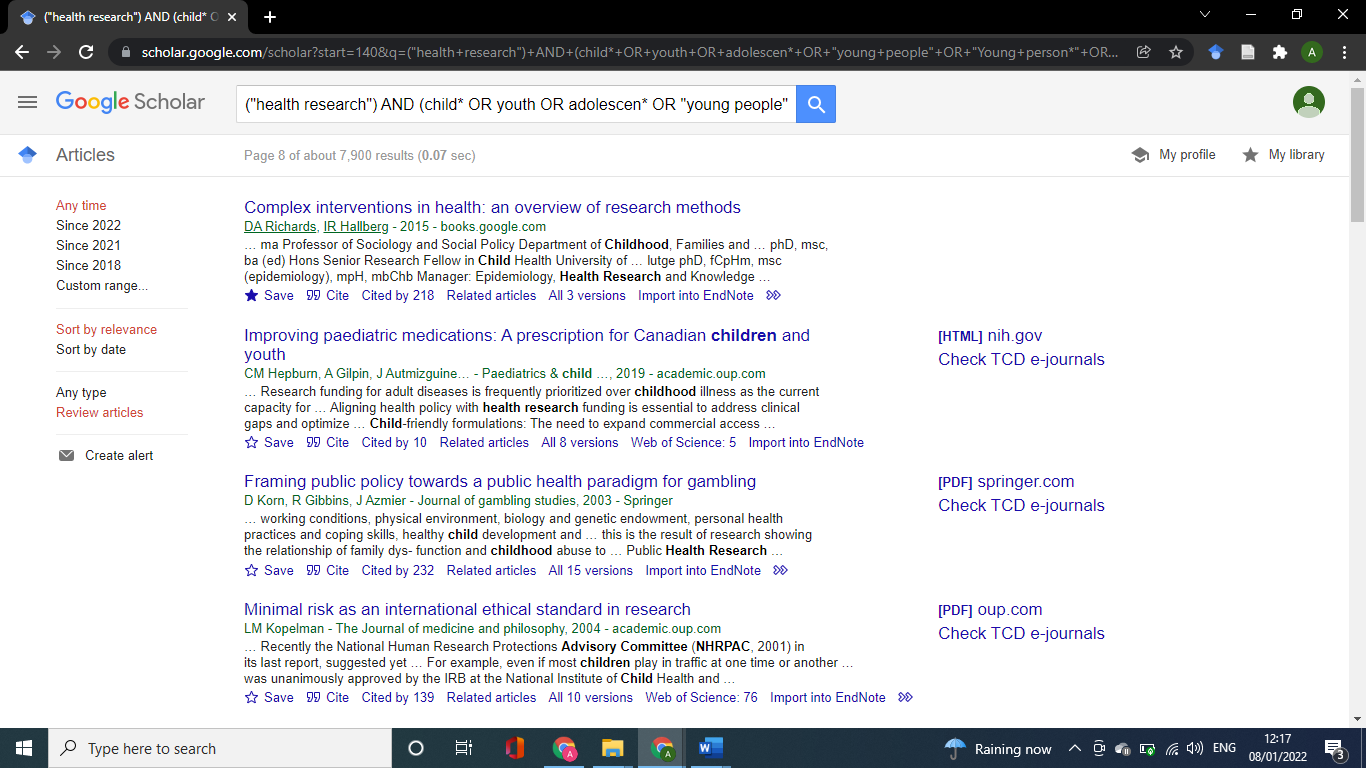 | | | | | | | | | | | | | | |
| 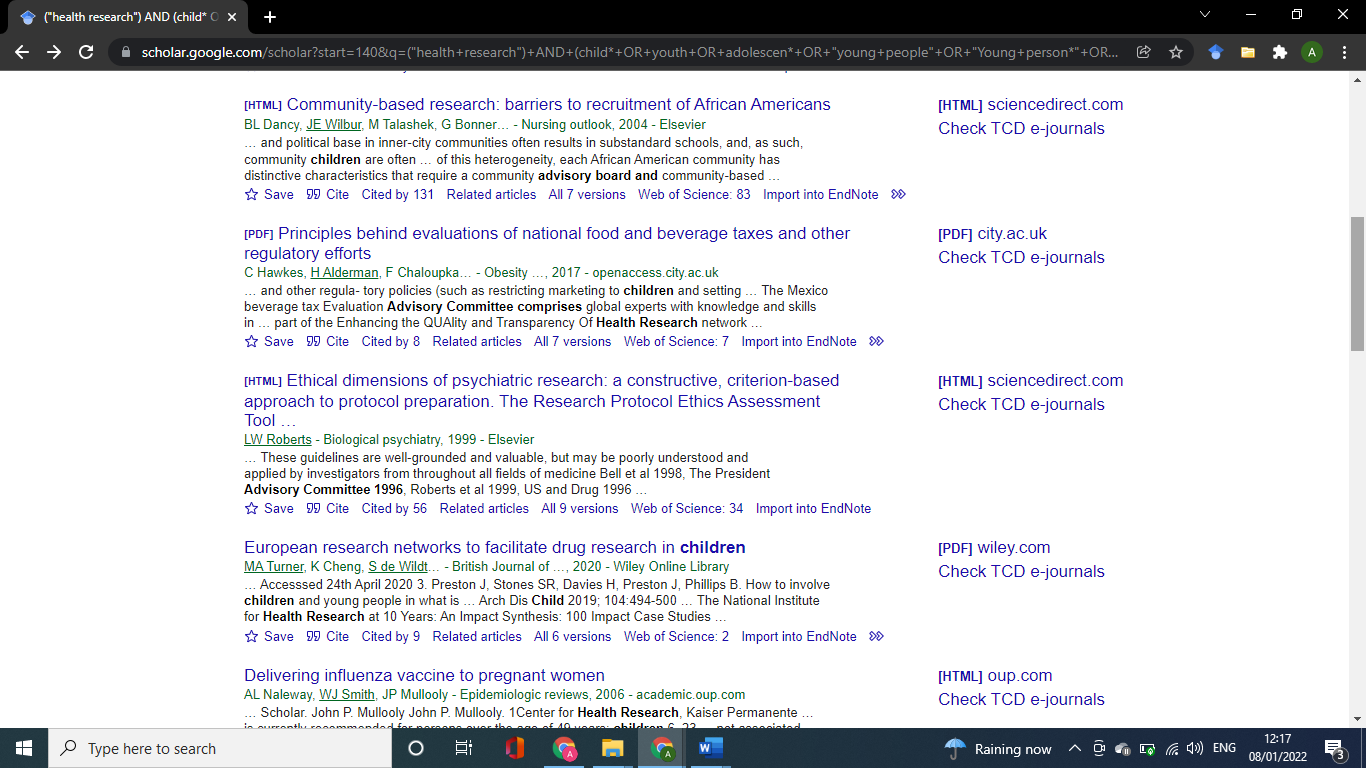 | | | | | | | | | | | | | | |
| 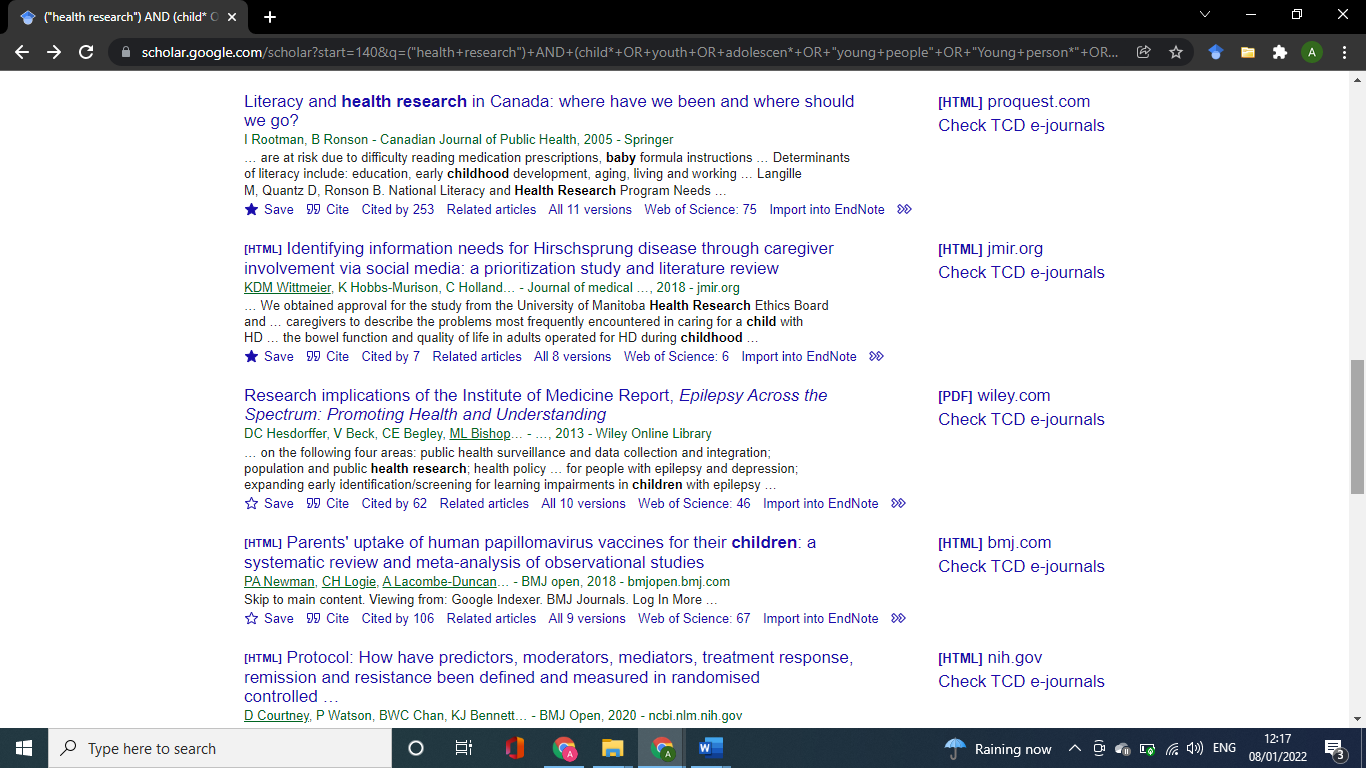 | | | | | | | | | | | | | | |
| 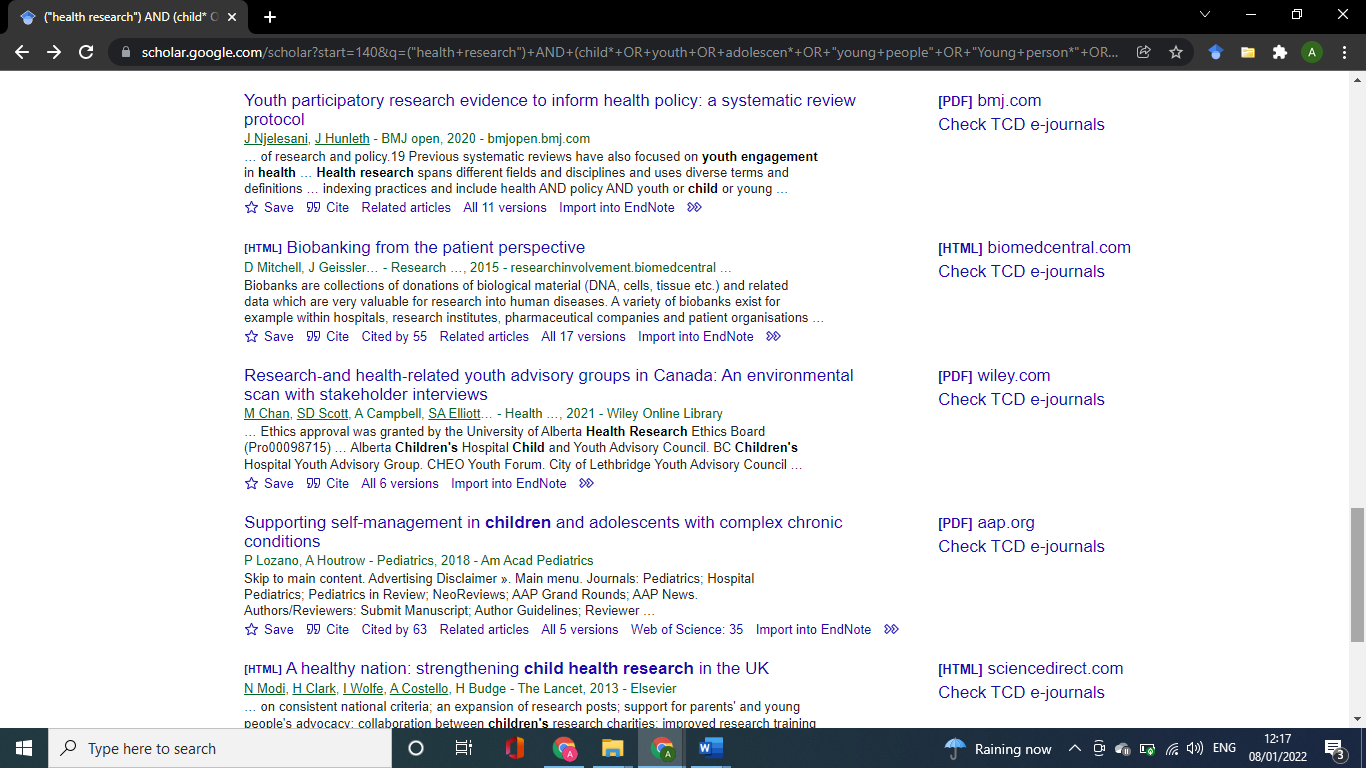 | | | | | | | | | | | | | | |
| 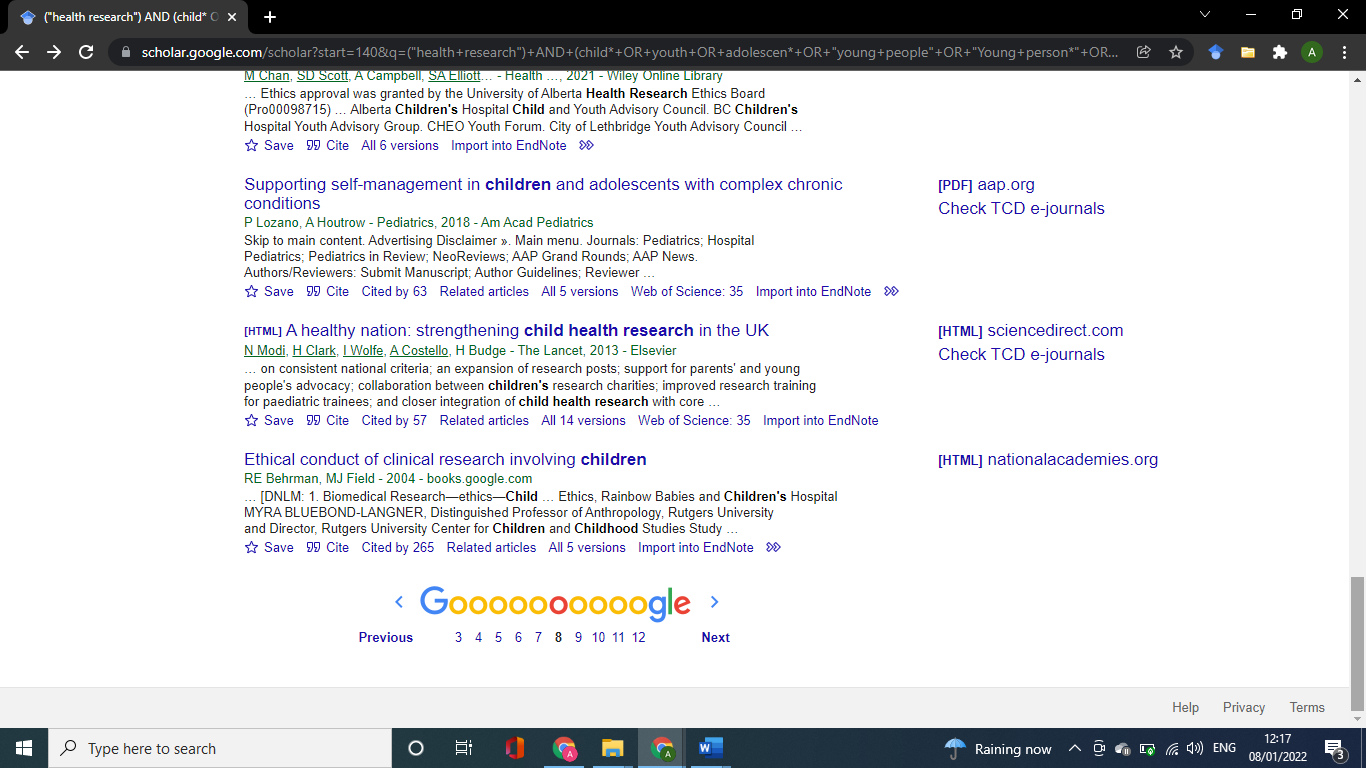 | | | | | | | | | | | | | | |
| Pg 9 | | | | | | | | | | | | | | |
| 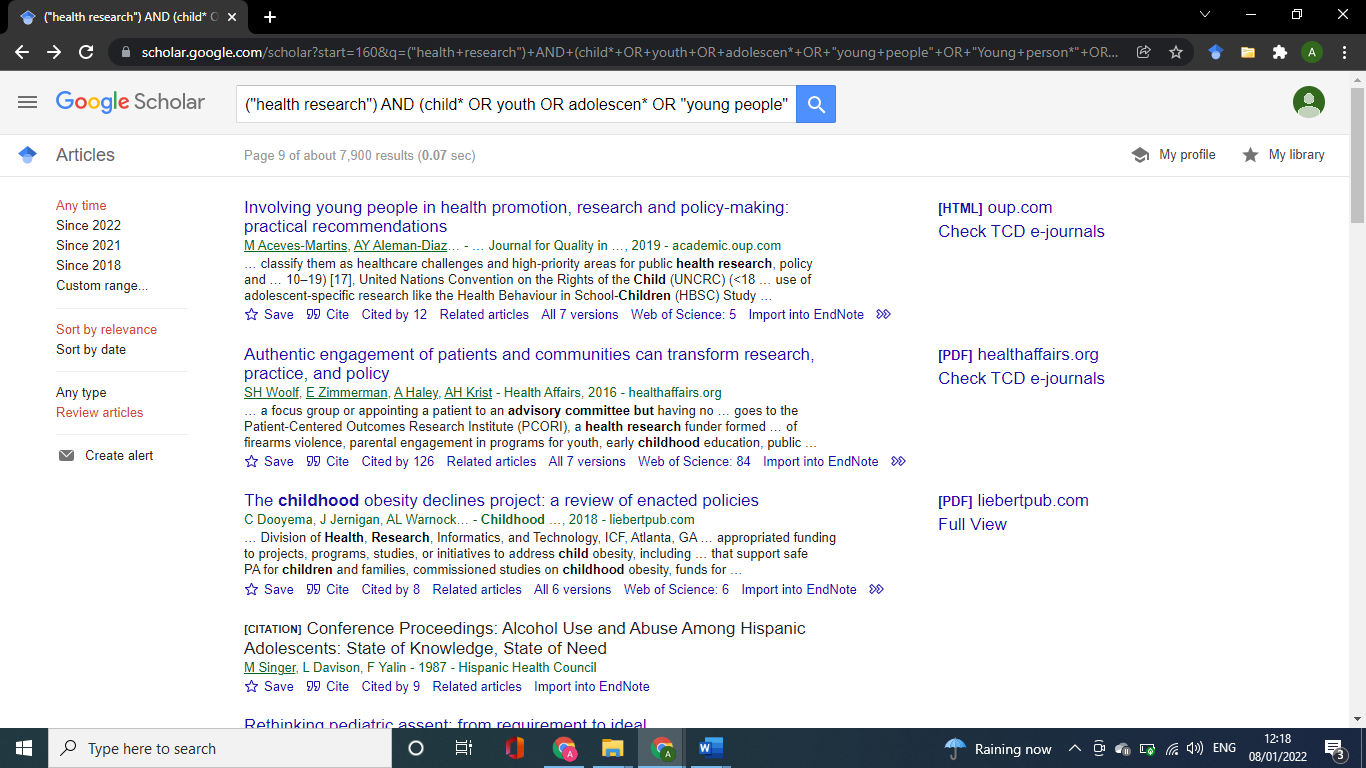 | | | | | | | | | | | | | | |
| 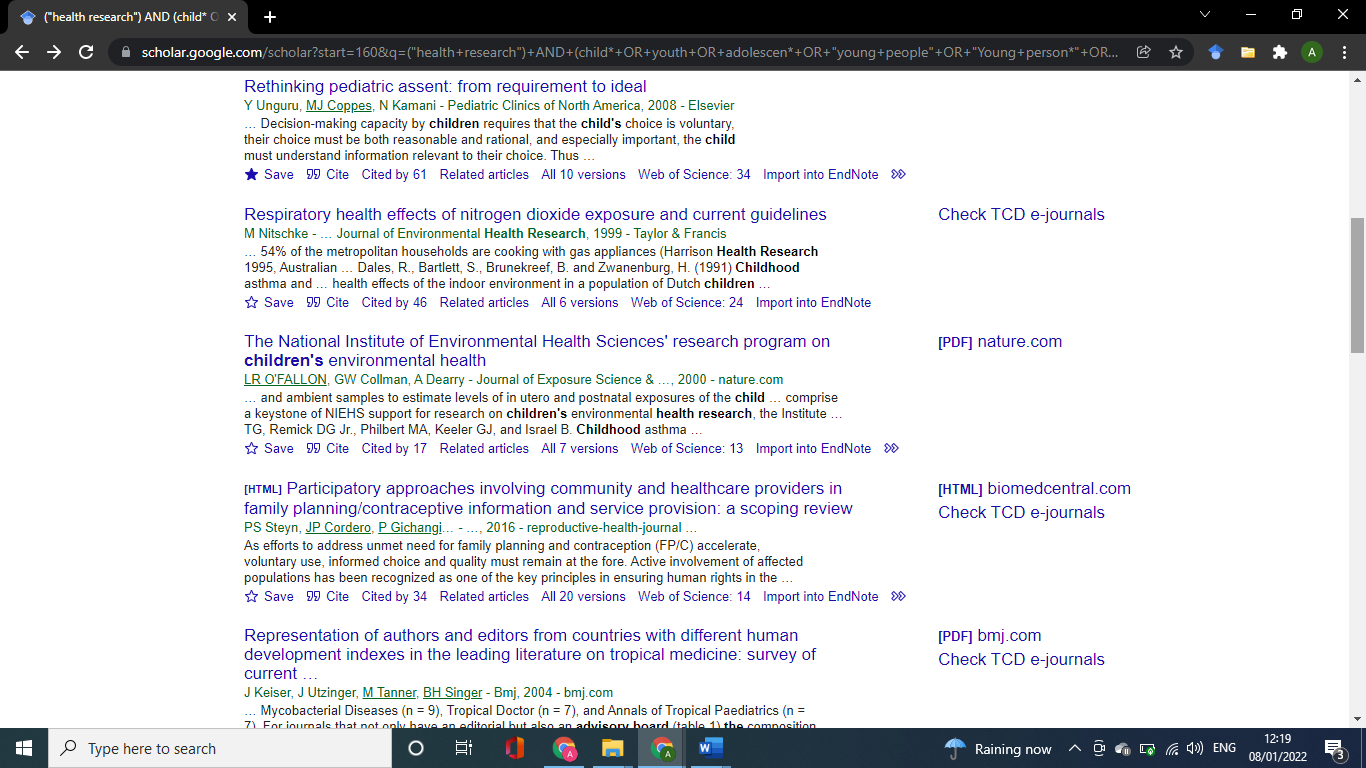 | | | | | | | | | | | | | | |
| 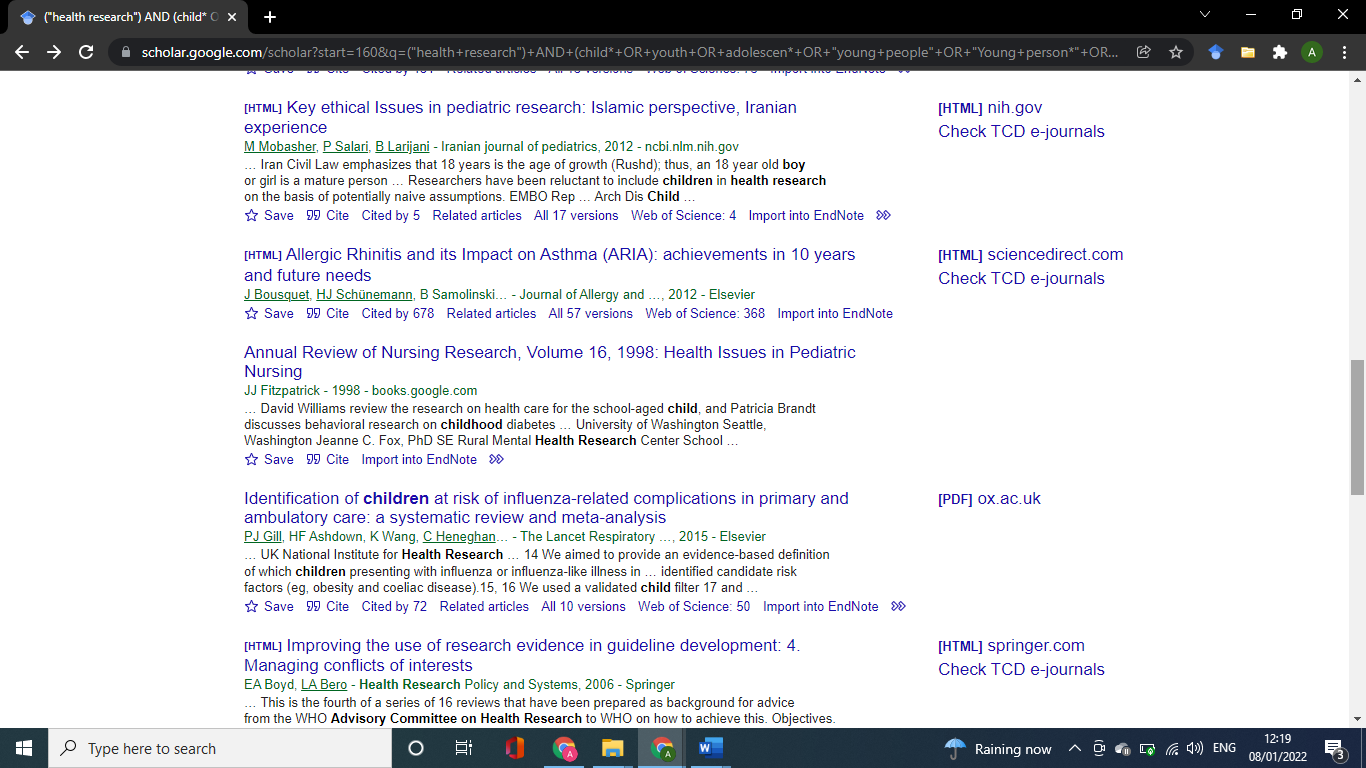 | | | | | | | | | | | | | | |
| 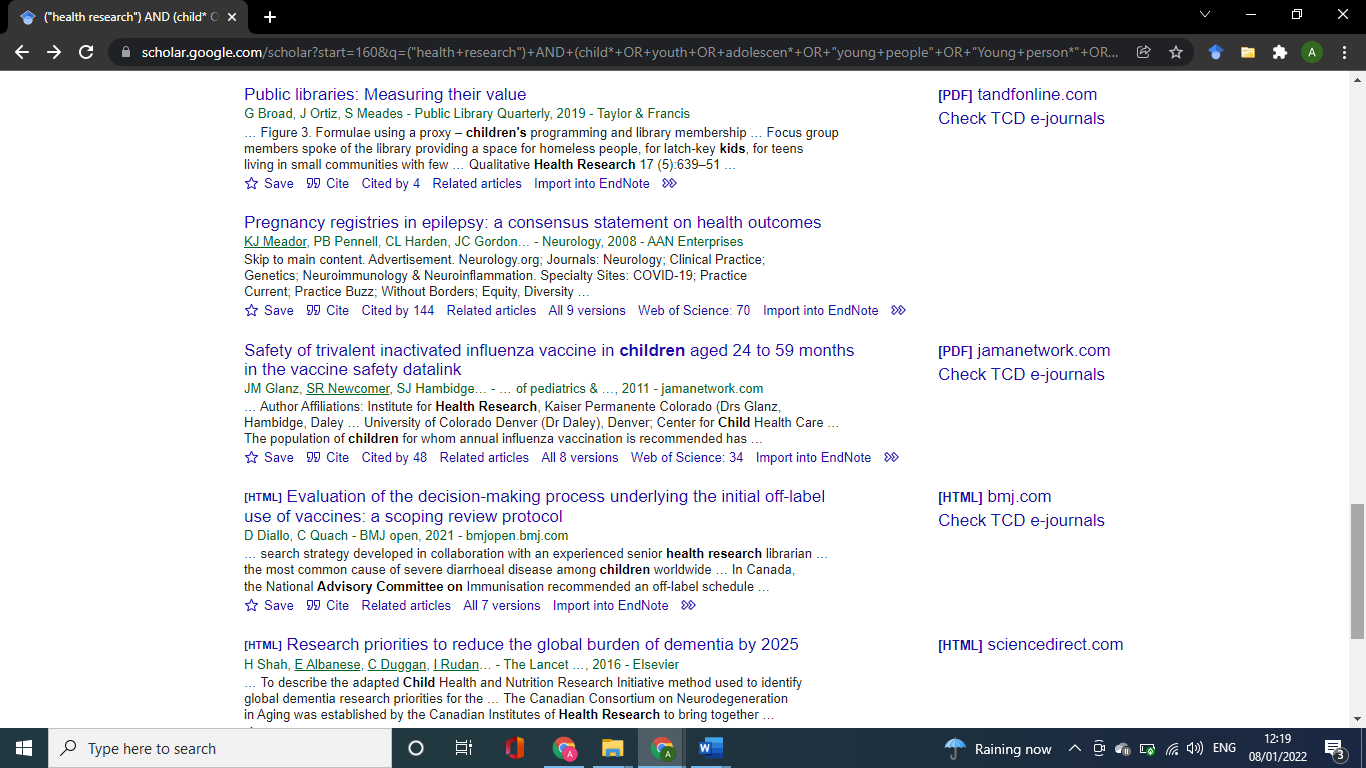 | | | | | | | | | | | | | | |
| 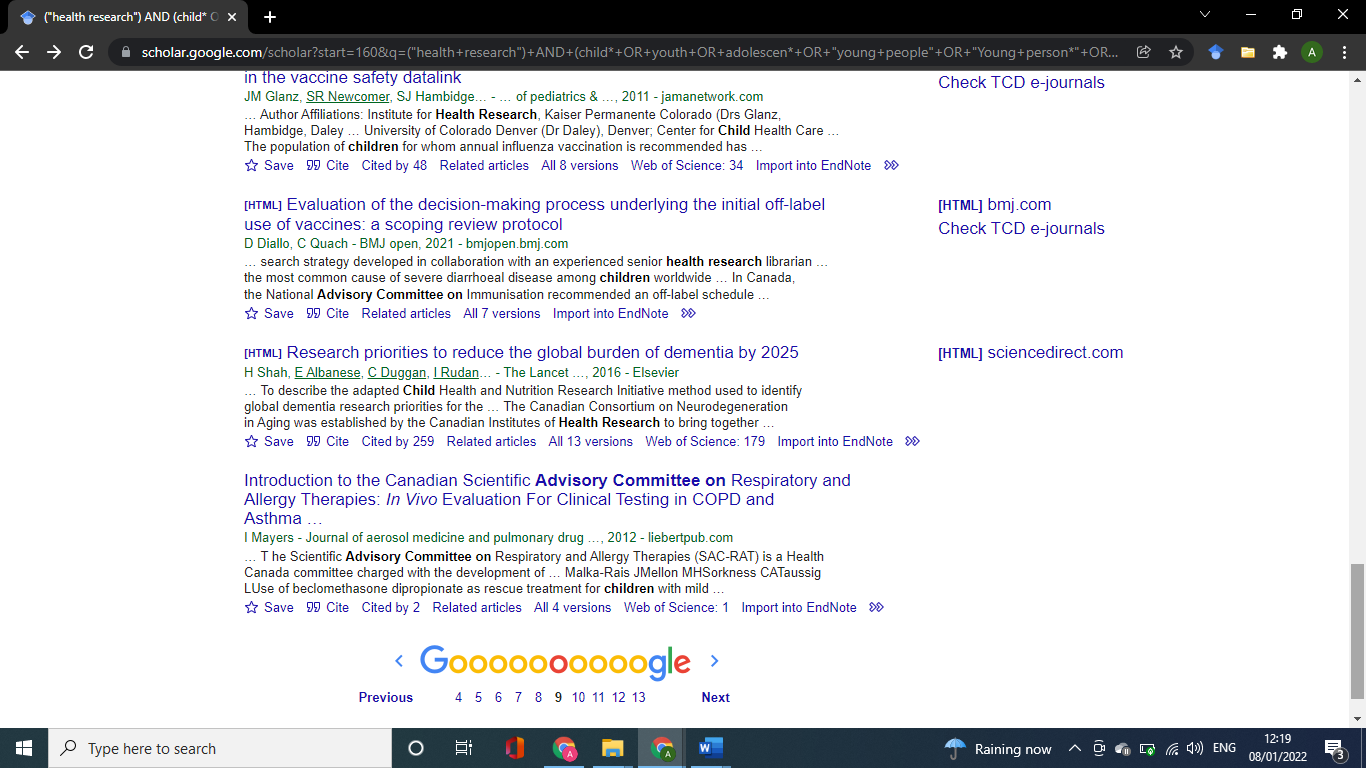 | | | | | | | | | | | | | | |
| Pg 10 | | | | | | | | | | | | | | |
| 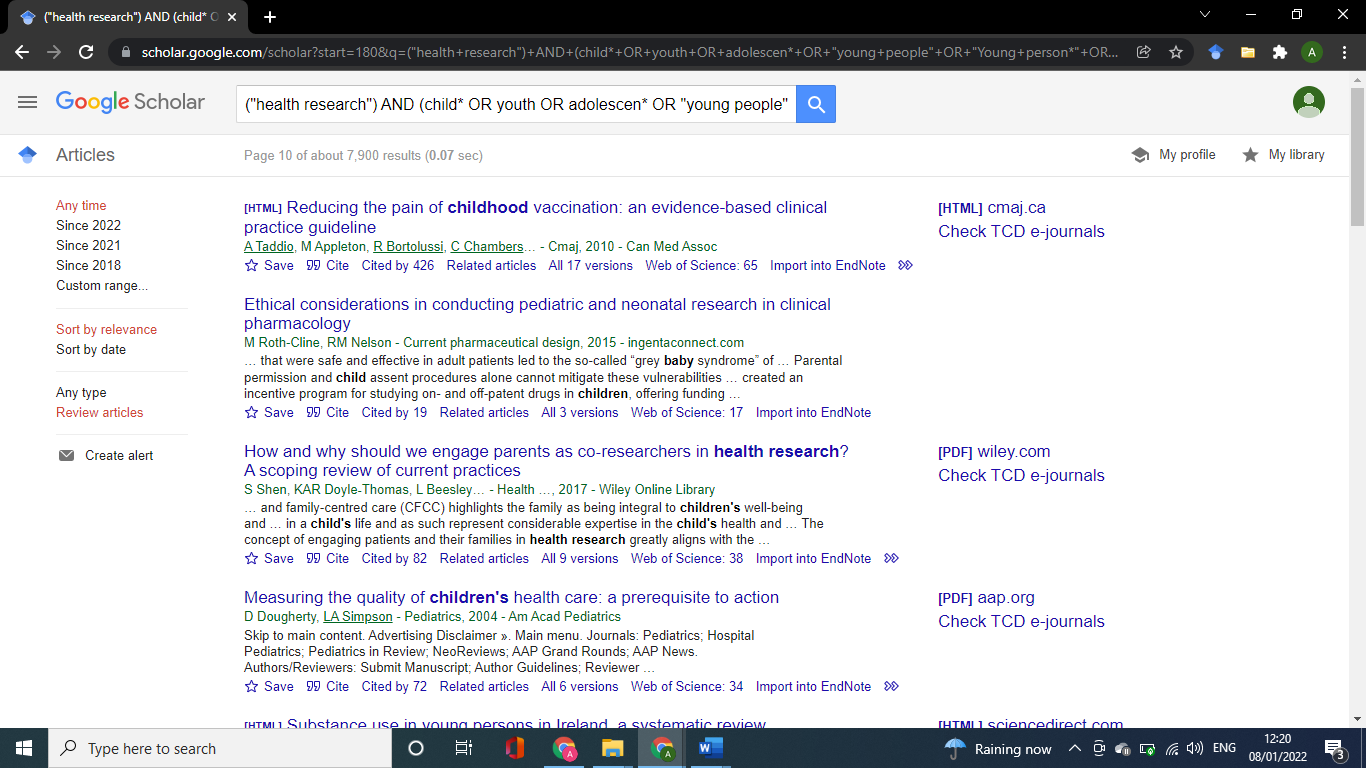 | | | | | | | | | | | | | | |
| 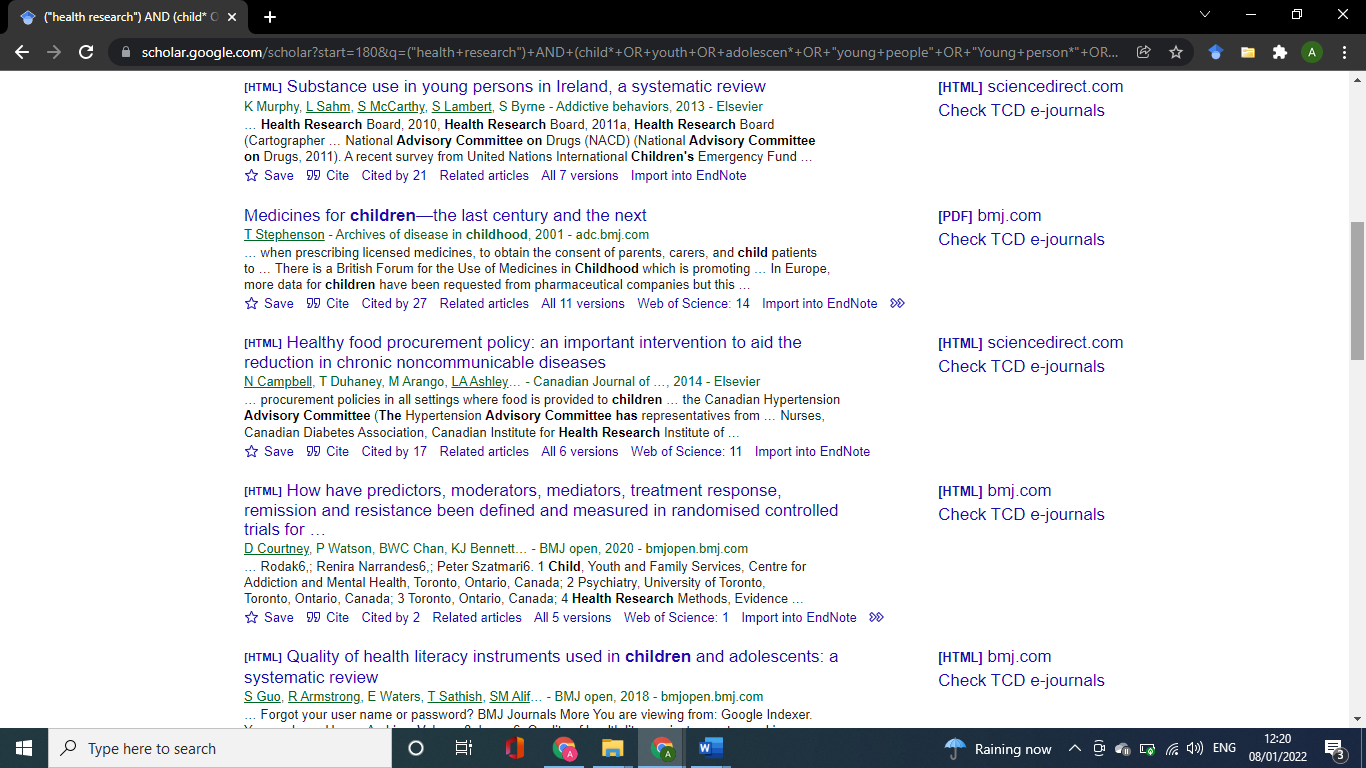 | | | | | | | | | | | | | | |
| 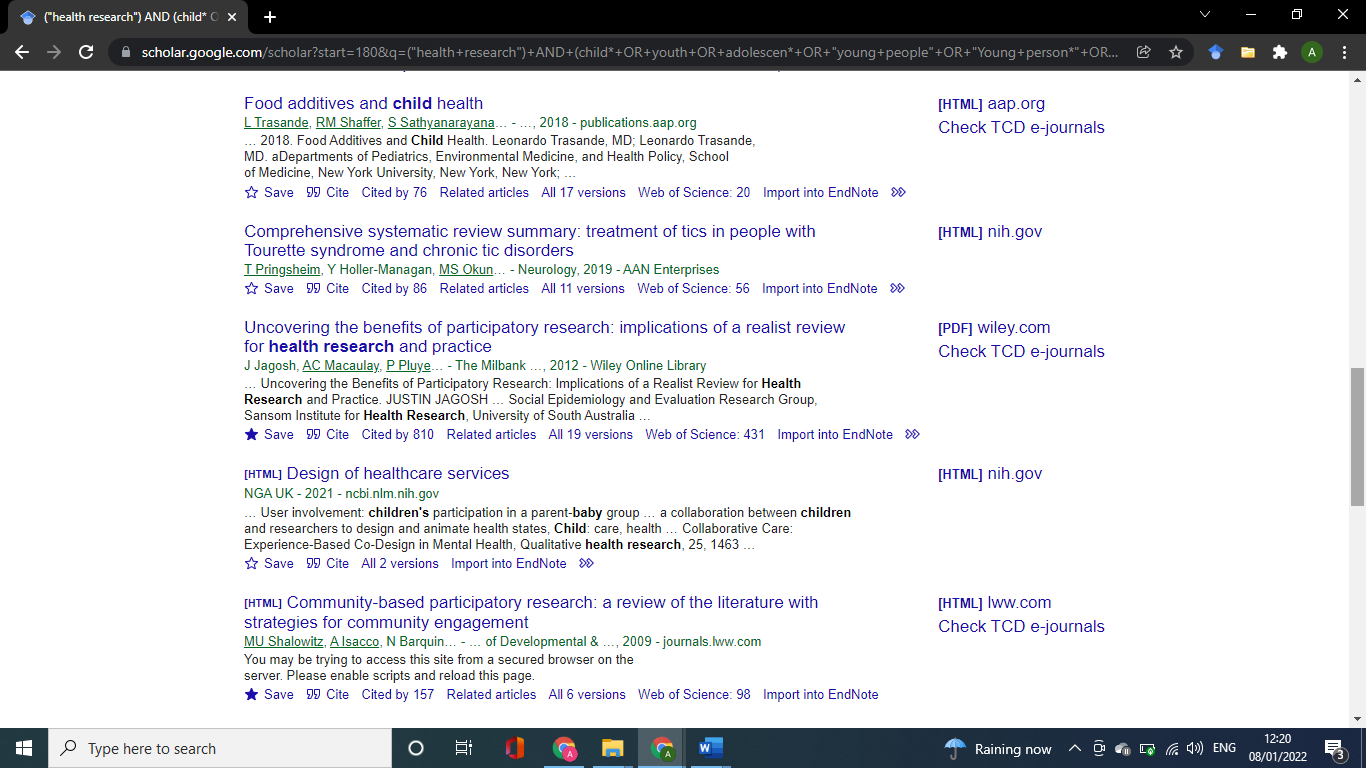 | | | | | | | | | | | | | | |
| 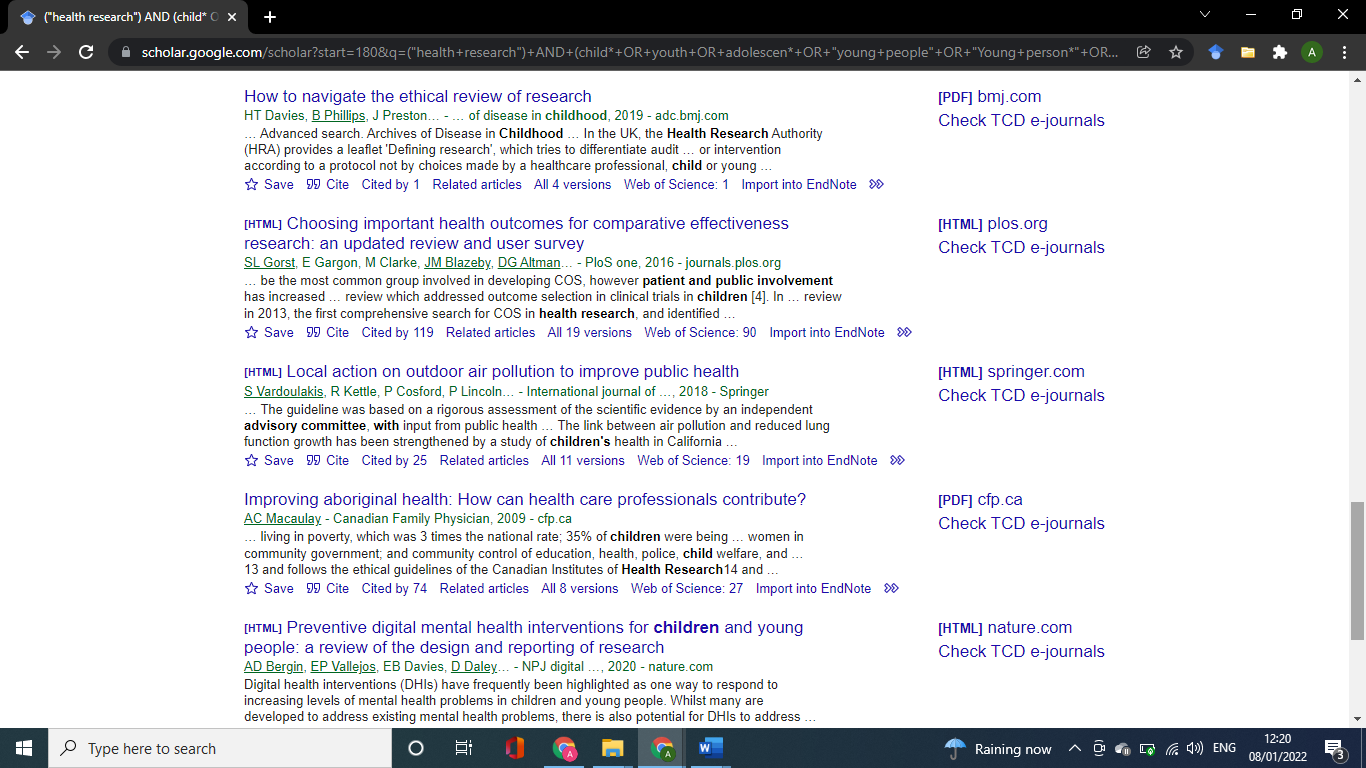 | | | | | | | | | | | | | | |
|  | | | | | | | | | | | | | | |
| 3 | | | | | ("health research") AND (child* OR youth OR adolescen* OR "young people" OR "Young person*" OR "Young adult*" OR teen* OR juven*) AND ("public and patient involvement" OR "public patient involvement" OR "community based participatory research") | | | | | 1740 | | | | |
| Pg 1 | | | | | | | | | | | | | | |
|  | | | | | | | | | | | | | | |
|  | | | | | | | | | | | | | | |
|  | | | | | | | | | | | | | | |
|  | | | | | | | | | | | | | | |
|  | | | | | | | | | | | | | | |
| Pg 2 | | | | | | | | | | | | | | |
|  | | | | | | | | | | | | | | |
|  | | | | | | | | | | | | | | |
|  | | | | | | | | | | | | | | |
|  | | | | | | | | | | | | | | |
|  | | | | | | | | | | | | | | |
| Pg 3 | | | | | | | | | | | | | | |
|  | | | | | | | | | | | | | | |
|  | | | | | | | | | | | | | | |
|  | | | | | | | | | | | | | | |
|  | | | | | | | | | | | | | | |
|  | | | | | | | | | | | | | | |
| Pg 4 | | | | | | | | | | | | | | |
|  | | | | | | | | | | | | | | |
|  | | | | | | | | | | | | | | |
|  | | | | | | | | | | | | | | |
|  | | | | | | | | | | | | | | |
|  | | | | | | | | | | | | | | |
| Pg 5 | | | | | | | | | | | | | | |
|  | | | | | | | | | | | | | | |
|  | | | | | | | | | | | | | | |
|  | | | | | | | | | | | | | | |
|  | | | | | | | | | | | | | | |
|  | | | | | | | | | | | | | | |
| Pg 6 | | | | | | | | | | | | | | |
|  | | | | | | | | | | | | | | |
|  | | | | | | | | | | | | | | |
|  | | | | | | | | | | | | | | |
|  | | | | | | | | | | | | | | |
|  | | | | | | | | | | | | | | |
| Pg 7 | | | | | | | | | | | | | | |
|  | | | | | | | | | | | | | | |
|  | | | | | | | | | | | | | | |
|  | | | | | | | | | | | | | | |
|  | | | | | | | | | | | | | | |
|  | | | | | | | | | | | | | | |
| Pg 8 | | | | | | | | | | | | | | |
|  | | | | | | | | | | | | | | |
|  | | | | | | | | | | | | | | |
|  | | | | | | | | | | | | | | |
|  | | | | | | | | | | | | | | |
|  | | | | | | | | | | | | | | |
|  | | | | | | | | | | | | | | |
| Pg 9 | | | | | | | | | | | | | | |
|  | | | | | | | | | | | | | | |
|  | | | | | | | | | | | | | | |
|  | | | | | | | | | | | | | | |
|  | | | | | | | | | | | | | | |
|  | | | | | | | | | | | | | | |
| Pg 10 | | | | | | | | | | | | | | |
|  | | | | | | | | | | | | | | |
|  | | | | | | | | | | | | | | |
|  | | | | | | | | | | | | | | |
|  | | | | | | | | | | | | | | |
|  | | | | | | | | | | | | | | |
| 4 | | | | ("health research") AND (child* OR youth OR adolescen* OR "young people" OR "Young person*" OR "Young adult*" OR teen* OR juven*) AND ("youth particip*" OR "adolescent engagement" OR "participatory design" OR "participatory action" OR "needs assessment*") | | | | | | | 3530 | | | |
| Pg1 | | | | | | | | | | | | | | |
|  | | | | | | | | | | | | | | |
|  | | | | | | | | | | | | | | |
|  | | | | | | | | | | | | | | |
|  | | | | | | | | | | | | | | |
|  | | | | | | | | | | | | | | |
|  | | | | | | | | | | | | | | |
| Pg 2 | | | | | | | | | | | | | | |
|  | | | | | | | | | | | | | | |
|  | | | | | | | | | | | | | | |
|  | | | | | | | | | | | | | | |
|  | | | | | | | | | | | | | | |
|  | | | | | | | | | | | | | | |
| Pg 3 | | | | | | | | | | | | | | |
|  | | | | | | | | | | | | | | |
|  | | | | | | | | | | | | | | |
|  | | | | | | | | | | | | | | |
|  | | | | | | | | | | | | | | |
|  | | | | | | | | | | | | | | |
| Pg 4 | | | | | | | | | | | | | | |
|  | | | | | | | | | | | | | | |
|  | | | | | | | | | | | | | | |
|  | | | | | | | | | | | | | | |
|  | | | | | | | | | | | | | | |
|  | | | | | | | | | | | | | | |
|  | | | | | | | | | | | | | | |
| Pg 5 | | | | | | | | | | | | | | |
|  | | | | | | | | | | | | | | |
|  | | | | | | | | | | | | | | |
|  | | | | | | | | | | | | | | |
|  | | | | | | | | | | | | | | |
|  | | | | | | | | | | | | | | |
|  | | | | | | | | | | | | | | |
| Pg 6 | | | | | | | | | | | | | | |
|  | | | | | | | | | | | | | | |
|  | | | | | | | | | | | | | | |
|  | | | | | | | | | | | | | | |
|  | | | | | | | | | | | | | | |
|  | | | | | | | | | | | | | | |
| Pg 7 | | | | | | | | | | | | | | |
|  | | | | | | | | | | | | | | |
|  | | | | | | | | | | | | | | |
|  | | | | | | | | | | | | | | |
|  | | | | | | | | | | | | | | |
|  | | | | | | | | | | | | | | |
| Pg 8 | | | | | | | | | | | | | | |
|  | | | | | | | | | | | | | | |
|  | | | | | | | | | | | | | | |
|  | | | | | | | | | | | | | | |
|  | | | | | | | | | | | | | | |
|  | | | | | | | | | | | | | | |
|  | | | | | | | | | | | | | | |
| Pg 9 | | | | | | | | | | | | | | |
|  | | | | | | | | | | | | | | |
|  | | | | | | | | | | | | | | |
|  | | | | | | | | | | | | | | |
|  | | | | | | | | | | | | | | |
|  | | | | | | | | | | | | | | |
|  | | | | | | | | | | | | | | |
| Pg 10 | | | | | | | | | | | | | | |
|  | | | | | | | | | | | | | | |
|  | | | | | | | | | | | | | | |
|  | | | | | | | | | | | | | | |
|  | | | | | | | | | | | | | | |
|  | | | | | | | | | | | | | | |
|  | | | | | | | | | | | | | | |
| 5 | | | ("health research") AND (child* OR youth OR adolescen* OR "young people" OR "Young person*" OR "Young adult*" OR teen* OR juven*) AND ("co produc*" OR "co design" OR "Human centered design" OR "Human centred design" OR "User centered design") | | | | | | | | | | 734 | |
| Pg 1 | | | | | | | | | | | | | | |
|  | | | | | | | | | | | | | | |
|  | | | | | | | | | | | | | | |
|  | | | | | | | | | | | | | | |
|  | | | | | | | | | | | | | | |
|  | | | | | | | | | | | | | | |
|  | | | | | | | | | | | | | | |
| Pg 2 | | | | | | | | | | | | | | |
|  | | | | | | | | | | | | | | |
|  | | | | | | | | | | | | | | |
|  | | | | | | | | | | | | | | |
|  | | | | | | | | | | | | | | |
|  | | | | | | | | | | | | | | |
|  | | | | | | | | | | | | | | |
| Pg 3 | | | | | | | | | | | | | | |
|  | | | | | | | | | | | | | | |
|  | | | | | | | | | | | | | | |
|  | | | | | | | | | | | | | | |
|  | | | | | | | | | | | | | | |
|  | | | | | | | | | | | | | | |
|  | | | | | | | | | | | | | | |
| Pg 4 | | | | | | | | | | | | | | |
|  | | | | | | | | | | | | | | |
|  | | | | | | | | | | | | | | |
|  | | | | | | | | | | | | | | |
|  | | | | | | | | | | | | | | |
|  | | | | | | | | | | | | | | |
| Pg 5 | | | | | | | | | | | | | | |
|  | | | | | | | | | | | | | | |
|  | | | | | | | | | | | | | | |
|  | | | | | | | | | | | | | | |
|  | | | | | | | | | | | | | | |
|  | | | | | | | | | | | | | | |
| Pg 6 | | | | | | | | | | | | | | |
|  | | | | | | | | | | | | | | |
|  | | | | | | | | | | | | | | |
|  | | | | | | | | | | | | | | |
|  | | | | | | | | | | | | | | |
|  | | | | | | | | | | | | | | |
| Pg 7 | | | | | | | | | | | | | | |
|  | | | | | | | | | | | | | | |
|  | | | | | | | | | | | | | | |
|  | | | | | | | | | | | | | | |
|  | | | | | | | | | | | | | | |
|  | | | | | | | | | | | | | | |
|  | | | | | | | | | | | | | | |
| Pg 8 | | | | | | | | | | | | | | |
|  | | | | | | | | | | | | | | |
|  | | | | | | | | | | | | | | |
|  | | | | | | | | | | | | | | |
|  | | | | | | | | | | | | | | |
|  | | | | | | | | | | | | | | |
| Pg 9 | | | | | | | | | | | | | | |
|  | | | | | | | | | | | | | | |
|  | | | | | | | | | | | | | | |
|  | | | | | | | | | | | | | | |
|  | | | | | | | | | | | | | | |
|  | | | | | | | | | | | | | | |
|  | | | | | | | | | | | | | | |
| Pg 10 | | | | | | | | | | | | | | |
|  | | | | | | | | | | | | | | |
|  | | | | | | | | | | | | | | |
|  | | | | | | | | | | | | | | |
|  | | | | | | | | | | | | | | |
|  | | | | | | | | | | | | | | |
|  | | | | | | | | | | | | | | |
| 6 | | ("health research") AND (child* OR youth OR adolescen* OR "young people" OR "Young person*" OR "Young adult*" OR teen* OR juven*) AND ("User centred design" OR "user involvement" OR "peer researcher*" OR "co researcher*" OR "Patient Participation") | | | | | | | | | | | | 1640 |
| Pg 1 | | | | | | | | | | | | | | |
|  | | | | | | | | | | | | | | |
|  | | | | | | | | | | | | | | |
|  | | | | | | | | | | | | | | |
|  | | | | | | | | | | | | | | |
|  | | | | | | | | | | | | | | |
|  | | | | | | | | | | | | | | |
| Pg 2 | | | | | | | | | | | | | | |
|  | | | | | | | | | | | | | | |
|  | | | | | | | | | | | | | | |
|  | | | | | | | | | | | | | | |
|  | | | | | | | | | | | | | | |
|  | | | | | | | | | | | | | | |
|  | | | | | | | | | | | | | | |
| Pg 3 | | | | | | | | | | | | | | |
|  | | | | | | | | | | | | | | |
|  | | | | | | | | | | | | | | |
|  | | | | | | | | | | | | | | |
|  | | | | | | | | | | | | | | |
|  | | | | | | | | | | | | | | |
|  | | | | | | | | | | | | | | |
| Pg 4 | | | | | | | | | | | | | | |
|  | | | | | | | | | | | | | | |
|  | | | | | | | | | | | | | | |
|  | | | | | | | | | | | | | | |
|  | | | | | | | | | | | | | | |
|  | | | | | | | | | | | | | | |
|  | | | | | | | | | | | | | | |
| Pg 5 | | | | | | | | | | | | | | |
|  | | | | | | | | | | | | | | |
|  | | | | | | | | | | | | | | |
|  | | | | | | | | | | | | | | |
|  | | | | | | | | | | | | | | |
|  | | | | | | | | | | | | | | |
|  | | | | | | | | | | | | | | |
| Pg 6 | | | | | | | | | | | | | | |
|  | | | | | | | | | | | | | | |
|  | | | | | | | | | | | | | | |
|  | | | | | | | | | | | | | | |
|  | | | | | | | | | | | | | | |
|  | | | | | | | | | | | | | | |
|  | | | | | | | | | | | | | | |
| Pg 7 | | | | | | | | | | | | | | |
|  | | | | | | | | | | | | | | |
|  | | | | | | | | | | | | | | |
|  | | | | | | | | | | | | | | |
|  | | | | | | | | | | | | | | |
|  | | | | | | | | | | | | | | |
|  | | | | | | | | | | | | | | |
| Pg 8 | | | | | | | | | | | | | | |
|  | | | | | | | | | | | | | | |
|  | | | | | | | | | | | | | | |
|  | | | | | | | | | | | | | | |
|  | | | | | | | | | | | | | | |
|  | | | | | | | | | | | | | | |
| Pg 9 | | | | | | | | | | | | | | |
|  | | | | | | | | | | | | | | |
|  | | | | | | | | | | | | | | |
|  | | | | | | | | | | | | | | |
|  | | | | | | | | | | | | | | |
|  | | | | | | | | | | | | | | |
| Pg 10 | | | | | | | | | | | | | | |
|  | | | | | | | | | | | | | | |
|  | | | | | | | | | | | | | | |
|  | | | | | | | | | | | | | | |
|  | | | | | | | | | | | | | | |
|  | | | | | | | | | | | | | | |
|  | | | | | | | | | | | | | | |
| 7 | ("health research") AND (child* OR youth OR adolescen* OR "young people" OR "Young person*" OR "Young adult*" OR teen* OR juven*) AND ("young researcher*" OR "lived experience") | | | | | | | | | | | 2810 | | |
| Pg 1 | | | | | | | | | | | | | | |
|  | | | | | | | | | | | | | | |
|  | | | | | | | | | | | | | | |
|  | | | | | | | | | | | | | | |
|  | | | | | | | | | | | | | | |
|  | | | | | | | | | | | | | | |
| Pg 2 | | | | | | | | | | | | | | |
|  | | | | | | | | | | | | | | |
|  | | | | | | | | | | | | | | |
|  | | | | | | | | | | | | | | |
|  | | | | | | | | | | | | | | |
|  | | | | | | | | | | | | | | |
|  | | | | | | | | | | | | | | |
| Pg 3 | | | | | | | | | | | | | | |
|  | | | | | | | | | | | | | | |
|  | | | | | | | | | | | | | | |
|  | | | | | | | | | | | | | | |
|  | | | | | | | | | | | | | | |
|  | | | | | | | | | | | | | | |
| Pg 4 | | | | | | | | | | | | | | |
|  | | | | | | | | | | | | | | |
|  | | | | | | | | | | | | | | |
|  | | | | | | | | | | | | | | |
|  | | | | | | | | | | | | | | |
|  | | | | | | | | | | | | | | |
| Pg 5 | | | | | | | | | | | | | | |
|  | | | | | | | | | | | | | | |
|  | | | | | | | | | | | | | | |
|  | | | | | | | | | | | | | | |
|  | | | | | | | | | | | | | | |
|  | | | | | | | | | | | | | | |
| Pg 6 | | | | | | | | | | | | | | |
|  | | | | | | | | | | | | | | |
|  | | | | | | | | | | | | | | |
|  | | | | | | | | | | | | | | |
|  | | | | | | | | | | | | | | |
|  | | | | | | | | | | | | | | |
| Pg 7 | | | | | | | | | | | | | | |
|  | | | | | | | | | | | | | | |
|  | | | | | | | | | | | | | | |
|  | | | | | | | | | | | | | | |
|  | | | | | | | | | | | | | | |
|  | | | | | | | | | | | | | | |
| Pg 8 | | | | | | | | | | | | | | |
|  | | | | | | | | | | | | | | |
|  | | | | | | | | | | | | | | |
|  | | | | | | | | | | | | | | |
|  | | | | | | | | | | | | | | |
|  | | | | | | | | | | | | | | |
| Pg 9 | | | | | | | | | | | | | | |
|  | | | | | | | | | | | | | | |
|  | | | | | | | | | | | | | | |
|  | | | | | | | | | | | | | | |
|  | | | | | | | | | | | | | | |
|  | | | | | | | | | | | | | | |
| Pg 10 | | | | | | | | | | | | | | |
|  | | | | | | | | | | | | | | |
|  | | | | | | | | | | | | | | |
|  | | | | | | | | | | | | | | |
|  | | | | | | | | | | | | | | |
|  | | | | | | | | | | | | | | |
