# Supplementary material for: An umbrella review of reviews on challenges to meaningful adolescent involvement in health research
Source: Health Expect. 2024 Jan 27;27(1):e13980. doi: 10.1111/hex.13980 (PMC10821743; doi:10.1111/hex.13980)
Supplement: Supplementary file 1 — Supporting information. [file HEX-27-e13980-s001.zip › Search record and results/Other sources/PROSPERO/PROSPERO search record.docx]

**PROSPERO searched for relevant reviews on youth involvement**

Search conducted on 6^th^ January 2022

| **Total results** | **123** |
| --- | --- |
| Youth involvement | 2 |
| Youth engagement | 5 |
| youth participation | 6 |
| youth led | 4 |
| MeSH DESCRIPTOR Community-Based Participatory Research EXPLODE ALL TREES | 6 |
| Stakeholder OR participatory OR advisory  (Filter=search in title) | 88 |
| public and patient involvement  (Filter=search in title) | 3 |
| co-production OR human-centered design  (Filter=search in title) | 9 |
| peer researcher OR young researcher OR co-researcher  (Filter=search in title) | 0 |
| **Duplicates** | 8 |
| **Ineligible** | 106 |
| **Unclear and not retrieved** | 2 |
| **Eligible** | 7 |
| **Authors of eligible reviews contacted** | 7 |
| Authors didn’t respond | 3 |
| Refused to share data | 4 |
