# Supplementary material for: An umbrella review of reviews on challenges to meaningful adolescent involvement in health research
Source: Health Expect. 2024 Jan 27;27(1):e13980. doi: 10.1111/hex.13980 (PMC10821743; doi:10.1111/hex.13980)
Supplement: Supplementary file 1 — Supporting information. [file HEX-27-e13980-s001.zip › Search record and results/Other sources/PROSPERO/PROSPERO search - screenshots.docx]

| Youth involvement=2  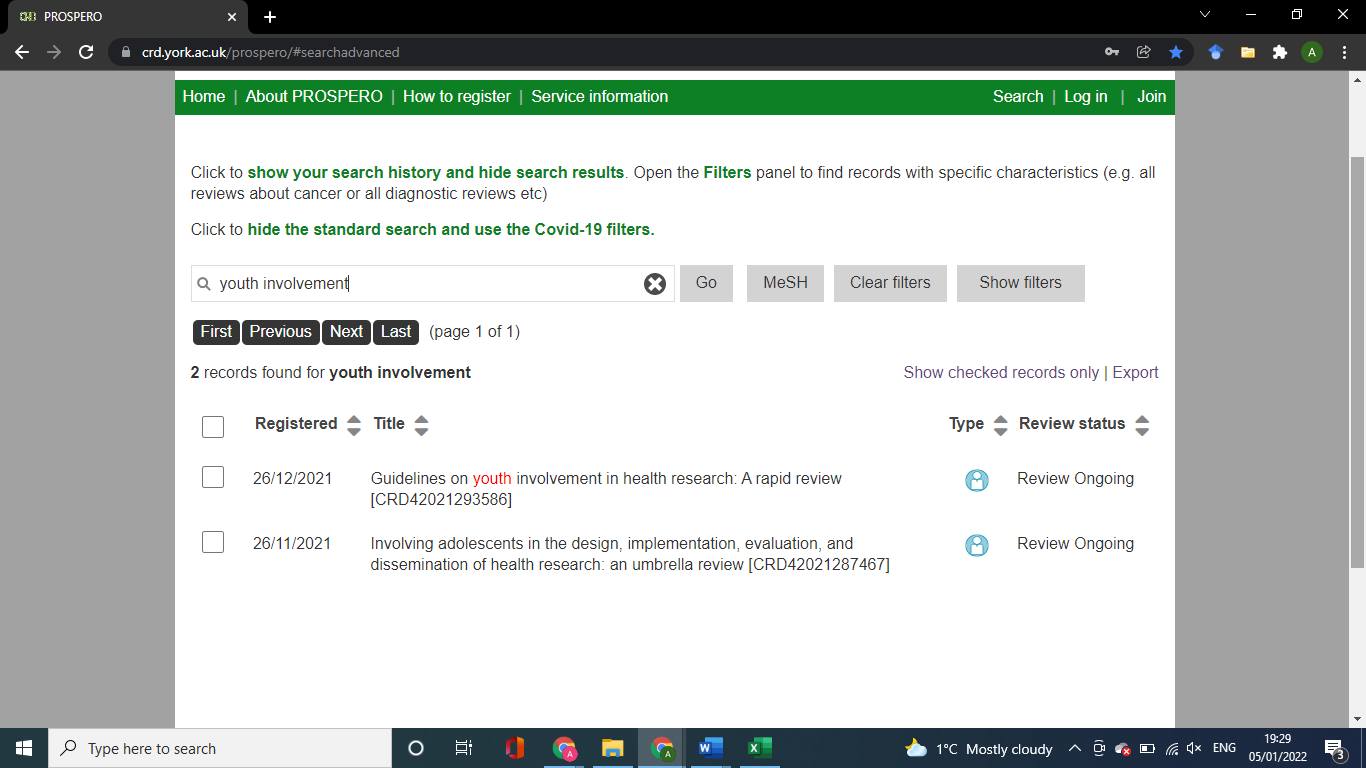  Status: Both reviews are ours. |
| --- |
| Youth engagement=5  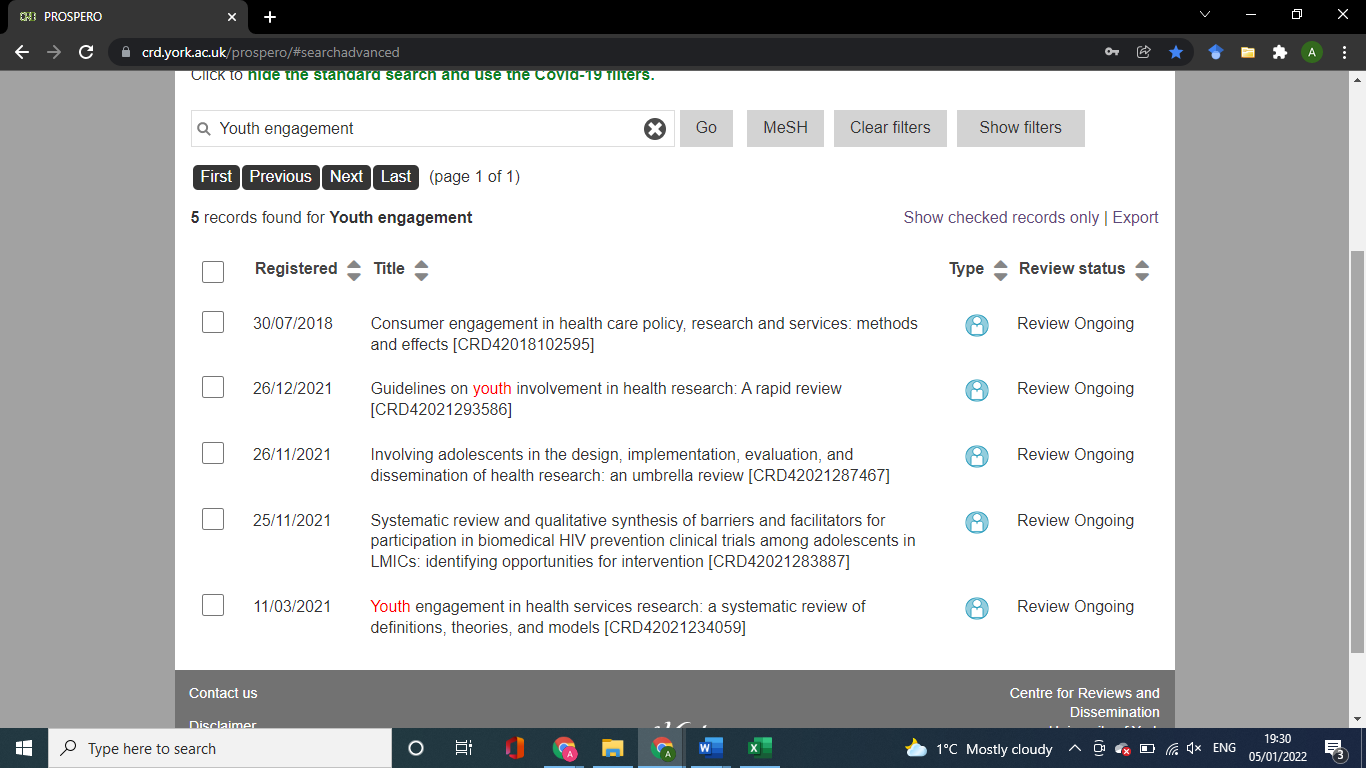 |
| Youth participation= 6  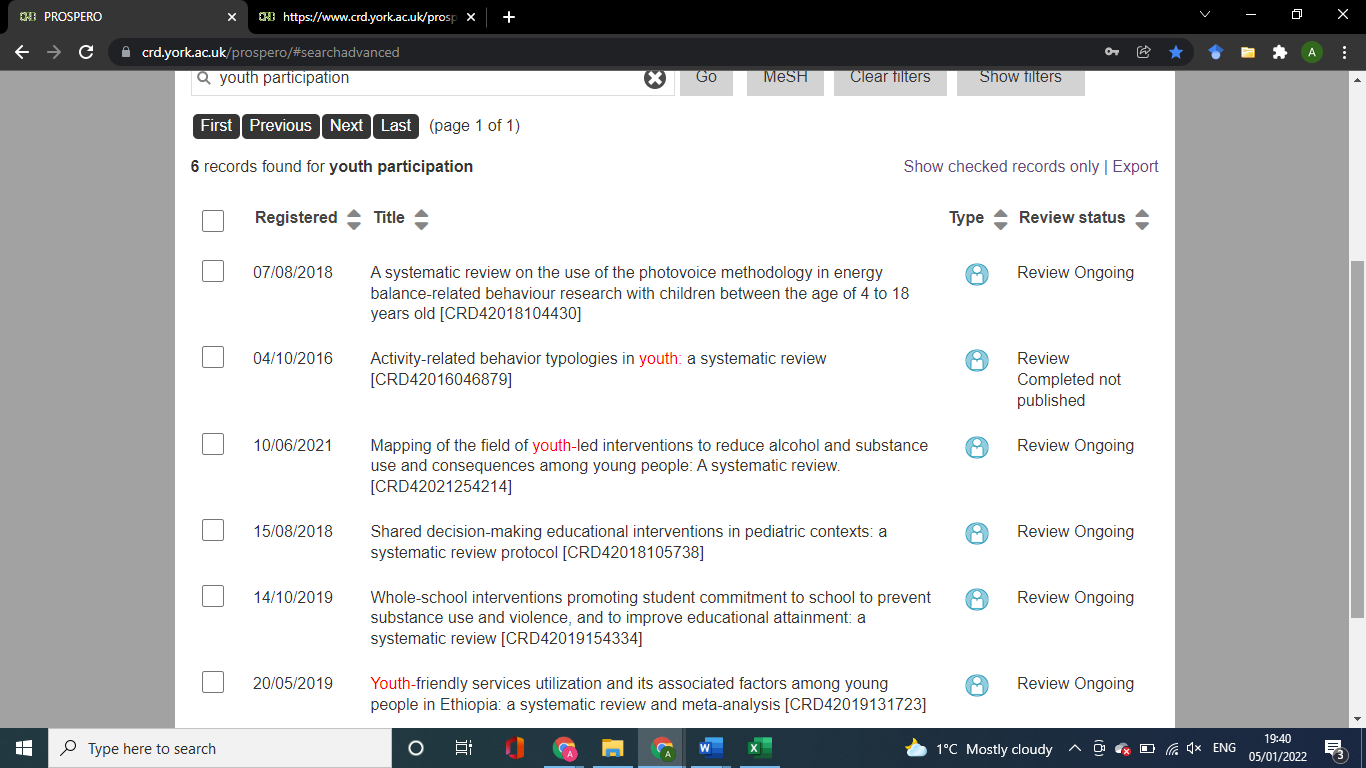 |
| youth led=4  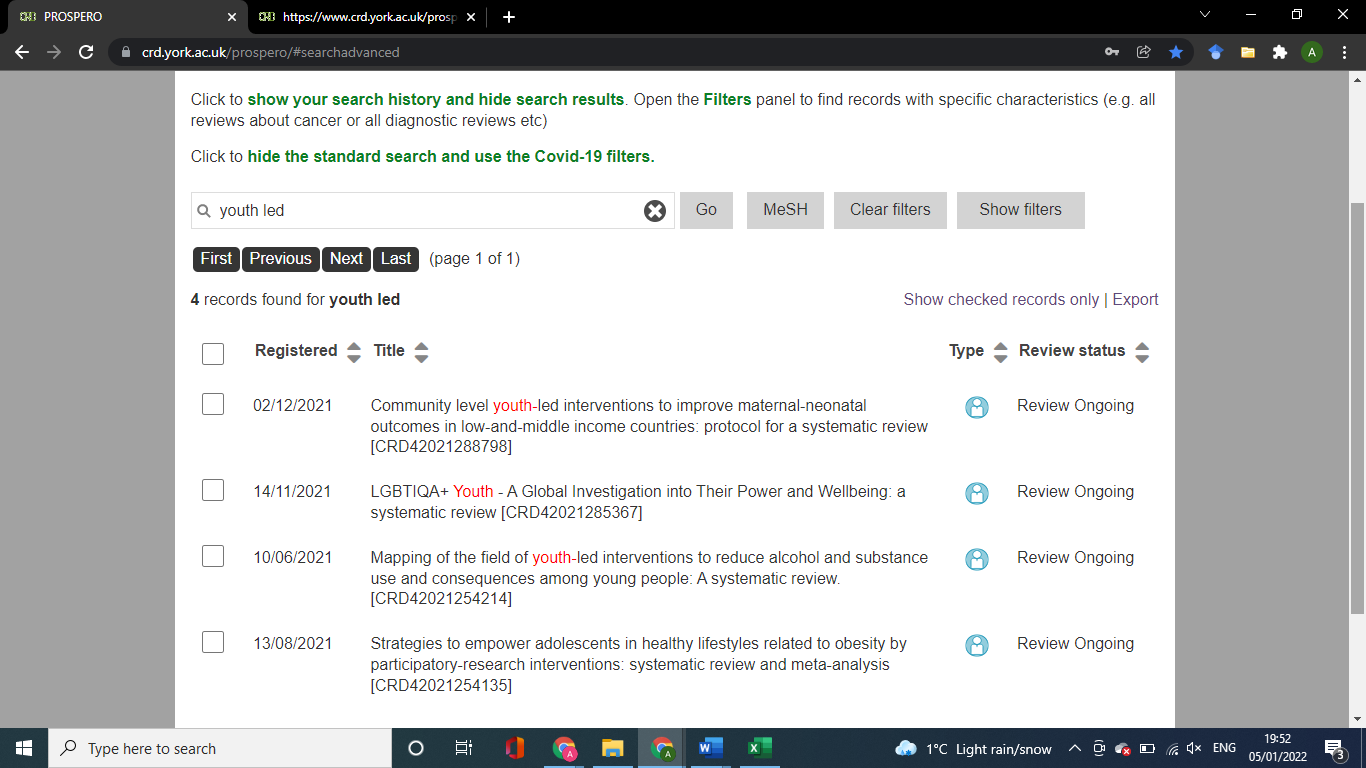 |
| MeSH DESCRIPTOR Community-Based Participatory Research EXPLODE ALL TREES  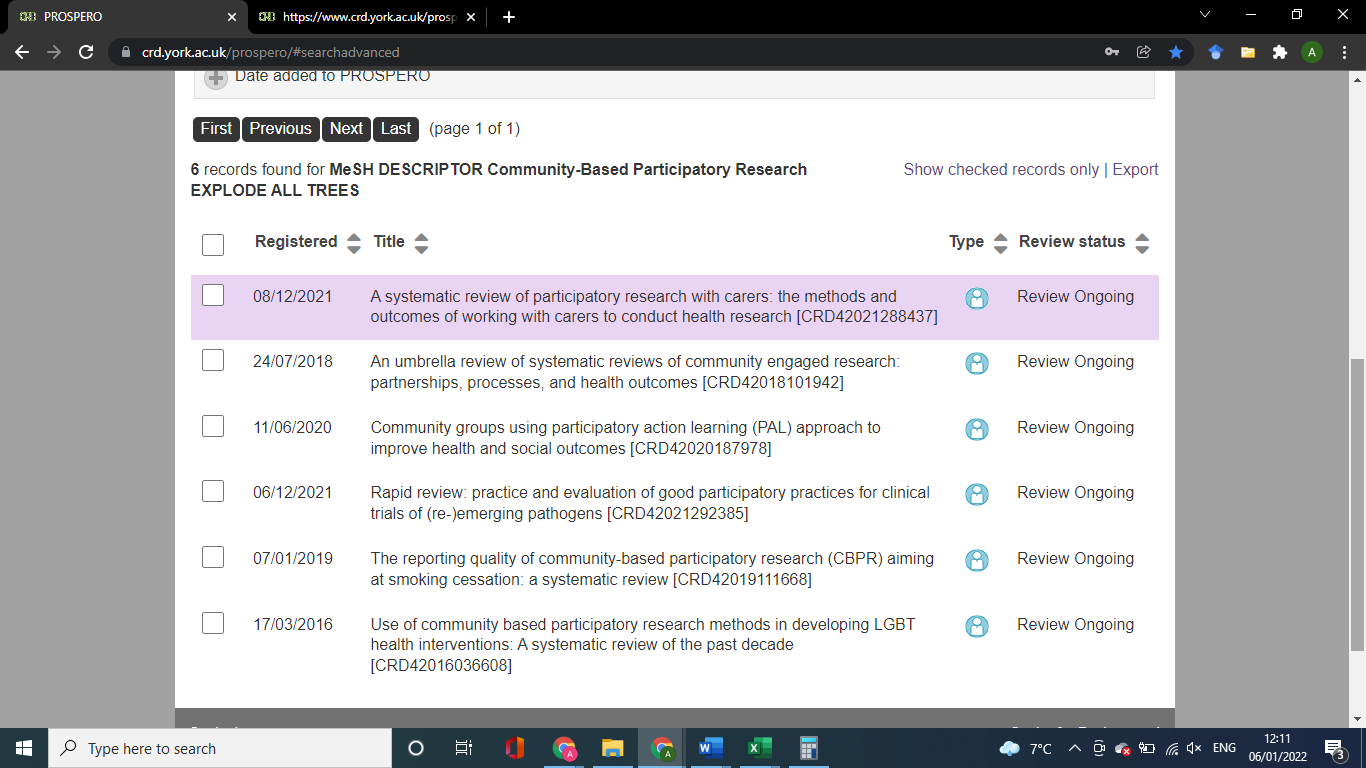 |
| public and patient involvement=3 (Filter=search in title)  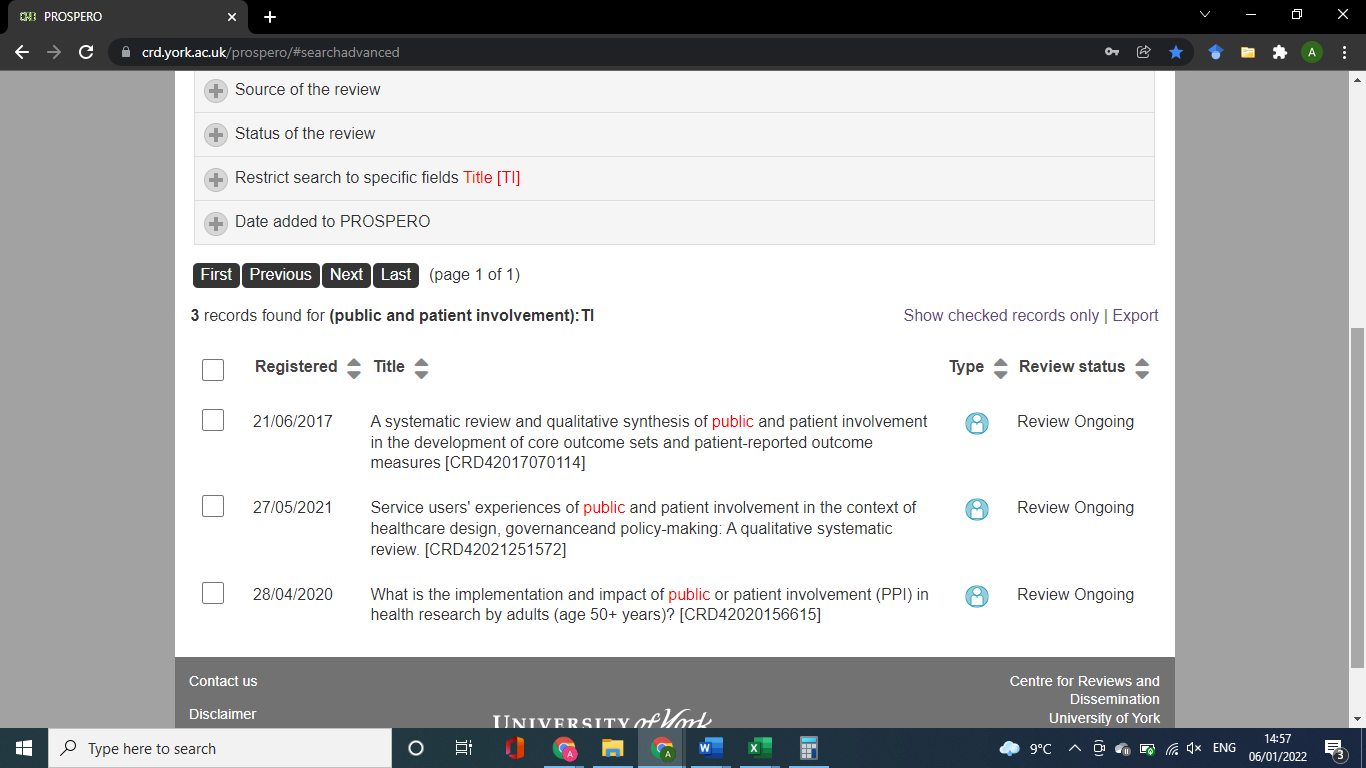 |
| co-production OR human-centered design=9 (Filter=search in title)  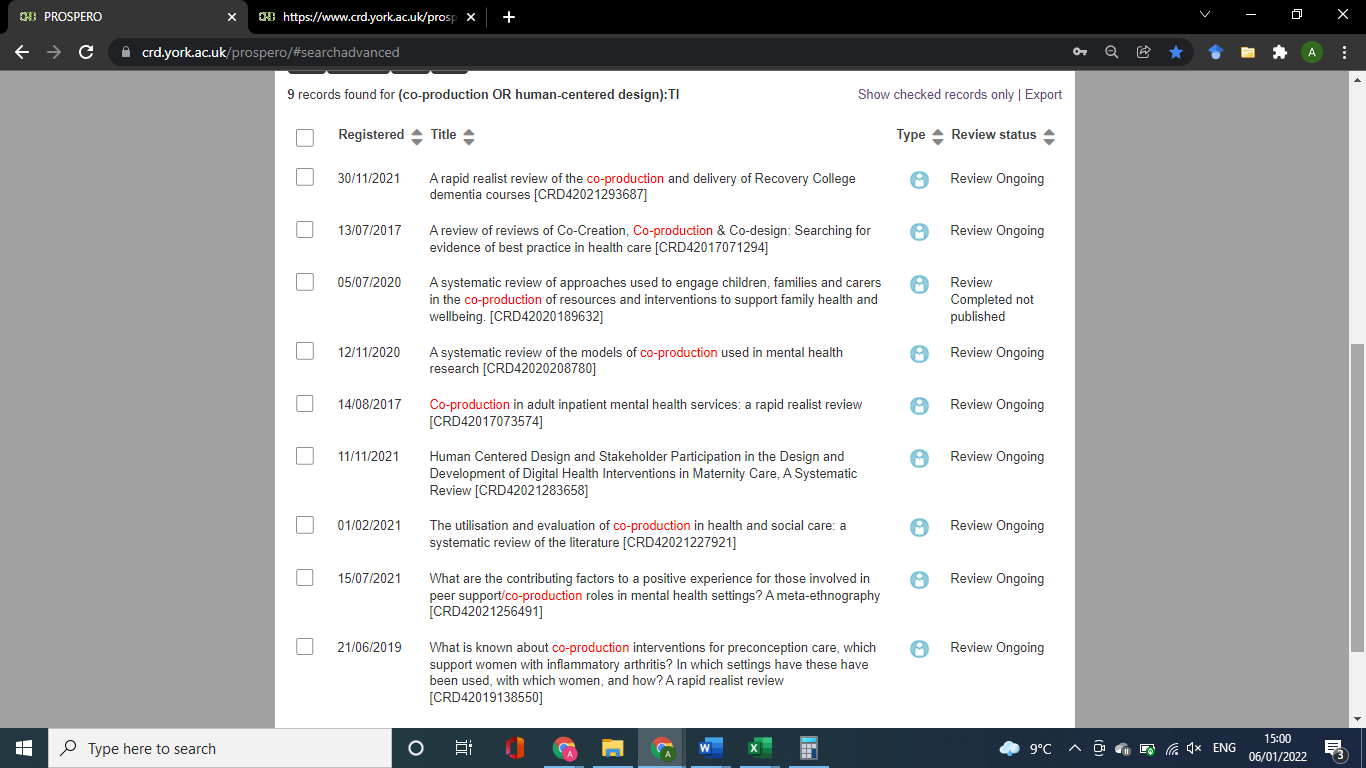 |
| peer researcher OR young researcher OR co-researcher=0 (Filter=search in title)  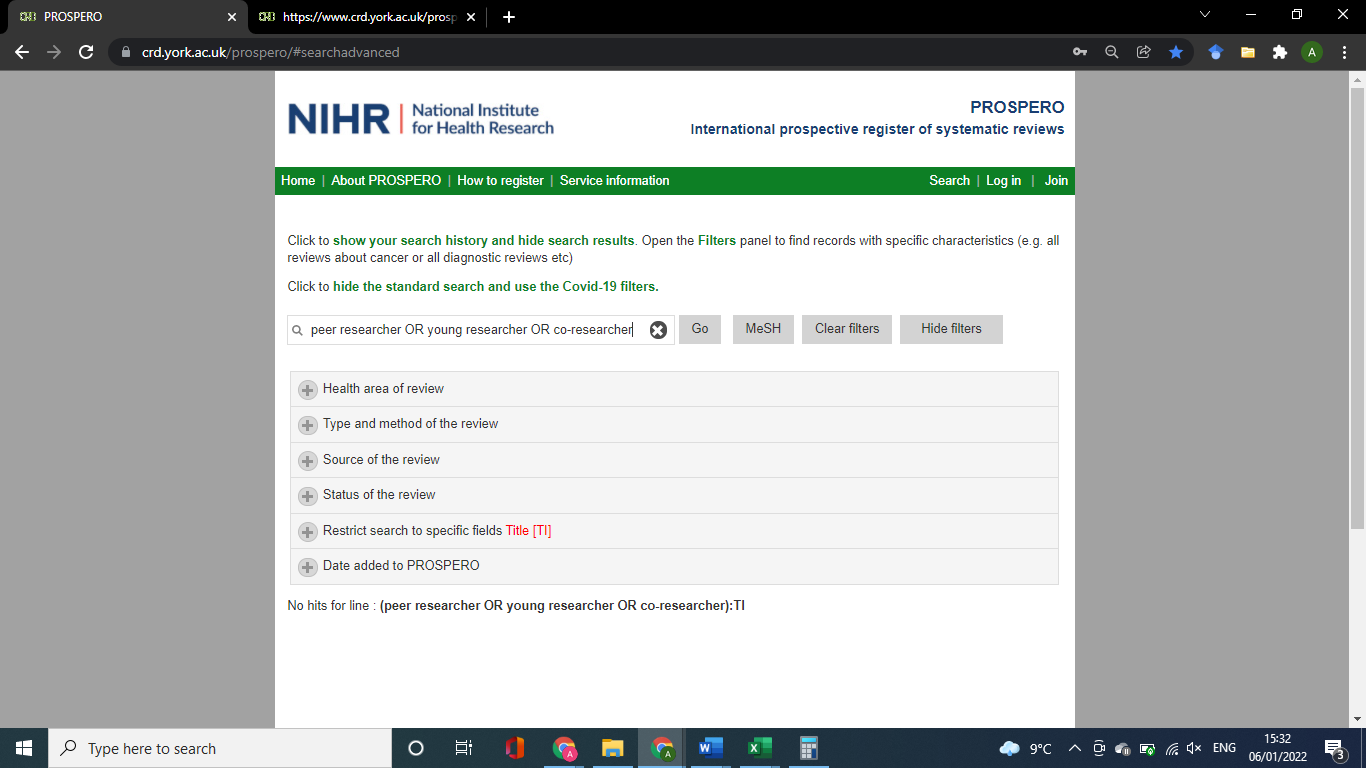 |
| Stakeholder OR participatory OR advisory=88 (Filter=search in title)  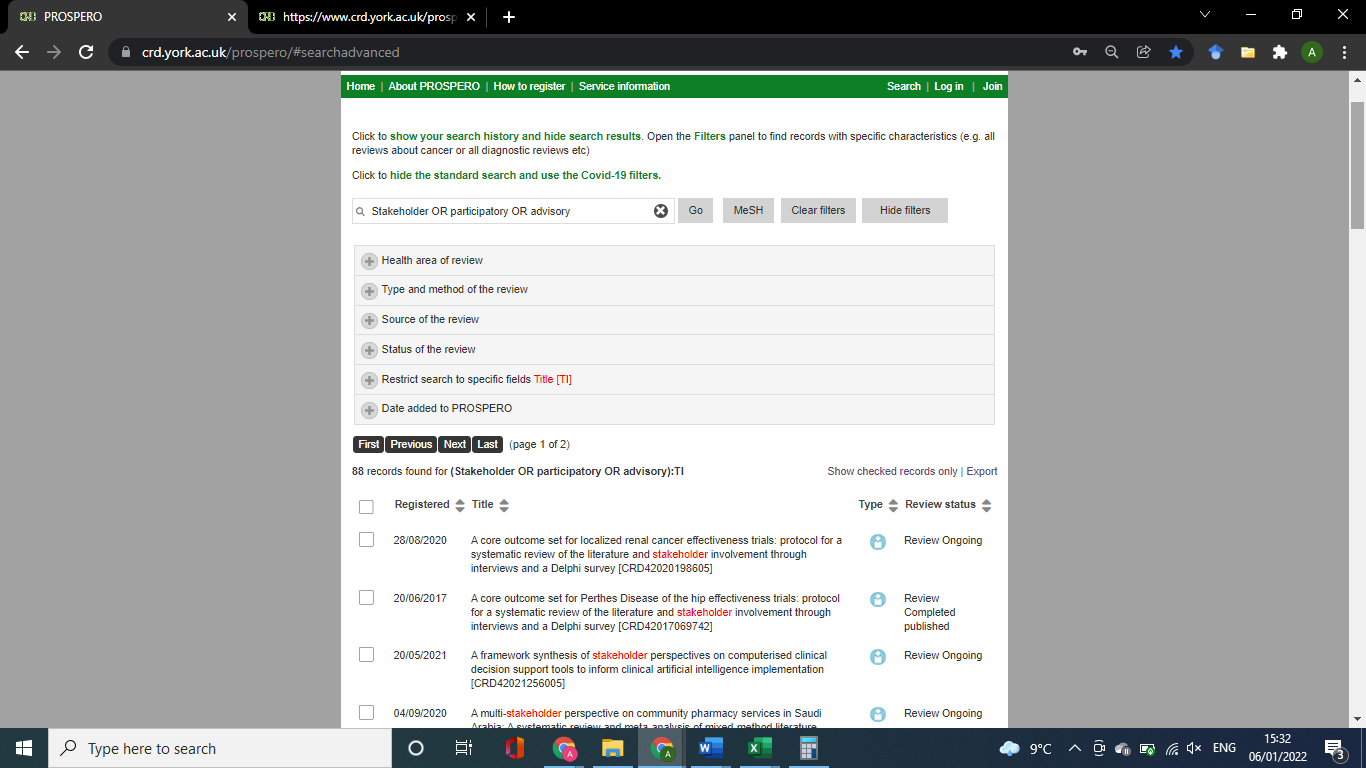 |
| 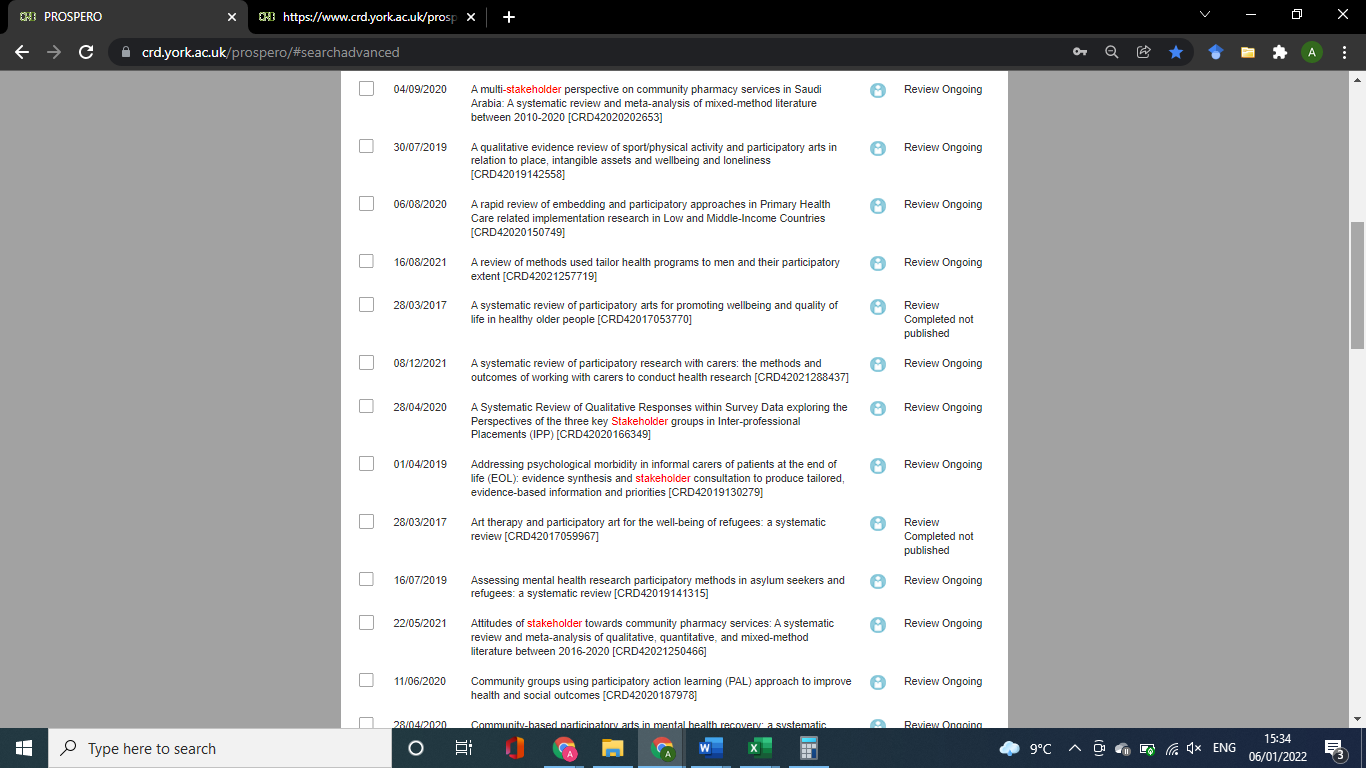 |
| 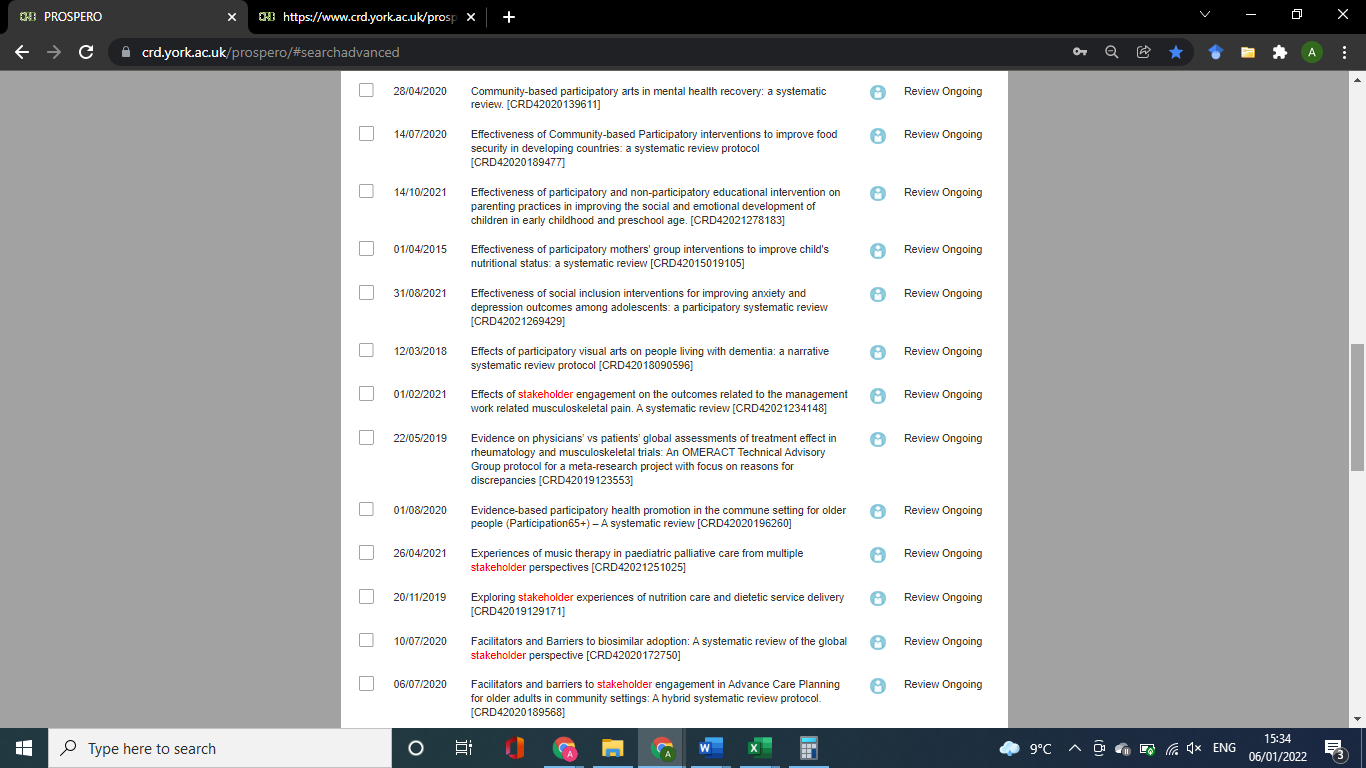 |
| 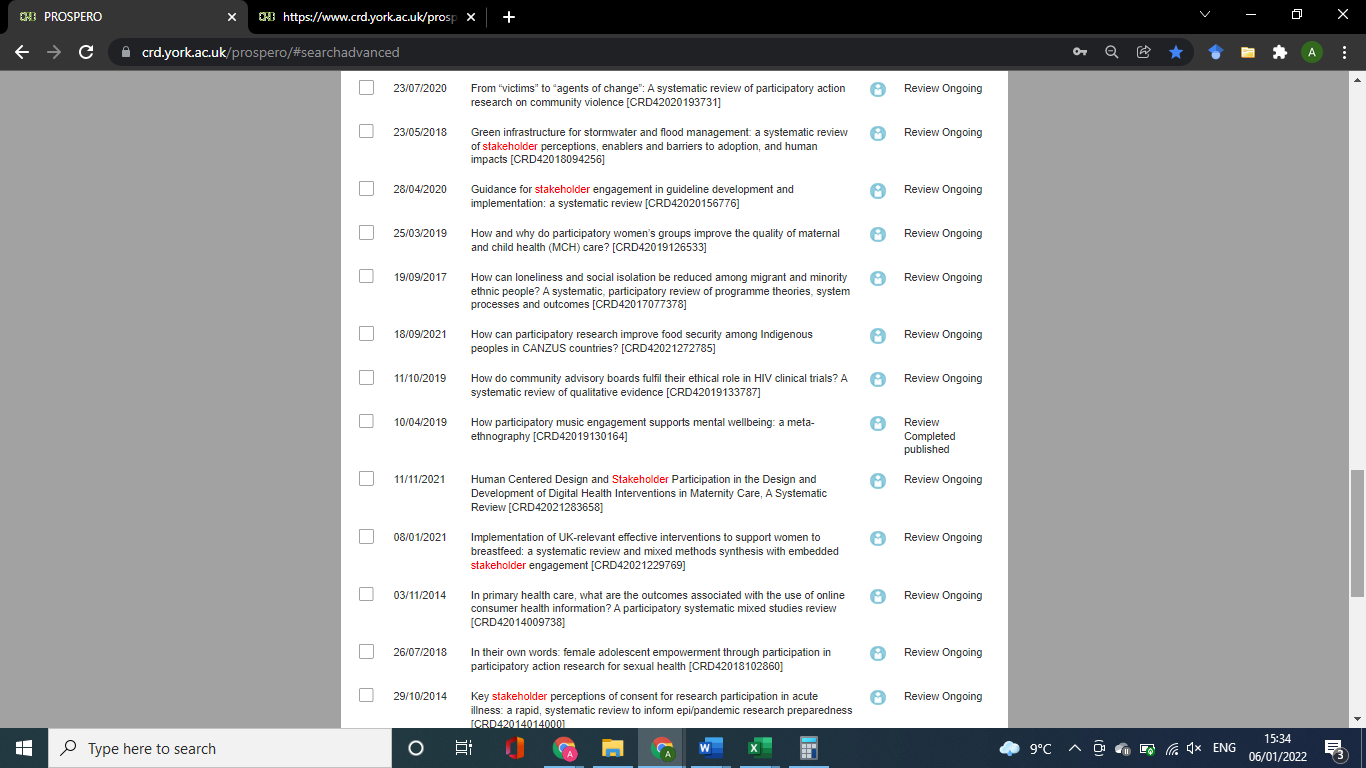 |
| 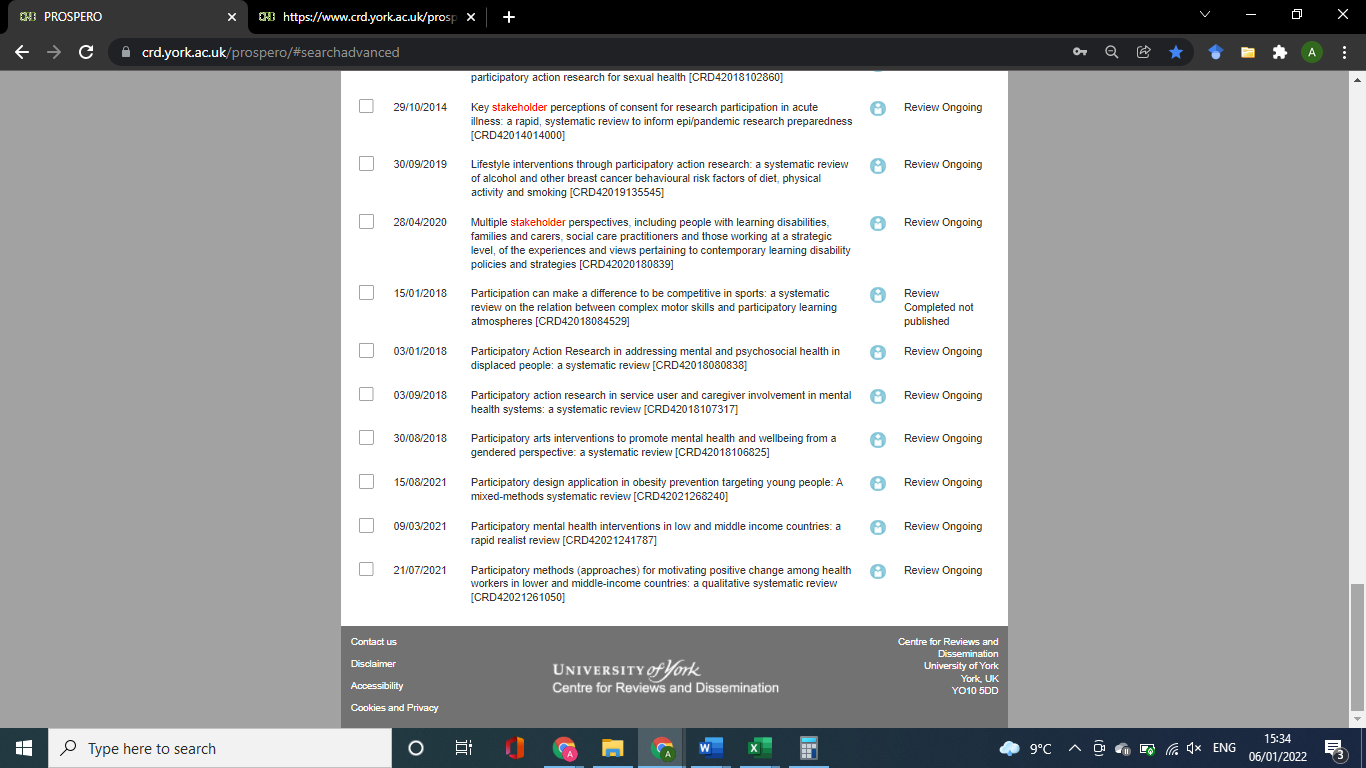 |
| 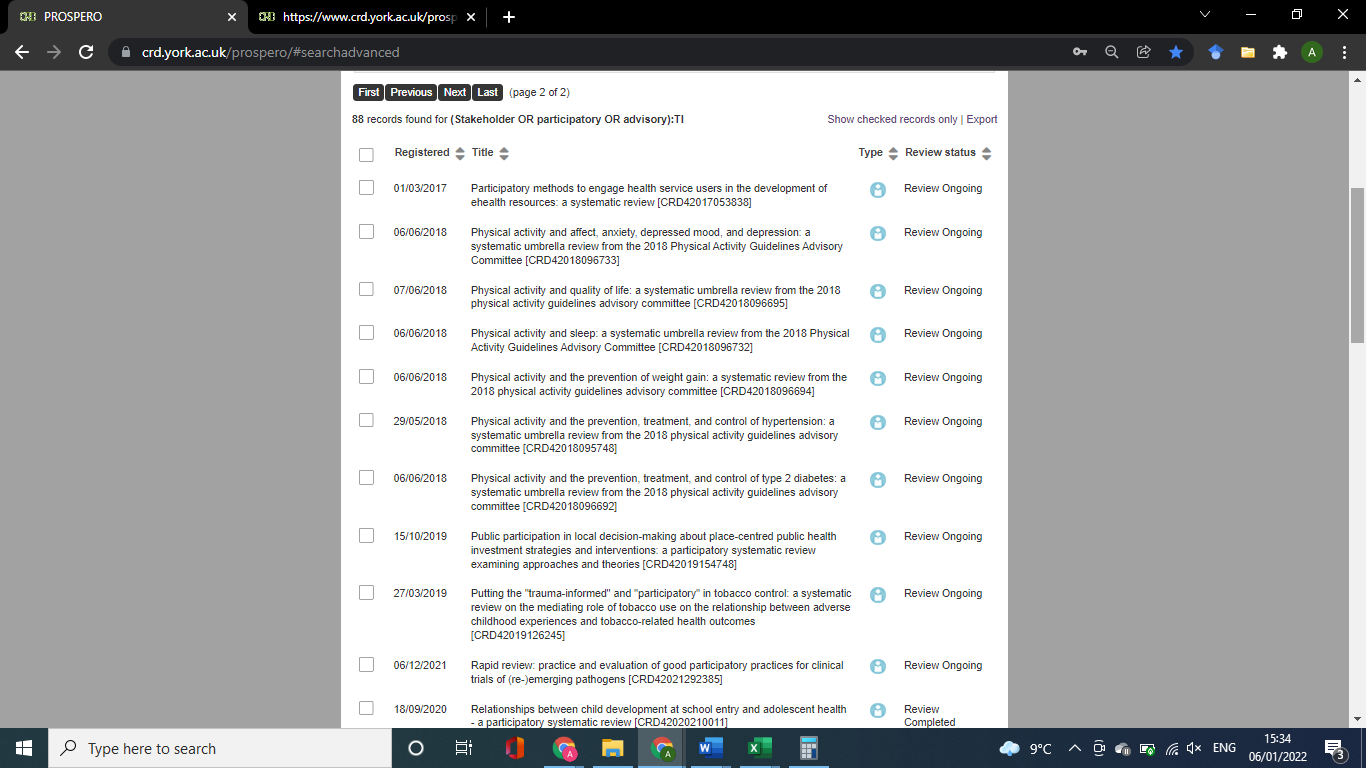 |
| 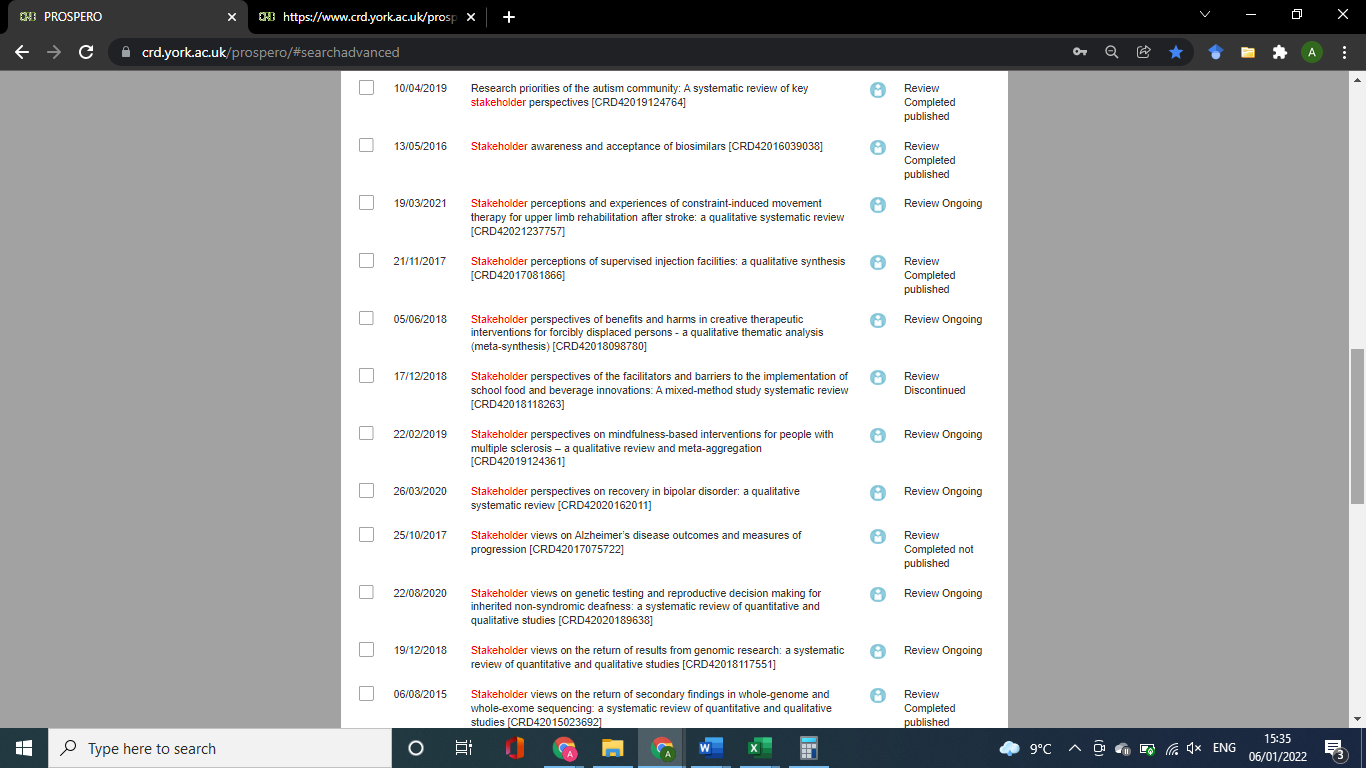 |
| 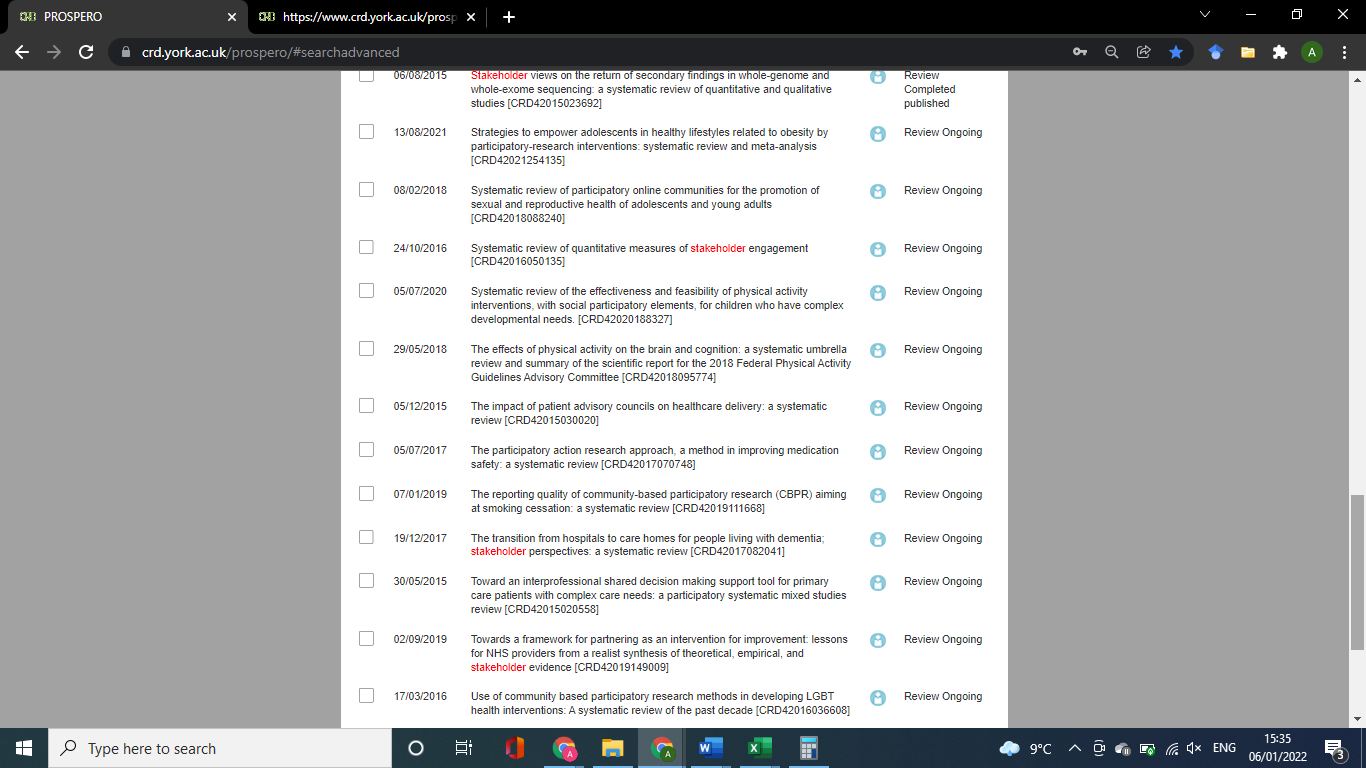 |
| 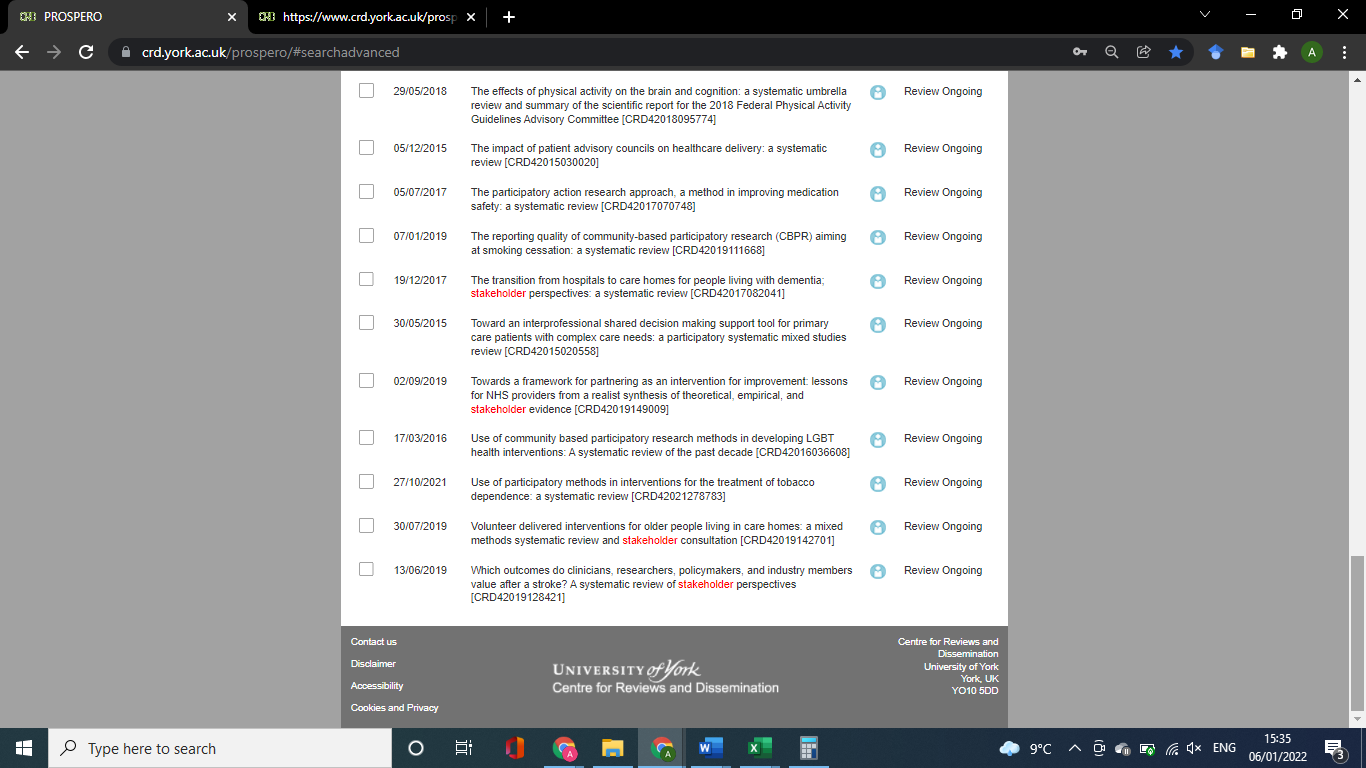 |
