# Supplementary material for: An umbrella review of reviews on challenges to meaningful adolescent involvement in health research
Source: Health Expect. 2024 Jan 27;27(1):e13980. doi: 10.1111/hex.13980 (PMC10821743; doi:10.1111/hex.13980)
Supplement: Supplementary file 1 — Supporting information. [file HEX-27-e13980-s001.zip › Search record and results/Other sources/Youth health researchers and youth engagement experts/Youth health researchers and youth engagement experts contact record.docx]

**Youth health researchers and youth engagement experts contact record**

| Number of experts contacted | 11 |
| --- | --- |
| Number of experts responded | 8 |
| Number of reviews shared | 6 |
| Eligible reviews | 4 |

**List of reviews shared**

| **Reviews** | **Eligibility** |
| --- | --- |
| Reed, H., Couturiaux, D., Davis, M., Edwards, A., Janes, E., Kim, H. S., ... & Evans, R. (2021). Co-production as an Emerging Methodology for Developing School-Based Health Interventions with Students Aged 11–16: Systematic Review of Intervention Types, Theories and Processes and Thematic Synthesis of Stakeholders’ Experiences. *Prevention Science*, *22*(4), 475-491. | Yes |
| Bradbury-Jones, C., Isham, L. and Taylor, J. (2018) “The complexities and contradictions in participatory research with vulnerable CYP: A qualitative systematic review,” Social Science and Medicine, 215(August), pp. 80–91. doi: 10.1016/j.socscimed.2018.08.038. | Yes |
| Brodie, I. et al. (2016) The participation of young people in child sexual exploitation services: a scoping review of the literature. Available at: https://www.alexiproject.org.uk/publications | No (Could not access full text data tables) |
| Walsham, M. (2020) Involving Young Londoners: A review of participatory approaches in the youth sector. Available at: https://www.trustforlondon.org.uk/publications/involving-young-londoners/ | Yes |
| Jorgensen, C.R. (2019) Children’s involvement in research – A review and comparison with service user involvement in health and social care. Social Sciences, 8(5), 149. Available from: https://doi. org/10.3390/socsci8050149. | Yes |
| Cowan, H., Kühlbrandt, C., & Riazuddin, H. (2022). Reordering the machinery of participation with young people. *Sociology of Health & Illness*. | No (Not youth involvement) |
