# Supplementary material for: An umbrella review of reviews on challenges to meaningful adolescent involvement in health research
Source: Health Expect. 2024 Jan 27;27(1):e13980. doi: 10.1111/hex.13980 (PMC10821743; doi:10.1111/hex.13980)
Supplement: Supplementary file 1 — Supporting information. [file HEX-27-e13980-s001.zip › Search record and results/Other sources/Protocols/Protocol papers search record.docx]

**Protocol papers search record**

Screened protocols included in the search results of academic databases

| **Total results** | **132** |
| --- | --- |
| **Duplicates** | 2 |
| **Ineligible** | 127 |
| **Eligible** | 3 |
| **Authors of eligible reviews contacted** | 3 |
| Authors didn’t respond | 1 |
| Outcome paper not published yet | 2 |
