# Supplementary material for: An umbrella review of reviews on challenges to meaningful adolescent involvement in health research
Source: Health Expect. 2024 Jan 27;27(1):e13980. doi: 10.1111/hex.13980 (PMC10821743; doi:10.1111/hex.13980)
Supplement: Supplementary file 1 — Supporting information. [file HEX-27-e13980-s001.zip › Search record and results/Other sources/10 Journals/1. JAMA/JAMA search strings and results.docx]

**Overview**

Journal 1: [JAMA pediatrics](https://www.scimagojr.com/journalsearch.php?q=15402&tip=sid&clean=0)

Date of search: 14^th^ January

Search terms/strings used to search the journal= 19

| Search terms/strings | Results |
| --- | --- |
| 1. “Youth involvement” | 24 |
| 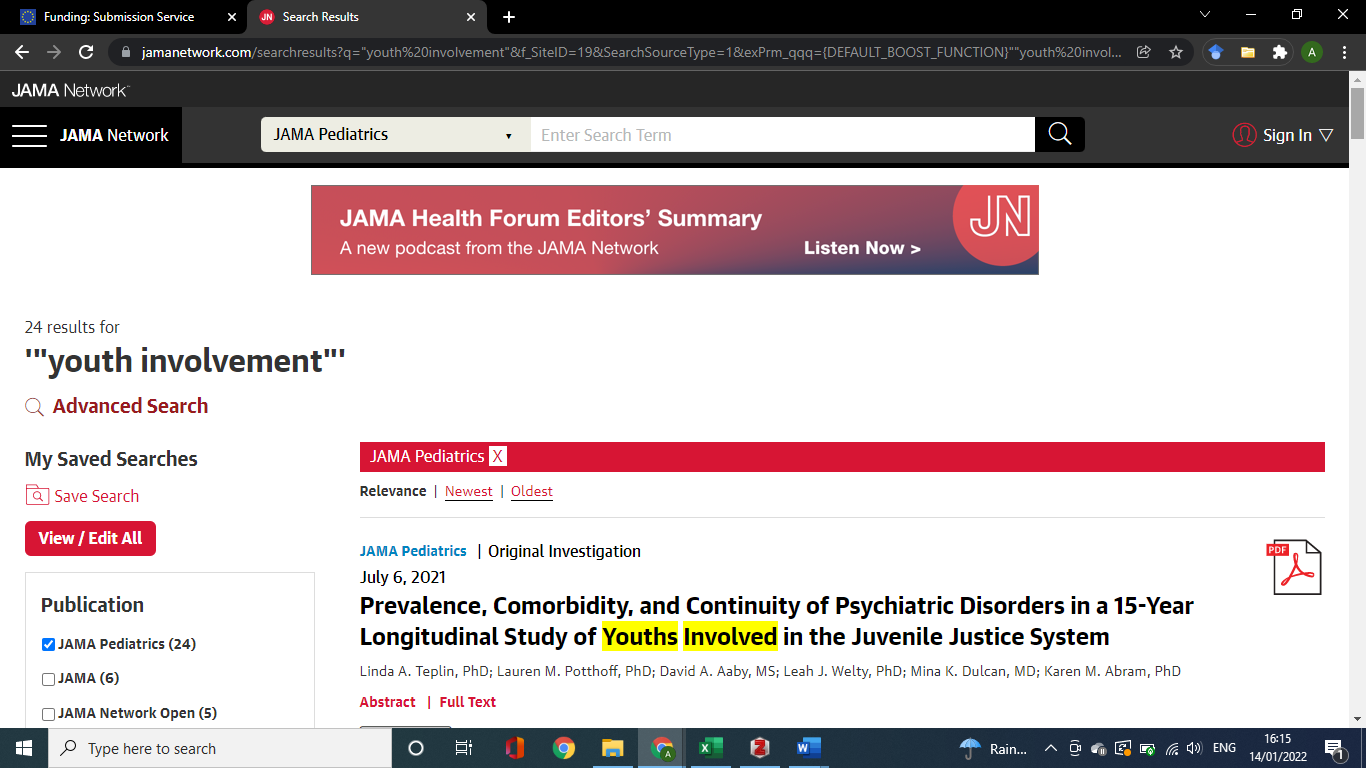 |  |
| 2. “Youth engagement” | 20 |
| 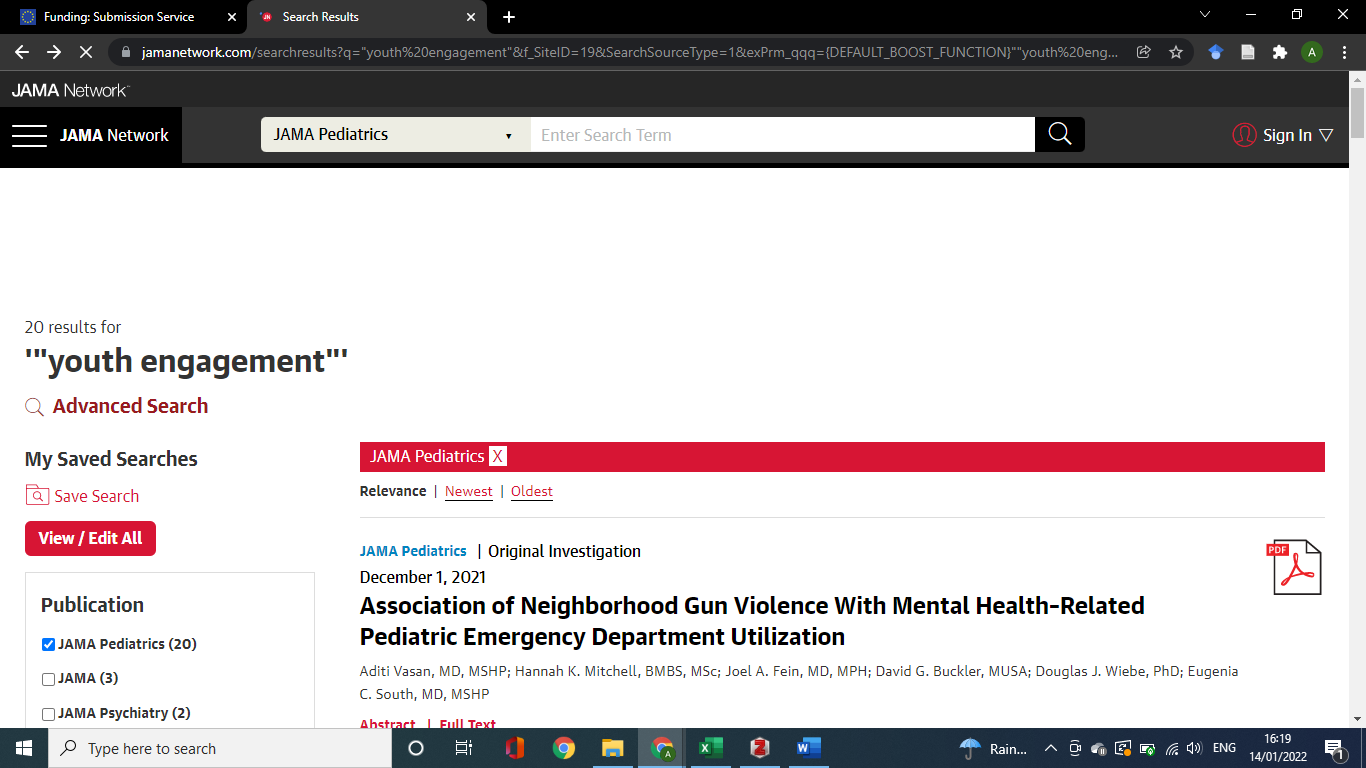 |  |
| 3. “adolescent involvement” | 2 |
| 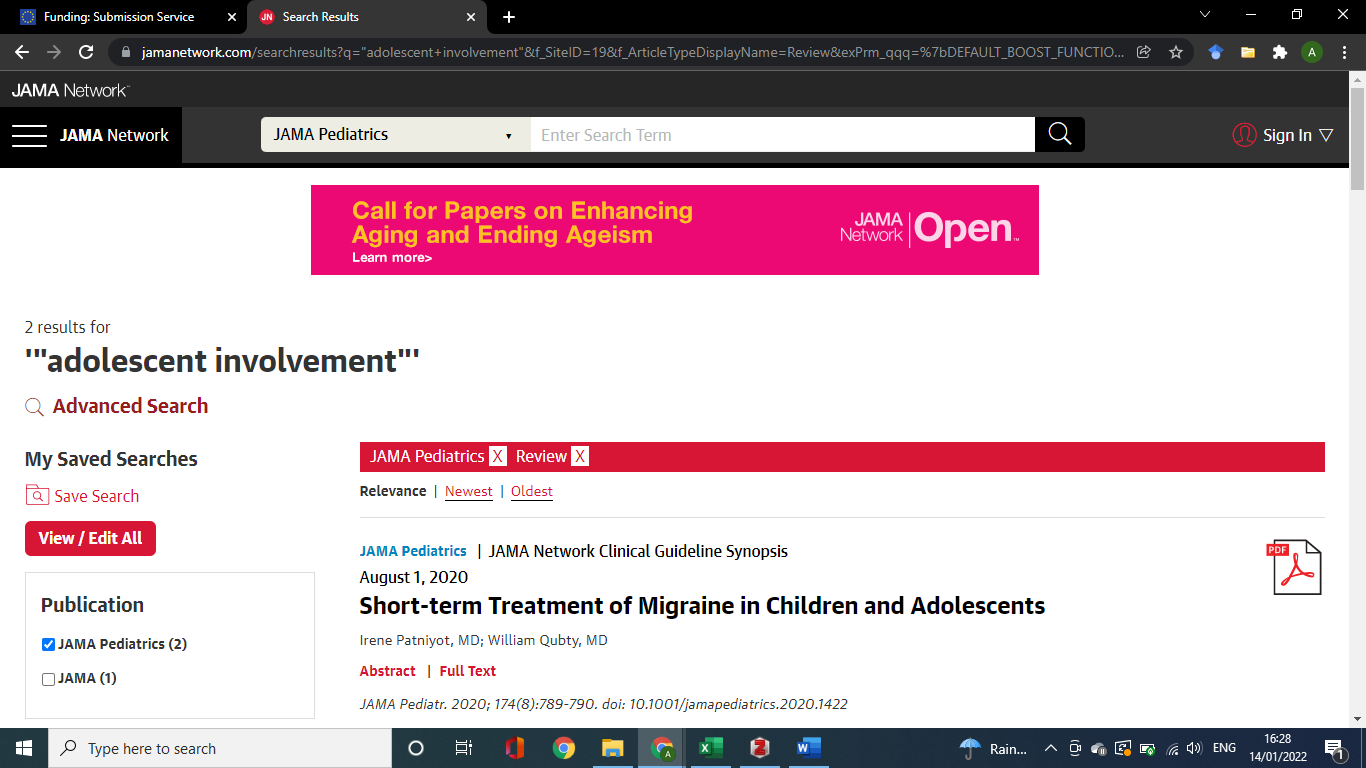 |  |
| 4. “adolescent engagement” | 33 |
| 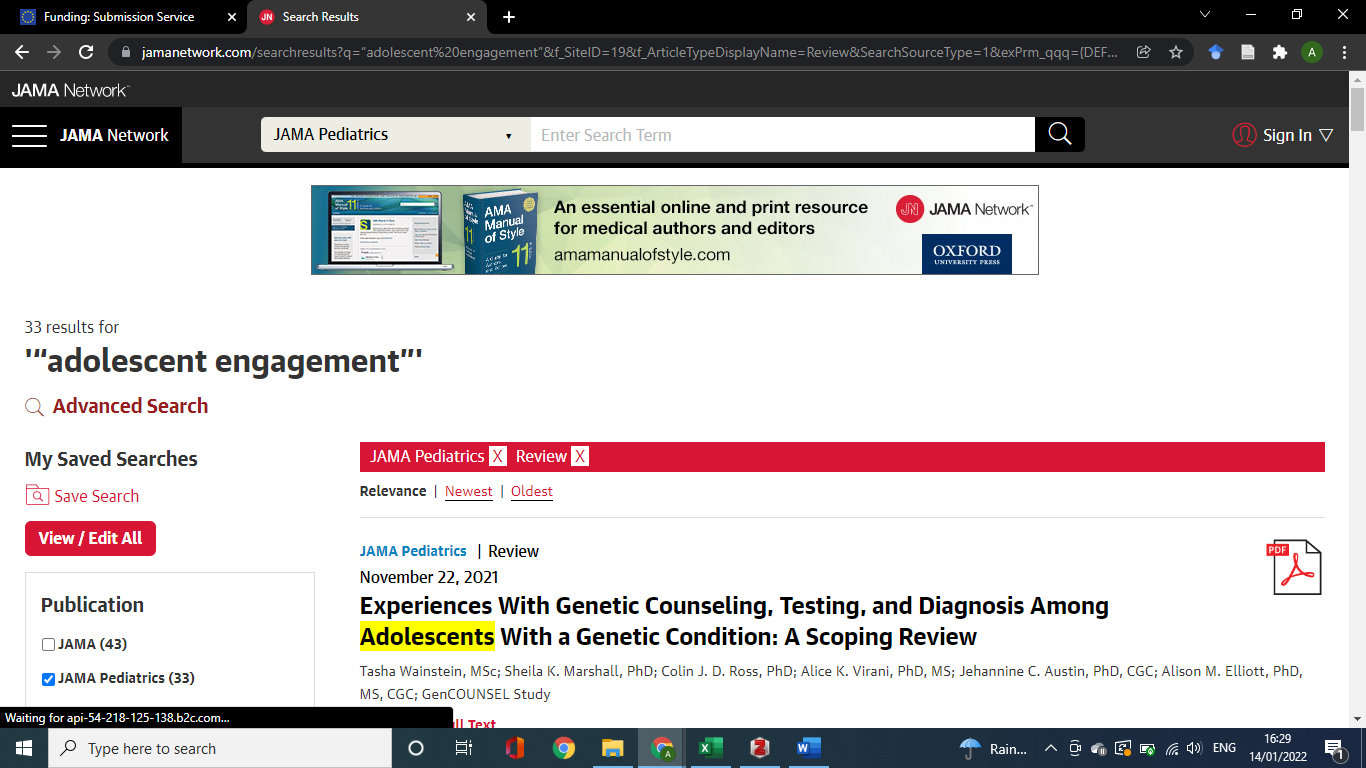 |  |
| 5. Stakeholder | 13 |
| 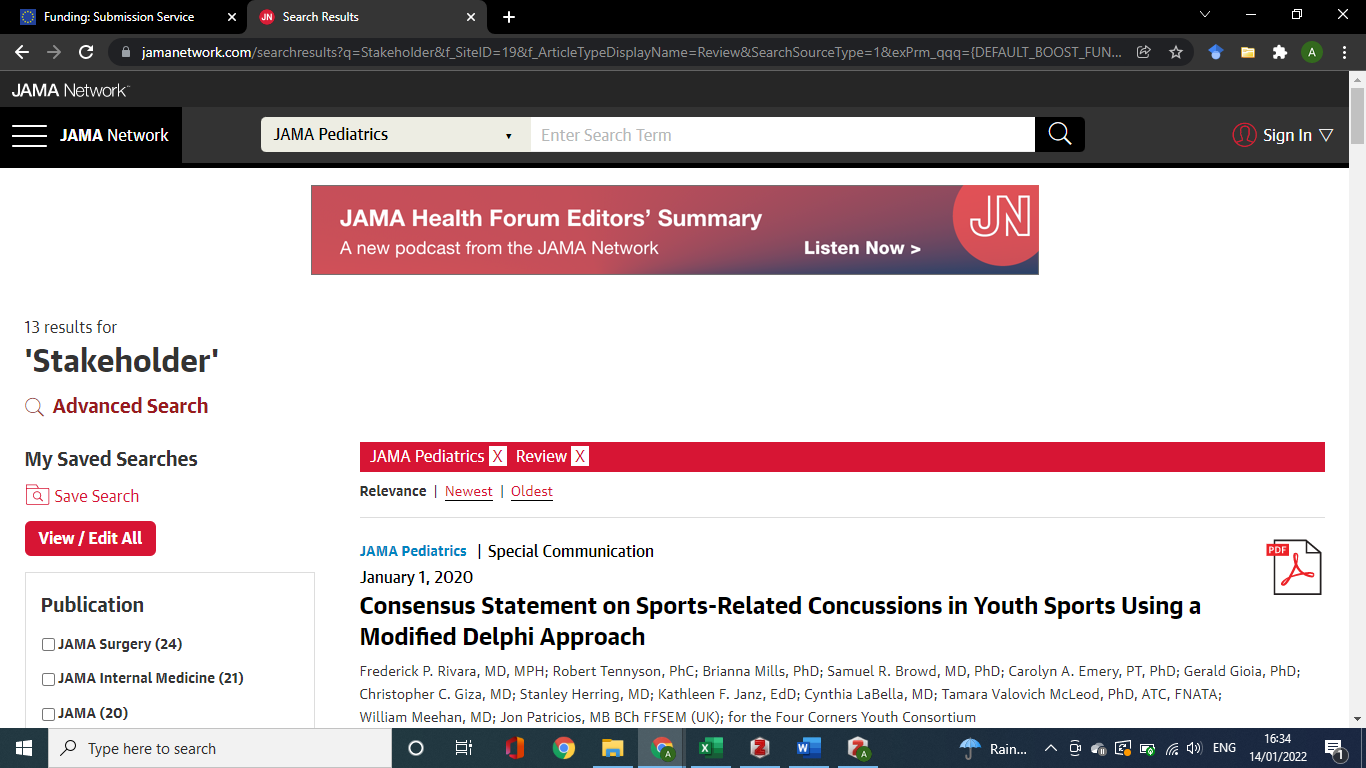 |  |
| 6. Participatory | 2 |
| 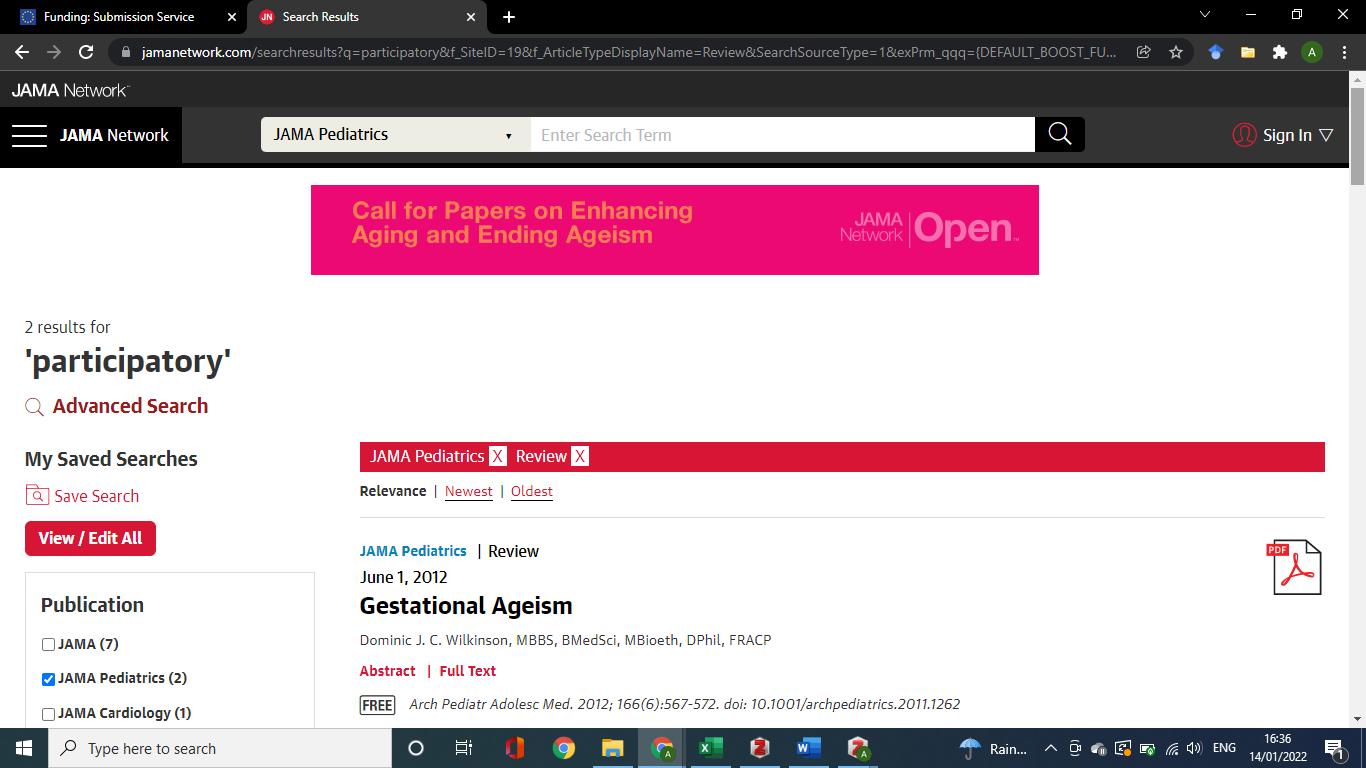 |  |
| 7. Advisory | 11 |
| 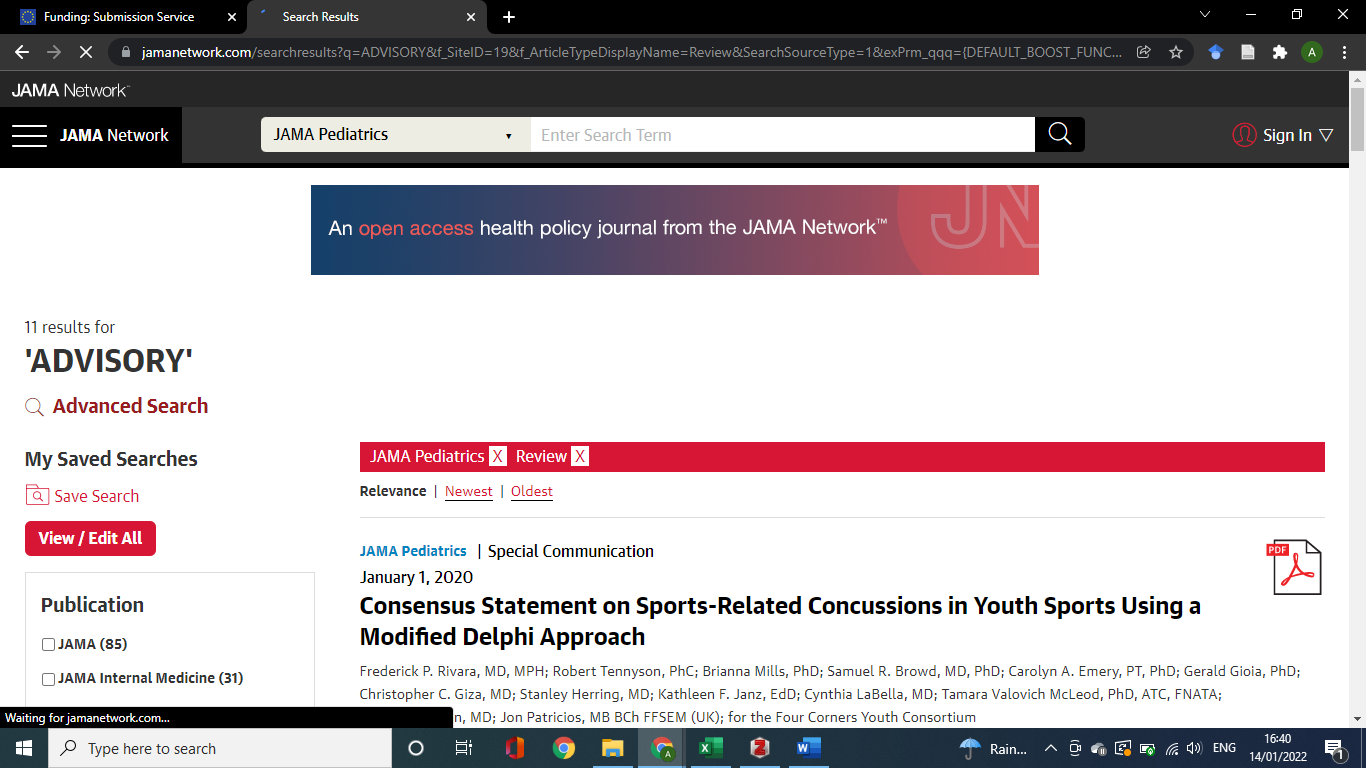 |  |
| 8. "patient and public involvement" | 0 |
| 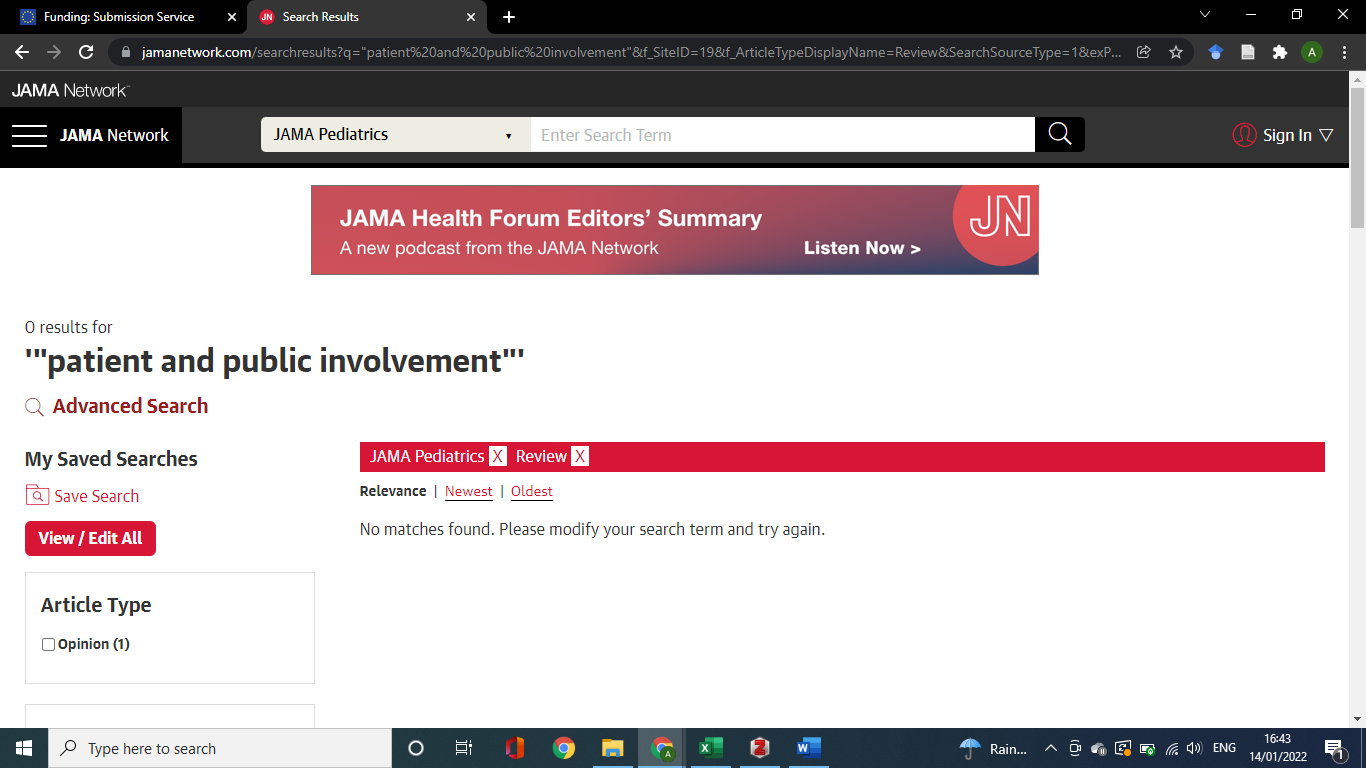 |  |
| 9. "public and patient involvement" | 0 |
| 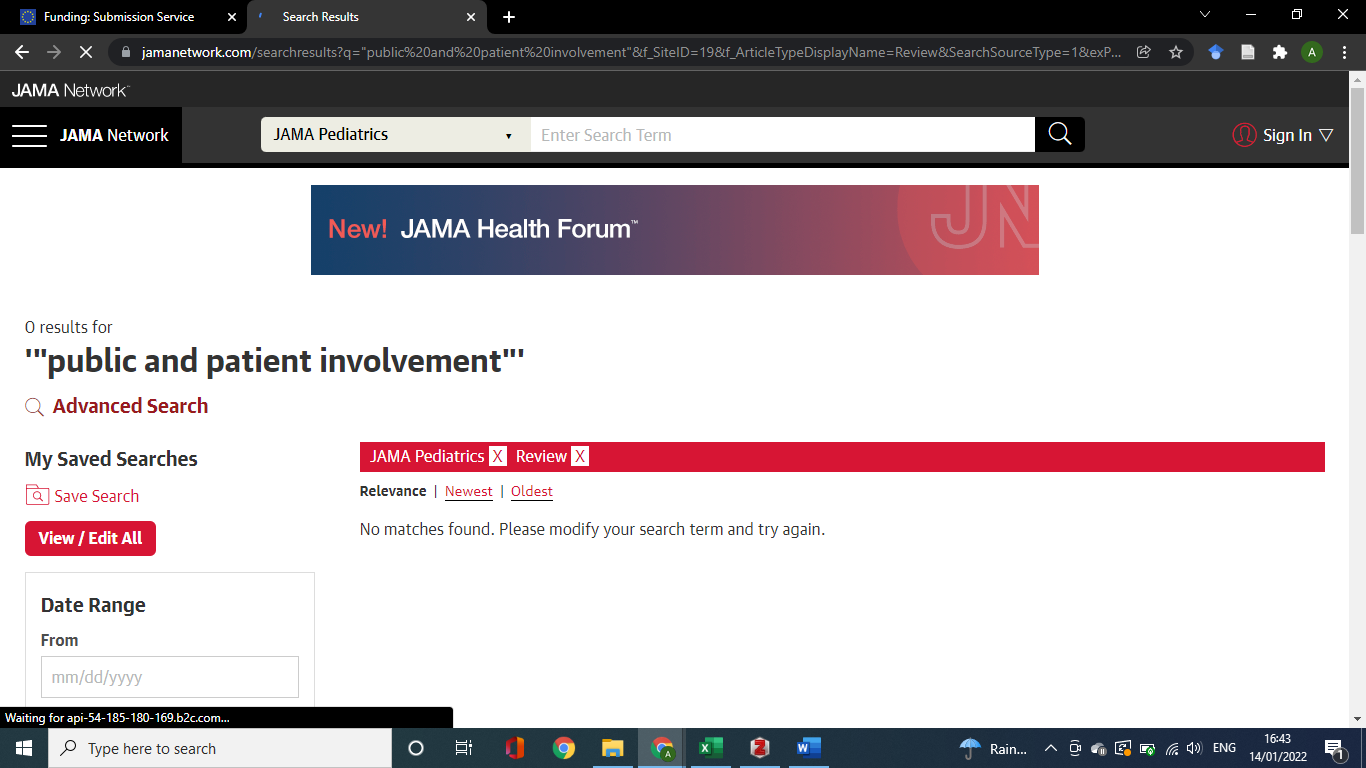 | |
| 10. "public patient involvement" | 0 |
| 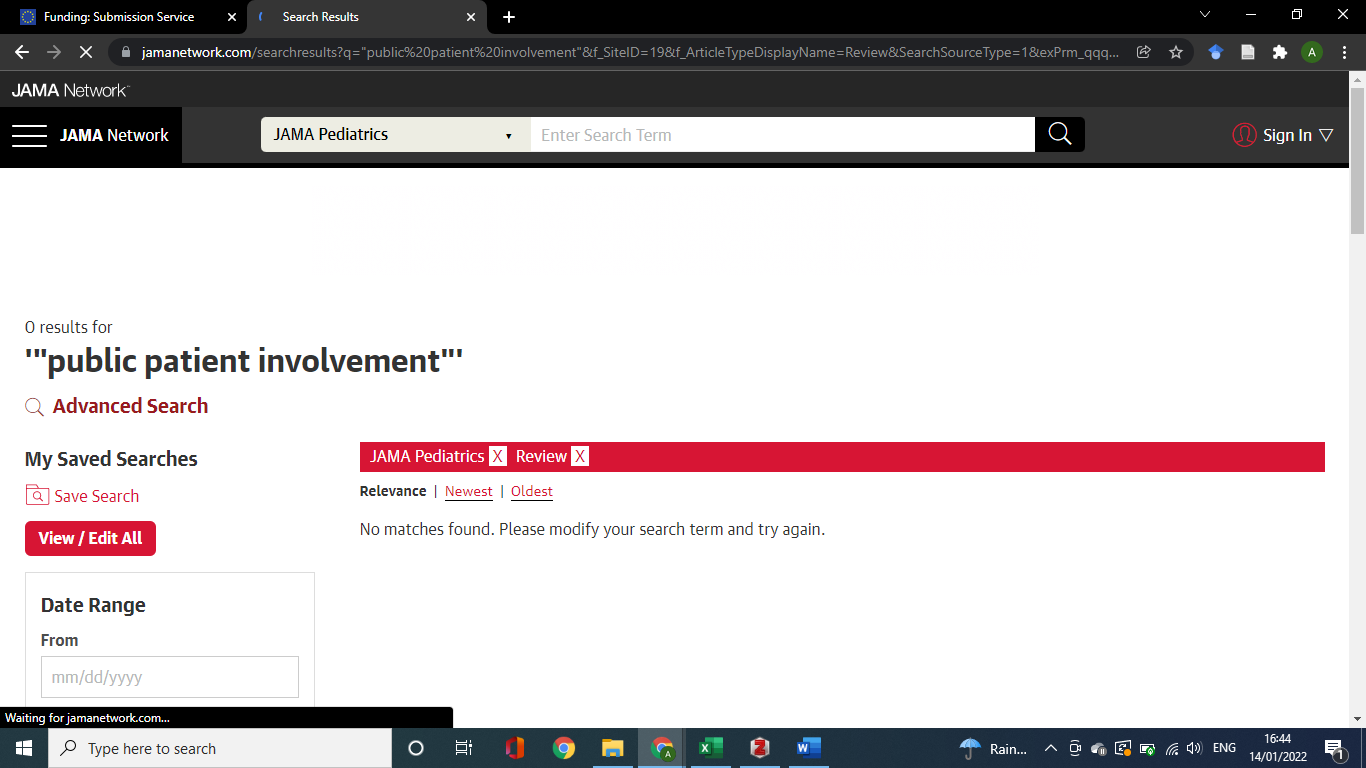 | |
| 11. "patient public involvement" | 0 |
| 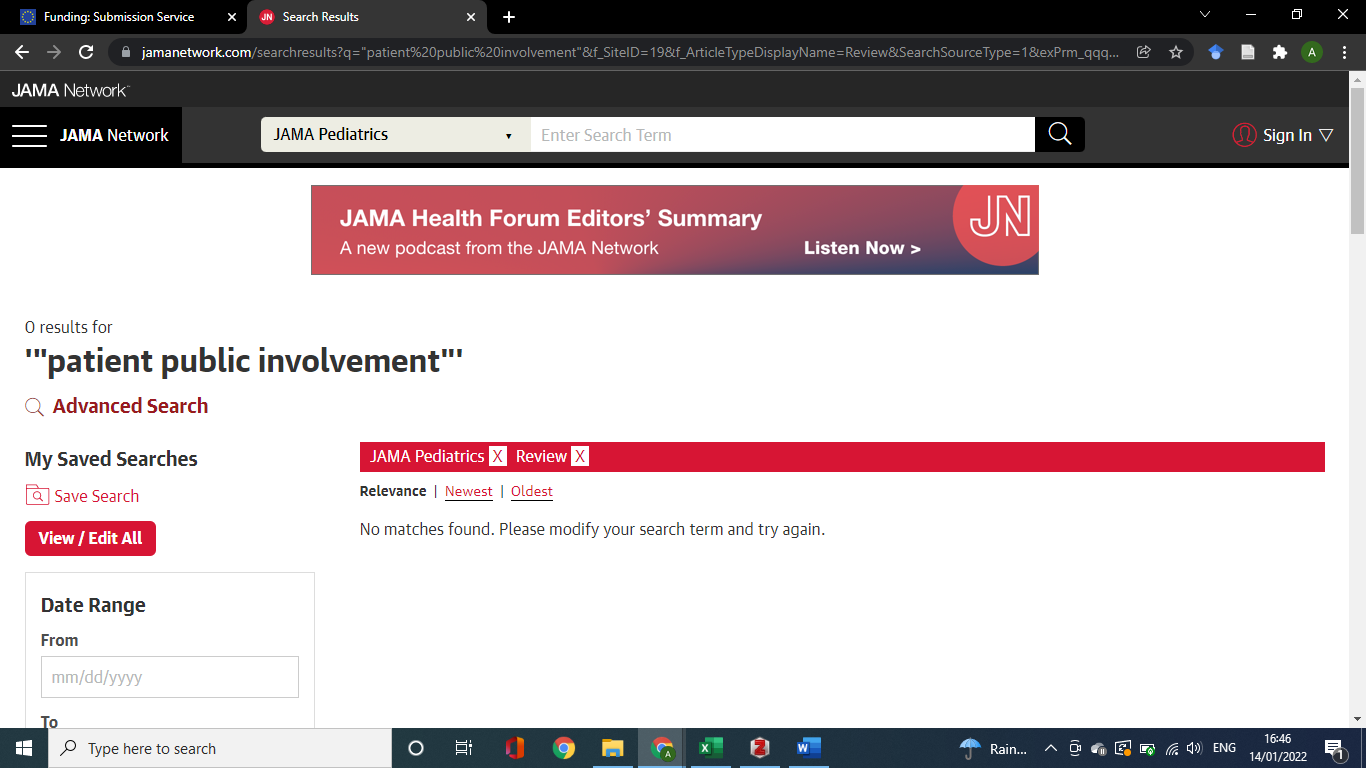 | |
| 12- "co production" | 0 |
| 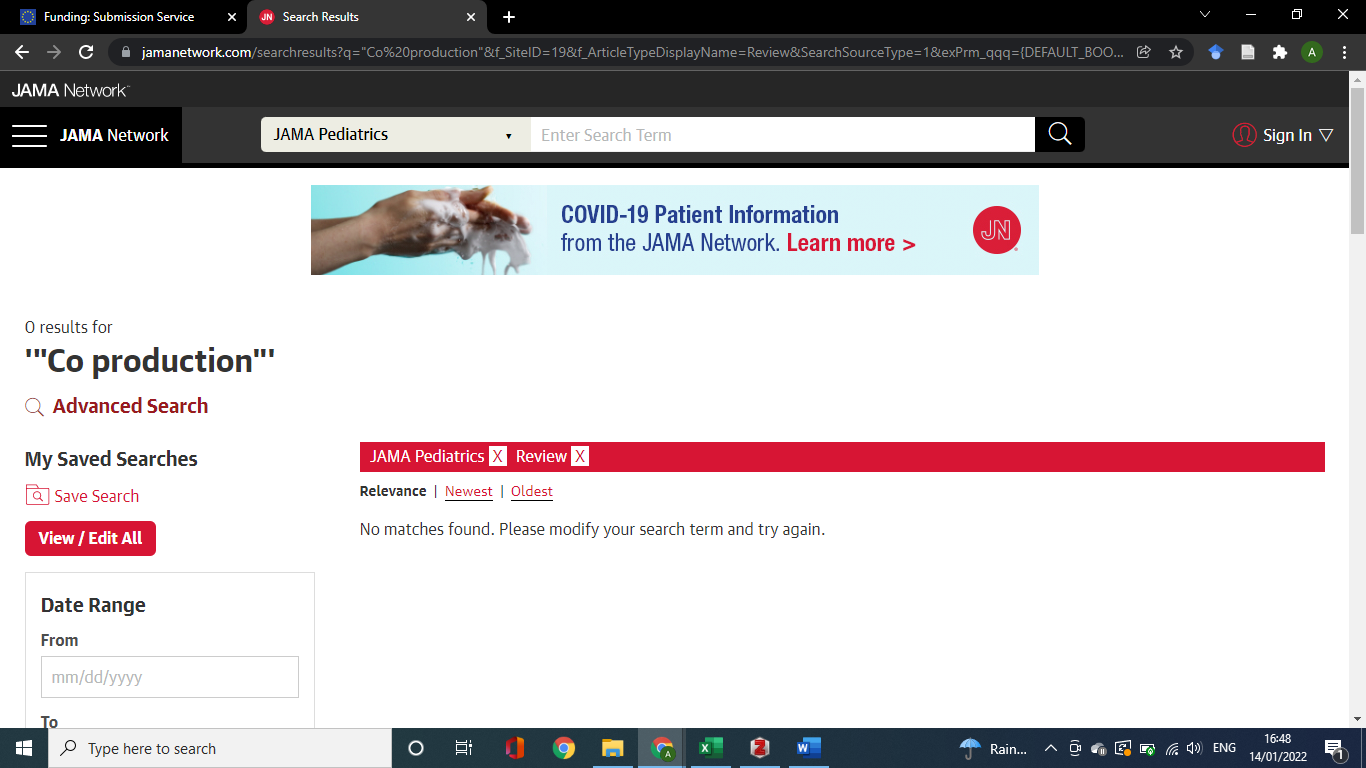 | |
| 13. "co design" | 15 |
| 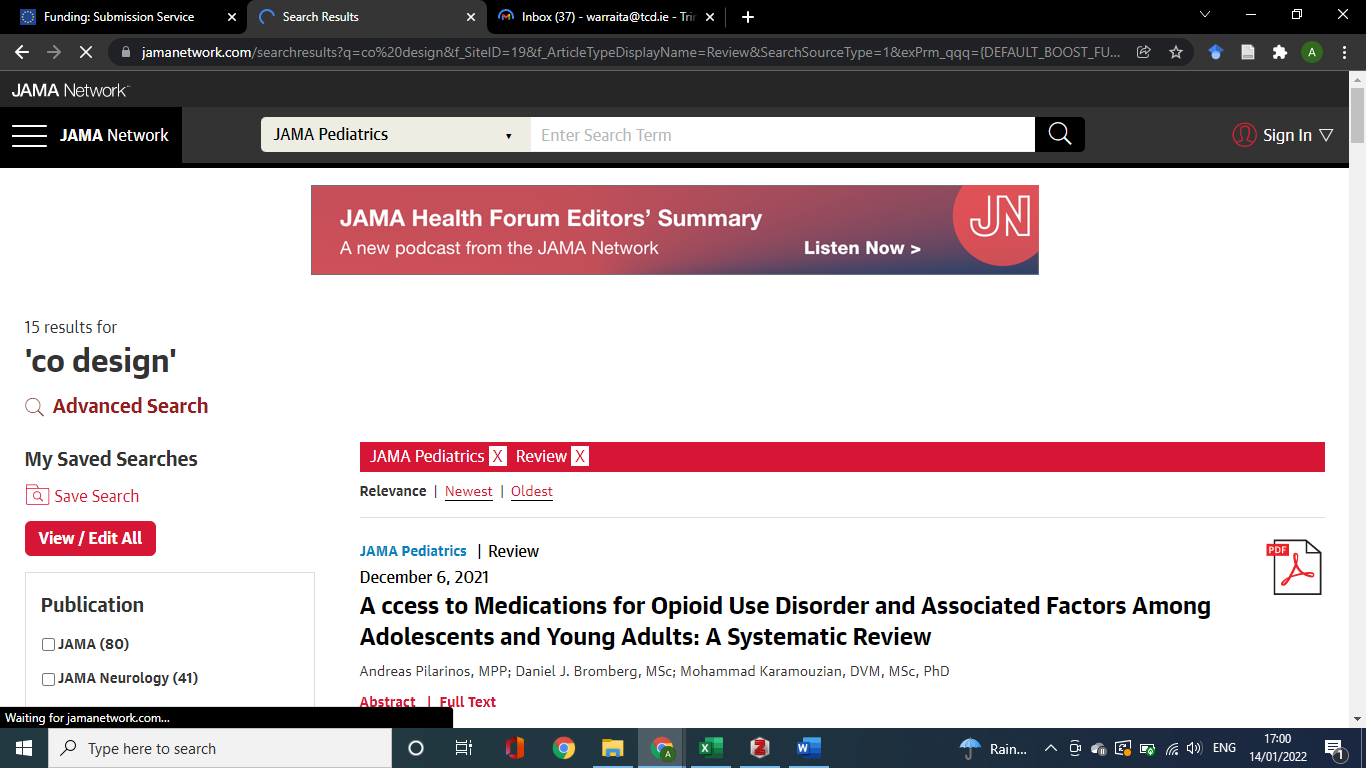 | |
| 14. "human centered design" / "human centred design" | 0 |
| 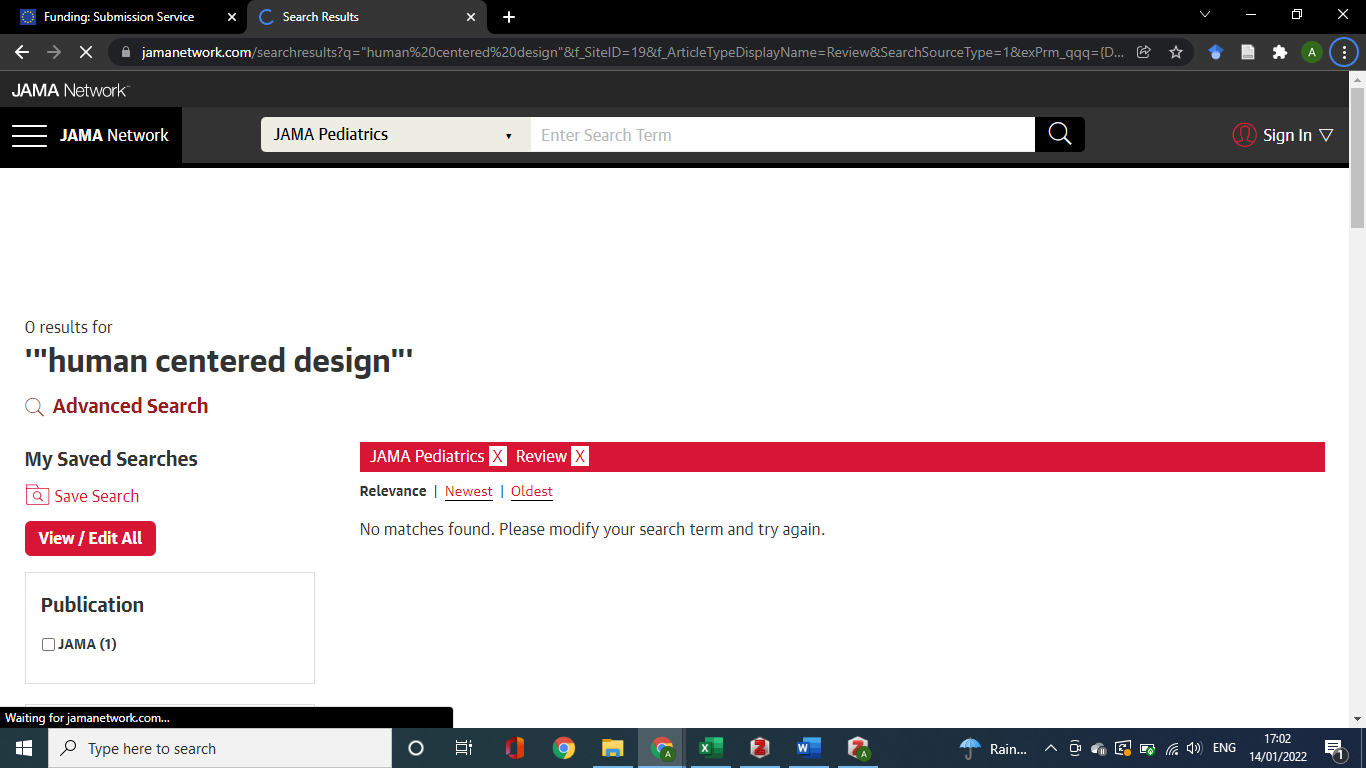 | |
| 15. "User centered design"/ "User centred design" | 0 |
| 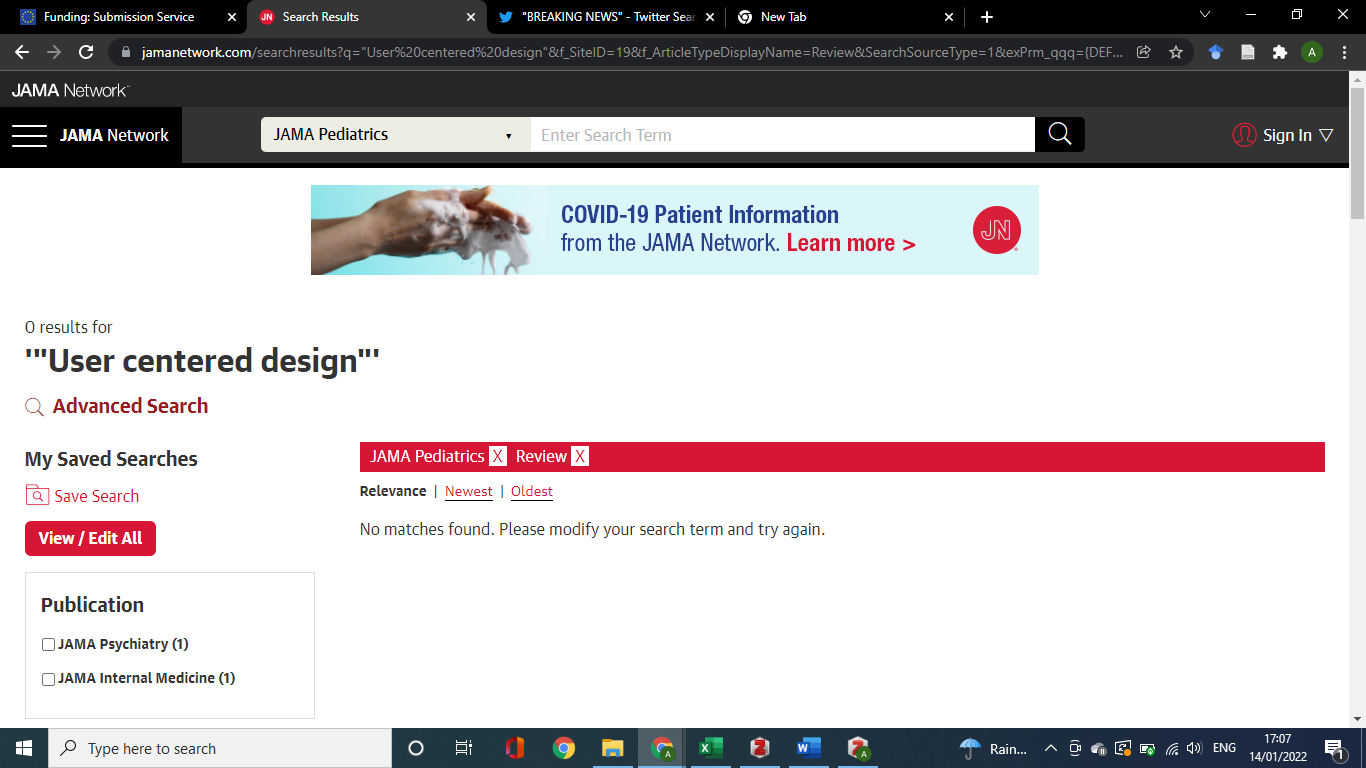 | |
| 16. "peer researcher” | 58 |
| 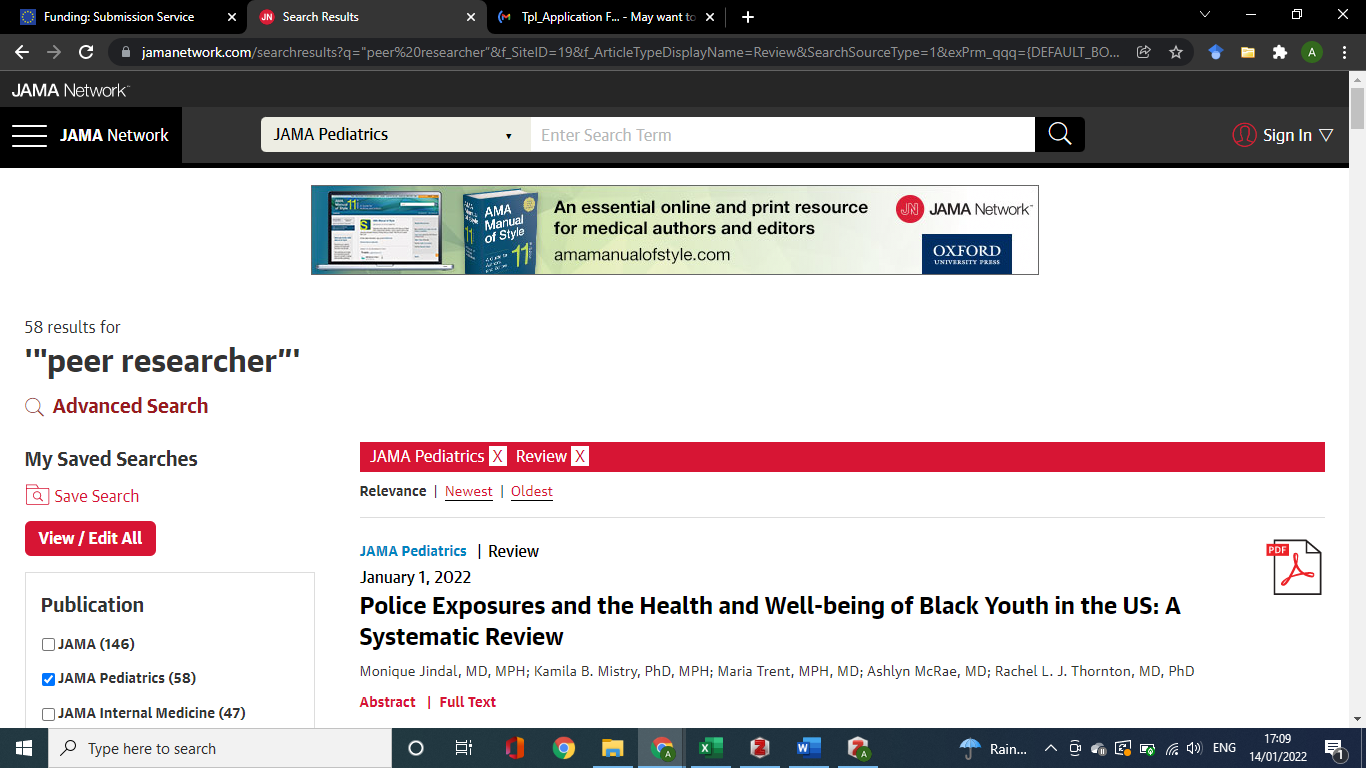 | |
| 17. "young researcher" | 0 |
| 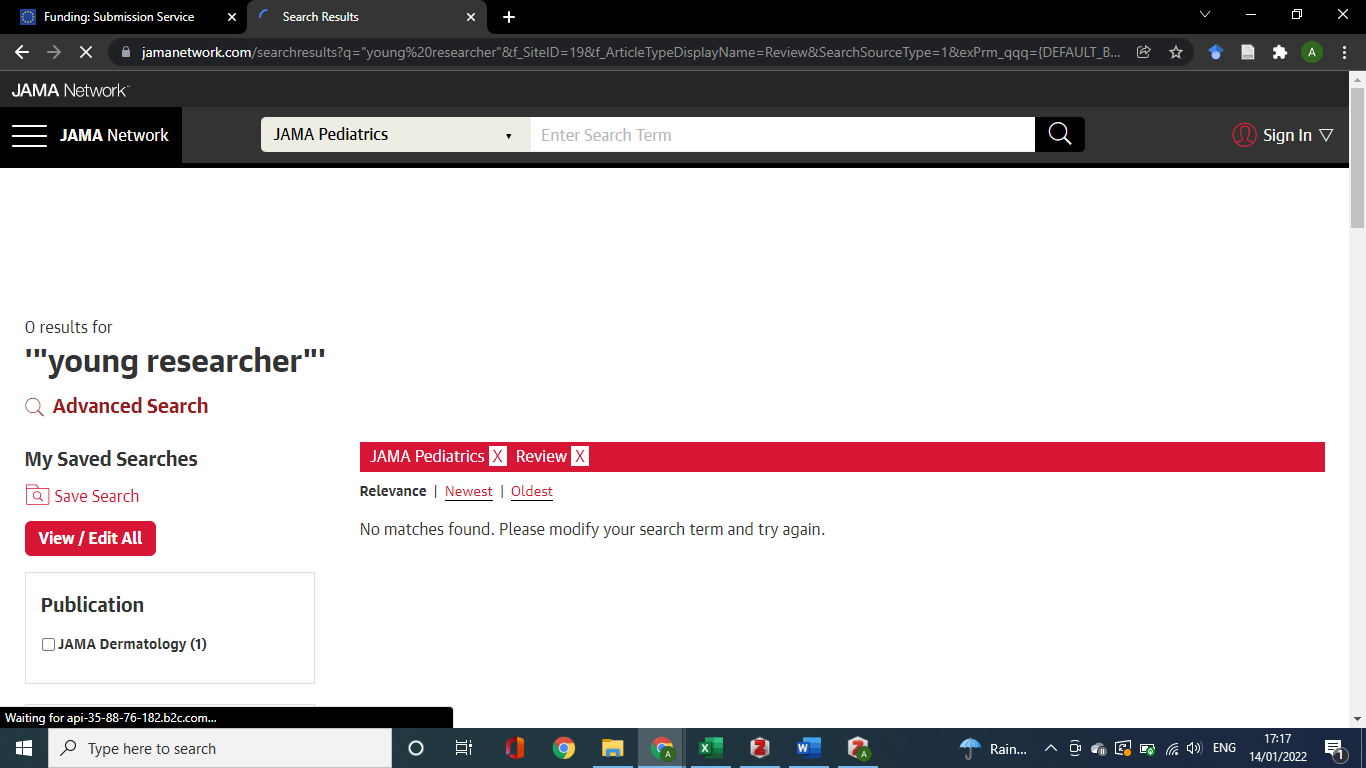 | |
| 18. "co researcher" | 0 |
| 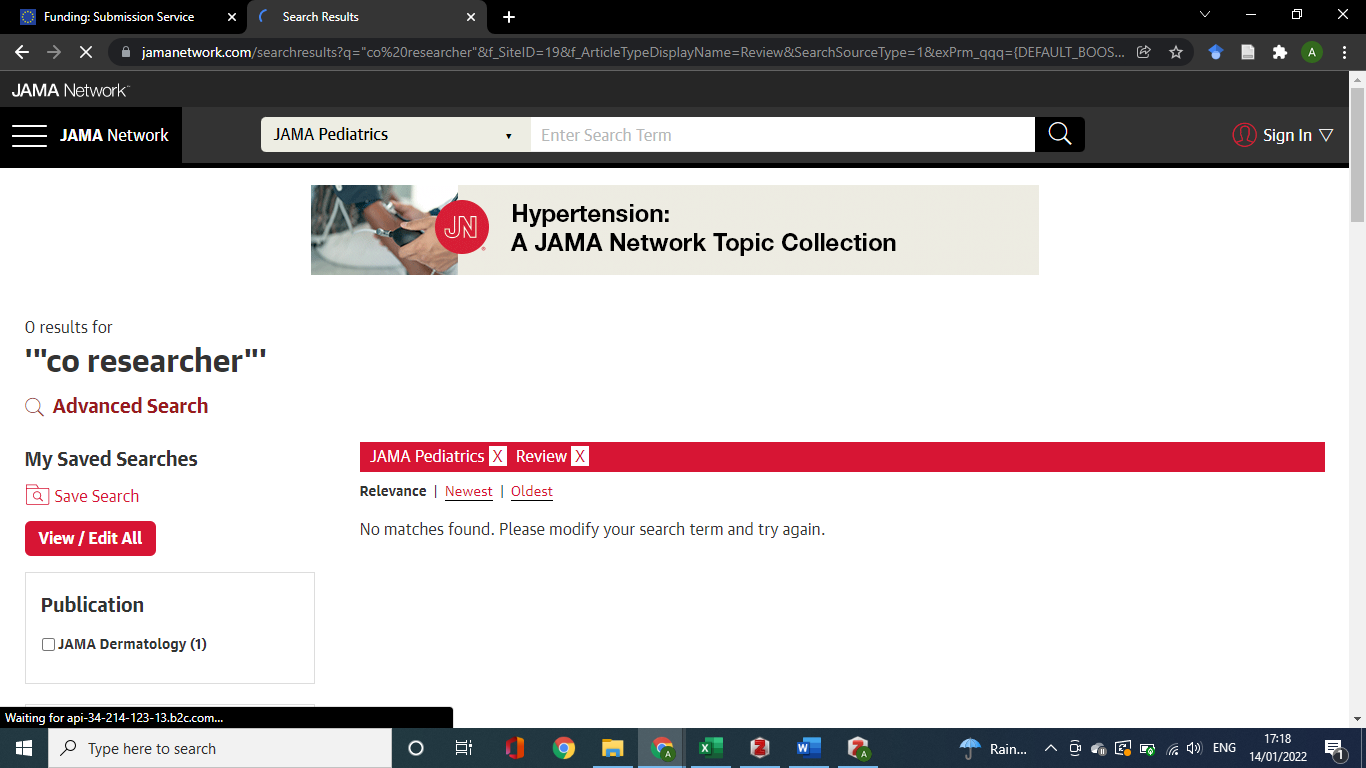 | |
| 19. "lived experience" | 3 |
| 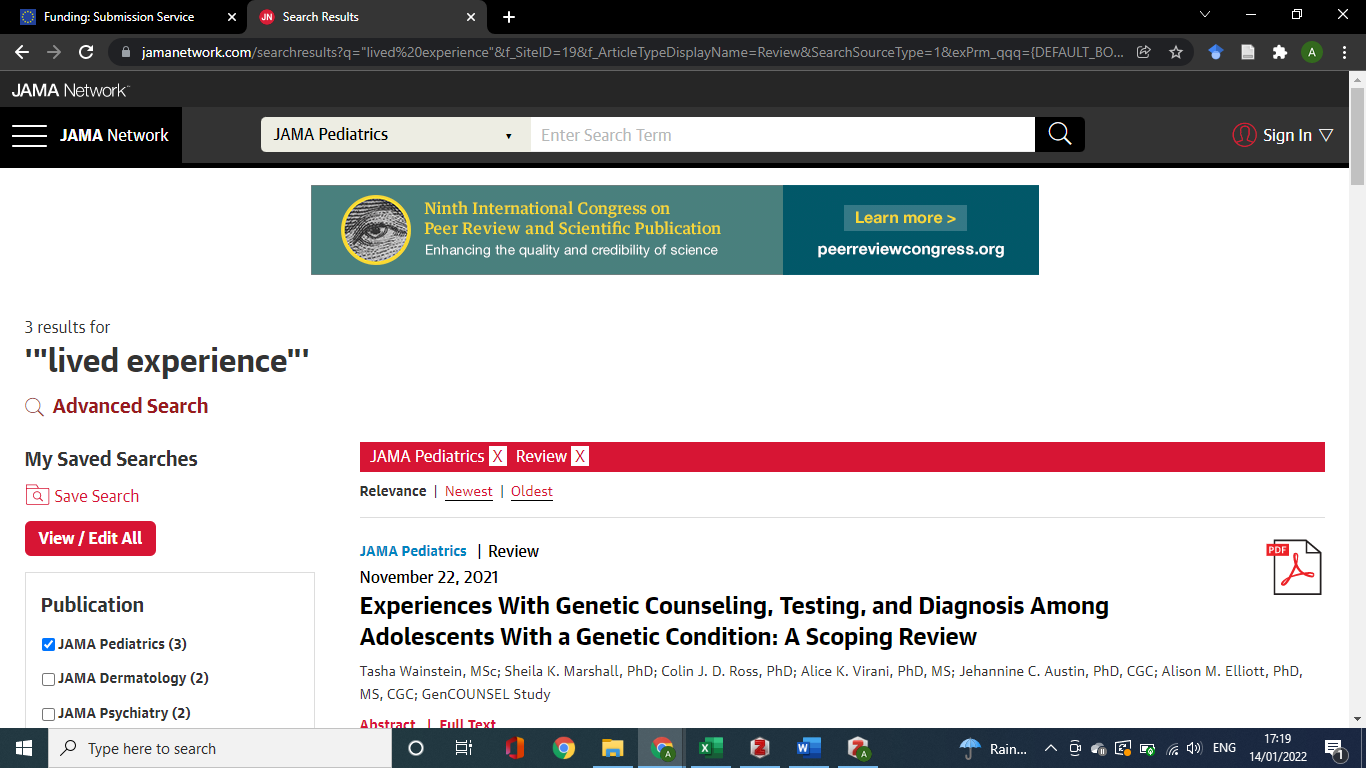 | |
