# Supplementary material for: An umbrella review of reviews on challenges to meaningful adolescent involvement in health research
Source: Health Expect. 2024 Jan 27;27(1):e13980. doi: 10.1111/hex.13980 (PMC10821743; doi:10.1111/hex.13980)
Supplement: Supplementary file 1 — Supporting information. [file HEX-27-e13980-s001.zip › Search record and results/Other sources/10 Journals/3. Developmental Review/Journal of Developmental Review search strings and results.docx]

**Overview**

Journal 1: Journal of Developmental Review

Date of search: 11^th^ January

Search terms/strings used to search the journal= 8

Search fields= Title, abstract, keywords

| **Search terms/strings** | **Results** | |
| --- | --- | --- |
| 1. “Youth involvement” | 0 | |
| 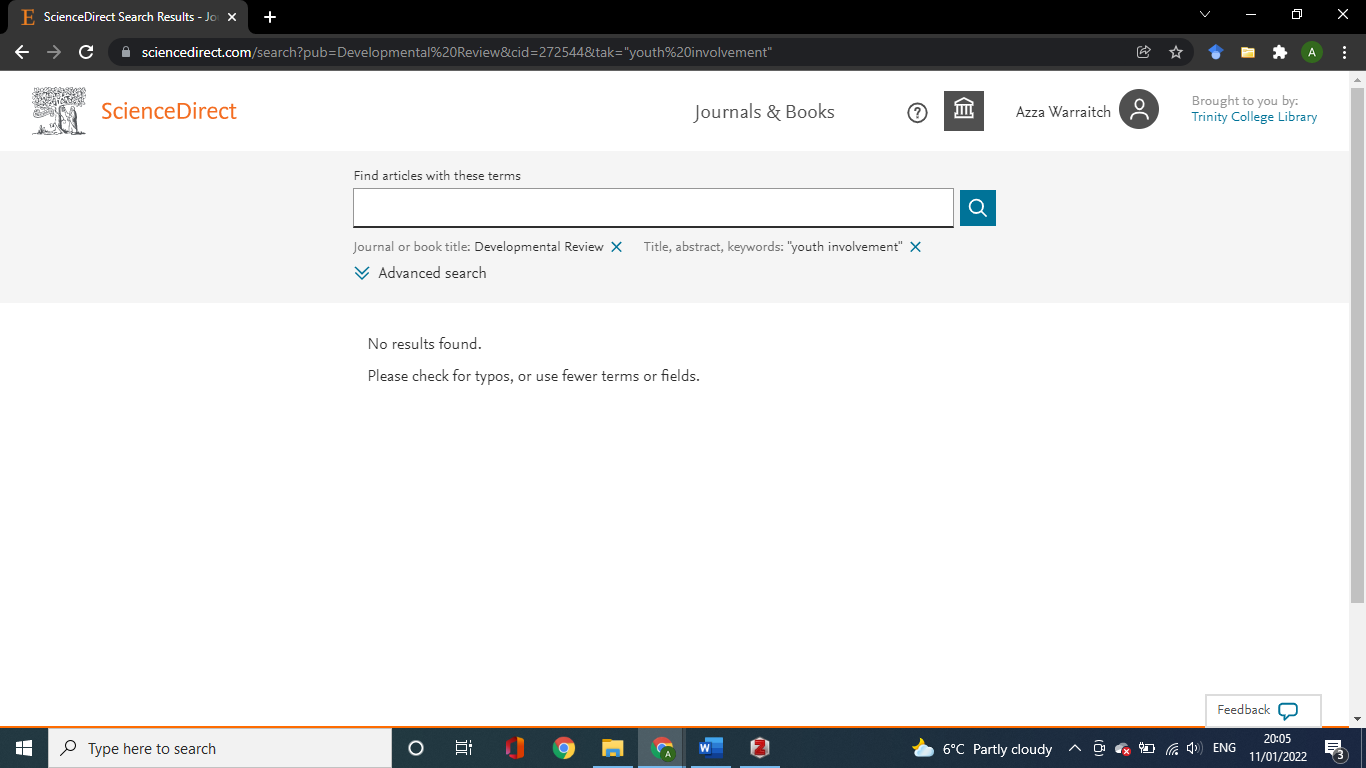 | | |
| 1. “Youth engagement” | 0 | |
| 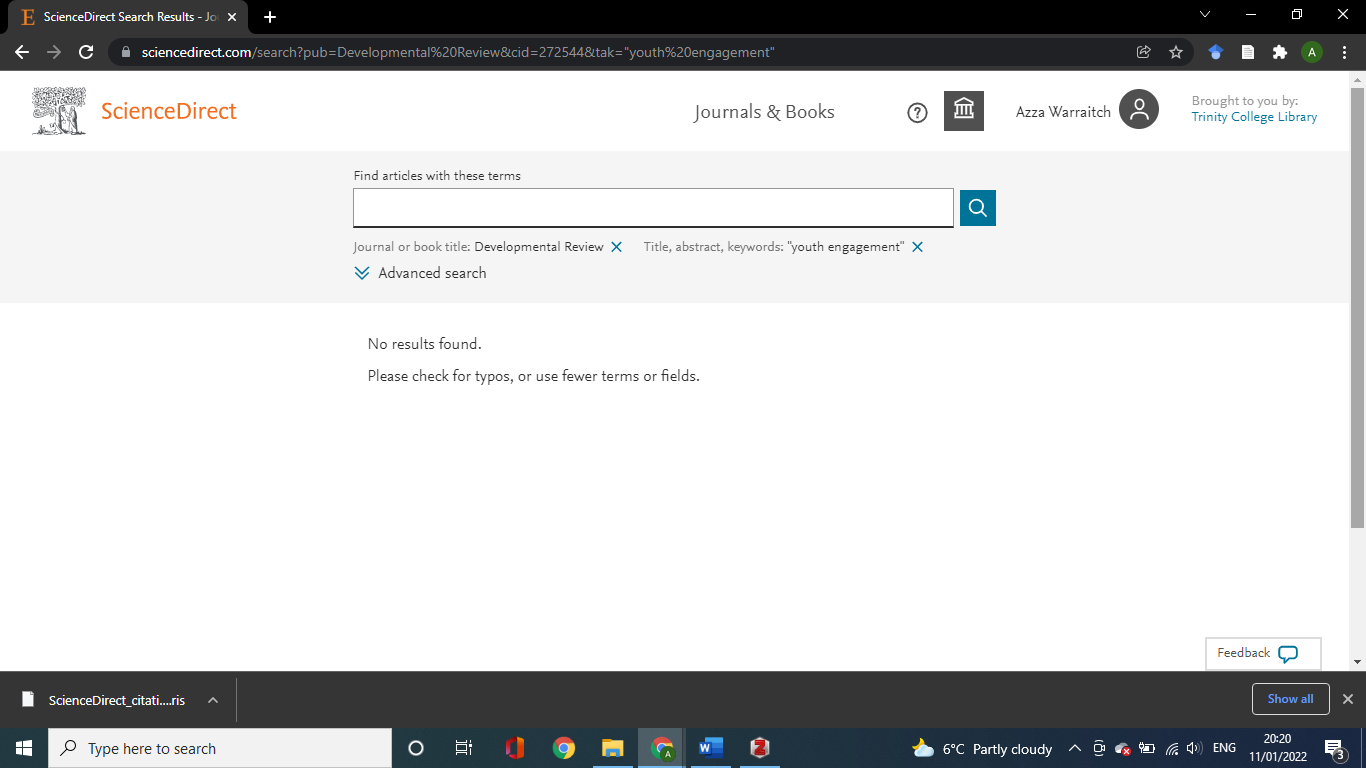 |  | |
| 1. "adolescent involvement" | 0 | |
| 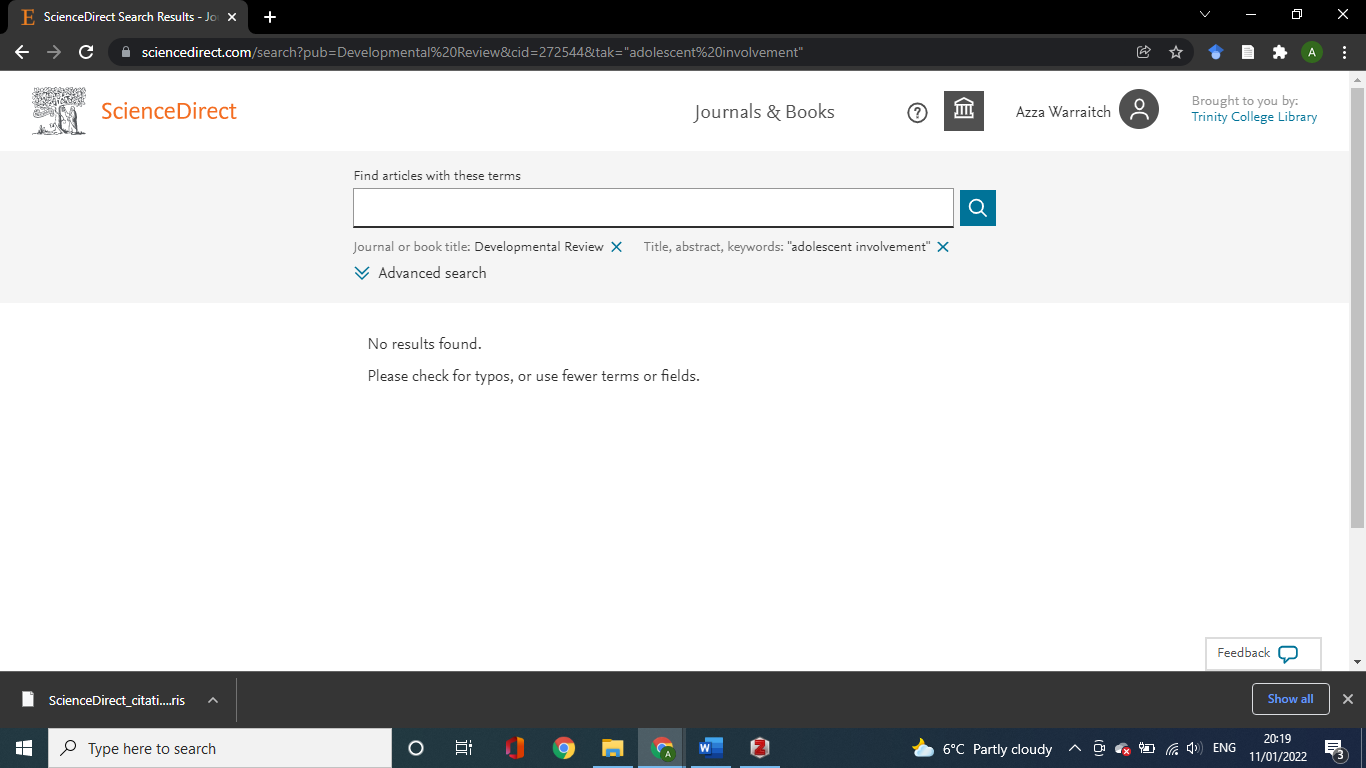 |  | |
| 1. "adolescent engagement" | 0 | |
| 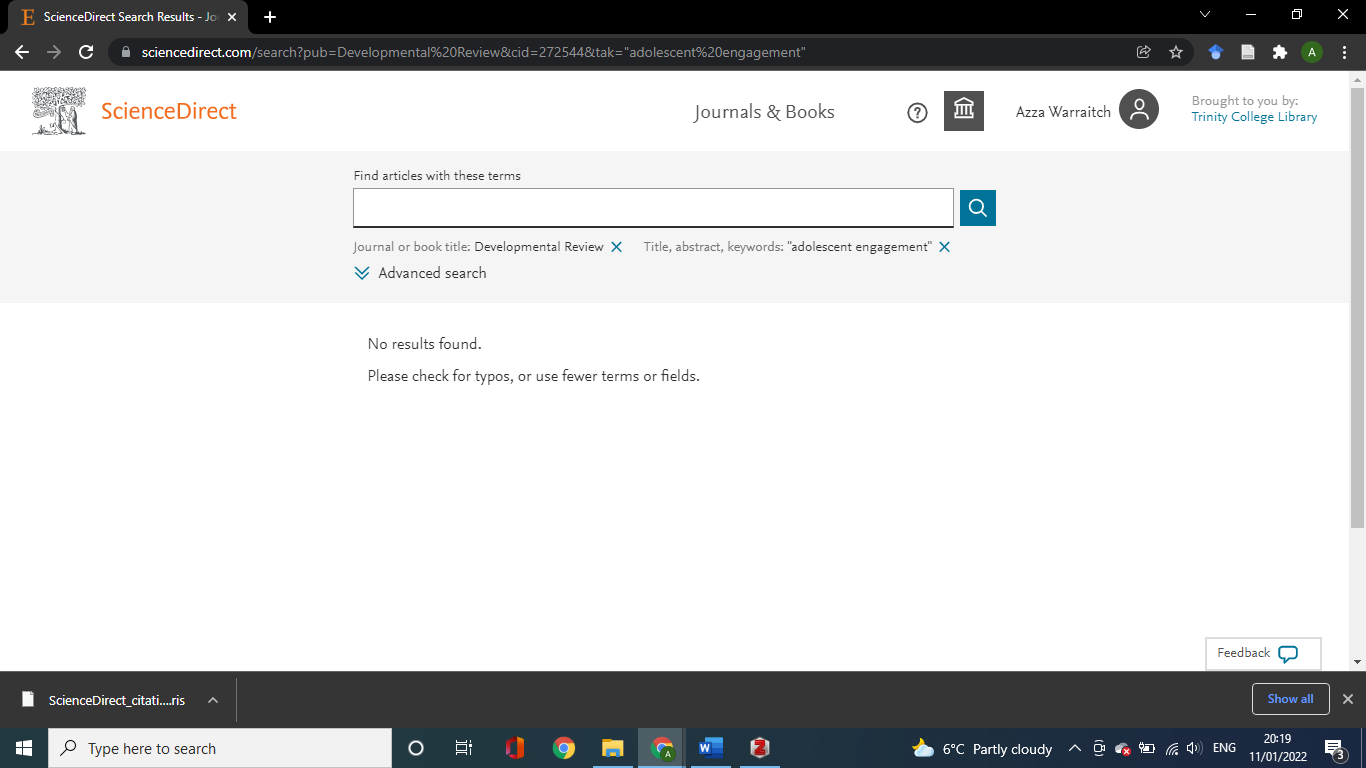 |  | |
| 5. Stakeholder OR participatory OR advisory | 0 | |
| 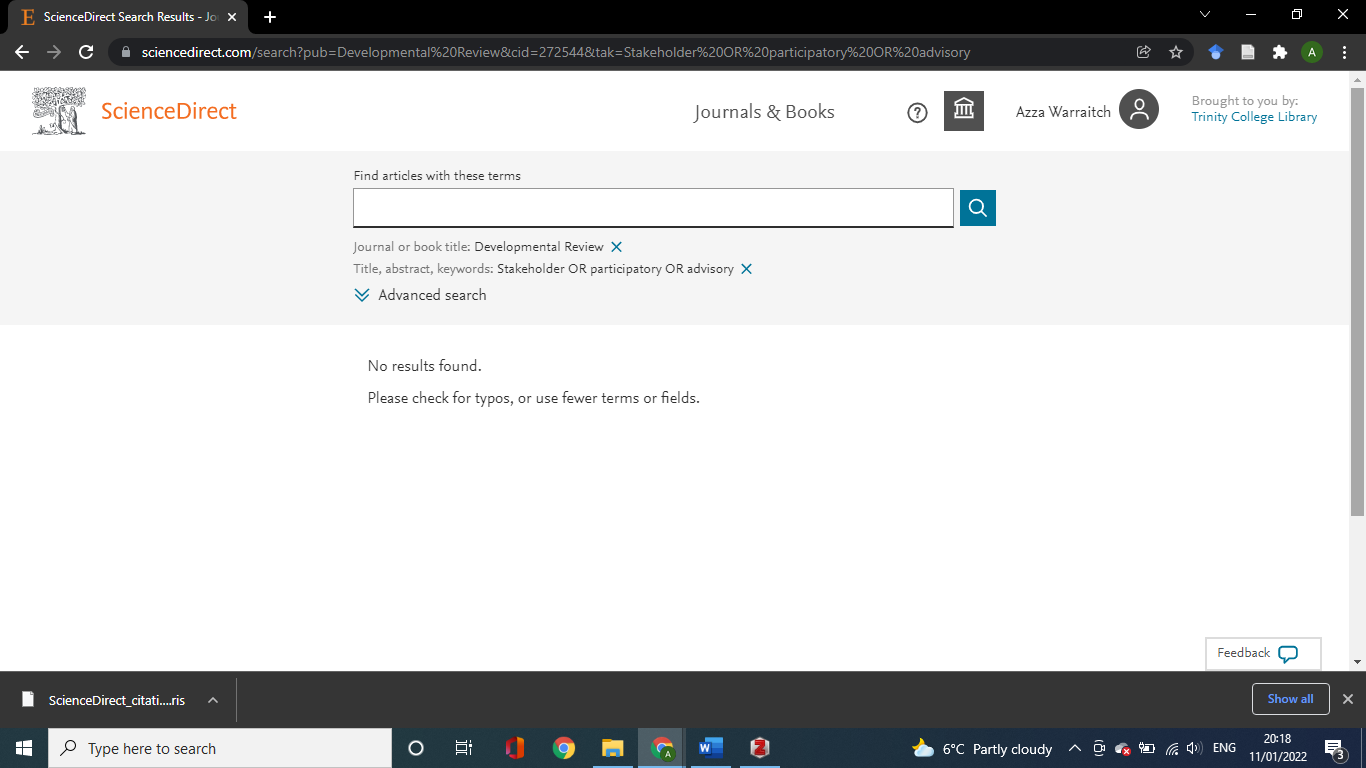 | | |
| 6. "patient and public involvement" OR "public and patient involvement" OR "public patient involvement" OR "patient public involvement" | 0 | |
| 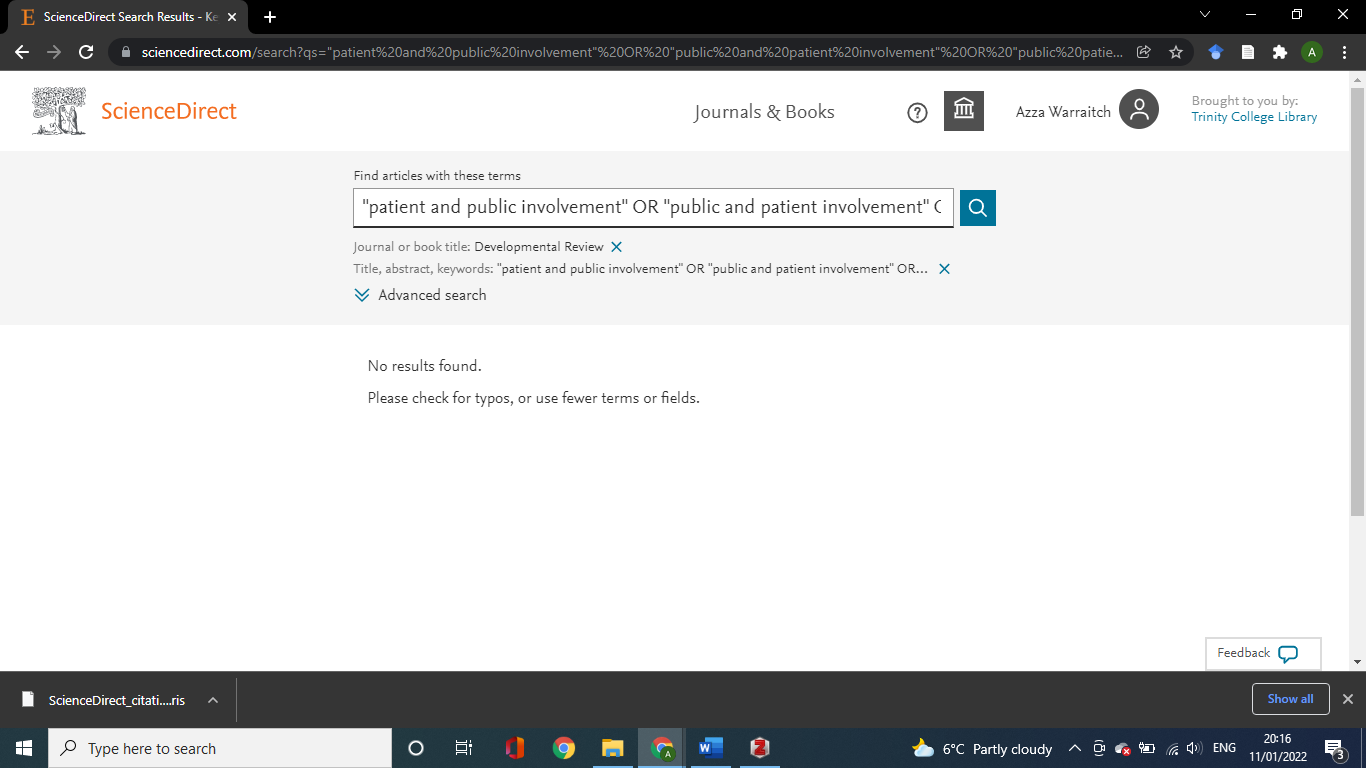 | | |
| 7. "co production" OR "co-design" OR "human centered design" OR "User centred design" | 0 | |
| 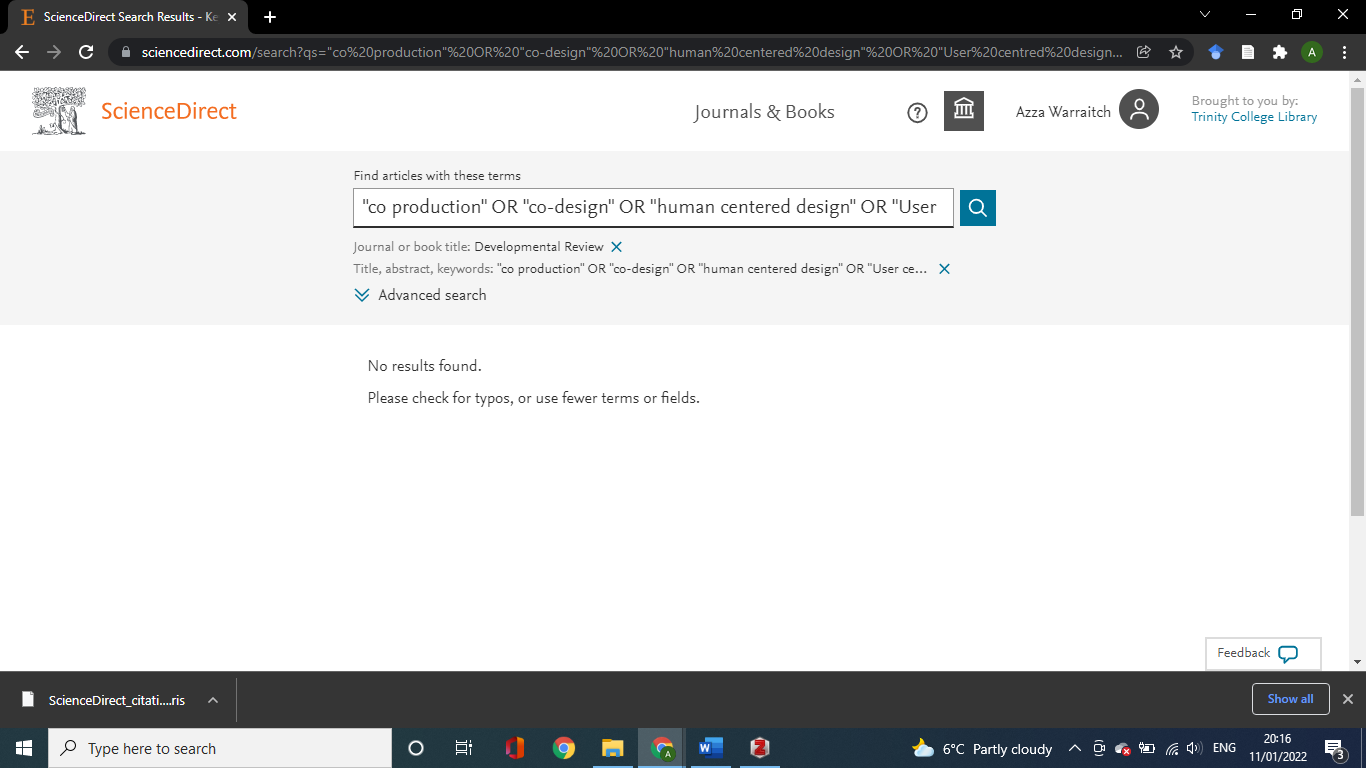 | | |
| 8. "peer researcher" OR "young researcher" OR "co researcher" OR "lived experience" | | 1 |
| 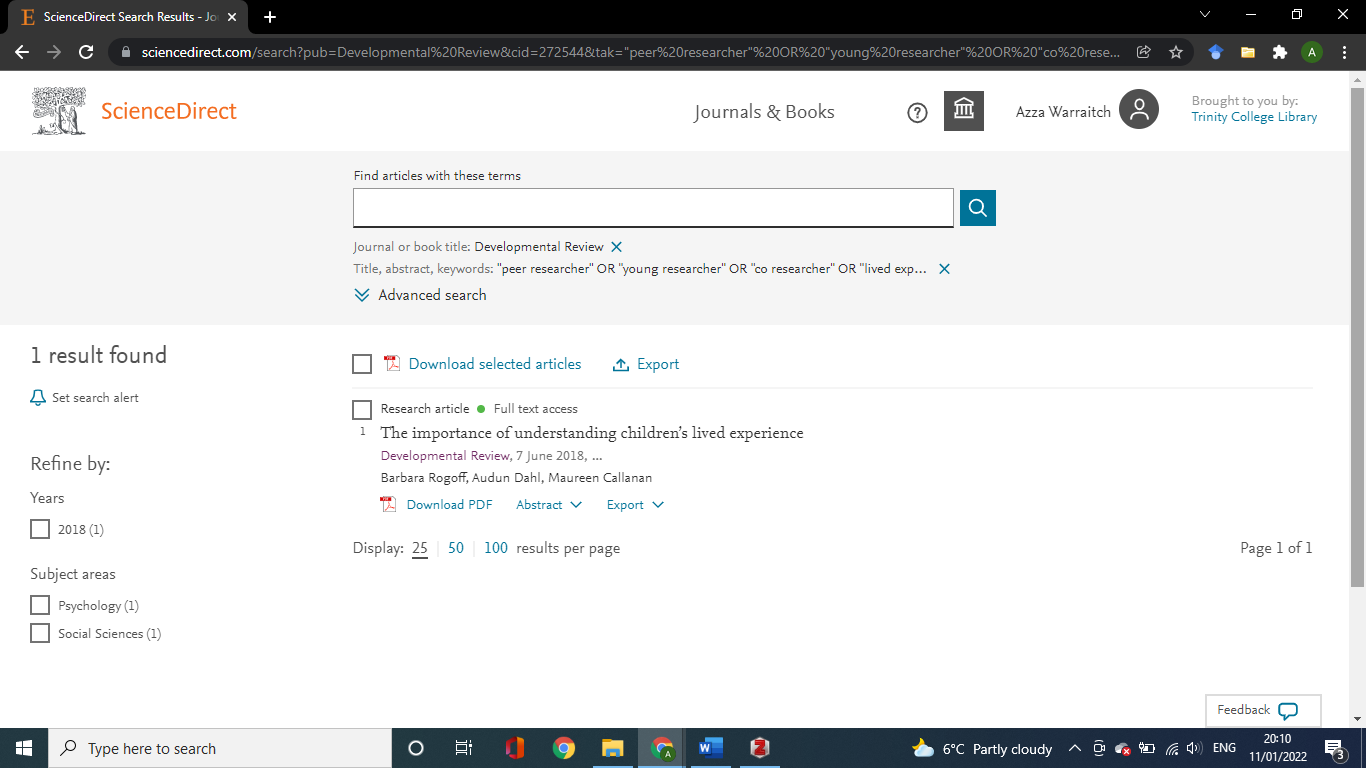 | | |
