# Supplementary material for: An umbrella review of reviews on challenges to meaningful adolescent involvement in health research
Source: Health Expect. 2024 Jan 27;27(1):e13980. doi: 10.1111/hex.13980 (PMC10821743; doi:10.1111/hex.13980)
Supplement: Supplementary file 1 — Supporting information. [file HEX-27-e13980-s001.zip › Search record and results/Other sources/10 Journals/10- Journal of Cystic Fibrosis/Journal of Cystic Fibrosis search strings and results.docx]

**Overview**

Journal 10: Journal of Cystic Fibrosis

Date of search: 11^th^ January

Search terms/strings used to search the journal= 6

| **Search terms/strings** | | **Results** |
| --- | --- | --- |
| 1. ("health research") AND (child* OR youth OR adolescen* OR "young people" OR "Young person*" OR "Young adult*" OR teen* OR juven*) AND (Involv* OR "advisory group*" OR "research advisory group" OR "research advisory panel*" OR "advisory panel" OR "advisory committee*" OR "advisory board*" OR "youth engagement" OR "patient and public involvement" OR "public and patient involvement" OR "public patient involvement" OR "community based participatory research" OR "youth particip*" OR "adolescent engagement" OR "participatory design" OR "participatory action" OR "needs assessment*" OR "co produc*" OR "co design" OR "Human centered design" OR "Human centred design" OR "User centered design" OR "User centred design" OR "user involvement" OR "peer researcher*" OR "co researcher*" OR "Patient Participation" OR "young researcher*" OR "lived experience") **search fields**=title, abstract, keywords, **filter**=review | | 0 |
| 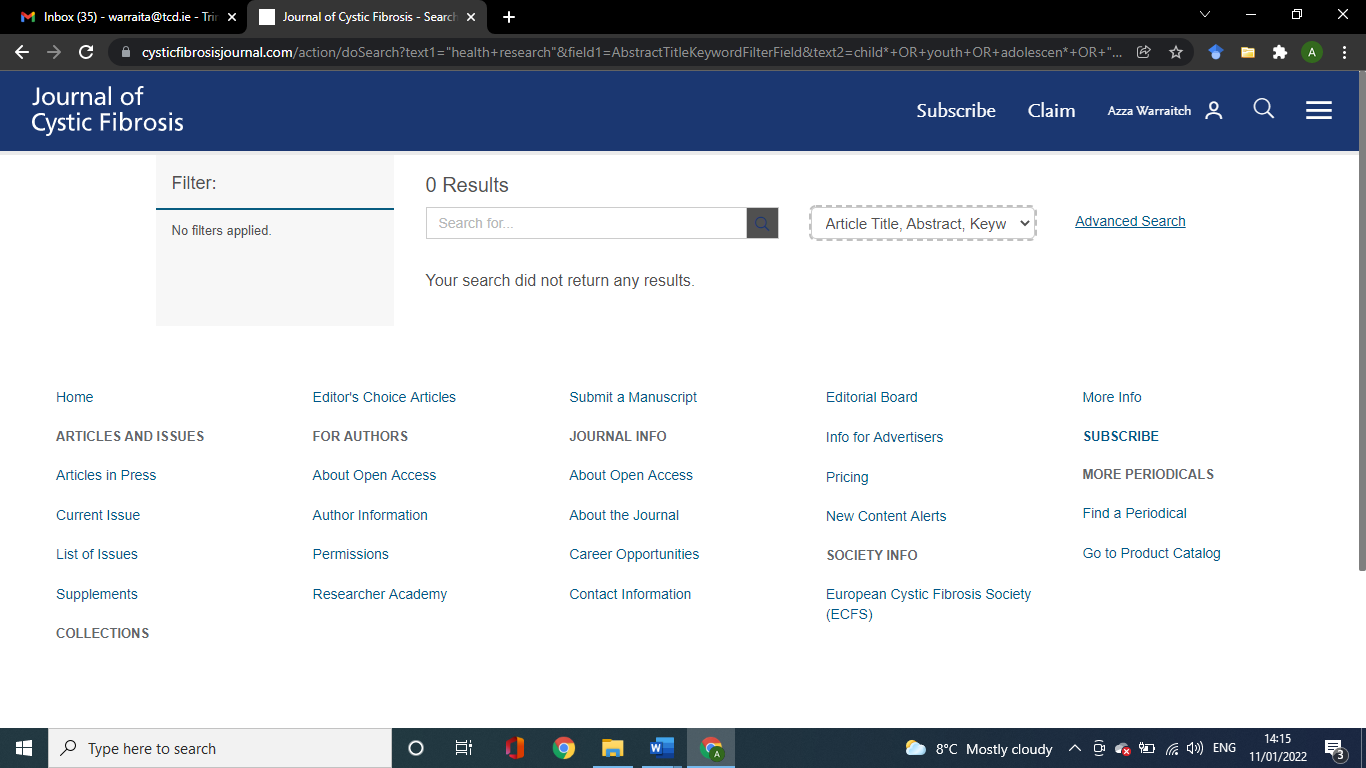 | | |
| 1. “Youth involvement” OR “Youth engagement” OR “adolescent involvement” or “adolescent engagement” **search fields**=title, abstract, keywords | | 0 |
| 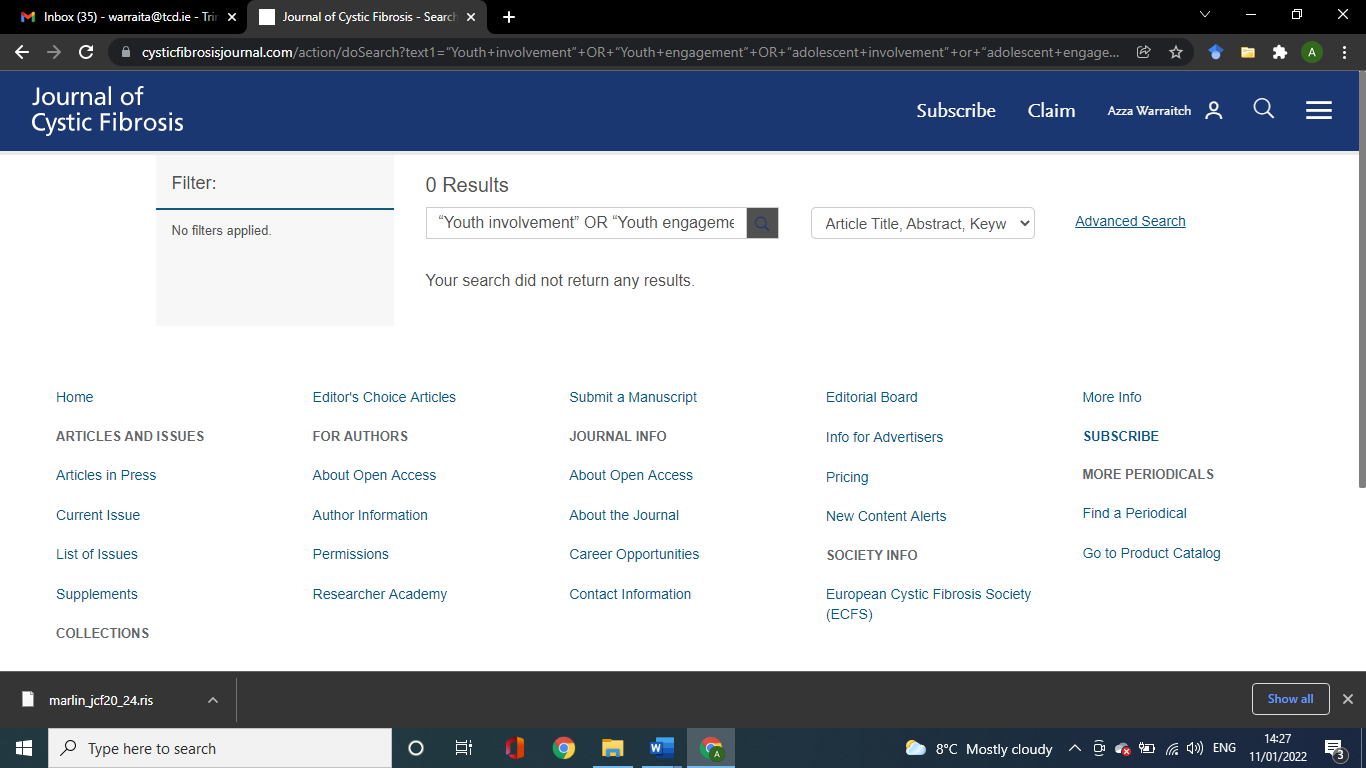 | | |
| 3. (child* OR youth OR adolescen* OR "young people" OR "Young person*" OR "Young adult*" OR teen* OR juven*) AND (Stakeholder OR participatory OR advisory) **search fields**=title, abstract, keywords, **filter**=review | | 3 |
| 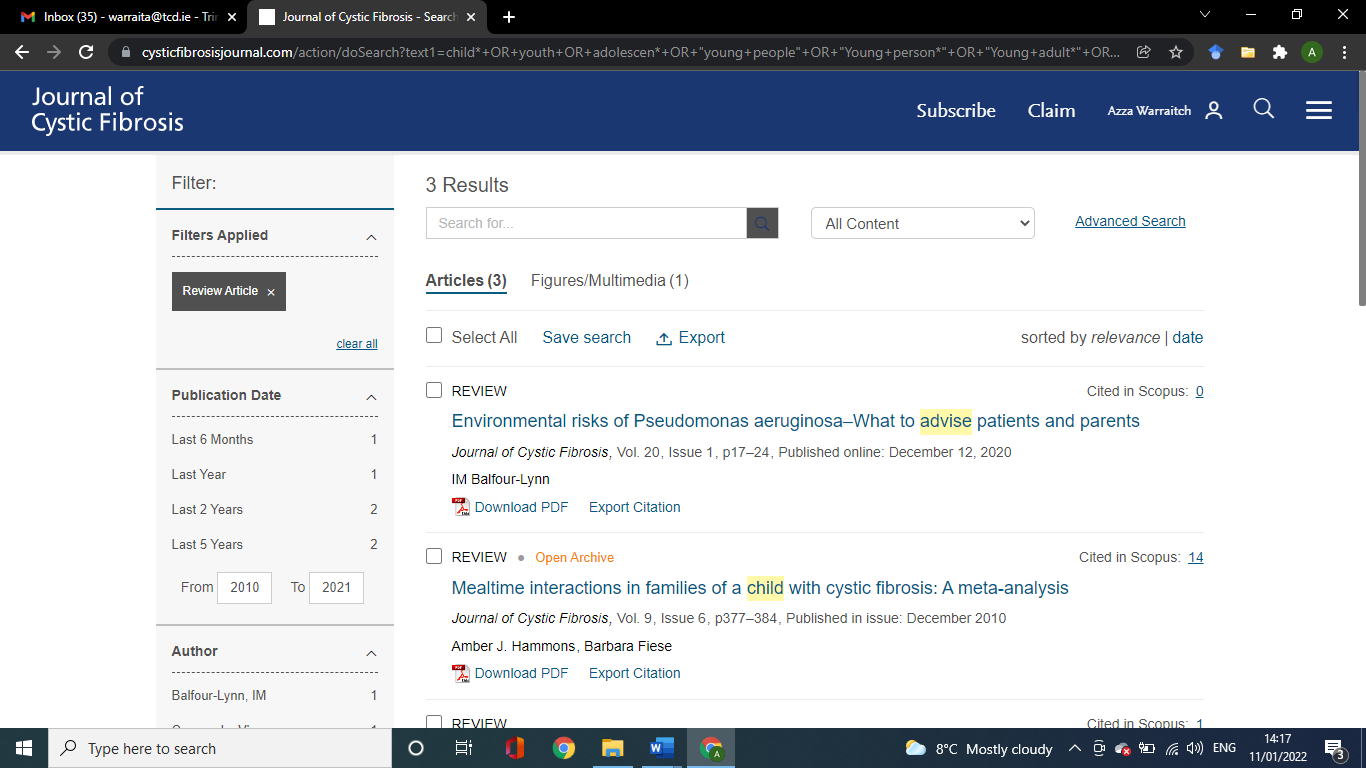 | | |
| 4. (child* OR youth OR adolescen* OR "young people" OR "Young person*" OR "Young adult*" OR teen* OR juven*) AND ("patient and public involvement" OR "public and patient involvement" OR "public patient involvement" OR "patient public involvement") **search fields**=title, abstract, keywords | | 0 |
| 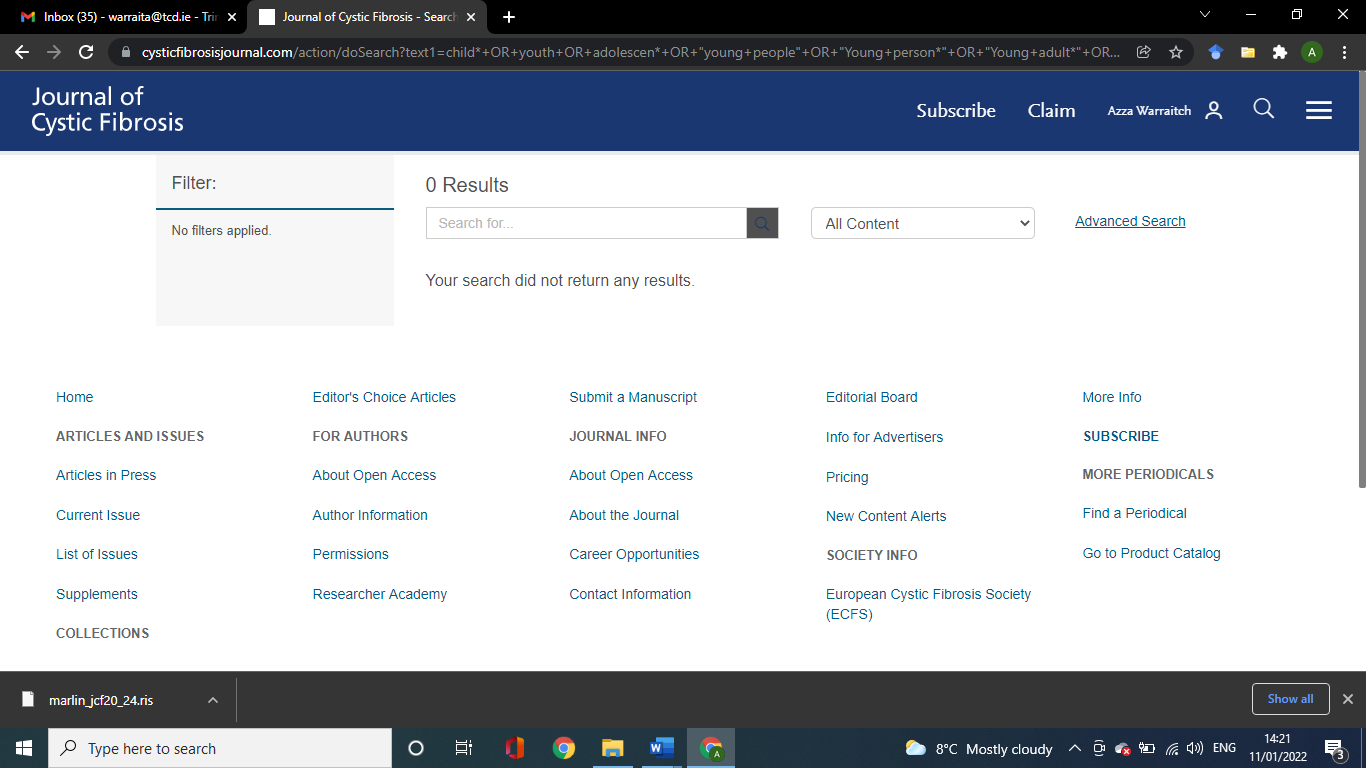 | | |
| 5. (child* OR youth OR adolescen* OR "young people" OR "Young person*" OR "Young adult*" OR teen* OR juven*) AND ("co production" OR "co-design" OR "human centered design" OR "User centred design") **search fields**=title, abstract, keywords | | 0 |
| 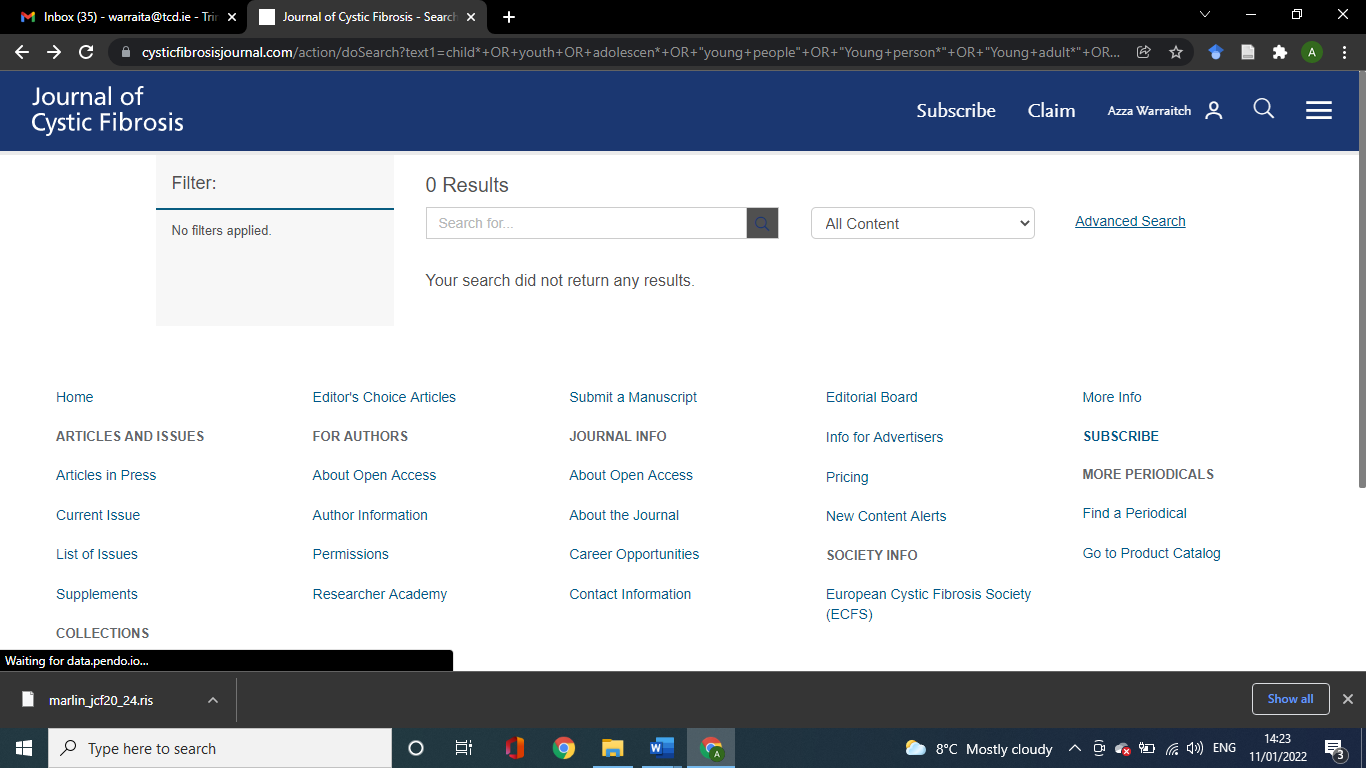 | | |
| 6. (child* OR youth OR adolescen* OR "young people" OR "Young person*" OR "Young adult*" OR teen* OR juven*) AND ("peer researcher" OR "young researcher" OR "co researcher" OR "lived experience") **search fields**=title, abstract, keywords, **filter**=review | 0 | |
|  | | |
