# Supplementary material for: An umbrella review of reviews on challenges to meaningful adolescent involvement in health research
Source: Health Expect. 2024 Jan 27;27(1):e13980. doi: 10.1111/hex.13980 (PMC10821743; doi:10.1111/hex.13980)
Supplement: Supplementary file 1 — Supporting information. [file HEX-27-e13980-s001.zip › Search record and results/Other sources/10 Journals/Total results and search record/Total results for ten journals.docx]

| **Journals** | **Results** |
| --- | --- |
| JAMA | 173* |
| Journal of Child Psychology and Psychiatry and Allied Disciplines | 741 |
| Journal of Developmental Review | 1 |
| Child Development | 17 |
| Child Development perspectives | 0 |
| Lancet | 3 |
| Pediatrics | 173** |
| Journal of Clinical Child and Family Psychology Review | 98 |
| Journal of Positive Behavior Interventions | 19 |
| Journal of Cystic Fibrosis | 3 |
| Total | 1228 |

**Total results for ten journals**

** 8 references not exported*

*** 2 references not exported*
