# Supplementary material for: An umbrella review of reviews on challenges to meaningful adolescent involvement in health research
Source: Health Expect. 2024 Jan 27;27(1):e13980. doi: 10.1111/hex.13980 (PMC10821743; doi:10.1111/hex.13980)
Supplement: Supplementary file 1 — Supporting information. [file HEX-27-e13980-s001.zip › Search record and results/Other sources/10 Journals/7- Pediatrics/Journal of Paediatrics search strings and results.docx]

**Overview**

Journal 7: Pediatrics

Date of search: 11^th^ January

Search terms/strings used to search the journal= 8

Filter=review

| **Search terms/strings** | **Results** |
| --- | --- |
| 1. “Youth involvement” | 3 |
| 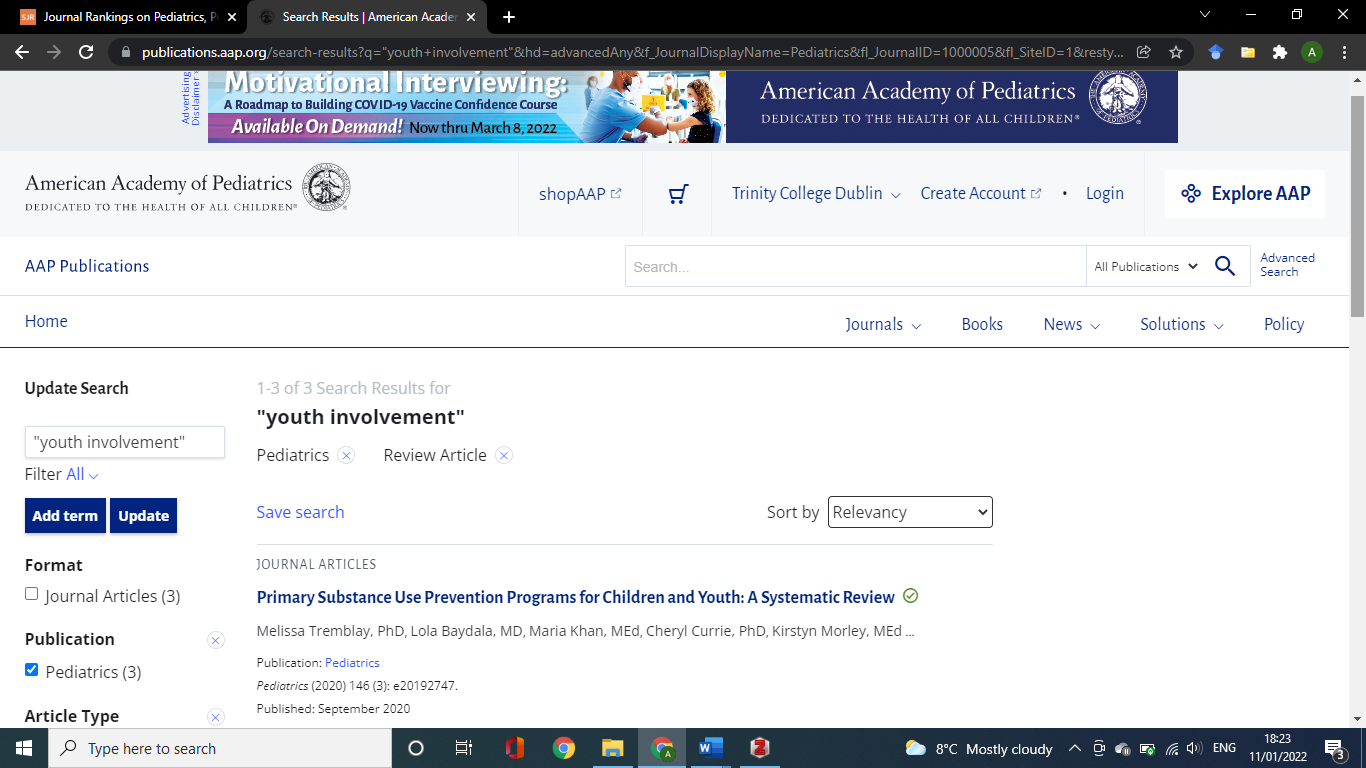 | |
| 1. “Youth engagement” | 1 |
| 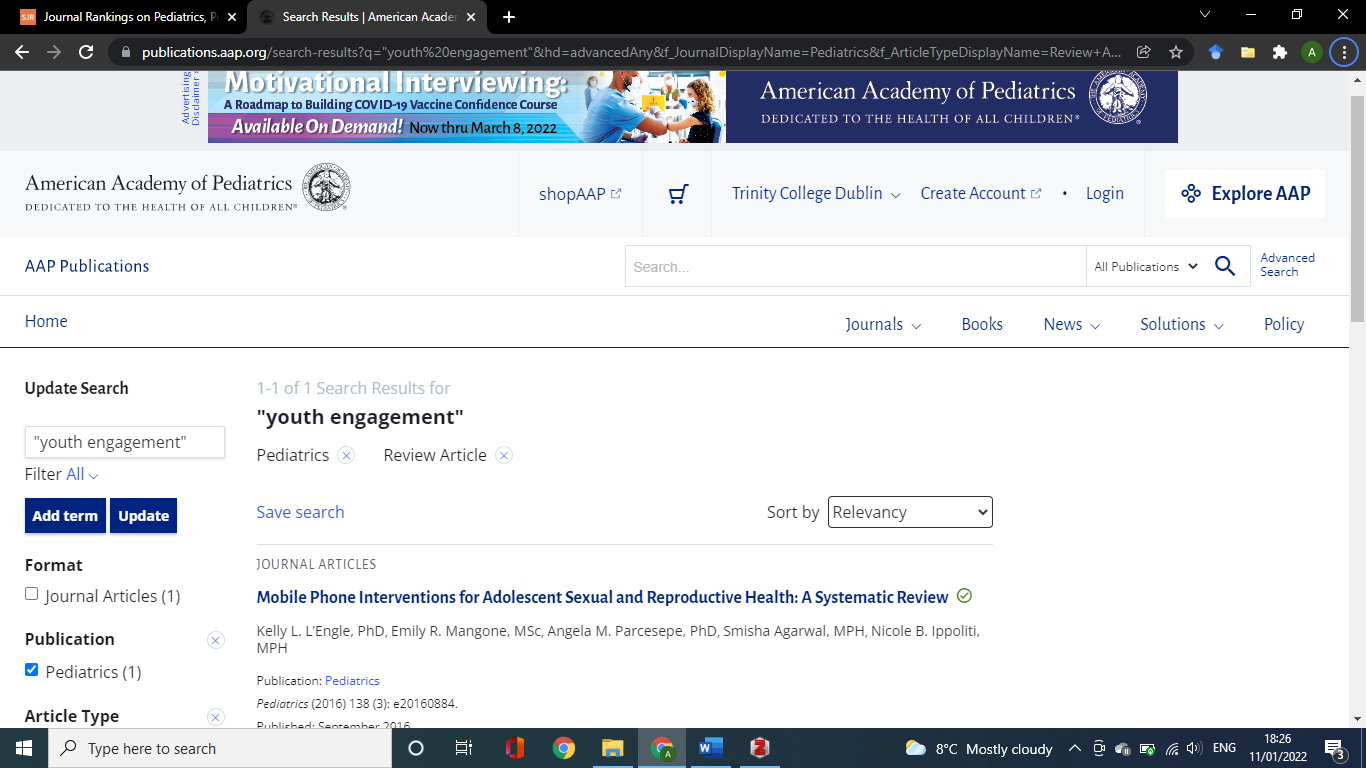 | |
| 1. “adolescent involvement” | 6 |
| 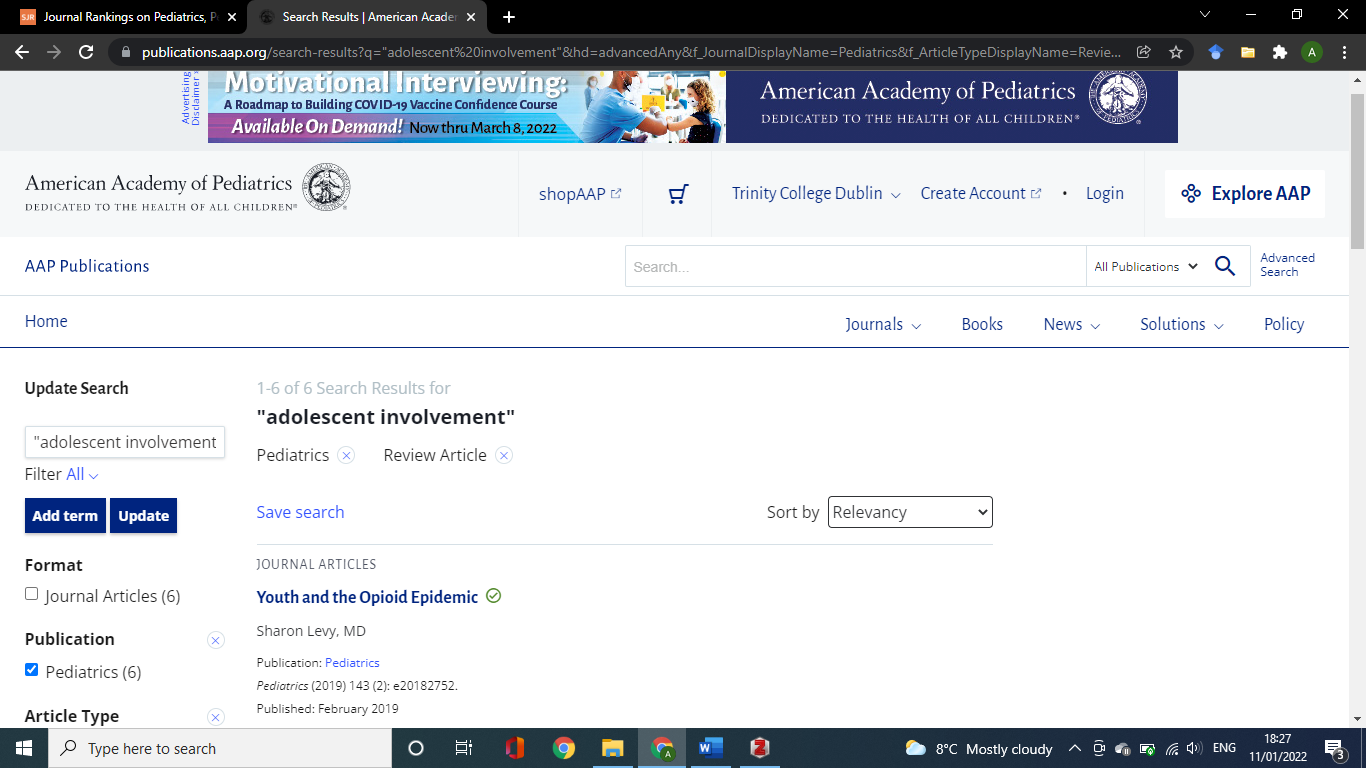 | |
| 1. “adolescent engagement” | 7 |
| 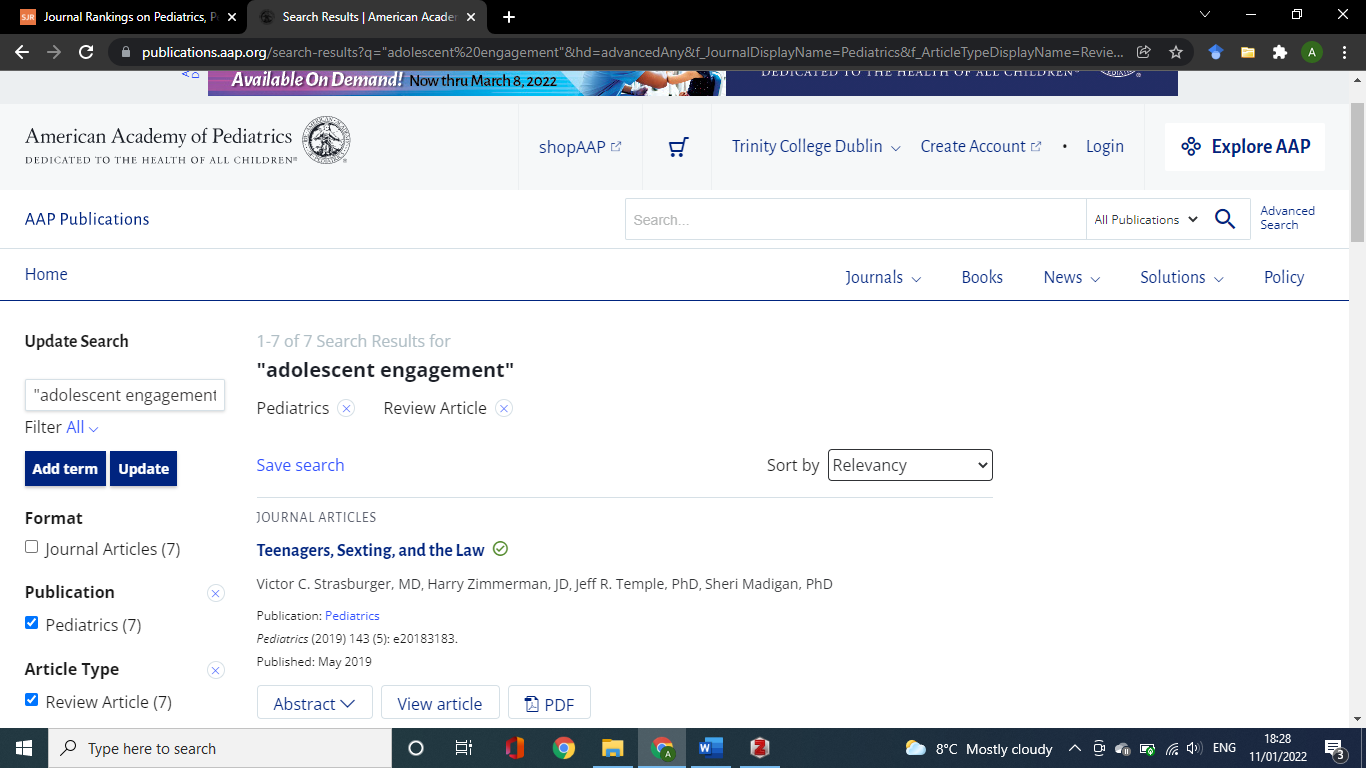 | 0 |
| Full Text: Stakeholder OR participatory OR advisory  [Close](https://publications.aap.org/search-results?hd=advancedAny&f_JournalDisplayName=Pediatrics&f_ArticleTypeDisplayName=Review+Article&fl_JournalID=1000005&fl_SiteID=1&restypeid=3&qb=%7b%22FullText1%22:%22Stakeholder%20OR%20participatory%20OR%20advisory%22,%22Abstract2%22:%22child*%20OR%20youth%20OR%20adolescen*%20OR%20\%22young%20people\%22%20OR%20\%22Young%20person*\%22%20OR%20\%22Young%20adult*\%22%20OR%20teen*%20OR%20juven*%22%7d&page=1)  Abstract: child* OR youth OR adolescen* OR "young people" OR "Young person*" OR "Young adult*" OR teen* OR juven* | 24 |
| 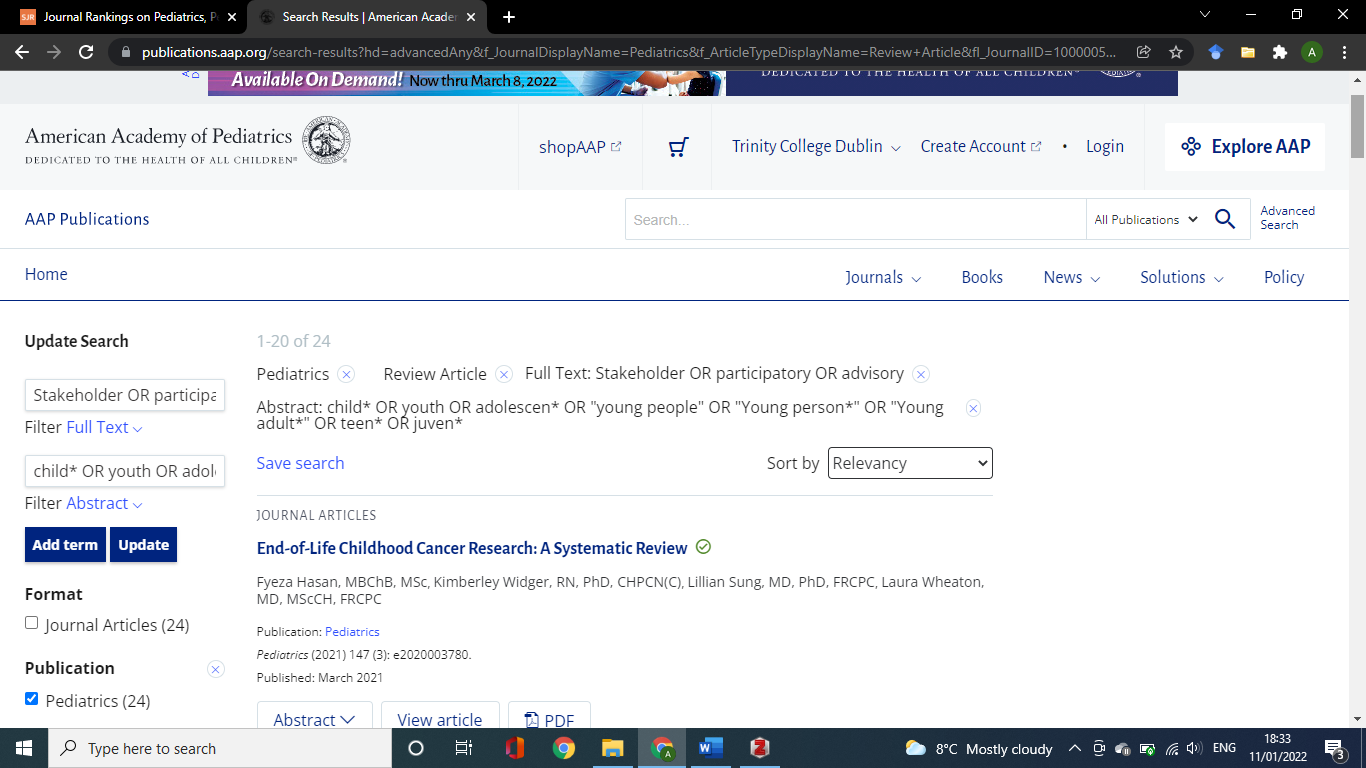 | |
| Review Article  Full Text: "patient and public involvement" OR "public and patient involvement" OR "public patient involvement" OR "patient public involvement"  Abstract: child* OR youth OR adolescen* OR "young people" OR "Young person*" OR "Young adult*" OR teen* OR juven* | 0 |
| 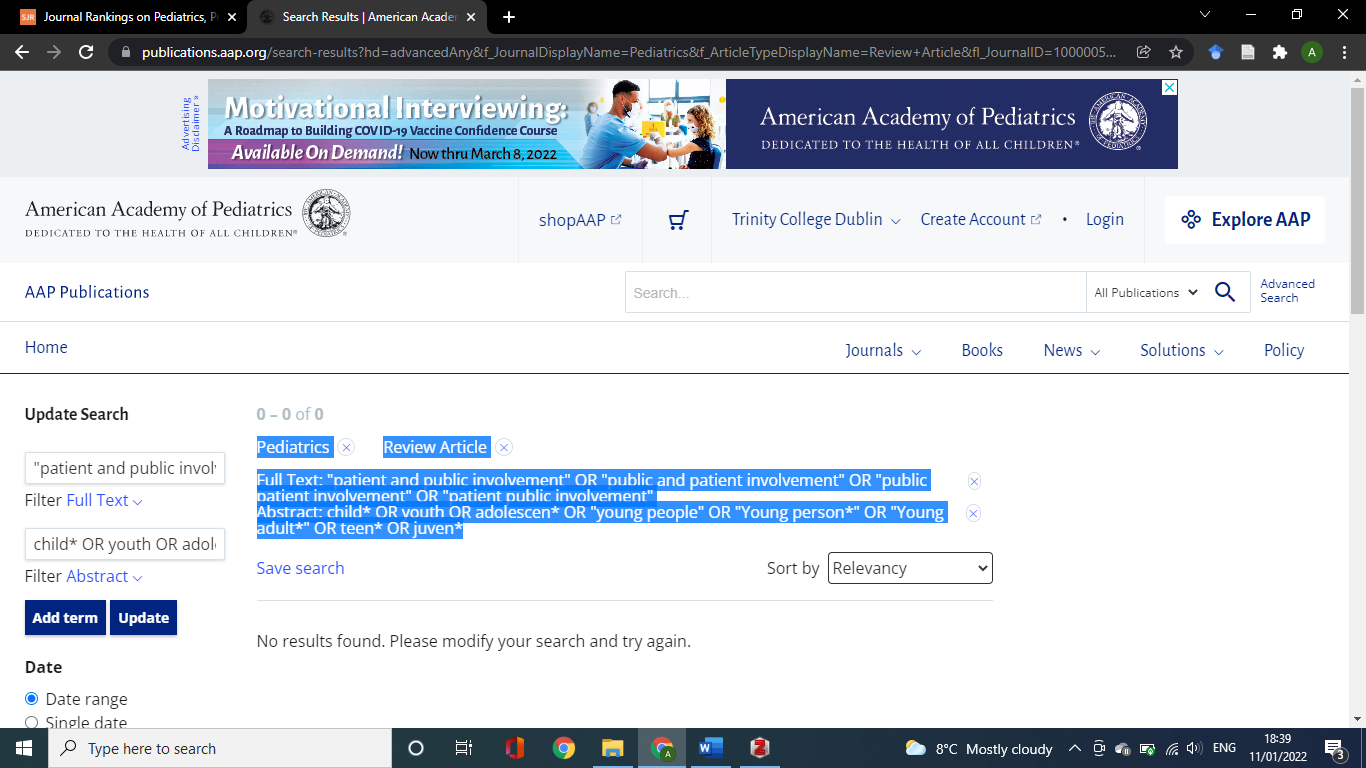 | |
| 1. Review Article   [Close](https://publications.aap.org/search-results?hd=advancedAny&f_JournalDisplayName=Pediatrics&f_ArticleTypeDisplayName=Review+Article&fl_JournalID=1000005&fl_SiteID=1&restypeid=3&qb=%7b%22FullText1%22:%22\%22co%20production\%22%20OR%20\%22co-design\%22%20OR%20\%22human%20centered%20design\%22%20OR%20\%22User%20centred%20design\%22%22,%22Abstract2%22:%22child*%20OR%20youth%20OR%20adolescen*%20OR%20\%22young%20people\%22%20OR%20\%22Young%20person*\%22%20OR%20\%22Young%20adult*\%22%20OR%20teen*%20OR%20juven*%22%7d&page=1)  Full Text: "co production" OR "co-design" OR "human centered design" OR "User centred design"  [Close](https://publications.aap.org/search-results?hd=advancedAny&f_JournalDisplayName=Pediatrics&f_ArticleTypeDisplayName=Review+Article&fl_JournalID=1000005&fl_SiteID=1&restypeid=3&qb=%7b%22FullText1%22:%22\%22co%20production\%22%20OR%20\%22co-design\%22%20OR%20\%22human%20centered%20design\%22%20OR%20\%22User%20centred%20design\%22%22,%22Abstract2%22:%22child*%20OR%20youth%20OR%20adolescen*%20OR%20\%22young%20people\%22%20OR%20\%22Young%20person*\%22%20OR%20\%22Young%20adult*\%22%20OR%20teen*%20OR%20juven*%22%7d&page=1)  Abstract: child* OR youth OR adolescen* OR "young people" OR "Young person*" OR "Young adult*" OR teen* OR juven* | 132 |
| 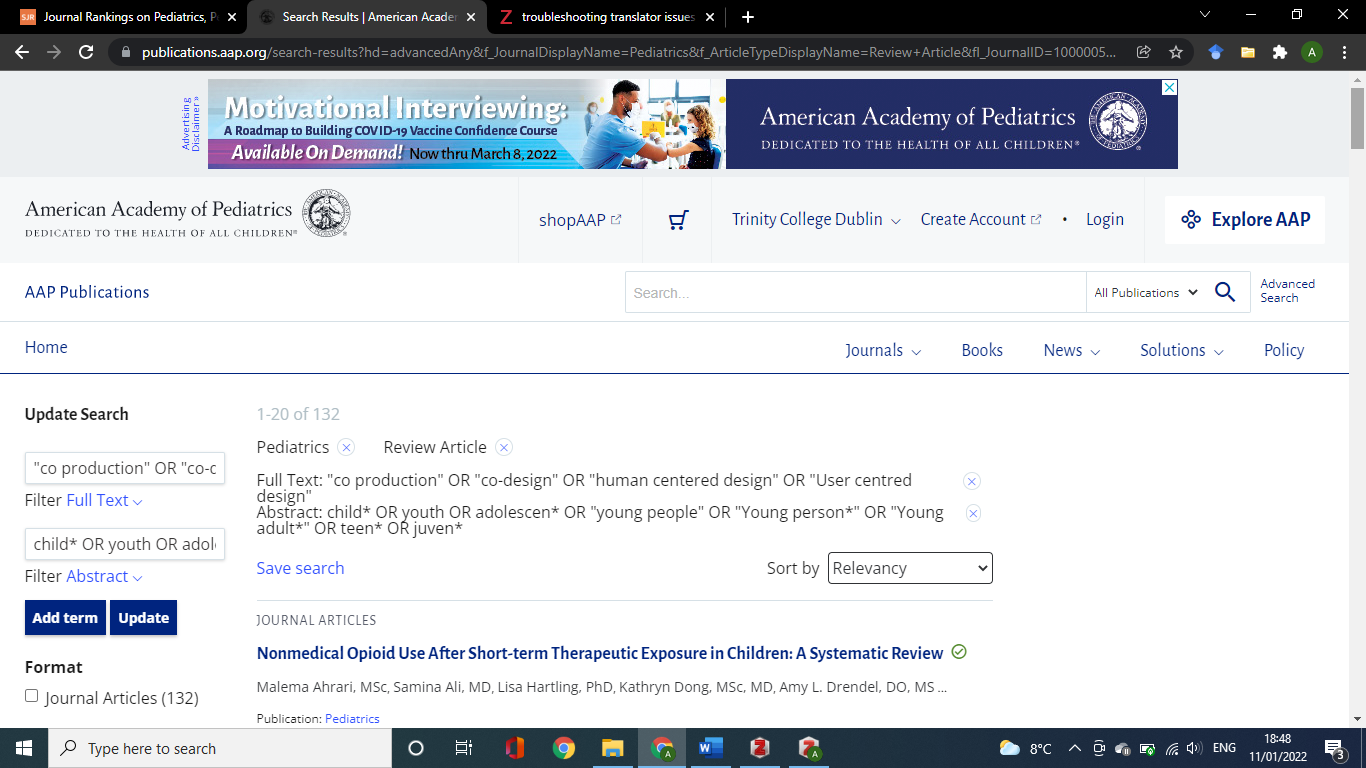 | |
| 1. Pediatrics   [Close](https://publications.aap.org/search-results?hd=advancedAny&f_JournalDisplayName=Pediatrics&f_ArticleTypeDisplayName=Review+Article&fl_JournalID=1000005&fl_SiteID=1&restypeid=3&qb=%7b%22FullText1%22:%22\%22peer%20researcher\%22%20OR%20\%22young%20researcher\%22%20OR%20\%22co%20researcher\%22%20OR%20\%22lived%20experience\%22%22,%22Abstract2%22:%22child*%20OR%20youth%20OR%20adolescen*%20OR%20\%22young%20people\%22%20OR%20\%22Young%20person*\%22%20OR%20\%22Young%20adult*\%22%20OR%20teen*%20OR%20juven*%22%7d&page=1)  Review Article  [Close](https://publications.aap.org/search-results?hd=advancedAny&f_JournalDisplayName=Pediatrics&f_ArticleTypeDisplayName=Review+Article&fl_JournalID=1000005&fl_SiteID=1&restypeid=3&qb=%7b%22FullText1%22:%22\%22peer%20researcher\%22%20OR%20\%22young%20researcher\%22%20OR%20\%22co%20researcher\%22%20OR%20\%22lived%20experience\%22%22,%22Abstract2%22:%22child*%20OR%20youth%20OR%20adolescen*%20OR%20\%22young%20people\%22%20OR%20\%22Young%20person*\%22%20OR%20\%22Young%20adult*\%22%20OR%20teen*%20OR%20juven*%22%7d&page=1)  Full Text: "peer researcher" OR "young researcher" OR "co researcher" OR "lived experience"  [Close](https://publications.aap.org/search-results?hd=advancedAny&f_JournalDisplayName=Pediatrics&f_ArticleTypeDisplayName=Review+Article&fl_JournalID=1000005&fl_SiteID=1&restypeid=3&qb=%7b%22FullText1%22:%22\%22peer%20researcher\%22%20OR%20\%22young%20researcher\%22%20OR%20\%22co%20researcher\%22%20OR%20\%22lived%20experience\%22%22,%22Abstract2%22:%22child*%20OR%20youth%20OR%20adolescen*%20OR%20\%22young%20people\%22%20OR%20\%22Young%20person*\%22%20OR%20\%22Young%20adult*\%22%20OR%20teen*%20OR%20juven*%22%7d&page=1)  Abstract: child* OR youth OR adolescen* OR "young people" OR "Young person*" OR "Young adult*" OR teen* OR juven* | 2 |
| 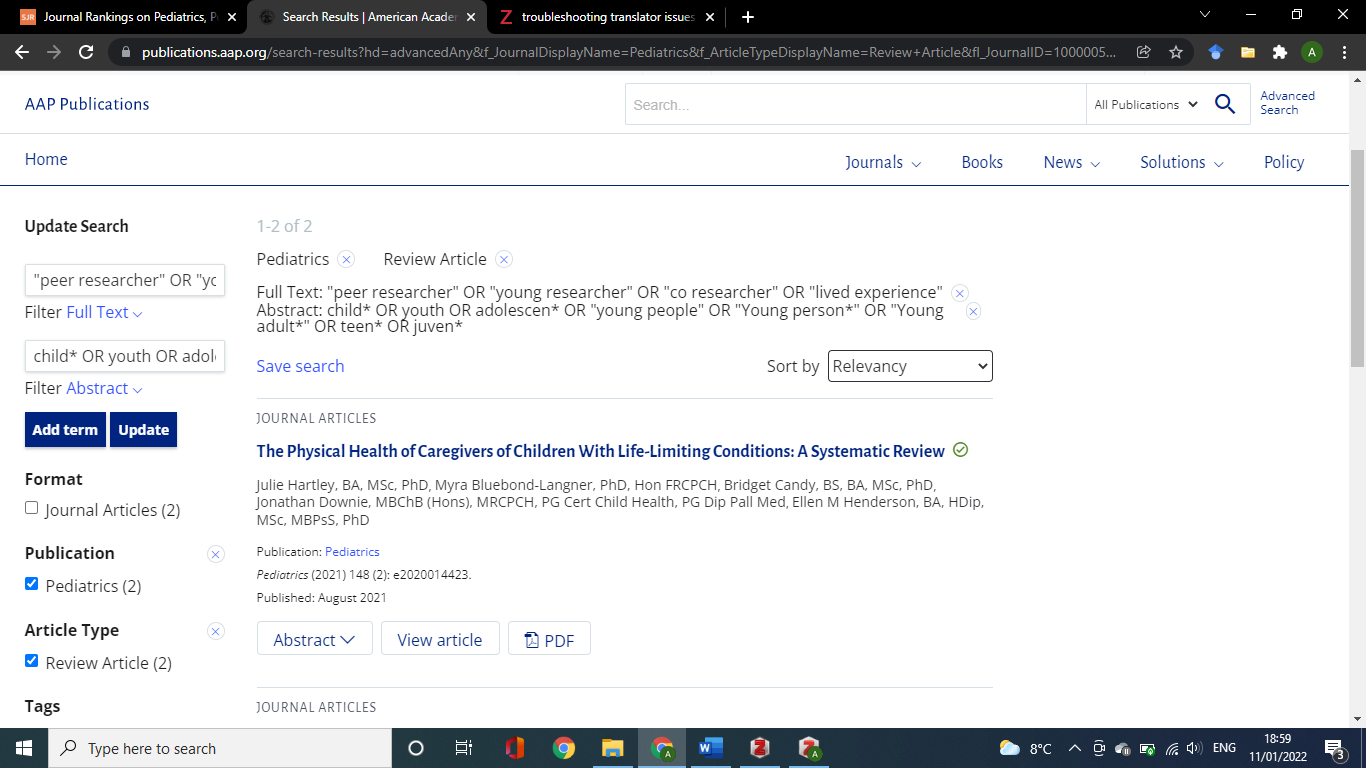 | |
