# Supplementary material for: An umbrella review of reviews on challenges to meaningful adolescent involvement in health research
Source: Health Expect. 2024 Jan 27;27(1):e13980. doi: 10.1111/hex.13980 (PMC10821743; doi:10.1111/hex.13980)
Supplement: Supplementary file 1 — Supporting information. [file HEX-27-e13980-s001.zip › Search record and results/Other sources/Websites of health organizations/Google/Search record for Google.docx]

**Search record for Google**

**Overview**: 20 pages of google searched for youth health organizations

**Date**: 1^st^ December 2021

Total websites identified= 271

Websites excluded= 105

- *Resource guides / resources= 10*
- *No website found/website down=7*
- *Blog/Wikipedia/video/commentary/news=40*
- *Articles=13*
- *Duplicates=10*
- *Course outline=1*
- *List=13*
- *Report=2*
- *Merchandise website=7*
- *Funding call=1*
- *Book=1*

Final number of websites identified from Google search = 166
