# Supplementary material for: An umbrella review of reviews on challenges to meaningful adolescent involvement in health research
Source: Health Expect. 2024 Jan 27;27(1):e13980. doi: 10.1111/hex.13980 (PMC10821743; doi:10.1111/hex.13980)
Supplement: Supplementary file 1 — Supporting information. [file HEX-27-e13980-s001.zip › Search record and results/Other sources/Websites of health organizations/Google search for 137 LMICs/Search record for 137 LMICs.docx]

**Search record for 137 LMICs**

**Overview**: Searched google for ‘youth health organizations’ combined with country name for each of the 137 LMICs and searched the website of the organization which was the top google result for each country (1 per country)

**Date**: 8^th^ – 9^th^ December 2021

Total websites identified= 137

Websites excluded= 0

Final number of websites identified from Google search = 137
