# Supplementary material for: An umbrella review of reviews on challenges to meaningful adolescent involvement in health research
Source: Health Expect. 2024 Jan 27;27(1):e13980. doi: 10.1111/hex.13980 (PMC10821743; doi:10.1111/hex.13980)
Supplement: Supplementary file 1 — Supporting information. [file HEX-27-e13980-s001.zip › Search record and results/Other sources/Websites of health organizations/Mental Health Innovation Network Database/Search record for MHIN.docx]

**Search record for MHIN**

**Overview**: Mental Health Innovation Network Database searched for youth health organizations

**Date**: 7^th^ December 2021

**Filter**: Child and adolescents

Total websites identified= 243

Websites excluded= 0

Final number of websites identified from Google search = 243
