# Supplementary material for: An umbrella review of reviews on challenges to meaningful adolescent involvement in health research
Source: Health Expect. 2024 Jan 27;27(1):e13980. doi: 10.1111/hex.13980 (PMC10821743; doi:10.1111/hex.13980)
Supplement: Supplementary file 1 — Supporting information. [file HEX-27-e13980-s001.zip › Search record and results/Other sources/Websites of health organizations/Mental Health Innovation Network Database/Screenshots- MHIN.docx]

| # | **MHIN database results screenshots** |
| --- | --- |
| 1 |  |
| 1 | ` |
| 1 |  |
|  |  |
|  |  |
| 2 |  |
|  |  |
|  |  |
| 3 |  |
|  |  |
|  |  |
| 4 |  |
|  |  |
|  |  |
| 5 |  |
|  |  |
|  |  |
| 6 |  |
|  |  |
|  |  |
| 7 |  |
|  |  |
|  |  |
| 8 |  |
|  |  |
|  |  |
| 9 |  |
