# Supplementary material for: An umbrella review of reviews on challenges to meaningful adolescent involvement in health research
Source: Health Expect. 2024 Jan 27;27(1):e13980. doi: 10.1111/hex.13980 (PMC10821743; doi:10.1111/hex.13980)
Supplement: Supplementary file 1 — Supporting information. [file HEX-27-e13980-s001.zip › Search record and results/Other sources/Websites of health organizations/Compiled list of websites, search strategies and results/Flow chart.docx]

**Flow chart for websites of health organizations searched for reviews on youth involvement**

Excluded (from google results) =105

- *Resource guides / resources= 10*
- *No website found/website down=7*
- *Blog/Wikipedia/video/commentary/news=40*
- *Articles=13*
- *Duplicates=10*
- *Course outline=1*
- *List=13*
- *Report=2*
- *Merchandise website=7*
- *Funding call=1*
- *Book=1*

**Organizations identified= 567**

- Up to 20 pages of Google search=271 (Final 166)
- MHIN database=243
- Country-specific Google search=137
- Websites known to authors=21

Removed=95

- *Duplicates = 27*
- *Website down=38*
- *Could not find website=24*
- *Website not in English=3*
- *Access denied=2*
- *No longer available= 1*

Relevant Reviews identified =11

Organizational websites searched for reviews and guidelines =472
