# Supplementary material for: An umbrella review of reviews on challenges to meaningful adolescent involvement in health research
Source: Health Expect. 2024 Jan 27;27(1):e13980. doi: 10.1111/hex.13980 (PMC10821743; doi:10.1111/hex.13980)
Supplement: Supplementary file 1 — Supporting information. [file HEX-27-e13980-s001.zip › Search record and results/Other sources/Websites of health organizations/Compiled list of websites, search strategies and results/Search strategy for websites.docx]

**Search strings used to search websites**

1. “Youth involvement” OR Involve OR “Youth engagement” OR engage OR Stakeholder OR Participatory OR “public and patient involvement” OR Advisory OR “co-production” OR “Human centered design” OR “peer researcher” OR co-researcher OR “young researcher” OR “lived experience” OR Guide OR Handbook OR Framework
2. “Youth involvement” Involve “Youth engagement” engage Stakeholder Participatory “public and patient involvement” Advisory “co-production” “Human centered design” “peer researcher” co-researcher “young researcher” “lived experience” Guide OR Handbook OR Framework
3. Youth involvement=
   Youth engagement=
   Stakeholder =

Participatory=
public and patient involvement=
Advisory =
co-production =
Human centered design =
peer researcher =
co-researcher =
young researcher =
lived experience=
Guide=
Handbook=
Framework=

1. Youth involvement OR Youth engagement=

Stakeholder OR participatory OR advisory=

public and patient involvement=

co-production OR human-centered design=

peer researcher OR young researcher OR co-researcher=

guide OR handbook OR framework

1. **Google**

“Youth involvement” OR “Youth engagement” OR Stakeholder OR Participatory OR “public and patient involvement” OR Advisory OR “co-production” OR “Human centered design” OR “peer researcher” OR co-researcher OR “young researcher” OR “lived experience” site:wellcome.org
