# Supplementary figures and images for: An umbrella review of reviews on challenges to meaningful adolescent involvement in health research
Source: Health Expect. 2024 Jan 27;27(1):e13980. doi: 10.1111/hex.13980 (PMC10821743; doi:10.1111/hex.13980)

**Types of included reviews**

**
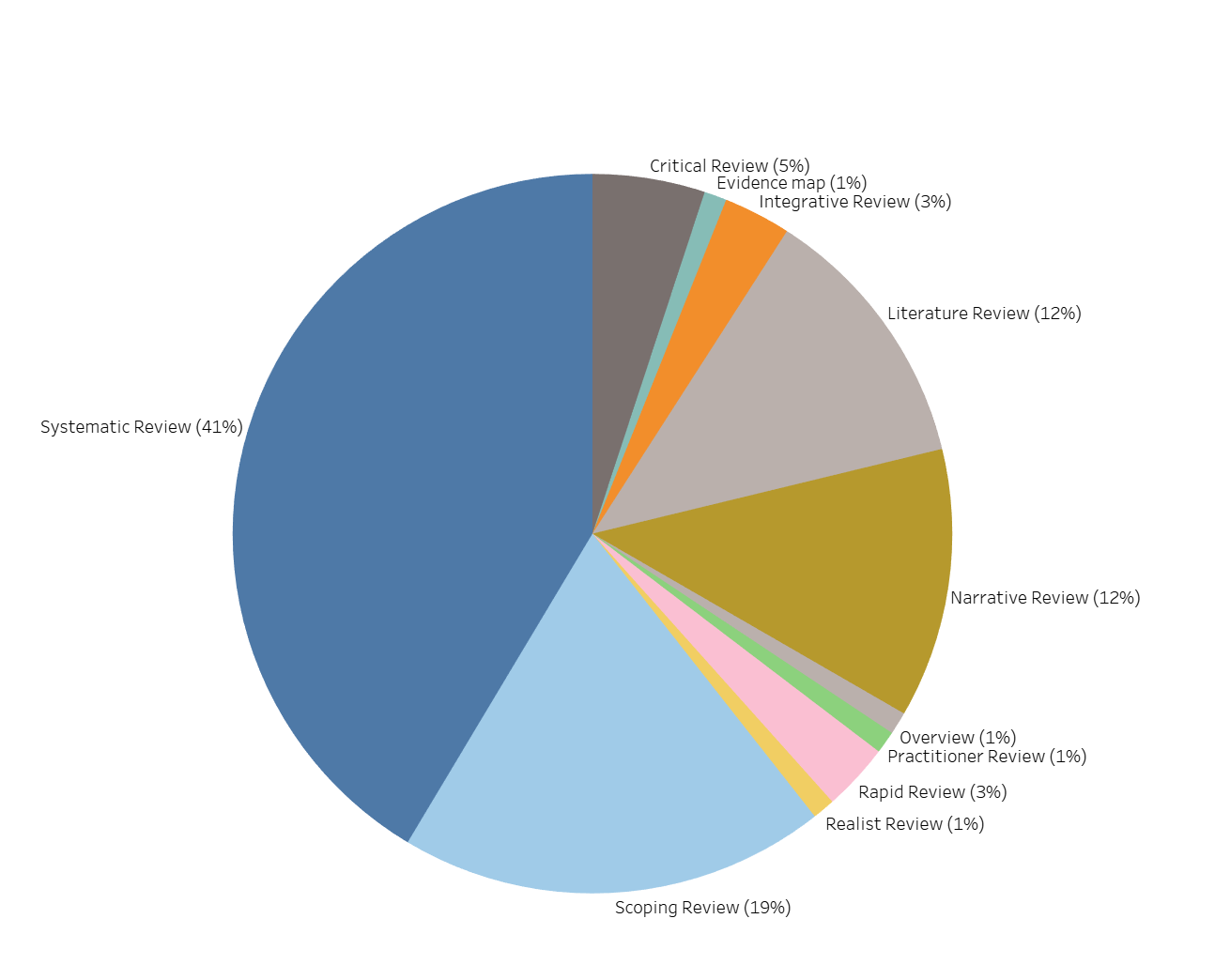
**

Supplement: Supplementary file 1 — Supporting information. [file HEX-27-e13980-s001.zip › Results/Types of included reviews.docx]

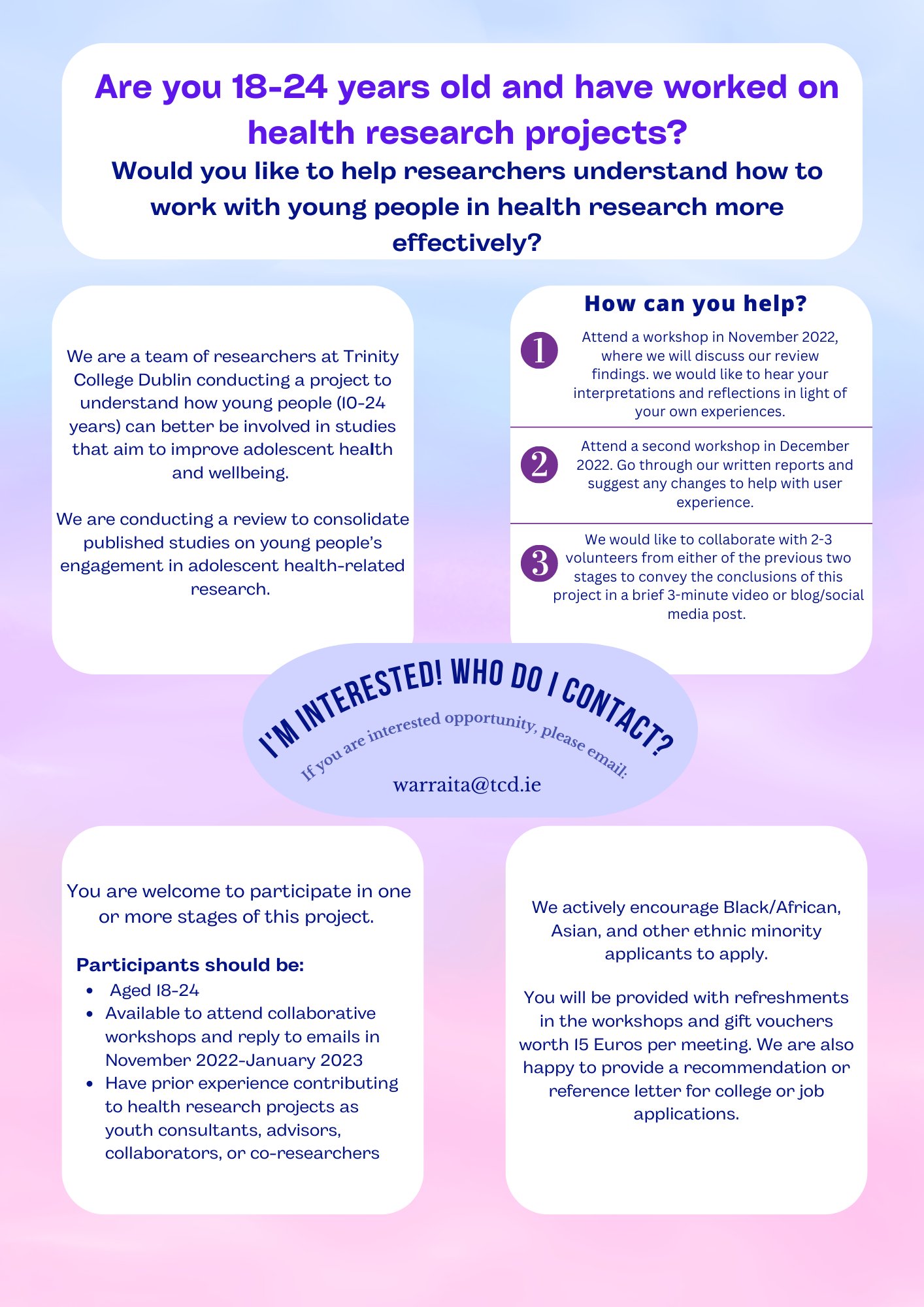

Supplement: Supplementary file 1 — Supporting information. [file HEX-27-e13980-s001.zip › Participatory workshops/Workshop 1/Leaflet.jpeg]

| Country wise list |
| --- |
| 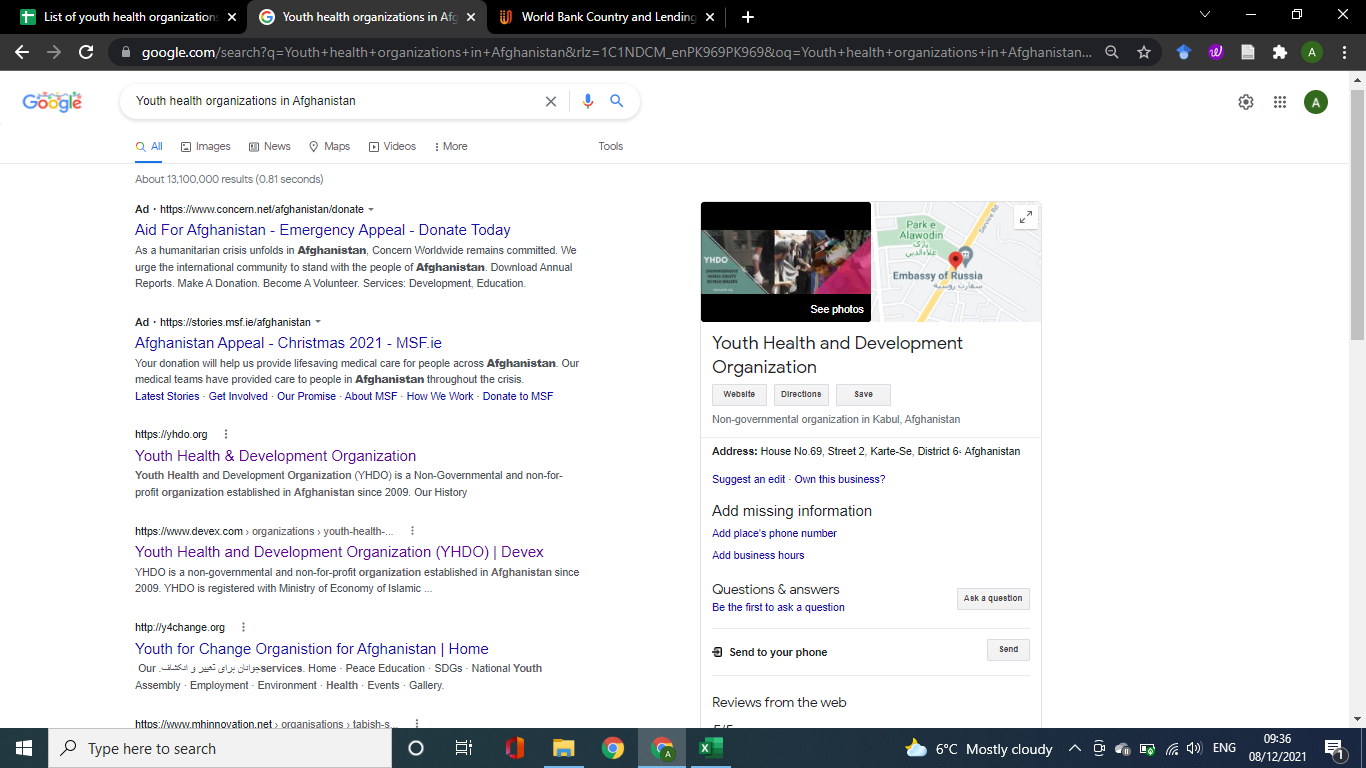 |
| 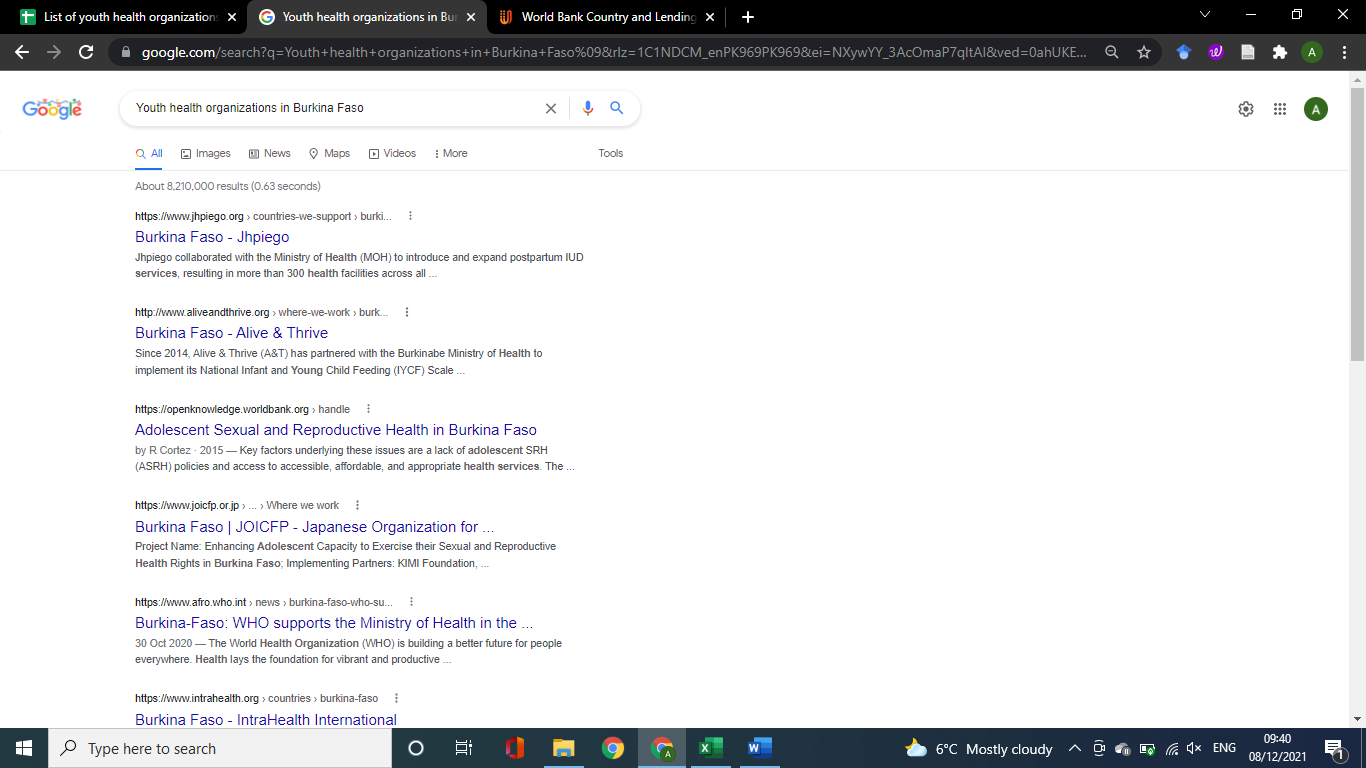 |
| 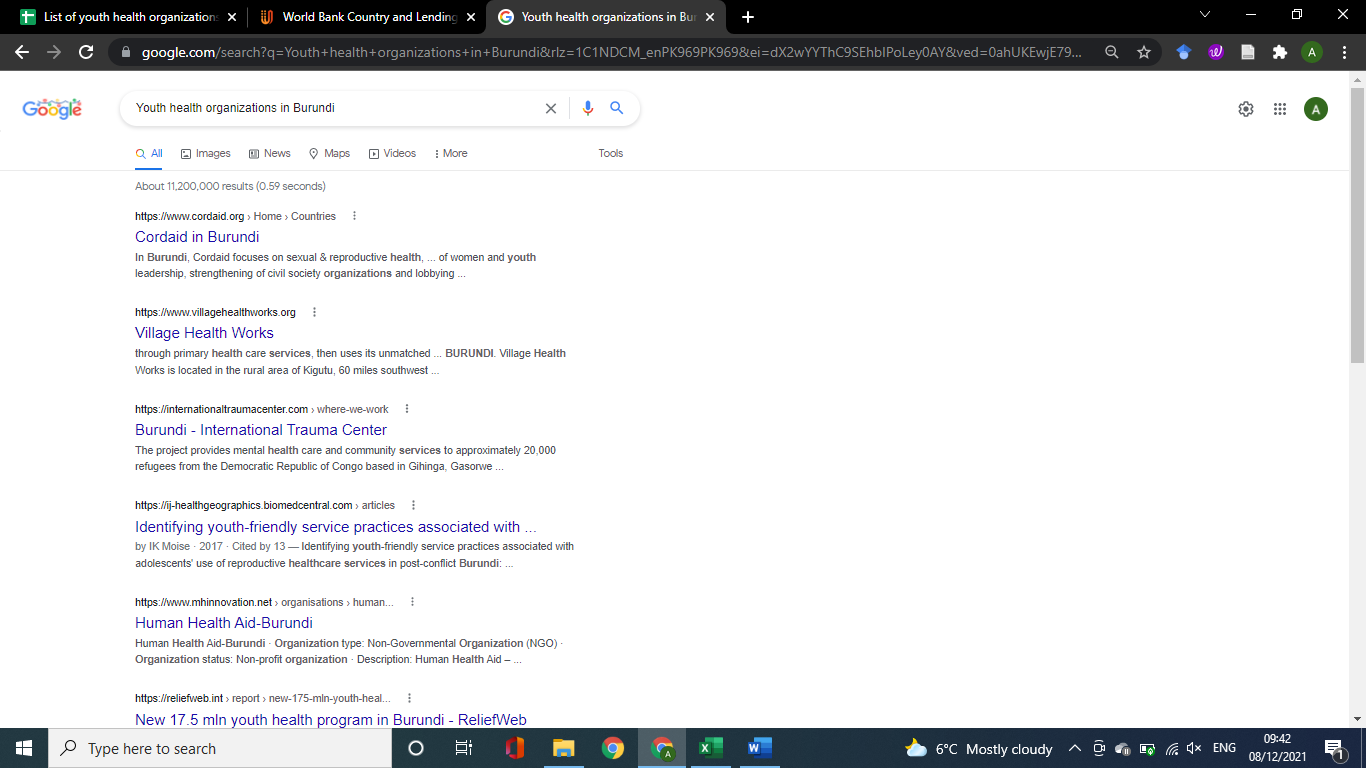 |
| 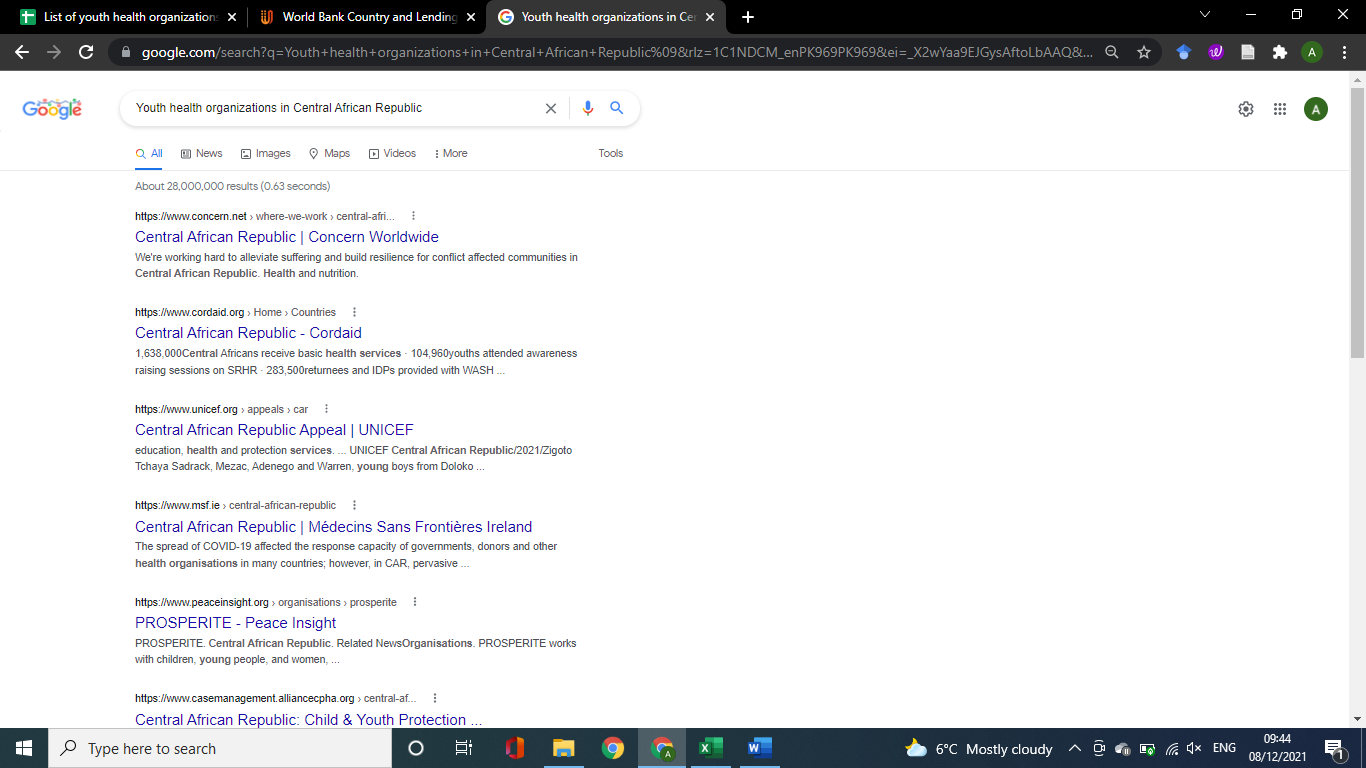 |
| 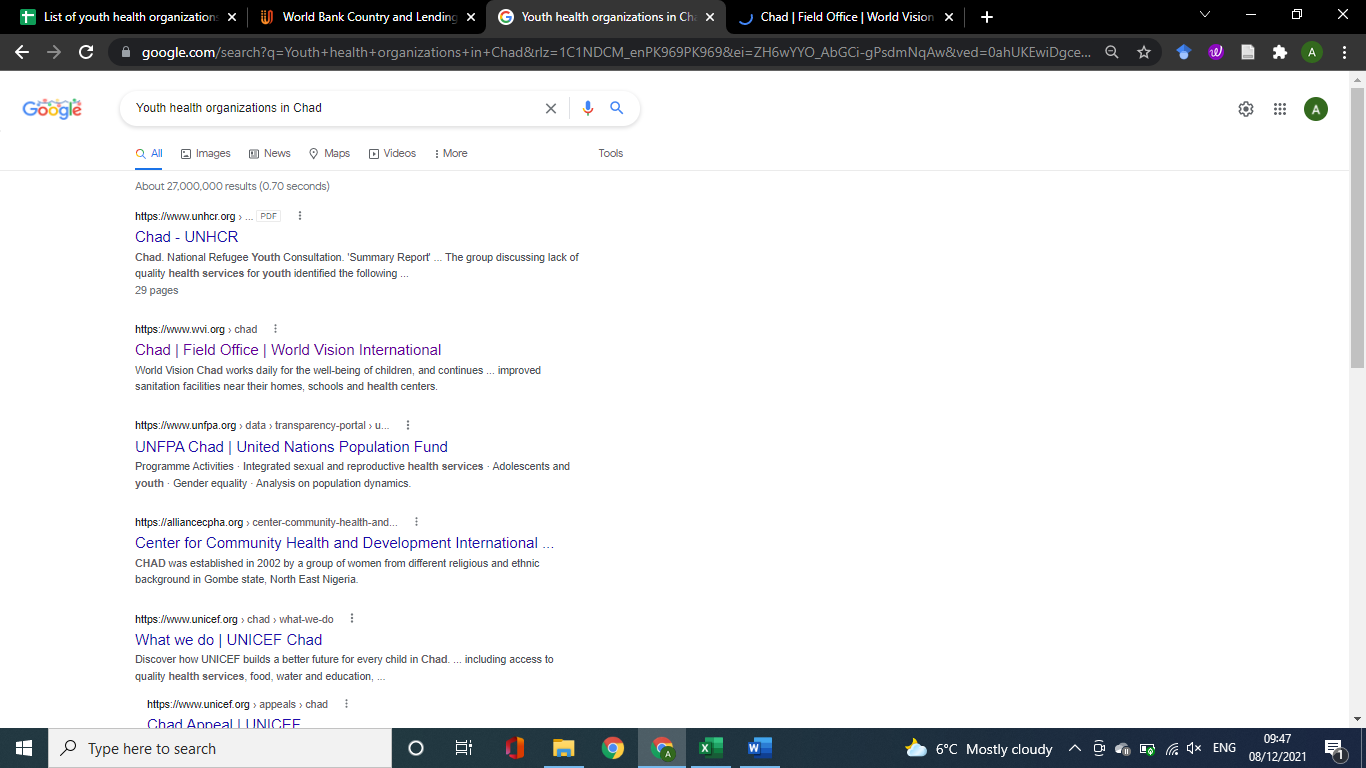 |
| 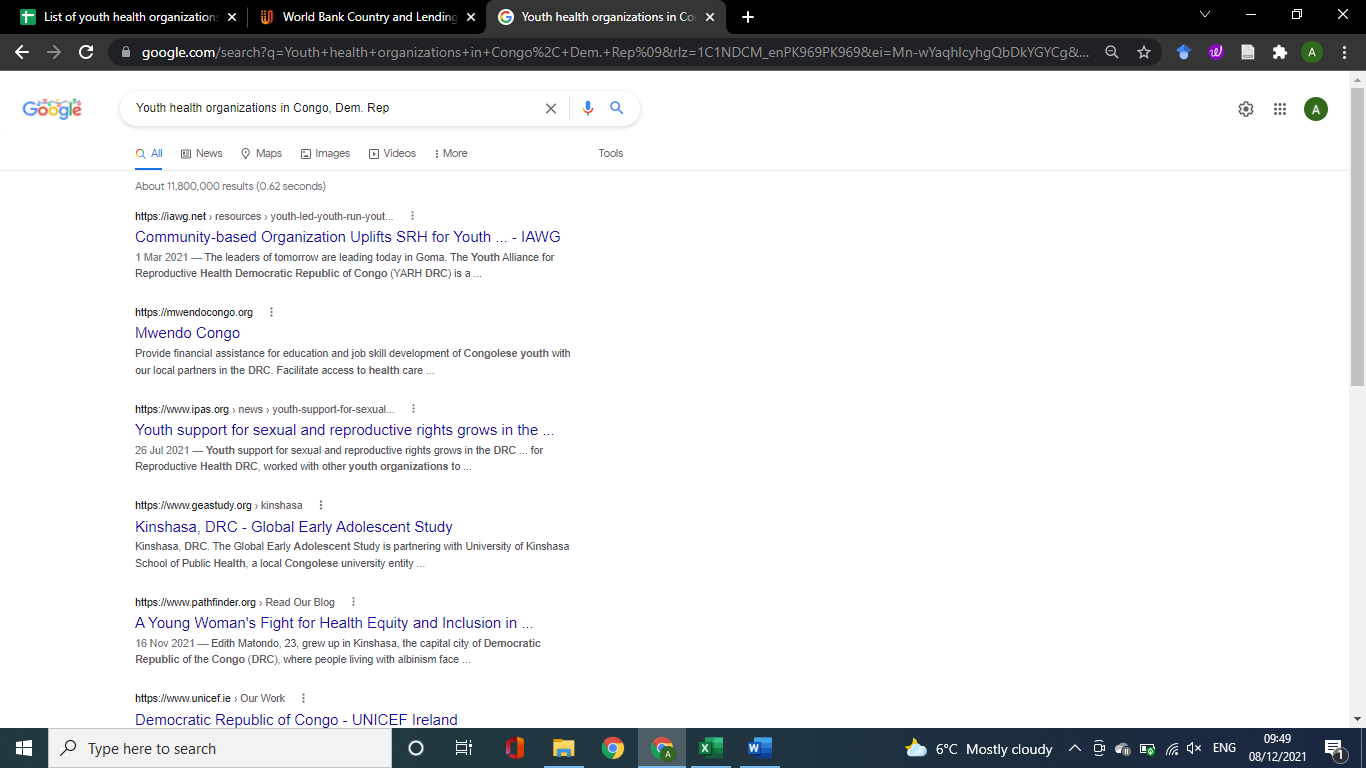 |
| 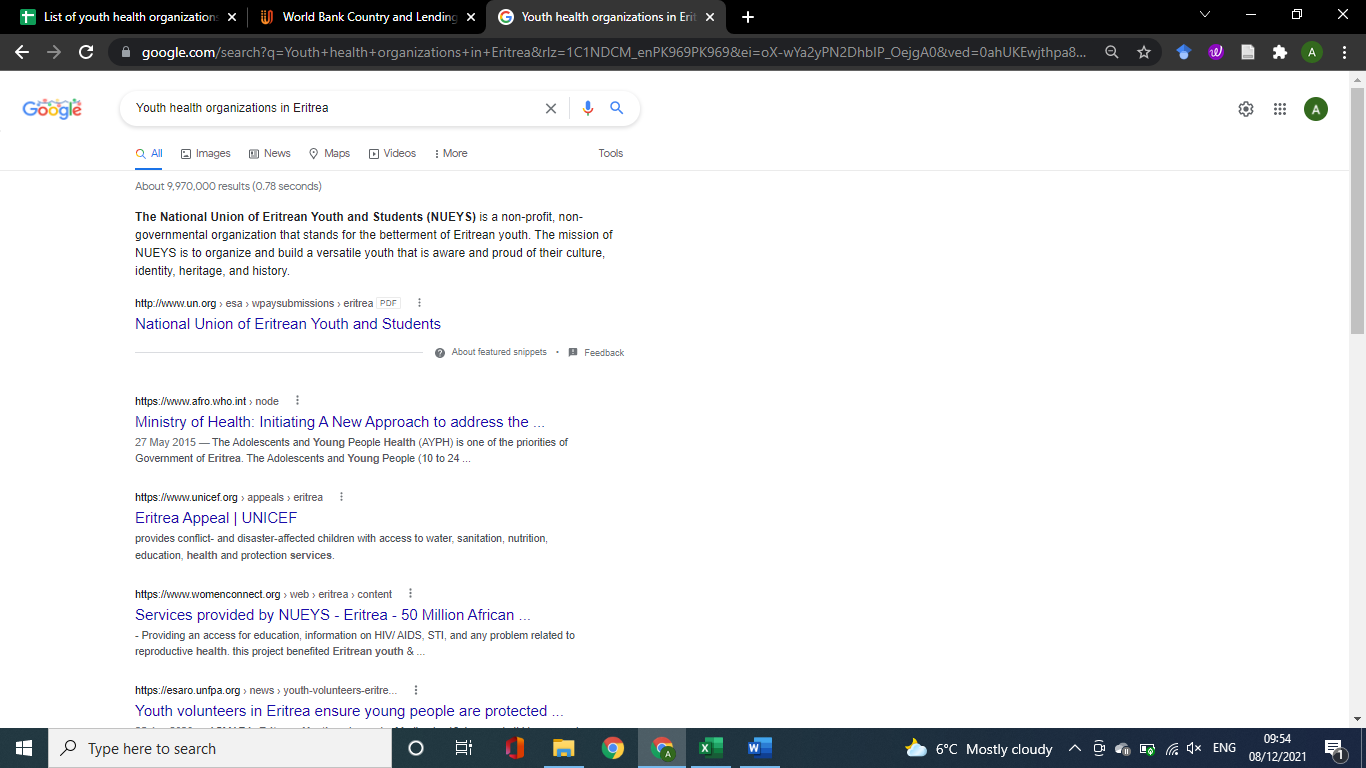 |
| 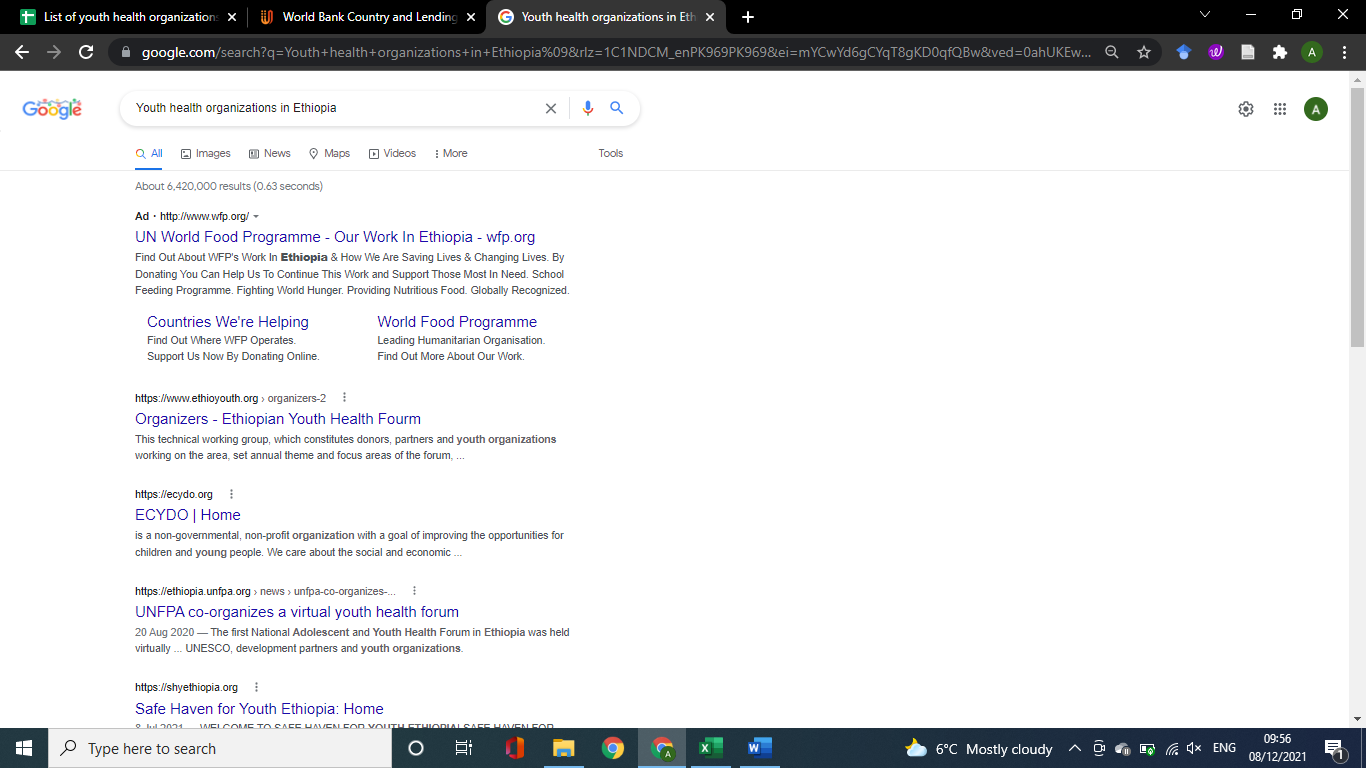 |
| 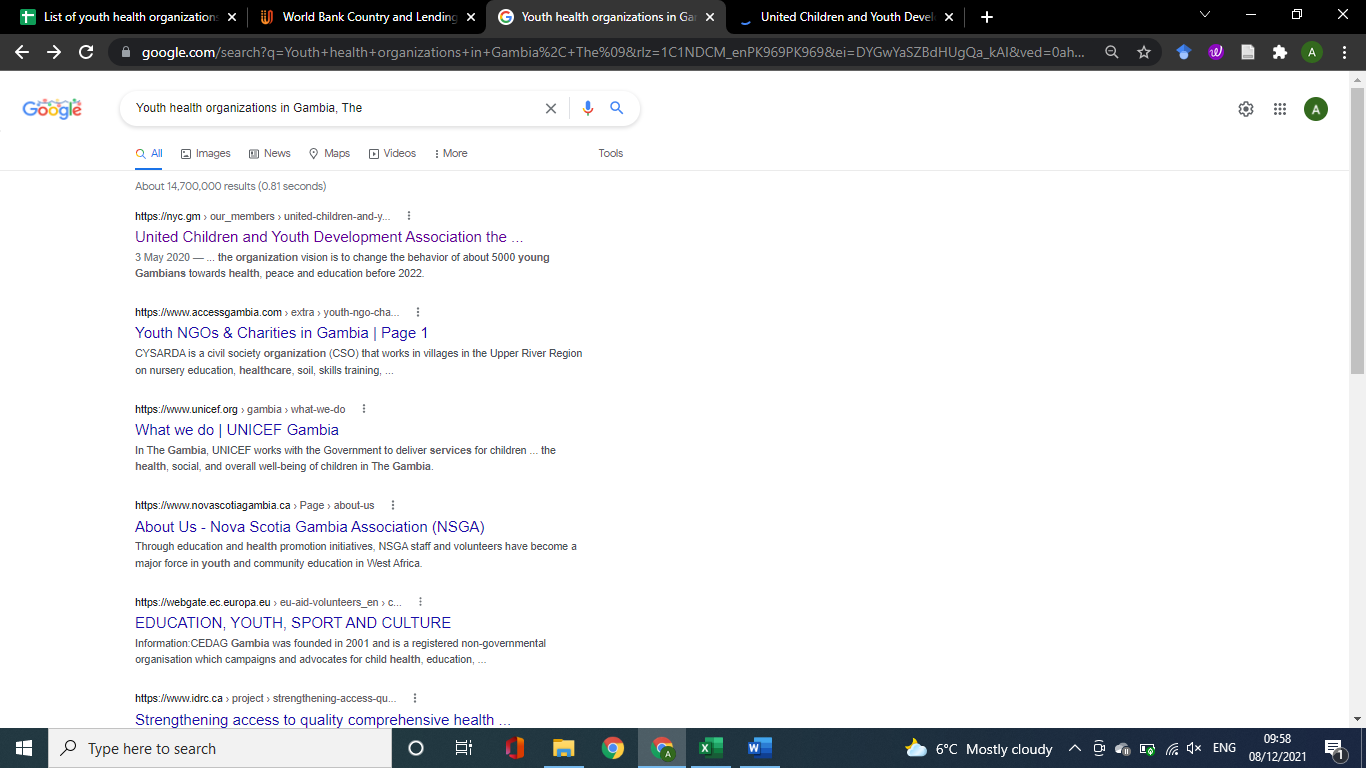 |
| 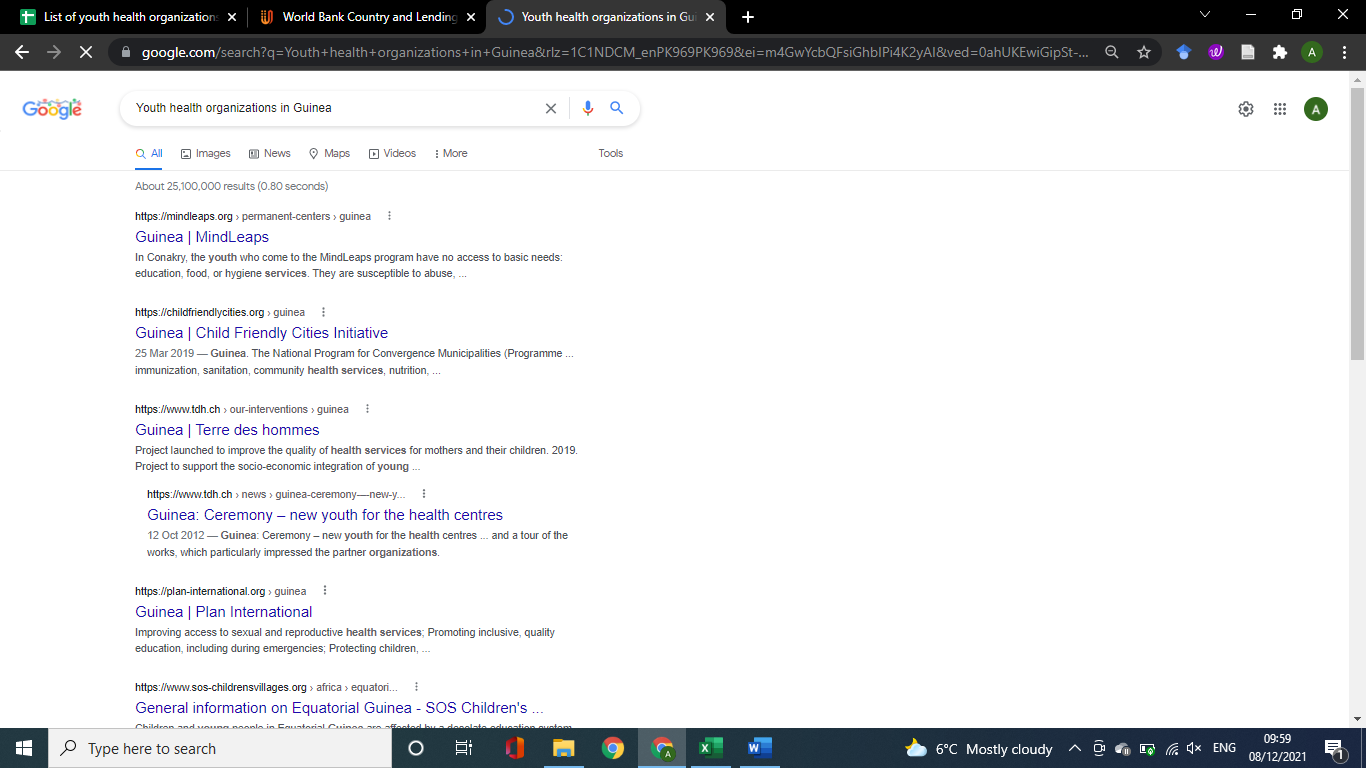 |
| 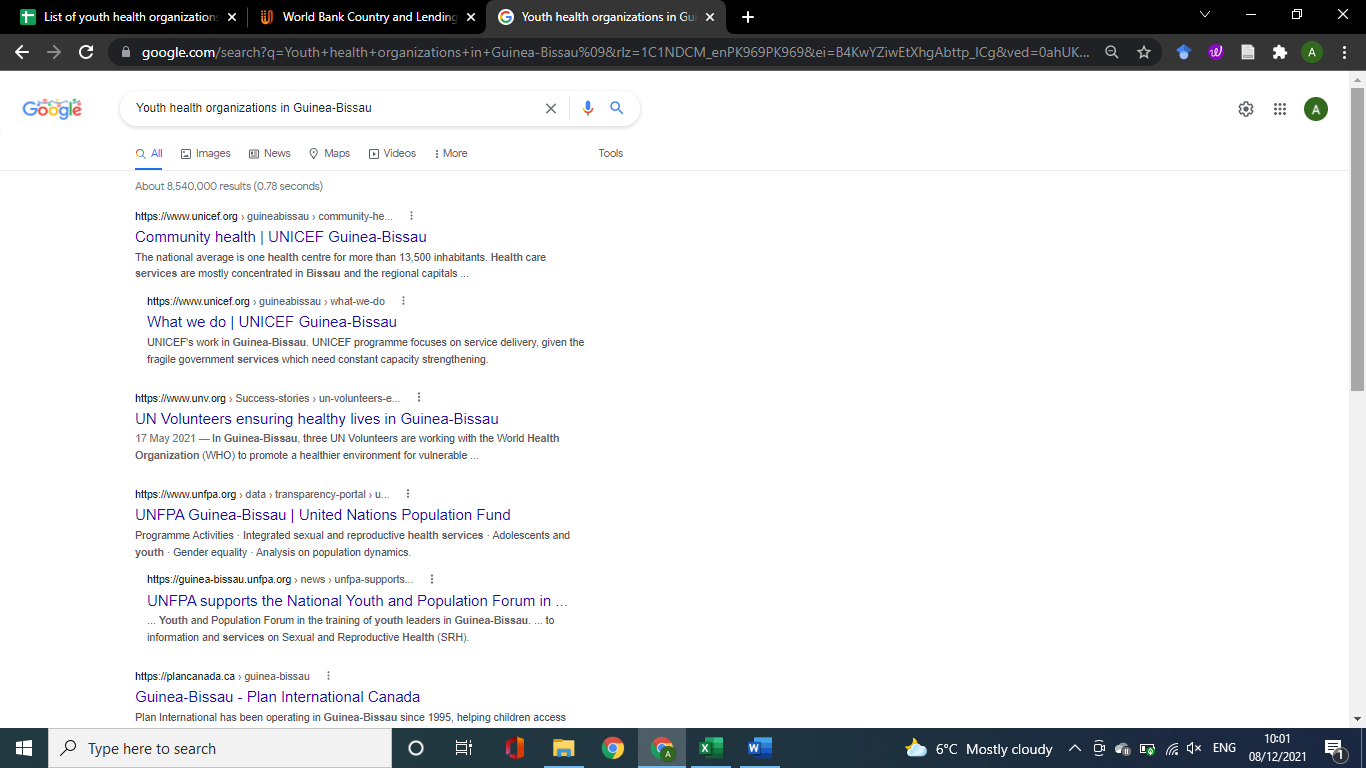 |
| 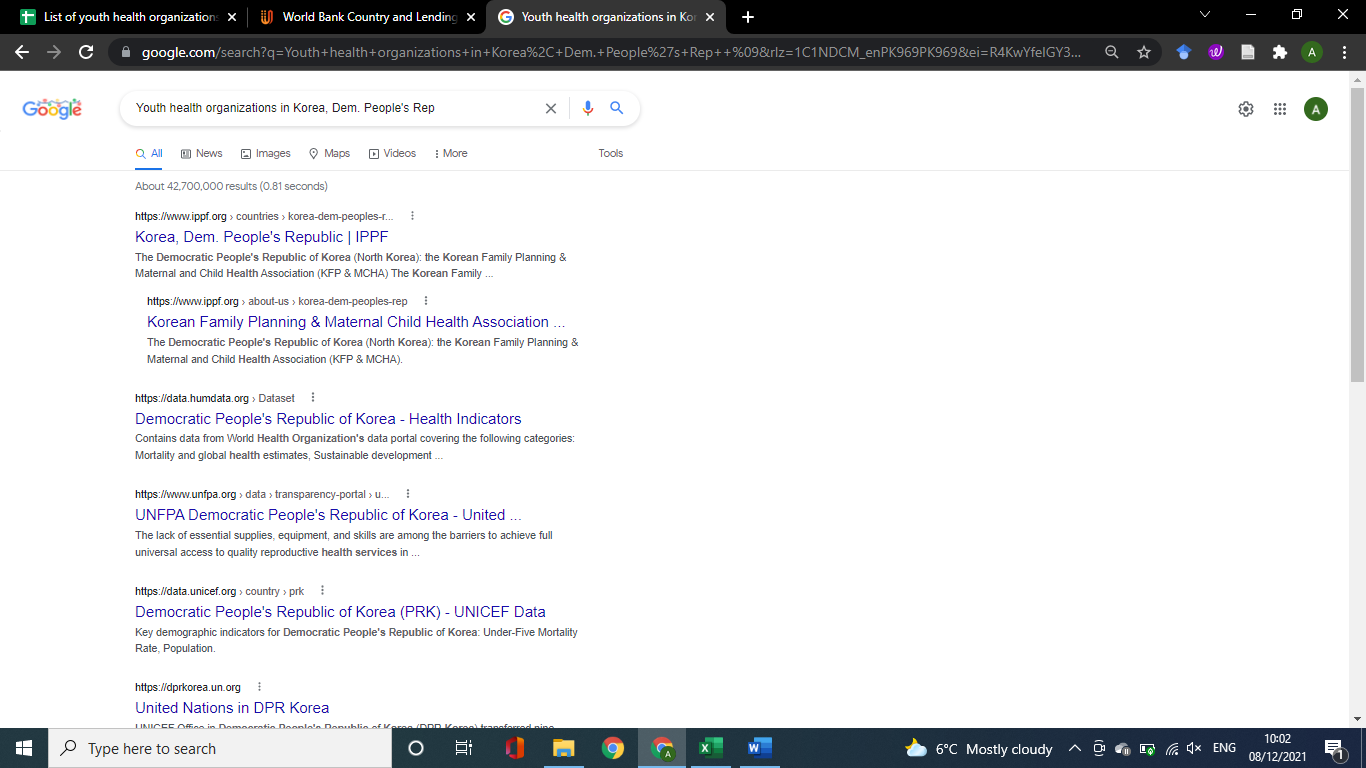 |
| 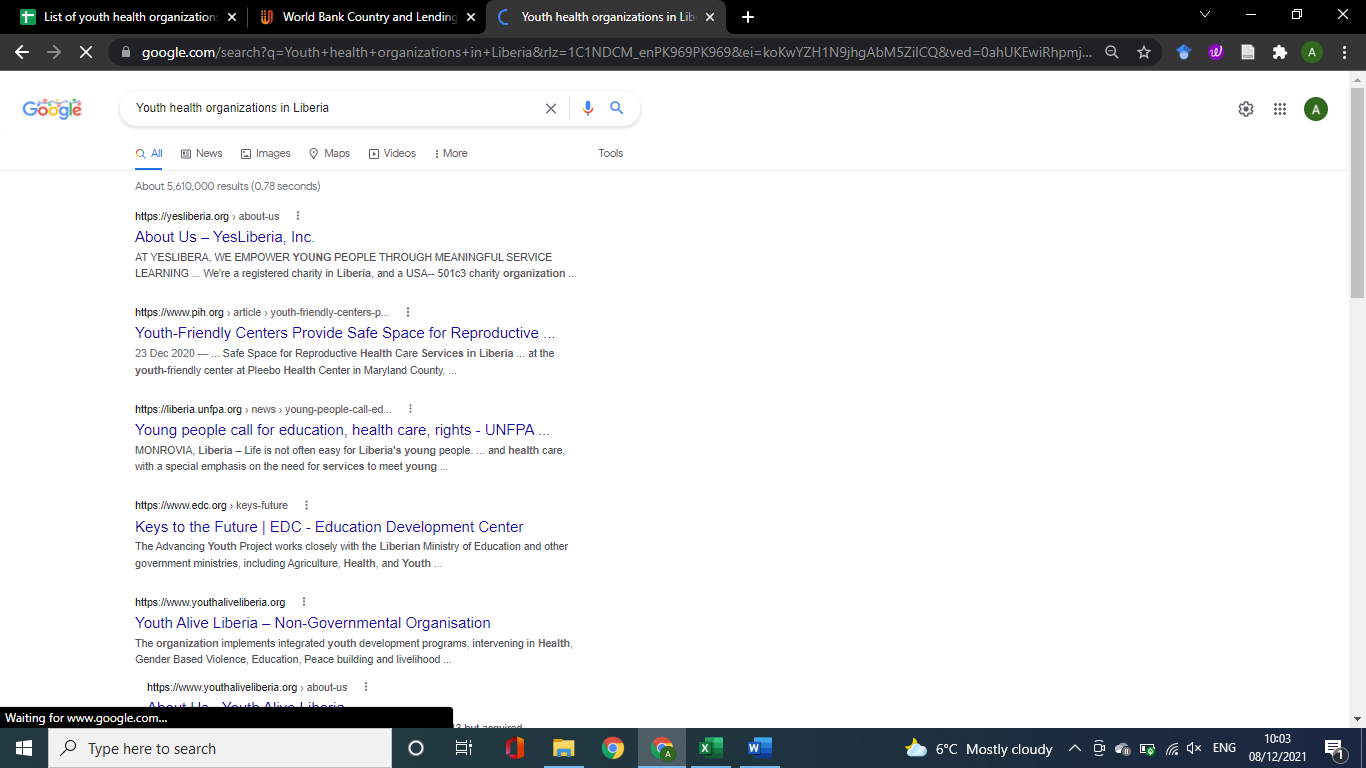 |
| 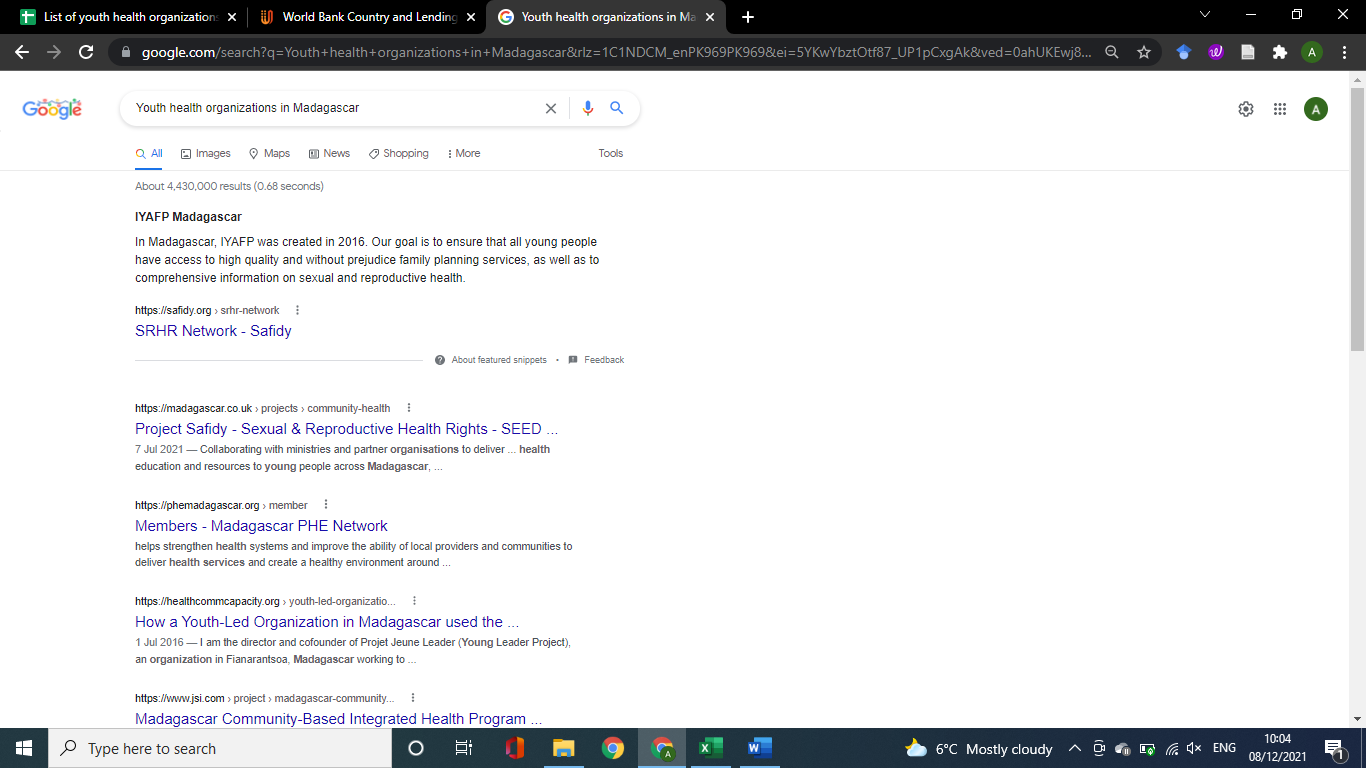 |
| 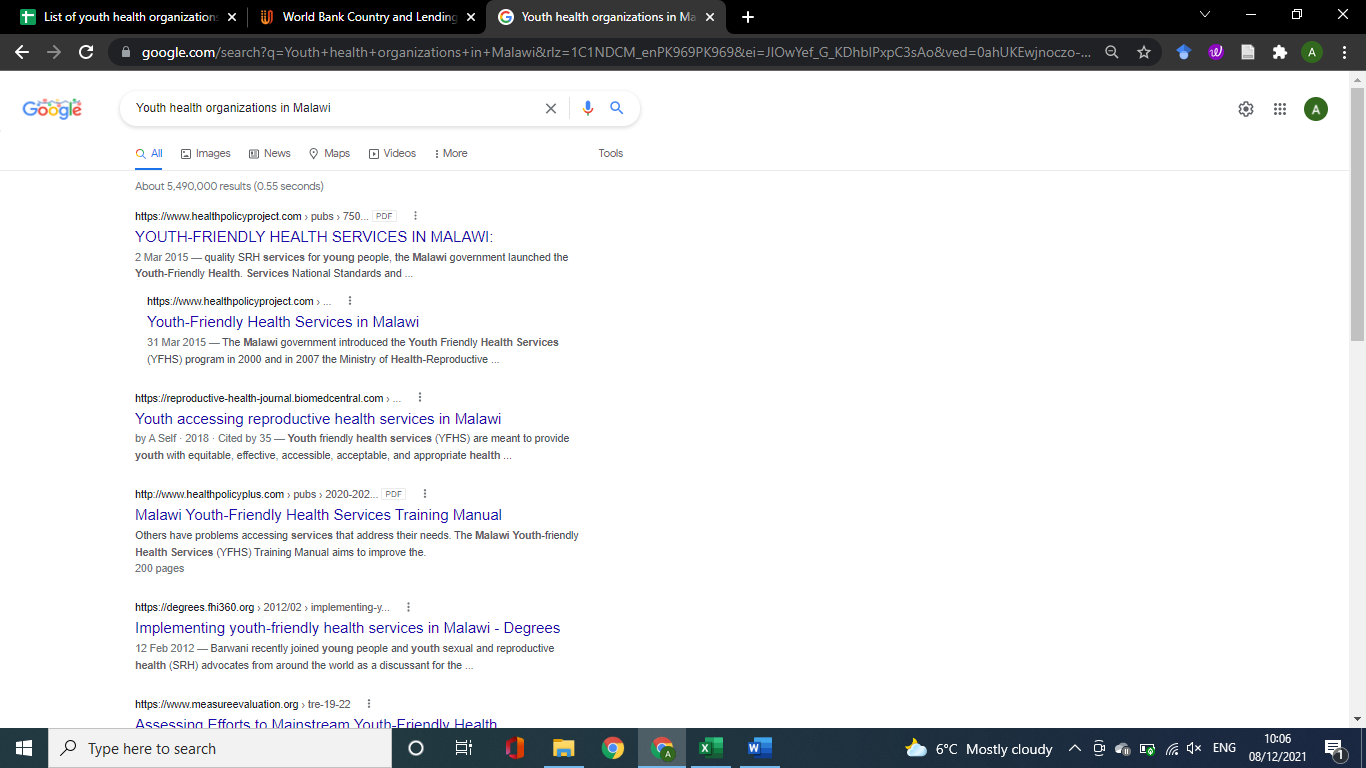 |
| 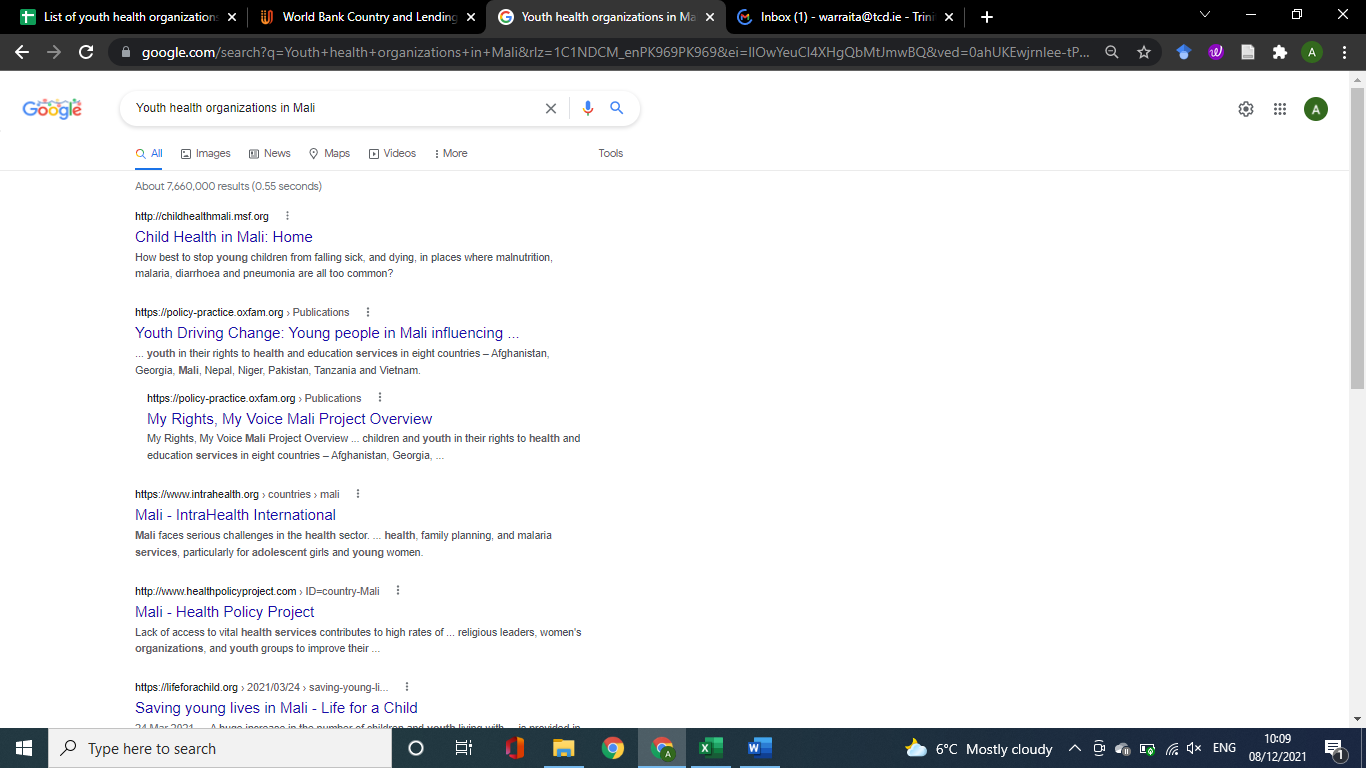 |
| 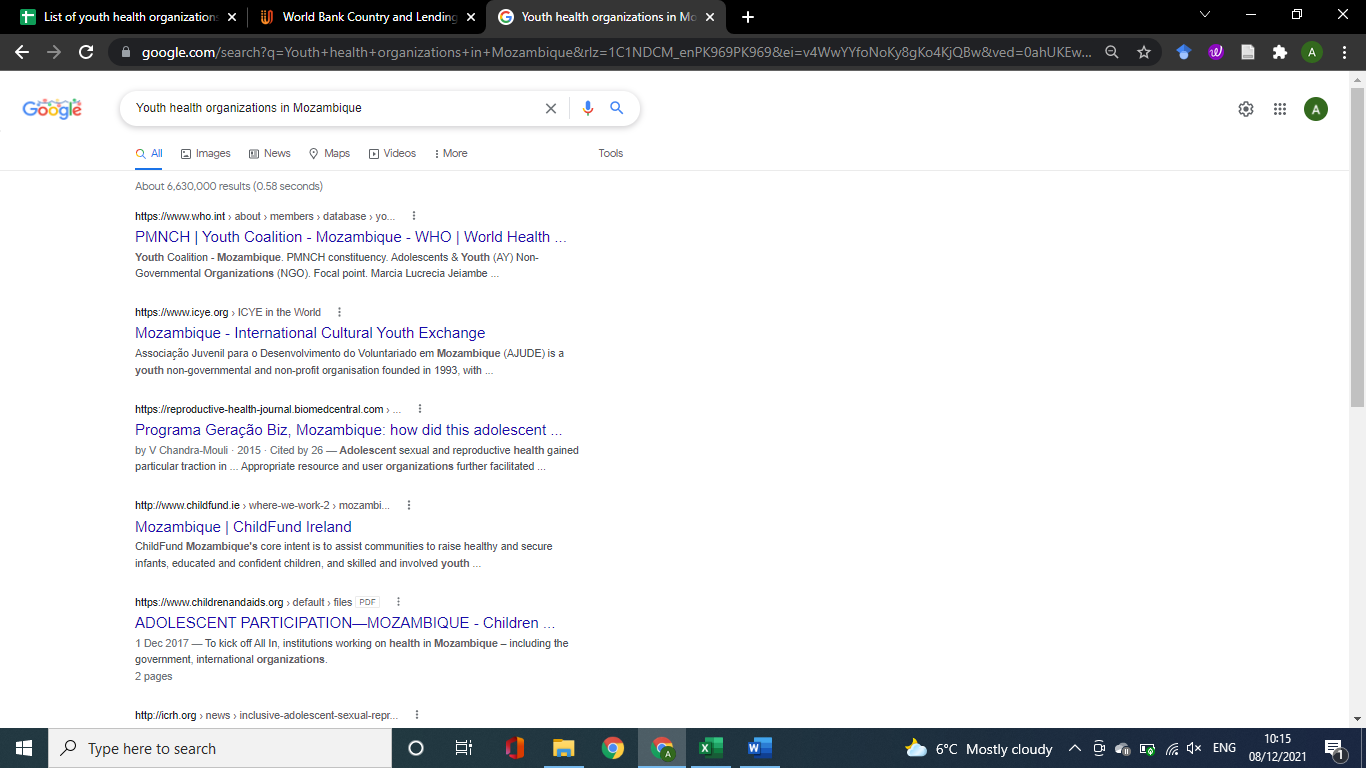 |
| 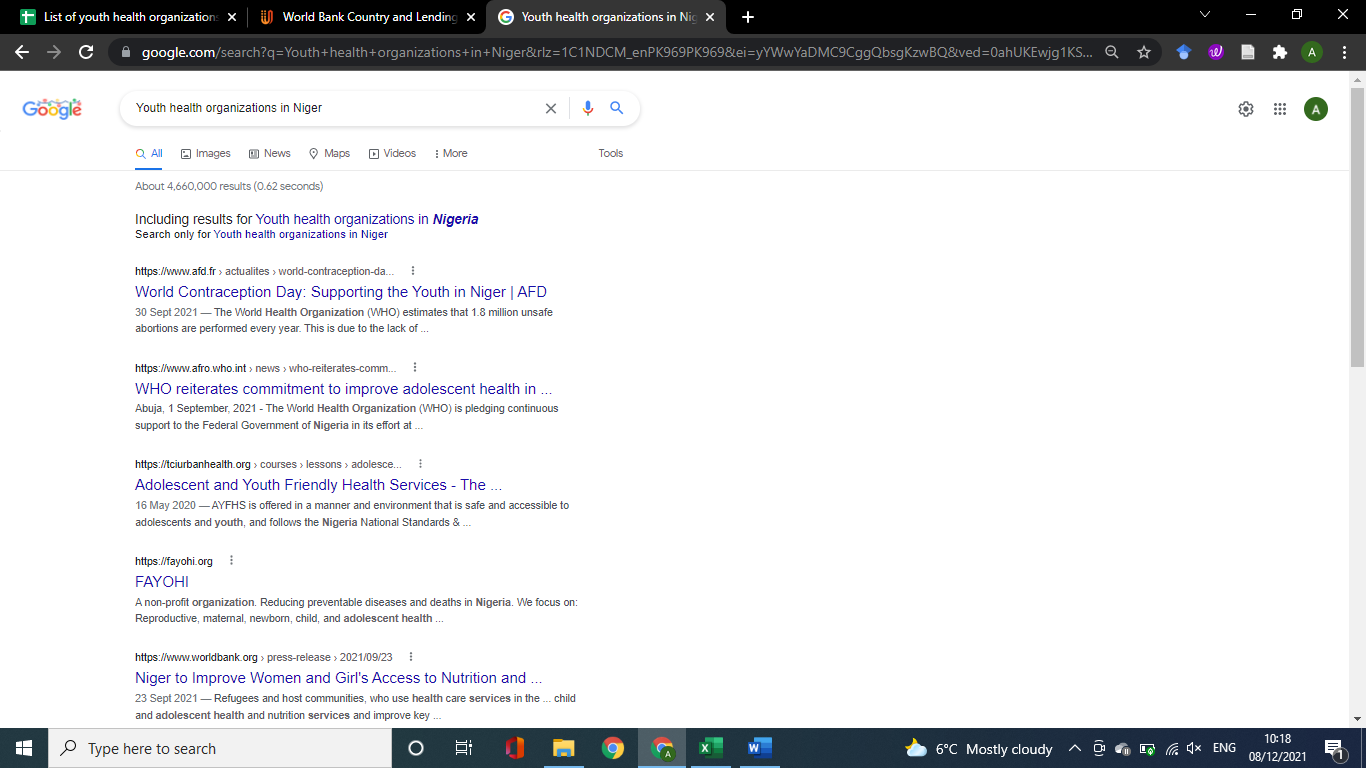 |
| 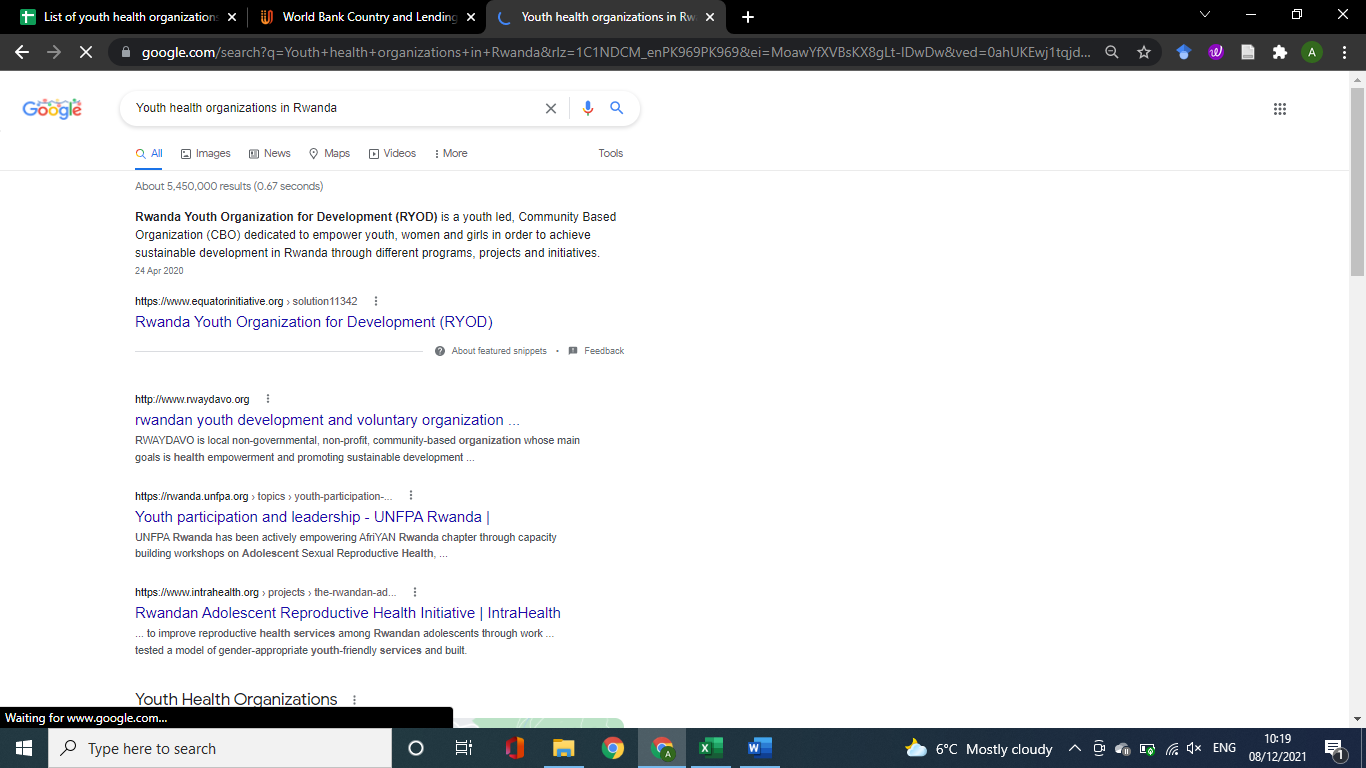 |
| 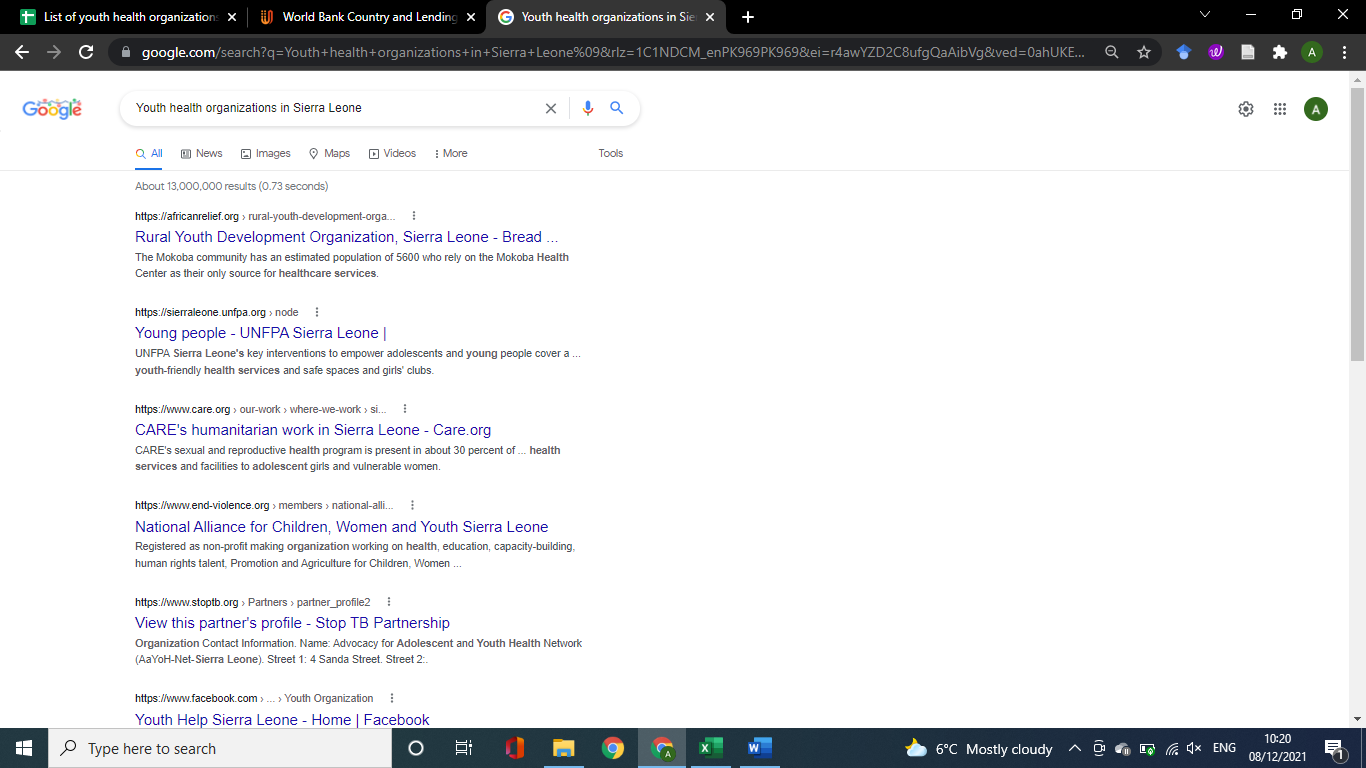 |
| 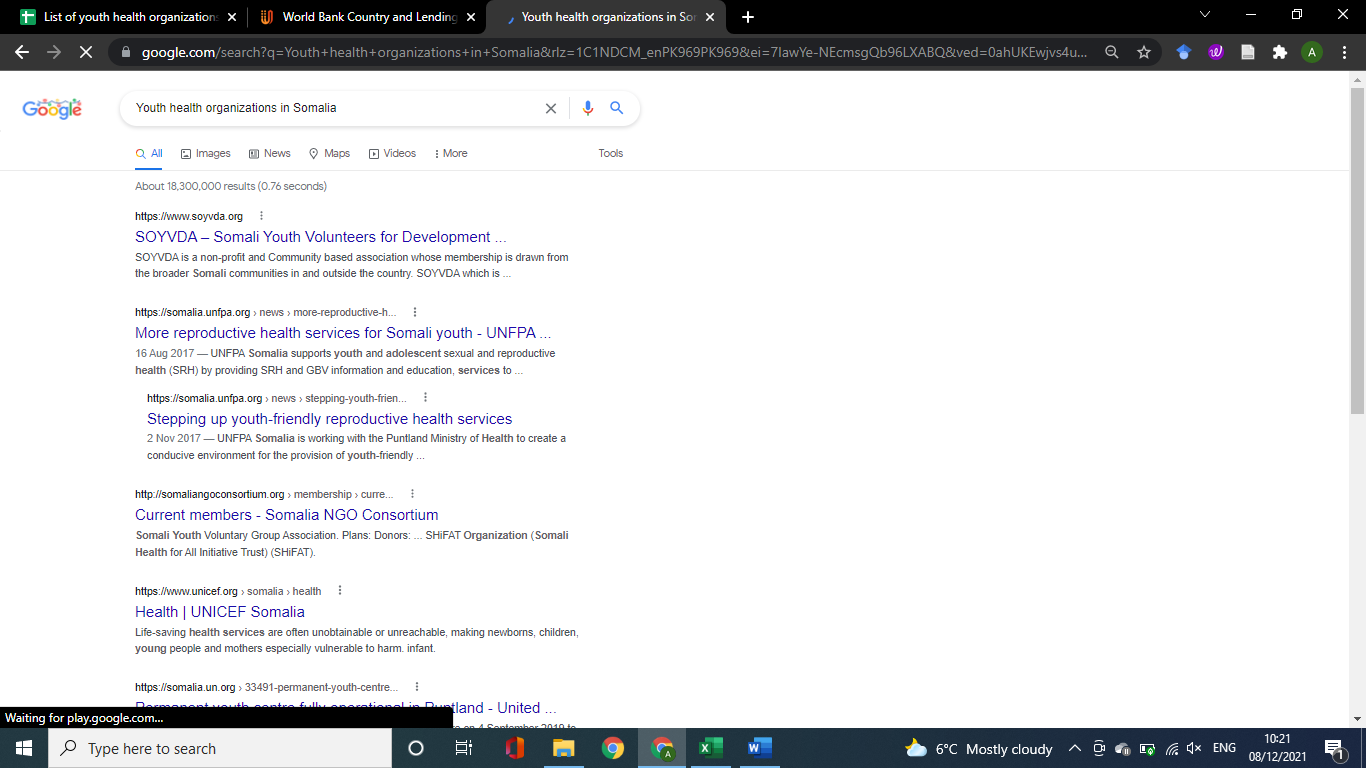 |
| 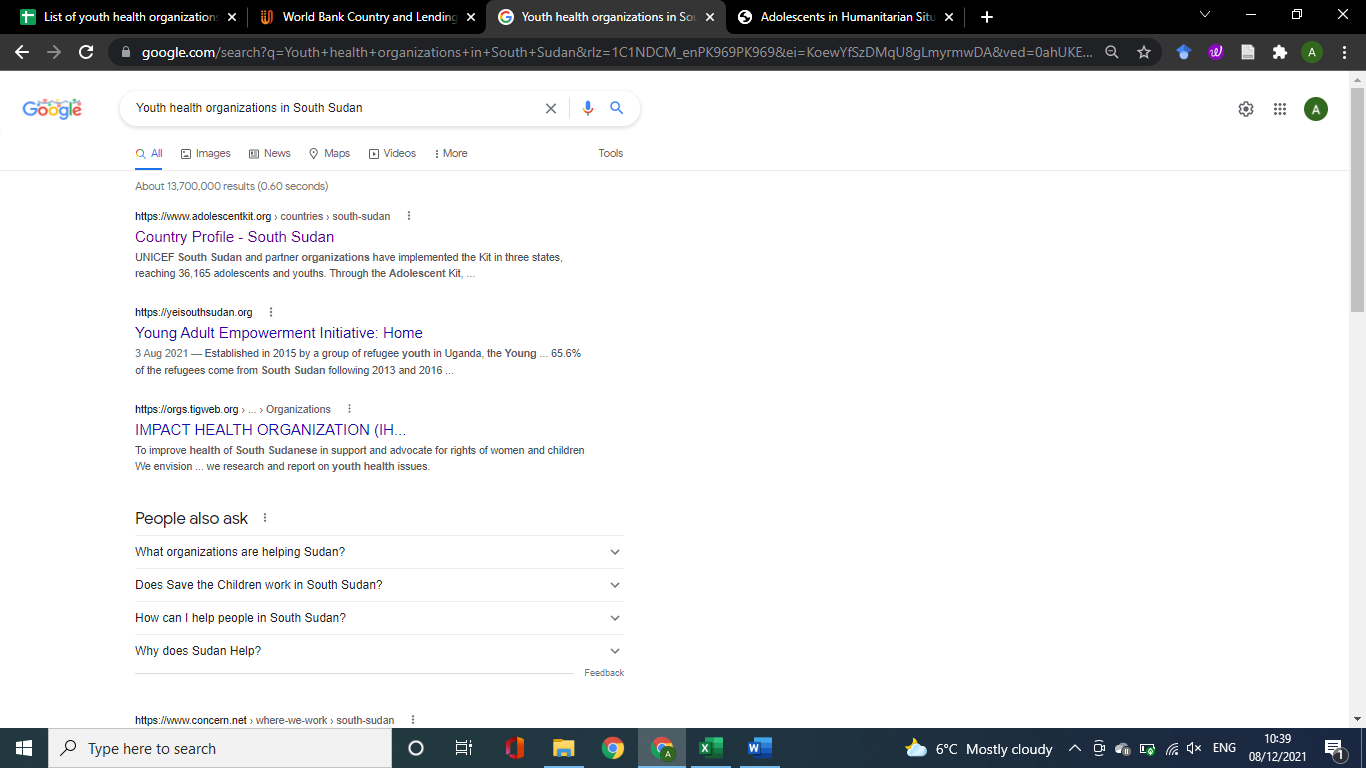 |
| 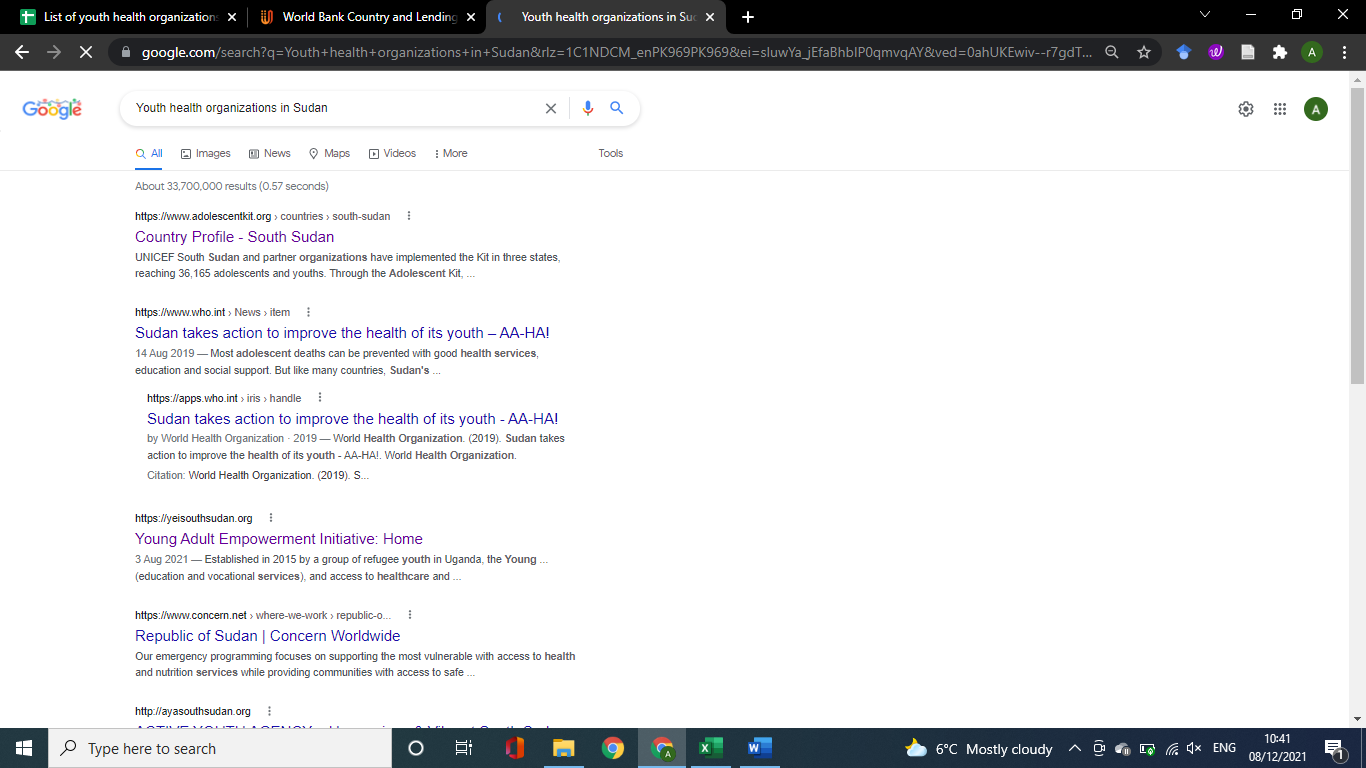 |
| 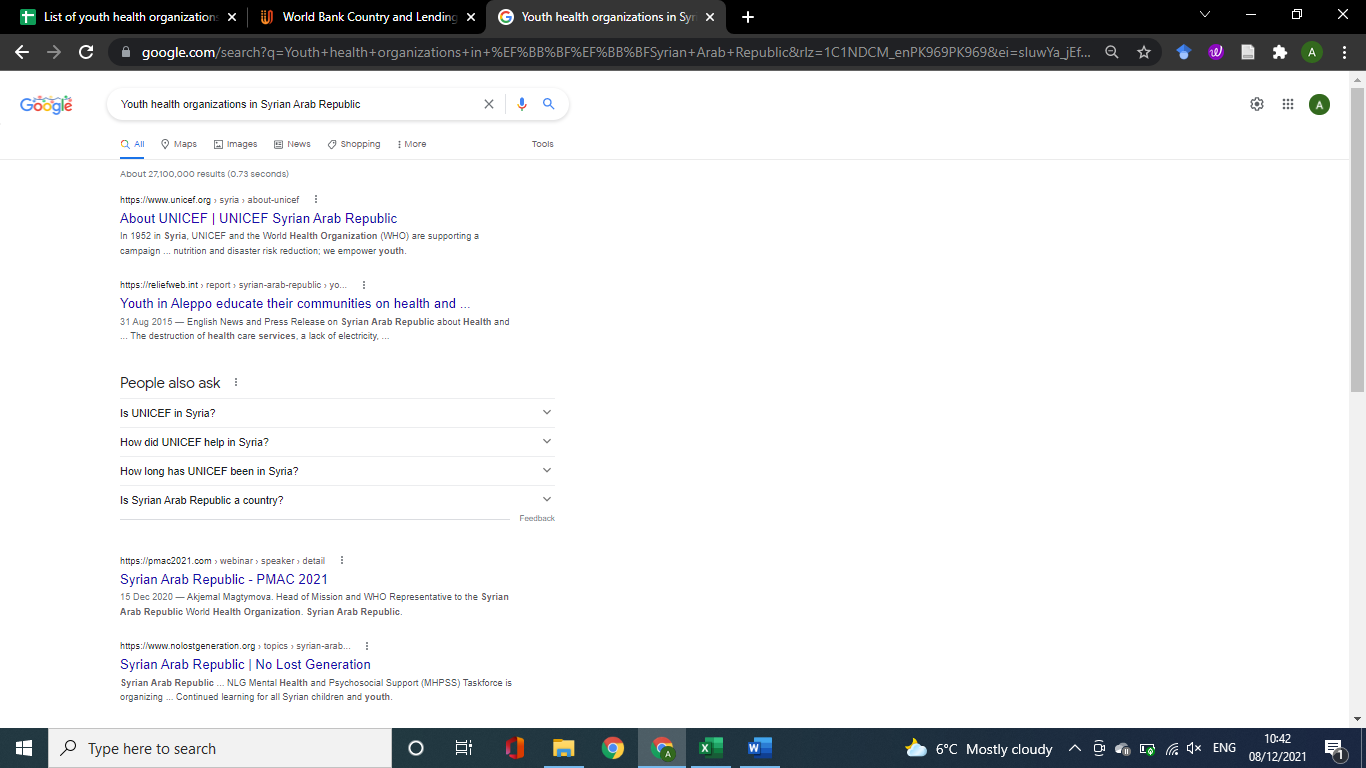 |
| 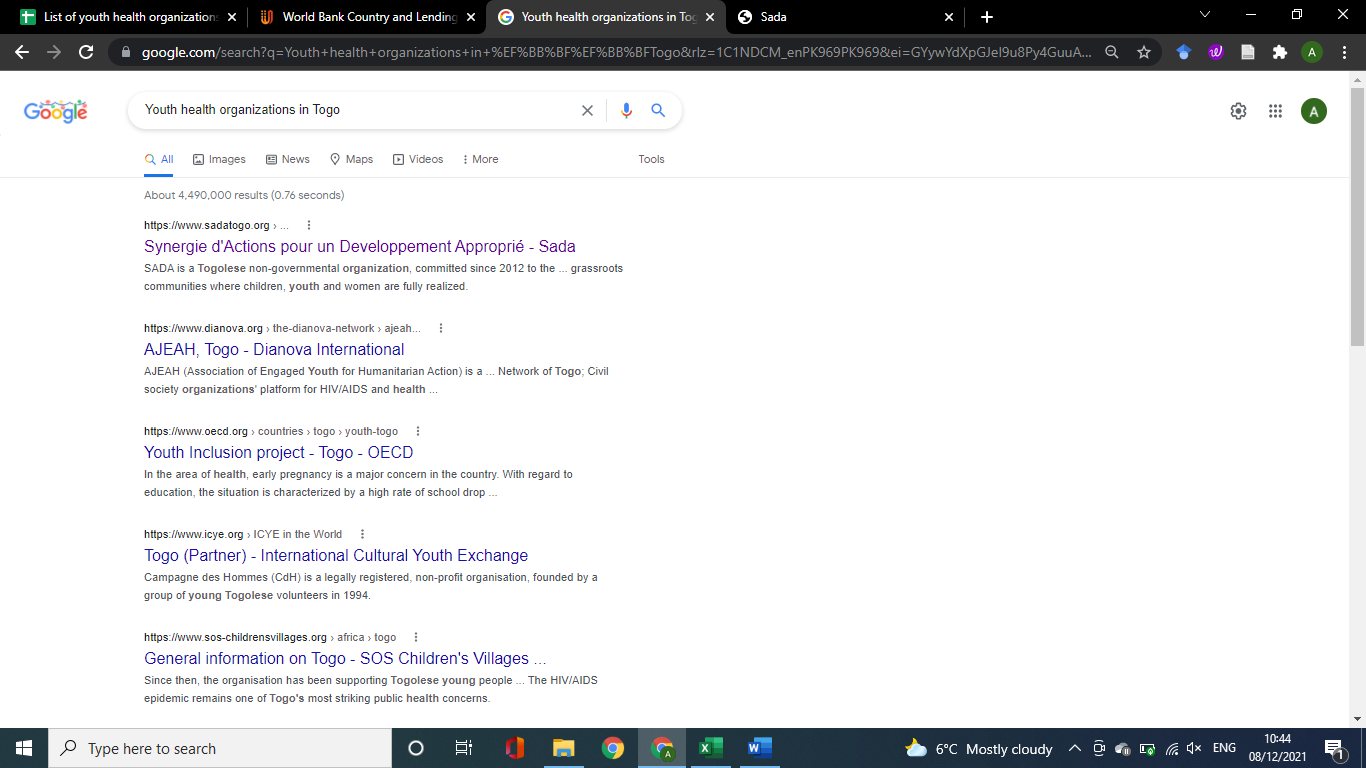 |
| 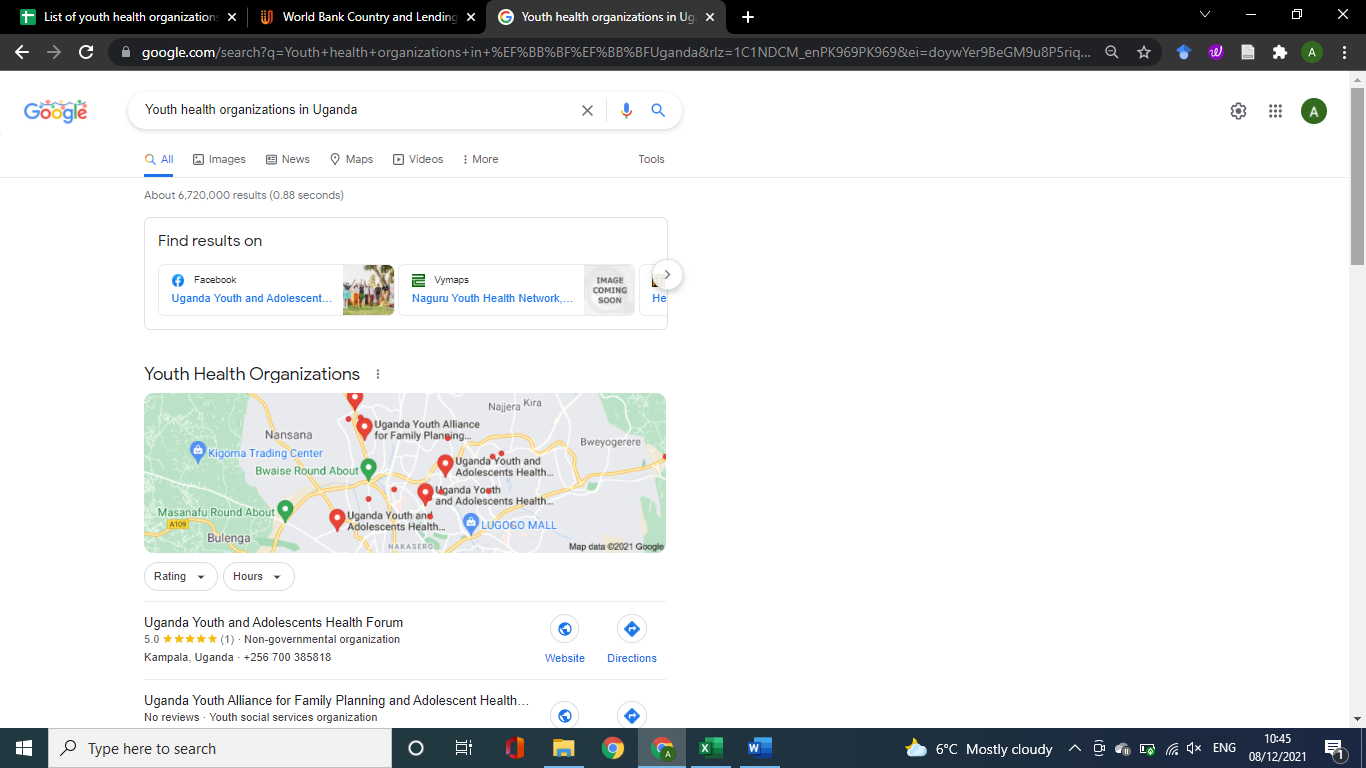 |
| 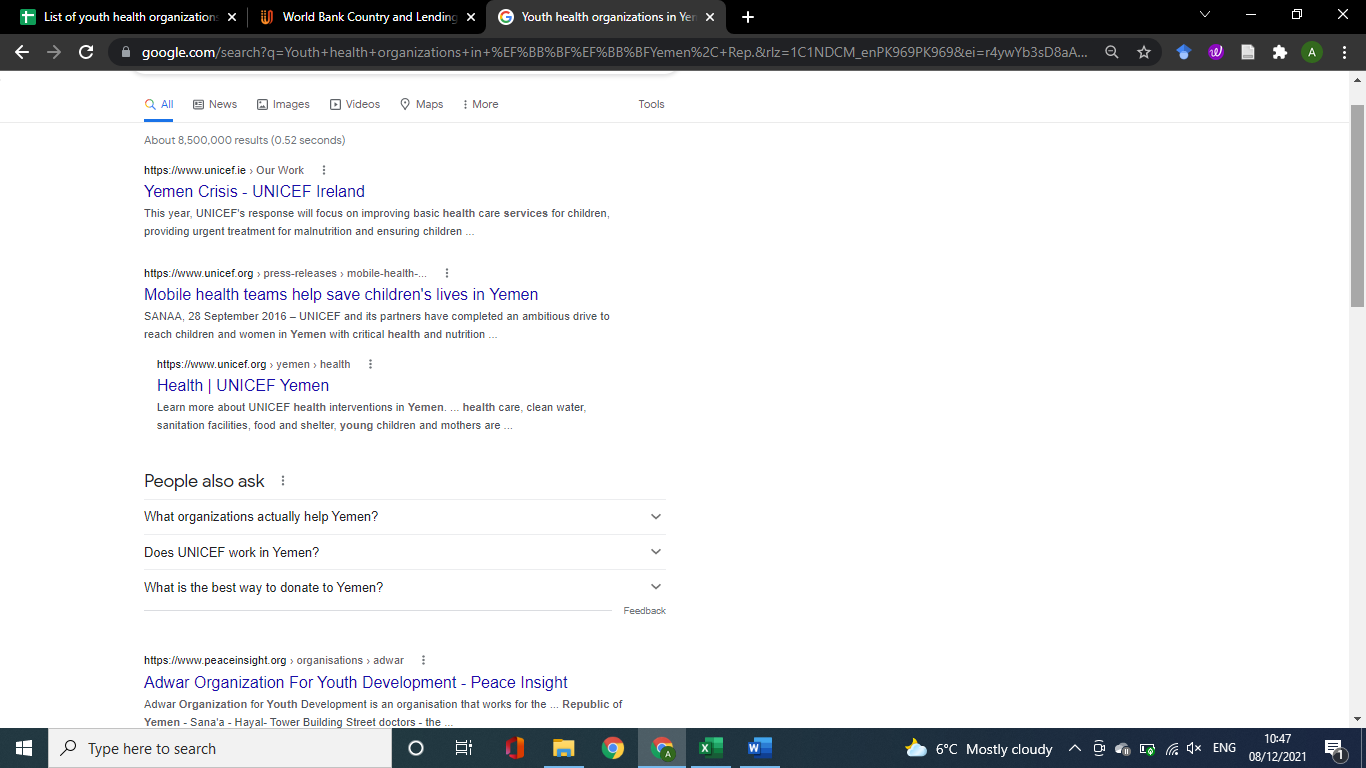 |
| 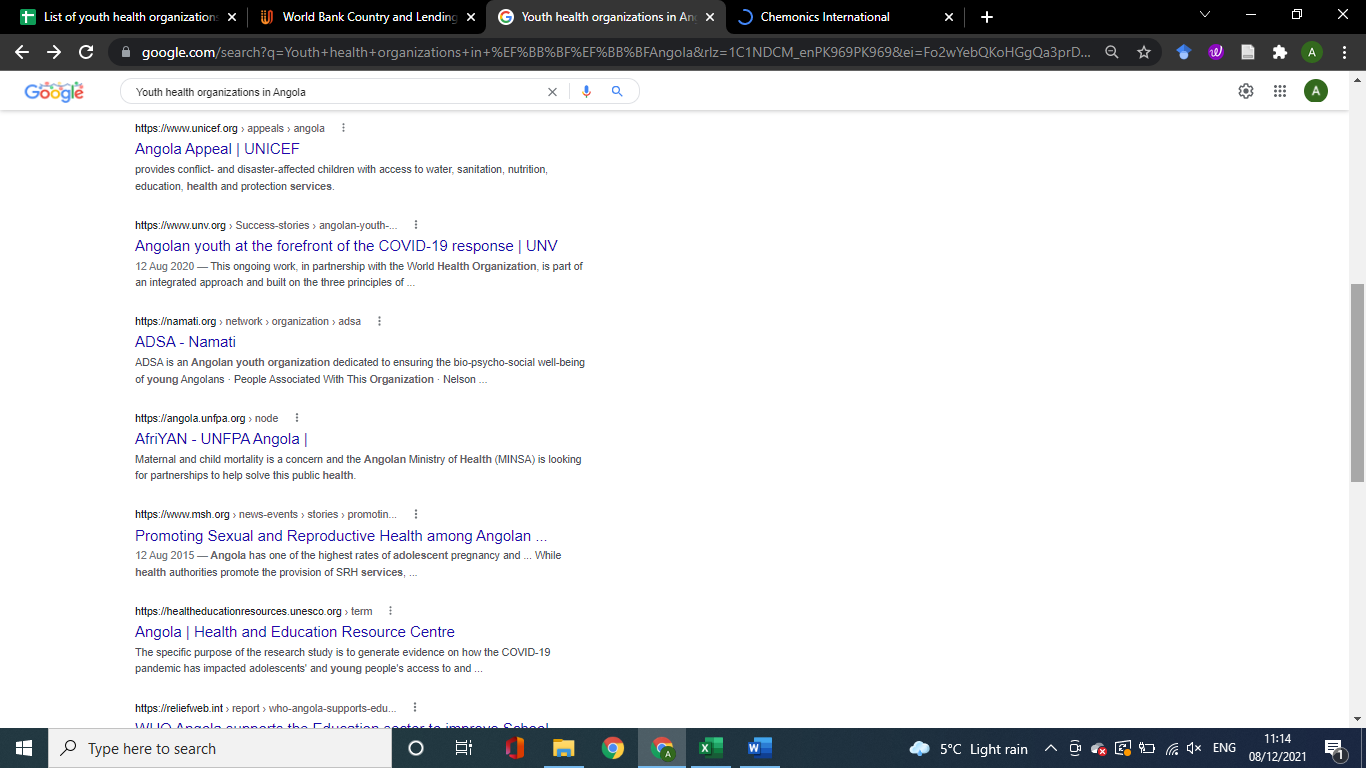 |
| 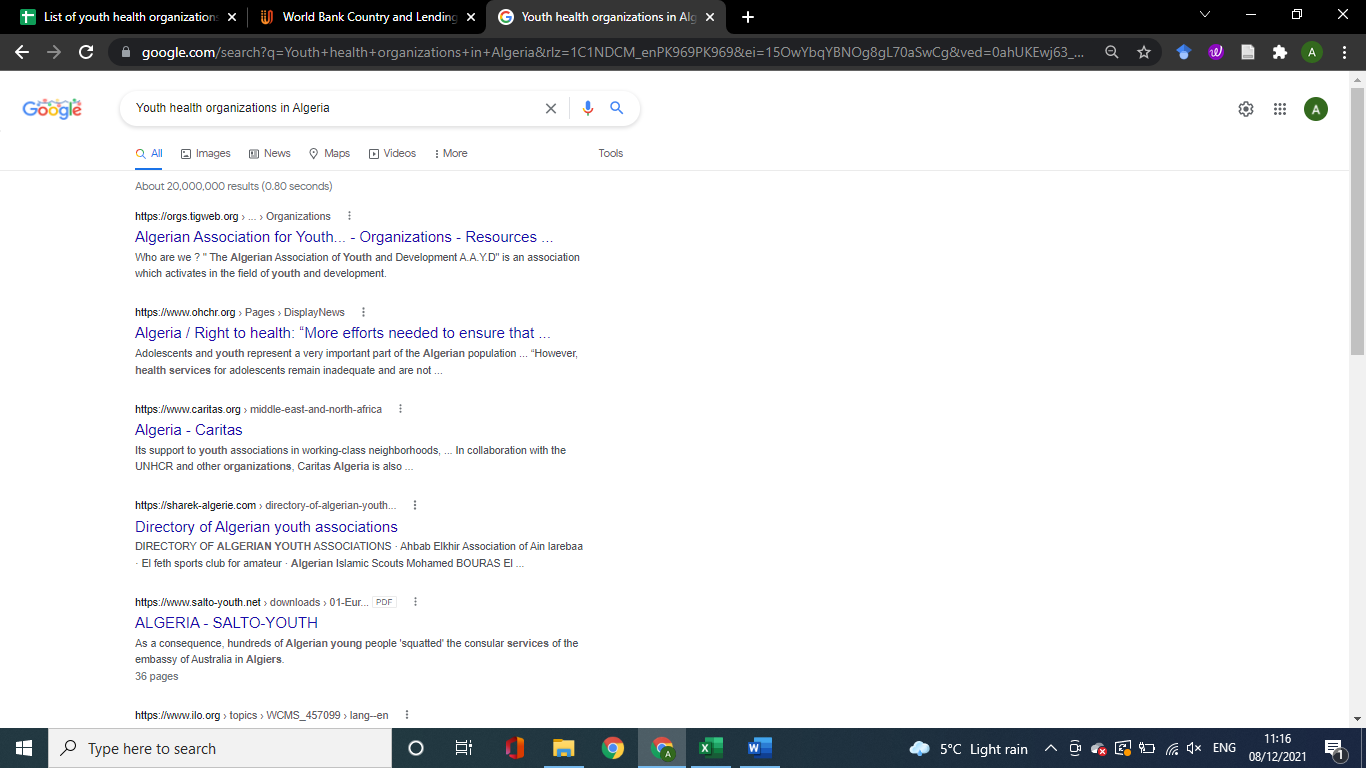 |
| 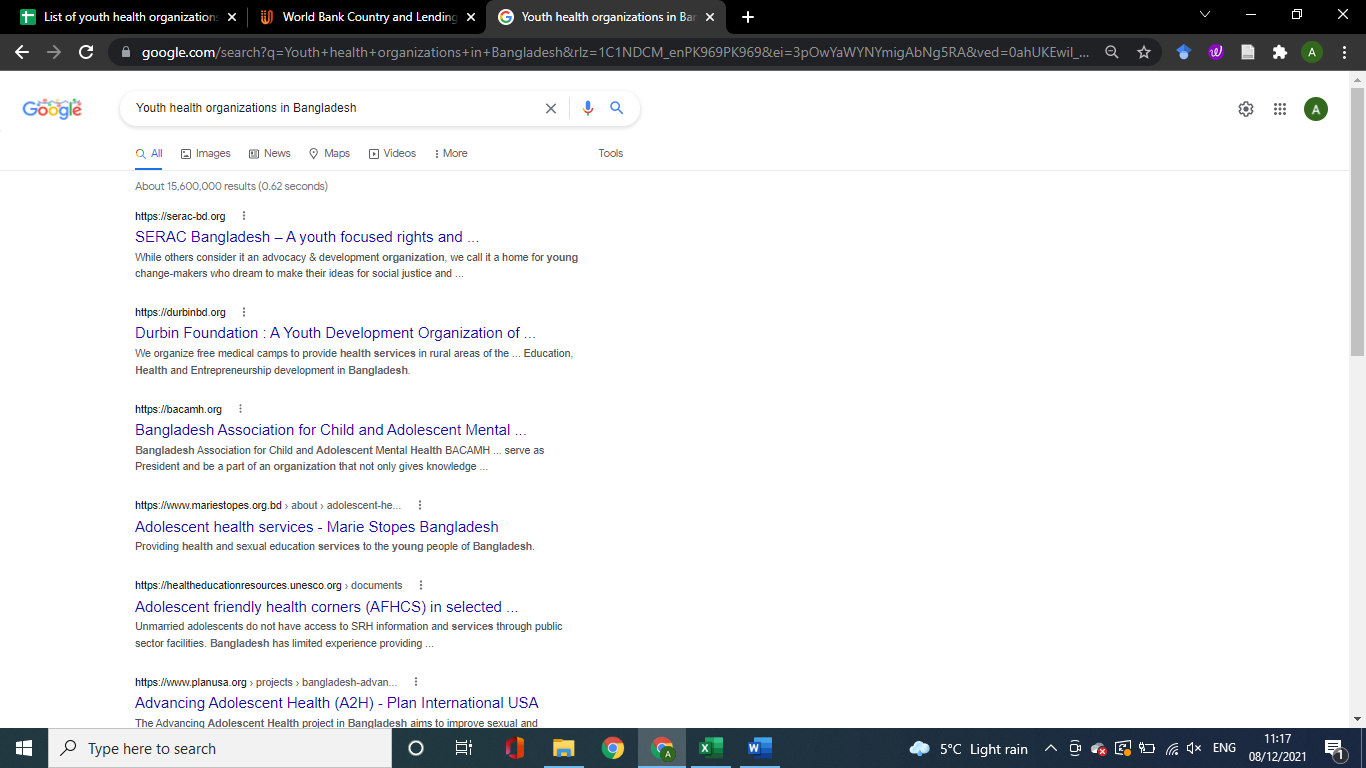 |
| 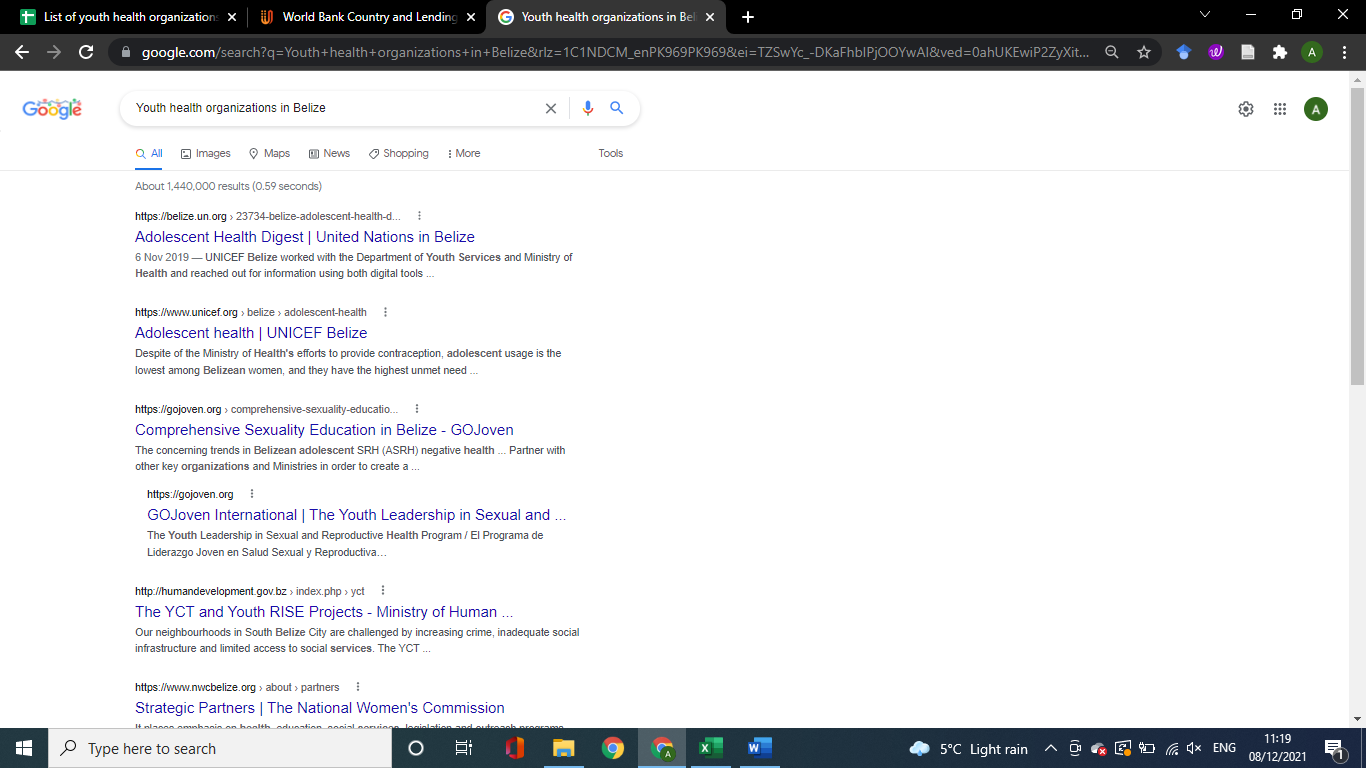 |
| 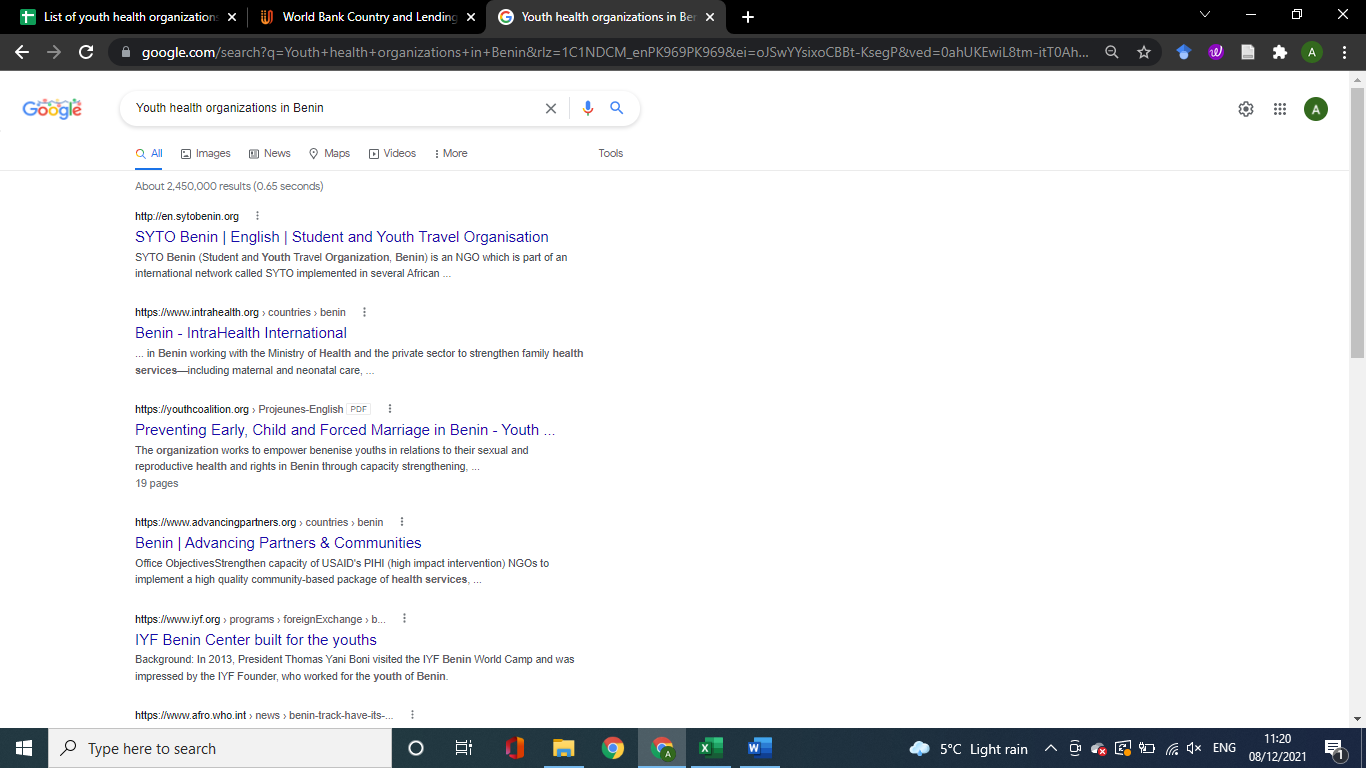 |
| 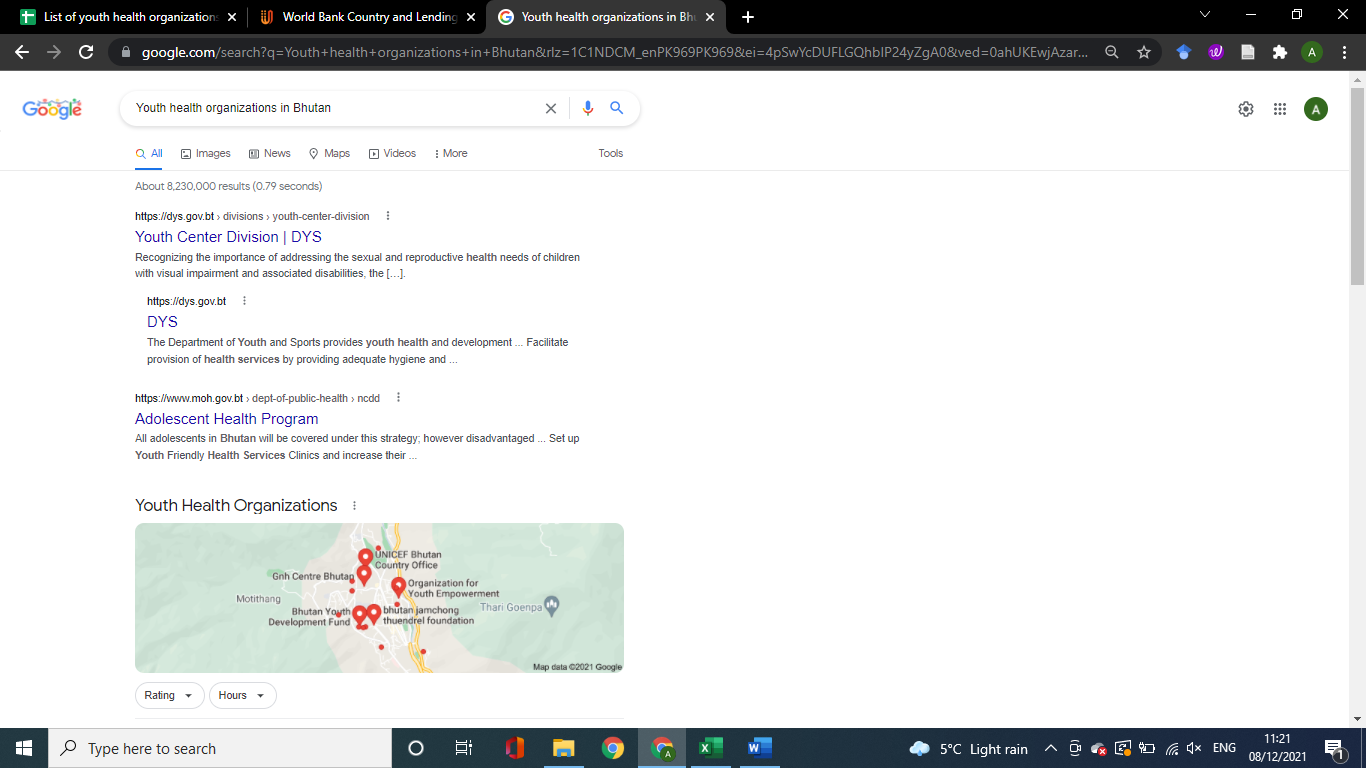 |
| 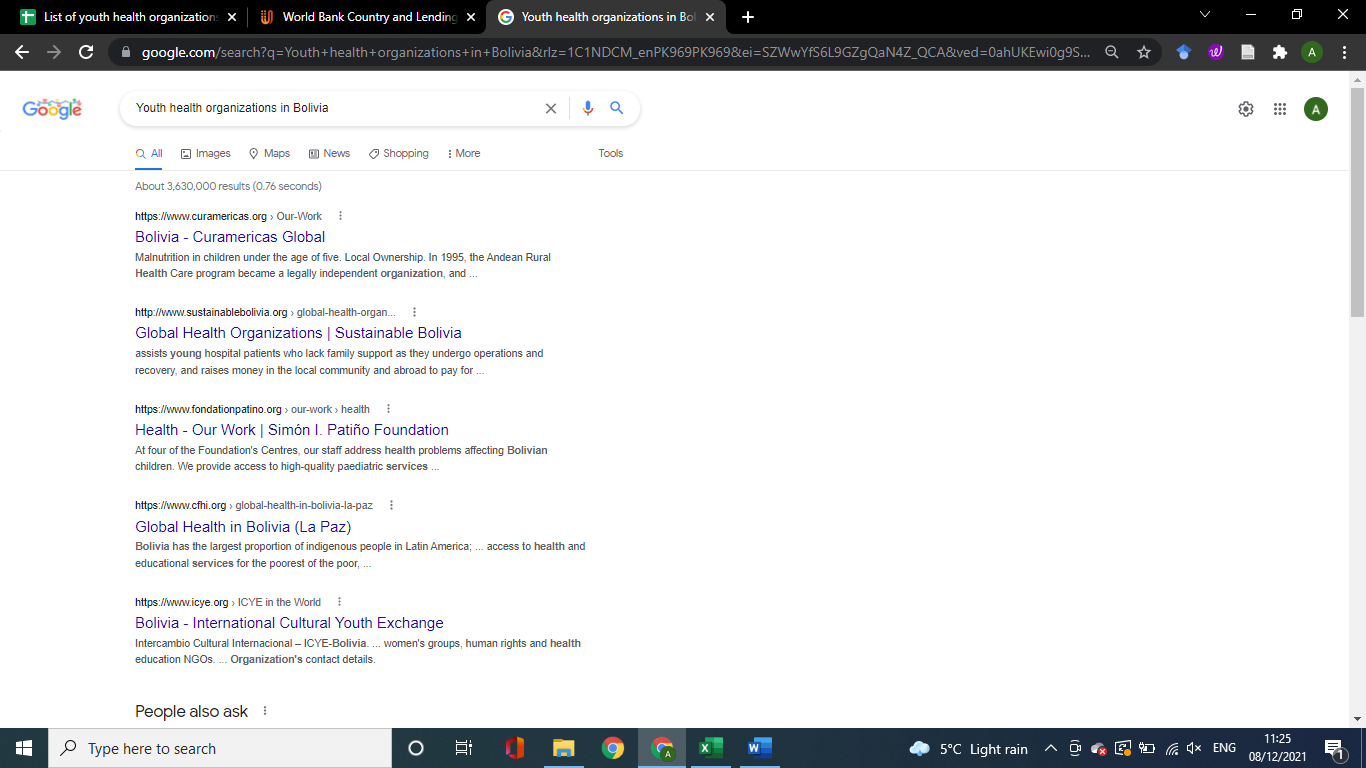 |
| 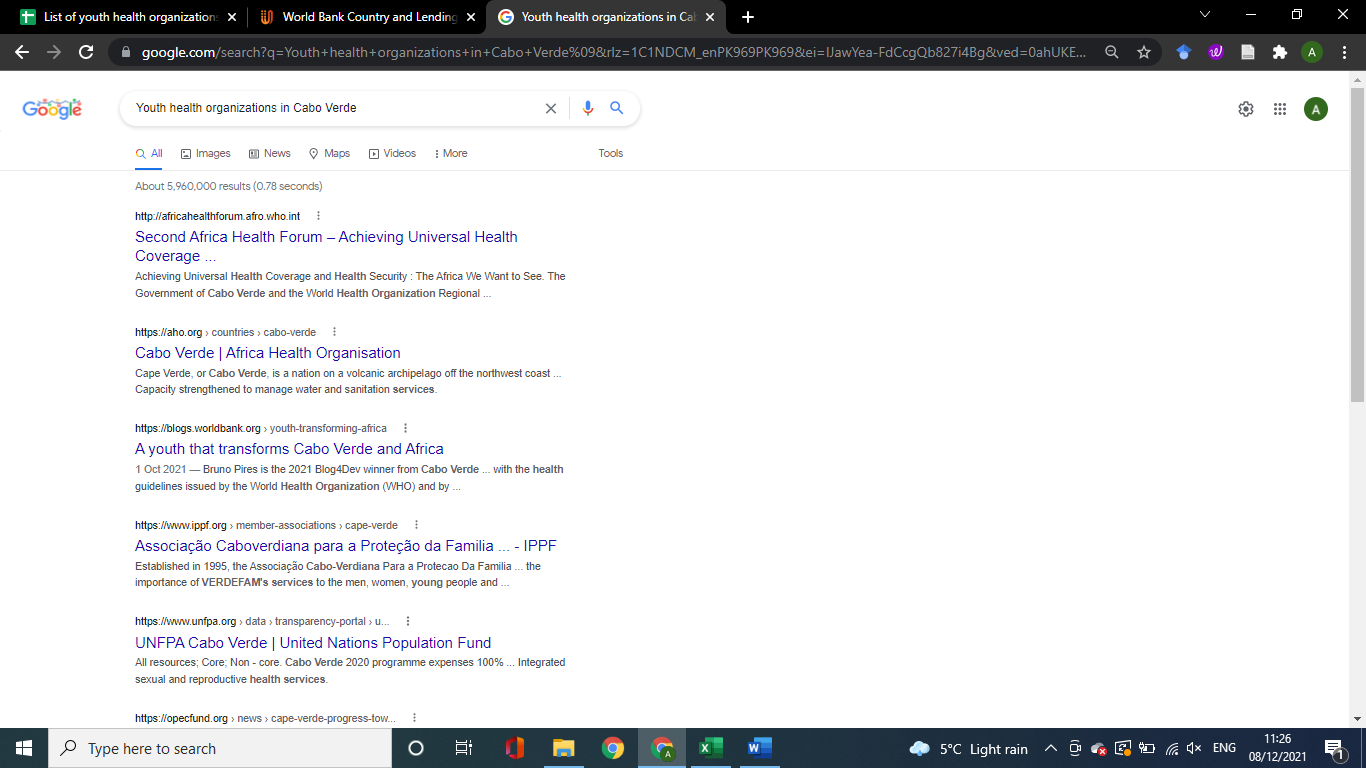 |
| 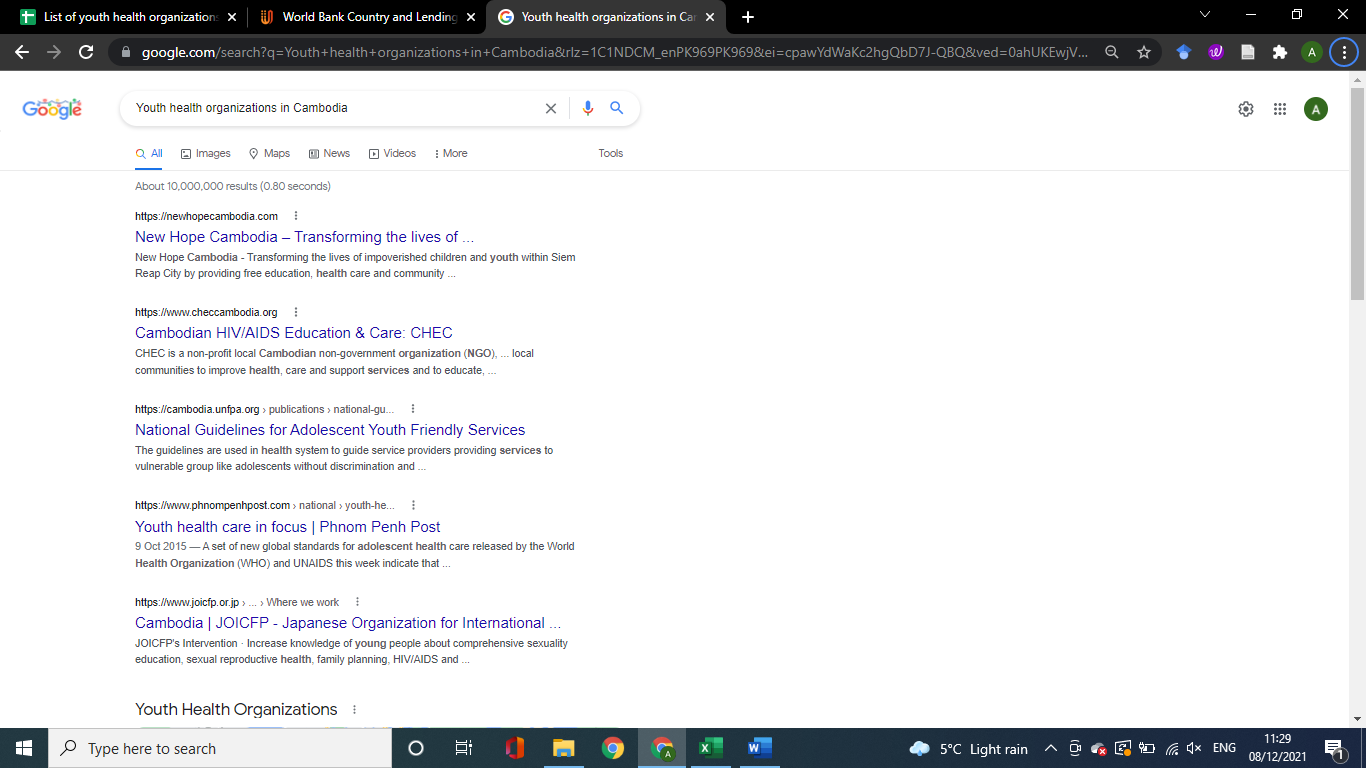 |
| 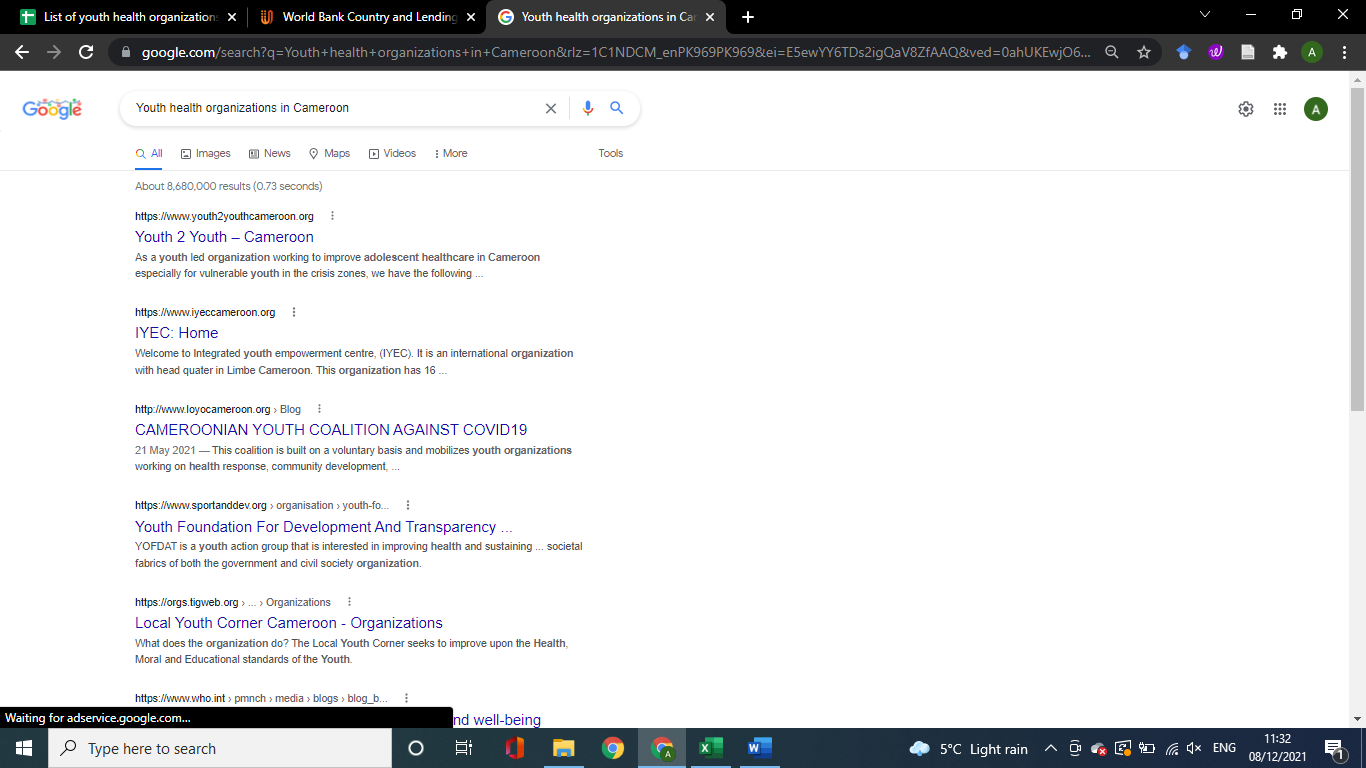 |
| 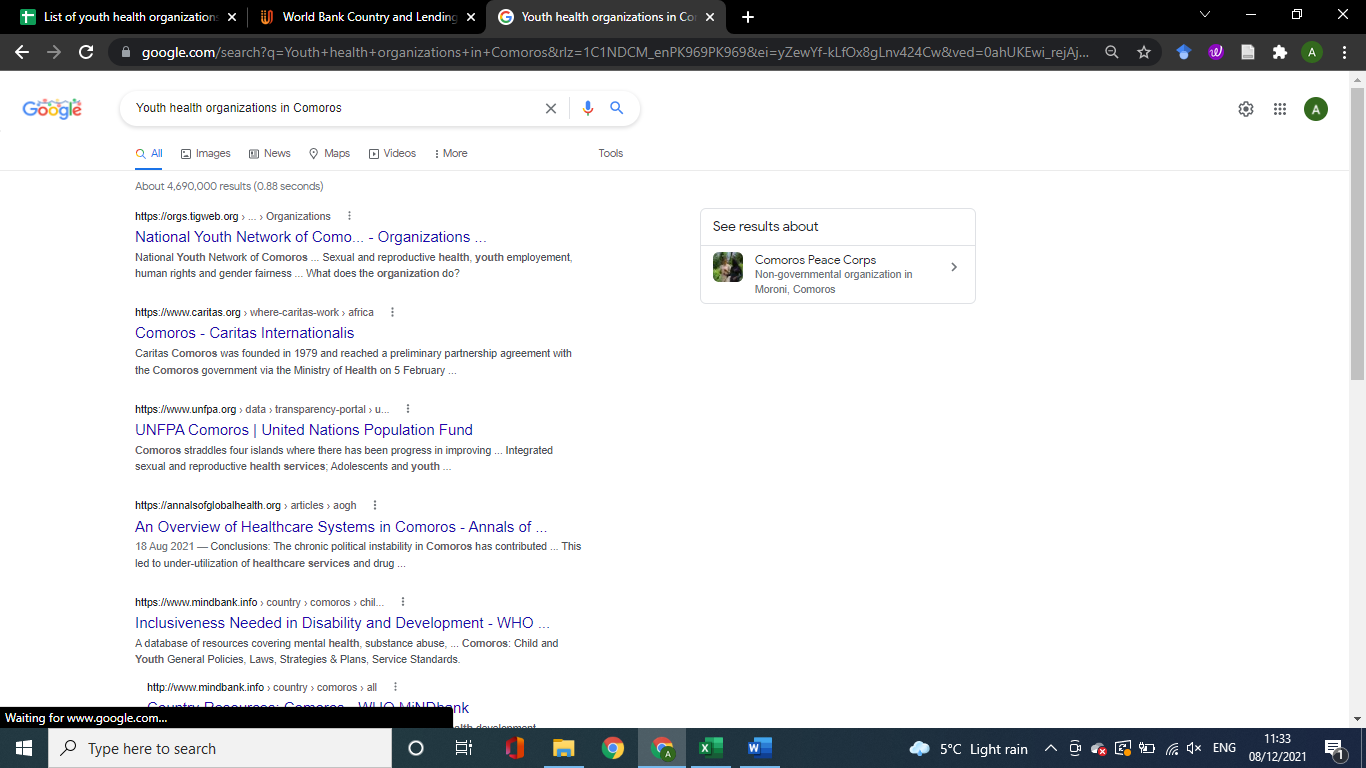 |
| 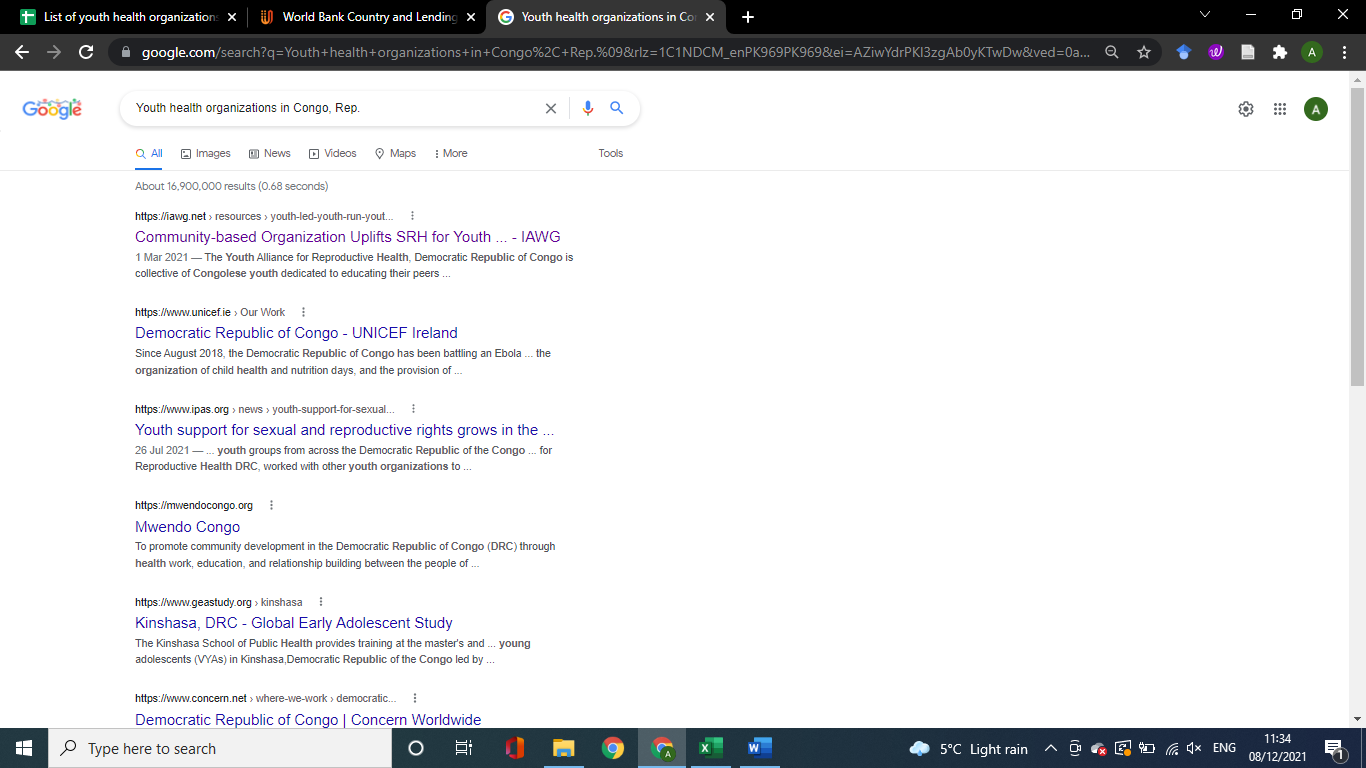 |
| 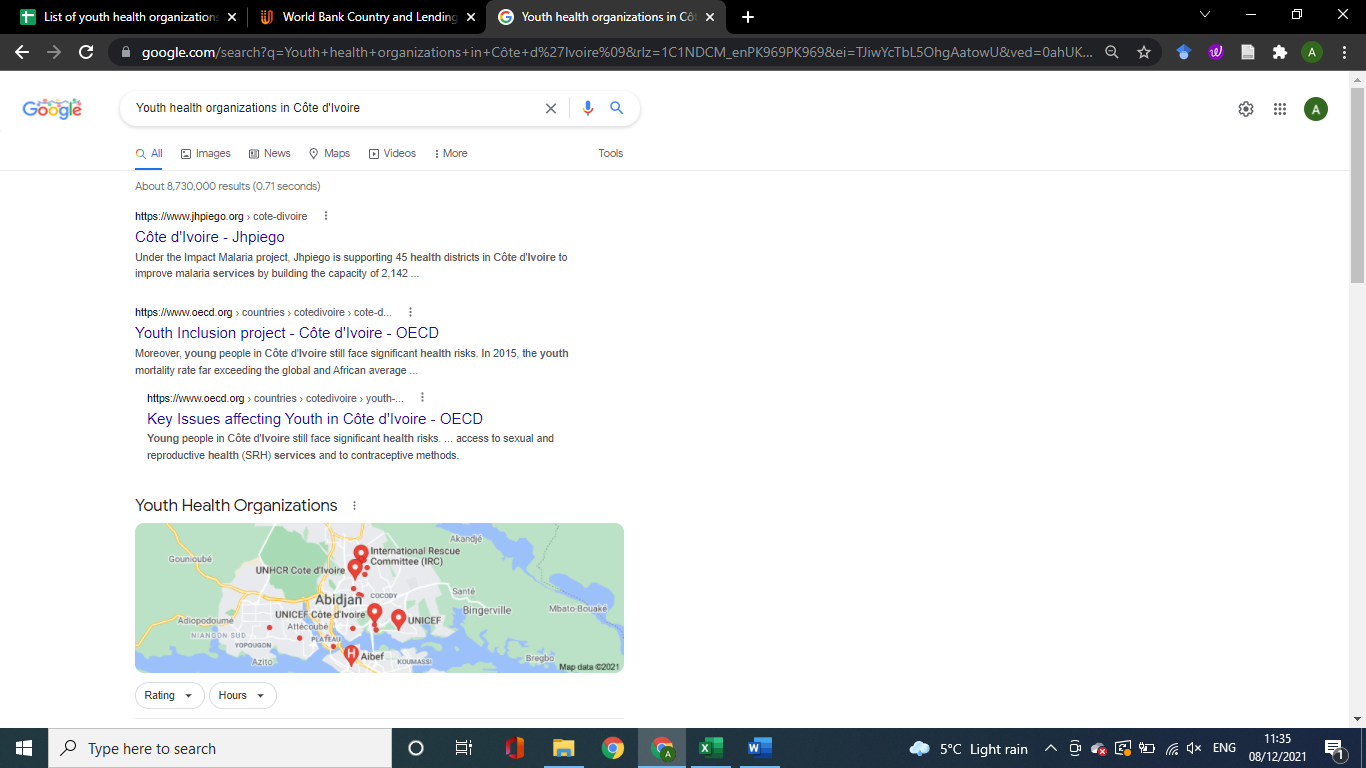 |
| 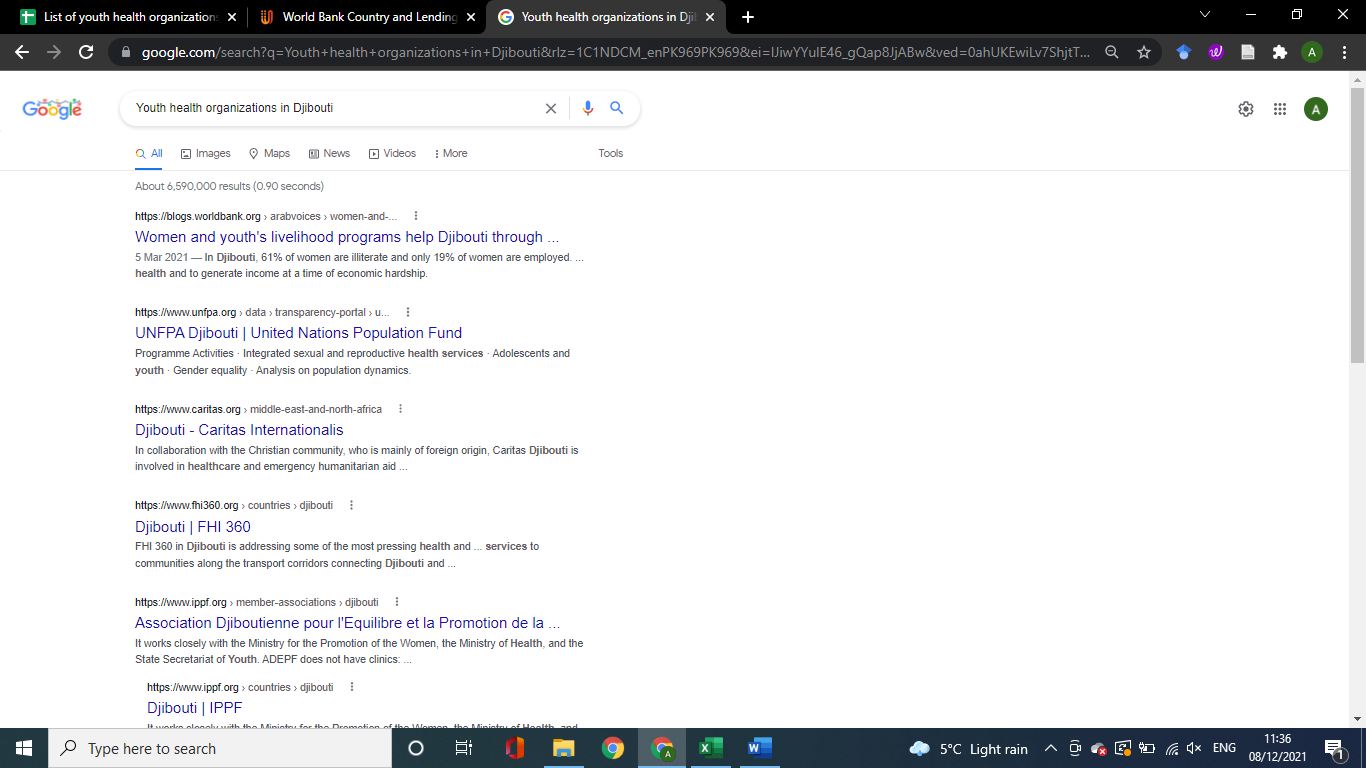 |
| 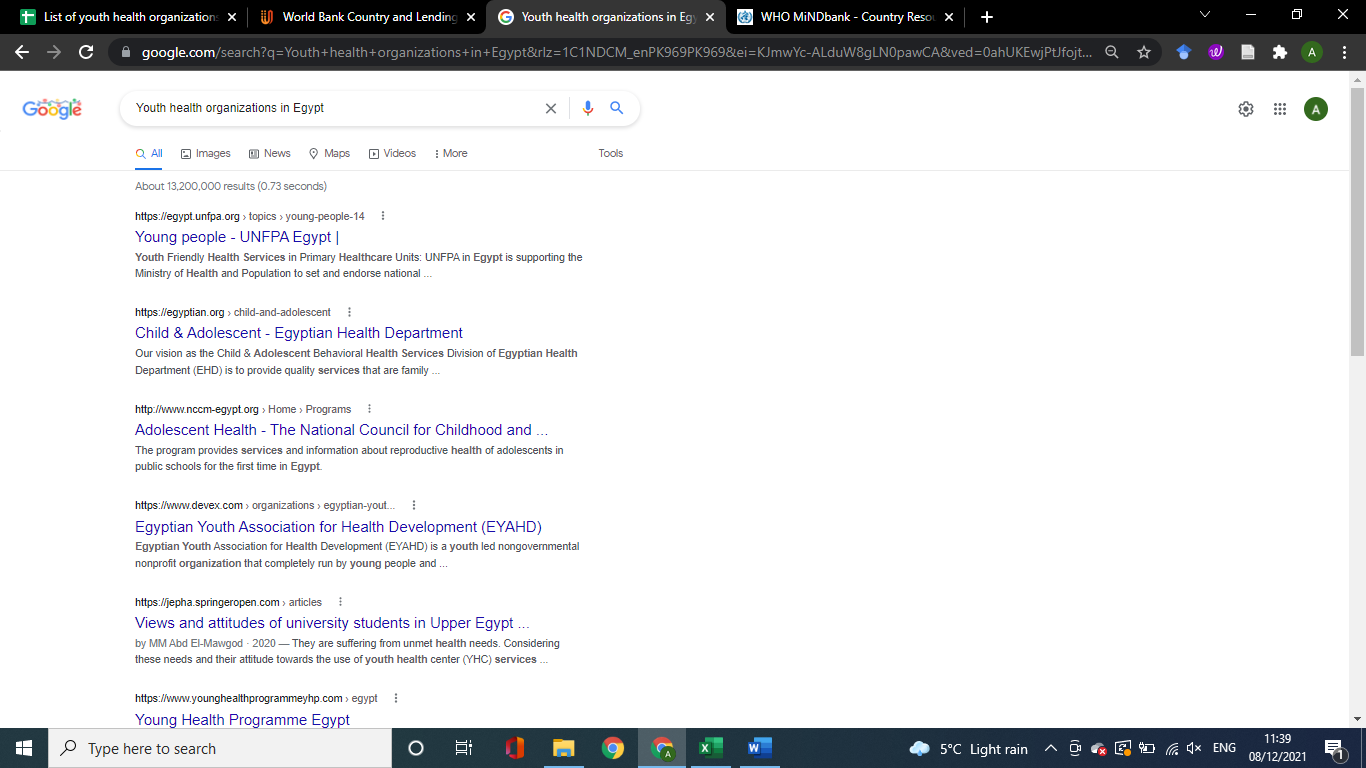 |
| 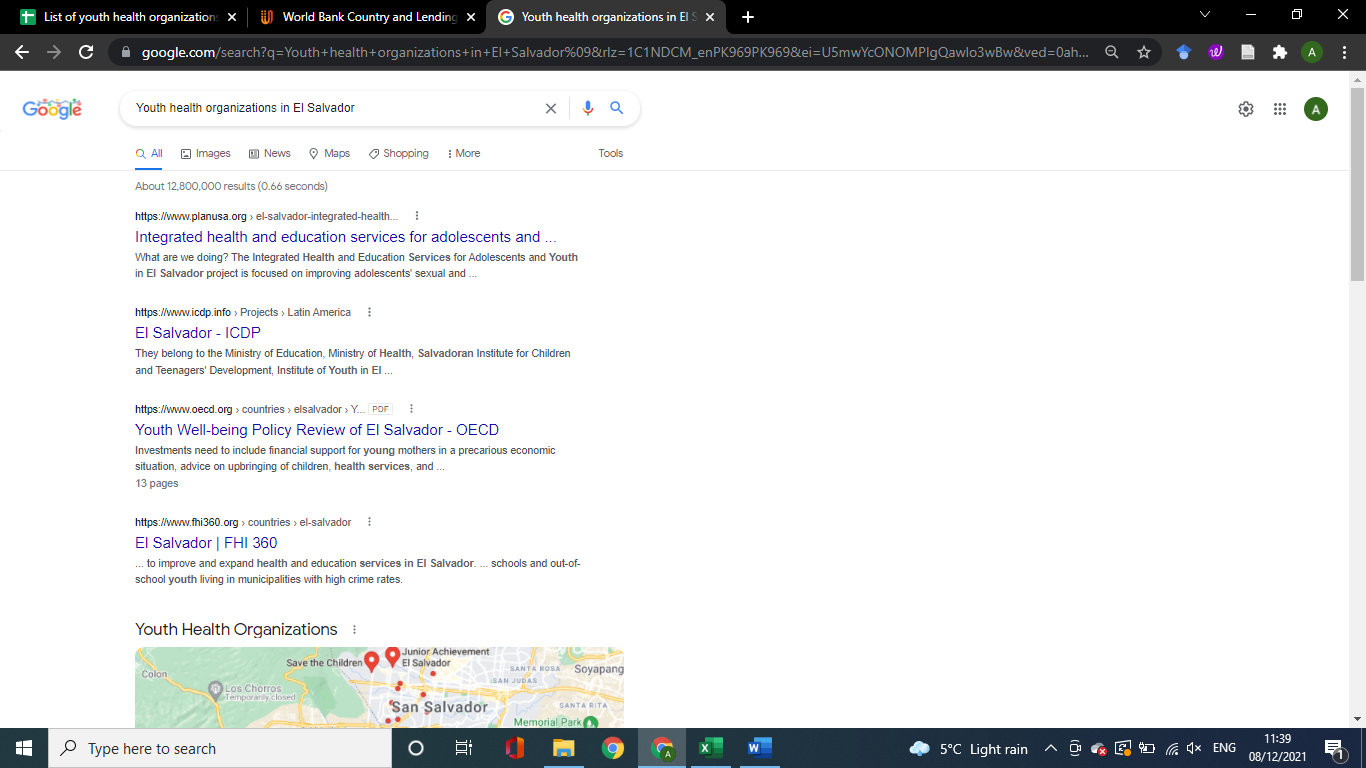 |
| 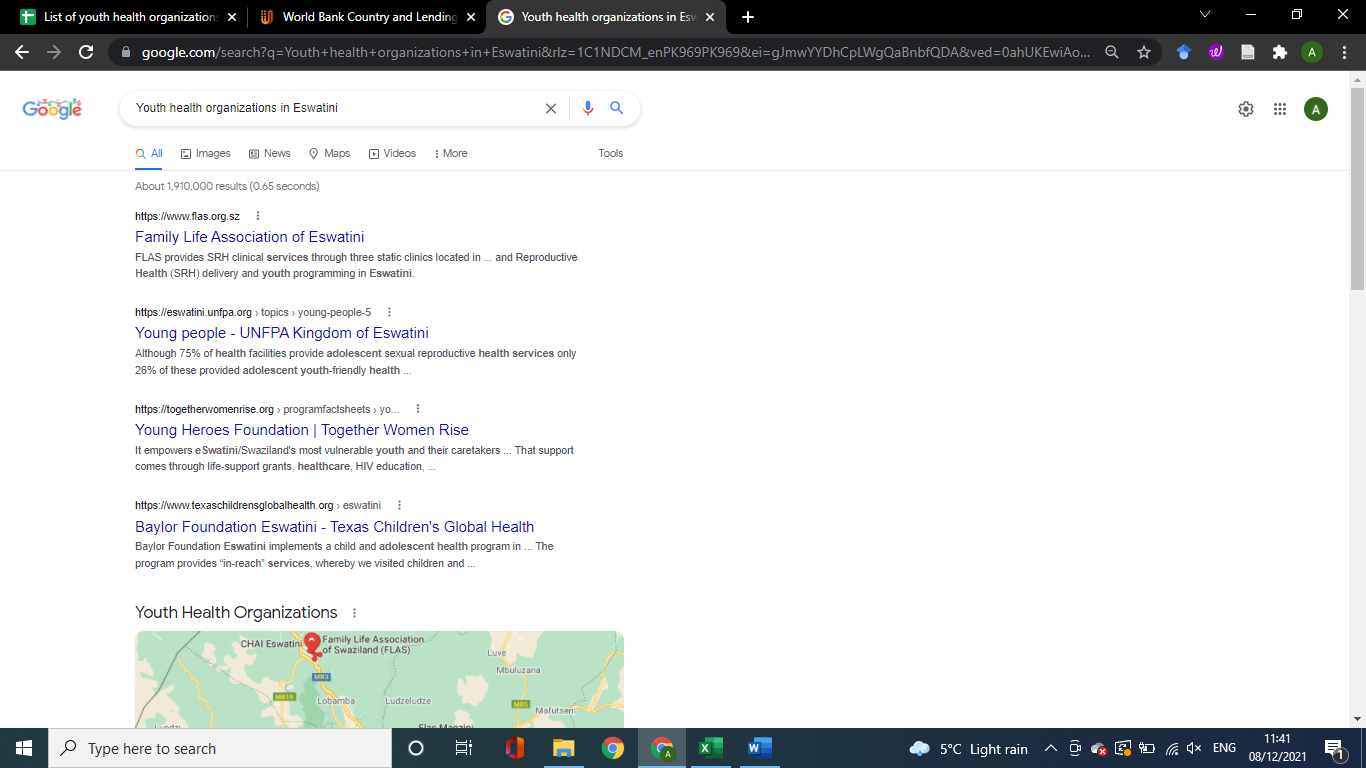 |
| 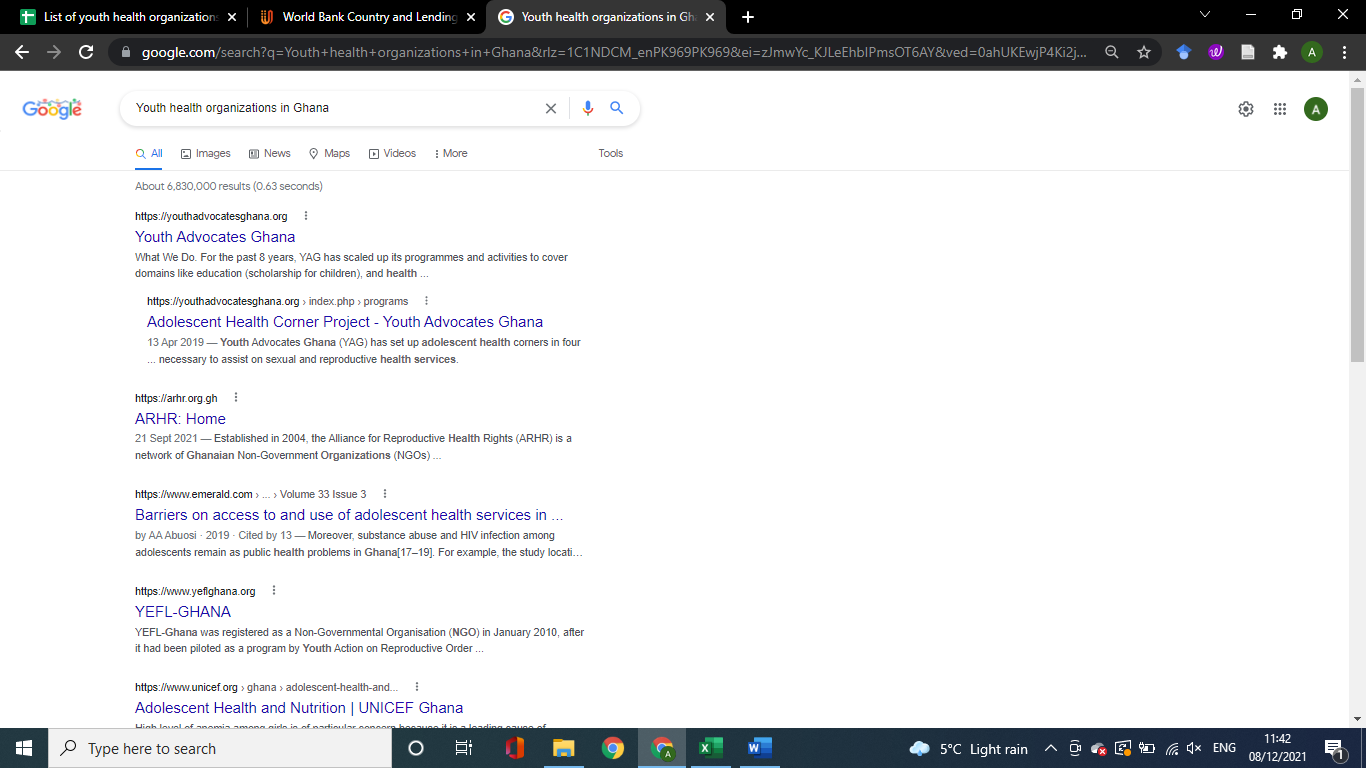 |
| 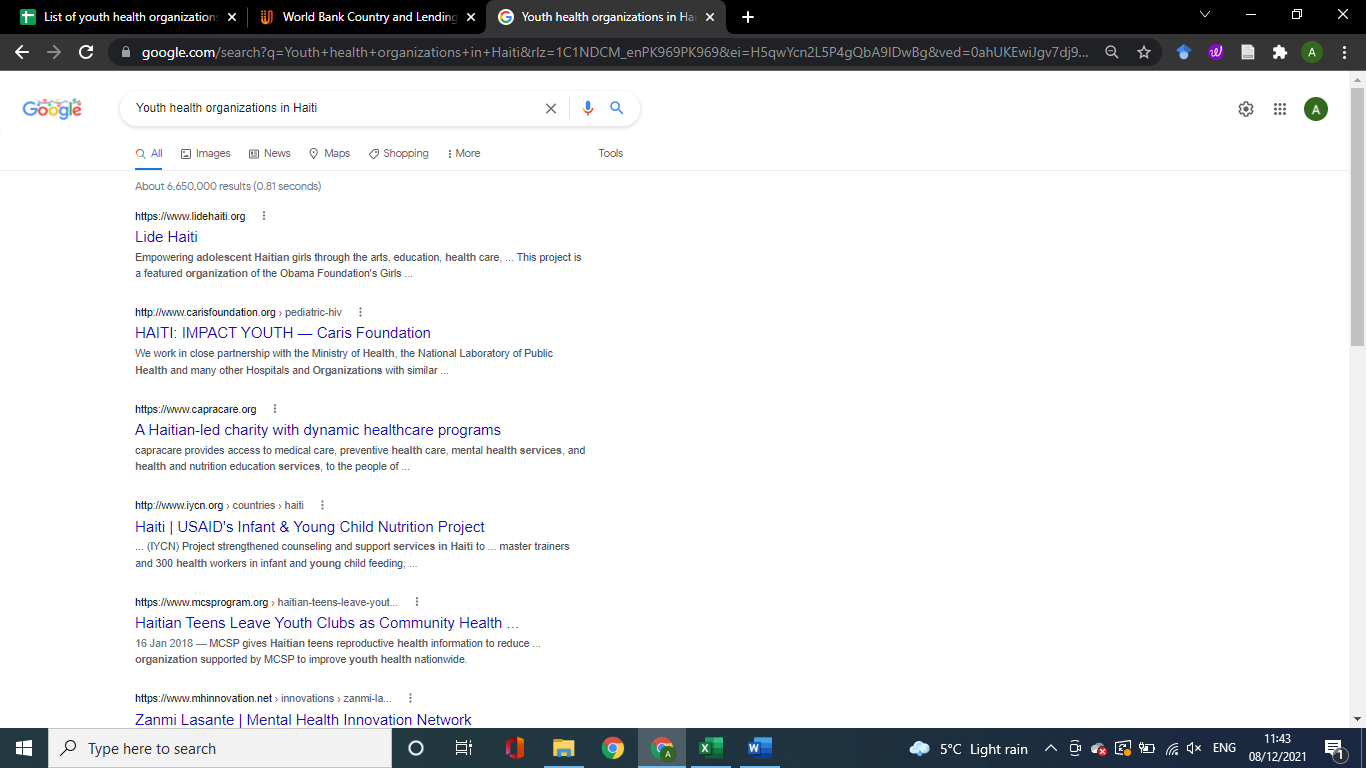 |
| 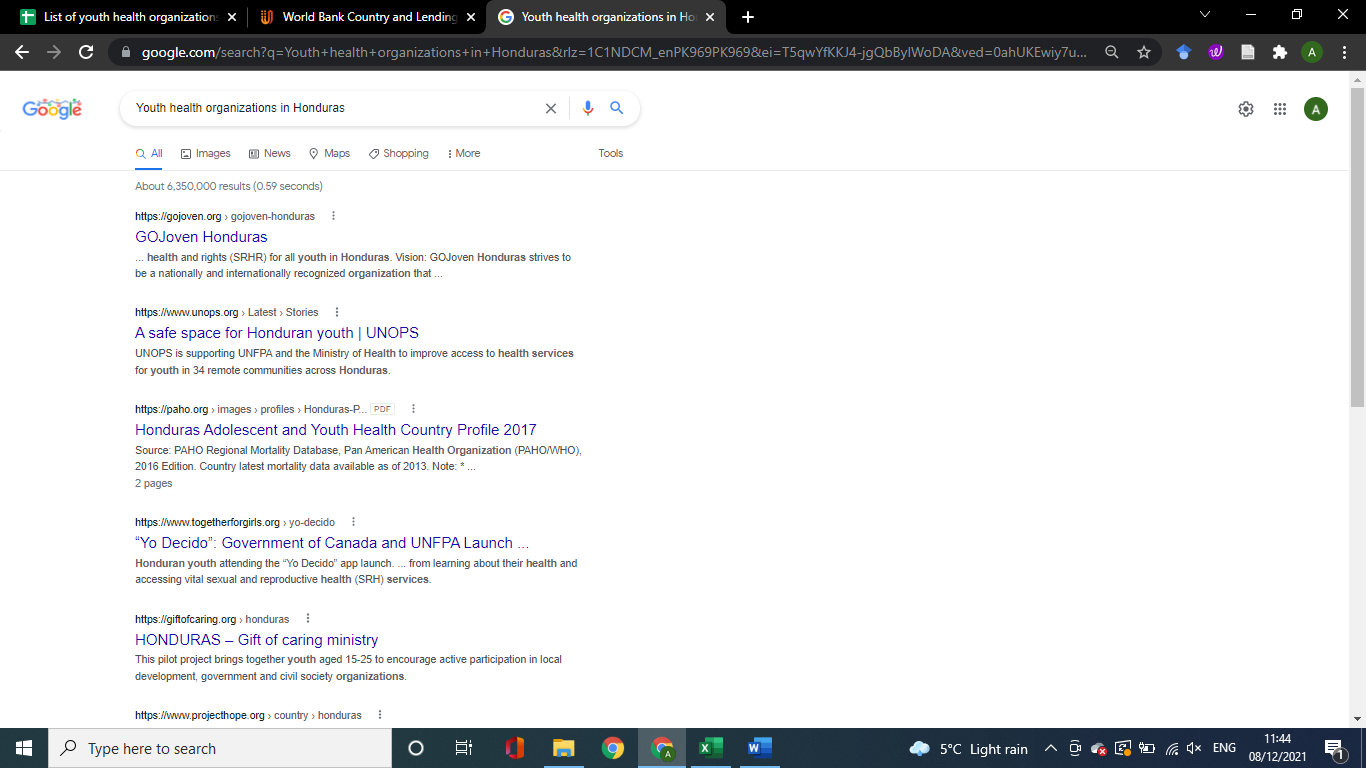 |
| 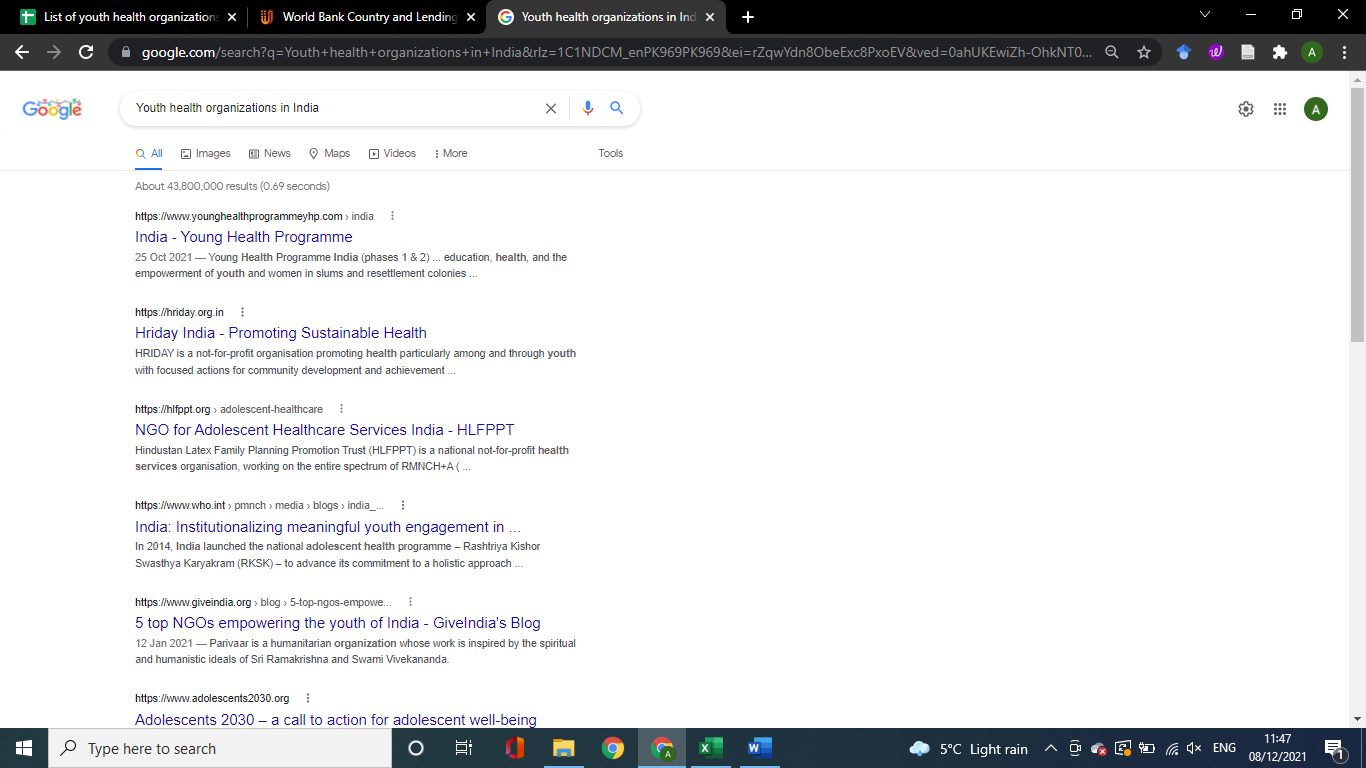 |
| 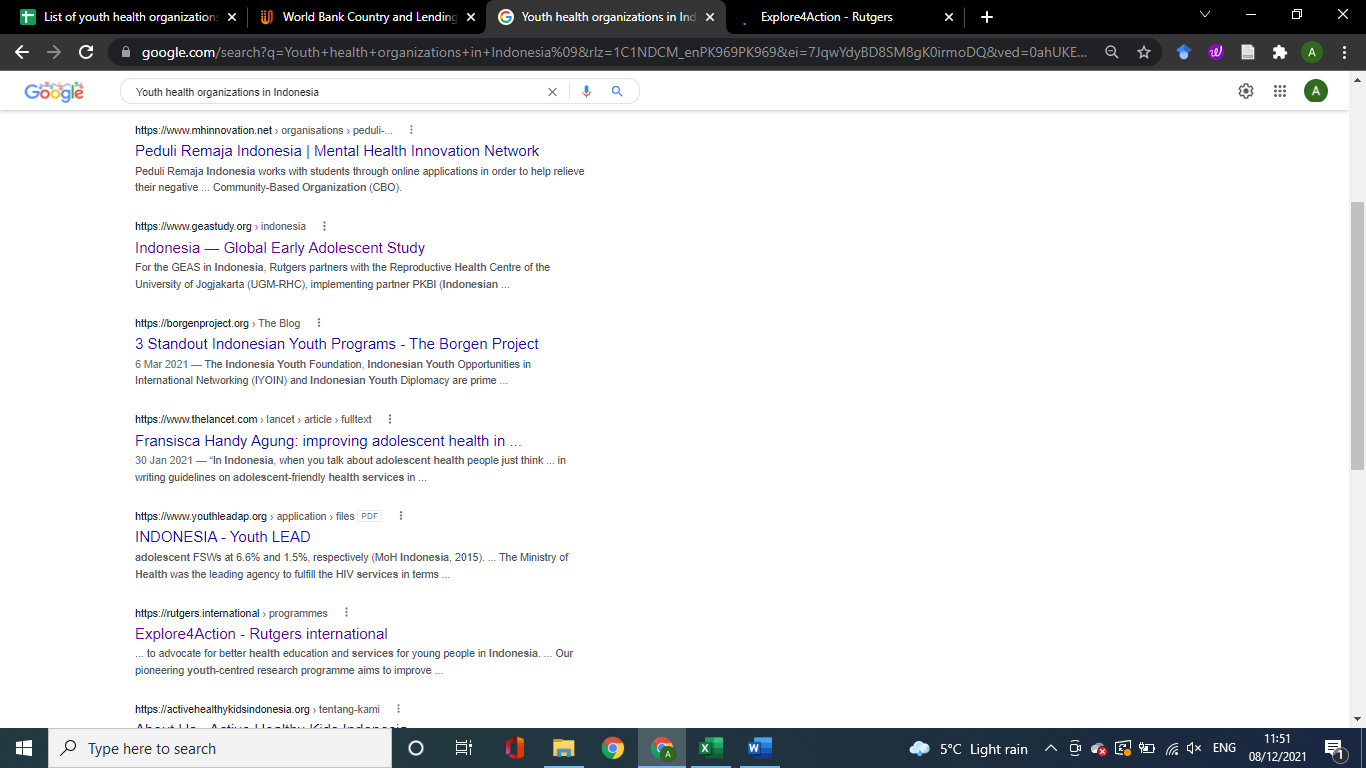 |
| 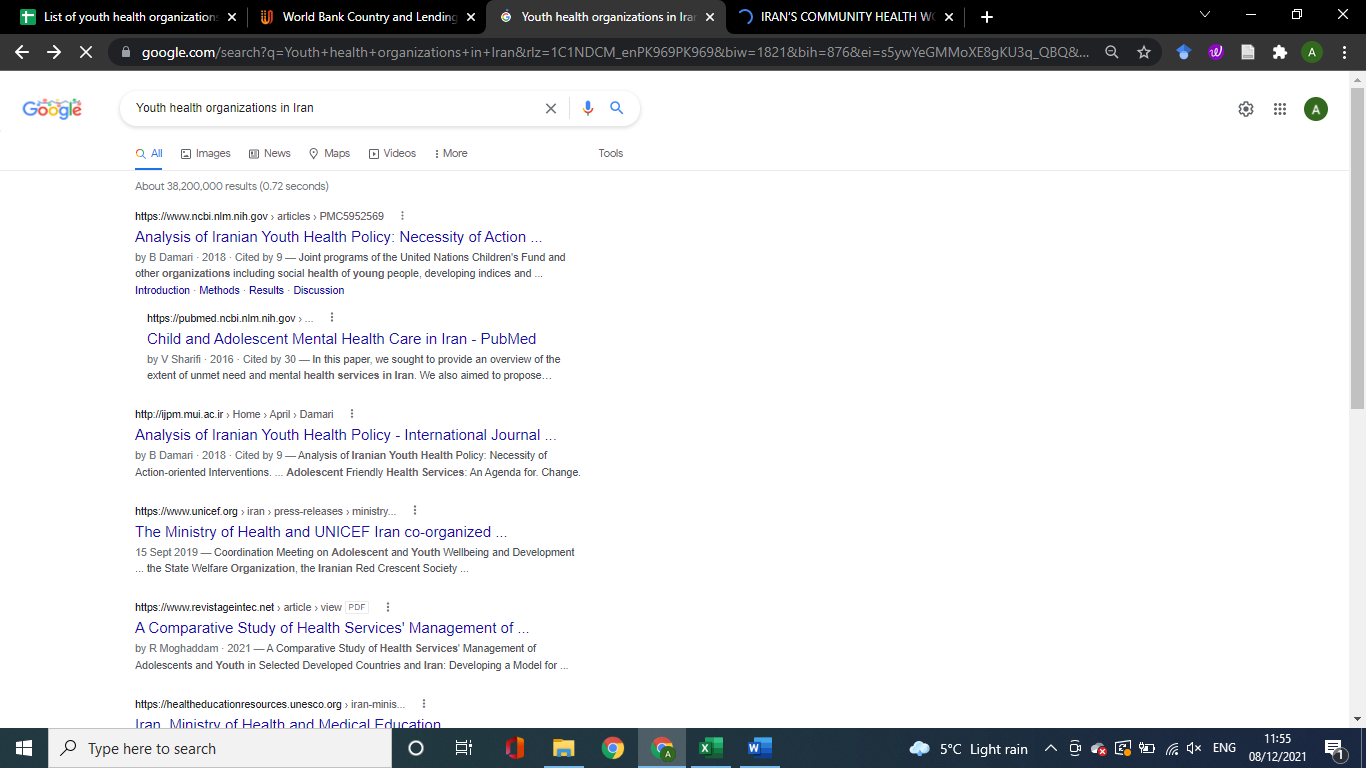 |
| 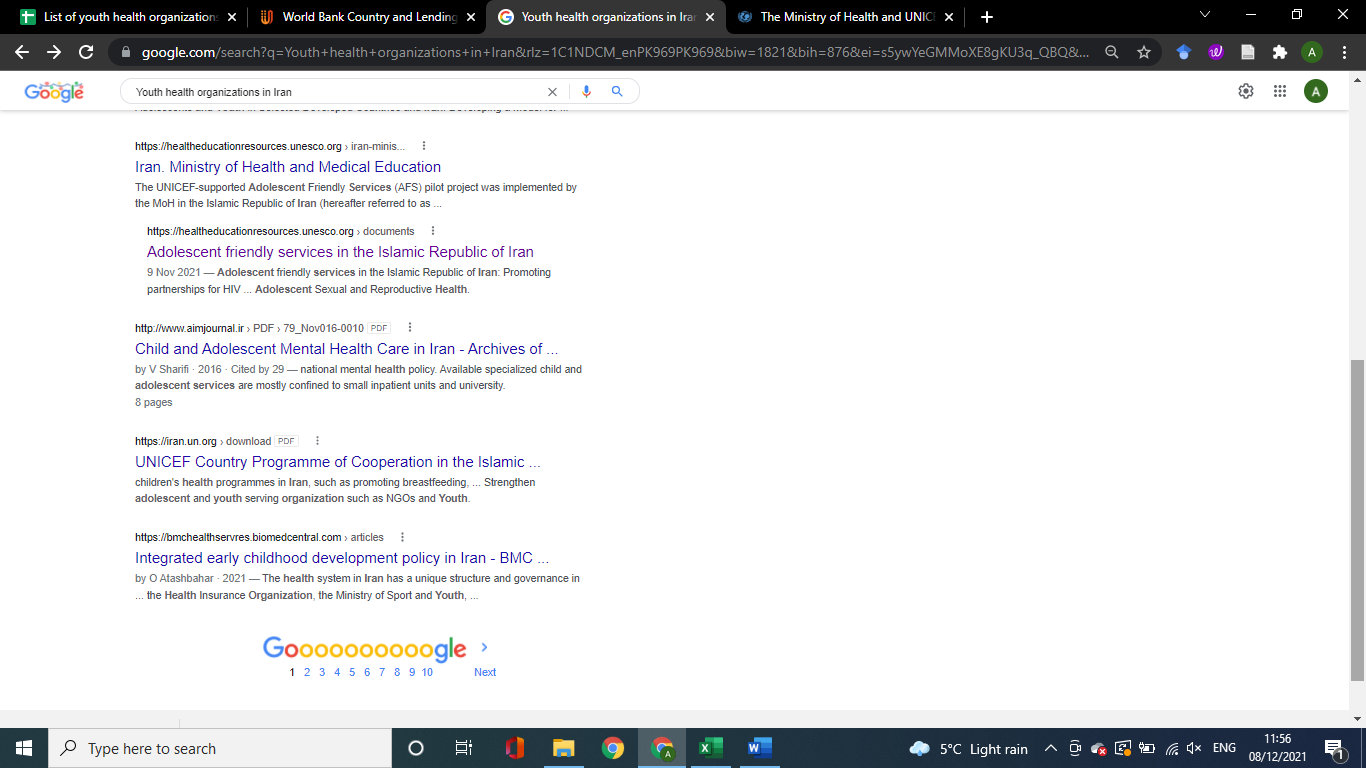 |
| 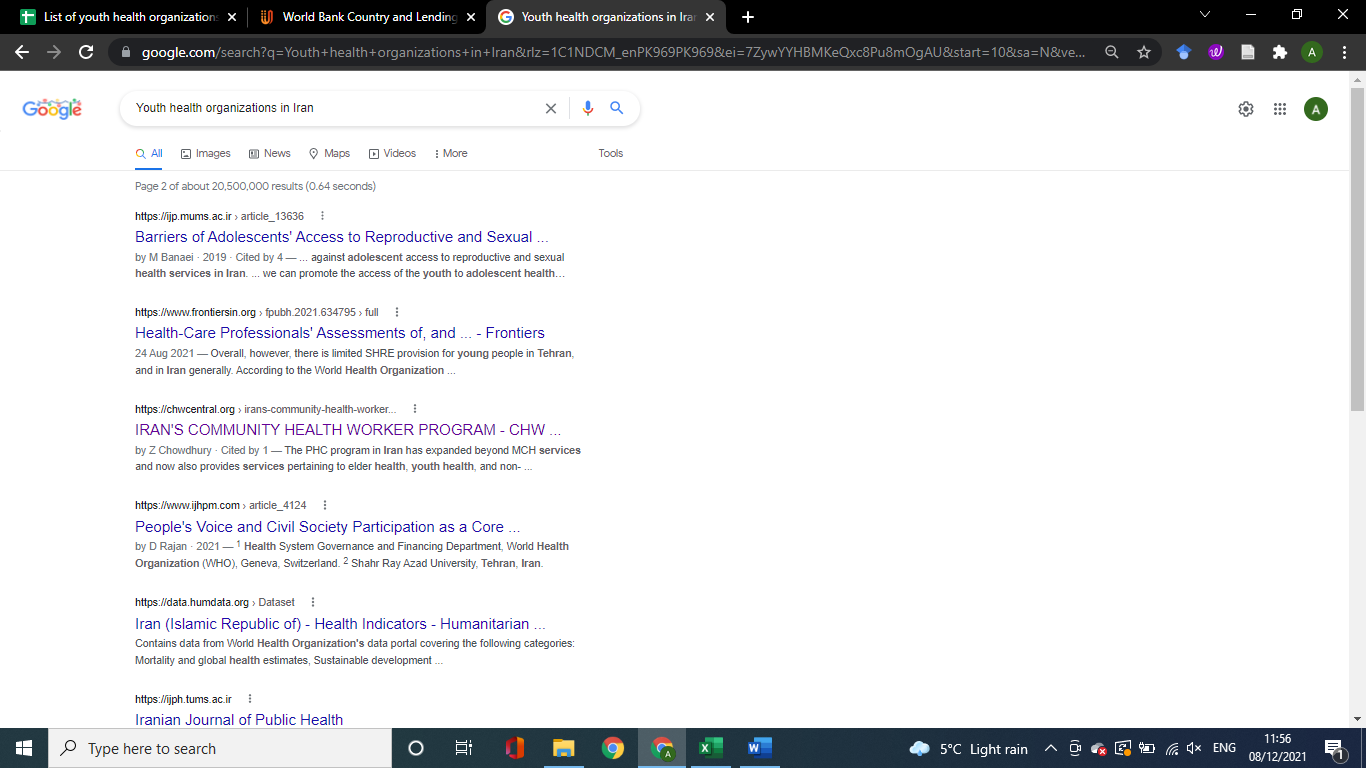 |
| 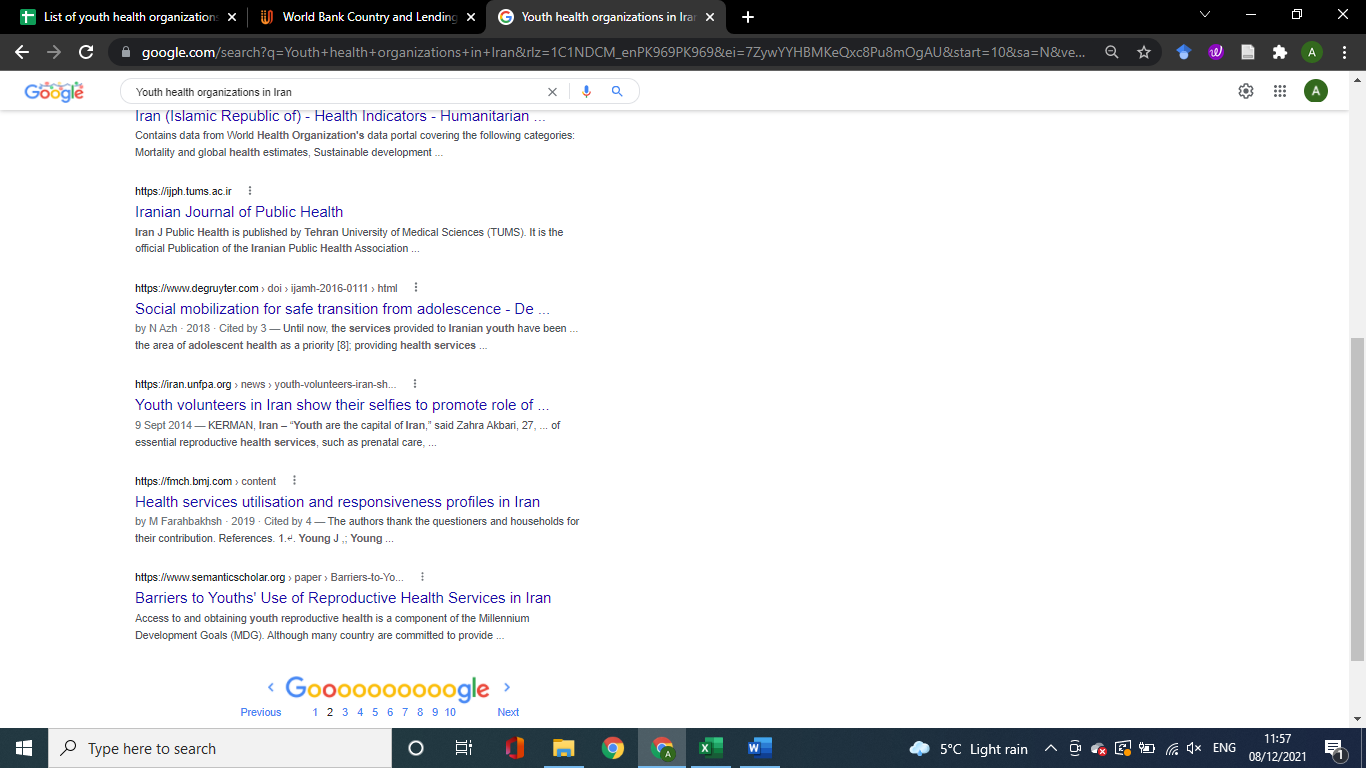 |
| 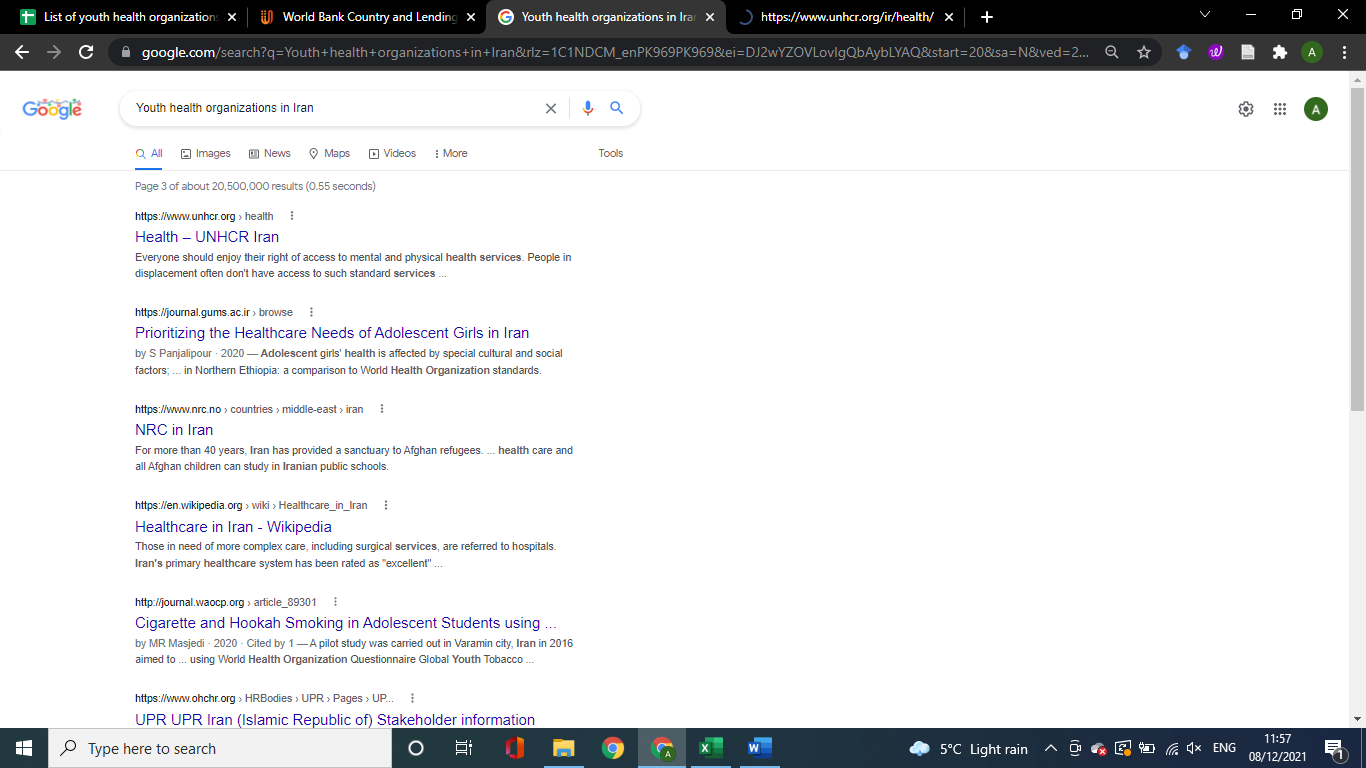 |
| 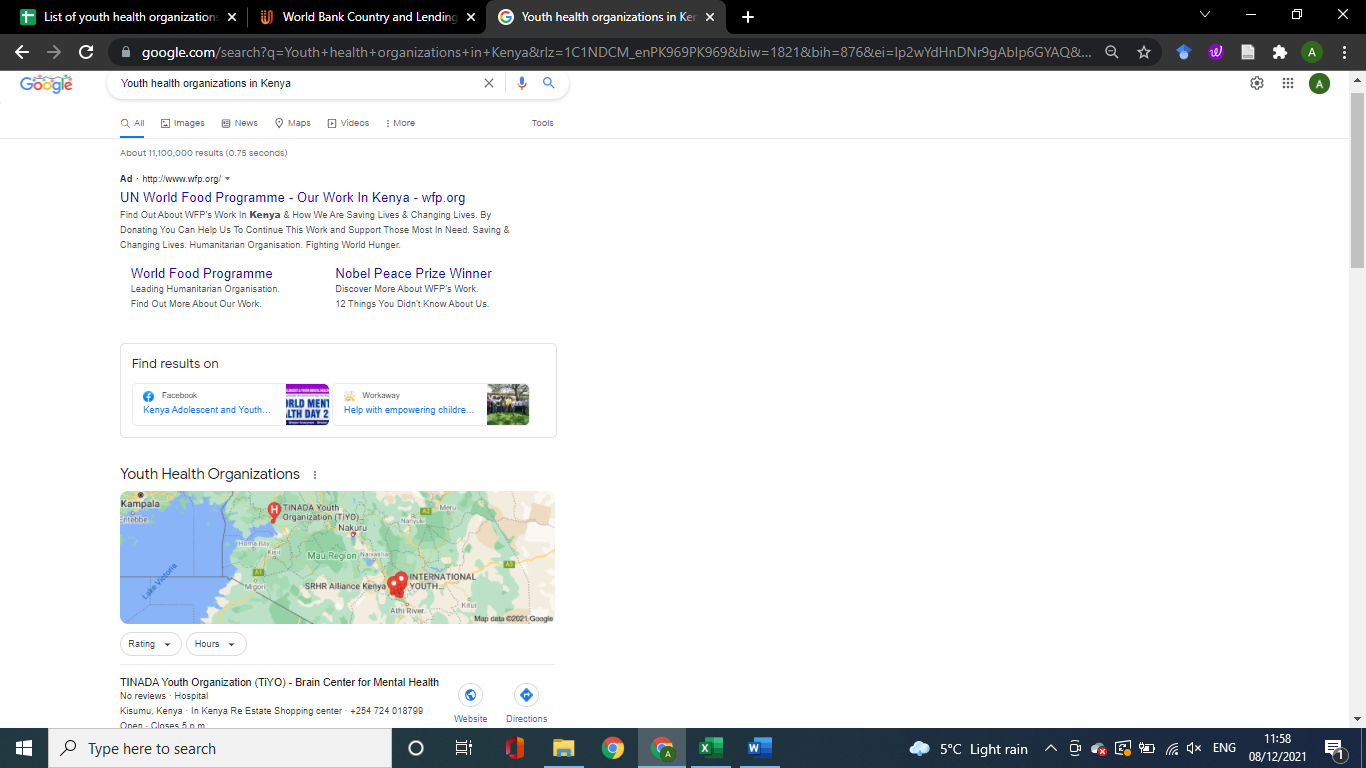 |
| 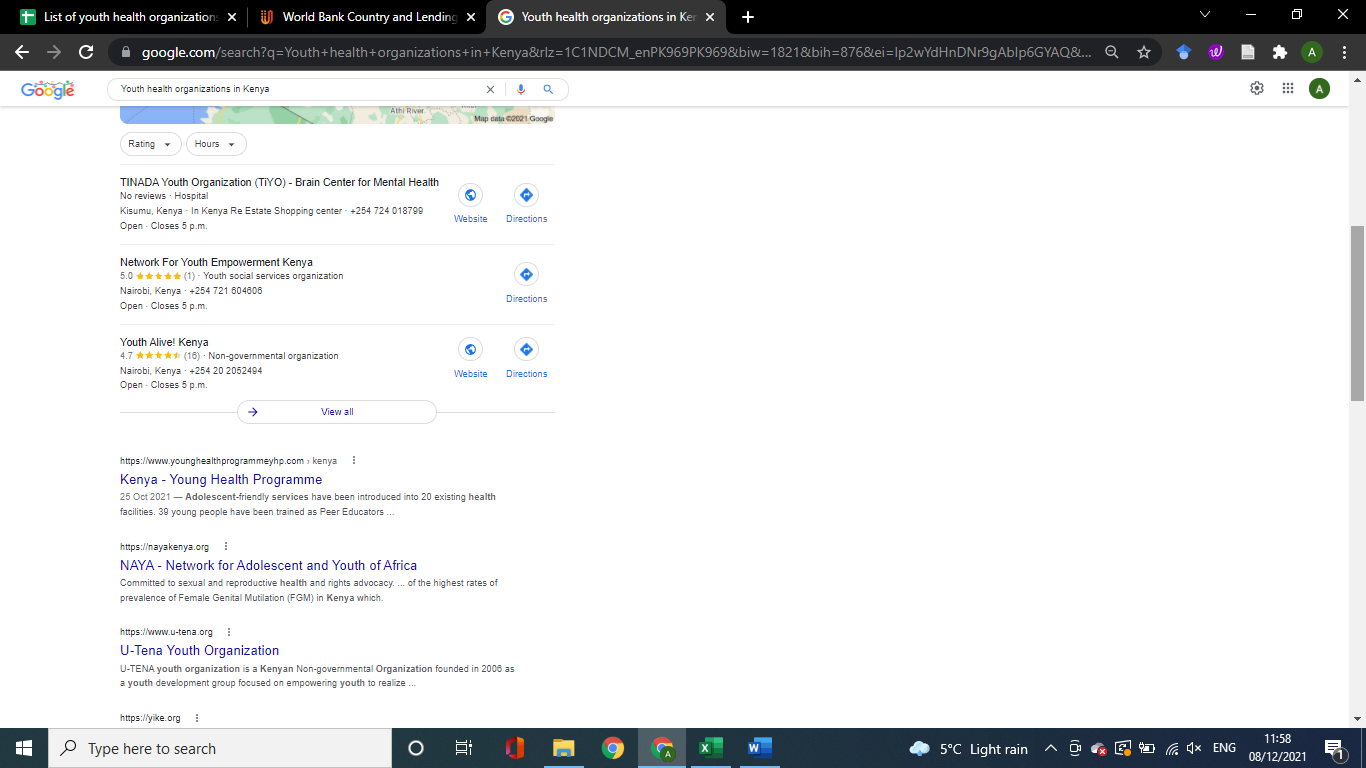 |
| 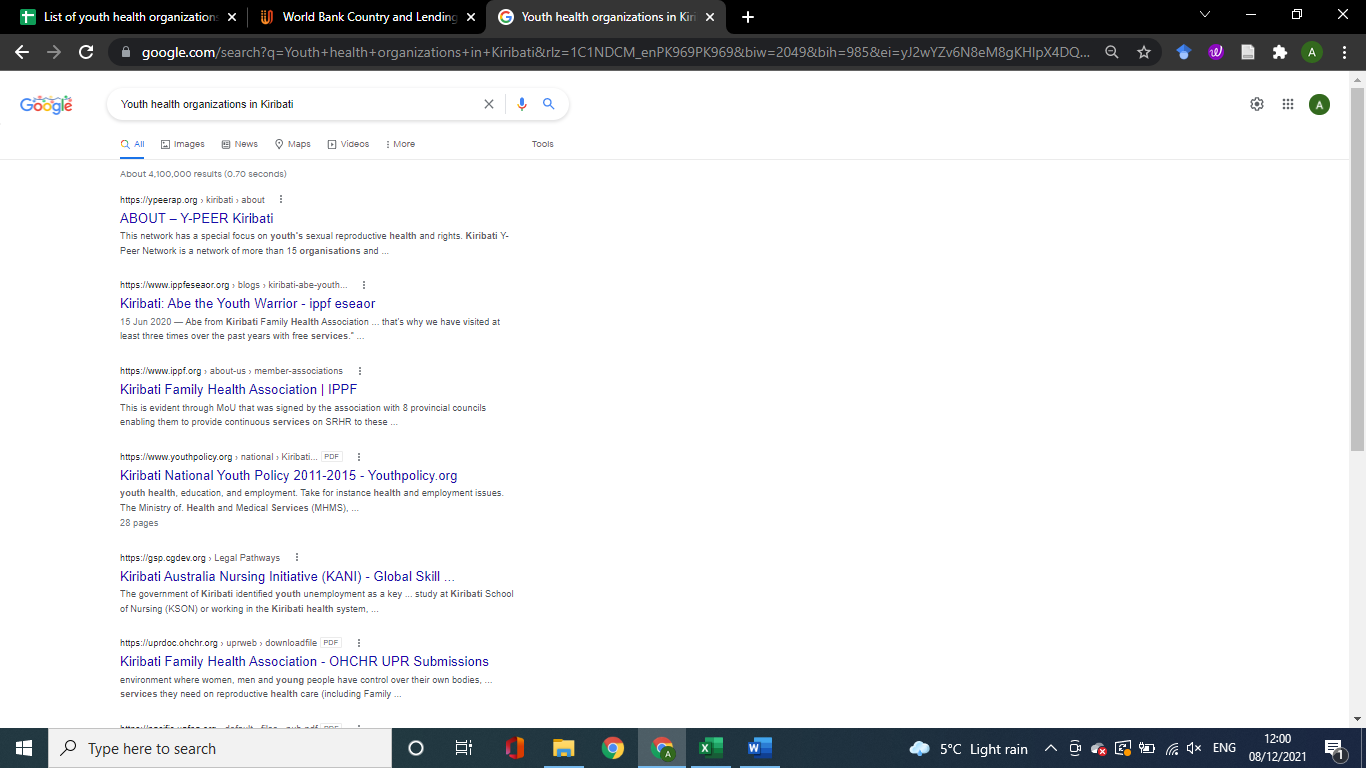 |
| 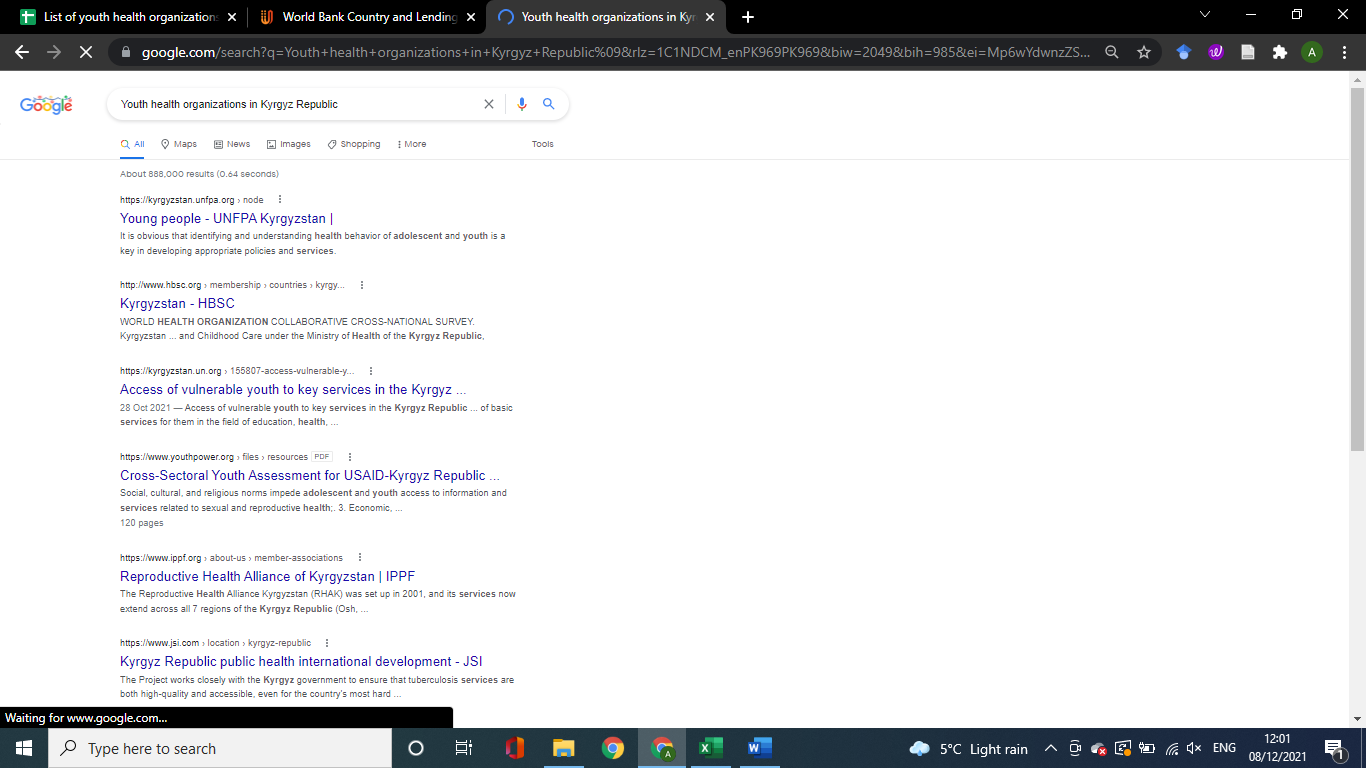 |
| 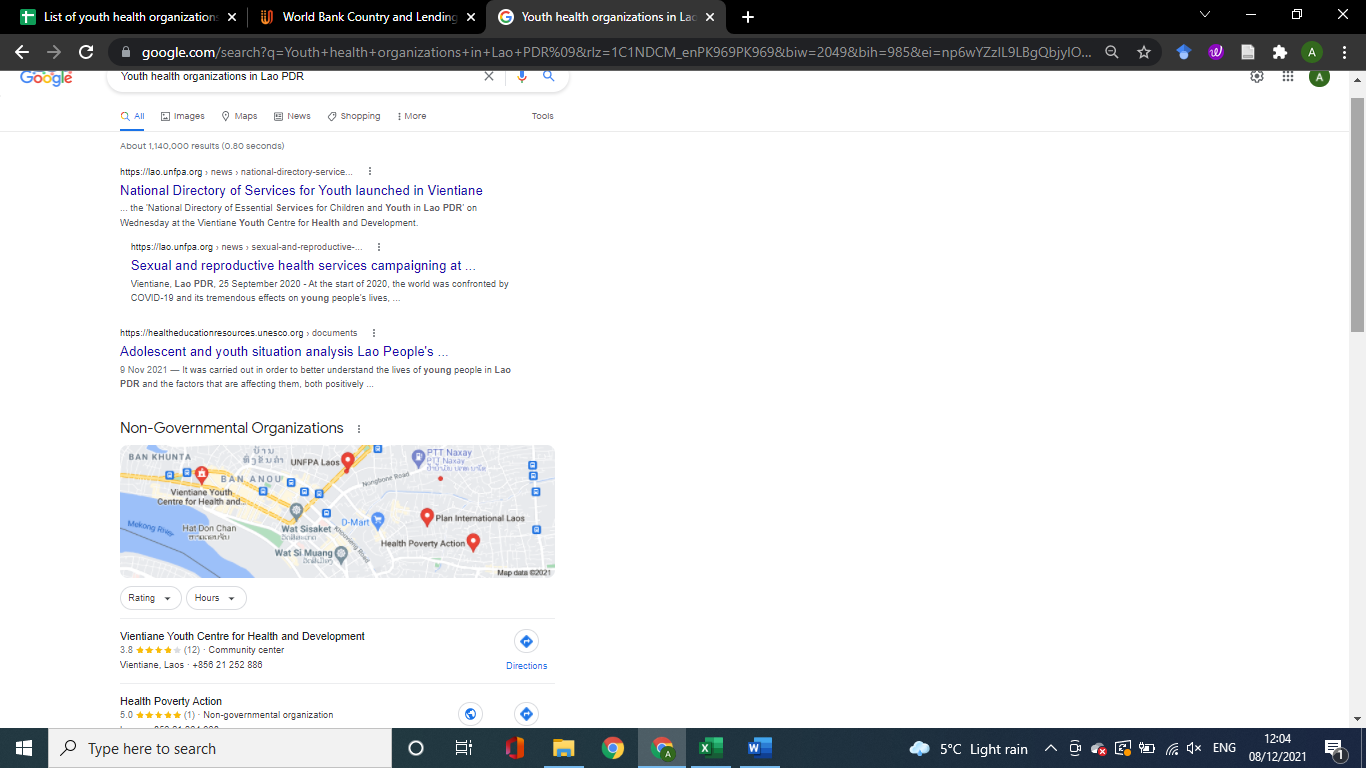 |
| 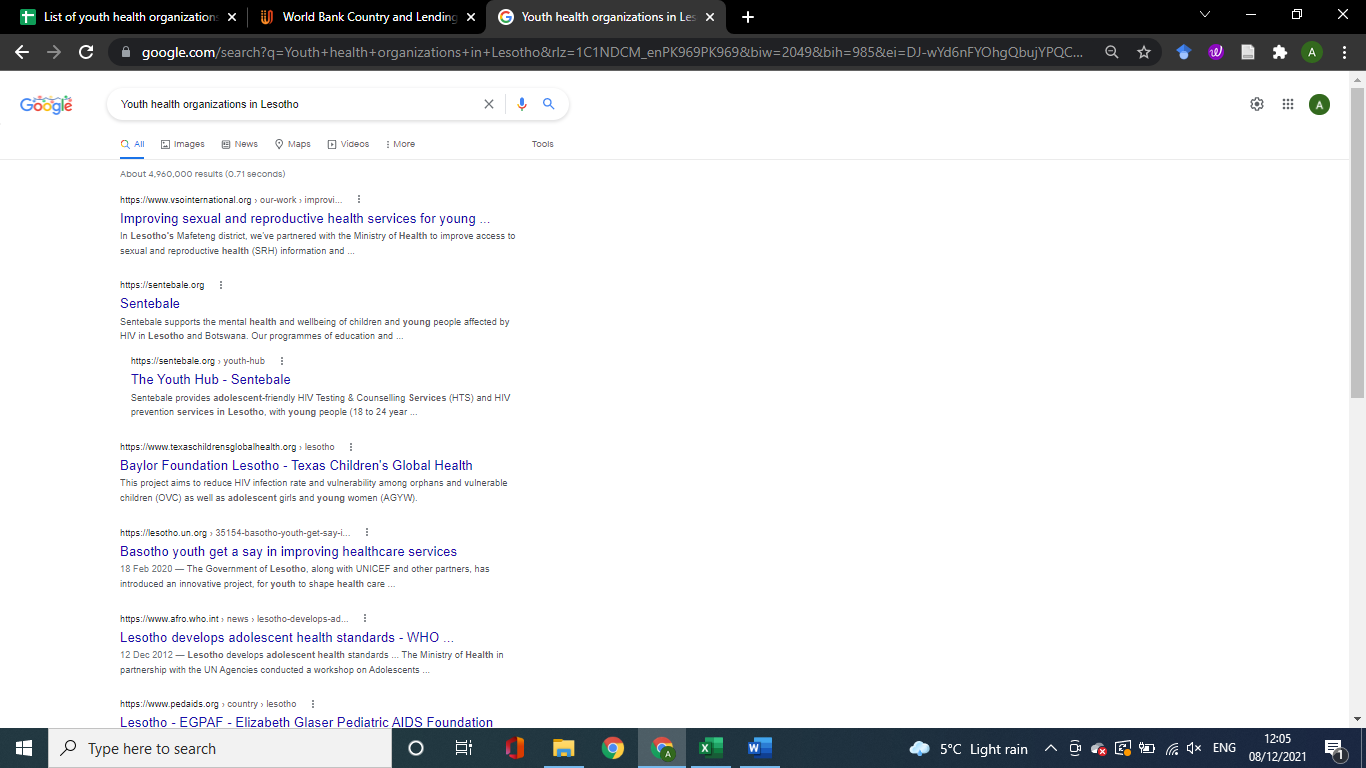 |
| 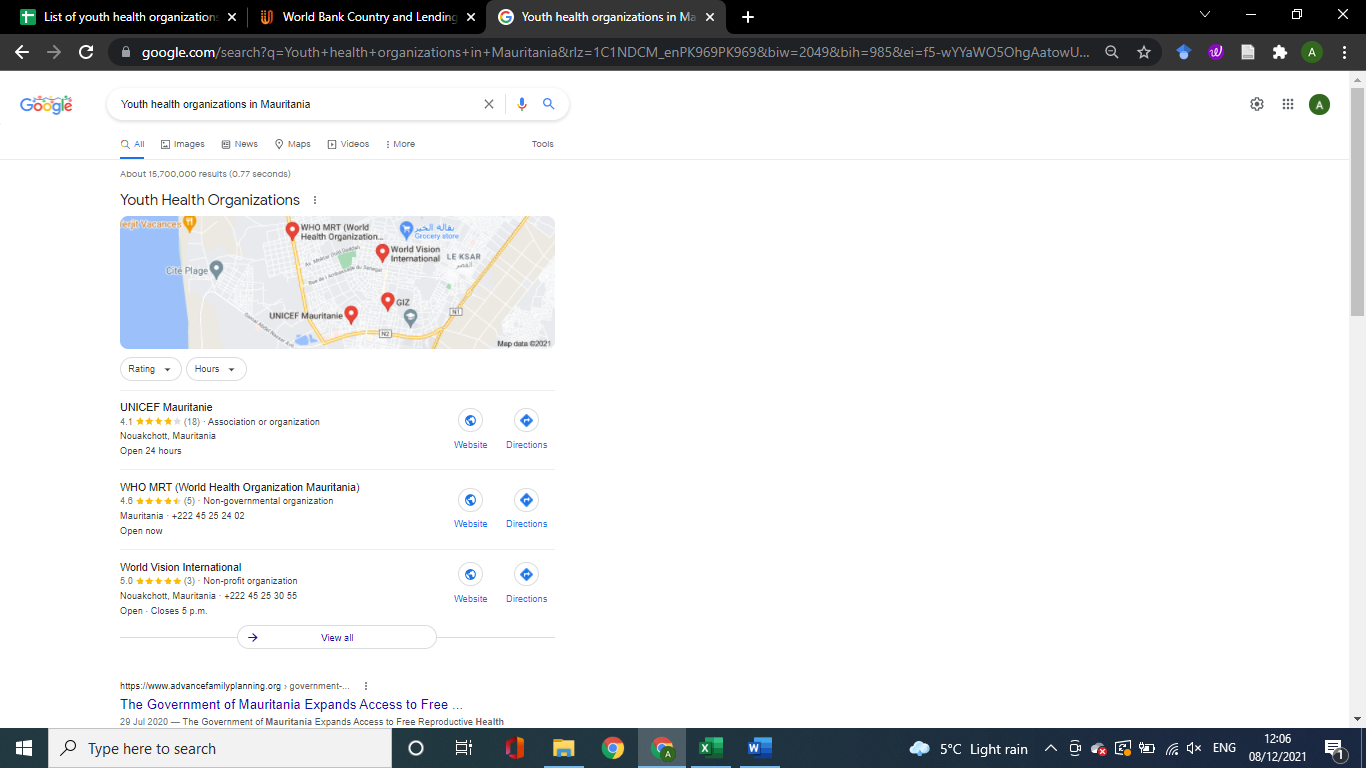 |
| 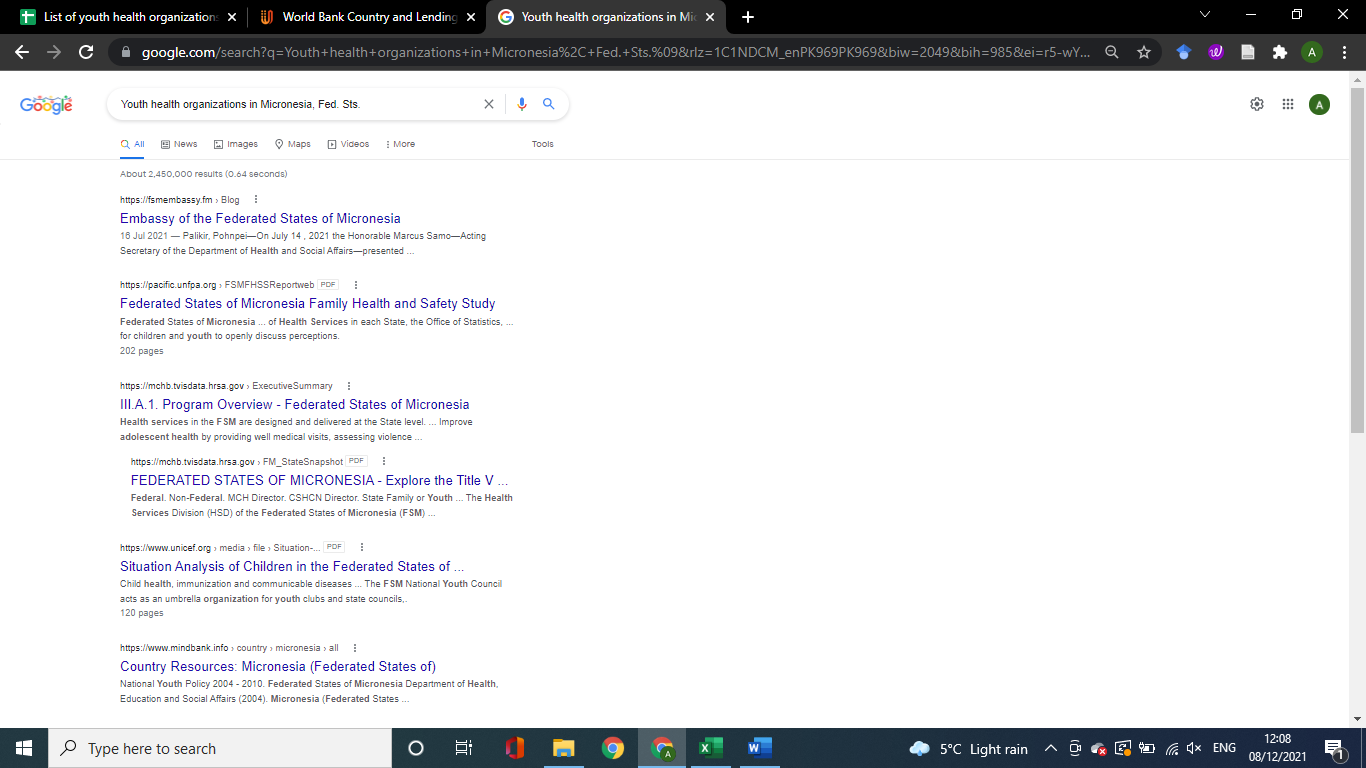 |
| 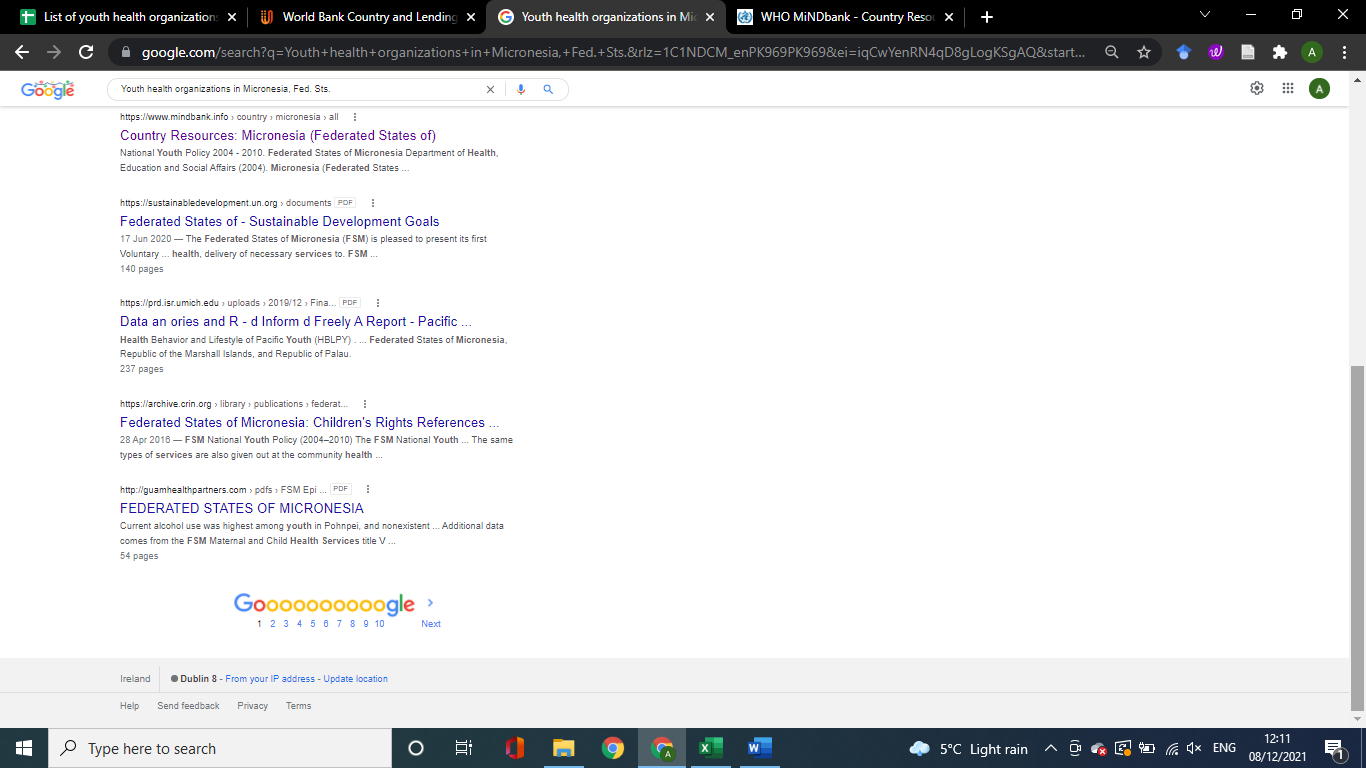 |
| 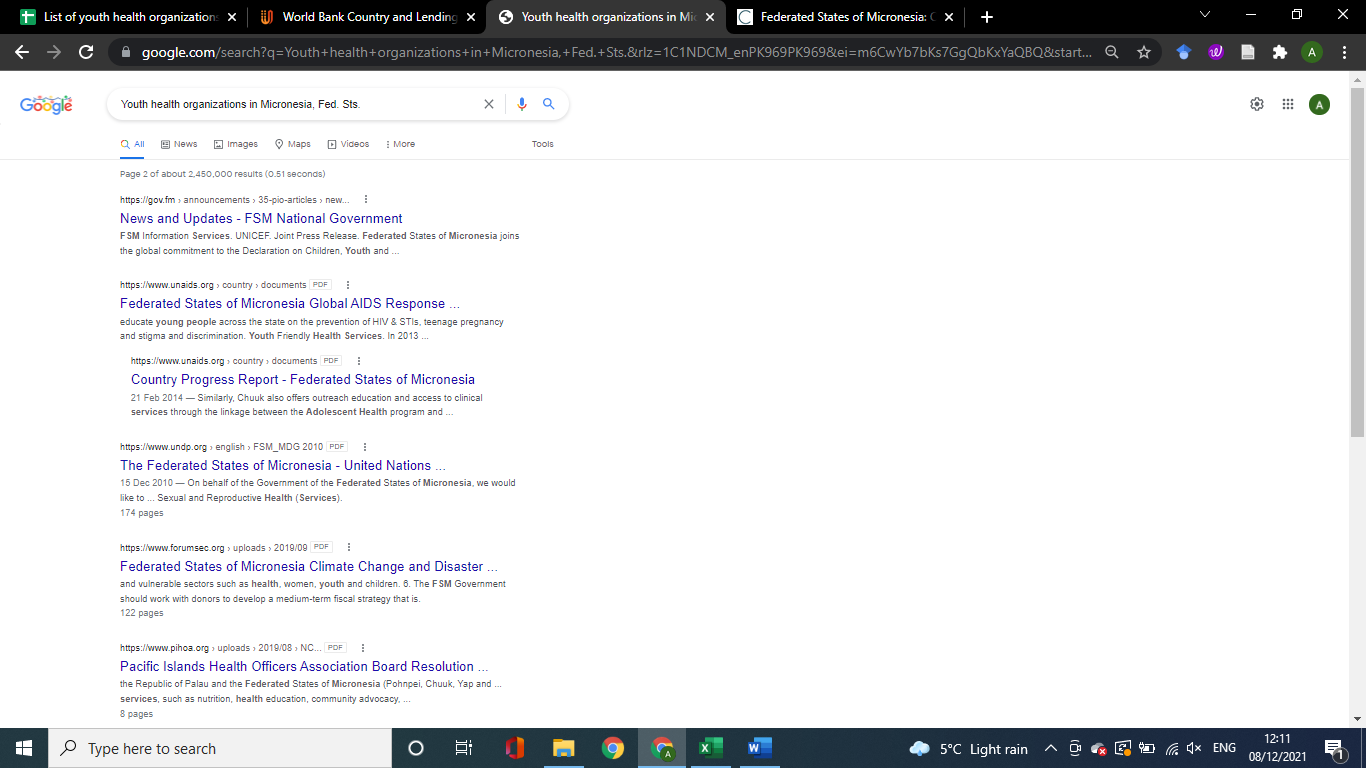 |
| 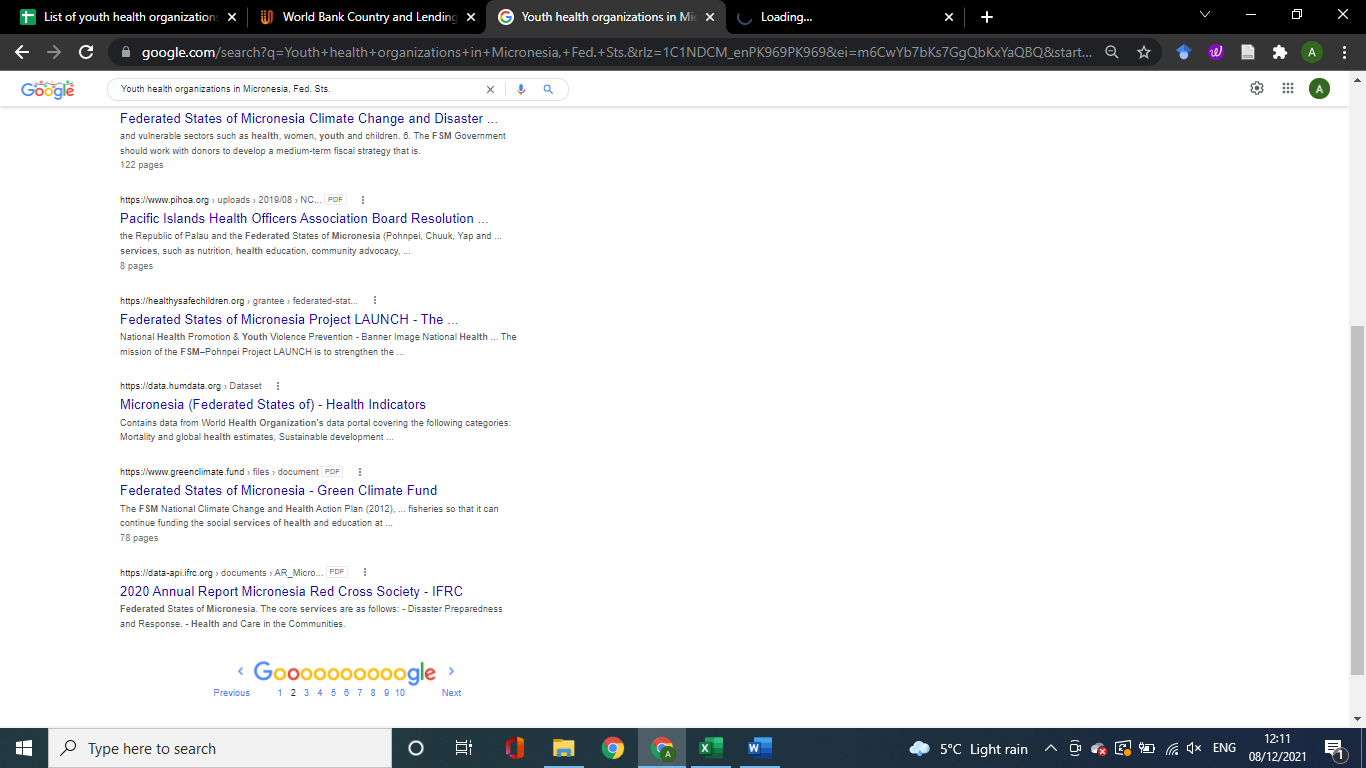 |
| 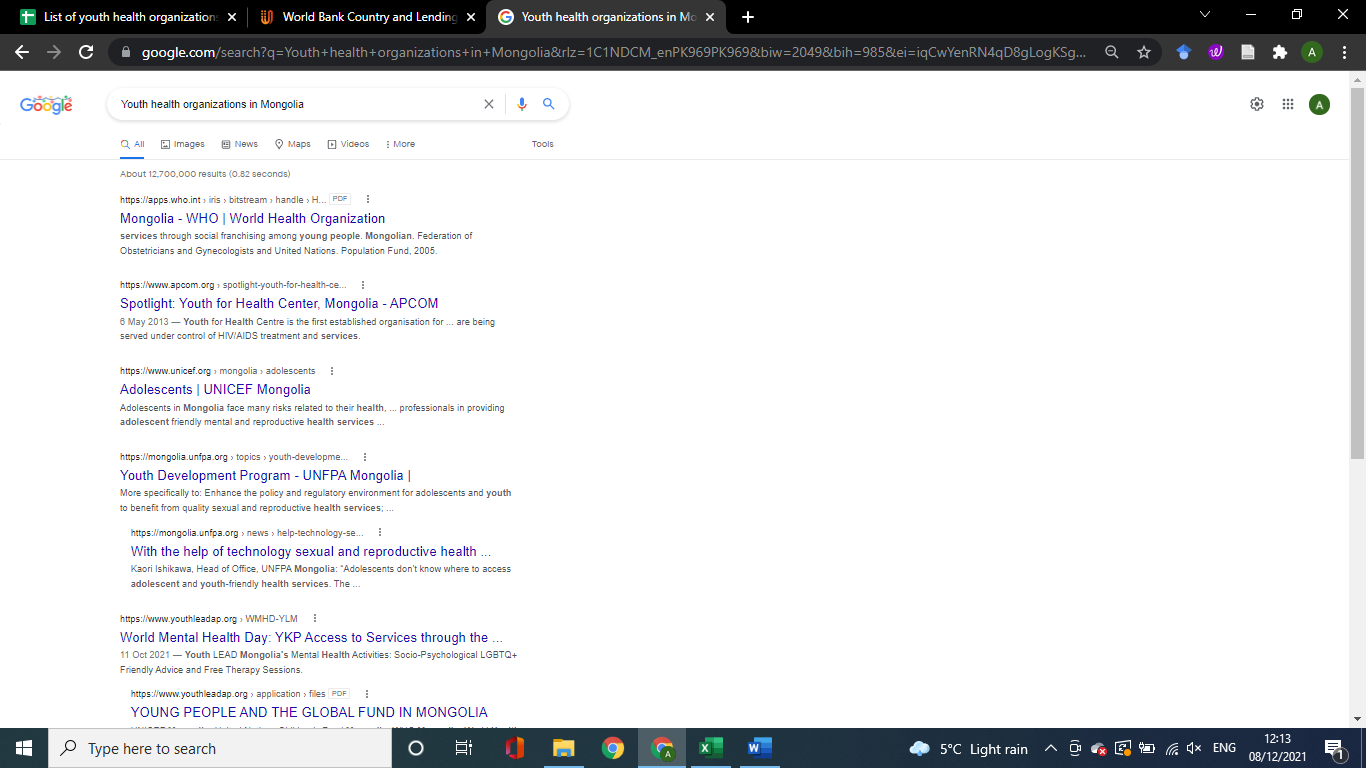 |
| 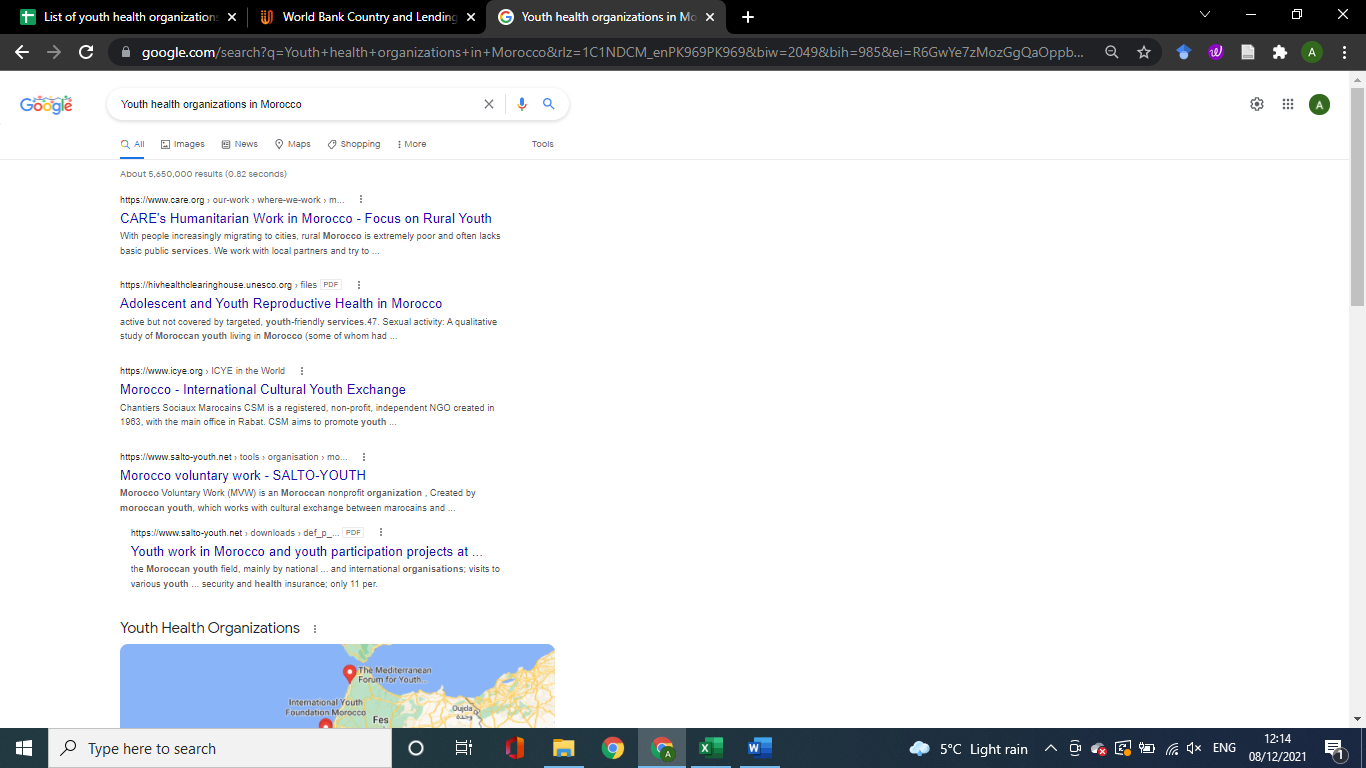 |
| 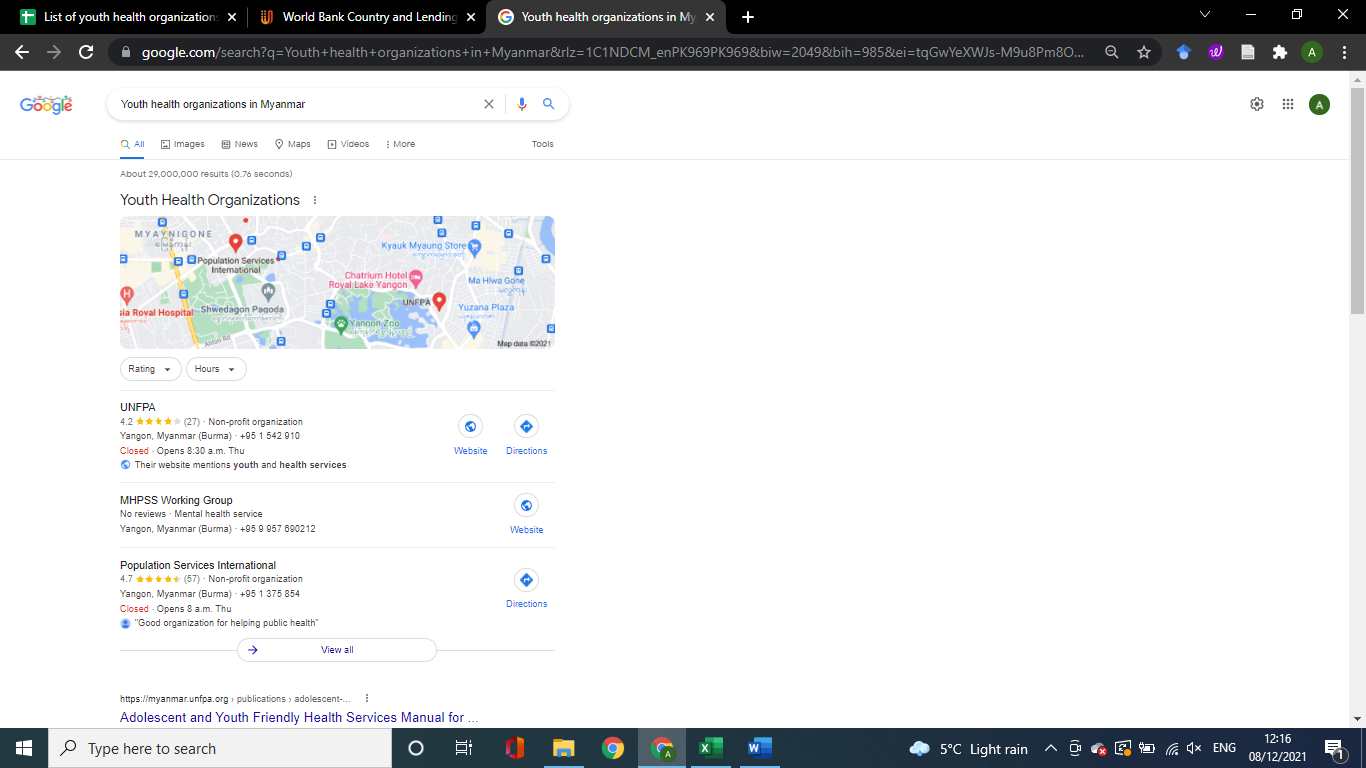 |
| 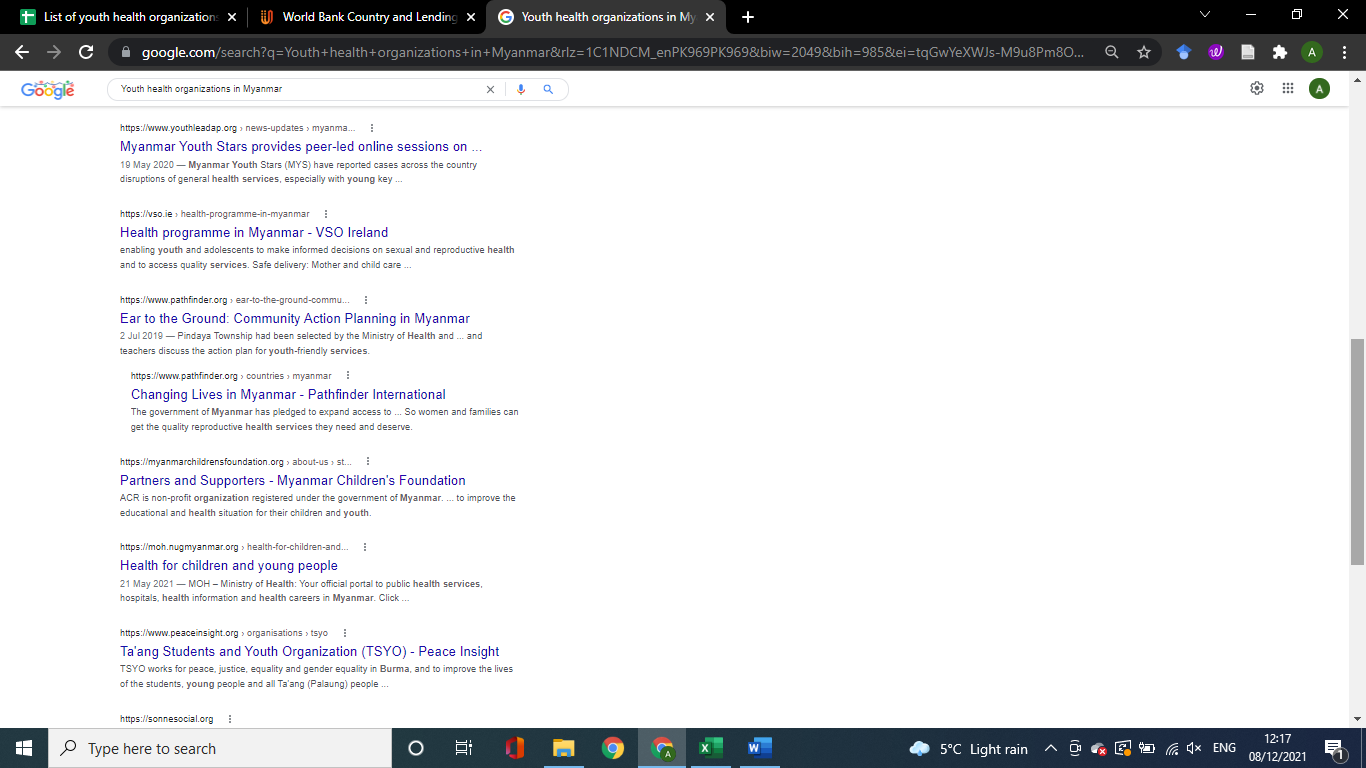 |
| 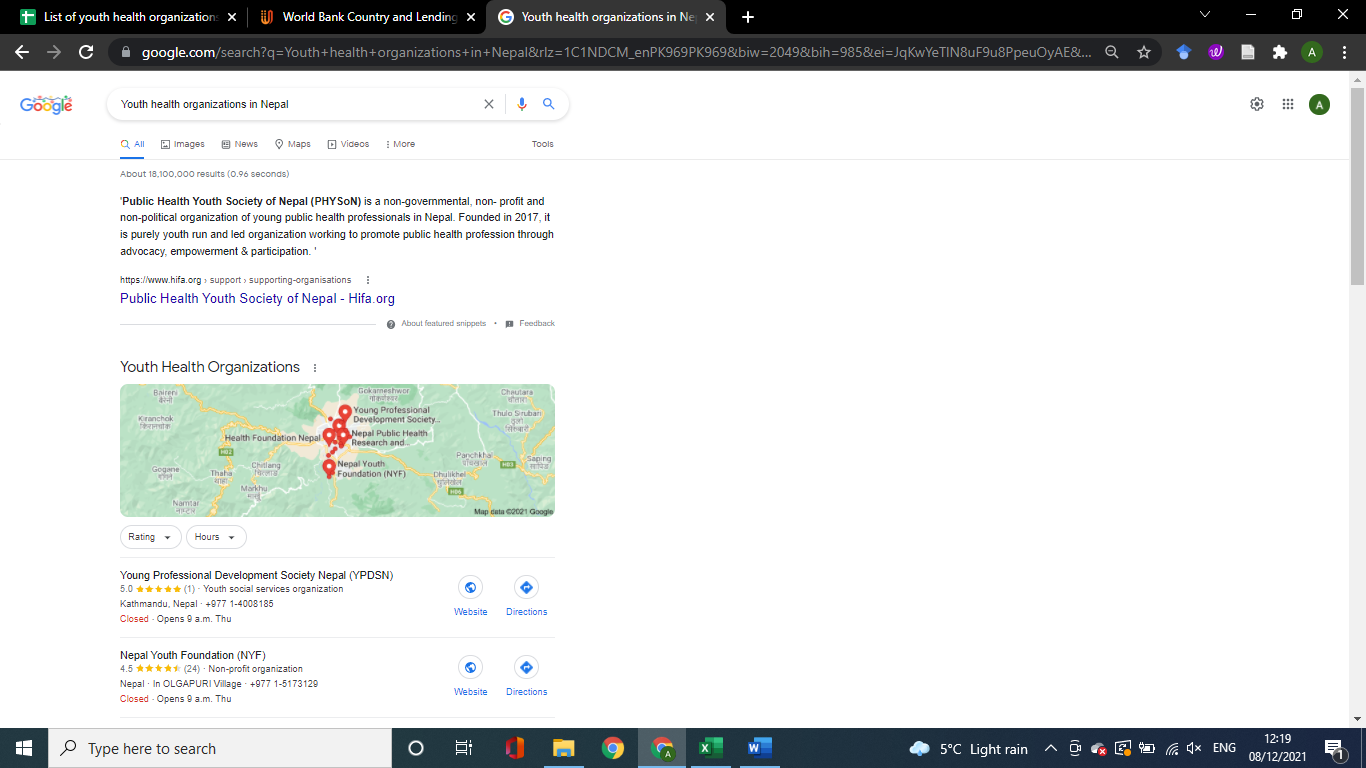 |
| 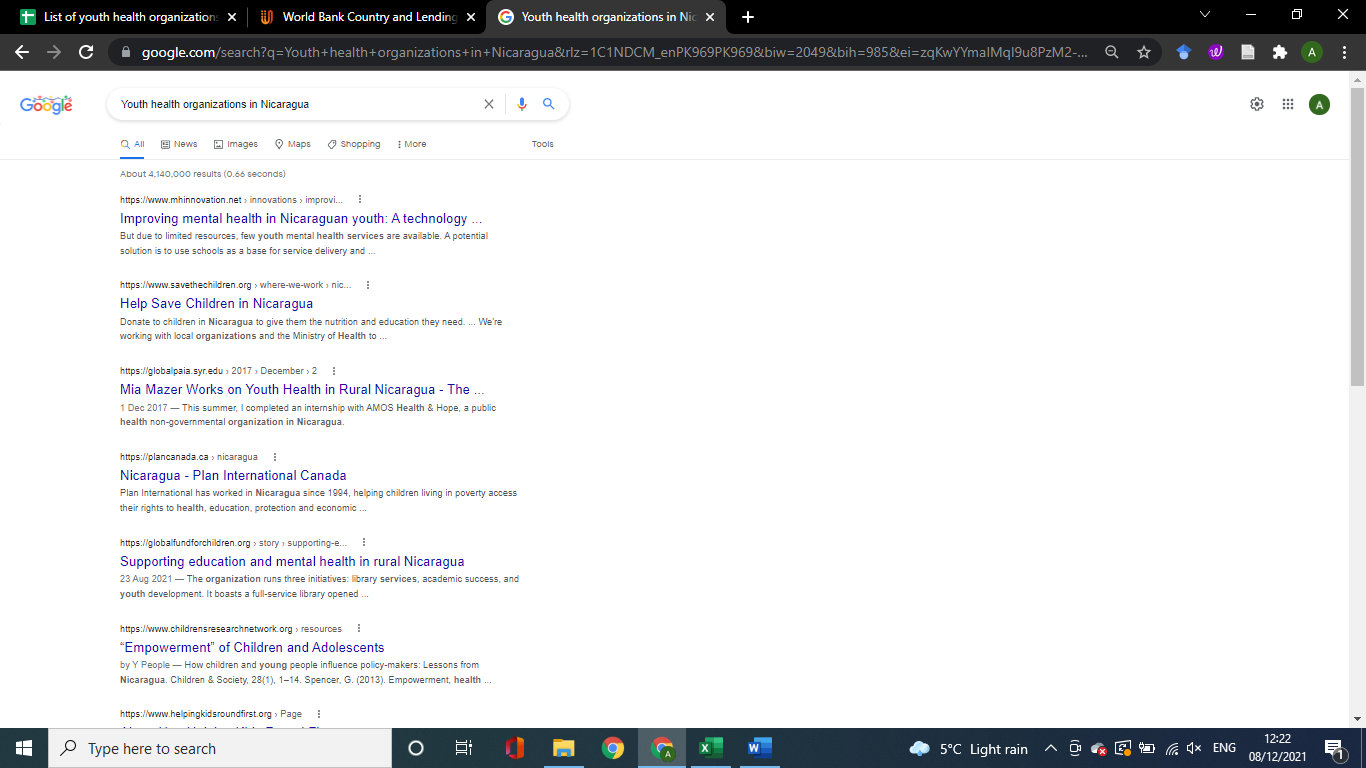 |
| 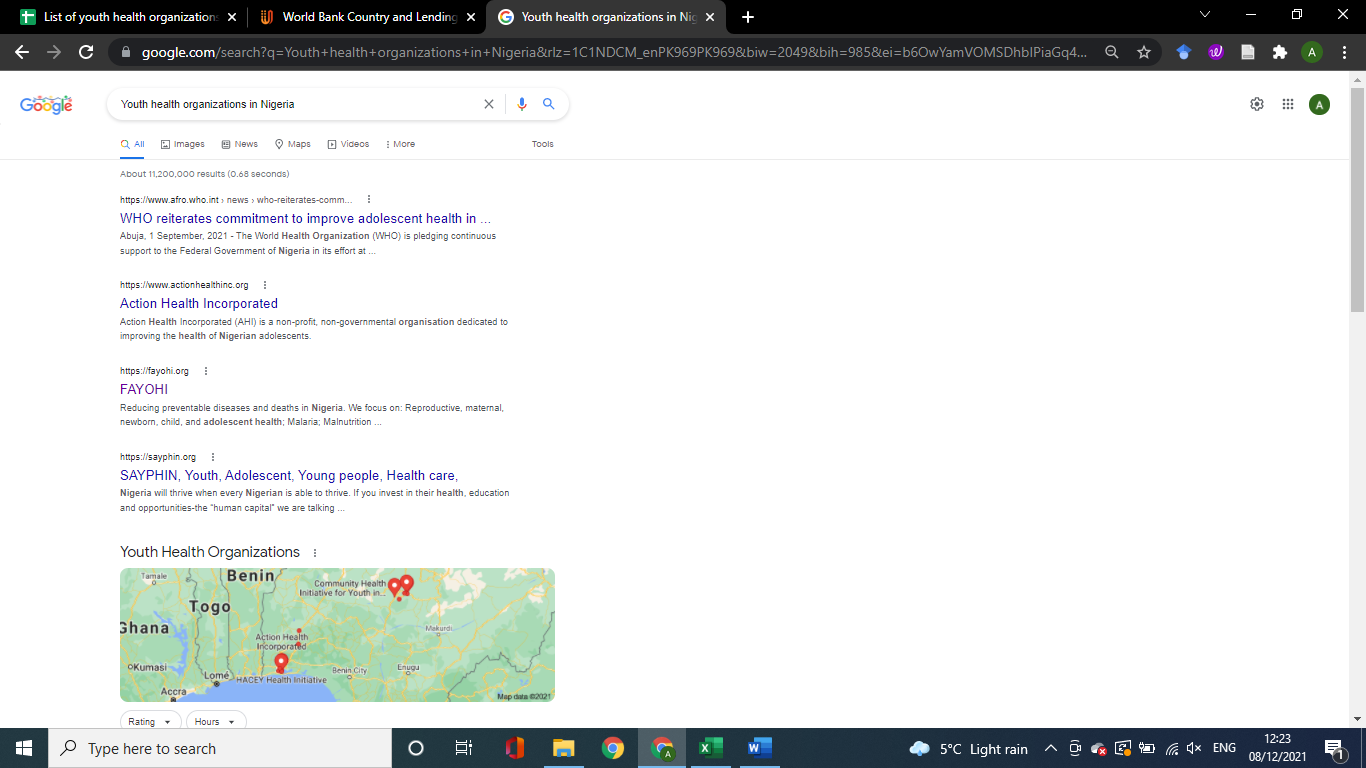 |
| 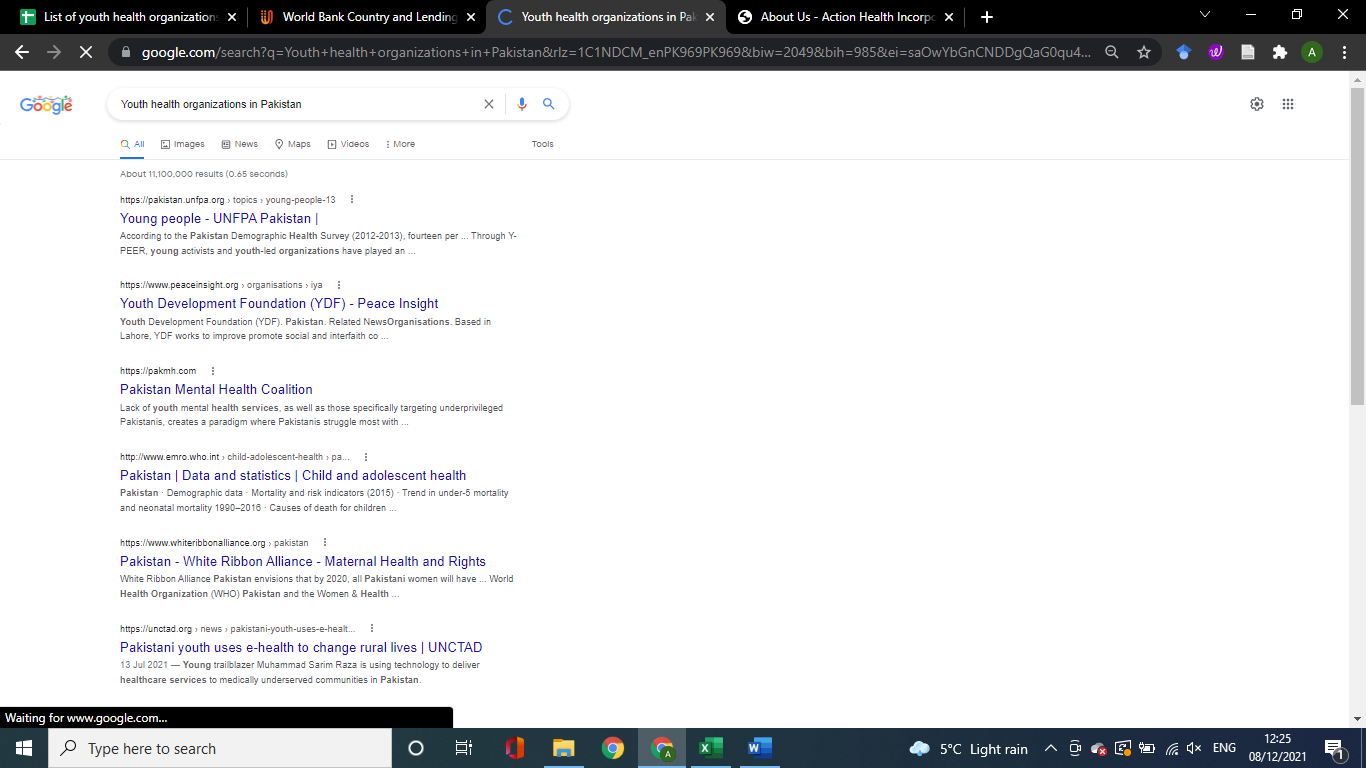 |
| 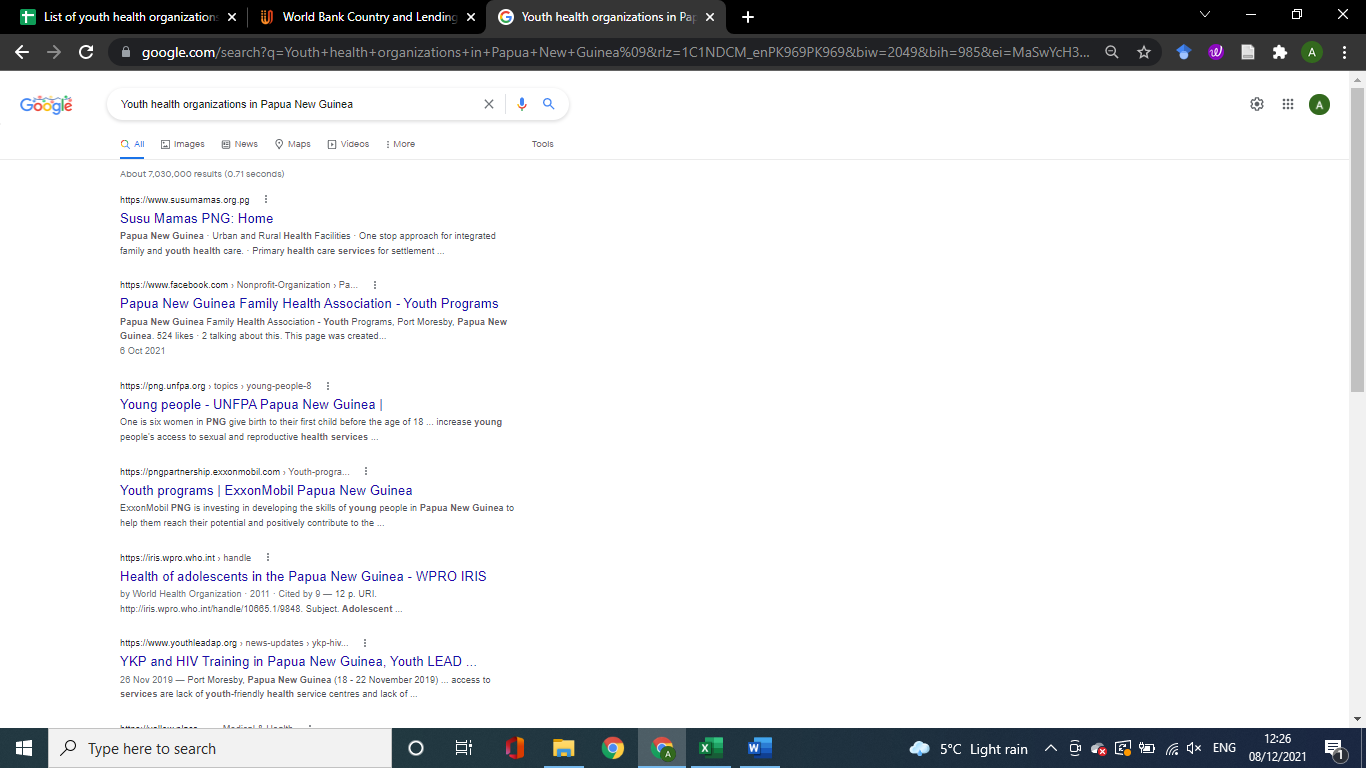 |
| 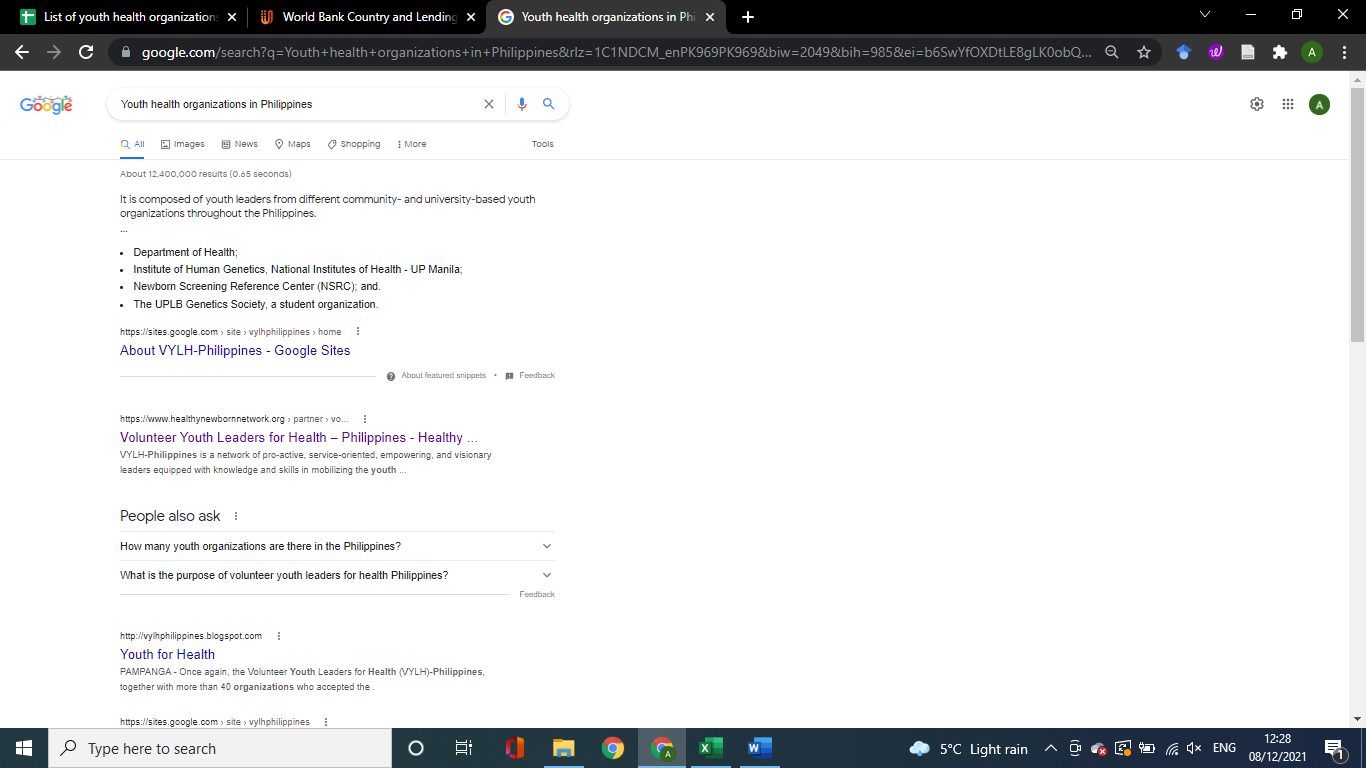 |
| 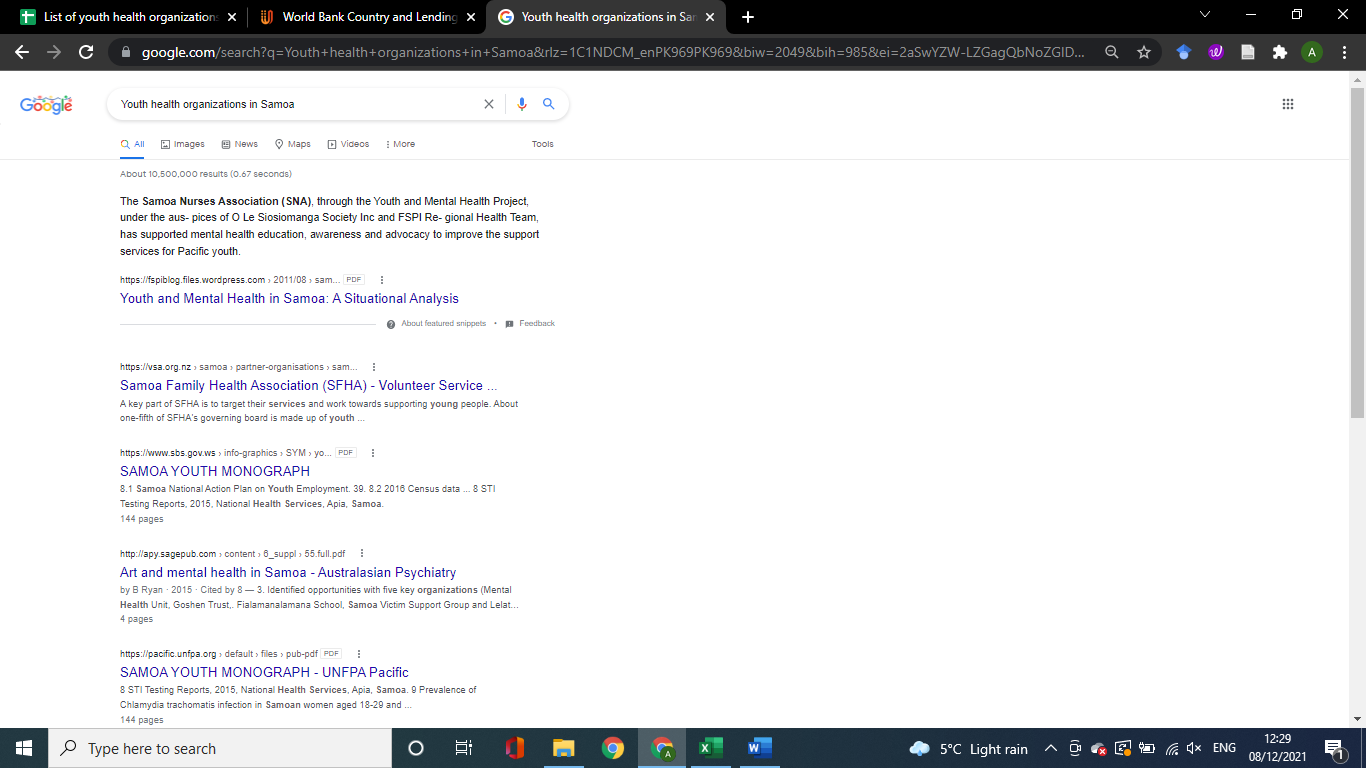 |
| 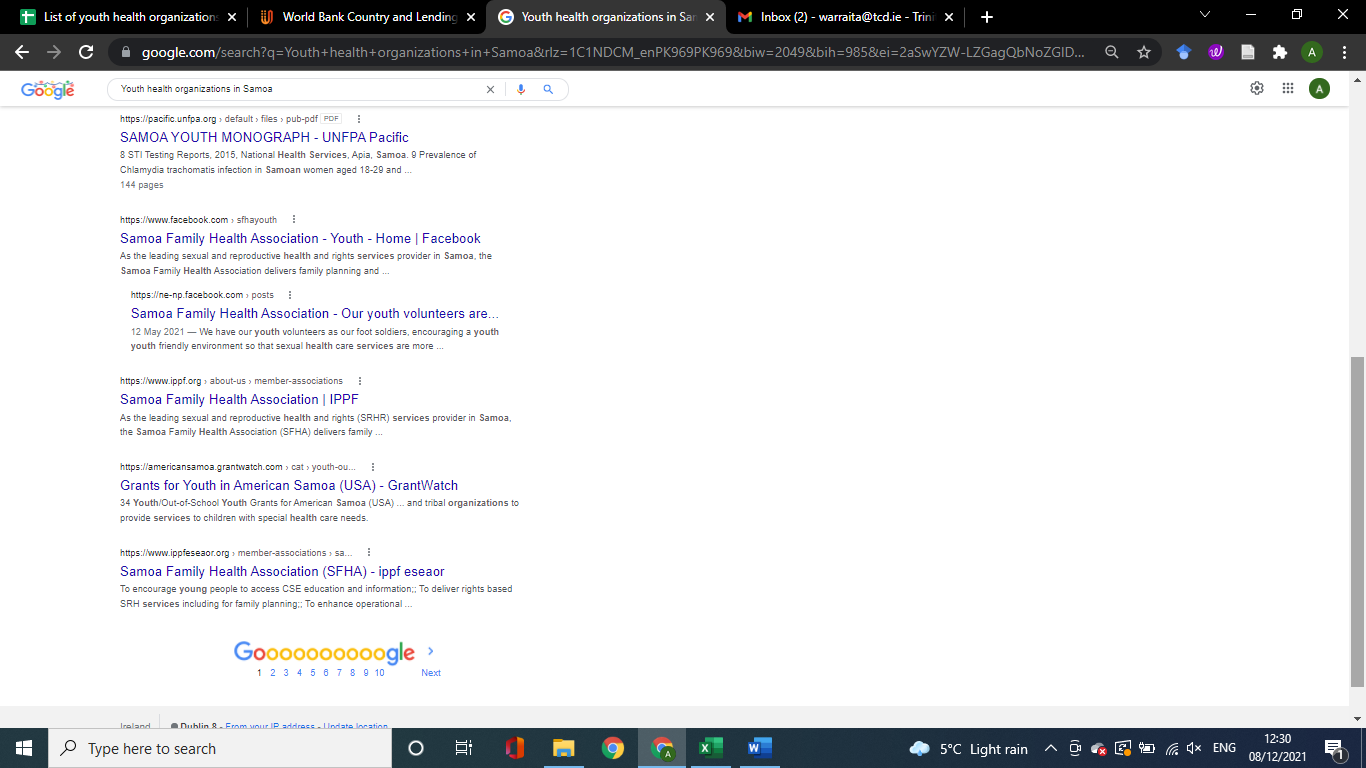 |
| 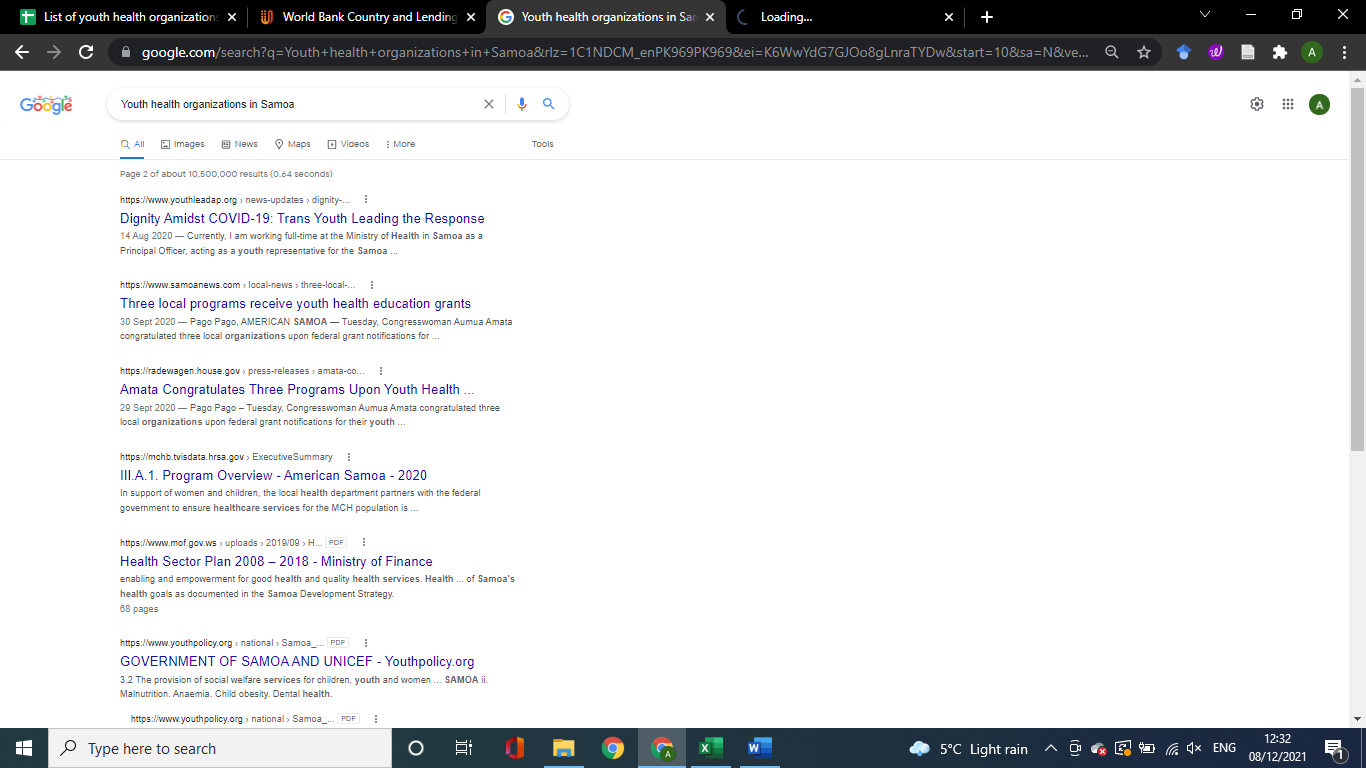 |
| 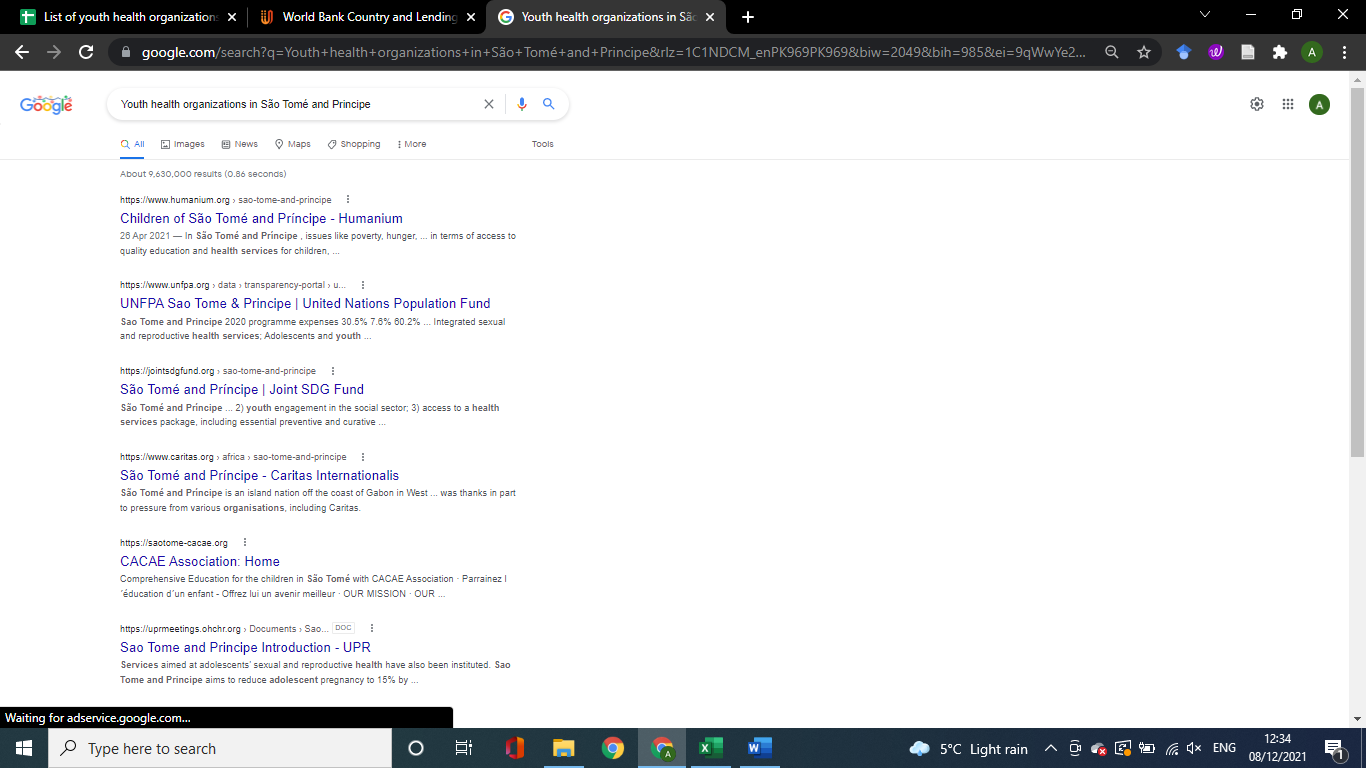 |
| 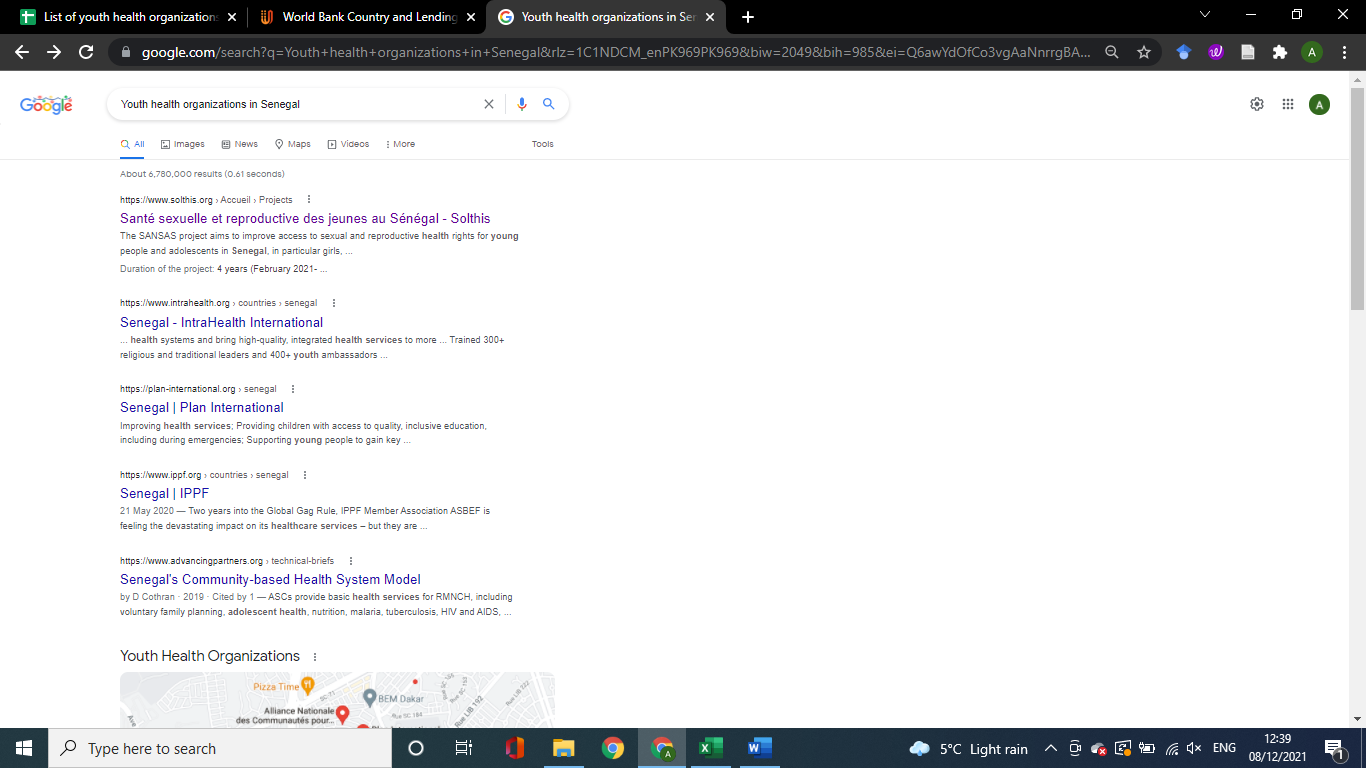 |
| 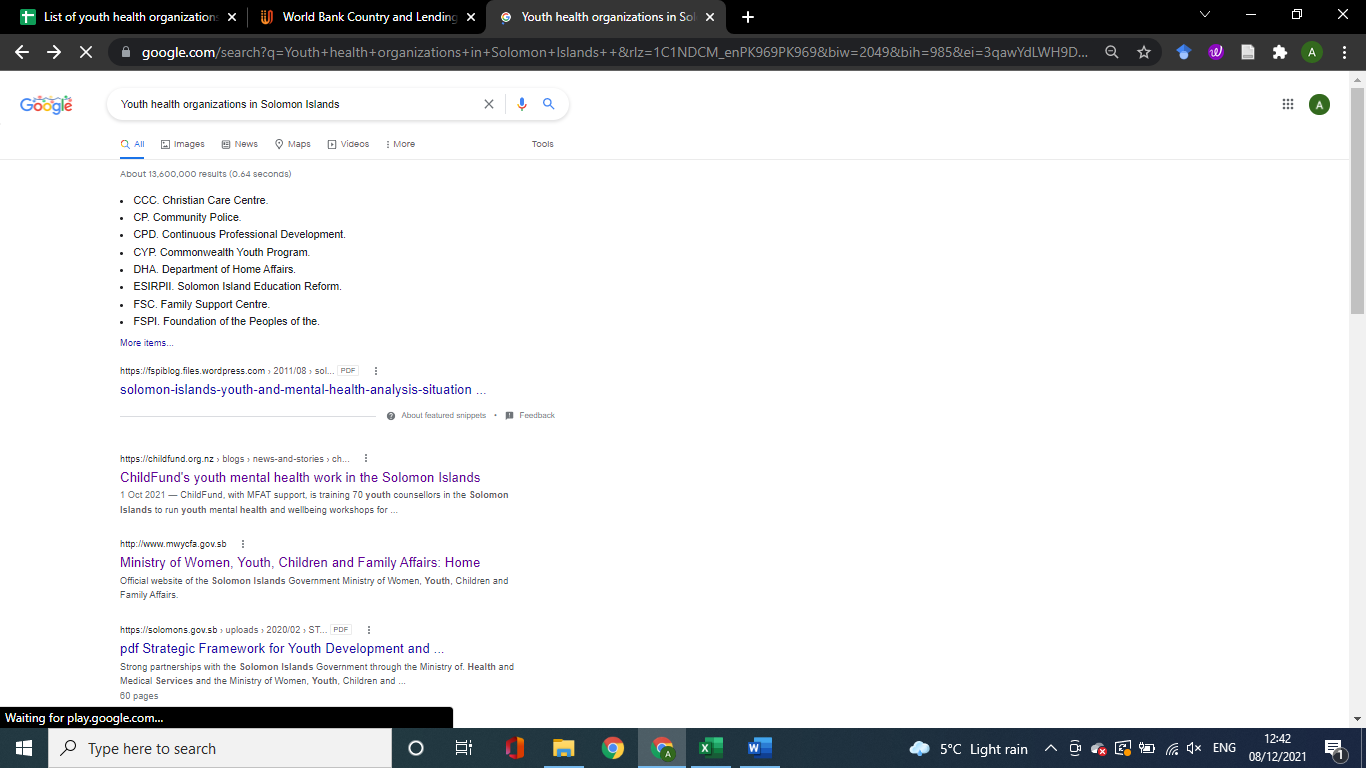 |
| 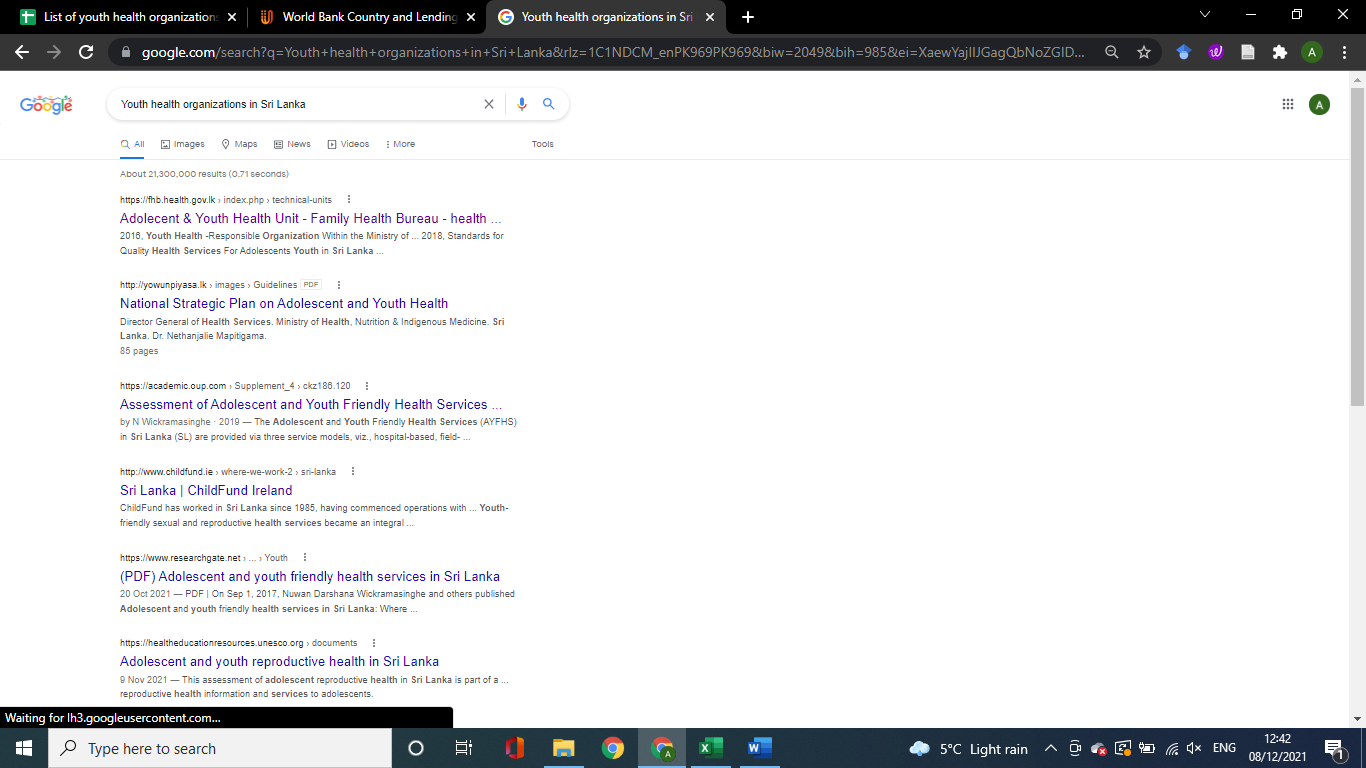 |
| 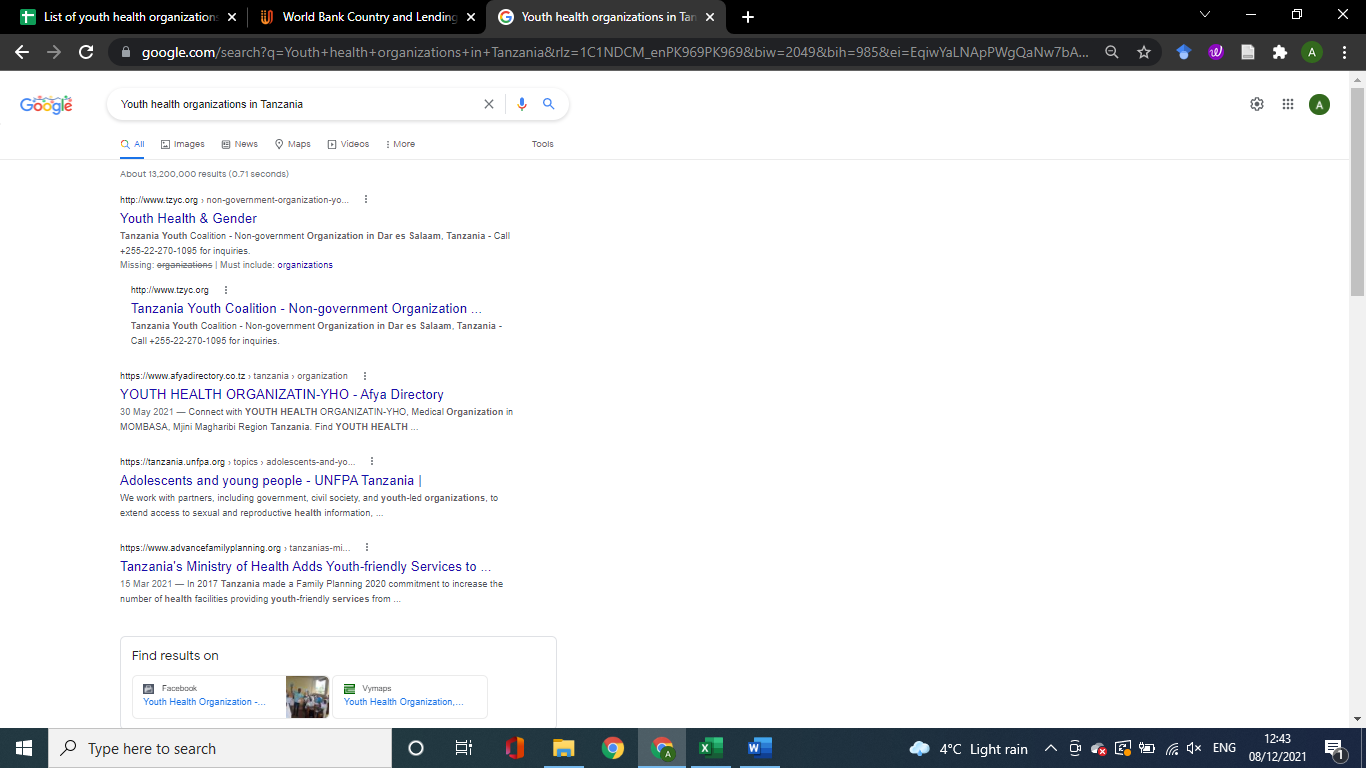 |
| 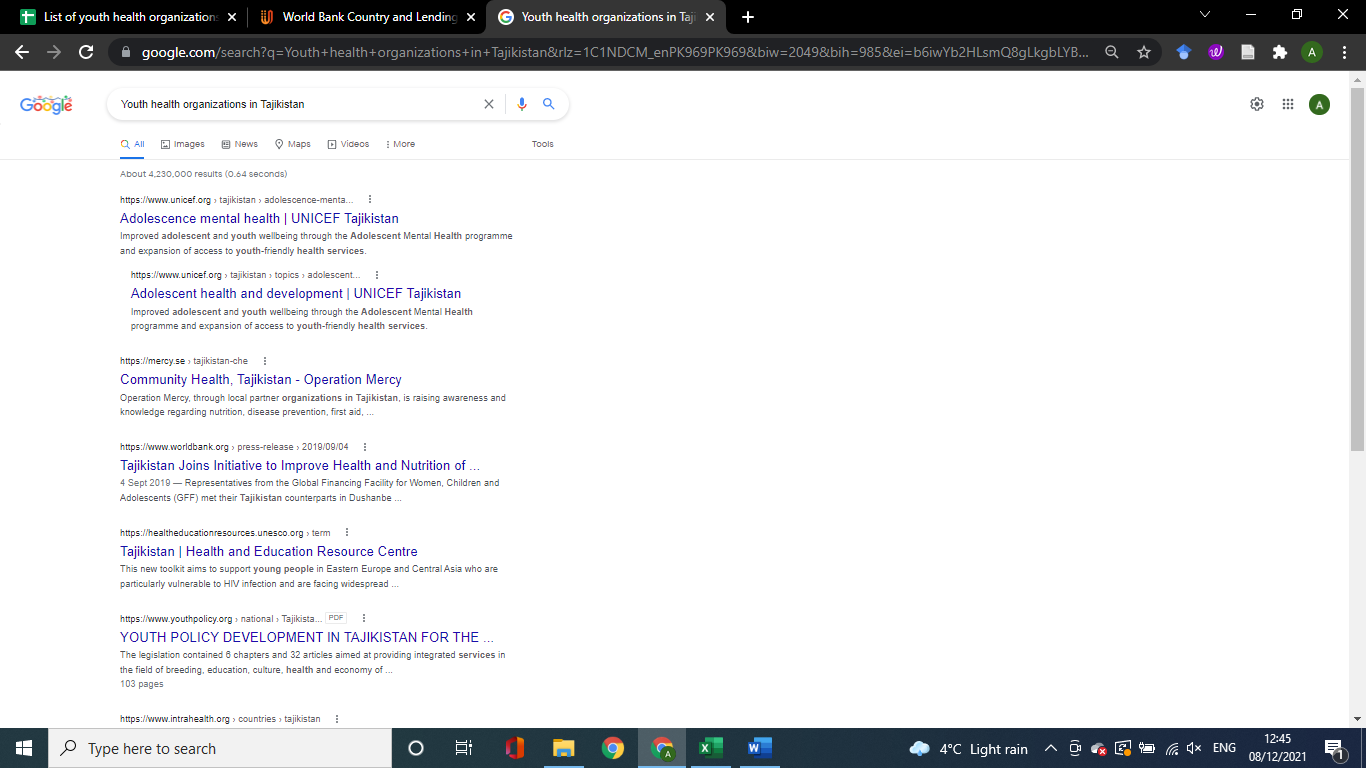 |
| 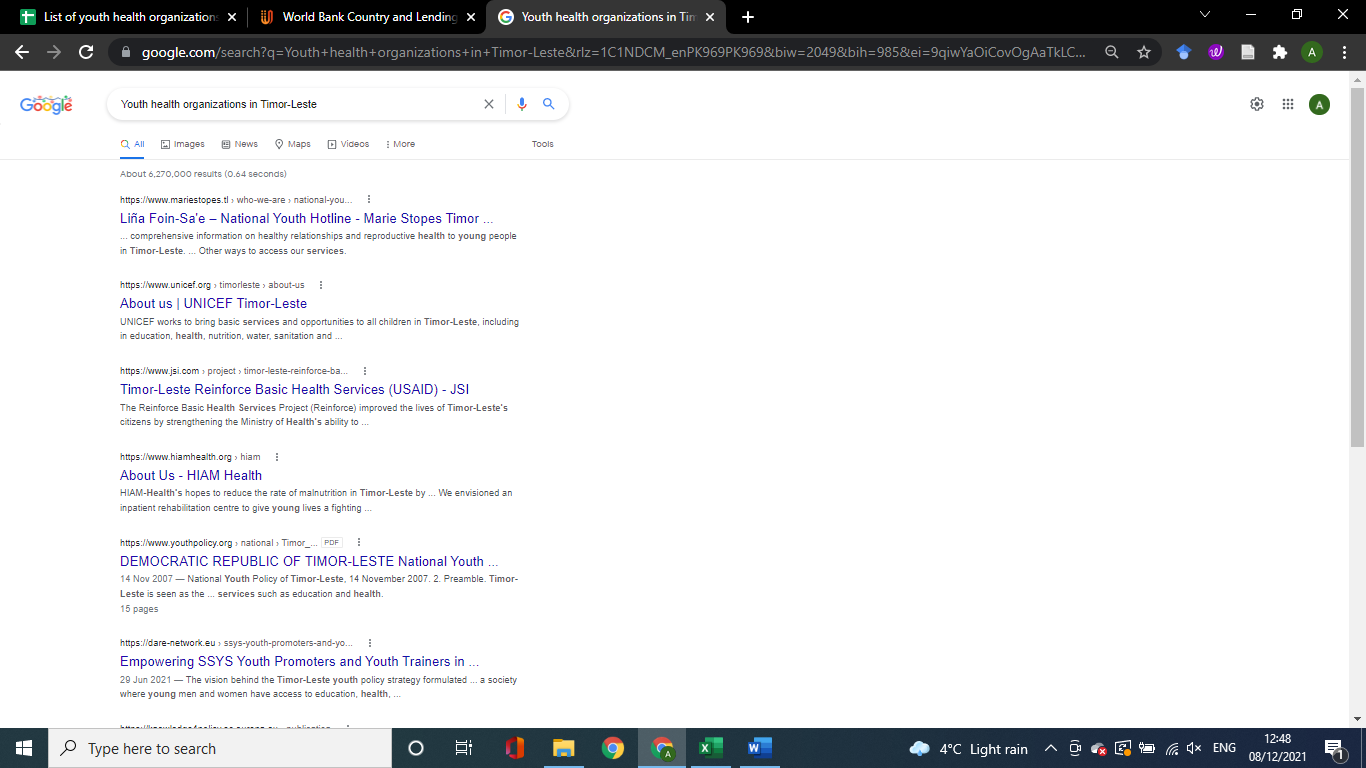 |
| 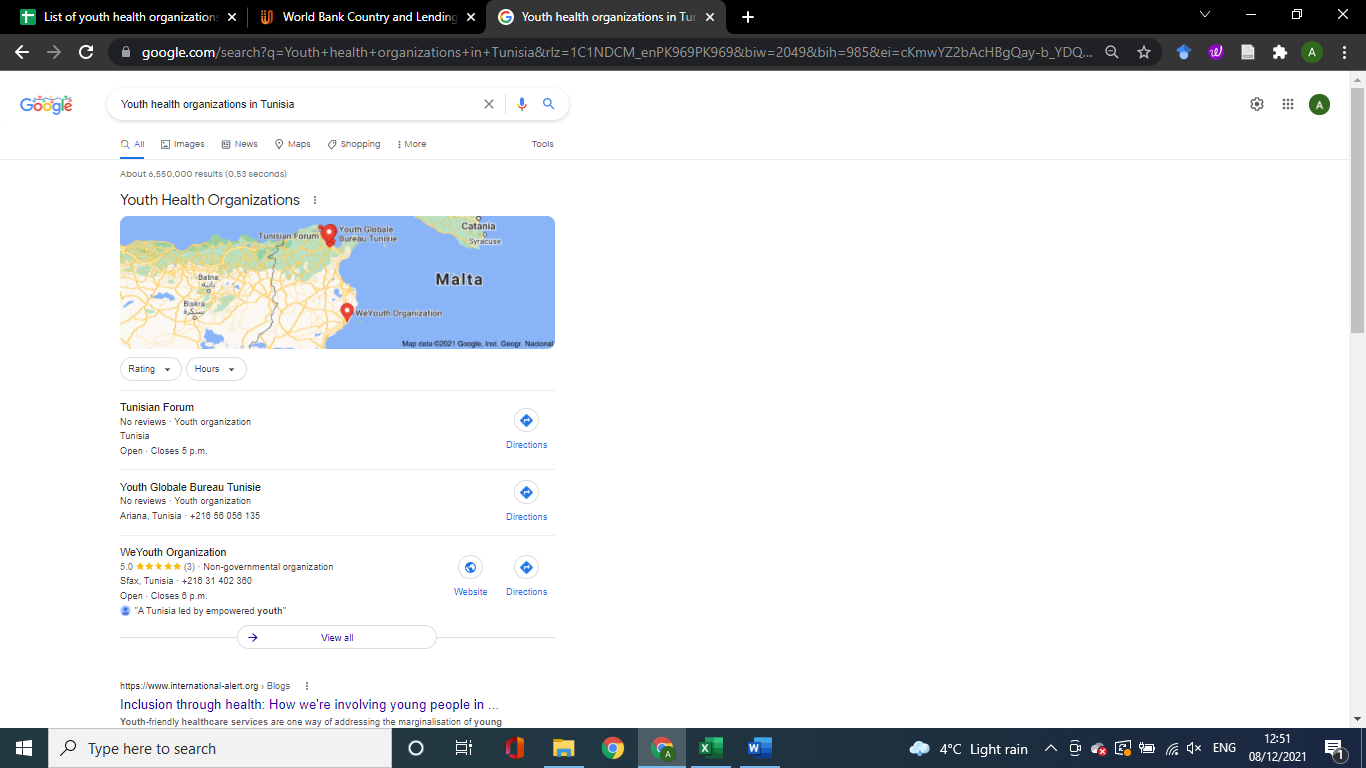 |
| 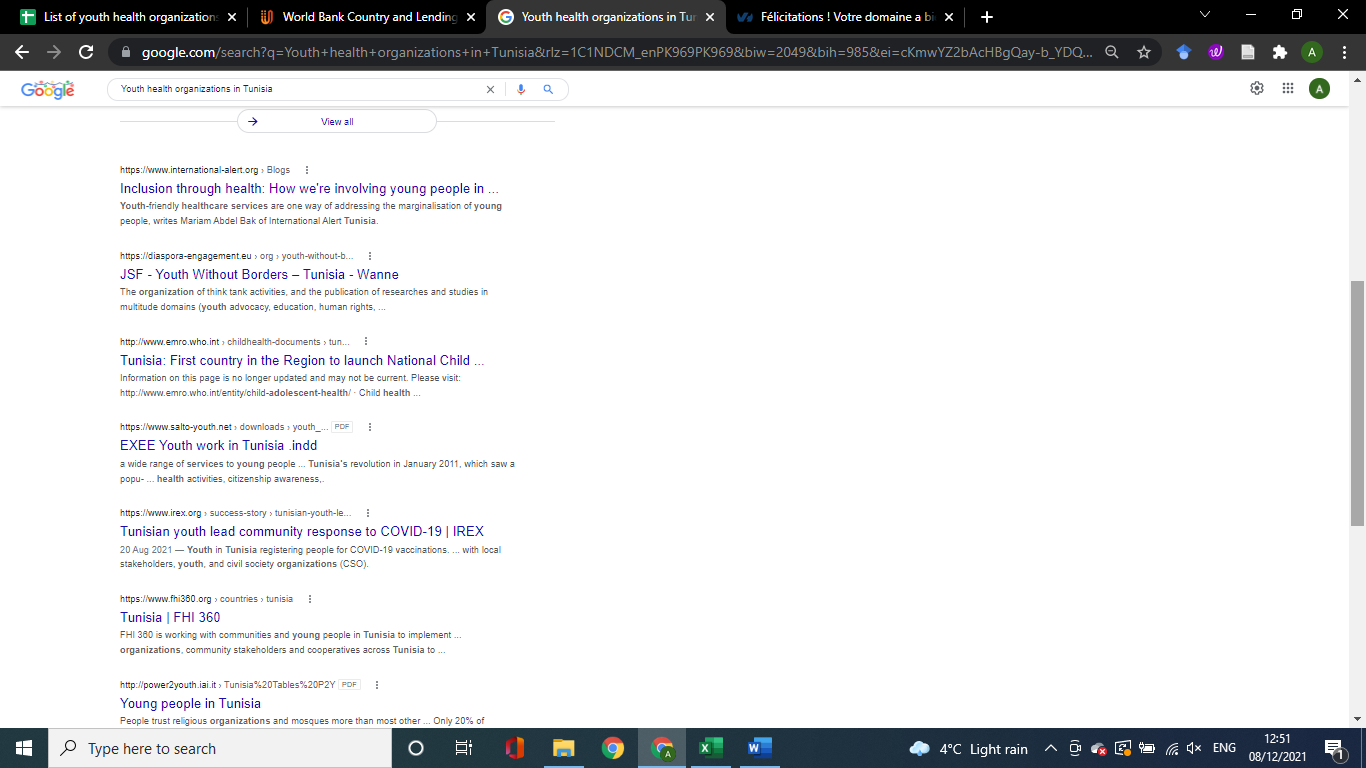 |
| 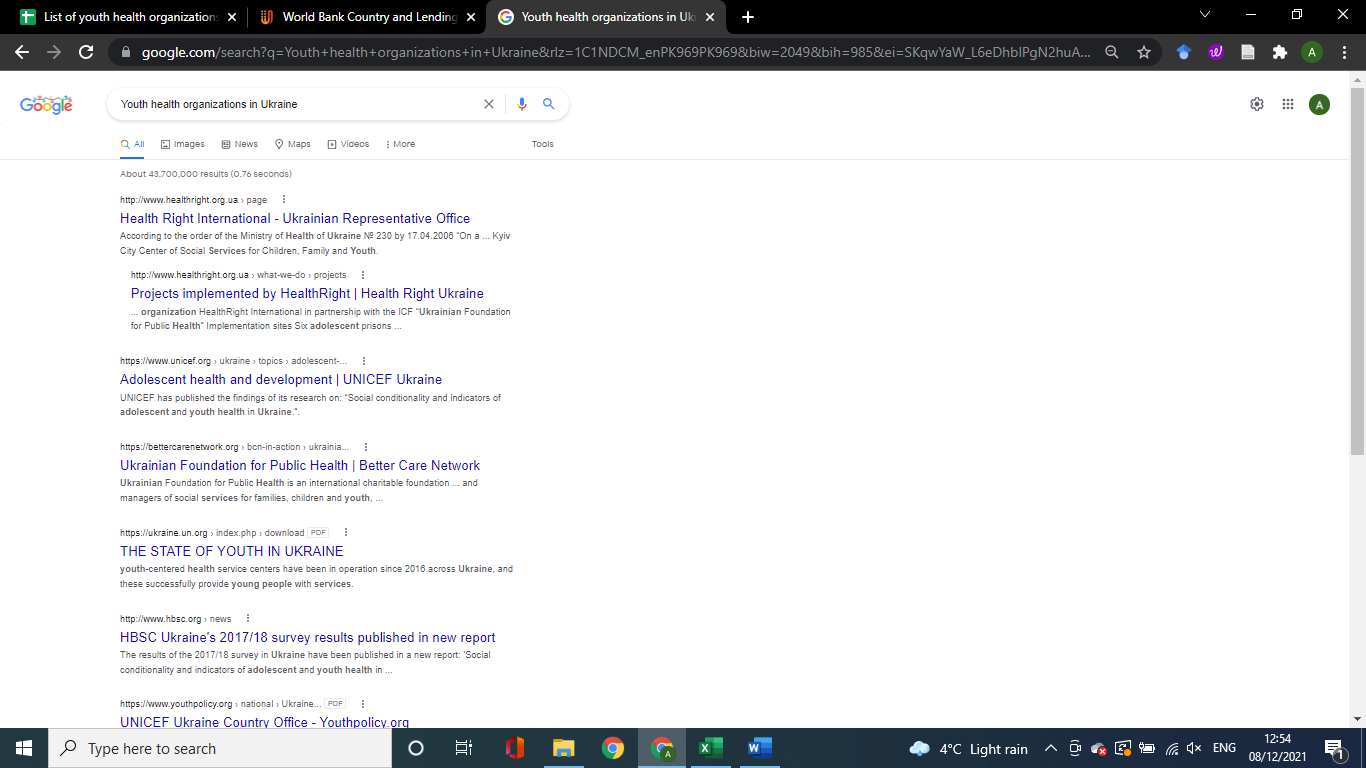 |
| 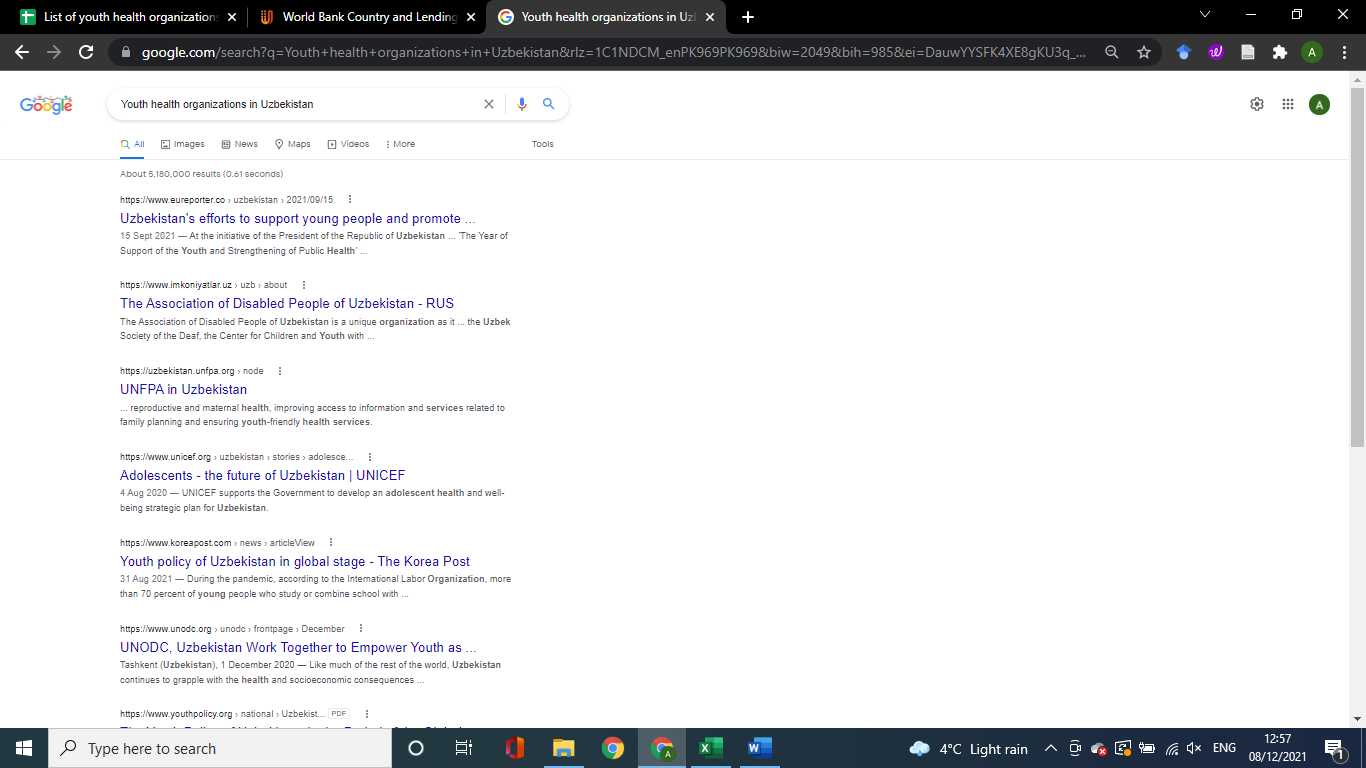 |
| 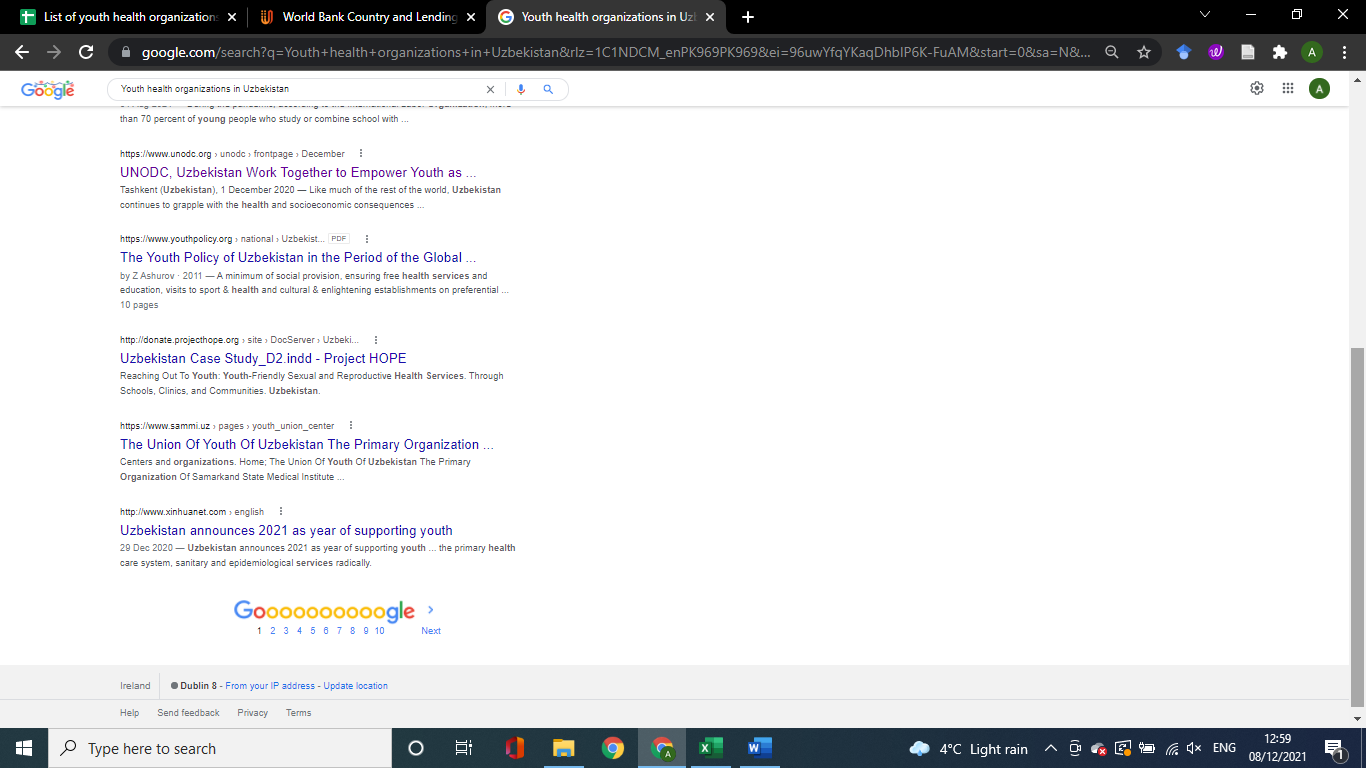 |
| 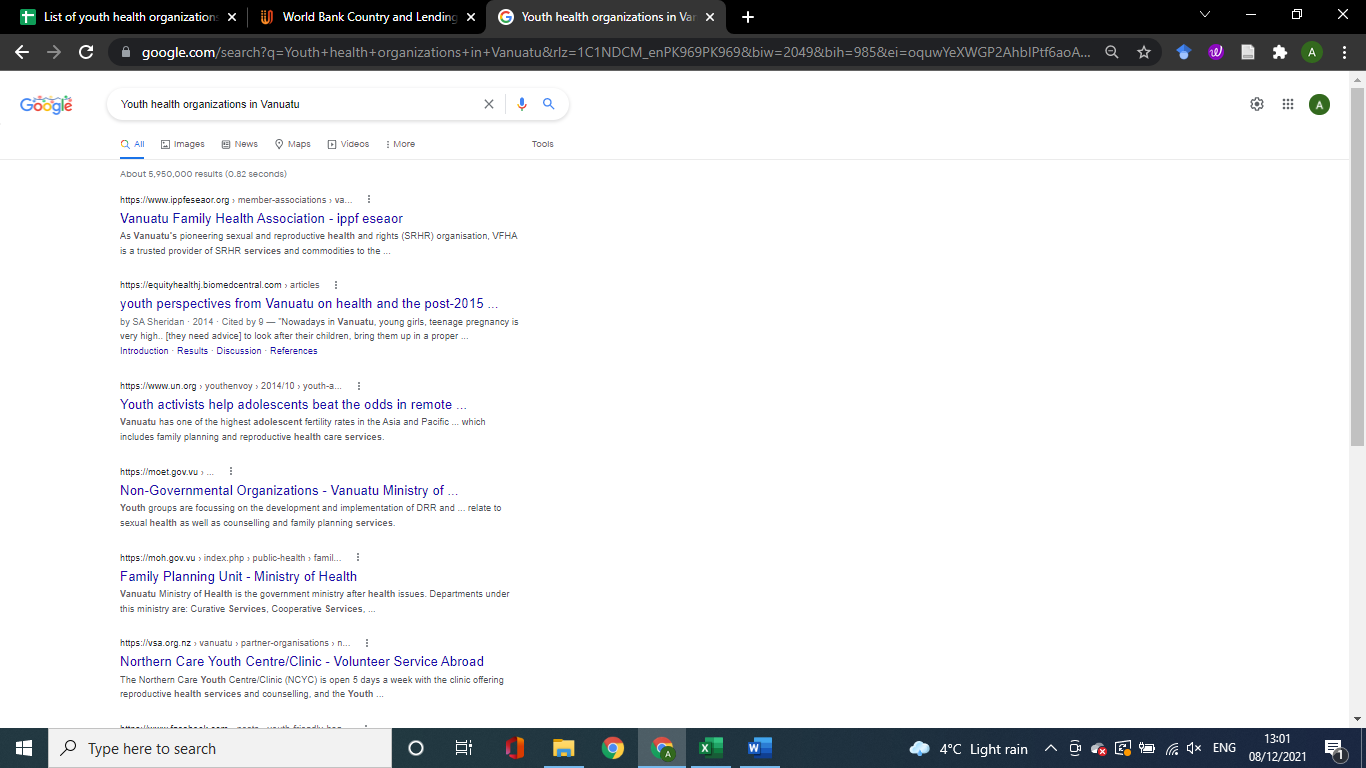 |
| 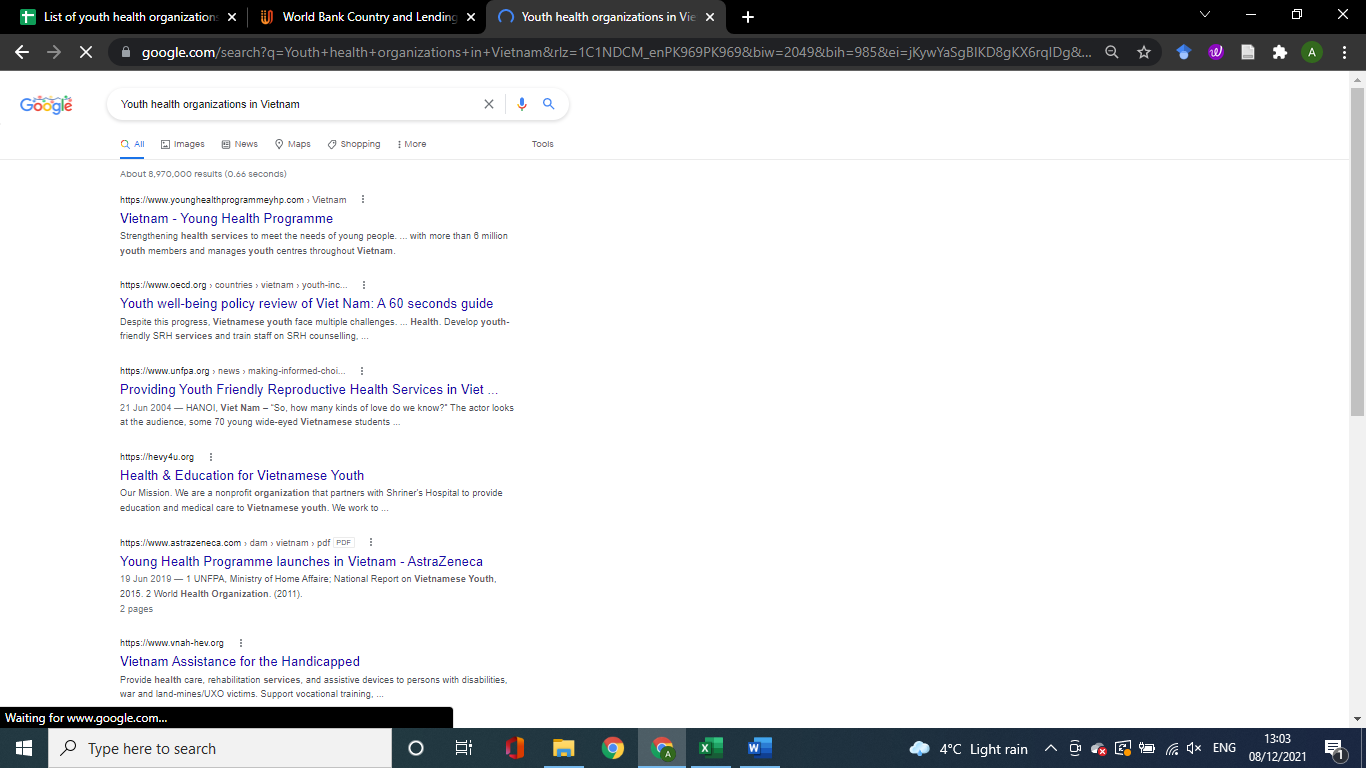 |
| 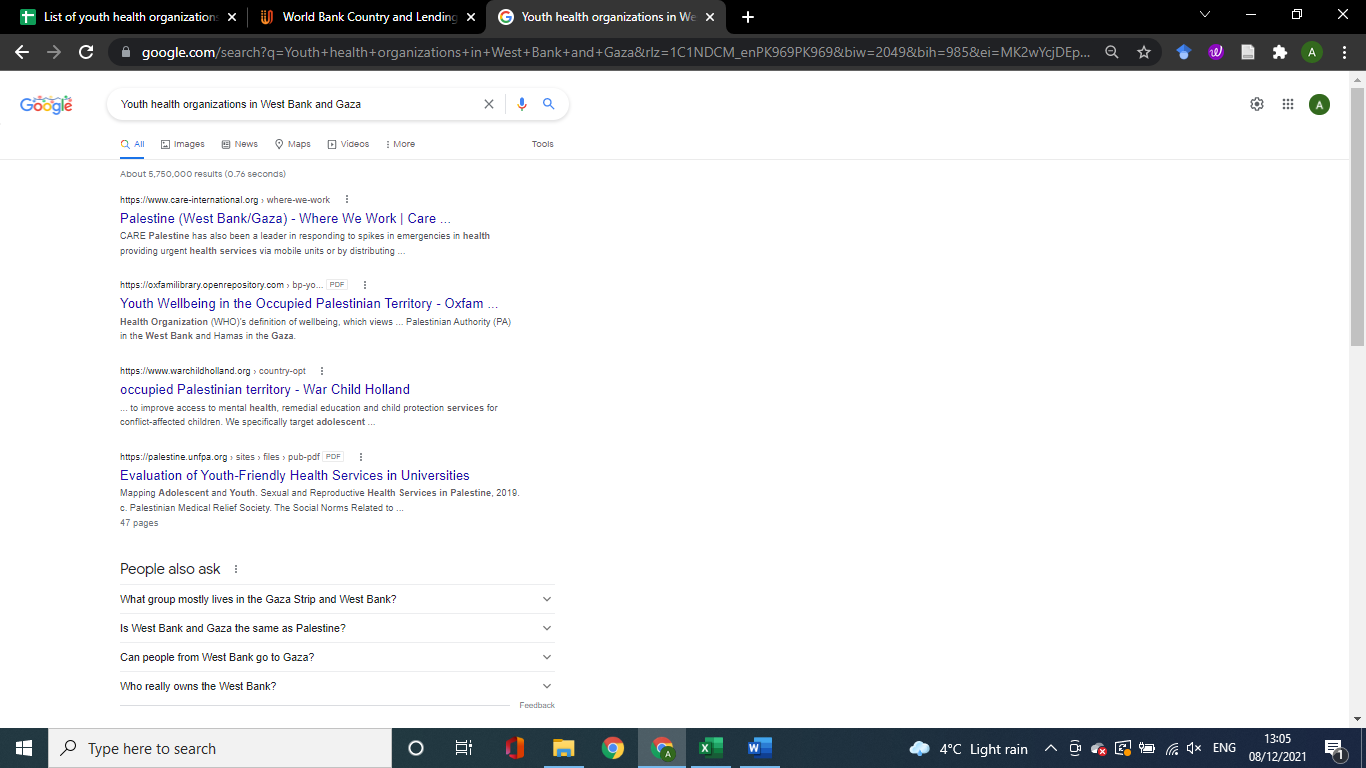 |
| 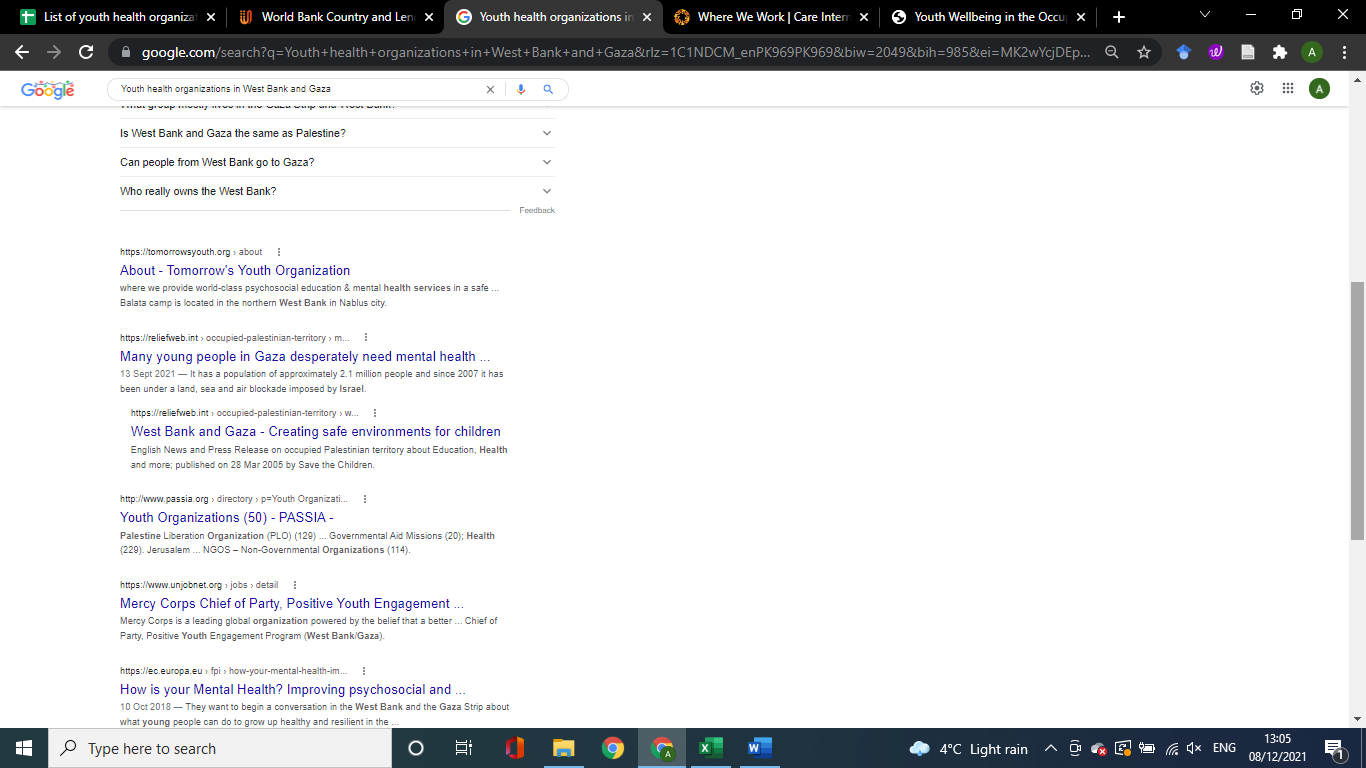 |
| 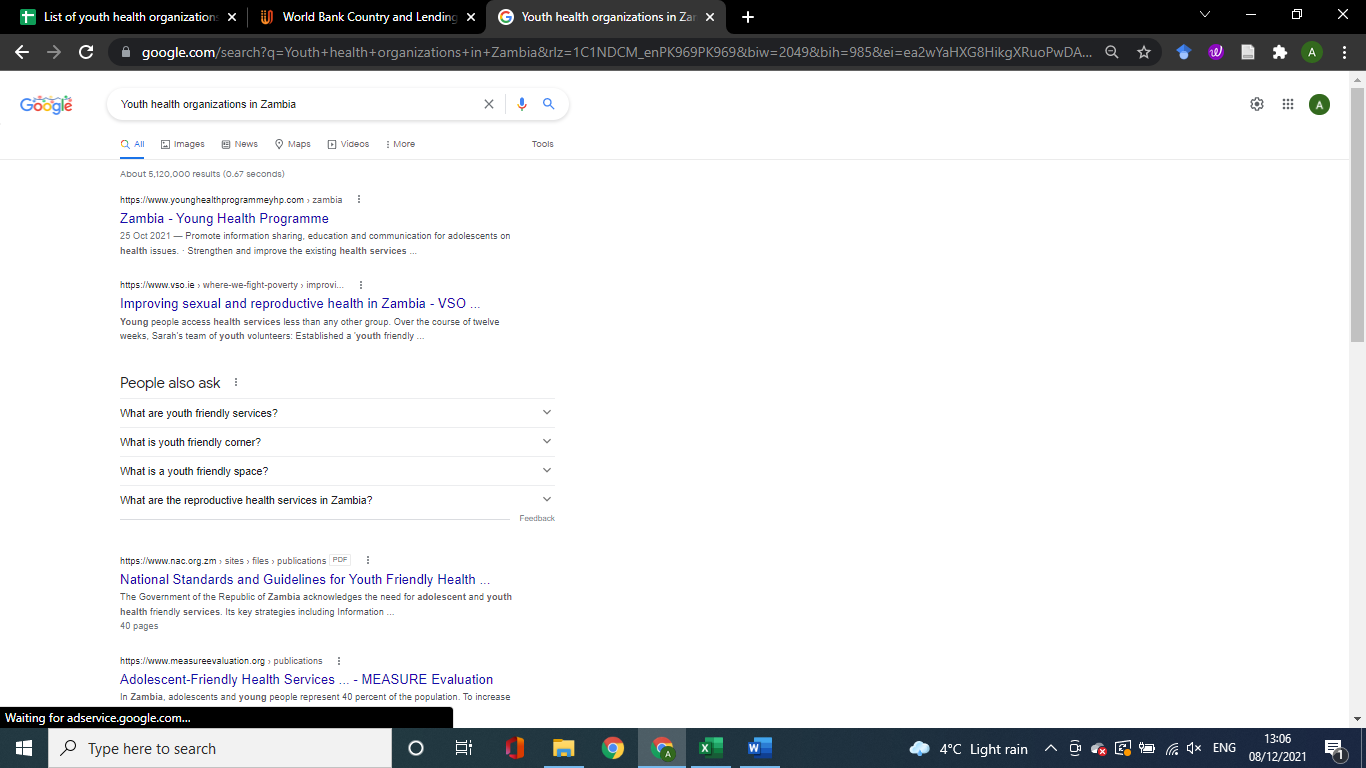 |
| 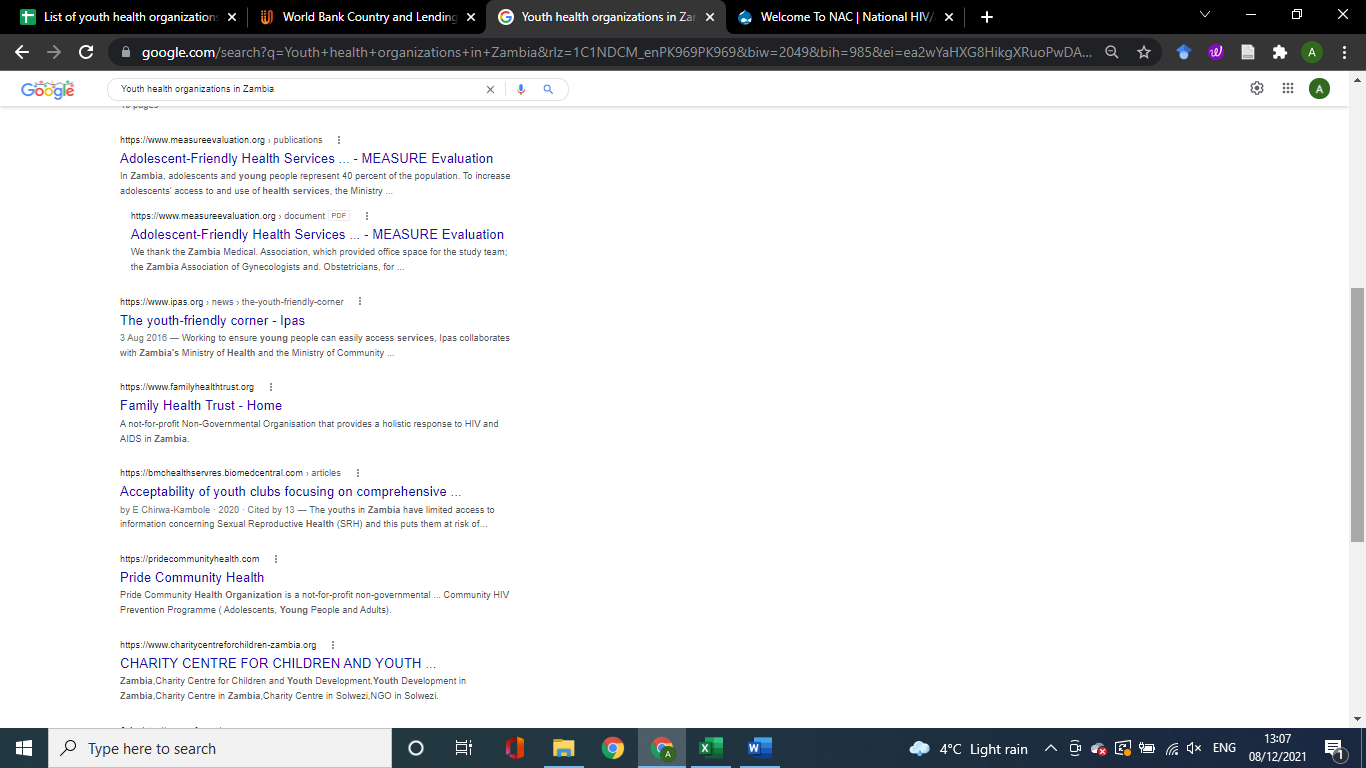 |
| 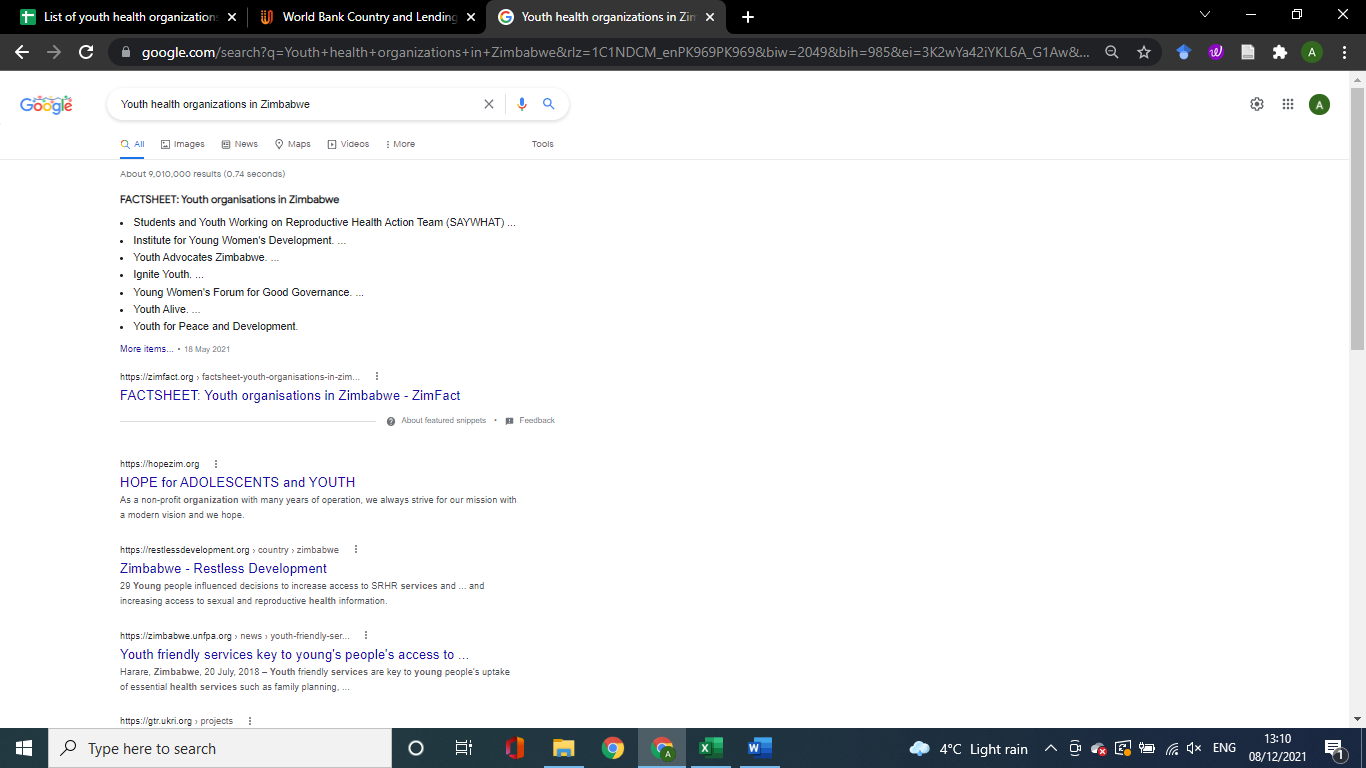 |
| 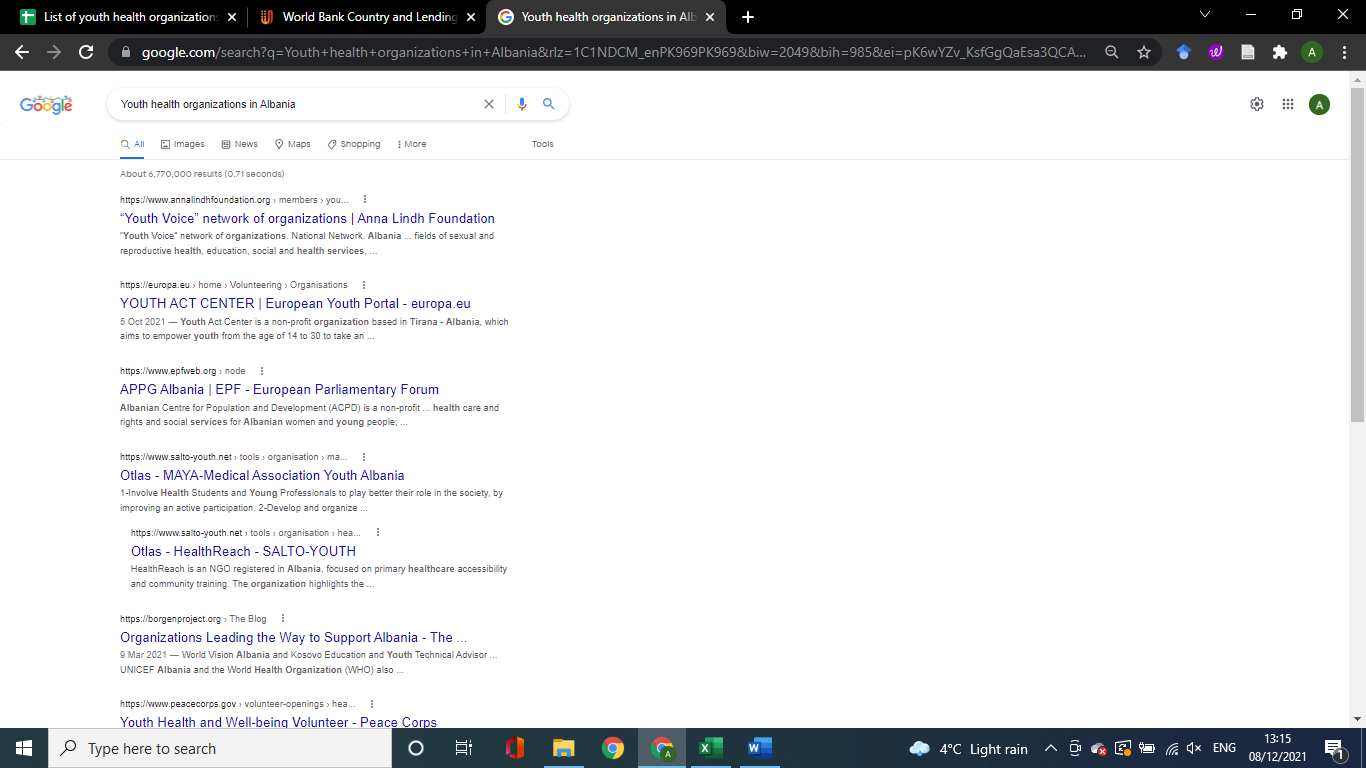 |
|  |
|  |
|  |
|  |
|  |
|  |
|  |
|  |
|  |
|  |
|  |
|  |
|  |
|  |
|  |
|  |
|  |
|  |
|  |
|  |
|  |
|  |
|  |
|  |
|  |
|  |
|  |
|  |
|  |
|  |
|  |
|  |
|  |
|  |
|  |
|  |
|  |
|  |
|  |
|  |
|  |
|  |
|  |
|  |
|  |
|  |
|  |
|  |
|  |
|  |
|  |
|  |
|  |
|  |
|  |
|  |
|  |
|  |
|  |
|  |
|  |
|  |
|  |
|  |
|  |
|  |
|  |
|  |
|  |
|  |
|  |
|  |
|  |
|  |
|  |
|  |
|  |
|  |
|  |

Supplement: Supplementary file 1 — Supporting information. [file HEX-27-e13980-s001.zip › Search record and results/Other sources/Websites of health organizations/Google search for 137 LMICs/Screenshots - Country wise list for LMICs organizations (1 per country).docx]
